# Supplementary material for: The role of mHealth intervention to improve maternal and child health: A provider-based qualitative study in Southern Ethiopia
Source: PLoS One. 2024 Feb 8;19(2):e0295539. doi: 10.1371/journal.pone.0295539 (PMC10852240; doi:10.1371/journal.pone.0295539)
Supplement: S1 Checklist — (DOCX) [file pone.0295539.s001.docx]

STROBE Statement—checklist of items that should be included in reports of observational studies

|  | Item No. | Recommendation | | Page  No. | Relevant text from manuscript |
| --- | --- | --- | --- | --- | --- |
| **Title and abstract** | 1 | 1. Indicate the study’s design with a commonly used term in the title or the abstract  - What do health professionals think about the role of mHealth in improving maternal and child health: A provider-based qualitative study in Southern Ethiopia | | 1 |  |
|  |  | 1. Provide in the abstract an informative and balanced summary of what was done and what was found   Introduction: Maternal and child mortality has remained high in developing regions such as Southern Ethiopia due to poor maternal and child health. Technologies such as mobile applications in health may be an opportunity to reduce maternal and child mortality because they can improve access to information. Therefore, the main aim of this study was to explore the role of mHealth in improving maternal and child health in Southern Ethiopia.  Methods: This study employed a qualitative study design to explore the role of mHealth in improving maternal and child health among health professionals in Southern Ethiopia from December 2022 to March 2023. We conducted nine in-depth interviews, six key informants’ in-depth interviews, and four focused group discussions among health professionals. This is followed by thematic analyses to synthesize the collected evidence.  Results: The results are based on 226 quotations, 5 major themes, and 24 subthemes. The study participants discussed the possible acceptance of mHealth in terms of its fitness in the existing health system, its support to health professionals, and its importance in improving maternal and child health. The participants’ ascertained the importance of awareness creation before the implementation mHealth among women, families, communities, and providers. They reported the importance of mHealth for mothers, and health professionals, and for the effectiveness of mHealth services. The participants stated that the main challenges related to acceptance, awareness, negligence, readiness, and workload. However, they also suggested strategic solutions such as using family support, provider support, mothers’ forums, and community forums.  Conclusion: The evidence generated during this analysis is important information for program implementations and can inform policy-making. It is especially important for the planned intervention to introduce mHealth in Southern Ethiopia. Planners, decision-makers, and researchers can use it in mobile technology-related interventions. For challenges identified, we recommend solution-identified-based interventions and quality studies. | | 2 |  |
| Introduction | | | | |  |
| Background/rationale | 2 | | Explain the scientific background and rationale for the investigation being reported   - Maternal and child mortality has remained high in developing regions such as Southern Ethiopia due to poor maternal and child health. Technologies such as mobile applications in health may be an opportunity to reduce maternal and child mortality because they can improve access to information. - Implementation attempts without the attention of the caregivers might be difficult, considering data security and interoperability between data collection, information use for care, and medical records. These concerns can be improved by giving attention to information from health caregivers. Therefore, this study aims to understand the health professionals’ perspective of mHealth implementation to improve maternal and child health by performing qualitative interviews. This may provide information on the possible future successful implementation strategies of mHealth in Southern Ethiopia. |  | 4 & 5 |
| Objectives | 3 | | State specific objectives, including any prespecified hypotheses   - The main aim of this study was to explore the role of mHealth in improving maternal and child health in Southern Ethiopia. | 2&5 |  |
| Methods | | | | |  |
| Study design | 4 | | Present key elements of study design early in the paper   - Health institution based qualitative study design | 5 |  |
| Setting | 5 | | Describe the setting, locations, and relevant dates, including periods of recruitment, exposure, follow-up, and data collection   - The South Nation Nationalities Peoples’ Region (SNNPR) is one of the elven regions in Ethiopia. The region is home to more than 55 Ethnic groups living in different administrative and cities.  The population of the southern Ethiopian region is 11,426,000. Half of the population are living under the poverty line and it is known for its high fertility rate, and higher infant and maternal mortality next to Somali and Afar | 5 |  |
| Participants | 6 | | (*a*) *Cohort study*—Give the eligibility criteria, and the sources and methods of selection of participants. Describe methods of follow-up  *Case-control study*—Give the eligibility criteria, and the sources and methods of case ascertainment and control selection. Give the rationale for the choice of cases and controls  *Cross-sectional study*—Give the eligibility criteria, and the sources and methods of selection of participants   - The source population for this study is all health professionals in all primary care facilities in the study area. The targets for the focused group discussion, in-depth interview, and key informants’ in-depth interview were physicians, public health officers (higher positions professionals next to general practitioners in Ethiopia’s health system), nurses (all types), midwifery, and health extension workers (HEWs - community health workers in the Ethiopian context). The key informants are the facility leaders and HEWs coordinators. The primary health care facilities with high achievement in maternal and child service have already been included in the overall project. The FGDs were composed of physicians, public health officers, nurses, and midwives for facility workers. Health extension workers or community health workers were included in a separate FGD to avoid the dominance of onsite staff. The relationship between health extension workers and onsite workers is a supervisor-worker relationship. | 6 |  |
|  |  |  | (*b*) *Cohort study*—For matched studies, give matching criteria and number of exposed and unexposed  *Case-control study*—For matched studies, give matching criteria and the number of controls per case |  |  |
| Variables | 7 | | Clearly define all outcomes, exposures, predictors, potential confounders, and effect modifiers. Give diagnostic criteria, if applicable  **Readiness:** This is the ability of health institutions to provide all necessary resources to implement mHealth to improve child and maternal health  **The role of mHealth to improve maternal and child health:** This is refers to the use of mobile phones, personal digital assistants (PDAs), patient monitoring devices, and other Information and Communication Technologies (ICT) to support and deliver maternal and child health healthcare services.  **Perceived benefits and challenges:** are positive**beliefs of health professionals about the extent that mHealth can improve maternal and child health by supporting their service provision and acknowledging the perceived challenges that may threaten its success.** | 7 |  |
| Data sources/ measurement | 8* | | For each variable of interest, give sources of data and details of methods of assessment (measurement). Describe comparability of assessment methods if there is more than one group   - This qualitative with data source of health professionals working in conveniently selected health institutions in Southern Ethiopia. Inferences and comparability are not possible in qualitative studies | *6* |  |
| Bias | 9 | | Describe any efforts to address potential sources of bias   - This study is based on the convenient design and was only conducted in Southern Ethiopia with a limited number of participants. As such, it only shows the view of the study participants in their local context. Trying taking the view of participants as general truth could possible introduce biases | 6 & 22 |  |
| Study size | 10 | | Explain how the study size was arrived at  We conducted four FGDs with a maximum number of participants of eight and a minimum of three. In addition, semi-structured interview guides such as eight in-depth interviews and six key informant in-depth interviews were also conducted. Interviews are key to understanding various perspectives of realities, and ordinary and extraordinary real-life events that health professionals may not speak about publically. We conveniently included eight health facilities (two primary hospitals, four health canters, and two health posts) to obtain the participants. All adult age group staff that have experience of at least one year in providing the services related to mothers and children were included conveniently. Participants were recruited from expanded program of immunization (EPI), Mother and Child Health (MCH), delivery, antenatal care, postnatal care, gynaecology, outpatient patient department, family planning, health posts, and under five year old care centres. | 6 & 7 |  |

Continued on next page

| Quantitative variables | 11 | Explain how quantitative variables were handled in the analyses. If applicable, describe which groupings were chosen and why   - Not applicable |  |  |
| --- | --- | --- | --- | --- |
| Statistical methods | 12 | 1. Describe all statistical methods, including those used to control for confounding  - This study is a qualitative study and just used Atlas.ti. Version 23 to condense the data |  | 8 |
|  |  | 1. Describe any methods used to examine subgroups and interactions  - Not applicable, we just used themes and subthemes |  | 8 |
|  |  | 1. Explain how missing data were addressed  - Not applicable |  |  |
|  |  | 1. *Cohort study*—If applicable, explain how loss to follow-up was addressed  - Not applicable   *Case-control study*—If applicable, explain how matching of cases and controls was addressed   - Not applicable   *Cross-sectional study*—If applicable, describe analytical methods taking account of sampling strategy   - The analysis only accounted codes which condensed into main and subthemes |  | 8 |
|  |  | (*e*) Describe any sensitivity analyses |  |  |
| Results | | | | |
| Participants | 13* | 1. Report numbers of individuals at each stage of study—eg numbers potentially eligible, examined for eligibility, confirmed eligible, included in the study, completing follow-up, and analysed  - Not applicable, this qualitative study that followed level of information saturation to capture maximum data |  |  |
|  |  | 1. Give reasons for non-participation at each stage  - Not applicable, all approached health professionals were participated |  |  |
|  |  | 1. Consider use of a flow diagram  - Not applicable, the follow diagram is provided as a supplementary file considering its relevance |  |  |
| Descriptive data | 14* | 1. Give characteristics of study participants (eg demographic, clinical, social) and information on exposures and potential confounders  - Provided in Table 1 |  | 9 |
|  |  | 1. Indicate number of participants with missing data for each variable of interest  - Not applicable |  |  |
|  |  | 1. *Cohort study*—Summarise follow-up time (eg, average and total amount)  - Not applicable |  |  |
| Outcome data | 15* | *Cohort study*—Report numbers of outcome events or summary measures over time   - Not applicable |  |  |
|  |  | *Case-control study—*Report numbers in each exposure category, or summary measures of exposure   - Not applicable |  |  |
|  |  | *Cross-sectional study—*Report numbers of outcome events or summary measures   - Not applicable |  |  |
| Main results | 16 | 1. Give unadjusted estimates and, if applicable, confounder-adjusted estimates and their precision (eg, 95% confidence interval). Make clear which confounders were adjusted for and why they were included  - Not applicable |  |  |
|  |  | 1. Report category boundaries when continuous variables were categorized  - Not applicable |  |  |
|  |  | 1. If relevant, consider translating estimates of relative risk into absolute risk for a meaningful time period  - Not applicable |  |  |

Continued on next page

| Other analyses | 17 | Report other analyses done—eg analyses of subgroups and interactions, and sensitivity analyses   - Not applicable, no other analysis |  |  |
| --- | --- | --- | --- | --- |
| Discussion | | | | |
| Key results | 18 | Summarise key results with reference to study objectives   - Health professionals also reported that mHealth could ease their tasks and modernize their services to clients.  They believe there could be a substantial improvement in the uptake of maternal and child healthcare and the workload of healthcare providers. They especially think that mHealth could reduce the time for supportive supervision, reporting, capturing data, and monitoring time especially, for community health workers. This shows that healthcare providers are willing to accept the introduction of mHealth into the current healthcare system, especially, to improve maternal and child health. The participants emphasized the importance of awareness creation for the community, families, mothers, and health professionals themselves for the success of mHealth. They also provided strategies such as using the Health Development Armies (HDA), mothers' forums, community voluntary health teams, community mobilization, community wings forums, and 1 to 5 networks, which are currently the base for community-based activities (**objective 1**) - Finally, participants questioned the current readiness of health institutions to implement mHealth, resource limitations, and continuity after implementation. Many previous studies acknowledge the presence of these challenges but also put substantial evidence of mHealth success in the face of the challenges(**objective 2**) - Although participants discussed many benefits and strategies of mHealth interventions, they also named possible challenges. For these challenges, they underlined the need for key solutions such as involving husbands, families, and communities in mHealth-based service provision. If the husband and family members are not aware that the wife is receiving some messages, they may become suspicious. The additional challenge is women’s lack of education to read messages. The women may use other people to read messages; however, this might reduce the women’s comfort due to the lack of privacy. The woman’s capacity to adapt to technology and her ability to manipulate mobile phones might add further challenges (**objective 3**) - The participants also provided strategic solutions for the reported challenges. They suggested that awareness among husbands, family members, and the community might reduce the challenges. The use of the local language and Golmasa Timhert (adult education) could enable mothers to read messages. Furthermore, the participants suggested unique strategies such as using HDA, voluntary health teams, community forums, community wing forums, HEWs, 1 to 5 networks, community-wide mobilization, and health professional support to improve the challenges. Other solutions such as training, technical support, economic support, creating an easy-to-use system at both ends and contextual management, and defining implementation strategies. The available literature shows consistency with our participant’s opinions (35,43). Participants are confident that the solutions they suggested are the possible remedy for the challenges and encourage implementation. (**objective 4**) |  | 21 & 22 |
| Limitations | 19 | Discuss limitations of the study, taking into account sources of potential bias or imprecision. Discuss both direction and magnitude of any potential bias   - A limitation of this study is that it was conducted in Southern Ethiopia with a limited number of participants. As such, it only shows the view of the study participants in their local context. Despite the limitations, this study provided substantial evidence on challenges and their remedies to implement mHealth to improve maternal and child health. |  | 22 |
| Interpretation | 20 | Give a cautious overall interpretation of results considering objectives, limitations, multiplicity of analyses, results from similar studies, and other relevant evidence   - The finding from this study should be interpreted only in light of local context |  | 22 |
| Generalisability | 21 | Discuss the generalisability (external validity) of the study results   - The study has high internal validity but poor external validity as per the nature of design applied. |  |  |
| Other information | |  | | |
| Funding | 22 | Give the source of funding and the role of the funders for the present study and, if applicable, for the original study on which the present article is based   - This study is funded by Arba Minch University Research Directorate - The fund was provided as a total to cover the upcoming trial and all objectives under the the large doctoral project |  | 23 |

*Give information separately for cases and controls in case-control studies and, if applicable, for exposed and unexposed groups in cohort and cross-sectional studies.

**Note:** An Explanation and Elaboration article discusses each checklist item and gives methodological background and published examples of transparent reporting. The STROBE checklist is best used in conjunction with this article (freely available on the Web sites of PLoS Medicine at http://www.plosmedicine.org/, Annals of Internal Medicine at http://www.annals.org/, and Epidemiology at http://www.epidem.com/). Information on the STROBE Initiative is available at www.strobe-statement.org.
