# Supplementary material for: The role of mHealth intervention to improve maternal and child health: A provider-based qualitative study in Southern Ethiopia
Source: PLoS One. 2024 Feb 8;19(2):e0295539. doi: 10.1371/journal.pone.0295539 (PMC10852240; doi:10.1371/journal.pone.0295539)
Supplement: S1 File — (ZIP) [file pone.0295539.s003.zip › supplementars evidences/Aranged codes with quotation in each theme.docx]

**ACCEPTABILITY THEMES**

**Acceptability: Fitting to existing health system**

*"It fits very well, but I think the process will take a long time to adapt"* [1:20 ¶ 32 in interview analysis](https://go.atlasti.com/32e35350-0ea2-49d6-8d06-20d1c3d30273/documents/b5c0cb13-2834-41fe-98e2-f35ea219267a/quotations/ac08cabf-73b6-4985-a168-908a965130e9)

*"I think it can go with the previous service"* [1:27 ¶ 36 in interview analysis](https://go.atlasti.com/32e35350-0ea2-49d6-8d06-20d1c3d30273/documents/b5c0cb13-2834-41fe-98e2-f35ea219267a/quotations/e8663888-f440-4f89-8d4e-ec02ea7df3c3)

*"There is no culture to my knowledge that can hinder the service"* [1:32 ¶ 38 in interview analysis](https://go.atlasti.com/32e35350-0ea2-49d6-8d06-20d1c3d30273/documents/b5c0cb13-2834-41fe-98e2-f35ea219267a/quotations/95d9ca78-e78e-4c97-bfea-3f2da3e2e903)

*"I do not think there is any opposing culture anywhere"* [1:33 ¶ 39 in interview analysis](https://go.atlasti.com/32e35350-0ea2-49d6-8d06-20d1c3d30273/documents/b5c0cb13-2834-41fe-98e2-f35ea219267a/quotations/5173d488-3eb9-481b-8bc1-8786bde39758)

*"No culture in opposite to this service"* [2:30 ¶ 46 in KII](https://go.atlasti.com/32e35350-0ea2-49d6-8d06-20d1c3d30273/documents/504671be-e9ef-4f58-8b5d-807935cb16a1/quotations/c99580d8-ad2c-4262-8257-376590281bd2)

*"Every culture is improving through technology and it is everywhere, so whatever technology is accepted no opposition"* [2:31 ¶ 47 in KII](https://go.atlasti.com/32e35350-0ea2-49d6-8d06-20d1c3d30273/documents/504671be-e9ef-4f58-8b5d-807935cb16a1/quotations/bb117dbe-a2c7-41ec-b77e-f2730a2fc5f3)

*"I hope they will accept easily because this is the part of health communication and no one should because there nothing new here"* [2:45 ¶ 65 in KII](https://go.atlasti.com/32e35350-0ea2-49d6-8d06-20d1c3d30273/documents/504671be-e9ef-4f58-8b5d-807935cb16a1/quotations/d15e0216-6f11-44fd-b647-0850f9a1fa0a)

*"Women always want something that allows better service so this can be important"* [3:7 ¶ 6 in Group discussion](https://go.atlasti.com/32e35350-0ea2-49d6-8d06-20d1c3d30273/documents/2569dddf-9aa1-42e1-9ece-a8d4dbbb0f25/quotations/2f462796-e106-47a8-b678-983f3bfd35d2)

*"They community as a whole may see this positively and acknowledge the real effort"* [3:14 ¶ 8 in Group discussion](https://go.atlasti.com/32e35350-0ea2-49d6-8d06-20d1c3d30273/documents/2569dddf-9aa1-42e1-9ece-a8d4dbbb0f25/quotations/8ac83178-5c28-4921-af46-32b346da812f)

**Acceptability: Improve care**

*"Improve the existing service in the positive direction"* [1:29 ¶ 37 in interview analysis](https://go.atlasti.com/32e35350-0ea2-49d6-8d06-20d1c3d30273/documents/b5c0cb13-2834-41fe-98e2-f35ea219267a/quotations/ef7d0444-227f-4fd1-949c-0376e79dfcbd)

*"We have previous requirements of following patients, so this strengthens the previous idea in practice. This time I don’t think anyone will oppose this"* [2:39 ¶ 58 in KII](https://go.atlasti.com/32e35350-0ea2-49d6-8d06-20d1c3d30273/documents/504671be-e9ef-4f58-8b5d-807935cb16a1/quotations/c37f4f27-9152-4bdb-84fd-e5ebbd8dccf5)

**Acceptability: support professionals**

*"If this is to support but not contradict with HEWs work it is important"* [1:47 ¶ 54 in interview analysis](https://go.atlasti.com/32e35350-0ea2-49d6-8d06-20d1c3d30273/documents/b5c0cb13-2834-41fe-98e2-f35ea219267a/quotations/7b5c0d01-c5b2-4d36-be90-6e3cdc3df022)

*"but she may also have to come to health professional for further investigations beside information access."* [1:52 ¶ 58 in interview analysis](https://go.atlasti.com/32e35350-0ea2-49d6-8d06-20d1c3d30273/documents/b5c0cb13-2834-41fe-98e2-f35ea219267a/quotations/e453a4f0-0753-470f-a82e-50d5b43d2324)

*"I think our institution workers will welcome this more than anything. This is modernizing what everyone is eager to have"* [2:13 ¶ 25 in KII](https://go.atlasti.com/32e35350-0ea2-49d6-8d06-20d1c3d30273/documents/504671be-e9ef-4f58-8b5d-807935cb16a1/quotations/f7200ffc-6795-4aa5-8cdf-f0f2c5001ea2)

*"Health professionals’ role should be high to make it accepted by the mothers and community"* [2:16 ¶ 28 in KII](https://go.atlasti.com/32e35350-0ea2-49d6-8d06-20d1c3d30273/documents/504671be-e9ef-4f58-8b5d-807935cb16a1/quotations/36fcf8d9-222d-4e85-9081-88623722991c)

*"this can easy our workers job, they will happy to accept as peacefully as anything"* [2:17 ¶ 29 in KII](https://go.atlasti.com/32e35350-0ea2-49d6-8d06-20d1c3d30273/documents/504671be-e9ef-4f58-8b5d-807935cb16a1/quotations/c7a16088-13b9-49a0-867d-6b247f6137b8)

*"We have previous requirements of following patients, so this strengthens the previous idea in practice. This time I don’t anyone will oppose this"* [2:39 ¶ 58 in KII](https://go.atlasti.com/32e35350-0ea2-49d6-8d06-20d1c3d30273/documents/504671be-e9ef-4f58-8b5d-807935cb16a1/quotations/c37f4f27-9152-4bdb-84fd-e5ebbd8dccf5)

*"Health education is necessary and our community is active for such new technology based services"* [2:41 ¶ 62 in KII](https://go.atlasti.com/32e35350-0ea2-49d6-8d06-20d1c3d30273/documents/504671be-e9ef-4f58-8b5d-807935cb16a1/quotations/6c689e82-4a54-418c-8896-2dfbf539a97a)

*"Making people understand the service objectives especially health professionals"* [2:44 ¶ 64 in KII](https://go.atlasti.com/32e35350-0ea2-49d6-8d06-20d1c3d30273/documents/504671be-e9ef-4f58-8b5d-807935cb16a1/quotations/ff214633-49a9-470b-b9a5-ab3a5fbb4e62)

*"It is interesting to use the technology at this level, so am happy. Mothers usually fear whatever health professionals order and can be comply when they ordered to meet on phone for the next information."* [3:31 ¶ 17 in Group discussion](https://go.atlasti.com/32e35350-0ea2-49d6-8d06-20d1c3d30273/documents/2569dddf-9aa1-42e1-9ece-a8d4dbbb0f25/quotations/a11b983c-34ef-4f6a-aba7-d89fe7fcbb3d)

*"accept the technology as the part of easing their work concerns. Awareness creation among mothers and community is critical"* [3:55 ¶ 40 in Group discussion](https://go.atlasti.com/32e35350-0ea2-49d6-8d06-20d1c3d30273/documents/2569dddf-9aa1-42e1-9ece-a8d4dbbb0f25/quotations/358bd084-ab8c-4693-bb57-84e24d7766e9)

**Acceptability: Technology care**

*"previous services are not different but this is automated, evidenced, and easy to apply"* [1:22 ¶ 33 in interview analysis](https://go.atlasti.com/32e35350-0ea2-49d6-8d06-20d1c3d30273/documents/b5c0cb13-2834-41fe-98e2-f35ea219267a/quotations/08ad50aa-cb7a-4d8c-9a7c-314879e39ebc)

*" I feel happy because it is modernized"* [2:2 ¶ 10 in KII](https://go.atlasti.com/32e35350-0ea2-49d6-8d06-20d1c3d30273/documents/504671be-e9ef-4f58-8b5d-807935cb16a1/quotations/f7752ca1-b9c5-4018-bd0d-a4b5c95c3e55)

*"Am happy that finally we will have technology to support our client especially for appoint"* [2:5 ¶ 14 in KII](https://go.atlasti.com/32e35350-0ea2-49d6-8d06-20d1c3d30273/documents/504671be-e9ef-4f58-8b5d-807935cb16a1/quotations/c022fb44-a0b9-4198-8d81-857008c15908)

*"I think they will be happy. Everyone love technology whatever it is. Everyone will accept, we are part of the technology"* [2:12 ¶ 24 in KII](https://go.atlasti.com/32e35350-0ea2-49d6-8d06-20d1c3d30273/documents/504671be-e9ef-4f58-8b5d-807935cb16a1/quotations/fa976403-6995-40df-a724-a27a1a4d9268)

*"People accept as something modern and will be happy"* [2:32 ¶ 50 in KII](https://go.atlasti.com/32e35350-0ea2-49d6-8d06-20d1c3d30273/documents/504671be-e9ef-4f58-8b5d-807935cb16a1/quotations/4b4532ac-b6fc-4330-8f82-66a2d0c9c976)

*"People currently eager to have new technology so if no payment for the technology no everyone will be happy to have this (mothers). It can also reduce excessive human power"* [2:37 ¶ 56 in KII](https://go.atlasti.com/32e35350-0ea2-49d6-8d06-20d1c3d30273/documents/504671be-e9ef-4f58-8b5d-807935cb16a1/quotations/c8af1197-886d-478f-8732-f318cfbbf9b4)

*"I think it will not be possible after sometime to live without or work without technology so will be happy to see"* [2:38 ¶ 57 in KII](https://go.atlasti.com/32e35350-0ea2-49d6-8d06-20d1c3d30273/documents/504671be-e9ef-4f58-8b5d-807935cb16a1/quotations/20dcb77e-dbc4-4c7a-bead-2a31cc080ecf)

*"Health education is necessary and our community is active for such new technology based services"* [2:41 ¶ 62 in KII](https://go.atlasti.com/32e35350-0ea2-49d6-8d06-20d1c3d30273/documents/504671be-e9ef-4f58-8b5d-807935cb16a1/quotations/6c689e82-4a54-418c-8896-2dfbf539a97a)

*"Women always want something that allows better service so this can be important"* [3:7 ¶ 6 in Group discussion](https://go.atlasti.com/32e35350-0ea2-49d6-8d06-20d1c3d30273/documents/2569dddf-9aa1-42e1-9ece-a8d4dbbb0f25/quotations/2f462796-e106-47a8-b678-983f3bfd35d2)

*"They community as a whole may see this positively and acknowledge the real effort"* [3:14 ¶ 8 in Group discussion](https://go.atlasti.com/32e35350-0ea2-49d6-8d06-20d1c3d30273/documents/2569dddf-9aa1-42e1-9ece-a8d4dbbb0f25/quotations/8ac83178-5c28-4921-af46-32b346da812f)

*"It is interesting to use the technology at this level, so am happy. Mothers usually fear whatever health professionals order and can be comply when they ordered to meet on phone for the next information."* [3:31 ¶ 17 in Group discussion](https://go.atlasti.com/32e35350-0ea2-49d6-8d06-20d1c3d30273/documents/2569dddf-9aa1-42e1-9ece-a8d4dbbb0f25/quotations/a11b983c-34ef-4f6a-aba7-d89fe7fcbb3d)

*"accept the technology as the part of easing their work concerns. Awareness creation among mothers and community is critical"* [3:55 ¶ 40 in Group discussion](https://go.atlasti.com/32e35350-0ea2-49d6-8d06-20d1c3d30273/documents/2569dddf-9aa1-42e1-9ece-a8d4dbbb0f25/quotations/358bd084-ab8c-4693-bb57-84e24d7766e9)

*"Expectation of mother to get SMS to take service, and otherwise consideration of no risk"* [3:69 ¶ 56 in Group discussion](https://go.atlasti.com/32e35350-0ea2-49d6-8d06-20d1c3d30273/documents/2569dddf-9aa1-42e1-9ece-a8d4dbbb0f25/quotations/b18f3632-006c-465d-a02c-e54cf51c045e)

**AWARENESS THEMES**

**Awareness creation: community awareness**

*"If one mother served all will need to take that will also motivate households to prepare mobile for every mother who get pregnant"* [1:1 ¶ 16 in interview analysis](https://go.atlasti.com/32e35350-0ea2-49d6-8d06-20d1c3d30273/documents/b5c0cb13-2834-41fe-98e2-f35ea219267a/quotations/e46a4f62-15e2-41de-b088-3073daec5825)

*"This is because of the need of familiarization or awareness creation both in the community and among health professionals, which will take some time"* [1:21 ¶ 32 in interview analysis](https://go.atlasti.com/32e35350-0ea2-49d6-8d06-20d1c3d30273/documents/b5c0cb13-2834-41fe-98e2-f35ea219267a/quotations/95585d11-41be-4405-9757-eea72bcfe97e)

*"Health development community members are the chance to convince mother and monitor. Currently due to the sanction we are suffering a lot, but when everything become alright, I think this the best thing our community deserves."* [2:23 ¶ 36 in KII](https://go.atlasti.com/32e35350-0ea2-49d6-8d06-20d1c3d30273/documents/504671be-e9ef-4f58-8b5d-807935cb16a1/quotations/b63143b7-2877-42f9-873e-ebf9e615ef96)

*"Encourage women education most importantly in the whole society. Use of education as a tool to have good pregnancy."* [2:34 ¶ 53 in KII](https://go.atlasti.com/32e35350-0ea2-49d6-8d06-20d1c3d30273/documents/504671be-e9ef-4f58-8b5d-807935cb16a1/quotations/f139f391-3be5-4129-b980-7f7d49a9a5bf)

*"There may be a way perhaps to change the current ignorant behavior of mothers"* [3:21 ¶ 12 in Group discussion](https://go.atlasti.com/32e35350-0ea2-49d6-8d06-20d1c3d30273/documents/2569dddf-9aa1-42e1-9ece-a8d4dbbb0f25/quotations/210adc6e-782f-4183-bcfe-6efa333335c0)

*"using mHealth consultation, providing care, or treating patient or client look special because we can do whatever when we can or at any time."* [3:40 ¶ 24 in Group discussion](https://go.atlasti.com/32e35350-0ea2-49d6-8d06-20d1c3d30273/documents/2569dddf-9aa1-42e1-9ece-a8d4dbbb0f25/quotations/609297cd-e068-4339-a7d5-19f32f238ddf)

*"After mothers forum or community mobilization our service our work may be easy through mHealth"* [3:45 ¶ 28 in Group discussion](https://go.atlasti.com/32e35350-0ea2-49d6-8d06-20d1c3d30273/documents/2569dddf-9aa1-42e1-9ece-a8d4dbbb0f25/quotations/76e193d4-52f9-4836-9cdf-f336a3c7dae3)

*"awareness in the community and improve works in those area according to the new technology"* [3:56 ¶ 41 in Group discussion](https://go.atlasti.com/32e35350-0ea2-49d6-8d06-20d1c3d30273/documents/2569dddf-9aa1-42e1-9ece-a8d4dbbb0f25/quotations/b24750ec-c5ee-469d-ab34-5b78457580b0)

**Awareness creation: family awareness**

*"I think if her husband is aware of the service, there will be no problem"* [1:30 ¶ 37 in interview analysis](https://go.atlasti.com/32e35350-0ea2-49d6-8d06-20d1c3d30273/documents/b5c0cb13-2834-41fe-98e2-f35ea219267a/quotations/7f08a728-2931-42d4-9d32-7b4636b0876b)

*"using mHealth consultation, providing care, or treating patient or client look special because we can do whatever when we can or at any time."* [3:40 ¶ 24 in Group discussion](https://go.atlasti.com/32e35350-0ea2-49d6-8d06-20d1c3d30273/documents/2569dddf-9aa1-42e1-9ece-a8d4dbbb0f25/quotations/609297cd-e068-4339-a7d5-19f32f238ddf)

*"After mothers forum or community mobilization our service our work may be easy through mHealth"* [3:45 ¶ 28 in Group discussion](https://go.atlasti.com/32e35350-0ea2-49d6-8d06-20d1c3d30273/documents/2569dddf-9aa1-42e1-9ece-a8d4dbbb0f25/quotations/76e193d4-52f9-4836-9cdf-f336a3c7dae3)

*"Reading ability. Many SMS sent by Ethio-tele. every day, so this need little awareness"* [3:64 ¶ 51 in Group discussion](https://go.atlasti.com/32e35350-0ea2-49d6-8d06-20d1c3d30273/documents/2569dddf-9aa1-42e1-9ece-a8d4dbbb0f25/quotations/3b01e81b-8a0c-4fbf-90d6-bb80bdc5da02)

**Awareness creation: provider awareness**

*"This is because of the need for familiarization or awareness creation both in the community and among health professionals, which will take some time"* [1:21 ¶ 32 in interview analysis](https://go.atlasti.com/32e35350-0ea2-49d6-8d06-20d1c3d30273/documents/b5c0cb13-2834-41fe-98e2-f35ea219267a/quotations/95585d11-41be-4405-9757-eea72bcfe97e)

*"we can also be aware and work on behavioral change for not accepting mothers"* [1:73 ¶ 74 in interview analysis](https://go.atlasti.com/32e35350-0ea2-49d6-8d06-20d1c3d30273/documents/b5c0cb13-2834-41fe-98e2-f35ea219267a/quotations/51c96e2f-7bc2-4af5-89dc-2a55e1cb12c4)

*"In Health institution awareness training for health profession, increasing positive feedback mechanism"* [2:20 ¶ 33 in KII](https://go.atlasti.com/32e35350-0ea2-49d6-8d06-20d1c3d30273/documents/504671be-e9ef-4f58-8b5d-807935cb16a1/quotations/18d33bca-725a-4cd4-9710-069789925c55)

*"They way of following success and promotion should be continuous. Mother can be promote even to use and buy mobile phone to be pregnant and this can be common practice for women in the community. They may even ask a woman why she pregnant before having a phone"* [3:26 ¶ 14 in Group discussion](https://go.atlasti.com/32e35350-0ea2-49d6-8d06-20d1c3d30273/documents/2569dddf-9aa1-42e1-9ece-a8d4dbbb0f25/quotations/8cca8d03-6291-4727-b72a-d548522be4f7)

**Awareness creation: women awareness**

*"If one mother served all will need to take that will aslo motivate households to prepare mobile for every mother get pregnant"* [1:1 ¶ 16 in interview analysis](https://go.atlasti.com/32e35350-0ea2-49d6-8d06-20d1c3d30273/documents/b5c0cb13-2834-41fe-98e2-f35ea219267a/quotations/e46a4f62-15e2-41de-b088-3073daec5825)

*"After mothers understand the service they will use and since they have access it will improve the service. First mothers need awareness after that the service can be effective"* [1:67 ¶ 69 in interview analysis](https://go.atlasti.com/32e35350-0ea2-49d6-8d06-20d1c3d30273/documents/b5c0cb13-2834-41fe-98e2-f35ea219267a/quotations/0c6a0a01-87c1-4cfb-8a0e-2f42afb1094b)

*"Consultation, treatment follow up, other health education can be given through health mHealth"* [1:69 ¶ 70 in interview analysis](https://go.atlasti.com/32e35350-0ea2-49d6-8d06-20d1c3d30273/documents/b5c0cb13-2834-41fe-98e2-f35ea219267a/quotations/9348a571-6d94-4541-a7cf-f663ad4ef4c2)

*"I do not think there will a problem because we have to teach train and aware mothers"* [1:71 ¶ 73 in interview analysis](https://go.atlasti.com/32e35350-0ea2-49d6-8d06-20d1c3d30273/documents/b5c0cb13-2834-41fe-98e2-f35ea219267a/quotations/1758a44d-5391-4673-95ec-f97656ce6438)

*"we can also be aware and work on behavioral change for not accepting mothers"* [1:73 ¶ 74 in interview analysis](https://go.atlasti.com/32e35350-0ea2-49d6-8d06-20d1c3d30273/documents/b5c0cb13-2834-41fe-98e2-f35ea219267a/quotations/51c96e2f-7bc2-4af5-89dc-2a55e1cb12c4)

*"I don’t think it will have any problem because when we tell mother “it will help her accept”. Further education is necessary before implementing anything"* [1:75 ¶ 76 in interview analysis](https://go.atlasti.com/32e35350-0ea2-49d6-8d06-20d1c3d30273/documents/b5c0cb13-2834-41fe-98e2-f35ea219267a/quotations/890e47d5-e57e-4be0-ac80-a01a583506c1)

*"Throughout mothers may need support. We can help with understanding. If the mother can read, have mobile, able to understand there will be no more obstacles"* [1:101 ¶ 100 in interview analysis](https://go.atlasti.com/32e35350-0ea2-49d6-8d06-20d1c3d30273/documents/b5c0cb13-2834-41fe-98e2-f35ea219267a/quotations/7a6a1ef8-80f2-44e1-85a5-0ade0d618562)

*"We have to teach mothers how to use mobile, what is mHealth, how to open, and others. If all input resources are fulfilled, there will be no problem"* [1:105 ¶ 105 in interview analysis](https://go.atlasti.com/32e35350-0ea2-49d6-8d06-20d1c3d30273/documents/b5c0cb13-2834-41fe-98e2-f35ea219267a/quotations/b5b6ad51-2691-4782-9f61-ca79acd7085d)

*"For mother it may need social and BCC to get it welcomed completely"* [2:14 ¶ 26 in KII](https://go.atlasti.com/32e35350-0ea2-49d6-8d06-20d1c3d30273/documents/504671be-e9ef-4f58-8b5d-807935cb16a1/quotations/afb8894e-880a-45e6-89f5-1a1245a85413)

*"Health development community members are the chance to convince mother and monitor. Currently due to the sanction we are suffering a lot, but when everything become alright, I think this the best thing our community deserves."* [2:23 ¶ 36 in KII](https://go.atlasti.com/32e35350-0ea2-49d6-8d06-20d1c3d30273/documents/504671be-e9ef-4f58-8b5d-807935cb16a1/quotations/b63143b7-2877-42f9-873e-ebf9e615ef96)

*"Encourage women education most importantly in the whole society. Use of education as a tool to have good pregnancy."* [2:34 ¶ 53 in KII](https://go.atlasti.com/32e35350-0ea2-49d6-8d06-20d1c3d30273/documents/504671be-e9ef-4f58-8b5d-807935cb16a1/quotations/f139f391-3be5-4129-b980-7f7d49a9a5bf)

*"Whatever the mother say either for refusal I think we can convince them. Everything at initial point has its own struggling and final accepted so there will be no new things here, we teach them they accep"* [3:16 ¶ 10 in Group discussion](https://go.atlasti.com/32e35350-0ea2-49d6-8d06-20d1c3d30273/documents/2569dddf-9aa1-42e1-9ece-a8d4dbbb0f25/quotations/bcee3dcd-bee2-4daa-9751-262381faac9a)

*"May be this mobile service can be accompanied with behavioral change educations"* [3:17 ¶ 12 in Group discussion](https://go.atlasti.com/32e35350-0ea2-49d6-8d06-20d1c3d30273/documents/2569dddf-9aa1-42e1-9ece-a8d4dbbb0f25/quotations/b4b3eb5b-388c-49f9-8341-b57b39dd11c9)

*"There may be a way perhaps to change the current ignorant behavior of mothers"* [3:21 ¶ 12 in Group discussion](https://go.atlasti.com/32e35350-0ea2-49d6-8d06-20d1c3d30273/documents/2569dddf-9aa1-42e1-9ece-a8d4dbbb0f25/quotations/210adc6e-782f-4183-bcfe-6efa333335c0)

*"They way of following success and promotion should be continuous. Mother can be promote even to use and buy mobile phone to be pregnant and this can be common practice for women in the community. They may even ask a woman why she pregnant before having a phone"* [3:26 ¶ 14 in Group discussion](https://go.atlasti.com/32e35350-0ea2-49d6-8d06-20d1c3d30273/documents/2569dddf-9aa1-42e1-9ece-a8d4dbbb0f25/quotations/8cca8d03-6291-4727-b72a-d548522be4f7)

*"This will create an option to counsel and follow up mothers are not counseled, have special follow up issue, and some suspicious issues"* [3:32 ¶ 19 in Group discussion](https://go.atlasti.com/32e35350-0ea2-49d6-8d06-20d1c3d30273/documents/2569dddf-9aa1-42e1-9ece-a8d4dbbb0f25/quotations/a8bd4835-0354-4fdd-a058-b57b56434b8d)

*"Consultation is what we usually do when circumstances allow us to do"* [3:39 ¶ 24 in Group discussion](https://go.atlasti.com/32e35350-0ea2-49d6-8d06-20d1c3d30273/documents/2569dddf-9aa1-42e1-9ece-a8d4dbbb0f25/quotations/1a1d0774-72c4-4af6-ac64-9a467ff4de17)

*"Using mHealth consultation, providing care, or treating patient or client look special because we can do whatever when we can or at any time."* [3:40 ¶ 24 in Group discussion](https://go.atlasti.com/32e35350-0ea2-49d6-8d06-20d1c3d30273/documents/2569dddf-9aa1-42e1-9ece-a8d4dbbb0f25/quotations/609297cd-e068-4339-a7d5-19f32f238ddf)

*"Reading ability. Many SMS sent by Ethio-tele. every day, so this need little awareness"* [3:64 ¶ 51 in Group discussion](https://go.atlasti.com/32e35350-0ea2-49d6-8d06-20d1c3d30273/documents/2569dddf-9aa1-42e1-9ece-a8d4dbbb0f25/quotations/3b01e81b-8a0c-4fbf-90d6-bb80bdc5da02)

**BENEFITS OF MHEALTH THEME**

**Benefits of mHealth: Alarming**

*"it helps when people are busy with their work to remember appointments"* [1:10 ¶ 24 in interview analysis](https://go.atlasti.com/32e35350-0ea2-49d6-8d06-20d1c3d30273/documents/b5c0cb13-2834-41fe-98e2-f35ea219267a/quotations/d7757d8c-468d-42f2-9121-1da7d28658dc)

*"Mothers may not miss their appointment because they forget i"* [1:25 ¶ 35 in interview analysis](https://go.atlasti.com/32e35350-0ea2-49d6-8d06-20d1c3d30273/documents/b5c0cb13-2834-41fe-98e2-f35ea219267a/quotations/d5f1a5a6-12f6-4056-b4be-b92d0d988bf7)

*"I think mHealth can improve access, appointment on time, availability of information for decision making increased"* [1:53 ¶ 59 in interview analysis](https://go.atlasti.com/32e35350-0ea2-49d6-8d06-20d1c3d30273/documents/b5c0cb13-2834-41fe-98e2-f35ea219267a/quotations/fcb3898d-335d-4d72-b7c0-67356cd5e50a)

*"Every system is getting digitalized so digitalizing health may have much advantage more than just appointme"* [1:68 ¶ 70 in interview analysis](https://go.atlasti.com/32e35350-0ea2-49d6-8d06-20d1c3d30273/documents/b5c0cb13-2834-41fe-98e2-f35ea219267a/quotations/ab6b89bf-1dbb-42cd-9e83-dd229d8ee603)

*"It can make the child health growth, for mothers to know danger signs, and during PNC to keep herself from unnecessary early pregnancy. It has no problem as long as the message receiver is the mother or someone oriented on the issue"* [3:27 ¶ 16 in Group discussion](https://go.atlasti.com/32e35350-0ea2-49d6-8d06-20d1c3d30273/documents/2569dddf-9aa1-42e1-9ece-a8d4dbbb0f25/quotations/af04de63-7662-4465-8278-b8ecb3463b91)

*"Appointment reminder, service on time, and reduce mortality, but before all these service awareness creation should take the first place."* [3:47 ¶ 30 in Group discussion](https://go.atlasti.com/32e35350-0ea2-49d6-8d06-20d1c3d30273/documents/2569dddf-9aa1-42e1-9ece-a8d4dbbb0f25/quotations/9827fac5-760b-4283-9670-61d95a20192f)

**Benefits of mHealth: Effective**

*"it can reduce cost, time, and other resources"* [1:48 ¶ 55 in interview analysis](https://go.atlasti.com/32e35350-0ea2-49d6-8d06-20d1c3d30273/documents/b5c0cb13-2834-41fe-98e2-f35ea219267a/quotations/9bd0bbad-5b1a-4c79-8d87-33d93ee7689d)

*"It will be very effective because it advances the services"* [1:60 ¶ 64 in interview analysis](https://go.atlasti.com/32e35350-0ea2-49d6-8d06-20d1c3d30273/documents/b5c0cb13-2834-41fe-98e2-f35ea219267a/quotations/8297d126-90c2-4246-83a1-63fa9e8c5a32)

*"I think it will be very effective because we can see the newly implemented health insurance"* [1:61 ¶ 65 in interview analysis](https://go.atlasti.com/32e35350-0ea2-49d6-8d06-20d1c3d30273/documents/b5c0cb13-2834-41fe-98e2-f35ea219267a/quotations/5b29d7f6-6636-4e30-a761-588a4617117c)

*"hope mothers will turn to use mobiles, but only after getting its importance that why health education should be crucial part of this implementation"* [1:81 ¶ 80 in interview analysis](https://go.atlasti.com/32e35350-0ea2-49d6-8d06-20d1c3d30273/documents/b5c0cb13-2834-41fe-98e2-f35ea219267a/quotations/5c9d7b77-c5d8-4eed-aae6-53392d5acf1b)

*"If all input resources are fulfilled, there will be no problem. It is not a chemical and is not harmful and it is helpful. I don’t see any obstacles because we give them by aware mothers, preparing how mother can read and all other necessary can fulfilling the service needs."* [1:98 ¶ 98 in interview analysis](https://go.atlasti.com/32e35350-0ea2-49d6-8d06-20d1c3d30273/documents/b5c0cb13-2834-41fe-98e2-f35ea219267a/quotations/b46b7098-38e9-40c3-a728-a55f3de4d793)

*"Yeah, it will have effective change on community"* [2:7 ¶ 17 in KII](https://go.atlasti.com/32e35350-0ea2-49d6-8d06-20d1c3d30273/documents/504671be-e9ef-4f58-8b5d-807935cb16a1/quotations/73f43d08-5cb1-4192-bccc-5f28703a0969)

**Benefits of mHealth: Help mothers**

*"action comes after understanding of benefits and harms"* [1:41 ¶ 47 in interview analysis](https://go.atlasti.com/32e35350-0ea2-49d6-8d06-20d1c3d30273/documents/b5c0cb13-2834-41fe-98e2-f35ea219267a/quotations/bc55914d-0865-479f-97e7-996e8e09646a)

*"Accessibility can be improved mothers get information at their homes"* [1:45 ¶ 52 in interview analysis](https://go.atlasti.com/32e35350-0ea2-49d6-8d06-20d1c3d30273/documents/b5c0cb13-2834-41fe-98e2-f35ea219267a/quotations/0332398e-d568-4038-9d79-ad49ecfc704c)

*"She can access any information"* [1:51 ¶ 58 in interview analysis](https://go.atlasti.com/32e35350-0ea2-49d6-8d06-20d1c3d30273/documents/b5c0cb13-2834-41fe-98e2-f35ea219267a/quotations/19ddd88d-4565-4692-acfd-d6af2fc3aa8d)

*"I think mHealth can improve access, appointment on time, availability of information for decision making increased"* [1:53 ¶ 59 in interview analysis](https://go.atlasti.com/32e35350-0ea2-49d6-8d06-20d1c3d30273/documents/b5c0cb13-2834-41fe-98e2-f35ea219267a/quotations/fcb3898d-335d-4d72-b7c0-67356cd5e50a)

*"Her husband or children will also push her to go even if the condition may make the on a different day"* [1:65 ¶ 67 in interview analysis](https://go.atlasti.com/32e35350-0ea2-49d6-8d06-20d1c3d30273/documents/b5c0cb13-2834-41fe-98e2-f35ea219267a/quotations/61bee170-7829-43a6-8528-9d0392ec506d)

*"Every system is getting digitalized so digitalizing health may have much advantage more than just appointme"* [1:68 ¶ 70 in interview analysis](https://go.atlasti.com/32e35350-0ea2-49d6-8d06-20d1c3d30273/documents/b5c0cb13-2834-41fe-98e2-f35ea219267a/quotations/ab6b89bf-1dbb-42cd-9e83-dd229d8ee603)

*"Rural mothers are respectful; they do what they told to do, so fear of poor success is not a problem. Technology is not a new. Mobile is everywhere; messaging common so I don’t there will be a problem. Teaching mothers will be important"* [1:77 ¶ 77 in interview analysis](https://go.atlasti.com/32e35350-0ea2-49d6-8d06-20d1c3d30273/documents/b5c0cb13-2834-41fe-98e2-f35ea219267a/quotations/546d2f0c-e7f8-480f-bd32-9c1dada49ab6)

*"hope mothers will turn to use mobiles, but only after getting its importance that why health education should be crucial part of this implementation"* [1:81 ¶ 80 in interview analysis](https://go.atlasti.com/32e35350-0ea2-49d6-8d06-20d1c3d30273/documents/b5c0cb13-2834-41fe-98e2-f35ea219267a/quotations/5c9d7b77-c5d8-4eed-aae6-53392d5acf1b)

*"It is helpful to also send information which are not appropriate in person"* [2:3 ¶ 11 in KII](https://go.atlasti.com/32e35350-0ea2-49d6-8d06-20d1c3d30273/documents/504671be-e9ef-4f58-8b5d-807935cb16a1/quotations/ac1c138e-e6b9-4363-b8ec-de1fe9538226)

*"Yeah, it will have effective change on community"* [2:7 ¶ 17 in KII](https://go.atlasti.com/32e35350-0ea2-49d6-8d06-20d1c3d30273/documents/504671be-e9ef-4f58-8b5d-807935cb16a1/quotations/73f43d08-5cb1-4192-bccc-5f28703a0969)

*"Yeah, previously mothers use the information they get during ANC, but mothers may forget as time progress so mHealth helpful backup"* [2:9 ¶ 19 in KII](https://go.atlasti.com/32e35350-0ea2-49d6-8d06-20d1c3d30273/documents/504671be-e9ef-4f58-8b5d-807935cb16a1/quotations/222b1b29-65cd-4ad8-8c2e-d48a8a16ace6)

*"Information access, no transportation cost, and no professional resource waste are very good parts of the mHealth"* [2:11 ¶ 21 in KII](https://go.atlasti.com/32e35350-0ea2-49d6-8d06-20d1c3d30273/documents/504671be-e9ef-4f58-8b5d-807935cb16a1/quotations/07608a31-e0cf-432e-908f-13cf0125d496)

*"No need of motor or vehicles because it is mHealth. I will be happy to tell people this service"* [2:36 ¶ 55 in KII](https://go.atlasti.com/32e35350-0ea2-49d6-8d06-20d1c3d30273/documents/504671be-e9ef-4f58-8b5d-807935cb16a1/quotations/91bb2d54-3efc-47c4-bdad-148519d9a2f4)

*"The difference for mHealth is mother always get counseling or health education unlike that of visits when large queue limits they counseling and professionals do finishing work. I think mHealth can be benefited if the current behavior can be improved"* [3:6 ¶ 5 in Group discussion](https://go.atlasti.com/32e35350-0ea2-49d6-8d06-20d1c3d30273/documents/2569dddf-9aa1-42e1-9ece-a8d4dbbb0f25/quotations/cb94919c-29f6-4f8e-b540-3213a1b144ea)

*"It looks like mothers are tired of current service provision so the mHealth may be liked by them"* [3:18 ¶ 12 in Group discussion](https://go.atlasti.com/32e35350-0ea2-49d6-8d06-20d1c3d30273/documents/2569dddf-9aa1-42e1-9ece-a8d4dbbb0f25/quotations/359357fc-fc13-4b4b-8da6-7aa224bea8b4)

*"Previously, people think poor health system and there are many compliant for which reason they are not coming to service area; mHealth may tell the people that the provider and the government are now giving good attention"* [3:23 ¶ 14 in Group discussion](https://go.atlasti.com/32e35350-0ea2-49d6-8d06-20d1c3d30273/documents/2569dddf-9aa1-42e1-9ece-a8d4dbbb0f25/quotations/3122f18c-8b00-4850-8f60-8f3b129159c1)

*"Over time this can change community perception and they may reebok mothers who faced the health problem because of poor follow up and poor usage of messages because they already knew that they are been under intensive attention follow up"* [3:24 ¶ 14 in Group discussion](https://go.atlasti.com/32e35350-0ea2-49d6-8d06-20d1c3d30273/documents/2569dddf-9aa1-42e1-9ece-a8d4dbbb0f25/quotations/6b56db59-1599-4a08-9d38-bf3d6316ef89)

*"There may be emerged women who are successful because of mHealth and they may tell the story of mHealth which further educate the community"* [3:25 ¶ 14 in Group discussion](https://go.atlasti.com/32e35350-0ea2-49d6-8d06-20d1c3d30273/documents/2569dddf-9aa1-42e1-9ece-a8d4dbbb0f25/quotations/c8082489-e7b1-4200-a311-1de0beaa60c8)

*"It can make the child health growth, for mothers to know danger signs, and during PNC to keep herself from unnecessary early pregnancy. It has no problem as long as the message receiver is the mother or someone oriented on the issue"* [3:27 ¶ 16 in Group discussion](https://go.atlasti.com/32e35350-0ea2-49d6-8d06-20d1c3d30273/documents/2569dddf-9aa1-42e1-9ece-a8d4dbbb0f25/quotations/af04de63-7662-4465-8278-b8ecb3463b91)

*"Of course having organized maternal data can also promote contacting atleast risky mothers even without funds."* [3:38 ¶ 22 in Group discussion](https://go.atlasti.com/32e35350-0ea2-49d6-8d06-20d1c3d30273/documents/2569dddf-9aa1-42e1-9ece-a8d4dbbb0f25/quotations/07fb40f3-7688-4d5e-88e4-ab2939c2102e)

*" It is a big opportunity to meet the patient again virtually especially when you missed something in person. Consultation normally should not be a onetime activity rather an effective consultation should be continuous. "* [3:41 ¶ 26 in Group discussion](https://go.atlasti.com/32e35350-0ea2-49d6-8d06-20d1c3d30273/documents/2569dddf-9aa1-42e1-9ece-a8d4dbbb0f25/quotations/92863043-b285-4b95-af3b-e95bc6f3b833)

*"Traditionally, when errors happen or if the patients go with the wrong drug there is no way to correct it back. It means the patient have to suffer the error and then come back if alive, but this time we have organized evidence and further messages"* [3:42 ¶ 26 in Group discussion](https://go.atlasti.com/32e35350-0ea2-49d6-8d06-20d1c3d30273/documents/2569dddf-9aa1-42e1-9ece-a8d4dbbb0f25/quotations/86937602-6b3d-40cc-8424-dbdb66fbc379)

*"Benefit for mothers for next pregnancies and healthy prospect for children"* [3:50 ¶ 33 in Group discussion](https://go.atlasti.com/32e35350-0ea2-49d6-8d06-20d1c3d30273/documents/2569dddf-9aa1-42e1-9ece-a8d4dbbb0f25/quotations/eed5d53c-9115-4dfc-93f8-cbb885fbe00e)

*"Satisfaction of their client service and familiarization with technology"* [3:59 ¶ 45 in Group discussion](https://go.atlasti.com/32e35350-0ea2-49d6-8d06-20d1c3d30273/documents/2569dddf-9aa1-42e1-9ece-a8d4dbbb0f25/quotations/defb5e9e-3b09-4748-b4c1-f179567dafd1)

*"Planning, decision making based on mHealth, and increased service takers"* [3:61 ¶ 47 in Group discussion](https://go.atlasti.com/32e35350-0ea2-49d6-8d06-20d1c3d30273/documents/2569dddf-9aa1-42e1-9ece-a8d4dbbb0f25/quotations/69dfd8d8-4bcd-4fc5-9773-9322cf6453ae)

*"Support related to mHealth, fulfillment of deficiencies of human and other resource limitations, fulfilling the maternal and child health vision, and respect from the community. Acceptance from the community"* [3:62 ¶ 48 in Group discussion](https://go.atlasti.com/32e35350-0ea2-49d6-8d06-20d1c3d30273/documents/2569dddf-9aa1-42e1-9ece-a8d4dbbb0f25/quotations/d760ea9a-4bee-4fa5-817b-a79ea56792a4)

*"he respect for pregnancy mothers may think gave many births and not caring seriously. Need of behavioral change for full considerations"* [3:67 ¶ 53 in Group discussion](https://go.atlasti.com/32e35350-0ea2-49d6-8d06-20d1c3d30273/documents/2569dddf-9aa1-42e1-9ece-a8d4dbbb0f25/quotations/05efc5e3-a479-46ef-9417-6acbea1f758e)

**Benefits of mHealth: Help professional**

*"We always give best service to our best capacity, but mHealth may advance this service"* [1:24 ¶ 34 in interview analysis](https://go.atlasti.com/32e35350-0ea2-49d6-8d06-20d1c3d30273/documents/b5c0cb13-2834-41fe-98e2-f35ea219267a/quotations/e0502ed6-acb3-4785-80d4-5a6dc5491337)

*"As HEWs we cannot reach for every mother on time but text can easily reach to every mother"* [1:34 ¶ 39 in interview analysis](https://go.atlasti.com/32e35350-0ea2-49d6-8d06-20d1c3d30273/documents/b5c0cb13-2834-41fe-98e2-f35ea219267a/quotations/81bb662b-4dec-4679-bf2a-eedcceeaa89e)

*"This mHealth better than our previous our service"* [1:36 ¶ 40 in interview analysis](https://go.atlasti.com/32e35350-0ea2-49d6-8d06-20d1c3d30273/documents/b5c0cb13-2834-41fe-98e2-f35ea219267a/quotations/4b5406ba-8183-4068-b671-3408da1f36ee)

*"further improve the already improving service"* [1:50 ¶ 57 in interview analysis](https://go.atlasti.com/32e35350-0ea2-49d6-8d06-20d1c3d30273/documents/b5c0cb13-2834-41fe-98e2-f35ea219267a/quotations/310566a7-eda3-408a-8b81-b2a5f7198617)

*"Our current ANC and other formats now request mother to register their favorite phone number."* [1:56 ¶ 62 in interview analysis](https://go.atlasti.com/32e35350-0ea2-49d6-8d06-20d1c3d30273/documents/b5c0cb13-2834-41fe-98e2-f35ea219267a/quotations/9cdc64fb-80ba-4fcc-b021-43e2a110e5d4)

*"Every system is getting digitalized so digitalizing health may have much advantage more than just appointme"* [1:68 ¶ 70 in interview analysis](https://go.atlasti.com/32e35350-0ea2-49d6-8d06-20d1c3d30273/documents/b5c0cb13-2834-41fe-98e2-f35ea219267a/quotations/ab6b89bf-1dbb-42cd-9e83-dd229d8ee603)

*"mothers usually take what we tell them, it will be successful. I don’t think reading is a problem because families have all learned people inside, the problem is which phone is accessible or near to mother"* [1:82 ¶ 81 in interview analysis](https://go.atlasti.com/32e35350-0ea2-49d6-8d06-20d1c3d30273/documents/b5c0cb13-2834-41fe-98e2-f35ea219267a/quotations/46d10ae6-f0c5-4c17-8e6e-ef0fde973d0a)

*"It is helpful to also send information which are not appropriate in person"* [2:3 ¶ 11 in KII](https://go.atlasti.com/32e35350-0ea2-49d6-8d06-20d1c3d30273/documents/504671be-e9ef-4f58-8b5d-807935cb16a1/quotations/ac1c138e-e6b9-4363-b8ec-de1fe9538226)

*"Yeah, it will have effective change on community"* [2:7 ¶ 17 in KII](https://go.atlasti.com/32e35350-0ea2-49d6-8d06-20d1c3d30273/documents/504671be-e9ef-4f58-8b5d-807935cb16a1/quotations/73f43d08-5cb1-4192-bccc-5f28703a0969)

*"Yeah, previously mothers use the information they get during ANC, but mothers may forget as time progress so mHealth helpful backup"* [2:9 ¶ 19 in KII](https://go.atlasti.com/32e35350-0ea2-49d6-8d06-20d1c3d30273/documents/504671be-e9ef-4f58-8b5d-807935cb16a1/quotations/222b1b29-65cd-4ad8-8c2e-d48a8a16ace6)

*"Since we have many gaps this should be seen as an opportunity"* [2:35 ¶ 54 in KII](https://go.atlasti.com/32e35350-0ea2-49d6-8d06-20d1c3d30273/documents/504671be-e9ef-4f58-8b5d-807935cb16a1/quotations/38376713-bfe7-40d5-891a-cad1297c7d55)

*"Previous teaching of mother through home to home is going to be through mHealth that very affordable and easily accessible."* [3:2 ¶ 3 in Group discussion](https://go.atlasti.com/32e35350-0ea2-49d6-8d06-20d1c3d30273/documents/2569dddf-9aa1-42e1-9ece-a8d4dbbb0f25/quotations/54cd25c0-0333-45bb-92d4-2a29c76810b8)

*"For educated mothers messages will be option less"* [3:12 ¶ 8 in Group discussion](https://go.atlasti.com/32e35350-0ea2-49d6-8d06-20d1c3d30273/documents/2569dddf-9aa1-42e1-9ece-a8d4dbbb0f25/quotations/4ef9e4ad-819f-487d-bf51-91545de8f535)

*"Starting from me we feed additional food at 2months because the kind of job we engaged, but now I think the government provided right for infant and this could be helpful"* [3:13 ¶ 8 in Group discussion](https://go.atlasti.com/32e35350-0ea2-49d6-8d06-20d1c3d30273/documents/2569dddf-9aa1-42e1-9ece-a8d4dbbb0f25/quotations/cdd45bd8-68f8-4cca-8298-14056efad687)

*"It looks like mothers are tired of current service provision so the mHealth may be liked by them"* [3:18 ¶ 12 in Group discussion](https://go.atlasti.com/32e35350-0ea2-49d6-8d06-20d1c3d30273/documents/2569dddf-9aa1-42e1-9ece-a8d4dbbb0f25/quotations/359357fc-fc13-4b4b-8da6-7aa224bea8b4)

*"There may be emerged women who are successful because of mHealth and they may tell the story of mHealth which further educate the community"* [3:25 ¶ 14 in Group discussion](https://go.atlasti.com/32e35350-0ea2-49d6-8d06-20d1c3d30273/documents/2569dddf-9aa1-42e1-9ece-a8d4dbbb0f25/quotations/c8082489-e7b1-4200-a311-1de0beaa60c8)

*"As a health professionals applying technology can only easy our burden of job. The main thing in this service is mothers having mobile"* [3:29 ¶ 17 in Group discussion](https://go.atlasti.com/32e35350-0ea2-49d6-8d06-20d1c3d30273/documents/2569dddf-9aa1-42e1-9ece-a8d4dbbb0f25/quotations/280e6dd0-324c-49af-9d8a-d9157174e1e2)

*"because of mHealth we may every address of women and now we can also call them when necessary. It will be very helpful specially organized service provision. Everyone respect health professionals so hopefully mother s also well respond to our requests"* [3:34 ¶ 19 in Group discussion](https://go.atlasti.com/32e35350-0ea2-49d6-8d06-20d1c3d30273/documents/2569dddf-9aa1-42e1-9ece-a8d4dbbb0f25/quotations/8244f6fe-ad73-4e80-bf73-c97974d336f7)

*"We will provide the service for mothers we know now, but previously we just provide service for whoever coming and do not those who are not coming"* [3:35 ¶ 20 in Group discussion](https://go.atlasti.com/32e35350-0ea2-49d6-8d06-20d1c3d30273/documents/2569dddf-9aa1-42e1-9ece-a8d4dbbb0f25/quotations/c2a21368-bce1-479f-b9a3-afdded774632)

*"Of course having organized maternal data can also promote contacting atleast risky mothers even without funds."* [3:38 ¶ 22 in Group discussion](https://go.atlasti.com/32e35350-0ea2-49d6-8d06-20d1c3d30273/documents/2569dddf-9aa1-42e1-9ece-a8d4dbbb0f25/quotations/07fb40f3-7688-4d5e-88e4-ab2939c2102e)

*"It is a big opportunity to meet the patient again virtually especially when you missed something in person. Consultation normally should not be a onetime activity rather an effective consultation should be continuous. "* [3:41 ¶ 26 in Group discussion](https://go.atlasti.com/32e35350-0ea2-49d6-8d06-20d1c3d30273/documents/2569dddf-9aa1-42e1-9ece-a8d4dbbb0f25/quotations/92863043-b285-4b95-af3b-e95bc6f3b833)

*"Traditionally, when errors happen or if the patients go with the wrong drug there is no way to correct it back. It means the patient have to suffer the error and then come back if alive, but this time we have organized evidence and further messages"* [3:42 ¶ 26 in Group discussion](https://go.atlasti.com/32e35350-0ea2-49d6-8d06-20d1c3d30273/documents/2569dddf-9aa1-42e1-9ece-a8d4dbbb0f25/quotations/86937602-6b3d-40cc-8424-dbdb66fbc379)

*"At the end this is very important piece of technology that can reduce mortality of mothers and infant"* [3:43 ¶ 28 in Group discussion](https://go.atlasti.com/32e35350-0ea2-49d6-8d06-20d1c3d30273/documents/2569dddf-9aa1-42e1-9ece-a8d4dbbb0f25/quotations/13e81744-fb75-48da-9ca0-eb32d164bd03)

*"Those infrastructure and awareness creation in staffs, mothers, and community will safe everything"* [3:46 ¶ 28 in Group discussion](https://go.atlasti.com/32e35350-0ea2-49d6-8d06-20d1c3d30273/documents/2569dddf-9aa1-42e1-9ece-a8d4dbbb0f25/quotations/22371208-b114-4d83-b18c-44e1df0442a0)

*"People will enjoy talking to others how health institution just concerned for their health, but some which is around previously may be suspected until they get full awareness."* [3:52 ¶ 36 in Group discussion](https://go.atlasti.com/32e35350-0ea2-49d6-8d06-20d1c3d30273/documents/2569dddf-9aa1-42e1-9ece-a8d4dbbb0f25/quotations/b735392b-e559-4c1d-87a7-b9ec4d2b86a0)

*"Improve work environment as per the need of the technology and owning the technology"* [3:57 ¶ 42 in Group discussion](https://go.atlasti.com/32e35350-0ea2-49d6-8d06-20d1c3d30273/documents/2569dddf-9aa1-42e1-9ece-a8d4dbbb0f25/quotations/7bc3082d-2bce-4e49-ad6f-c3a9fd18ab03)

*"Aspiring more technologies and helping mothers, taking trainings, giving trainings after getting themselves initially, helping new employee, continuing with the technology application"* [3:58 ¶ 43 in Group discussion](https://go.atlasti.com/32e35350-0ea2-49d6-8d06-20d1c3d30273/documents/2569dddf-9aa1-42e1-9ece-a8d4dbbb0f25/quotations/e0c9d15a-b243-466c-8a7c-31d482a01f59)

*"Experience with technology. Enable them to identify areas where mHealth can be applied. Achieving their objectives"* [3:60 ¶ 46 in Group discussion](https://go.atlasti.com/32e35350-0ea2-49d6-8d06-20d1c3d30273/documents/2569dddf-9aa1-42e1-9ece-a8d4dbbb0f25/quotations/7b5b0a2b-6d17-4f40-af02-ca027bfbdb95)

*"Planning, decision making based on mHealth, and increased service takers"* [3:61 ¶ 47 in Group discussion](https://go.atlasti.com/32e35350-0ea2-49d6-8d06-20d1c3d30273/documents/2569dddf-9aa1-42e1-9ece-a8d4dbbb0f25/quotations/69dfd8d8-4bcd-4fc5-9773-9322cf6453ae)

*"Support related to mHealth, fulfillment of deficiencies of human and other resource limitations, fulfilling the maternal and child health vision, and respect from the community. Acceptance from the community"* [3:62 ¶ 48 in Group discussion](https://go.atlasti.com/32e35350-0ea2-49d6-8d06-20d1c3d30273/documents/2569dddf-9aa1-42e1-9ece-a8d4dbbb0f25/quotations/d760ea9a-4bee-4fa5-817b-a79ea56792a4)

**Benefits of mHealth: Improve decision**

*"it will improve mothers’ time wastage for information that they can get through mHealth"* [1:5 ¶ 20 in interview analysis](https://go.atlasti.com/32e35350-0ea2-49d6-8d06-20d1c3d30273/documents/b5c0cb13-2834-41fe-98e2-f35ea219267a/quotations/8d0529de-bf85-49d8-be79-b233bf30d7f9)

*"will be helpful because it will remain in the mother’s hand for a long time and she can read at a time"* [1:23 ¶ 33 in interview analysis](https://go.atlasti.com/32e35350-0ea2-49d6-8d06-20d1c3d30273/documents/b5c0cb13-2834-41fe-98e2-f35ea219267a/quotations/2cca46d5-fa1e-410f-9559-fd449875c844)

*" it can improve their decision making. This service could increase women self-decision especially, on ANC, PNC, feeding practice, evidence can helps her"* [1:40 ¶ 45 in interview analysis](https://go.atlasti.com/32e35350-0ea2-49d6-8d06-20d1c3d30273/documents/b5c0cb13-2834-41fe-98e2-f35ea219267a/quotations/f5918bda-cc9d-4c7c-9a15-82a58661e0af)

*"provide information to make decisions, motivate her to convince her husband."* [1:42 ¶ 49 in interview analysis](https://go.atlasti.com/32e35350-0ea2-49d6-8d06-20d1c3d30273/documents/b5c0cb13-2834-41fe-98e2-f35ea219267a/quotations/e2058b2f-f698-4b6d-86ad-c4109348b618)

*"Having information on time can improve the health of mothers and children"* [1:49 ¶ 56 in interview analysis](https://go.atlasti.com/32e35350-0ea2-49d6-8d06-20d1c3d30273/documents/b5c0cb13-2834-41fe-98e2-f35ea219267a/quotations/3805e358-70a7-4c27-81e8-191c68fe4894)

*"mHealth can provide evidence to make decision and can improve everything mothers need to take the service"* [1:55 ¶ 60 in interview analysis](https://go.atlasti.com/32e35350-0ea2-49d6-8d06-20d1c3d30273/documents/b5c0cb13-2834-41fe-98e2-f35ea219267a/quotations/7abcbadc-853f-45ea-b4b6-cee4efc81936)

*"However, having this information in her hand will continuously strike her to go to the appointment"* [1:64 ¶ 67 in interview analysis](https://go.atlasti.com/32e35350-0ea2-49d6-8d06-20d1c3d30273/documents/b5c0cb13-2834-41fe-98e2-f35ea219267a/quotations/7e44d1bc-5bc6-45ab-a5f7-6df7ac11b9fb)

*"Service can be improved through mHealth because atleast having information is better than nothing"* [1:70 ¶ 71 in interview analysis](https://go.atlasti.com/32e35350-0ea2-49d6-8d06-20d1c3d30273/documents/b5c0cb13-2834-41fe-98e2-f35ea219267a/quotations/5ac5982b-be29-41ec-aab9-03851b6c3abc)

*"Mothers should understand initially the important, but on our side I don’t think there will be any problem"* [2:15 ¶ 27 in KII](https://go.atlasti.com/32e35350-0ea2-49d6-8d06-20d1c3d30273/documents/504671be-e9ef-4f58-8b5d-807935cb16a1/quotations/3a8207c5-3fc5-492c-bfe3-a8d022f7eee8)

*"The difference for mHealth is mother always get counseling or health education unlike that of visits when large queue limits they counseling and professionals do finishing work. I think mHealth can be benefited if the current behavior can be improved"* [3:6 ¶ 5 in Group discussion](https://go.atlasti.com/32e35350-0ea2-49d6-8d06-20d1c3d30273/documents/2569dddf-9aa1-42e1-9ece-a8d4dbbb0f25/quotations/cb94919c-29f6-4f8e-b540-3213a1b144ea)

*"Mother are already exposed to health education, but this one is directly motivates mothers at their home. I think it will improve maternal and child health if we use it appropriately"* [3:10 ¶ 8 in Group discussion](https://go.atlasti.com/32e35350-0ea2-49d6-8d06-20d1c3d30273/documents/2569dddf-9aa1-42e1-9ece-a8d4dbbb0f25/quotations/92d2b149-e4ed-4bec-9618-744292b38739)

*"They may even consider the increased risk of danger related to pregnancy, child feeding, and vaccination. May be the emergence of new diseases can enforce them to vaccinate their children"* [3:20 ¶ 12 in Group discussion](https://go.atlasti.com/32e35350-0ea2-49d6-8d06-20d1c3d30273/documents/2569dddf-9aa1-42e1-9ece-a8d4dbbb0f25/quotations/45091896-0483-4b1b-8e1b-90b0ae2d58e4)

*"Over time this can change community perception and they may reebok mothers who faced the health problem because of poor follow up and poor usage of messages because they already knew that they are been under intensive attention follow up"* [3:24 ¶ 14 in Group discussion](https://go.atlasti.com/32e35350-0ea2-49d6-8d06-20d1c3d30273/documents/2569dddf-9aa1-42e1-9ece-a8d4dbbb0f25/quotations/6b56db59-1599-4a08-9d38-bf3d6316ef89)

*"It can also introduce labor division because we may know who must serve whom or how many. Previously we just communicate numbers and percent, and tell people reduced or increased but we do not know why and when the number becomes that."* [3:37 ¶ 20 in Group discussion](https://go.atlasti.com/32e35350-0ea2-49d6-8d06-20d1c3d30273/documents/2569dddf-9aa1-42e1-9ece-a8d4dbbb0f25/quotations/4edb88e3-e87a-4903-898a-0881f6167a7c)

*"Service at their home and Information and on time vaccination and Healthy child live"* [3:48 ¶ 31 in Group discussion](https://go.atlasti.com/32e35350-0ea2-49d6-8d06-20d1c3d30273/documents/2569dddf-9aa1-42e1-9ece-a8d4dbbb0f25/quotations/4af73540-5ed7-4c4c-826a-3525e70043ff)

*"People will enjoy talking to others how health institution just concerned for their health, but some which is around previously may be suspected until they get full awareness."* [3:52 ¶ 36 in Group discussion](https://go.atlasti.com/32e35350-0ea2-49d6-8d06-20d1c3d30273/documents/2569dddf-9aa1-42e1-9ece-a8d4dbbb0f25/quotations/b735392b-e559-4c1d-87a7-b9ec4d2b86a0)

*"he respect for pregnancy mothers may think gave many births and not caring seriously. Need of behavioral change for full considerations"* [3:67 ¶ 53 in Group discussion](https://go.atlasti.com/32e35350-0ea2-49d6-8d06-20d1c3d30273/documents/2569dddf-9aa1-42e1-9ece-a8d4dbbb0f25/quotations/05efc5e3-a479-46ef-9417-6acbea1f758e)

**Benefits of mHealth: Improve MCH**

*"Non-use of the service because of negligence and forgetting can be improved by frequent messages"* [1:6 ¶ 22 in interview analysis](https://go.atlasti.com/32e35350-0ea2-49d6-8d06-20d1c3d30273/documents/b5c0cb13-2834-41fe-98e2-f35ea219267a/quotations/4d323275-4454-4ee5-976f-c72807b45fb1)

*"It can increase utilization better than that we have previously"* [1:26 ¶ 35 in interview analysis](https://go.atlasti.com/32e35350-0ea2-49d6-8d06-20d1c3d30273/documents/b5c0cb13-2834-41fe-98e2-f35ea219267a/quotations/386fa2cc-2754-4ffd-9ada-bdb003a6c941)

*"Even since this digital it can further improve the service and increase interest"* [1:28 ¶ 36 in interview analysis](https://go.atlasti.com/32e35350-0ea2-49d6-8d06-20d1c3d30273/documents/b5c0cb13-2834-41fe-98e2-f35ea219267a/quotations/70f81484-0344-440a-9e25-4b881e2bb6cd)

*"This mHealth better than our previous our service"* [1:36 ¶ 40 in interview analysis](https://go.atlasti.com/32e35350-0ea2-49d6-8d06-20d1c3d30273/documents/b5c0cb13-2834-41fe-98e2-f35ea219267a/quotations/4b5406ba-8183-4068-b671-3408da1f36ee)

*"For maternal and child health the decision to take service is mothers duty"* [1:43 ¶ 50 in interview analysis](https://go.atlasti.com/32e35350-0ea2-49d6-8d06-20d1c3d30273/documents/b5c0cb13-2834-41fe-98e2-f35ea219267a/quotations/f868fb8f-27ed-4ff3-9c84-96b68d9437fe)

*"Having information on time can improve the health of mothers and children"* [1:49 ¶ 56 in interview analysis](https://go.atlasti.com/32e35350-0ea2-49d6-8d06-20d1c3d30273/documents/b5c0cb13-2834-41fe-98e2-f35ea219267a/quotations/3805e358-70a7-4c27-81e8-191c68fe4894)

*"further improve the already improving service"* [1:50 ¶ 57 in interview analysis](https://go.atlasti.com/32e35350-0ea2-49d6-8d06-20d1c3d30273/documents/b5c0cb13-2834-41fe-98e2-f35ea219267a/quotations/310566a7-eda3-408a-8b81-b2a5f7198617)

*"if it continue, it can be effective and be important than previous way of service provision"* [1:57 ¶ 63 in interview analysis](https://go.atlasti.com/32e35350-0ea2-49d6-8d06-20d1c3d30273/documents/b5c0cb13-2834-41fe-98e2-f35ea219267a/quotations/d3e33a10-5744-4ade-b41f-98e1d9cab490)

*"I think whatever we put in to the community to improve health service are always bring improvement beside the some resistances that can be happen"* [1:59 ¶ 64 in interview analysis](https://go.atlasti.com/32e35350-0ea2-49d6-8d06-20d1c3d30273/documents/b5c0cb13-2834-41fe-98e2-f35ea219267a/quotations/902a056d-dd13-4e22-9e69-071e15a57823)

*"This can improve and put our usual service one step forward"* [1:62 ¶ 66 in interview analysis](https://go.atlasti.com/32e35350-0ea2-49d6-8d06-20d1c3d30273/documents/b5c0cb13-2834-41fe-98e2-f35ea219267a/quotations/e8d2f73d-6610-420d-ae4f-2f003d2b3ca2)

*"I don’t think people can be affected negatively because they love to learn new things, especially, that improve service, they appreciate how government improve health"* [1:74 ¶ 75 in interview analysis](https://go.atlasti.com/32e35350-0ea2-49d6-8d06-20d1c3d30273/documents/b5c0cb13-2834-41fe-98e2-f35ea219267a/quotations/13b1d67e-fb44-4b7d-8e38-d7f5255f1981)

*"Rural mothers are respectful; they do what they told to do, so fear of poor success is not a problem. Technology is not a new. Mobile is everywhere; messaging common so I don’t there will be a problem. Teaching mothers will be important"* [1:77 ¶ 77 in interview analysis](https://go.atlasti.com/32e35350-0ea2-49d6-8d06-20d1c3d30273/documents/b5c0cb13-2834-41fe-98e2-f35ea219267a/quotations/546d2f0c-e7f8-480f-bd32-9c1dada49ab6)

*"One thing I assure is it will increase up taking maternal and child health service"* [1:78 ¶ 78 in interview analysis](https://go.atlasti.com/32e35350-0ea2-49d6-8d06-20d1c3d30273/documents/b5c0cb13-2834-41fe-98e2-f35ea219267a/quotations/6de8e6ff-138a-47e9-93ea-9d8d838666fd)

*"It can remind which is especially important for family planning. Without human travel to home, to tell teach mothers and no transportation cost. For mother who needs C/S and SC children, it will be helpful"* [1:83 ¶ 83 in interview analysis](https://go.atlasti.com/32e35350-0ea2-49d6-8d06-20d1c3d30273/documents/b5c0cb13-2834-41fe-98e2-f35ea219267a/quotations/36d25fef-0941-4101-8593-6f97f0d6a2f0)

*"If health system organized this way it will be helpful"* [2:4 ¶ 12 in KII](https://go.atlasti.com/32e35350-0ea2-49d6-8d06-20d1c3d30273/documents/504671be-e9ef-4f58-8b5d-807935cb16a1/quotations/a652448e-0a9e-4a06-b1af-057ba540e38c)

*"this is the main thing to improve maternal and child care especially using message education"* [2:6 ¶ 16 in KII](https://go.atlasti.com/32e35350-0ea2-49d6-8d06-20d1c3d30273/documents/504671be-e9ef-4f58-8b5d-807935cb16a1/quotations/d821c466-e7bc-4de0-8af6-e61312e8b058)

*"After counseling and aware mother it can be improved"* [3:8 ¶ 6 in Group discussion](https://go.atlasti.com/32e35350-0ea2-49d6-8d06-20d1c3d30273/documents/2569dddf-9aa1-42e1-9ece-a8d4dbbb0f25/quotations/d9cd8dda-a335-4978-bdd9-7c9b59687f4e)

*"It can improve those listed service (ANC, PNC, Breastfeeding and vaccine)."* [3:9 ¶ 8 in Group discussion](https://go.atlasti.com/32e35350-0ea2-49d6-8d06-20d1c3d30273/documents/2569dddf-9aa1-42e1-9ece-a8d4dbbb0f25/quotations/4624659f-1333-410a-a54d-e1ed0305f2de)

*"Mother are already exposed to health education, but this one is directly motivates mothers at their home. I think it will improve maternal and child health if we use it appropriately"* [3:10 ¶ 8 in Group discussion](https://go.atlasti.com/32e35350-0ea2-49d6-8d06-20d1c3d30273/documents/2569dddf-9aa1-42e1-9ece-a8d4dbbb0f25/quotations/92d2b149-e4ed-4bec-9618-744292b38739)

*"It looks like mothers are tired of current service provision so the mHealth may be liked by them"* [3:18 ¶ 12 in Group discussion](https://go.atlasti.com/32e35350-0ea2-49d6-8d06-20d1c3d30273/documents/2569dddf-9aa1-42e1-9ece-a8d4dbbb0f25/quotations/359357fc-fc13-4b4b-8da6-7aa224bea8b4)

*"So in that case it can completely change the stream and interest. Or they may consider the attention they are getting."* [3:19 ¶ 12 in Group discussion](https://go.atlasti.com/32e35350-0ea2-49d6-8d06-20d1c3d30273/documents/2569dddf-9aa1-42e1-9ece-a8d4dbbb0f25/quotations/e0e7fc0b-4be1-4f25-9b77-a5b55774be68)

*"They may even consider the increased risk of danger related to pregnancy, child feeding, and vaccination. May be the emergence of new diseases can enforce them to vaccinate their children"* [3:20 ¶ 12 in Group discussion](https://go.atlasti.com/32e35350-0ea2-49d6-8d06-20d1c3d30273/documents/2569dddf-9aa1-42e1-9ece-a8d4dbbb0f25/quotations/45091896-0483-4b1b-8e1b-90b0ae2d58e4)

*"At the end this is very important piece of technology that can reduce mortality of mothers and infant"* [3:43 ¶ 28 in Group discussion](https://go.atlasti.com/32e35350-0ea2-49d6-8d06-20d1c3d30273/documents/2569dddf-9aa1-42e1-9ece-a8d4dbbb0f25/quotations/13e81744-fb75-48da-9ca0-eb32d164bd03)

*"Service at their home and Information and on time vaccination and Healthy child live"* [3:48 ¶ 31 in Group discussion](https://go.atlasti.com/32e35350-0ea2-49d6-8d06-20d1c3d30273/documents/2569dddf-9aa1-42e1-9ece-a8d4dbbb0f25/quotations/4af73540-5ed7-4c4c-826a-3525e70043ff)

*"Healthy child feeding and behavioral change for mother that wll be long lasting"* [3:49 ¶ 32 in Group discussion](https://go.atlasti.com/32e35350-0ea2-49d6-8d06-20d1c3d30273/documents/2569dddf-9aa1-42e1-9ece-a8d4dbbb0f25/quotations/fb626012-2340-4a0a-ae8d-e0005cae45bd)

**CHALLENGES OF MHEALTH IMPLEMENTATION THEMES**

**Challenges of mHealth implementation: Acceptance**

*"I think it will improve but if someone get information from her phone, she should be someone who actively using mobile"* [1:18 ¶ 30 in interview analysis](https://go.atlasti.com/32e35350-0ea2-49d6-8d06-20d1c3d30273/documents/b5c0cb13-2834-41fe-98e2-f35ea219267a/quotations/77d26772-cffe-4459-81c2-8b5ef37d4cb7)

*"Sometimes people may think mobile make women rude"* [1:35 ¶ 39 in interview analysis](https://go.atlasti.com/32e35350-0ea2-49d6-8d06-20d1c3d30273/documents/b5c0cb13-2834-41fe-98e2-f35ea219267a/quotations/3ba990a2-efa9-4bda-859a-d716c1d40fe9)

*"But, when women take messages husband may think she is meeting someone. This service may increase workload on professionals"* [1:37 ¶ 40 in interview analysis](https://go.atlasti.com/32e35350-0ea2-49d6-8d06-20d1c3d30273/documents/b5c0cb13-2834-41fe-98e2-f35ea219267a/quotations/2791684b-9b88-4ddc-8ad2-5bb746c66d00)

*"sometime our community may not say pregnancy until the conception reach third months, so application of mHealth may declare it early so which may make some inconvenience"* [1:84 ¶ 84 in interview analysis](https://go.atlasti.com/32e35350-0ea2-49d6-8d06-20d1c3d30273/documents/b5c0cb13-2834-41fe-98e2-f35ea219267a/quotations/b1c9bb91-6c53-490c-b820-6efede24841e)

*"Reading is one of the difficulties. May be absence of electricity, inability to read, lack of mobile phone, lack of interest, lack of support, uninviting political environment"* [1:102 ¶ 101 in interview analysis](https://go.atlasti.com/32e35350-0ea2-49d6-8d06-20d1c3d30273/documents/b5c0cb13-2834-41fe-98e2-f35ea219267a/quotations/cc8680e7-0727-414e-901f-6bdf9bb3adc6)

*"All of them are solvable but may need time to solve"* [1:106 ¶ 106 in interview analysis](https://go.atlasti.com/32e35350-0ea2-49d6-8d06-20d1c3d30273/documents/b5c0cb13-2834-41fe-98e2-f35ea219267a/quotations/5495c258-d7c2-4993-914c-a1ff8991ed6c)

*"The practice will make better because our community need more such service"* [2:8 ¶ 18 in KII](https://go.atlasti.com/32e35350-0ea2-49d6-8d06-20d1c3d30273/documents/504671be-e9ef-4f58-8b5d-807935cb16a1/quotations/2a0a6ec1-1e96-4579-a6cf-075086842e18)

*"Some projects phase out without making any impact"* [2:25 ¶ 40 in KII](https://go.atlasti.com/32e35350-0ea2-49d6-8d06-20d1c3d30273/documents/504671be-e9ef-4f58-8b5d-807935cb16a1/quotations/f21972b2-3399-4cc3-b6d0-d9b2fe6e68c3)

*"Rejection by government/politics. Everyone is not modernized, but for using mothers it will be best"* [2:26 ¶ 41 in KII](https://go.atlasti.com/32e35350-0ea2-49d6-8d06-20d1c3d30273/documents/504671be-e9ef-4f58-8b5d-807935cb16a1/quotations/9638dc46-285c-4a6b-891d-09c506d7361d)

*"Competition between groups"* [2:27 ¶ 42 in KII](https://go.atlasti.com/32e35350-0ea2-49d6-8d06-20d1c3d30273/documents/504671be-e9ef-4f58-8b5d-807935cb16a1/quotations/49c984f3-8fb7-440e-bbc1-65c870190548)

*"Message understanding of the mothers and questions related to why mHealth"* [2:28 ¶ 43 in KII](https://go.atlasti.com/32e35350-0ea2-49d6-8d06-20d1c3d30273/documents/504671be-e9ef-4f58-8b5d-807935cb16a1/quotations/f532d0b7-e8b9-43dc-a33a-dd282d7bae1c)

*"In my view, husbands my think different if some for example calling every time"* [2:33 ¶ 51 in KII](https://go.atlasti.com/32e35350-0ea2-49d6-8d06-20d1c3d30273/documents/504671be-e9ef-4f58-8b5d-807935cb16a1/quotations/9ea48cbd-c90d-435c-b48d-42cf55feb11d)

*"Preparing place for installation, computers and mobiles are necessary, and staff awareness"* [2:43 ¶ 61 in KII](https://go.atlasti.com/32e35350-0ea2-49d6-8d06-20d1c3d30273/documents/504671be-e9ef-4f58-8b5d-807935cb16a1/quotations/2676f43c-c736-45a3-b26c-9f7eb45fe204)

*"Sometimes people may leave support and the program may fail but if strictly followed it will be important"* [3:11 ¶ 8 in Group discussion](https://go.atlasti.com/32e35350-0ea2-49d6-8d06-20d1c3d30273/documents/2569dddf-9aa1-42e1-9ece-a8d4dbbb0f25/quotations/e510220d-2704-49fc-80d0-442d76000b9a)

*"It is a reborn for health system, we hope more technology will come to resurrect or health care."* [3:44 ¶ 28 in Group discussion](https://go.atlasti.com/32e35350-0ea2-49d6-8d06-20d1c3d30273/documents/2569dddf-9aa1-42e1-9ece-a8d4dbbb0f25/quotations/8e18297d-c363-4c6a-bbce-8d9c598bc656)

*"No unique culture, it is all similar. In our culture I think people like learning new things. Even it is not helpful since provided by health professional, mothers always respect. I can’t see any culture contradict the technology"* [3:51 ¶ 35 in Group discussion](https://go.atlasti.com/32e35350-0ea2-49d6-8d06-20d1c3d30273/documents/2569dddf-9aa1-42e1-9ece-a8d4dbbb0f25/quotations/f44f981a-754d-4f78-8767-5ba03385c070)

*": No cultural influence because our respect whatever health professionals order"* [3:54 ¶ 38 in Group discussion](https://go.atlasti.com/32e35350-0ea2-49d6-8d06-20d1c3d30273/documents/2569dddf-9aa1-42e1-9ece-a8d4dbbb0f25/quotations/450efbb5-ad8c-4ab2-85fa-d55c09a44933)

**Challenges of mHealth implementation: Awareness**

*"here when a child is born it feels like good responsibility to give diet immediately"* [1:8 ¶ 23 in interview analysis](https://go.atlasti.com/32e35350-0ea2-49d6-8d06-20d1c3d30273/documents/b5c0cb13-2834-41fe-98e2-f35ea219267a/quotations/62778de0-4452-454a-b3cd-e93ad7158a56)

*"People are proud of this early feeding/food/"* [1:9 ¶ 23 in interview analysis](https://go.atlasti.com/32e35350-0ea2-49d6-8d06-20d1c3d30273/documents/b5c0cb13-2834-41fe-98e2-f35ea219267a/quotations/c017894a-ba8a-49b2-a05f-a15625645857)

*"We know usually even hospitals allow zero level milk and that may be confusing mother to give milk at eerily time"* [1:15 ¶ 28 in interview analysis](https://go.atlasti.com/32e35350-0ea2-49d6-8d06-20d1c3d30273/documents/b5c0cb13-2834-41fe-98e2-f35ea219267a/quotations/6ad6b6e5-91c1-496d-b714-1aad993d6d1c)

*"messages should consider educational level and understanding of mothers"* [1:66 ¶ 68 in interview analysis](https://go.atlasti.com/32e35350-0ea2-49d6-8d06-20d1c3d30273/documents/b5c0cb13-2834-41fe-98e2-f35ea219267a/quotations/8f12b493-24fb-4442-8c8a-a14b04960897)

*"If the mother is not someone who read the message directly, the children or husband might need awareness too"* [1:91 ¶ 90 in interview analysis](https://go.atlasti.com/32e35350-0ea2-49d6-8d06-20d1c3d30273/documents/b5c0cb13-2834-41fe-98e2-f35ea219267a/quotations/1d6b32d3-32e1-49c4-b2c1-9846a9396ab9)

*"When the mother takes these service without the knowledge of her husband that can be a problem when he find out. So the husband should be aware too. May be only when mother search for someone to read her message outside the HH"* [1:92 ¶ 91 in interview analysis](https://go.atlasti.com/32e35350-0ea2-49d6-8d06-20d1c3d30273/documents/b5c0cb13-2834-41fe-98e2-f35ea219267a/quotations/e2ab64eb-b54a-4d21-ad24-96e9f59c04c7)

*"There is always an obstacle but can be reduced by awareness creation. Readability, education, mobile access, network and refusal may be problems"* [1:99 ¶ 99 in interview analysis](https://go.atlasti.com/32e35350-0ea2-49d6-8d06-20d1c3d30273/documents/b5c0cb13-2834-41fe-98e2-f35ea219267a/quotations/cbf8b85f-4b25-4b11-8bff-0e7a0866cc20)

*"Since most people mothers use mobile I think this is the better way of getting best out of it"* [2:10 ¶ 20 in KII](https://go.atlasti.com/32e35350-0ea2-49d6-8d06-20d1c3d30273/documents/504671be-e9ef-4f58-8b5d-807935cb16a1/quotations/d617651e-821e-4a06-9efc-4f0752e4f5c4)

*"If no mobile in mothers’ hand. Uneducated mothers"* [2:24 ¶ 39 in KII](https://go.atlasti.com/32e35350-0ea2-49d6-8d06-20d1c3d30273/documents/504671be-e9ef-4f58-8b5d-807935cb16a1/quotations/7b7782b6-eb69-4c9a-9d52-bd1951d993c3)

*"Whatever it is, we counsel them all the time and they knew that they will face medically dangerous problem if ANC, PNC, and feeding of children was not followed accordingly. However, they do not follow and take more service even they consider as no difference"* [3:5 ¶ 4 in Group discussion](https://go.atlasti.com/32e35350-0ea2-49d6-8d06-20d1c3d30273/documents/2569dddf-9aa1-42e1-9ece-a8d4dbbb0f25/quotations/889722e7-3b19-4a2d-a436-274e2399bf3f)

*"Maternal education, phone, maternal work behavior, and lack of awareness of mHealth benefits"* [3:63 ¶ 50 in Group discussion](https://go.atlasti.com/32e35350-0ea2-49d6-8d06-20d1c3d30273/documents/2569dddf-9aa1-42e1-9ece-a8d4dbbb0f25/quotations/ac1acdc8-52d3-4b9a-8a4d-d00d31d5562b)

**Challenges of mHealth implementation: Devices handling**

*"But no all mothers have mobiles. Some time they may loss their mobile while on appointment and there is still the chance of losing mother even with mHealth"* [1:54 ¶ 59 in interview analysis](https://go.atlasti.com/32e35350-0ea2-49d6-8d06-20d1c3d30273/documents/b5c0cb13-2834-41fe-98e2-f35ea219267a/quotations/c234b5f4-2fe9-4162-9f2c-6d0e3f638c43)

*"This can improve the service but not all women have phone, many mothers"* [1:58 ¶ 63 in interview analysis](https://go.atlasti.com/32e35350-0ea2-49d6-8d06-20d1c3d30273/documents/b5c0cb13-2834-41fe-98e2-f35ea219267a/quotations/cbe82fc9-b65b-48e1-8ba8-887cce3fe4dd)

*"I can say it can be effective, but not all mother have mobiles, mobile is now expensive"* [1:63 ¶ 66 in interview analysis](https://go.atlasti.com/32e35350-0ea2-49d6-8d06-20d1c3d30273/documents/b5c0cb13-2834-41fe-98e2-f35ea219267a/quotations/c075cc72-92ea-4a18-a527-a89e1193abba)

*"Economy, maternal capacity of reading and understanding. Our mothers are living in rural and further farm areas where they may not have charged mobile."* [1:93 ¶ 93 in interview analysis](https://go.atlasti.com/32e35350-0ea2-49d6-8d06-20d1c3d30273/documents/b5c0cb13-2834-41fe-98e2-f35ea219267a/quotations/913afe08-7f4d-4840-98b2-56af9a79f94b)

*"Uneducated, inability to read, electricity, Mothers may not want to use mobile phone"* [1:94 ¶ 94 in interview analysis](https://go.atlasti.com/32e35350-0ea2-49d6-8d06-20d1c3d30273/documents/b5c0cb13-2834-41fe-98e2-f35ea219267a/quotations/dfb5ca2c-6ff5-49f6-9f99-e556148c9f16)

*"May not have phone because of economy i.e. every mother may not phone and less familiarity with the devices"* [1:96 ¶ 96 in interview analysis](https://go.atlasti.com/32e35350-0ea2-49d6-8d06-20d1c3d30273/documents/b5c0cb13-2834-41fe-98e2-f35ea219267a/quotations/6234cf8d-19aa-47a2-b3d1-7ee00f90d60a)

*"Mother lost existing phone within the service period, broken Phone which cannot allow mother read or access, and current market to get new mobile phone"* [1:97 ¶ 97 in interview analysis](https://go.atlasti.com/32e35350-0ea2-49d6-8d06-20d1c3d30273/documents/b5c0cb13-2834-41fe-98e2-f35ea219267a/quotations/6e5915b3-0521-427d-94ff-6f6f5c5477a2)

*"All of them are solvable but may need time to solve"* [1:106 ¶ 106 in interview analysis](https://go.atlasti.com/32e35350-0ea2-49d6-8d06-20d1c3d30273/documents/b5c0cb13-2834-41fe-98e2-f35ea219267a/quotations/5495c258-d7c2-4993-914c-a1ff8991ed6c)

*"Infants are not owners of the service but with family only and if family have not capacity to use due to different reason, for instance, everyone may not use mobile"* [2:29 ¶ 44 in KII](https://go.atlasti.com/32e35350-0ea2-49d6-8d06-20d1c3d30273/documents/504671be-e9ef-4f58-8b5d-807935cb16a1/quotations/1bc96506-56a9-42af-a36b-a7ca6a02262f)

*"This service is possible and when mother do have mobile unless another option is available"* [3:28 ¶ 17 in Group discussion](https://go.atlasti.com/32e35350-0ea2-49d6-8d06-20d1c3d30273/documents/2569dddf-9aa1-42e1-9ece-a8d4dbbb0f25/quotations/8698915f-ee23-49fc-87fd-f1aa999a9803)

*"Mobile is now part of the basic needs of our community. Mobile services are the most used thing in the community, so what possible culture could avoid using mHealth? Exactly there is no culture"* [3:53 ¶ 37 in Group discussion](https://go.atlasti.com/32e35350-0ea2-49d6-8d06-20d1c3d30273/documents/2569dddf-9aa1-42e1-9ece-a8d4dbbb0f25/quotations/10a26e99-6d5c-4502-bb21-5939287fb9c7)

*"Sustainability of the program is the concern, who will always look after computer"* [3:68 ¶ 55 in Group discussion](https://go.atlasti.com/32e35350-0ea2-49d6-8d06-20d1c3d30273/documents/2569dddf-9aa1-42e1-9ece-a8d4dbbb0f25/quotations/408e440a-ada6-44e8-a38e-a6ca9af41f35)

**Challenges of mHealth implementation: negligence**

*"but some are negligent, and mothers knew the consequence of not complying"* [1:3 ¶ 19 in interview analysis](https://go.atlasti.com/32e35350-0ea2-49d6-8d06-20d1c3d30273/documents/b5c0cb13-2834-41fe-98e2-f35ea219267a/quotations/b2185780-cf77-4d75-b704-48bf0a3a1525)

*"Normally, the mother come to health institution at 45 days but some still negligent or forget the date or not aware of the date and do not come that is where mHealth can change"* [1:7 ¶ 22 in interview analysis](https://go.atlasti.com/32e35350-0ea2-49d6-8d06-20d1c3d30273/documents/b5c0cb13-2834-41fe-98e2-f35ea219267a/quotations/f671c7a6-4e69-419d-887c-e2aaa45987e0)

*"I don’t think there will be a problem unless the carelessness of professionals affected those mothers acceptance; they might say is there anything like that?"* [1:72 ¶ 74 in interview analysis](https://go.atlasti.com/32e35350-0ea2-49d6-8d06-20d1c3d30273/documents/b5c0cb13-2834-41fe-98e2-f35ea219267a/quotations/6042ae70-3c9c-4f76-8dd8-3bcccc75380d)

*"Because of this service is new some acceptance can be happen until everyone understand that awareness is necessary because every new service need some effort to aware the users and the potential users, meaning the whole community should get awareness on this issue"* [1:95 ¶ 95 in interview analysis](https://go.atlasti.com/32e35350-0ea2-49d6-8d06-20d1c3d30273/documents/b5c0cb13-2834-41fe-98e2-f35ea219267a/quotations/b8f8aa8e-07e7-44c8-b07b-970c17ca5529)

*"I think it will be more helpful for rural mothers, for towns every mother feed her child early additional food and that look like unchangeable"* [3:15 ¶ 10 in Group discussion](https://go.atlasti.com/32e35350-0ea2-49d6-8d06-20d1c3d30273/documents/2569dddf-9aa1-42e1-9ece-a8d4dbbb0f25/quotations/ace2743b-2c8c-455f-b2d1-dbb8569f3081)

*"Reluctance, being bored of many messages over time, .pre occupation with important thing, hand-to-mouth life style, and daily laborer work"* [3:65 ¶ 52 in Group discussion](https://go.atlasti.com/32e35350-0ea2-49d6-8d06-20d1c3d30273/documents/2569dddf-9aa1-42e1-9ece-a8d4dbbb0f25/quotations/bdb2787e-3c5b-401f-a296-e0264192c9fb)

*"Previous ignorance nature for services"* [3:66 ¶ 53 in Group discussion](https://go.atlasti.com/32e35350-0ea2-49d6-8d06-20d1c3d30273/documents/2569dddf-9aa1-42e1-9ece-a8d4dbbb0f25/quotations/a0da7785-1e88-4cad-9944-68cd86c3d889)

**Challenges of mHealth implementation: Readiness**

*"There could be a little challenge for this because the service is new"* [1:79 ¶ 78 in interview analysis](https://go.atlasti.com/32e35350-0ea2-49d6-8d06-20d1c3d30273/documents/b5c0cb13-2834-41fe-98e2-f35ea219267a/quotations/2e5b480c-2e48-45b1-8c2a-9dfa9d6c053f)

*"But we have to stress continuity and ownership, otherwise when there is an interruption mother may lose hope and distribute the information"* [1:85 ¶ 85 in interview analysis](https://go.atlasti.com/32e35350-0ea2-49d6-8d06-20d1c3d30273/documents/b5c0cb13-2834-41fe-98e2-f35ea219267a/quotations/837ee82a-3142-4d79-ae0f-7f9639b77f54)

*"Economy, maternal capacity of reading and understanding. Our mothers are living in rural and further farm areas where they may not have charged mobile."* [1:93 ¶ 93 in interview analysis](https://go.atlasti.com/32e35350-0ea2-49d6-8d06-20d1c3d30273/documents/b5c0cb13-2834-41fe-98e2-f35ea219267a/quotations/913afe08-7f4d-4840-98b2-56af9a79f94b)

*"Uneducated, inability to read, electricity, Mothers may not want to use mobile phone"* [1:94 ¶ 94 in interview analysis](https://go.atlasti.com/32e35350-0ea2-49d6-8d06-20d1c3d30273/documents/b5c0cb13-2834-41fe-98e2-f35ea219267a/quotations/dfb5ca2c-6ff5-49f6-9f99-e556148c9f16)

*"All of them are solvable but may need time to solve"* [1:106 ¶ 106 in interview analysis](https://go.atlasti.com/32e35350-0ea2-49d6-8d06-20d1c3d30273/documents/b5c0cb13-2834-41fe-98e2-f35ea219267a/quotations/5495c258-d7c2-4993-914c-a1ff8991ed6c)

*"If anything necessary fulfilled, it is possible to implement."* [2:1 ¶ 9 in KII](https://go.atlasti.com/32e35350-0ea2-49d6-8d06-20d1c3d30273/documents/504671be-e9ef-4f58-8b5d-807935cb16a1/quotations/03e12670-67c3-45a5-9873-b916182d448d)

*"Yes, it will be difficult without infrastructure, it will be completely impossible for example if no network, internet, and professional/skill of these activities"* [2:18 ¶ 31 in KII](https://go.atlasti.com/32e35350-0ea2-49d6-8d06-20d1c3d30273/documents/504671be-e9ef-4f58-8b5d-807935cb16a1/quotations/6f30147e-8335-4f40-8a5d-131de0b74098)

*"This health facility is little bit less organized in materials computers"* [2:21 ¶ 34 in KII](https://go.atlasti.com/32e35350-0ea2-49d6-8d06-20d1c3d30273/documents/504671be-e9ef-4f58-8b5d-807935cb16a1/quotations/5602bfe9-45a0-4f4b-9dd3-b9b8e7ba1ca7)

*"I think rural health institutions have a better maternal and child health related service"* [2:22 ¶ 35 in KII](https://go.atlasti.com/32e35350-0ea2-49d6-8d06-20d1c3d30273/documents/504671be-e9ef-4f58-8b5d-807935cb16a1/quotations/3d82f977-7979-4bef-bc0b-422ec8f1f1bd)

*"I have to full resources to start this service. Awareness to health professionals"* [2:40 ¶ 60 in KII](https://go.atlasti.com/32e35350-0ea2-49d6-8d06-20d1c3d30273/documents/504671be-e9ef-4f58-8b5d-807935cb16a1/quotations/019ad53a-9d8e-4129-ace5-ac6a973206c1)

*"Fulfilling infrastructures and giving trainings or creating awareness both in staffs and communities"* [2:42 ¶ 63 in KII](https://go.atlasti.com/32e35350-0ea2-49d6-8d06-20d1c3d30273/documents/504671be-e9ef-4f58-8b5d-807935cb16a1/quotations/5f3e59c7-546d-4a9d-8fd0-fb0db2149f78)

*"May be it is more fit to towns and less likely for rural because of different reasons"* [3:1 ¶ 3 in Group discussion](https://go.atlasti.com/32e35350-0ea2-49d6-8d06-20d1c3d30273/documents/2569dddf-9aa1-42e1-9ece-a8d4dbbb0f25/quotations/8af0519c-3d76-4249-a13f-90f7c5bc3ba1)

*"Mobile is now part of the basic needs of our community. Mobile services are the most used thing in the community, so what possible culture could avoid using mHealth? Exactly there is no culture"* [3:53 ¶ 37 in Group discussion](https://go.atlasti.com/32e35350-0ea2-49d6-8d06-20d1c3d30273/documents/2569dddf-9aa1-42e1-9ece-a8d4dbbb0f25/quotations/10a26e99-6d5c-4502-bb21-5939287fb9c7)

*"Sustainability of the program is the concern, who will always look after computer"* [3:68 ¶ 55 in Group discussion](https://go.atlasti.com/32e35350-0ea2-49d6-8d06-20d1c3d30273/documents/2569dddf-9aa1-42e1-9ece-a8d4dbbb0f25/quotations/408e440a-ada6-44e8-a38e-a6ca9af41f35)

*"Resource to maintain the service. Interruption of supports, government concerns. Internet availability, and electricity interruptions and internet interruption might tempt the service"* [3:70 ¶ 58 in Group discussion](https://go.atlasti.com/32e35350-0ea2-49d6-8d06-20d1c3d30273/documents/2569dddf-9aa1-42e1-9ece-a8d4dbbb0f25/quotations/09c3b8f7-60b9-40a8-96ac-33f56c245e94)

**Challenges of mHealth implementation: Security**

*"Reading ability and of a person who can read in some cases can cause some problem, if children are not even learned"* [1:80 ¶ 79 in interview analysis](https://go.atlasti.com/32e35350-0ea2-49d6-8d06-20d1c3d30273/documents/b5c0cb13-2834-41fe-98e2-f35ea219267a/quotations/6b469c73-58ca-423a-9bf3-567ea3554278)

*"Privacy and confidentiality are not a problem as the exchange is between mother and professional"* [1:87 ¶ 85 in interview analysis](https://go.atlasti.com/32e35350-0ea2-49d6-8d06-20d1c3d30273/documents/b5c0cb13-2834-41fe-98e2-f35ea219267a/quotations/5b87a4f8-711b-419f-bf5c-e005e2582f79)

*"If everyone can access the information every women accept, there could be little security issue. The information going to mothers hand not anywhere else so why we fear"* [1:88 ¶ 86 in interview analysis](https://go.atlasti.com/32e35350-0ea2-49d6-8d06-20d1c3d30273/documents/b5c0cb13-2834-41fe-98e2-f35ea219267a/quotations/762ff471-52d9-44ac-bde7-b72d90008710)

*"Mothers do not hide information. Health professionals have ethics, so I don’t the any breaching on provider side"* [1:89 ¶ 87 in interview analysis](https://go.atlasti.com/32e35350-0ea2-49d6-8d06-20d1c3d30273/documents/b5c0cb13-2834-41fe-98e2-f35ea219267a/quotations/2329ef72-6dfb-40fa-9362-d417ab326516)

*"As health professionals we have to keep client privacy. Generally we don’t think this will breach client privacy. No security issue because the exchange of information is between professionals and mothers."* [1:90 ¶ 88 in interview analysis](https://go.atlasti.com/32e35350-0ea2-49d6-8d06-20d1c3d30273/documents/b5c0cb13-2834-41fe-98e2-f35ea219267a/quotations/893a852c-4f35-4b80-ace2-11795b88aebc)

*"Confidentiality and privacy should be kept by a person who registering information i.e. understanding of the message should be considered otherwise mother may go for who can read the message"* [1:100 ¶ 99 in interview analysis](https://go.atlasti.com/32e35350-0ea2-49d6-8d06-20d1c3d30273/documents/b5c0cb13-2834-41fe-98e2-f35ea219267a/quotations/a7544bed-2c01-4bca-a67d-42aba59960a8)

**Challenges of mHealth implementation: workload**

*"By the way mother knew their appointment but it is just the load of work they have everyday. Unless she says I am sick, she cannot allowed to go to the health institutions. It looks like there is no way to change but there should be some strategies for mother of daily laborer"* [1:2 ¶ 18 in interview analysis](https://go.atlasti.com/32e35350-0ea2-49d6-8d06-20d1c3d30273/documents/b5c0cb13-2834-41fe-98e2-f35ea219267a/quotations/0b3825ca-6e92-4c9d-9d4a-e1bdb5624aca)

*"Usually mothers know their date but since they are daily laborer, they do not have time, when they are not working and also want to come the have complain false reason to come for ANC"* [1:16 ¶ 29 in interview analysis](https://go.atlasti.com/32e35350-0ea2-49d6-8d06-20d1c3d30273/documents/b5c0cb13-2834-41fe-98e2-f35ea219267a/quotations/d2fa3e93-00f4-452b-926d-17b4f2553b3a)

*"Some mothers are the heavy workers and cannot avoid missing the appointment. This will fill our gap and reduce mortality"* [1:17 ¶ 30 in interview analysis](https://go.atlasti.com/32e35350-0ea2-49d6-8d06-20d1c3d30273/documents/b5c0cb13-2834-41fe-98e2-f35ea219267a/quotations/8de8a48d-0bba-4d7c-b78e-38702035e4a4)

*"this will increase pressure on professionals"* [1:31 ¶ 38 in interview analysis](https://go.atlasti.com/32e35350-0ea2-49d6-8d06-20d1c3d30273/documents/b5c0cb13-2834-41fe-98e2-f35ea219267a/quotations/a3089d5a-0dd7-446a-a954-3a702a2d8df5)

*"I appreciate to have technology assistance, but mothers do not forget appointment but they held back by their work intensity. May be they are giving more attention for their work than health"* [3:3 ¶ 4 in Group discussion](https://go.atlasti.com/32e35350-0ea2-49d6-8d06-20d1c3d30273/documents/2569dddf-9aa1-42e1-9ece-a8d4dbbb0f25/quotations/72985be9-ac09-48b7-be2c-aac16346eb09)

*"Daily laborers usually do not have time for themselves because of the strictness of the work."* [3:4 ¶ 4 in Group discussion](https://go.atlasti.com/32e35350-0ea2-49d6-8d06-20d1c3d30273/documents/2569dddf-9aa1-42e1-9ece-a8d4dbbb0f25/quotations/6389585a-0a48-4556-bf06-f7702975b9f6)

*"Reluctance, being bored of many messages over time, .pre occupation with important thing, hand-to-mouth life style, and daily laborer work"* [3:65 ¶ 52 in Group discussion](https://go.atlasti.com/32e35350-0ea2-49d6-8d06-20d1c3d30273/documents/2569dddf-9aa1-42e1-9ece-a8d4dbbb0f25/quotations/bdb2787e-3c5b-401f-a296-e0264192c9fb)

**SOLUTIONS THEME**

**Solutions: Family_help**

*"She can use her children’s education to use the service"* [1:12 ¶ 26 in interview analysis](https://go.atlasti.com/32e35350-0ea2-49d6-8d06-20d1c3d30273/documents/b5c0cb13-2834-41fe-98e2-f35ea219267a/quotations/afe90da2-6881-4ee1-a9f0-cf86e317880a)

*"The thing is there are learned children in every household, so possibly every mother can use this service if implemented."* [1:103 ¶ 103 in interview analysis](https://go.atlasti.com/32e35350-0ea2-49d6-8d06-20d1c3d30273/documents/b5c0cb13-2834-41fe-98e2-f35ea219267a/quotations/1955fc1b-ea6a-4409-a51b-289cc0e7d7cf)

*"Most women read the local language so if it can be in the local language it will be helpful. This means more than half of mothers can use this service. Almost every husband has a mobile and there taught children in every house to solve the maternal inability to read. A mobilizing community for maternal education and behavioral change is importan"* [1:107 ¶ 106 in interview analysis](https://go.atlasti.com/32e35350-0ea2-49d6-8d06-20d1c3d30273/documents/b5c0cb13-2834-41fe-98e2-f35ea219267a/quotations/3960b93f-2c4d-4d0a-b6bd-a14b6910ac7a)

*"Teaching households to use phone especially to have successful pregnancy and child development. Because of the importance of the service mother should request to have a phone nearby her. For uneducated mothers, Golmasa education, use of their children"* [1:108 ¶ 107 in interview analysis](https://go.atlasti.com/32e35350-0ea2-49d6-8d06-20d1c3d30273/documents/b5c0cb13-2834-41fe-98e2-f35ea219267a/quotations/f82c1470-a7aa-4572-bd8f-bdea31b00c84)

*"Women’s education and society overall should be promoted to learn at least enable them to read is important. Before application of mHealth overall discussion might be necessary with the whole community"* [1:110 ¶ 109 in interview analysis](https://go.atlasti.com/32e35350-0ea2-49d6-8d06-20d1c3d30273/documents/b5c0cb13-2834-41fe-98e2-f35ea219267a/quotations/a6ab369b-821a-4146-8905-9d2d56f1fdd2)

*"To get mothers using this program husband should take large responsibility, if we cannot do that they may even stand against the program. If the mother do not have mobile I think we can use husband, child, any phone in the house to access the mother. We have to use very possible strategies to get mother into this service"* [1:111 ¶ 110 in interview analysis](https://go.atlasti.com/32e35350-0ea2-49d6-8d06-20d1c3d30273/documents/b5c0cb13-2834-41fe-98e2-f35ea219267a/quotations/1f8fd70d-c9cb-4412-b4ba-faeaa12cf576)

*"The service indeed can be provided when any phone in house like spouse, children and even neighborhoods phone"* [3:30 ¶ 17 in Group discussion](https://go.atlasti.com/32e35350-0ea2-49d6-8d06-20d1c3d30273/documents/2569dddf-9aa1-42e1-9ece-a8d4dbbb0f25/quotations/a981c6c1-3c49-4bc5-8cd0-559b6a41022b)

*"Maternal education can be improved through adult education (Golmasa timhert). Educating female as a community and using women forum to reach and aware mothers"* [3:71 ¶ 60 in Group discussion](https://go.atlasti.com/32e35350-0ea2-49d6-8d06-20d1c3d30273/documents/2569dddf-9aa1-42e1-9ece-a8d4dbbb0f25/quotations/1f516008-035f-4cd4-94c8-b34b18115153)

**Solutions: Forums help**

*"For uneducated we have to teach through mothers forum, community forum, and adult education"* [1:11 ¶ 26 in interview analysis](https://go.atlasti.com/32e35350-0ea2-49d6-8d06-20d1c3d30273/documents/b5c0cb13-2834-41fe-98e2-f35ea219267a/quotations/b431b28e-45ac-480b-8ac7-3fbeda8ad83a)

*"Most women read the local language so if it can be in the local language it will be helpful. This means more than half of mothers can use this service. Almost every husband has a mobile and there taught children in every house to solve the maternal inability to read. A mobilizing community for maternal education and behavioral change is importan"* [1:107 ¶ 106 in interview analysis](https://go.atlasti.com/32e35350-0ea2-49d6-8d06-20d1c3d30273/documents/b5c0cb13-2834-41fe-98e2-f35ea219267a/quotations/3960b93f-2c4d-4d0a-b6bd-a14b6910ac7a)

*"using mothers forum, HDA, voluntary health team(በጎ ጤና ቡድን), community wing movement(የህዝብ ክንፍ ንቅናቄ), one-to-five (1 ለ 5), and using HEWs usual program. Mothers’ forum will be a nice thing to increase awareness of mothers. Using HEWs can also help the mother get aware of the mHealth care. In villages without electricity, there are solar centers for charging mobiles so no problem just we can support those centers"* [1:109 ¶ 108 in interview analysis](https://go.atlasti.com/32e35350-0ea2-49d6-8d06-20d1c3d30273/documents/b5c0cb13-2834-41fe-98e2-f35ea219267a/quotations/f19adc26-c109-486f-b0ad-ddc25ad4a80f)

*"Creating conducive political environment, creating awareness, using mothers forum appropriately, using voluntary health team of the kebeles, trying through health development army, using one-to-five political arrangement for information dissemination. When mothers take an intended service, previously there is a time when small hats provided for their children so may such an issues can be helpful if possible. And this promotes utilizations"* [1:112 ¶ 111 in interview analysis](https://go.atlasti.com/32e35350-0ea2-49d6-8d06-20d1c3d30273/documents/b5c0cb13-2834-41fe-98e2-f35ea219267a/quotations/93f2a2c8-2353-4722-9bcb-4db34325cdab)

*"Human development and health development army can improve all the limitation between HEWs, HC, and community"* [2:19 ¶ 32 in KII](https://go.atlasti.com/32e35350-0ea2-49d6-8d06-20d1c3d30273/documents/504671be-e9ef-4f58-8b5d-807935cb16a1/quotations/7ec2844b-7462-4a7b-872c-b7bd9733ba3b)

*"Maternal education can be improved through adult education (Golmasa timhert). Educating female as a community and using women forum to reach and aware mothers"* [3:71 ¶ 60 in Group discussion](https://go.atlasti.com/32e35350-0ea2-49d6-8d06-20d1c3d30273/documents/2569dddf-9aa1-42e1-9ece-a8d4dbbb0f25/quotations/1f516008-035f-4cd4-94c8-b34b18115153)

*"Using voluntary health team in each community to promote mHealth implications, using every contact to promote the mHealth service, working in collaboration with other stakeholders for resources and support, and using politics/government in area of difficult to support community mobilization."* [3:74 ¶ 63 in Group discussion](https://go.atlasti.com/32e35350-0ea2-49d6-8d06-20d1c3d30273/documents/2569dddf-9aa1-42e1-9ece-a8d4dbbb0f25/quotations/eb0f96a5-5f73-458d-a1ef-6a0c105baf00)

**Solutions: Professional_support**

*"Rural mothers do not have road, transport, money, and motivation or need support. mHealth"* [1:13 ¶ 27 in interview analysis](https://go.atlasti.com/32e35350-0ea2-49d6-8d06-20d1c3d30273/documents/b5c0cb13-2834-41fe-98e2-f35ea219267a/quotations/981c4d5f-2488-48f8-8614-d8c74fe52518)

*"It can change the community behavior and they may then become dependent on mHealth"* [1:14 ¶ 28 in interview analysis](https://go.atlasti.com/32e35350-0ea2-49d6-8d06-20d1c3d30273/documents/b5c0cb13-2834-41fe-98e2-f35ea219267a/quotations/60e88edb-2b48-4250-a016-66e6dbf5d70c)

*"We usually expect some challenges or struggle to make a new service breakthrough and accepted safely by every profession, mothers, and community at large. Creating awareness and finding a person to look after them"* [1:104 ¶ 104 in interview analysis](https://go.atlasti.com/32e35350-0ea2-49d6-8d06-20d1c3d30273/documents/b5c0cb13-2834-41fe-98e2-f35ea219267a/quotations/b8341656-65a4-4e82-b663-a4f49b518950)

*"Except primi gravida, all para gravida mothers knew the consequence of not using the counseling information in enough amounts. It is important to maintain that information flowing but behavioral change could be very important to solve this problem"* [3:22 ¶ 12 in Group discussion](https://go.atlasti.com/32e35350-0ea2-49d6-8d06-20d1c3d30273/documents/2569dddf-9aa1-42e1-9ece-a8d4dbbb0f25/quotations/1429826e-6865-4b45-a899-9d5c01c1cedb)

*"Large queues are not disrupting counseling service provision because we have another chance to get the mother"* [3:33 ¶ 19 in Group discussion](https://go.atlasti.com/32e35350-0ea2-49d6-8d06-20d1c3d30273/documents/2569dddf-9aa1-42e1-9ece-a8d4dbbb0f25/quotations/20bed780-04da-4553-b5e0-2d066fb38deb)

*"We really do not know what we are doing while this mHealth may change this so that we can know every one we serve."* [3:36 ¶ 20 in Group discussion](https://go.atlasti.com/32e35350-0ea2-49d6-8d06-20d1c3d30273/documents/2569dddf-9aa1-42e1-9ece-a8d4dbbb0f25/quotations/ad1b1b66-87f1-42dd-b38f-6d2120d60bdb)

*"Maternal education can be improved through adult education (Golmasa timhert). Educating female as a community and using women forum to reach and aware mothers"* [3:71 ¶ 60 in Group discussion](https://go.atlasti.com/32e35350-0ea2-49d6-8d06-20d1c3d30273/documents/2569dddf-9aa1-42e1-9ece-a8d4dbbb0f25/quotations/1f516008-035f-4cd4-94c8-b34b18115153)

*"Using HEWs to reach to lost or bored mothers. Considering children for those mother are not learned. Additionally, use of spouse phone is a big alternative, Involving partner in maternal and child care, and use of children phone"* [3:72 ¶ 61 in Group discussion](https://go.atlasti.com/32e35350-0ea2-49d6-8d06-20d1c3d30273/documents/2569dddf-9aa1-42e1-9ece-a8d4dbbb0f25/quotations/5c6d6feb-0a94-47ee-b190-cccd23c99a0a)

*"Increasing the maternal and child care to household level and intensive health education to ensure women aware of her problems. Community mobilization to motivate women to use mHealth might be also important. Using community wing political office to educate, motivate women and community can be used"* [3:73 ¶ 62 in Group discussion](https://go.atlasti.com/32e35350-0ea2-49d6-8d06-20d1c3d30273/documents/2569dddf-9aa1-42e1-9ece-a8d4dbbb0f25/quotations/881a0c24-18f9-4dc3-ad49-e4b4d5afd9c8)
