# Supplementary material for: The role of mHealth intervention to improve maternal and child health: A provider-based qualitative study in Southern Ethiopia
Source: PLoS One. 2024 Feb 8;19(2):e0295539. doi: 10.1371/journal.pone.0295539 (PMC10852240; doi:10.1371/journal.pone.0295539)
Supplement: S1 File — (ZIP) [file pone.0295539.s003.zip › supplementars evidences/Report final.pdf]

Project: Mhealth

Report created by Girma Gilano on 3/20/2023

Quotation Report – Grouped by: Codes

All (226) quotations

Acceptability: Fiting_current

Created by Girma Gilano on 3/13/2023

9 Quotations:

1:20 ¶ 32 in interview analysis

Text quotation

Created by Girma Gilano on 3/8/2023

It fits very well, but I think the process will take a long time to adapt

1 Codes:

Acceptability: Fiting_current

9 Quotations:

1:20 ¶ 32, It fits very well, but I think the process will take a long time to ad… in interview analysis / 1:27 ¶ 36, I think it can go with the previous service in interview analysis / 1:32 ¶ 38, There is no culture to my knowledge that can hinder the service in interview analysis / 1:33 ¶ 39, I do not think there is any opposing culture anywhere in interview analysis / 2:30 ¶ 46, No culture in opposite to this service in KII / 2:31 ¶ 47, Every culture is improving through technology and it is everywhere, so… in KII / 2:45 ¶ 65, I hope they will accept easily because this is the part of health comm… in KII / 3:7 ¶ 6, Women always want something that allows better service so this can be… in Group discussion / 3:14 ¶ 8, They community as a whole may see this positively and acknowledge the… in Group discussion

1:27 ¶ 36 in interview analysis

Text quotation

Created by Girma Gilano on 3/8/2023

I think it can go with the previous service

1 Codes:

- **Acceptability: Fiting_current**

9 Quotations:

1:20 ¶ 32, It fits very well, but I think the process will take a long time to ad… in interview analysis / 1:27 ¶ 36, I think it can go with the previous service in interview analysis / 1:32 ¶ 38, There is no culture to my knowledge that can hinder the service in interview analysis / 1:33 ¶ 39, I do not think there is any opposing culture anywhere in interview analysis / 2:30 ¶ 46, No culture in opposite to this service in KII / 2:31 ¶ 47, Every culture is improving through technology and it is everywhere, so… in KII / 2:45 ¶ 65, I hope they will accept easily because this is the part of health comm… in KII / 3:7 ¶ 6, Women always want something that allows better service so this can be… in Group discussion / 3:14 ¶ 8, They community as a whole may see this positively and acknowledge the… in Group discussion

1:32 ¶ 38 in interview analysis

Text quotation

Created by Girma Gilano on 3/8/2023

There is no culture to my knowledge that can hinder the service

1 Codes:

- **Acceptability: Fiting_current**

9 Quotations:

1:20 ¶ 32, It fits very well, but I think the process will take a long time to ad… in interview analysis / 1:27 ¶ 36, I think it can go with the previous service in interview analysis / 1:32 ¶ 38, There is no culture to my knowledge that can hinder the service in interview analysis / 1:33 ¶ 39, I do not think there is any opposing culture anywhere in interview analysis / 2:30 ¶ 46, No culture in opposite to this service in KII / 2:31 ¶ 47, Every culture is improving through technology and it is everywhere, so… in KII / 2:45 ¶ 65, I hope they will accept easily because this is the part of health comm… in KII / 3:7 ¶ 6, Women always want something that allows better service so this can be… in Group discussion / 3:14 ¶ 8, They community as a whole may see this positively and acknowledge the… in Group discussion

1:33 ¶ 39 in interview analysis

Text quotation

Created by Girma Gilano on 3/8/2023

I do not think there is any opposing culture anywhere

1 Codes:

- **Acceptability: Fiting_current**

9 Quotations:

1:20 ¶ 32, It fits very well, but I think the process will take a long time to ad… in interview analysis / 1:27 ¶ 36, I think it can go with the previous service in interview analysis / 1:32 ¶ 38, There is no culture to my knowledge that can hinder the service in interview analysis / 1:33 ¶ 39, I do not think there is any opposing culture anywhere in interview analysis / 2:30 ¶ 46, No culture in opposite to this service in KII /

2:31 ¶ 47, Every culture is improving through technology and it is everywhere, so… in KII / 2:45 ¶ 65, I hope they will accept easily because this is the part of health comm… in KII / 3:7 ¶ 6, Women always want something that allows better service so this can be… in Group discussion / 3:14 ¶ 8, They community as a whole may see this positively and acknowledge the… in Group discussion

2:30 ¶ 46 in KII

Text quotation

Created by Girma Gilano on 3/9/2023

No culture in opposite to this service

1 Codes:

- **Acceptability: Fiting_current**

9 Quotations:

1:20 ¶ 32, It fits very well, but I think the process will take a long time to ad… in interview analysis / 1:27 ¶ 36, I think it can go with the previous service in interview analysis / 1:32 ¶ 38, There is no culture to my knowledge that can hinder the service in interview analysis / 1:33 ¶ 39, I do not think there is any opposing culture anywhere in interview analysis / 2:30 ¶ 46, No culture in opposite to this service in KII / 2:31 ¶ 47, Every culture is improving through technology and it is everywhere, so… in KII / 2:45 ¶ 65, I hope they will accept easily because this is the part of health comm… in KII / 3:7 ¶ 6, Women always want something that allows better service so this can be… in Group discussion / 3:14 ¶ 8, They community as a whole may see this positively and acknowledge the… in Group discussion

2:31 ¶ 47 in KII

Text quotation

Created by Girma Gilano on 3/9/2023

Every culture is improving through technology and it is everywhere, so whatever technology is accepted no opposition

1 Codes:

- **Acceptability: Fiting_current**

9 Quotations:

1:20 ¶ 32, It fits very well, but I think the process will take a long time to ad… in interview analysis / 1:27 ¶ 36, I think it can go with the previous service in interview analysis / 1:32 ¶ 38, There is no culture to my knowledge that can hinder the service in interview analysis / 1:33 ¶ 39, I do not think there is any opposing culture anywhere in interview analysis / 2:30 ¶ 46, No culture in opposite to this service in KII / 2:31 ¶ 47, Every culture is improving through technology and it is everywhere, so… in KII / 2:45 ¶ 65, I hope they will accept easily because this is the part of health comm… in KII / 3:7 ¶ 6, Women always want something that allows better service so this can be… in Group discussion / 3:14 ¶ 8, They community as a whole may see this positively and acknowledge the… in Group discussion

2:45 ¶ 65 in KII

Text quotation

Created by Girma Gilano on 3/9/2023

I hope they will accept easily because this is the part of health communication and no one should because there nothing new here

1 Codes:

- **Acceptability: Fiting_current**

9 Quotations:

1:20 ¶ 32, It fits very well, but I think the process will take a long time to ad… in interview analysis / 1:27 ¶ 36, I think it can go with the previous service in interview analysis / 1:32 ¶ 38, There is no culture to my knowledge that can hinder the service in interview analysis / 1:33 ¶ 39, I do not think there is any opposing culture anywhere in interview analysis / 2:30 ¶ 46, No culture in opposite to this service in KII / 2:31 ¶ 47, Every culture is improving through technology and it is everywhere, so… in KII / 2:45 ¶ 65, I hope they will accept easily because this is the part of health comm… in KII / 3:7 ¶ 6, Women always want something that allows better service so this can be… in Group discussion / 3:14 ¶ 8, They community as a whole may see this positively and acknowledge the… in Group discussion

3:7 ¶ 6 in Group discussion

Text quotation

Created by Girma Gilano on 3/9/2023

Women always want something that allows better service so this can be important

2 Codes:

- **Acceptability: Fiting_current**

9 Quotations:

1:20 ¶ 32, It fits very well, but I think the process will take a long time to ad… in interview analysis / 1:27 ¶ 36, I think it can go with the previous service in interview analysis / 1:32 ¶ 38, There is no culture to my knowledge that can hinder the service in interview analysis / 1:33 ¶ 39, I do not think there is any opposing culture anywhere in interview analysis / 2:30 ¶ 46, No culture in opposite to this service in KII / 2:31 ¶ 47, Every culture is improving through technology and it is everywhere, so… in KII / 2:45 ¶ 65, I hope they will accept easily because this is the part of health comm… in KII / 3:7 ¶ 6, Women always want something that allows better service so this can be… in Group discussion / 3:14 ¶ 8, They community as a whole may see this positively and acknowledge the… in Group discussion

- **Acceptability: Technology_care**

13 Quotations:

1:22 ¶ 33, revious services are not different but this is automated, evidenced, a… in interview analysis / 2:2 ¶ 10, I feel happy because it is modernized in KII / 2:5 ¶ 14, Am happy that finally we will have technology to support our client es… in KII / 2:12 ¶ 24, I think they will be happy. Everyone love technology whatever it is. E… in KII / 2:32 ¶ 50, People accept as something modern and will be happy in KII / 2:37 ¶ 56, People currently eager to have new technology so if no payment for the… in KII / 2:38 ¶ 57, I think it will not be possible after sometime to live without or work… in KII / 2:41 ¶ 62, Health education is necessary and our community is active for such new… in KII / 3:7 ¶ 6, Women always want

something that allows better service so this can be… in Group discussion / 3:14 ¶ 8, They community as a whole may see this positively and acknowledge the… in Group discussion / 3:31 ¶ 17, It is interesting to use the technology at this level, so am happy. Mo… in Group discussion / 3:55 ¶ 40, ccept the technology as the part of easing their work concerns. Awaren… in Group discussion / 3:69 ¶ 56, Expectation of mother to get SMS to take service, and otherwise consid… in Group discussion

3:14 ¶ 8 in Group discussion

Text quotation

Created by Girma Gilano on 3/9/2023

They community as a whole may see this positively and acknowledge the real effort

2 Codes:

- **Acceptability: Fiting_current**

9 Quotations:

1:20 ¶ 32, It fits very well, but I think the process will take a long time to ad… in interview analysis / 1:27 ¶ 36, I think it can go with the previous service in interview analysis / 1:32 ¶ 38, There is no culture to my knowledge that can hinder the service in interview analysis / 1:33 ¶ 39, I do not think there is any opposing culture anywhere in interview analysis / 2:30 ¶ 46, No culture in opposite to this service in KII / 2:31 ¶ 47, Every culture is improving through technology and it is everywhere, so… in KII / 2:45 ¶ 65, I hope they will accept easily because this is the part of health comm… in KII / 3:7 ¶ 6, Women always want something that allows better service so this can be… in Group discussion / 3:14 ¶ 8, They community as a whole may see this positively and acknowledge the… in Group discussion

- **Acceptability: Technology_care**

13 Quotations:

1:22 ¶ 33, revious services are not different but this is automated, evidenced, a… in interview analysis / 2:2 ¶ 10, I feel happy because it is modernized in KII / 2:5 ¶ 14, Am happy that finally we will have technology to support our client es… in KII / 2:12 ¶ 24, I think they will be happy. Everyone love technology whatever it is. E… in KII / 2:32 ¶ 50, People accept as something modern and will be happy in KII / 2:37 ¶ 56, People currently eager to have new technology so if no payment for the… in KII / 2:38 ¶ 57, I think it will not be possible after sometime to live without or work… in KII / 2:41 ¶ 62, Health education is necessary and our community is active for such new… in KII / 3:7 ¶ 6, Women always want something that allows better service so this can be… in Group discussion / 3:14 ¶ 8, They community as a whole may see this positively and acknowledge the… in Group discussion / 3:31 ¶ 17, It is interesting to use the technology at this level, so am happy. Mo… in Group discussion / 3:55 ¶ 40, ccept the technology as the part of easing their work concerns. Awaren… in Group discussion / 3:69 ¶ 56, Expectation of mother to get SMS to take service, and otherwise consid… in Group discussion

---

Acceptability: Improve_care

Created by Girma Gilano on 3/13/2023

2 Quotations:

1:29 ¶ 37 in interview analysis

Text quotation

Created by Girma Gilano on 3/8/2023

improve the existing service in the positive directio

1 Codes:

- **Acceptability: Improve_care**

2 Quotations:

1:29 ¶ 37, improve the existing service in the positive directio in interview analysis / 2:39 ¶ 58, We have previous requirements of following patients, so this strengthe… in KII

2:39 ¶ 58 in KII

Text quotation

Created by Girma Gilano on 3/9/2023

We have previous requirements of following patients, so this strengthen the previous idea in practice. This time I don’t anyone will oppose this

2 Codes:

- **Acceptability: Improve_care**

2 Quotations:

1:29 ¶ 37, improve the existing service in the positive directio in interview analysis / 2:39 ¶ 58, We have previous requirements of following patients, so this strengthe… in KII

- **Acceptability: support_professionals**

10 Quotations:

1:47 ¶ 54, If this is to support but not contradict with HEWs work it is importa… in interview analysis / 1:52 ¶ 58, but she may also have to come to health professional for further inves… in interview analysis / 2:13 ¶ 25, I think our institution workers will welcome this more than anything.… in KII / 2:16 ¶ 28, Health professionals’ role should be high to make it accepted by the m… in KII / 2:17 ¶ 29, is is can easy our workers job, they will happy to accept as peacefull… in KII / 2:39 ¶ 58, We have previous requirements of following patients, so this strengthe… in KII / 2:41 ¶ 62, Health education is necessary and our community is active for such new… in KII / 2:44 ¶ 64, Making people understand the service objectives especially health prof… in KII / 3:31 ¶ 17, It is interesting to use the technology at this level, so am happy. Mo… in Group discussion / 3:55 ¶ 40, ccept the technology as the part of easing their work concerns. Awaren… in Group discussion

Acceptability: support_professionals

Created by Girma Gilano on 3/13/2023

10 Quotations:

1:47 ¶ 54 in interview analysis

Text quotation

Created by Girma Gilano on 3/8/2023

If this is to support but not contradict with HEWs work it is important

1 Codes:

Acceptability: support_professionals

10 Quotations:

1:47 ¶ 54, If this is to support but not contradict with HEWs work it is importa… in interview analysis / 1:52 ¶ 58, but she may also have to come to health professional for further inves… in interview analysis / 2:13 ¶ 25, I think our institution workers will welcome this more than anything.… in KII / 2:16 ¶ 28, Health professionals' role should be high to make it accepted by the m… in KII / 2:17 ¶ 29, is is can easy our workers job, they will happy to accept as peacefull… in KII / 2:39 ¶ 58, We have previous requirements of following patients, so this strengthe… in KII / 2:41 ¶ 62, Health education is necessary and our community is active for such new… in KII / 2:44 ¶ 64, Making people understand the service objectives especially health prof… in KII / 3:31 ¶ 17, It is interesting to use the technology at this level, so am happy. Mo… in Group discussion / 3:55 ¶ 40, ccept the technology as the part of easing their work concerns. Awaren… in Group discussion

1:52 ¶ 58 in interview analysis

Text quotation

Created by Girma Gilano on 3/8/2023

but she may also have to come to health professional for further investigations beside information access.

1 Codes:

Acceptability: support_professionals

10 Quotations:

1:47 ¶ 54, If this is to support but not contradict with HEWs work it is importa… in interview analysis / 1:52 ¶ 58, but she may also have to come to health professional for further inves… in interview analysis / 2:13 ¶ 25, I think our institution workers will welcome this more than anything.… in KII / 2:16 ¶ 28, Health professionals' role should be high to make it accepted by the m… in KII / 2:17 ¶ 29, is is can easy our workers job, they will happy to accept as peacefull… in KII / 2:39 ¶ 58, We have previous requirements

of following patients, so this strengthe… in KII / 2:41 ¶ 62, Health education is necessary and our community is active for such new… in KII / 2:44 ¶ 64, Making people understand the service objectives especially health prof… in KII / 3:31 ¶ 17, It is interesting to use the technology at this level, so am happy. Mo… in Group discussion / 3:55 ¶ 40, ccept the technology as the part of easing their work concerns. Awaren… in Group discussion

2:13 ¶ 25 in KII

Text quotation

Created by Girma Gilano on 3/9/2023

I think our institution workers will welcome this more than anything. This is modernizing what everyone is eager to have

1 Codes:

- **Acceptability: support_professionals**

10 Quotations:

1:47 ¶ 54, If this is to support but not contradict with HEWs work it is importa… in interview analysis / 1:52 ¶ 58, but she may also have to come to health professional for further inves… in interview analysis / 2:13 ¶ 25, I think our institution workers will welcome this more than anything.… in KII / 2:16 ¶ 28, Health professionals' role should be high to make it accepted by the m… in KII / 2:17 ¶ 29, is is can easy our workers job, they will happy to accept as peacefull… in KII / 2:39 ¶ 58, We have previous requirements of following patients, so this strengthe… in KII / 2:41 ¶ 62, Health education is necessary and our community is active for such new… in KII / 2:44 ¶ 64, Making people understand the service objectives especially health prof… in KII / 3:31 ¶ 17, It is interesting to use the technology at this level, so am happy. Mo… in Group discussion / 3:55 ¶ 40, ccept the technology as the part of easing their work concerns. Awaren… in Group discussion

2:16 ¶ 28 in KII

Text quotation

Created by Girma Gilano on 3/9/2023

Health professionals' role should be high to make it accepted by the mothers and community

1 Codes:

- **Acceptability: support_professionals**

10 Quotations:

1:47 ¶ 54, If this is to support but not contradict with HEWs work it is importa… in interview analysis / 1:52 ¶ 58, but she may also have to come to health professional for further inves… in interview analysis / 2:13 ¶ 25, I think our institution workers will welcome this more than anything.… in KII / 2:16 ¶ 28, Health professionals' role should be high to make it accepted by the m… in KII / 2:17 ¶ 29, is is can easy our workers job, they will happy to accept as peacefull… in KII / 2:39 ¶ 58, We have previous requirements of following patients, so this strengthe… in KII / 2:41 ¶ 62, Health education is necessary and our community is active for such new… in KII / 2:44 ¶ 64, Making people understand the service objectives especially health prof… in KII / 3:31 ¶ 17, It is interesting to use the technology at this level, so am

happy. Mo… in Group discussion / 3:55 ¶ 40, ccept the technology as the part of easing their work concerns. Awaren… in Group discussion

2:17 ¶ 29 in KII

Text quotation

Created by Girma Gilano on 3/9/2023

is is can easy our workers job, they will happy to accept as peacefully as anything

1 Codes:

- **Acceptability: support_professionals**

10 Quotations:

1:47 ¶ 54, If this is to support but not contradict with HEWs work it is importa… in interview analysis / 1:52 ¶ 58, but she may also have to come to health professional for further inves… in interview analysis / 2:13 ¶ 25, I think our institution workers will welcome this more than anything.… in KII / 2:16 ¶ 28, Health professionals' role should be high to make it accepted by the m… in KII / 2:17 ¶ 29, is is can easy our workers job, they will happy to accept as peacefull… in KII / 2:39 ¶ 58, We have previous requirements of following patients, so this strengthe… in KII / 2:41 ¶ 62, Health education is necessary and our community is active for such new… in KII / 2:44 ¶ 64, Making people understand the service objectives especially health prof… in KII / 3:31 ¶ 17, It is interesting to use the technology at this level, so am happy. Mo… in Group discussion / 3:55 ¶ 40, ccept the technology as the part of easing their work concerns. Awaren… in Group discussion

2:39 ¶ 58 in KII

Text quotation

Created by Girma Gilano on 3/9/2023

We have previous requirements of following patients, so this strengthen the previous idea in practice. This time I don't anyone will oppose this

2 Codes:

- **Acceptability: Improve_care**

2 Quotations:

1:29 ¶ 37, improve the existing service in the positive directio in interview analysis / 2:39 ¶ 58, We have previous requirements of following patients, so this strengthe… in KII

- **Acceptability: support_professionals**

10 Quotations:

1:47 ¶ 54, If this is to support but not contradict with HEWs work it is importa… in interview analysis / 1:52 ¶ 58, but she may also have to come to health professional for further inves… in interview analysis / 2:13 ¶ 25, I think our institution workers will welcome this more than anything.… in KII / 2:16 ¶ 28, Health professionals' role should be high to make it accepted by the m… in KII / 2:17 ¶ 29, is is can easy our

workers job, they will happy to accept as peacefull… in KII / 2:39 ¶ 58, We have previous requirements of following patients, so this strengthe… in KII / 2:41 ¶ 62, Health education is necessary and our community is active for such new… in KII / 2:44 ¶ 64, Making people understand the service objectives especially health prof… in KII / 3:31 ¶ 17, It is interesting to use the technology at this level, so am happy. Mo… in Group discussion / 3:55 ¶ 40, ccept the technology as the part of easing their work concerns. Awaren… in Group discussion

2:41 ¶ 62 in KII

Text quotation

Created by Girma Gilano on 3/9/2023

Health education is necessary and our community is active for such new technology based services

2 Codes:

- **Acceptability: support_professionals**

10 Quotations:

1:47 ¶ 54, If this is to support but not contradict with HEWs work it is importa… in interview analysis / 1:52 ¶ 58, but she may also have to come to health professional for further inves… in interview analysis / 2:13 ¶ 25, I think our institution workers will welcome this more than anything.… in KII / 2:16 ¶ 28, Health professionals' role should be high to make it accepted by the m… in KII / 2:17 ¶ 29, is is can easy our workers job, they will happy to accept as peacefull… in KII / 2:39 ¶ 58, We have previous requirements of following patients, so this strengthe… in KII / 2:41 ¶ 62, Health education is necessary and our community is active for such new… in KII / 2:44 ¶ 64, Making people understand the service objectives especially health prof… in KII / 3:31 ¶ 17, It is interesting to use the technology at this level, so am happy. Mo… in Group discussion / 3:55 ¶ 40, ccept the technology as the part of easing their work concerns. Awaren… in Group discussion

- **Acceptability: Technology_care**

13 Quotations:

1:22 ¶ 33, revious services are not different but this is automated, evidenced, a… in interview analysis / 2:2 ¶ 10, I feel happy because it is modernized in KII / 2:5 ¶ 14, Am happy that finally we will have technology to support our client es… in KII / 2:12 ¶ 24, I think they will be happy. Everyone love technology whatever it is. E… in KII / 2:32 ¶ 50, People accept as something modern and will be happy in KII / 2:37 ¶ 56, People currently eager to have new technology so if no payment for the… in KII / 2:38 ¶ 57, I think it will not be possible after sometime to live without or work… in KII / 2:41 ¶ 62, Health education is necessary and our community is active for such new… in KII / 3:7 ¶ 6, Women always want something that allows better service so this can be… in Group discussion / 3:14 ¶ 8, They community as a whole may see this positively and acknowledge the… in Group discussion / 3:31 ¶ 17, It is interesting to use the technology at this level, so am happy. Mo… in Group discussion / 3:55 ¶ 40, ccept the technology as the part of easing their work concerns. Awaren… in Group discussion / 3:69 ¶ 56, Expectation of mother to get SMS to take service, and otherwise consid… in Group discussion

2:44 ¶ 64 in KII

Text quotation

Created by Girma Gilano on 3/9/2023

Making people understand the service objectives especially health professionals

1 Codes:

● Acceptability: support_professionals

10 Quotations:

1:47 ¶ 54, If this is to support but not contradict with HEWs work it is importa… in interview analysis / 1:52 ¶ 58, but she may also have to come to health professional for further inves… in interview analysis / 2:13 ¶ 25, I think our institution workers will welcome this more than anything.… in KII / 2:16 ¶ 28, Health professionals' role should be high to make it accepted by the m… in KII / 2:17 ¶ 29, is is can easy our workers job, they will happy to accept as peacefull… in KII / 2:39 ¶ 58, We have previous requirements of following patients, so this strengthe… in KII / 2:41 ¶ 62, Health education is necessary and our community is active for such new… in KII / 2:44 ¶ 64, Making people understand the service objectives especially health prof… in KII / 3:31 ¶ 17, It is interesting to use the technology at this level, so am happy. Mo… in Group discussion / 3:55 ¶ 40, ccept the technology as the part of easing their work concerns. Awaren… in Group discussion

3:31 ¶ 17 in Group discussion

Text quotation

Created by Girma Gilano on 3/9/2023

It is interesting to use the technology at this level, so am happy. Mothers usually fear whatever health professionals order and can be comply when they ordered to meet on phone for the next information.

2 Codes:

● Acceptability: support_professionals

10 Quotations:

1:47 ¶ 54, If this is to support but not contradict with HEWs work it is importa… in interview analysis / 1:52 ¶ 58, but she may also have to come to health professional for further inves… in interview analysis / 2:13 ¶ 25, I think our institution workers will welcome this more than anything.… in KII / 2:16 ¶ 28, Health professionals' role should be high to make it accepted by the m… in KII / 2:17 ¶ 29, is is can easy our workers job, they will happy to accept as peacefull… in KII / 2:39 ¶ 58, We have previous requirements of following patients, so this strengthe… in KII / 2:41 ¶ 62, Health education is necessary and our community is active for such new… in KII / 2:44 ¶ 64, Making people understand the service objectives especially health prof… in KII / 3:31 ¶ 17, It is interesting to use the technology at this level, so am happy. Mo… in Group discussion / 3:55 ¶ 40, ccept the technology as the part of easing their work concerns. Awaren… in Group discussion

● Acceptability: Technology_care

13 Quotations:

1:22 ¶ 33, revious services are not different but this is automated, evidenced, a… in interview analysis / 2:2 ¶ 10, I feel happy because it is modernized in KII / 2:5 ¶ 14, Am happy that finally we will have technology to support our client es… in KII / 2:12 ¶ 24, I think they will be happy. Everyone love technology whatever it is. E… in KII / 2:32 ¶ 50, People accept as something modern and will be happy in KII / 2:37 ¶ 56, People currently eager to have new technology so if no payment for the… in KII / 2:38

¶ 57, I think it will not be possible after sometime to live without or work… in KII / 2:41 ¶ 62, Health education is necessary and our community is active for such new… in KII / 3:7 ¶ 6, Women always want something that allows better service so this can be… in Group discussion / 3:14 ¶ 8, They community as a whole may see this positively and acknowledge the… in Group discussion / 3:31 ¶ 17, It is interesting to use the technology at this level, so am happy. Mo… in Group discussion / 3:55 ¶ 40, ccept the technology as the part of easing their work concerns. Awaren… in Group discussion / 3:69 ¶ 56, Expectation of mother to get SMS to take service, and otherwise consid… in Group discussion

3:55 ¶ 40 in Group discussion

Text quotation

Created by Girma Gilano on 3/9/2023

ccept the technology as the part of easing their work concerns. Awareness creation among mothers and community is critical

2 Codes:

- **Acceptability: support_professionals**

10 Quotations:

1:47 ¶ 54, If this is to support but not contradict with HEWs work it is importa… in interview analysis / 1:52 ¶ 58, but she may also have to come to health professional for further inves… in interview analysis / 2:13 ¶ 25, I think our institution workers will welcome this more than anything.… in KII / 2:16 ¶ 28, Health professionals' role should be high to make it accepted by the m… in KII / 2:17 ¶ 29, is is can easy our workers job, they will happy to accept as peacefull… in KII / 2:39 ¶ 58, We have previous requirements of following patients, so this strengthe… in KII / 2:41 ¶ 62, Health education is necessary and our community is active for such new… in KII / 2:44 ¶ 64, Making people understand the service objectives especially health prof… in KII / 3:31 ¶ 17, It is interesting to use the technology at this level, so am happy. Mo… in Group discussion / 3:55 ¶ 40, ccept the technology as the part of easing their work concerns. Awaren… in Group discussion

- **Acceptability: Technology_care**

13 Quotations:

1:22 ¶ 33, revious services are not different but this is automated, evidenced, a… in interview analysis / 2:2 ¶ 10, I feel happy because it is modernized in KII / 2:5 ¶ 14, Am happy that finally we will have technology to support our client es… in KII / 2:12 ¶ 24, I think they will be happy. Everyone love technology whatever it is. E… in KII / 2:32 ¶ 50, People accept as something modern and will be happy in KII / 2:37 ¶ 56, People currently eager to have new technology so if no payment for the… in KII / 2:38 ¶ 57, I think it will not be possible after sometime to live without or work… in KII / 2:41 ¶ 62, Health education is necessary and our community is active for such new… in KII / 3:7 ¶ 6, Women always want something that allows better service so this can be… in Group discussion / 3:14 ¶ 8, They community as a whole may see this positively and acknowledge the… in Group discussion / 3:31 ¶ 17, It is interesting to use the technology at this level, so am happy. Mo… in Group discussion / 3:55 ¶ 40, ccept the technology as the part of easing their work concerns. Awaren… in Group discussion / 3:69 ¶ 56, Expectation of mother to get SMS to take service, and otherwise consid… in Group discussion

---

Acceptability: Technology_care

Created by Girma Gilano on 3/13/2023

13 Quotations:

1:22 ¶ 33 in interview analysis

Text quotation

Created by Girma Gilano on 3/8/2023

revious services are not different but this is automated, evidenced, and easy to apply

1 Codes:

- **Acceptability: Technology_care**

13 Quotations:

1:22 ¶ 33, revious services are not different but this is automated, evidenced, a… in interview analysis / 2:2 ¶ 10, I feel happy because it is modernized in KII / 2:5 ¶ 14, Am happy that finally we will have technology to support our client es… in KII / 2:12 ¶ 24, I think they will be happy. Everyone love technology whatever it is. E… in KII / 2:32 ¶ 50, People accept as something modern and will be happy in KII / 2:37 ¶ 56, People currently eager to have new technology so if no payment for the… in KII / 2:38 ¶ 57, I think it will not be possible after sometime to live without or work… in KII / 2:41 ¶ 62, Health education is necessary and our community is active for such new… in KII / 3:7 ¶ 6, Women always want something that allows better service so this can be… in Group discussion / 3:14 ¶ 8, They community as a whole may see this positively and acknowledge the… in Group discussion / 3:31 ¶ 17, It is interesting to use the technology at this level, so am happy. Mo… in Group discussion / 3:55 ¶ 40, ccept the technology as the part of easing their work concerns. Awaren… in Group discussion / 3:69 ¶ 56, Expectation of mother to get SMS to take service, and otherwise consid… in Group discussion

2:2 ¶ 10 in KII

Text quotation

Created by Girma Gilano on 3/9/2023, **modified** by Girma Gilano on 3/9/2023

I feel happy because it is modernized

1 Codes:

- **Acceptability: Technology_care**

13 Quotations:

1:22 ¶ 33, revious services are not different but this is automated, evidenced, a… in interview analysis / 2:2 ¶ 10, I feel happy because it is modernized in KII / 2:5 ¶ 14, Am happy that finally we will have technology to support our client es… in KII / 2:12 ¶ 24, I think they will be happy. Everyone love technology whatever it is. E… in KII / 2:32 ¶ 50, People accept as something modern and will be happy in KII / 2:37 ¶ 56, People currently eager to have new technology so if no payment for the… in KII / 2:38 ¶ 57, I think it will not be possible after sometime to live without or work… in KII / 2:41 ¶ 62, Health education is necessary and our community is active for such new… in KII / 3:7 ¶ 6, Women always want something that allows better service so this can be… in Group discussion / 3:14 ¶ 8, They community as a whole may see this positively and acknowledge the… in Group discussion / 3:31 ¶ 17, It is interesting

to use the technology at this level, so am happy. Mo… in Group discussion / 3:55 ¶ 40, ccept the technology as the part of easing their work concerns. Awaren… in Group discussion / 3:69 ¶ 56, Expectation of mother to get SMS to take service, and otherwise consid… in Group discussion

2:5 ¶ 14 in KII

Text quotation

Created by Girma Gilano on 3/9/2023

Am happy that finally we will have technology to support our client especially for appoint

1 Codes:

- **Acceptability: Technology_care**

13 Quotations:

1:22 ¶ 33, revious services are not different but this is automated, evidenced, a… in interview analysis / 2:2 ¶ 10, I feel happy because it is modernized in KII / 2:5 ¶ 14, Am happy that finally we will have technology to support our client es… in KII / 2:12 ¶ 24, I think they will be happy. Everyone love technology whatever it is. E… in KII / 2:32 ¶ 50, People accept as something modern and will be happy in KII / 2:37 ¶ 56, People currently eager to have new technology so if no payment for the… in KII / 2:38 ¶ 57, I think it will not be possible after sometime to live without or work… in KII / 2:41 ¶ 62, Health education is necessary and our community is active for such new… in KII / 3:7 ¶ 6, Women always want something that allows better service so this can be… in Group discussion / 3:14 ¶ 8, They community as a whole may see this positively and acknowledge the… in Group discussion / 3:31 ¶ 17, It is interesting to use the technology at this level, so am happy. Mo… in Group discussion / 3:55 ¶ 40, ccept the technology as the part of easing their work concerns. Awaren… in Group discussion / 3:69 ¶ 56, Expectation of mother to get SMS to take service, and otherwise consid… in Group discussion

2:12 ¶ 24 in KII

Text quotation

Created by Girma Gilano on 3/9/2023

I think they will be happy. Everyone love technology whatever it is. Everyone will accept, we are part of the technology

1 Codes:

- **Acceptability: Technology_care**

13 Quotations:

1:22 ¶ 33, revious services are not different but this is automated, evidenced, a… in interview analysis / 2:2 ¶ 10, I feel happy because it is modernized in KII / 2:5 ¶ 14, Am happy that finally we will have technology to support our client es… in KII / 2:12 ¶ 24, I think they will be happy. Everyone love technology whatever it is. E… in KII / 2:32 ¶ 50, People accept as something modern and will be happy in KII / 2:37 ¶ 56, People currently eager to have new technology so if no payment for the… in KII / 2:38 ¶ 57, I think it will not be possible after sometime to live without or work… in KII / 2:41 ¶ 62, Health education is necessary and our community is active for such new… in KII / 3:7 ¶ 6, Women always want something that allows better service so this can be… in Group discussion / 3:14 ¶ 8, They community as a whole may see this positively and acknowledge the… in Group discussion / 3:31 ¶ 17, It is interesting

to use the technology at this level, so am happy. Mo… in Group discussion / 3:55 ¶ 40, ccept the technology as the part of easing their work concerns. Awaren… in Group discussion / 3:69 ¶ 56, Expectation of mother to get SMS to take service, and otherwise consid… in Group discussion

2:32 ¶ 50 in KII

Text quotation

Created by Girma Gilano on 3/9/2023

People accept as something modern and will be happy

1 Codes:

- **Acceptability: Technology_care**

13 Quotations:

1:22 ¶ 33, revious services are not different but this is automated, evidenced, a… in interview analysis / 2:2 ¶ 10, I feel happy because it is modernized in KII / 2:5 ¶ 14, Am happy that finally we will have technology to support our client es… in KII / 2:12 ¶ 24, I think they will be happy. Everyone love technology whatever it is. E… in KII / 2:32 ¶ 50, People accept as something modern and will be happy in KII / 2:37 ¶ 56, People currently eager to have new technology so if no payment for the… in KII / 2:38 ¶ 57, I think it will not be possible after sometime to live without or work… in KII / 2:41 ¶ 62, Health education is necessary and our community is active for such new… in KII / 3:7 ¶ 6, Women always want something that allows better service so this can be… in Group discussion / 3:14 ¶ 8, They community as a whole may see this positively and acknowledge the… in Group discussion / 3:31 ¶ 17, It is interesting to use the technology at this level, so am happy. Mo… in Group discussion / 3:55 ¶ 40, ccept the technology as the part of easing their work concerns. Awaren… in Group discussion / 3:69 ¶ 56, Expectation of mother to get SMS to take service, and otherwise consid… in Group discussion

2:37 ¶ 56 in KII

Text quotation

Created by Girma Gilano on 3/9/2023

People currently eager to have new technology so if no payment for the technology no everyone will be happy to have this (mothers). It can also reduce excessive human power

1 Codes:

- **Acceptability: Technology_care**

13 Quotations:

1:22 ¶ 33, revious services are not different but this is automated, evidenced, a… in interview analysis / 2:2 ¶ 10, I feel happy because it is modernized in KII / 2:5 ¶ 14, Am happy that finally we will have technology to support our client es… in KII / 2:12 ¶ 24, I think they will be happy. Everyone love technology whatever it is. E… in KII / 2:32 ¶ 50, People accept as something modern and will be happy in KII / 2:37 ¶ 56, People currently eager to have new technology so if no payment for the… in KII / 2:38 ¶ 57, I think it will not be possible after sometime to live without or work… in KII / 2:41 ¶ 62, Health education is necessary and our community is active for such new… in KII / 3:7 ¶ 6, Women always want something that allows better service so this can be… in Group discussion / 3:14 ¶ 8, They community as a whole may see this positively and acknowledge the… in Group discussion / 3:31 ¶ 17, It is interesting

to use the technology at this level, so am happy. Mo… in Group discussion / 3:55 ¶ 40, ccept the technology as the part of easing their work concerns. Awaren… in Group discussion / 3:69 ¶ 56, Expectation of mother to get SMS to take service, and otherwise consid… in Group discussion

2:38 ¶ 57 in KII

Text quotation

Created by Girma Gilano on 3/9/2023

I think it will not be possible after sometime to live without or work without technology so will be happy to see

1 Codes:

- **Acceptability: Technology_care**

13 Quotations:

1:22 ¶ 33, revious services are not different but this is automated, evidenced, a… in interview analysis / 2:2 ¶ 10, I feel happy because it is modernized in KII / 2:5 ¶ 14, Am happy that finally we will have technology to support our client es… in KII / 2:12 ¶ 24, I think they will be happy. Everyone love technology whatever it is. E… in KII / 2:32 ¶ 50, People accept as something modern and will be happy in KII / 2:37 ¶ 56, People currently eager to have new technology so if no payment for the… in KII / 2:38 ¶ 57, I think it will not be possible after sometime to live without or work… in KII / 2:41 ¶ 62, Health education is necessary and our community is active for such new… in KII / 3:7 ¶ 6, Women always want something that allows better service so this can be… in Group discussion / 3:14 ¶ 8, They community as a whole may see this positively and acknowledge the… in Group discussion / 3:31 ¶ 17, It is interesting to use the technology at this level, so am happy. Mo… in Group discussion / 3:55 ¶ 40, ccept the technology as the part of easing their work concerns. Awaren… in Group discussion / 3:69 ¶ 56, Expectation of mother to get SMS to take service, and otherwise consid… in Group discussion

2:41 ¶ 62 in KII

Text quotation

Created by Girma Gilano on 3/9/2023

Health education is necessary and our community is active for such new technology based services

2 Codes:

- **Acceptability: support_professionals**

10 Quotations:

1:47 ¶ 54, If this is to support but not contradict with HEWs work it is importa… in interview analysis / 1:52 ¶ 58, but she may also have to come to health professional for further inves… in interview analysis / 2:13 ¶ 25, I think our institution workers will welcome this more than anything.… in KII / 2:16 ¶ 28, Health professionals' role should be high to make it accepted by the m… in KII / 2:17 ¶ 29, is is can easy our workers job, they will happy to accept as peacefull… in KII / 2:39 ¶ 58, We have previous requirements of following patients, so this strengthe… in KII / 2:41 ¶ 62, Health education is necessary and our community is active for such new… in KII / 2:44 ¶ 64, Making people understand the service objectives especially health prof… in KII / 3:31 ¶ 17, It is interesting to use the technology at this level, so am

happy. Mo… in Group discussion / 3:55 ¶ 40, ccept the technology as the part of easing their work concerns. Awaren… in Group discussion

- **Acceptability: Technology_care**

13 Quotations:

1:22 ¶ 33, revious services are not different but this is automated, evidenced, a… in interview analysis / 2:2 ¶ 10, I feel happy because it is modernized in KII / 2:5 ¶ 14, Am happy that finally we will have technology to support our client es… in KII / 2:12 ¶ 24, I think they will be happy. Everyone love technology whatever it is. E… in KII / 2:32 ¶ 50, People accept as something modern and will be happy in KII / 2:37 ¶ 56, People currently eager to have new technology so if no payment for the… in KII / 2:38 ¶ 57, I think it will not be possible after sometime to live without or work… in KII / 2:41 ¶ 62, Health education is necessary and our community is active for such new… in KII / 3:7 ¶ 6, Women always want something that allows better service so this can be… in Group discussion / 3:14 ¶ 8, They community as a whole may see this positively and acknowledge the… in Group discussion / 3:31 ¶ 17, It is interesting to use the technology at this level, so am happy. Mo… in Group discussion / 3:55 ¶ 40, ccept the technology as the part of easing their work concerns. Awaren… in Group discussion / 3:69 ¶ 56, Expectation of mother to get SMS to take service, and otherwise consid… in Group discussion

3:7 ¶ 6 in Group discussion

Text quotation

Created by Girma Gilano on 3/9/2023

Women always want something that allows better service so this can be important

2 Codes:

- **Acceptability: Fiting_current**

9 Quotations:

1:20 ¶ 32, It fits very well, but I think the process will take a long time to ad… in interview analysis / 1:27 ¶ 36, I think it can go with the previous service in interview analysis / 1:32 ¶ 38, There is no culture to my knowledge that can hinder the service in interview analysis / 1:33 ¶ 39, I do not think there is any opposing culture anywhere in interview analysis / 2:30 ¶ 46, No culture in opposite to this service in KII / 2:31 ¶ 47, Every culture is improving through technology and it is everywhere, so… in KII / 2:45 ¶ 65, I hope they will accept easily because this is the part of health comm… in KII / 3:7 ¶ 6, Women always want something that allows better service so this can be… in Group discussion / 3:14 ¶ 8, They community as a whole may see this positively and acknowledge the… in Group discussion

- **Acceptability: Technology_care**

13 Quotations:

1:22 ¶ 33, revious services are not different but this is automated, evidenced, a… in interview analysis / 2:2 ¶ 10, I feel happy because it is modernized in KII / 2:5 ¶ 14, Am happy that finally we will have technology to support our client es… in KII / 2:12 ¶ 24, I think they will be happy. Everyone love technology whatever it is. E… in KII / 2:32 ¶ 50, People accept as something modern and will be happy in KII / 2:37 ¶ 56, People currently eager to have new technology so if no payment for the… in KII / 2:38 ¶ 57, I think it will not be possible after sometime to live without or work… in KII / 2:41 ¶ 62, Health education is necessary and our community is active for such new… in KII / 3:7 ¶ 6, Women always want something that allows better service so this can be… in Group discussion / 3:14 ¶ 8, They community as a whole may see this positively and acknowledge the… in Group discussion / 3:31 ¶ 17, It is interesting

to use the technology at this level, so am happy. Mo… in Group discussion / 3:55 ¶ 40, ccept the technology as the part of easing their work concerns. Awaren… in Group discussion / 3:69 ¶ 56, Expectation of mother to get SMS to take service, and otherwise consid… in Group discussion

3:14 ¶ 8 in Group discussion

Text quotation

Created by Girma Gilano on 3/9/2023

They community as a whole may see this positively and acknowledge the real effort

2 Codes:

- **Acceptability: Fiting_current**

9 Quotations:

1:20 ¶ 32, It fits very well, but I think the process will take a long time to ad… in interview analysis / 1:27 ¶ 36, I think it can go with the previous service in interview analysis / 1:32 ¶ 38, There is no culture to my knowledge that can hinder the service in interview analysis / 1:33 ¶ 39, I do not think there is any opposing culture anywhere in interview analysis / 2:30 ¶ 46, No culture in opposite to this service in KII / 2:31 ¶ 47, Every culture is improving through technology and it is everywhere, so… in KII / 2:45 ¶ 65, I hope they will accept easily because this is the part of health comm… in KII / 3:7 ¶ 6, Women always want something that allows better service so this can be… in Group discussion / 3:14 ¶ 8, They community as a whole may see this positively and acknowledge the… in Group discussion

- **Acceptability: Technology_care**

13 Quotations:

1:22 ¶ 33, revious services are not different but this is automated, evidenced, a… in interview analysis / 2:2 ¶ 10, I feel happy because it is modernized in KII / 2:5 ¶ 14, Am happy that finally we will have technology to support our client es… in KII / 2:12 ¶ 24, I think they will be happy. Everyone love technology whatever it is. E… in KII / 2:32 ¶ 50, People accept as something modern and will be happy in KII / 2:37 ¶ 56, People currently eager to have new technology so if no payment for the… in KII / 2:38 ¶ 57, I think it will not be possible after sometime to live without or work… in KII / 2:41 ¶ 62, Health education is necessary and our community is active for such new… in KII / 3:7 ¶ 6, Women always want something that allows better service so this can be… in Group discussion / 3:14 ¶ 8, They community as a whole may see this positively and acknowledge the… in Group discussion / 3:31 ¶ 17, It is interesting to use the technology at this level, so am happy. Mo… in Group discussion / 3:55 ¶ 40, ccept the technology as the part of easing their work concerns. Awaren… in Group discussion / 3:69 ¶ 56, Expectation of mother to get SMS to take service, and otherwise consid… in Group discussion

3:31 ¶ 17 in Group discussion

Text quotation

Created by Girma Gilano on 3/9/2023

It is interesting to use the technology at this level, so am happy. Mothers usually fear whatever health professionals order and can be comply when they ordered to meet on phone for the next information.

2 Codes:

- **Acceptability: support_professionals**

10 Quotations:

1:47 ¶ 54, If this is to support but not contradict with HEWs work it is importa… in interview analysis / 1:52 ¶ 58, but she may also have to come to health professional for further inves… in interview analysis / 2:13 ¶ 25, I think our institution workers will welcome this more than anything.… in KII / 2:16 ¶ 28, Health professionals' role should be high to make it accepted by the m… in KII / 2:17 ¶ 29, is is can easy our workers job, they will happy to accept as peacefull… in KII / 2:39 ¶ 58, We have previous requirements of following patients, so this strengthe… in KII / 2:41 ¶ 62, Health education is necessary and our community is active for such new… in KII / 2:44 ¶ 64, Making people understand the service objectives especially health prof… in KII / 3:31 ¶ 17, It is interesting to use the technology at this level, so am happy. Mo… in Group discussion / 3:55 ¶ 40, ccept the technology as the part of easing their work concerns. Awaren… in Group discussion

- **Acceptability: Technology_care**

13 Quotations:

1:22 ¶ 33, revious services are not different but this is automated, evidenced, a… in interview analysis / 2:2 ¶ 10, I feel happy because it is modernized in KII / 2:5 ¶ 14, Am happy that finally we will have technology to support our client es… in KII / 2:12 ¶ 24, I think they will be happy. Everyone love technology whatever it is. E… in KII / 2:32 ¶ 50, People accept as something modern and will be happy in KII / 2:37 ¶ 56, People currently eager to have new technology so if no payment for the… in KII / 2:38 ¶ 57, I think it will not be possible after sometime to live without or work… in KII / 2:41 ¶ 62, Health education is necessary and our community is active for such new… in KII / 3:7 ¶ 6, Women always want something that allows better service so this can be… in Group discussion / 3:14 ¶ 8, They community as a whole may see this positively and acknowledge the… in Group discussion / 3:31 ¶ 17, It is interesting to use the technology at this level, so am happy. Mo… in Group discussion / 3:55 ¶ 40, ccept the technology as the part of easing their work concerns. Awaren… in Group discussion / 3:69 ¶ 56, Expectation of mother to get SMS to take service, and otherwise consid… in Group discussion

3:55 ¶ 40 in Group discussion

Text quotation

Created by Girma Gilano on 3/9/2023

ccept the technology as the part of easing their work concerns. Awareness creation among mothers and community is critical

2 Codes:

- **Acceptability: support_professionals**

10 Quotations:

1:47 ¶ 54, If this is to support but not contradict with HEWs work it is importa… in interview analysis / 1:52 ¶ 58, but she may also have to come to health professional for further inves… in interview analysis / 2:13 ¶ 25, I think our institution workers will welcome this more than anything.… in KII / 2:16 ¶ 28, Health professionals' role should be high to make it accepted by the m… in KII / 2:17 ¶ 29, is is can easy our workers job, they will happy to accept as peacefull… in KII / 2:39 ¶ 58, We have previous requirements of following patients, so this strengthe… in KII / 2:41 ¶ 62, Health education is necessary and our community is active for such new… in KII / 2:44 ¶ 64, Making people understand the service objectives especially health prof… in KII / 3:31 ¶ 17, It is interesting to use the technology at this level, so am

happy. Mo… in Group discussion / 3:55 ¶ 40, ccept the technology as the part of easing their work concerns. Awaren… in Group discussion

- **Acceptability: Technology_care**

13 Quotations:

1:22 ¶ 33, revious services are not different but this is automated, evidenced, a… in interview analysis / 2:2 ¶ 10, I feel happy because it is modernized in KII / 2:5 ¶ 14, Am happy that finally we will have technology to support our client es… in KII / 2:12 ¶ 24, I think they will be happy. Everyone love technology whatever it is. E… in KII / 2:32 ¶ 50, People accept as something modern and will be happy in KII / 2:37 ¶ 56, People currently eager to have new technology so if no payment for the… in KII / 2:38 ¶ 57, I think it will not be possible after sometime to live without or work… in KII / 2:41 ¶ 62, Health education is necessary and our community is active for such new… in KII / 3:7 ¶ 6, Women always want something that allows better service so this can be… in Group discussion / 3:14 ¶ 8, They community as a whole may see this positively and acknowledge the… in Group discussion / 3:31 ¶ 17, It is interesting to use the technology at this level, so am happy. Mo… in Group discussion / 3:55 ¶ 40, ccept the technology as the part of easing their work concerns. Awaren… in Group discussion / 3:69 ¶ 56, Expectation of mother to get SMS to take service, and otherwise consid… in Group discussion

3:69 ¶ 56 in Group discussion

Text quotation

Created by Girma Gilano on 3/9/2023

Expectation of mother to get SMS to take service, and otherwise consideration of no risk

1 Codes:

- **Acceptability: Technology_care**

13 Quotations:

1:22 ¶ 33, revious services are not different but this is automated, evidenced, a… in interview analysis / 2:2 ¶ 10, I feel happy because it is modernized in KII / 2:5 ¶ 14, Am happy that finally we will have technology to support our client es… in KII / 2:12 ¶ 24, I think they will be happy. Everyone love technology whatever it is. E… in KII / 2:32 ¶ 50, People accept as something modern and will be happy in KII / 2:37 ¶ 56, People currently eager to have new technology so if no payment for the… in KII / 2:38 ¶ 57, I think it will not be possible after sometime to live without or work… in KII / 2:41 ¶ 62, Health education is necessary and our community is active for such new… in KII / 3:7 ¶ 6, Women always want something that allows better service so this can be… in Group discussion / 3:14 ¶ 8, They community as a whole may see this positively and acknowledge the… in Group discussion / 3:31 ¶ 17, It is interesting to use the technology at this level, so am happy. Mo… in Group discussion / 3:55 ¶ 40, ccept the technology as the part of easing their work concerns. Awaren… in Group discussion / 3:69 ¶ 56, Expectation of mother to get SMS to take service, and otherwise consid… in Group discussion

---

● Awareness creation: community_awarness

Created by Girma Gilano on 3/12/2023

8 Quotations:

1:1 ¶ 16 in interview analysis

Text quotation

Created by Girma Gilano on 3/8/2023

If one mother served all will need to take that will aslo motivate households to prepare mobile for every mother get pregnant

2 Codes:

- **Awareness creation: community_awarness**

8 Quotations:

1:1 ¶ 16, If one mother served all will need to take that will aslo motivate hou… in interview analysis / 1:21 ¶ 32, is is because of the need for familiarization or awareness creation bo… in interview analysis / 2:23 ¶ 36, Health development community members are the chance to convince mother… in KII / 2:34 ¶ 53, Encourage women education most importantly in the whole society. Use o… in KII / 3:21 ¶ 12, There may be a way perhaps to change the current ignorant behavior of… in Group discussion / 3:40 ¶ 24, sing mHealth consultation, providing care, or treating patient or clie… in Group discussion / 3:45 ¶ 28, After mothers forum or community mobilization our service our work may… in Group discussion / 3:56 ¶ 41, wareness in the community and improve works in those area according to… in Group discussion

- **Awareness creation: women_awarness**

19 Quotations:

1:1 ¶ 16, If one mother served all will need to take that will aslo motivate hou… in interview analysis / 1:67 ¶ 69, After mothers understand the service they will use and since they have… in interview analysis / 1:69 ¶ 70, onsultation, treatment follow up, other health education can be given… in interview analysis / 1:71 ¶ 73, I do not think there will a problem because we have to teach train and… in interview analysis / 1:73 ¶ 74, e can also be aware and work on behavioral change for not accepting mo… in interview analysis / 1:75 ¶ 76, I don’t think it will have any problem because when we tell mother “it… in interview analysis / 1:101 ¶ 100, Throughout mothers may need support. We can help with understanding. I… in interview analysis / 1:105 ¶ 105, We have to teach mothers how to use mobile, what is mHealth, how to op… in interview analysis / 2:14 ¶ 26, For mother it may need social and BCC to get it welcomed completely in KII / 2:23 ¶ 36, Health development community members are the chance to convince mother… in KII / 2:34 ¶ 53, Encourage women education most importantly in the whole society. Use o… in KII / 3:16 ¶ 10, Whatever the mother say either for refusal I think we can convince the… in Group discussion / 3:17 ¶ 12, May be this mobile service can be accompanied with behavioral change e… in Group discussion / 3:21 ¶ 12, There may be a way perhaps to change the current ignorant behavior of… in Group discussion / 3:26 ¶ 14, They way of following success and promotion should be continuous. Moth… in Group discussion / 3:32 ¶ 19, This will create an option to counsel and follow up mothers are not co… in Group discussion / 3:39 ¶ 24, Consultation is what we usually do when circumstances allow us to do in Group discussion / 3:40 ¶ 24, sing mHealth consultation, providing care, or treating patient or clie… in Group discussion / 3:64 ¶ 51, Reading ability. Many SMS sent by Ethio-tele. every day, so this need… in Group discussion

1:21 ¶ 32 in interview analysis

Text quotation

Created by Girma Gilano on 3/8/2023

is is because of the need for familiarization or awareness creation both in the community and among health professionals, which will take some time

2 Codes:

- **Awareness creation: community_awarness**

8 Quotations:

1:1 ¶ 16, If one mother served all will need to take that will aslo motivate hou… in interview analysis / 1:21 ¶ 32, is is because of the need for familiarization or awareness creation bo… in interview analysis / 2:23 ¶ 36, Health development community members are the chance to convince mother… in KII / 2:34 ¶ 53, Encourage women education most importantly in the whole society. Use o… in KII / 3:21 ¶ 12, There may be a way perhaps to change the current ignorant behavior of… in Group discussion / 3:40 ¶ 24, sing mHealth consultation, providing care, or treating patient or clie… in Group discussion / 3:45 ¶ 28, After mothers forum or community mobilization our service our work may… in Group discussion / 3:56 ¶ 41, wareness in the community and improve works in those area according to… in Group discussion

- **Awareness creation: provider_awarness**

4 Quotations:

1:21 ¶ 32, is is because of the need for familiarization or awareness creation bo… in interview analysis / 1:73 ¶ 74, e can also be aware and work on behavioral change for not accepting mo… in interview analysis / 2:20 ¶ 33, In Health institution awareness training for health profession, increa… in KII / 3:26 ¶ 14, They way of following success and promotion should be continuous. Moth… in Group discussion

2:23 ¶ 36 in KII

Text quotation

Created by Girma Gilano on 3/9/2023

Health development community members are the chance to convince mother and monitor. Currently due to the sanction we are suffering a lot, but when everything become alright, I think this the best thing our community deserves.

2 Codes:

- **Awareness creation: community_awarness**

8 Quotations:

1:1 ¶ 16, If one mother served all will need to take that will aslo motivate hou… in interview analysis / 1:21 ¶ 32, is is because of the need for familiarization or awareness creation bo… in interview analysis / 2:23 ¶ 36, Health development community members are the chance to convince mother… in KII / 2:34 ¶ 53, Encourage women education most importantly in the whole society. Use o… in KII / 3:21 ¶ 12, There may be a way perhaps to change the current ignorant behavior of… in Group discussion / 3:40 ¶ 24, sing mHealth consultation, providing care, or treating patient or clie… in Group discussion / 3:45 ¶ 28, After mothers forum or community mobilization our service our work may… in Group discussion / 3:56 ¶ 41, wareness in the community and improve works in those area according to… in Group discussion

- **Awareness creation: women_awarness**

19 Quotations:

1:1 ¶ 16, If one mother served all will need to take that will aslo motivate hou… in interview analysis / 1:67 ¶ 69, After mothers understand the service they will use and since they have… in interview analysis / 1:69 ¶ 70, onsultation, treatment follow up, other health education can be given… in interview analysis / 1:71 ¶ 73, I do not think there will a problem because we have to teach train and… in interview analysis / 1:73 ¶ 74, e can also be aware and work on behavioral change for not accepting mo… in interview analysis / 1:75 ¶ 76, I don't think it will have any problem because when we tell mother "it… in interview analysis / 1:101 ¶ 100, Throughout mothers may need support. We can help with understanding. I… in interview analysis / 1:105 ¶ 105, We have to teach mothers how to use mobile, what is mHealth, how to op… in interview analysis / 2:14 ¶ 26, For mother it may need social and BCC to get it welcomed completely in KII / 2:23 ¶ 36, Health development community members are the chance to convince mother… in KII / 2:34 ¶ 53, Encourage women education most importantly in the whole society. Use o… in KII / 3:16 ¶ 10, Whatever the mother say either for refusal I think we can convince the… in Group discussion / 3:17 ¶ 12, May be this mobile service can be accompanied with behavioral change e… in Group discussion / 3:21 ¶ 12, There may be a way perhaps to change the current ignorant behavior of… in Group discussion / 3:26 ¶ 14, They way of following success and promotion should be continuous. Moth… in Group discussion / 3:32 ¶ 19, This will create an option to counsel and follow up mothers are not co… in Group discussion / 3:39 ¶ 24, Consultation is what we usually do when circumstances allow us to do in Group discussion / 3:40 ¶ 24, sing mHealth consultation, providing care, or treating patient or clie… in Group discussion / 3:64 ¶ 51, Reading ability. Many SMS sent by Ethio-tele. every day, so this need… in Group discussion

2:34 ¶ 53 in KII

Text quotation

Created by Girma Gilano on 3/9/2023

Encourage women education most importantly in the whole society. Use of education as a tool to have good pregnancy.

2 Codes:

- **Awareness creation: community_awarness**

8 Quotations:

1:1 ¶ 16, If one mother served all will need to take that will aslo motivate hou… in interview analysis / 1:21 ¶ 32, is is because of the need for familiarization or awareness creation bo… in interview analysis / 2:23 ¶ 36, Health development community members are the chance to convince mother… in KII / 2:34 ¶ 53, Encourage women education most importantly in the whole society. Use o… in KII / 3:21 ¶ 12, There may be a way perhaps to change the current ignorant behavior of… in Group discussion / 3:40 ¶ 24, sing mHealth consultation, providing care, or treating patient or clie… in Group discussion / 3:45 ¶ 28, After mothers forum or community mobilization our service our work may… in Group discussion / 3:56 ¶ 41, wareness in the community and improve works in those area according to… in Group discussion

- **Awareness creation: women_awarness**

19 Quotations:

1:1 ¶ 16, If one mother served all will need to take that will aslo motivate hou… in interview analysis / 1:67 ¶ 69, After mothers understand the service they will use and since they have… in interview analysis / 1:69 ¶ 70, onsultation, treatment follow up, other health education can be given… in interview analysis / 1:71 ¶ 73, I do not think there will a problem because we have to teach train and… in interview analysis / 1:73 ¶ 74, e can also be aware and work on behavioral change for not accepting mo… in interview analysis / 1:75 ¶ 76, I don't think it will have any problem because when we tell mother "it… in interview

analysis / 1:101 ¶ 100, Throughout mothers may need support. We can help with understanding. I… in interview analysis / 1:105 ¶ 105, We have to teach mothers how to use mobile, what is mHealth, how to op… in interview analysis / 2:14 ¶ 26, For mother it may need social and BCC to get it welcomed completely in KII / 2:23 ¶ 36, Health development community members are the chance to convince mother… in KII / 2:34 ¶ 53, Encourage women education most importantly in the whole society. Use o… in KII / 3:16 ¶ 10, Whatever the mother say either for refusal I think we can convince the… in Group discussion / 3:17 ¶ 12, May be this mobile service can be accompanied with behavioral change e… in Group discussion / 3:21 ¶ 12, There may be a way perhaps to change the current ignorant behavior of… in Group discussion / 3:26 ¶ 14, They way of following success and promotion should be continuous. Moth… in Group discussion / 3:32 ¶ 19, This will create an option to counsel and follow up mothers are not co… in Group discussion / 3:39 ¶ 24, Consultation is what we usually do when circumstances allow us to do in Group discussion / 3:40 ¶ 24, sing mHealth consultation, providing care, or treating patient or clie… in Group discussion / 3:64 ¶ 51, Reading ability. Many SMS sent by Ethio-tele. every day, so this need… in Group discussion

3:21 ¶ 12 in Group discussion

Text quotation

Created by Girma Gilano on 3/9/2023

There may be a way perhaps to change the current ignorant behavior of mothers

2 Codes:

- **Awareness creation: community_awarness**

8 Quotations:

1:1 ¶ 16, If one mother served all will need to take that will aslo motivate hou… in interview analysis / 1:21 ¶ 32, is is because of the need for familiarization or awareness creation bo… in interview analysis / 2:23 ¶ 36, Health development community members are the chance to convince mother… in KII / 2:34 ¶ 53, Encourage women education most importantly in the whole society. Use o… in KII / 3:21 ¶ 12, There may be a way perhaps to change the current ignorant behavior of… in Group discussion / 3:40 ¶ 24, sing mHealth consultation, providing care, or treating patient or clie… in Group discussion / 3:45 ¶ 28, After mothers forum or community mobilization our service our work may… in Group discussion / 3:56 ¶ 41, wareness in the community and improve works in those area according to… in Group discussion

- **Awareness creation: women_awarness**

19 Quotations:

1:1 ¶ 16, If one mother served all will need to take that will aslo motivate hou… in interview analysis / 1:67 ¶ 69, After mothers understand the service they will use and since they have… in interview analysis / 1:69 ¶ 70, onsultation, treatment follow up, other health education can be given… in interview analysis / 1:71 ¶ 73, I do not think there will a problem because we have to teach train and… in interview analysis / 1:73 ¶ 74, e can also be aware and work on behavioral change for not accepting mo… in interview analysis / 1:75 ¶ 76, I don’t think it will have any problem because when we tell mother “it… in interview analysis / 1:101 ¶ 100, Throughout mothers may need support. We can help with understanding. I… in interview analysis / 1:105 ¶ 105, We have to teach mothers how to use mobile, what is mHealth, how to op… in interview analysis / 2:14 ¶ 26, For mother it may need social and BCC to get it welcomed completely in KII / 2:23 ¶ 36, Health development community members are the chance to convince mother… in KII / 2:34 ¶ 53, Encourage women education most importantly in the whole society. Use o… in KII / 3:16 ¶ 10, Whatever the mother say either for refusal I think we can convince the… in Group discussion / 3:17 ¶ 12, May be this mobile service can be accompanied with behavioral change e… in Group discussion / 3:21 ¶ 12, There may be a way perhaps to change the current ignorant behavior of… in Group discussion / 3:26 ¶ 14, They way of following success and promotion should be continuous.

Moth… in Group discussion / 3:32 ¶ 19, This will create an option to counsel and follow up mothers are not co… in Group discussion / 3:39 ¶ 24, Consultation is what we usually do when circumstances allow us to do in Group discussion / 3:40 ¶ 24, sing mHealth consultation, providing care, or treating patient or clie… in Group discussion / 3:64 ¶ 51, Reading ability. Many SMS sent by Ethio-tele. every day, so this need… in Group discussion

3:40 ¶ 24 in Group discussion

Text quotation

Created by Girma Gilano on 3/9/2023

sing mHealth consultation, providing care, or treating patient or client look special because we can do whatever when we can or at any time.

3 Codes:

- **Awareness creation: community_awarness**

8 Quotations:

1:1 ¶ 16, If one mother served all will need to take that will aslo motivate hou… in interview analysis / 1:21 ¶ 32, is is because of the need for familiarization or awareness creation bo… in interview analysis / 2:23 ¶ 36, Health development community members are the chance to convince mother… in KII / 2:34 ¶ 53, Encourage women education most importantly in the whole society. Use o… in KII / 3:21 ¶ 12, There may be a way perhaps to change the current ignorant behavior of… in Group discussion / 3:40 ¶ 24, sing mHealth consultation, providing care, or treating patient or clie… in Group discussion / 3:45 ¶ 28, After mothers forum or community mobilization our service our work may… in Group discussion / 3:56 ¶ 41, wareness in the community and improve works in those area according to… in Group discussion

- **Awareness creation: family_awareness**

4 Quotations:

1:30 ¶ 37, I think if her husband is aware of the service, there will be no probl… in interview analysis / 3:40 ¶ 24, sing mHealth consultation, providing care, or treating patient or clie… in Group discussion / 3:45 ¶ 28, After mothers forum or community mobilization our service our work may… in Group discussion / 3:64 ¶ 51, Reading ability. Many SMS sent by Ethio-tele. every day, so this need… in Group discussion

- **Awareness creation: women_awarness**

19 Quotations:

1:1 ¶ 16, If one mother served all will need to take that will aslo motivate hou… in interview analysis / 1:67 ¶ 69, After mothers understand the service they will use and since they have… in interview analysis / 1:69 ¶ 70, onsultation, treatment follow up, other health education can be given… in interview analysis / 1:71 ¶ 73, I do not think there will a problem because we have to teach train and… in interview analysis / 1:73 ¶ 74, e can also be aware and work on behavioral change for not accepting mo… in interview analysis / 1:75 ¶ 76, I don’t think it will have any problem because when we tell mother “it… in interview analysis / 1:101 ¶ 100, Throughout mothers may need support. We can help with understanding. I… in interview analysis / 1:105 ¶ 105, We have to teach mothers how to use mobile, what is mHealth, how to op… in interview analysis / 2:14 ¶ 26, For mother it may need social and BCC to get it welcomed completely in KII / 2:23 ¶ 36, Health development community members are the chance to convince mother… in KII / 2:34 ¶ 53, Encourage women education most importantly in the whole society. Use o… in KII / 3:16 ¶ 10, Whatever the mother say either for refusal I think we can convince the… in Group

discussion / 3:17 ¶ 12, May be this mobile service can be accompanied with behavioral change e… in Group discussion / 3:21 ¶ 12, There may be a way perhaps to change the current ignorant behavior of… in Group discussion / 3:26 ¶ 14, They way of following success and promotion should be continuous. Moth… in Group discussion / 3:32 ¶ 19, This will create an option to counsel and follow up mothers are not co… in Group discussion / 3:39 ¶ 24, Consultation is what we usually do when circumstances allow us to do in Group discussion / 3:40 ¶ 24, sing mHealth consultation, providing care, or treating patient or clie… in Group discussion / 3:64 ¶ 51, Reading ability. Many SMS sent by Ethio-tele. every day, so this need… in Group discussion

3:45 ¶ 28 in Group discussion

Text quotation

Created by Girma Gilano on 3/9/2023

After mothers forum or community mobilization our service our work may be easy through mHealth

2 Codes:

- **Awareness creation: community_awarness**

8 Quotations:

1:1 ¶ 16, If one mother served all will need to take that will aslo motivate hou… in interview analysis / 1:21 ¶ 32, is is because of the need for familiarization or awareness creation bo… in interview analysis / 2:23 ¶ 36, Health development community members are the chance to convince mother… in KII / 2:34 ¶ 53, Encourage women education most importantly in the whole society. Use o… in KII / 3:21 ¶ 12, There may be a way perhaps to change the current ignorant behavior of… in Group discussion / 3:40 ¶ 24, sing mHealth consultation, providing care, or treating patient or clie… in Group discussion / 3:45 ¶ 28, After mothers forum or community mobilization our service our work may… in Group discussion / 3:56 ¶ 41, wareness in the community and improve works in those area according to… in Group discussion

- **Awareness creation: family_awareness**

4 Quotations:

1:30 ¶ 37, I think if her husband is aware of the service, there will be no probl… in interview analysis / 3:40 ¶ 24, sing mHealth consultation, providing care, or treating patient or clie… in Group discussion / 3:45 ¶ 28, After mothers forum or community mobilization our service our work may… in Group discussion / 3:64 ¶ 51, Reading ability. Many SMS sent by Ethio-tele. every day, so this need… in Group discussion

3:56 ¶ 41 in Group discussion

Text quotation

Created by Girma Gilano on 3/9/2023

wareness in the community and improve works in those area according to the new technology

1 Codes:

- **Awareness creation: community_awarness**

8 Quotations:

1:1 ¶ 16, If one mother served all will need to take that will aslo motivate hou… in interview analysis / 1:21 ¶ 32, is is because of the need for familiarization or awareness creation bo… in interview analysis / 2:23 ¶ 36, Health development community members are the chance to convince mother… in KII / 2:34 ¶ 53, Encourage women education most importantly in the whole society. Use o… in KII / 3:21 ¶ 12, There may be a way perhaps to change the current ignorant behavior of… in Group discussion / 3:40 ¶ 24, sing mHealth consultation, providing care, or treating patient or clie… in Group discussion / 3:45 ¶ 28, After mothers forum or community mobilization our service our work may… in Group discussion / 3:56 ¶ 41, wareness in the community and improve works in those area according to… in Group discussion

---

Awareness creation: family_awareness

Created by Girma Gilano on 3/12/2023

4 Quotations:

1:30 ¶ 37 in interview analysis

Text quotation

Created by Girma Gilano on 3/8/2023

I think if her husband is aware of the service, there will be no problem

1 Codes:

- **Awareness creation: family_awareness**

4 Quotations:

1:30 ¶ 37, I think if her husband is aware of the service, there will be no probl… in interview analysis / 3:40 ¶ 24, sing mHealth consultation, providing care, or treating patient or clie… in Group discussion / 3:45 ¶ 28, After mothers forum or community mobilization our service our work may… in Group discussion / 3:64 ¶ 51, Reading ability. Many SMS sent by Ethio-tele. every day, so this need… in Group discussion

3:40 ¶ 24 in Group discussion

Text quotation

Created by Girma Gilano on 3/9/2023

sing mHealth consultation, providing care, or treating patient or client look special because we can do whatever when we can or at any time.

3 Codes:

- **Awareness creation: community_awarness**

8 Quotations:

1:1 ¶ 16, If one mother served all will need to take that will aslo motivate hou… in interview analysis / 1:21 ¶ 32, is is because of the need for familiarization or awareness creation bo… in interview analysis / 2:23 ¶ 36, Health development community members are the chance to convince mother… in KII / 2:34 ¶ 53, Encourage women education most importantly in the whole society. Use o… in KII / 3:21 ¶ 12, There may be a way perhaps to change the current ignorant behavior of… in Group discussion / 3:40 ¶ 24, sing mHealth consultation, providing care, or treating patient or clie… in Group discussion / 3:45 ¶ 28, After mothers forum or community mobilization our service our work may… in Group discussion / 3:56 ¶ 41, wareness in the community and improve works in those area according to… in Group discussion

- **Awareness creation: family_awareness**

4 Quotations:

1:30 ¶ 37, I think if her husband is aware of the service, there will be no probl… in interview analysis / 3:40 ¶ 24, sing mHealth consultation, providing care, or treating patient or clie… in Group discussion / 3:45 ¶ 28, After mothers forum or community mobilization our service our work may… in Group discussion / 3:64 ¶ 51, Reading ability. Many SMS sent by Ethio-tele. every day, so this need… in Group discussion

- **Awareness creation: women_awarness**

19 Quotations:

1:1 ¶ 16, If one mother served all will need to take that will aslo motivate hou… in interview analysis / 1:67 ¶ 69, After mothers understand the service they will use and since they have… in interview analysis / 1:69 ¶ 70, onsultation, treatment follow up, other health education can be given… in interview analysis / 1:71 ¶ 73, I do not think there will a problem because we have to teach train and… in interview analysis / 1:73 ¶ 74, e can also be aware and work on behavioral change for not accepting mo… in interview analysis / 1:75 ¶ 76, I don't think it will have any problem because when we tell mother "it… in interview analysis / 1:101 ¶ 100, Throughout mothers may need support. We can help with understanding. I… in interview analysis / 1:105 ¶ 105, We have to teach mothers how to use mobile, what is mHealth, how to op… in interview analysis / 2:14 ¶ 26, For mother it may need social and BCC to get it welcomed completely in KII / 2:23 ¶ 36, Health development community members are the chance to convince mother… in KII / 2:34 ¶ 53, Encourage women education most importantly in the whole society. Use o… in KII / 3:16 ¶ 10, Whatever the mother say either for refusal I think we can convince the… in Group discussion / 3:17 ¶ 12, May be this mobile service can be accompanied with behavioral change e… in Group discussion / 3:21 ¶ 12, There may be a way perhaps to change the current ignorant behavior of… in Group discussion / 3:26 ¶ 14, They way of following success and promotion should be continuous. Moth… in Group discussion / 3:32 ¶ 19, This will create an option to counsel and follow up mothers are not co… in Group discussion / 3:39 ¶ 24, Consultation is what we usually do when circumstances allow us to do in Group discussion / 3:40 ¶ 24, sing mHealth consultation, providing care, or treating patient or clie… in Group discussion / 3:64 ¶ 51, Reading ability. Many SMS sent by Ethio-tele. every day, so this need… in Group discussion

3:45 ¶ 28 in Group discussion

Text quotation

Created by Girma Gilano on 3/9/2023

After mothers forum or community mobilization our service our work may be easy through mHealth

2 Codes:

- **Awareness creation: community_awarness**

8 Quotations:

1:1 ¶ 16, If one mother served all will need to take that will aslo motivate hou… in interview analysis / 1:21 ¶ 32, is is because of the need for familiarization or awareness creation bo… in interview analysis / 2:23 ¶ 36, Health development community members are the chance to convince mother… in KII / 2:34 ¶ 53, Encourage women education most importantly in the whole society. Use o… in KII / 3:21 ¶ 12, There may be a way perhaps to change the current ignorant behavior of… in Group discussion / 3:40 ¶ 24, sing mHealth consultation, providing care, or treating patient or clie… in Group discussion / 3:45 ¶ 28, After mothers forum or community mobilization our service our work may… in Group discussion / 3:56 ¶ 41, wareness in the community and improve works in those area according to… in Group discussion

- **Awareness creation: family_awareness**

4 Quotations:

1:30 ¶ 37, I think if her husband is aware of the service, there will be no probl… in interview analysis / 3:40 ¶ 24, sing mHealth consultation, providing care, or treating patient or clie… in Group discussion / 3:45 ¶ 28, After mothers forum or community mobilization our service our work may… in Group discussion / 3:64 ¶ 51, Reading ability. Many SMS sent by Ethio-tele. every day, so this need… in Group discussion

3:64 ¶ 51 in Group discussion

Text quotation

Created by Girma Gilano on 3/9/2023

Reading ability. Many SMS sent by Ethio-tele. every day, so this need little awareness

2 Codes:

- **Awareness creation: family_awareness**

4 Quotations:

1:30 ¶ 37, I think if her husband is aware of the service, there will be no probl… in interview analysis / 3:40 ¶ 24, sing mHealth consultation, providing care, or treating patient or clie… in Group discussion / 3:45 ¶ 28, After mothers forum or community mobilization our service our work may… in Group discussion / 3:64 ¶ 51, Reading ability. Many SMS sent by Ethio-tele. every day, so this need… in Group discussion

- **Awareness creation: women_awarness**

19 Quotations:

1:1 ¶ 16, If one mother served all will need to take that will aslo motivate hou… in interview analysis / 1:67 ¶ 69, After mothers understand the service they will use and since they have… in interview analysis / 1:69 ¶ 70, onsultation, treatment follow up, other health education can be given… in interview analysis / 1:71 ¶ 73, I do not think there will a problem because we have to teach train and… in interview analysis / 1:73 ¶ 74, e can also be aware and work on behavioral change for not accepting mo… in interview

analysis / 1:75 ¶ 76, I don't think it will have any problem because when we tell mother "it… in interview analysis / 1:101 ¶ 100, Throughout mothers may need support. We can help with understanding. I… in interview analysis / 1:105 ¶ 105, We have to teach mothers how to use mobile, what is mHealth, how to op… in interview analysis / 2:14 ¶ 26, For mother it may need social and BCC to get it welcomed completely in KII / 2:23 ¶ 36, Health development community members are the chance to convince mother… in KII / 2:34 ¶ 53, Encourage women education most importantly in the whole society. Use o… in KII / 3:16 ¶ 10, Whatever the mother say either for refusal I think we can convince the… in Group discussion / 3:17 ¶ 12, May be this mobile service can be accompanied with behavioral change e… in Group discussion / 3:21 ¶ 12, There may be a way perhaps to change the current ignorant behavior of… in Group discussion / 3:26 ¶ 14, They way of following success and promotion should be continuous. Moth… in Group discussion / 3:32 ¶ 19, This will create an option to counsel and follow up mothers are not co… in Group discussion / 3:39 ¶ 24, Consultation is what we usually do when circumstances allow us to do in Group discussion / 3:40 ¶ 24, sing mHealth consultation, providing care, or treating patient or clie… in Group discussion / 3:64 ¶ 51, Reading ability. Many SMS sent by Ethio-tele. every day, so this need… in Group discussion

---

● Awareness creation: provider_awarness

Created by Girma Gilano on 3/12/2023

4 Quotations:

1:21 ¶ 32 in interview analysis

Text quotation

Created by Girma Gilano on 3/8/2023

is is because of the need for familiarization or awareness creation both in the community and among health professionals, which will take some time

2 Codes:

- **Awareness creation: community_awarness**

  8 Quotations:

  1:1 ¶ 16, If one mother served all will need to take that will aslo motivate hou… in interview analysis / 1:21 ¶ 32, is is because of the need for familiarization or awareness creation bo… in interview analysis / 2:23 ¶ 36, Health development community members are the chance to convince mother… in KII / 2:34 ¶ 53, Encourage women education most importantly in the whole society. Use o… in KII / 3:21 ¶ 12, There may be a way perhaps to change the current ignorant behavior of… in Group discussion / 3:40 ¶ 24, sing mHealth consultation, providing care, or treating patient or clie… in Group discussion / 3:45 ¶ 28, After mothers forum or community mobilization our service our work may… in Group discussion / 3:56 ¶ 41, wareness in the community and improve works in those area according to… in Group discussion

- **Awareness creation: provider_awarness**

  4 Quotations:

  1:21 ¶ 32, is is because of the need for familiarization or awareness creation bo… in interview analysis / 1:73 ¶ 74, e can also be aware and work on behavioral change for not accepting mo… in interview

analysis / 2:20 ¶ 33, In Health institution awareness training for health profession, increa… in KII / 3:26 ¶ 14, They way of following success and promotion should be continuous. Moth… in Group discussion

1:73 ¶ 74 in interview analysis

Text quotation

Created by Girma Gilano on 3/8/2023

e can also be aware and work on behavioral change for not accepting mothers

2 Codes:

- **Awareness creation: provider_awarness**

4 Quotations:

1:21 ¶ 32, is is because of the need for familiarization or awareness creation bo… in interview analysis / 1:73 ¶ 74, e can also be aware and work on behavioral change for not accepting mo… in interview analysis / 2:20 ¶ 33, In Health institution awareness training for health profession, increa… in KII / 3:26 ¶ 14, They way of following success and promotion should be continuous. Moth… in Group discussion

- **Awareness creation: women_awarness**

19 Quotations:

1:1 ¶ 16, If one mother served all will need to take that will aslo motivate hou… in interview analysis / 1:67 ¶ 69, After mothers understand the service they will use and since they have… in interview analysis / 1:69 ¶ 70, onsultation, treatment follow up, other health education can be given… in interview analysis / 1:71 ¶ 73, I do not think there will a problem because we have to teach train and… in interview analysis / 1:73 ¶ 74, e can also be aware and work on behavioral change for not accepting mo… in interview analysis / 1:75 ¶ 76, I don't think it will have any problem because when we tell mother "it… in interview analysis / 1:101 ¶ 100, Throughout mothers may need support. We can help with understanding. I… in interview analysis / 1:105 ¶ 105, We have to teach mothers how to use mobile, what is mHealth, how to op… in interview analysis / 2:14 ¶ 26, For mother it may need social and BCC to get it welcomed completely in KII / 2:23 ¶ 36, Health development community members are the chance to convince mother… in KII / 2:34 ¶ 53, Encourage women education most importantly in the whole society. Use o… in KII / 3:16 ¶ 10, Whatever the mother say either for refusal I think we can convince the… in Group discussion / 3:17 ¶ 12, May be this mobile service can be accompanied with behavioral change e… in Group discussion / 3:21 ¶ 12, There may be a way perhaps to change the current ignorant behavior of… in Group discussion / 3:26 ¶ 14, They way of following success and promotion should be continuous. Moth… in Group discussion / 3:32 ¶ 19, This will create an option to counsel and follow up mothers are not co… in Group discussion / 3:39 ¶ 24, Consultation is what we usually do when circumstances allow us to do in Group discussion / 3:40 ¶ 24, sing mHealth consultation, providing care, or treating patient or clie… in Group discussion / 3:64 ¶ 51, Reading ability. Many SMS sent by Ethio-tele. every day, so this need… in Group discussion

2:20 ¶ 33 in KII

Text quotation

Created by Girma Gilano on 3/9/2023

In Health institution awareness training for health profession, increasing positive feedback mechanism

1 Codes:

- **Awareness creation: provider_awarness**

4 Quotations:

1:21 ¶ 32, is is because of the need for familiarization or awareness creation bo… in interview analysis / 1:73 ¶ 74, e can also be aware and work on behavioral change for not accepting mo… in interview analysis / 2:20 ¶ 33, In Health institution awareness training for health profession, increa… in KII / 3:26 ¶ 14, They way of following success and promotion should be continuous. Moth… in Group discussion

3:26 ¶ 14 in Group discussion

Text quotation

Created by Girma Gilano on 3/9/2023

They way of following success and promotion should be continuous. Mother can be promote even to use and buy mobile phone to be pregnant and this can be common practice for women in the community. They may even ask a woman why she pregnant before having a phone

2 Codes:

- **Awareness creation: provider_awarness**

4 Quotations:

1:21 ¶ 32, is is because of the need for familiarization or awareness creation bo… in interview analysis / 1:73 ¶ 74, e can also be aware and work on behavioral change for not accepting mo… in interview analysis / 2:20 ¶ 33, In Health institution awareness training for health profession, increa… in KII / 3:26 ¶ 14, They way of following success and promotion should be continuous. Moth… in Group discussion

- **Awareness creation: women_awarness**

19 Quotations:

1:1 ¶ 16, If one mother served all will need to take that will aslo motivate hou… in interview analysis / 1:67 ¶ 69, After mothers understand the service they will use and since they have… in interview analysis / 1:69 ¶ 70, onsultation, treatment follow up, other health education can be given… in interview analysis / 1:71 ¶ 73, I do not think there will a problem because we have to teach train and… in interview analysis / 1:73 ¶ 74, e can also be aware and work on behavioral change for not accepting mo… in interview analysis / 1:75 ¶ 76, I don’t think it will have any problem because when we tell mother “it… in interview analysis / 1:101 ¶ 100, Throughout mothers may need support. We can help with understanding. I… in interview analysis / 1:105 ¶ 105, We have to teach mothers how to use mobile, what is mHealth, how to op… in interview analysis / 2:14 ¶ 26, For mother it may need social and BCC to get it welcomed completely in KII / 2:23 ¶ 36, Health development community members are the chance to convince mother… in KII / 2:34 ¶ 53, Encourage women education most importantly in the whole society. Use o… in KII / 3:16 ¶ 10, Whatever the mother say either for refusal I think we can convince the… in Group discussion / 3:17 ¶ 12, May be this mobile service can be accompanied with behavioral change e… in Group discussion / 3:21 ¶ 12, There may be a way perhaps to change the current ignorant behavior of… in Group discussion / 3:26 ¶ 14, They way of following success and promotion should be continuous. Moth… in Group discussion / 3:32 ¶ 19, This will create an option to counsel and follow up mothers are not co… in Group discussion / 3:39 ¶ 24, Consultation is what we usually do when circumstances allow us to do in Group discussion / 3:40 ¶ 24, sing mHealth consultation, providing care, or treating patient or

clie… in Group discussion / 3:64 ¶ 51, Reading ability. Many SMS sent by Ethio-tele. every day, so this need… in Group discussion

---

Awareness creation: women_awarness

Created by Girma Gilano on 3/12/2023

19 Quotations:

1:1 ¶ 16 in interview analysis

Text quotation

Created by Girma Gilano on 3/8/2023

If one mother served all will need to take that will aslo motivate households to prepare mobile for every mother get pregnant

2 Codes:

- **Awareness creation: community_awarness**

8 Quotations:

1:1 ¶ 16, If one mother served all will need to take that will aslo motivate hou… in interview analysis / 1:21 ¶ 32, is is because of the need for familiarization or awareness creation bo… in interview analysis / 2:23 ¶ 36, Health development community members are the chance to convince mother… in KII / 2:34 ¶ 53, Encourage women education most importantly in the whole society. Use o… in KII / 3:21 ¶ 12, There may be a way perhaps to change the current ignorant behavior of… in Group discussion / 3:40 ¶ 24, sing mHealth consultation, providing care, or treating patient or clie… in Group discussion / 3:45 ¶ 28, After mothers forum or community mobilization our service our work may… in Group discussion / 3:56 ¶ 41, wareness in the community and improve works in those area according to… in Group discussion

- **Awareness creation: women_awarness**

19 Quotations:

1:1 ¶ 16, If one mother served all will need to take that will aslo motivate hou… in interview analysis / 1:67 ¶ 69, After mothers understand the service they will use and since they have… in interview analysis / 1:69 ¶ 70, onsultation, treatment follow up, other health education can be given… in interview analysis / 1:71 ¶ 73, I do not think there will a problem because we have to teach train and… in interview analysis / 1:73 ¶ 74, e can also be aware and work on behavioral change for not accepting mo… in interview analysis / 1:75 ¶ 76, I don't think it will have any problem because when we tell mother "it… in interview analysis / 1:101 ¶ 100, Throughout mothers may need support. We can help with understanding. I… in interview analysis / 1:105 ¶ 105, We have to teach mothers how to use mobile, what is mHealth, how to op… in interview analysis / 2:14 ¶ 26, For mother it may need social and BCC to get it welcomed completely in KII / 2:23 ¶ 36, Health development community members are the chance to convince mother… in KII / 2:34 ¶ 53, Encourage women education most importantly in the whole society. Use o… in KII / 3:16 ¶ 10, Whatever the mother say either for refusal I think we can convince the… in Group discussion / 3:17 ¶ 12, May be this mobile service can be accompanied with behavioral change e… in Group discussion / 3:21 ¶ 12, There may be a way perhaps to change the current ignorant behavior of… in Group discussion / 3:26 ¶ 14, They way of following success and promotion should be continuous.

Moth… in Group discussion / 3:32 ¶ 19, This will create an option to counsel and follow up mothers are not co… in Group discussion / 3:39 ¶ 24, Consultation is what we usually do when circumstances allow us to do in Group discussion / 3:40 ¶ 24, sing mHealth consultation, providing care, or treating patient or clie… in Group discussion / 3:64 ¶ 51, Reading ability. Many SMS sent by Ethio-tele. every day, so this need… in Group discussion

1:67 ¶ 69 in interview analysis

Text quotation

Created by Girma Gilano on 3/8/2023, **modified** by Girma Gilano on 3/8/2023

After mothers understand the service they will use and since they have access it will improve the service. first mothers need awareness after that the service can be effective

1 Codes:

- **Awareness creation: women_awarness**

19 Quotations:

1:1 ¶ 16, If one mother served all will need to take that will aslo motivate hou… in interview analysis / 1:67 ¶ 69, After mothers understand the service they will use and since they have… in interview analysis / 1:69 ¶ 70, onsultation, treatment follow up, other health education can be given… in interview analysis / 1:71 ¶ 73, I do not think there will a problem because we have to teach train and… in interview analysis / 1:73 ¶ 74, e can also be aware and work on behavioral change for not accepting mo… in interview analysis / 1:75 ¶ 76, I don't think it will have any problem because when we tell mother "it… in interview analysis / 1:101 ¶ 100, Throughout mothers may need support. We can help with understanding. I… in interview analysis / 1:105 ¶ 105, We have to teach mothers how to use mobile, what is mHealth, how to op… in interview analysis / 2:14 ¶ 26, For mother it may need social and BCC to get it welcomed completely in KII / 2:23 ¶ 36, Health development community members are the chance to convince mother… in KII / 2:34 ¶ 53, Encourage women education most importantly in the whole society. Use o… in KII / 3:16 ¶ 10, Whatever the mother say either for refusal I think we can convince the… in Group discussion / 3:17 ¶ 12, May be this mobile service can be accompanied with behavioral change e… in Group discussion / 3:21 ¶ 12, There may be a way perhaps to change the current ignorant behavior of… in Group discussion / 3:26 ¶ 14, They way of following success and promotion should be continuous. Moth… in Group discussion / 3:32 ¶ 19, This will create an option to counsel and follow up mothers are not co… in Group discussion / 3:39 ¶ 24, Consultation is what we usually do when circumstances allow us to do in Group discussion / 3:40 ¶ 24, sing mHealth consultation, providing care, or treating patient or clie… in Group discussion / 3:64 ¶ 51, Reading ability. Many SMS sent by Ethio-tele. every day, so this need… in Group discussion

1:69 ¶ 70 in interview analysis

Text quotation

Created by Girma Gilano on 3/8/2023

onsultation, treatment follow up, other health education can be given through health mHealth

1 Codes:

- **Awareness creation: women_awarness**

19 Quotations:

1:1 ¶ 16, If one mother served all will need to take that will aslo motivate hou… in interview analysis / 1:67 ¶ 69, After mothers understand the service they will use and since they have… in interview analysis / 1:69 ¶ 70, onsultation, treatment follow up, other health education can be given… in interview analysis / 1:71 ¶ 73, I do not think there will a problem because we have to teach train and… in interview analysis / 1:73 ¶ 74, e can also be aware and work on behavioral change for not accepting mo… in interview analysis / 1:75 ¶ 76, I don't think it will have any problem because when we tell mother "it… in interview analysis / 1:101 ¶ 100, Throughout mothers may need support. We can help with understanding. I… in interview analysis / 1:105 ¶ 105, We have to teach mothers how to use mobile, what is mHealth, how to op… in interview analysis / 2:14 ¶ 26, For mother it may need social and BCC to get it welcomed completely in KII / 2:23 ¶ 36, Health development community members are the chance to convince mother… in KII / 2:34 ¶ 53, Encourage women education most importantly in the whole society. Use o… in KII / 3:16 ¶ 10, Whatever the mother say either for refusal I think we can convince the… in Group discussion / 3:17 ¶ 12, May be this mobile service can be accompanied with behavioral change e… in Group discussion / 3:21 ¶ 12, There may be a way perhaps to change the current ignorant behavior of… in Group discussion / 3:26 ¶ 14, They way of following success and promotion should be continuous. Moth… in Group discussion / 3:32 ¶ 19, This will create an option to counsel and follow up mothers are not co… in Group discussion / 3:39 ¶ 24, Consultation is what we usually do when circumstances allow us to do in Group discussion / 3:40 ¶ 24, sing mHealth consultation, providing care, or treating patient or clie… in Group discussion / 3:64 ¶ 51, Reading ability. Many SMS sent by Ethio-tele. every day, so this need… in Group discussion

1:71 ¶ 73 in interview analysis

Text quotation

Created by Girma Gilano on 3/8/2023

I do not think there will a problem because we have to teach train and aware mothers

1 Codes:

- **Awareness creation: women_awarness**

19 Quotations:

1:1 ¶ 16, If one mother served all will need to take that will aslo motivate hou… in interview analysis / 1:67 ¶ 69, After mothers understand the service they will use and since they have… in interview analysis / 1:69 ¶ 70, onsultation, treatment follow up, other health education can be given… in interview analysis / 1:71 ¶ 73, I do not think there will a problem because we have to teach train and… in interview analysis / 1:73 ¶ 74, e can also be aware and work on behavioral change for not accepting mo… in interview analysis / 1:75 ¶ 76, I don't think it will have any problem because when we tell mother "it… in interview analysis / 1:101 ¶ 100, Throughout mothers may need support. We can help with understanding. I… in interview analysis / 1:105 ¶ 105, We have to teach mothers how to use mobile, what is mHealth, how to op… in interview analysis / 2:14 ¶ 26, For mother it may need social and BCC to get it welcomed completely in KII / 2:23 ¶ 36, Health development community members are the chance to convince mother… in KII / 2:34 ¶ 53, Encourage women education most importantly in the whole society. Use o… in KII / 3:16 ¶ 10, Whatever the mother say either for refusal I think we can convince the… in Group discussion / 3:17 ¶ 12, May be this mobile service can be accompanied with behavioral change e… in Group discussion / 3:21 ¶ 12, There may be a way perhaps to change the current ignorant behavior of… in Group discussion / 3:26 ¶ 14, They way of following success and promotion should be continuous. Moth… in Group discussion / 3:32 ¶ 19, This will create an option to counsel and follow up mothers are not co… in Group discussion / 3:39 ¶ 24, Consultation is what we usually do when circumstances allow us to do in Group discussion / 3:40 ¶ 24, sing mHealth consultation, providing care, or treating patient or clie… in Group discussion / 3:64 ¶ 51, Reading ability. Many SMS sent by Ethio-tele. every day, so this need… in Group discussion

1:73 ¶ 74 in interview analysis

Text quotation

Created by Girma Gilano on 3/8/2023

e can also be aware and work on behavioral change for not accepting mothers

2 Codes:

- **Awareness creation: provider_awarness**

4 Quotations:

1:21 ¶ 32, is is because of the need for familiarization or awareness creation bo… in interview analysis / 1:73 ¶ 74, e can also be aware and work on behavioral change for not accepting mo… in interview analysis / 2:20 ¶ 33, In Health institution awareness training for health profession, increa… in KII / 3:26 ¶ 14, They way of following success and promotion should be continuous. Moth… in Group discussion

- **Awareness creation: women_awarness**

19 Quotations:

1:1 ¶ 16, If one mother served all will need to take that will aslo motivate hou… in interview analysis / 1:67 ¶ 69, After mothers understand the service they will use and since they have… in interview analysis / 1:69 ¶ 70, onsultation, treatment follow up, other health education can be given… in interview analysis / 1:71 ¶ 73, I do not think there will a problem because we have to teach train and… in interview analysis / 1:73 ¶ 74, e can also be aware and work on behavioral change for not accepting mo… in interview analysis / 1:75 ¶ 76, I don't think it will have any problem because when we tell mother "it… in interview analysis / 1:101 ¶ 100, Throughout mothers may need support. We can help with understanding. I… in interview analysis / 1:105 ¶ 105, We have to teach mothers how to use mobile, what is mHealth, how to op… in interview analysis / 2:14 ¶ 26, For mother it may need social and BCC to get it welcomed completely in KII / 2:23 ¶ 36, Health development community members are the chance to convince mother… in KII / 2:34 ¶ 53, Encourage women education most importantly in the whole society. Use o… in KII / 3:16 ¶ 10, Whatever the mother say either for refusal I think we can convince the… in Group discussion / 3:17 ¶ 12, May be this mobile service can be accompanied with behavioral change e… in Group discussion / 3:21 ¶ 12, There may be a way perhaps to change the current ignorant behavior of… in Group discussion / 3:26 ¶ 14, They way of following success and promotion should be continuous. Moth… in Group discussion / 3:32 ¶ 19, This will create an option to counsel and follow up mothers are not co… in Group discussion / 3:39 ¶ 24, Consultation is what we usually do when circumstances allow us to do in Group discussion / 3:40 ¶ 24, sing mHealth consultation, providing care, or treating patient or clie… in Group discussion / 3:64 ¶ 51, Reading ability. Many SMS sent by Ethio-tele. every day, so this need… in Group discussion

1:75 ¶ 76 in interview analysis

Text quotation

Created by Girma Gilano on 3/8/2023, **modified** by Girma Gilano on 3/8/2023

I don't think it will have any problem because when we tell mother "it will help her accept". Further education is necessary before implementing anything

1 Codes:

- **Awareness creation: women_awarness**

19 Quotations:

1:1 ¶ 16, If one mother served all will need to take that will aslo motivate hou… in interview analysis / 1:67 ¶ 69, After mothers understand the service they will use and since they have… in interview analysis / 1:69 ¶ 70, onsultation, treatment follow up, other health education can be given… in interview analysis / 1:71 ¶ 73, I do not think there will a problem because we have to teach train and… in interview analysis / 1:73 ¶ 74, e can also be aware and work on behavioral change for not accepting mo… in interview analysis / 1:75 ¶ 76, I don’t think it will have any problem because when we tell mother “it… in interview analysis / 1:101 ¶ 100, Throughout mothers may need support. We can help with understanding. I… in interview analysis / 1:105 ¶ 105, We have to teach mothers how to use mobile, what is mHealth, how to op… in interview analysis / 2:14 ¶ 26, For mother it may need social and BCC to get it welcomed completely in KII / 2:23 ¶ 36, Health development community members are the chance to convince mother… in KII / 2:34 ¶ 53, Encourage women education most importantly in the whole society. Use o… in KII / 3:16 ¶ 10, Whatever the mother say either for refusal I think we can convince the… in Group discussion / 3:17 ¶ 12, May be this mobile service can be accompanied with behavioral change e… in Group discussion / 3:21 ¶ 12, There may be a way perhaps to change the current ignorant behavior of… in Group discussion / 3:26 ¶ 14, They way of following success and promotion should be continuous. Moth… in Group discussion / 3:32 ¶ 19, This will create an option to counsel and follow up mothers are not co… in Group discussion / 3:39 ¶ 24, Consultation is what we usually do when circumstances allow us to do in Group discussion / 3:40 ¶ 24, sing mHealth consultation, providing care, or treating patient or clie… in Group discussion / 3:64 ¶ 51, Reading ability. Many SMS sent by Ethio-tele. every day, so this need… in Group discussion

1:101 ¶ 100 in interview analysis

Text quotation

Created by Girma Gilano on 3/9/2023

Throughout mothers may need support. We can help with understanding. If the mother can read, have mobile, able to understand there will be no more obstacles

1 Codes:

- **Awareness creation: women_awarness**

19 Quotations:

1:1 ¶ 16, If one mother served all will need to take that will aslo motivate hou… in interview analysis / 1:67 ¶ 69, After mothers understand the service they will use and since they have… in interview analysis / 1:69 ¶ 70, onsultation, treatment follow up, other health education can be given… in interview analysis / 1:71 ¶ 73, I do not think there will a problem because we have to teach train and… in interview analysis / 1:73 ¶ 74, e can also be aware and work on behavioral change for not accepting mo… in interview analysis / 1:75 ¶ 76, I don’t think it will have any problem because when we tell mother “it… in interview analysis / 1:101 ¶ 100, Throughout mothers may need support. We can help with understanding. I… in interview analysis / 1:105 ¶ 105, We have to teach mothers how to use mobile, what is mHealth, how to op… in interview analysis / 2:14 ¶ 26, For mother it may need social and BCC to get it welcomed completely in KII / 2:23 ¶ 36, Health development community members are the chance to convince mother… in KII / 2:34 ¶ 53, Encourage women education most importantly in the whole society. Use o… in KII / 3:16 ¶ 10, Whatever the mother say either for refusal I think we can convince the… in Group discussion / 3:17 ¶ 12, May be this mobile service can be accompanied with behavioral change e… in Group discussion / 3:21 ¶ 12, There may be a way perhaps to change the current ignorant behavior of… in Group discussion / 3:26 ¶ 14, They way of following success and promotion should be continuous. Moth… in Group discussion / 3:32 ¶ 19, This will create an option to counsel and follow up mothers are not co… in Group discussion / 3:39 ¶ 24, Consultation is what we usually do when circumstances allow

us to do in Group discussion / 3:40 ¶ 24, sing mHealth consultation, providing care, or treating patient or clie… in Group discussion / 3:64 ¶ 51, Reading ability. Many SMS sent by Ethio-tele. every day, so this need… in Group discussion

1:105 ¶ 105 in interview analysis

Text quotation

Created by Girma Gilano on 3/9/2023

We have to teach mothers how to use mobile, what is mHealth, how to open, and others. If all input resources are fulfilled, there will be no problem

1 Codes:

- **Awareness creation: women_awarness**

19 Quotations:

1:1 ¶ 16, If one mother served all will need to take that will aslo motivate hou… in interview analysis / 1:67 ¶ 69, After mothers understand the service they will use and since they have… in interview analysis / 1:69 ¶ 70, onsultation, treatment follow up, other health education can be given… in interview analysis / 1:71 ¶ 73, I do not think there will a problem because we have to teach train and… in interview analysis / 1:73 ¶ 74, e can also be aware and work on behavioral change for not accepting mo… in interview analysis / 1:75 ¶ 76, I don't think it will have any problem because when we tell mother "it… in interview analysis / 1:101 ¶ 100, Throughout mothers may need support. We can help with understanding. I… in interview analysis / 1:105 ¶ 105, We have to teach mothers how to use mobile, what is mHealth, how to op… in interview analysis / 2:14 ¶ 26, For mother it may need social and BCC to get it welcomed completely in KII / 2:23 ¶ 36, Health development community members are the chance to convince mother… in KII / 2:34 ¶ 53, Encourage women education most importantly in the whole society. Use o… in KII / 3:16 ¶ 10, Whatever the mother say either for refusal I think we can convince the… in Group discussion / 3:17 ¶ 12, May be this mobile service can be accompanied with behavioral change e… in Group discussion / 3:21 ¶ 12, There may be a way perhaps to change the current ignorant behavior of… in Group discussion / 3:26 ¶ 14, They way of following success and promotion should be continuous. Moth… in Group discussion / 3:32 ¶ 19, This will create an option to counsel and follow up mothers are not co… in Group discussion / 3:39 ¶ 24, Consultation is what we usually do when circumstances allow us to do in Group discussion / 3:40 ¶ 24, sing mHealth consultation, providing care, or treating patient or clie… in Group discussion / 3:64 ¶ 51, Reading ability. Many SMS sent by Ethio-tele. every day, so this need… in Group discussion

2:14 ¶ 26 in KII

Text quotation

Created by Girma Gilano on 3/9/2023

For mother it may need social and BCC to get it welcomed completely

1 Codes:

- **Awareness creation: women_awarness**

19 Quotations:

1:1 ¶ 16, If one mother served all will need to take that will aslo motivate hou… in interview analysis / 1:67 ¶ 69, After mothers understand the service they will use and since they have… in interview analysis / 1:69 ¶ 70, onsultation, treatment follow up, other health education can be given… in interview analysis / 1:71 ¶ 73, I do not think there will a problem because we have to teach train and… in interview analysis / 1:73 ¶ 74, e can also be aware and work on behavioral change for not accepting mo… in interview analysis / 1:75 ¶ 76, I don't think it will have any problem because when we tell mother "it… in interview analysis / 1:101 ¶ 100, Throughout mothers may need support. We can help with understanding. I… in interview analysis / 1:105 ¶ 105, We have to teach mothers how to use mobile, what is mHealth, how to op… in interview analysis / 2:14 ¶ 26, For mother it may need social and BCC to get it welcomed completely in KII / 2:23 ¶ 36, Health development community members are the chance to convince mother… in KII / 2:34 ¶ 53, Encourage women education most importantly in the whole society. Use o… in KII / 3:16 ¶ 10, Whatever the mother say either for refusal I think we can convince the… in Group discussion / 3:17 ¶ 12, May be this mobile service can be accompanied with behavioral change e… in Group discussion / 3:21 ¶ 12, There may be a way perhaps to change the current ignorant behavior of… in Group discussion / 3:26 ¶ 14, They way of following success and promotion should be continuous. Moth… in Group discussion / 3:32 ¶ 19, This will create an option to counsel and follow up mothers are not co… in Group discussion / 3:39 ¶ 24, Consultation is what we usually do when circumstances allow us to do in Group discussion / 3:40 ¶ 24, sing mHealth consultation, providing care, or treating patient or clie… in Group discussion / 3:64 ¶ 51, Reading ability. Many SMS sent by Ethio-tele. every day, so this need… in Group discussion

2:23 ¶ 36 in KII

Text quotation

Created by Girma Gilano on 3/9/2023

Health development community members are the chance to convince mother and monitor. Currently due to the sanction we are suffering a lot, but when everything become alright, I think this the best thing our community deserves.

2 Codes:

- **Awareness creation: community_awarness**

8 Quotations:

1:1 ¶ 16, If one mother served all will need to take that will aslo motivate hou… in interview analysis / 1:21 ¶ 32, is is because of the need for familiarization or awareness creation bo… in interview analysis / 2:23 ¶ 36, Health development community members are the chance to convince mother… in KII / 2:34 ¶ 53, Encourage women education most importantly in the whole society. Use o… in KII / 3:21 ¶ 12, There may be a way perhaps to change the current ignorant behavior of… in Group discussion / 3:40 ¶ 24, sing mHealth consultation, providing care, or treating patient or clie… in Group discussion / 3:45 ¶ 28, After mothers forum or community mobilization our service our work may… in Group discussion / 3:56 ¶ 41, wareness in the community and improve works in those area according to… in Group discussion

- **Awareness creation: women_awarness**

19 Quotations:

1:1 ¶ 16, If one mother served all will need to take that will aslo motivate hou… in interview analysis / 1:67 ¶ 69, After mothers understand the service they will use and since they have… in interview analysis / 1:69 ¶ 70, onsultation, treatment follow up, other health education can be given… in interview analysis / 1:71 ¶ 73, I do not think there will a problem because we have to teach train and… in interview analysis / 1:73 ¶ 74, e can also be aware and work on behavioral change for not accepting mo… in interview analysis / 1:75 ¶ 76, I don't think it will have any problem because when we tell mother "it… in interview analysis / 1:101 ¶ 100, Throughout mothers may need support. We can help with understanding. I… in

interview analysis / 1:105 ¶ 105, We have to teach mothers how to use mobile, what is mHealth, how to op… in interview analysis / 2:14 ¶ 26, For mother it may need social and BCC to get it welcomed completely in KII / 2:23 ¶ 36, Health development community members are the chance to convince mother… in KII / 2:34 ¶ 53, Encourage women education most importantly in the whole society. Use o… in KII / 3:16 ¶ 10, Whatever the mother say either for refusal I think we can convince the… in Group discussion / 3:17 ¶ 12, May be this mobile service can be accompanied with behavioral change e… in Group discussion / 3:21 ¶ 12, There may be a way perhaps to change the current ignorant behavior of… in Group discussion / 3:26 ¶ 14, They way of following success and promotion should be continuous. Moth… in Group discussion / 3:32 ¶ 19, This will create an option to counsel and follow up mothers are not co… in Group discussion / 3:39 ¶ 24, Consultation is what we usually do when circumstances allow us to do in Group discussion / 3:40 ¶ 24, sing mHealth consultation, providing care, or treating patient or clie… in Group discussion / 3:64 ¶ 51, Reading ability. Many SMS sent by Ethio-tele. every day, so this need… in Group discussion

2:34 ¶ 53 in KII

Text quotation

Created by Girma Gilano on 3/9/2023

Encourage women education most importantly in the whole society. Use of education as a tool to have good pregnancy.

2 Codes:

- **Awareness creation: community_awarness**

8 Quotations:

1:1 ¶ 16, If one mother served all will need to take that will aslo motivate hou… in interview analysis / 1:21 ¶ 32, is is because of the need for familiarization or awareness creation bo… in interview analysis / 2:23 ¶ 36, Health development community members are the chance to convince mother… in KII / 2:34 ¶ 53, Encourage women education most importantly in the whole society. Use o… in KII / 3:21 ¶ 12, There may be a way perhaps to change the current ignorant behavior of… in Group discussion / 3:40 ¶ 24, sing mHealth consultation, providing care, or treating patient or clie… in Group discussion / 3:45 ¶ 28, After mothers forum or community mobilization our service our work may… in Group discussion / 3:56 ¶ 41, wareness in the community and improve works in those area according to… in Group discussion

- **Awareness creation: women_awarness**

19 Quotations:

1:1 ¶ 16, If one mother served all will need to take that will aslo motivate hou… in interview analysis / 1:67 ¶ 69, After mothers understand the service they will use and since they have… in interview analysis / 1:69 ¶ 70, onsultation, treatment follow up, other health education can be given… in interview analysis / 1:71 ¶ 73, I do not think there will a problem because we have to teach train and… in interview analysis / 1:73 ¶ 74, e can also be aware and work on behavioral change for not accepting mo… in interview analysis / 1:75 ¶ 76, I don't think it will have any problem because when we tell mother "it… in interview analysis / 1:101 ¶ 100, Throughout mothers may need support. We can help with understanding. I… in interview analysis / 1:105 ¶ 105, We have to teach mothers how to use mobile, what is mHealth, how to op… in interview analysis / 2:14 ¶ 26, For mother it may need social and BCC to get it welcomed completely in KII / 2:23 ¶ 36, Health development community members are the chance to convince mother… in KII / 2:34 ¶ 53, Encourage women education most importantly in the whole society. Use o… in KII / 3:16 ¶ 10, Whatever the mother say either for refusal I think we can convince the… in Group discussion / 3:17 ¶ 12, May be this mobile service can be accompanied with behavioral change e… in Group discussion / 3:21 ¶ 12, There may be a way perhaps to change the current ignorant behavior of… in Group discussion / 3:26 ¶ 14, They way of following success and promotion should be continuous.

Moth… in Group discussion / 3:32 ¶ 19, This will create an option to counsel and follow up mothers are not co… in Group discussion / 3:39 ¶ 24, Consultation is what we usually do when circumstances allow us to do in Group discussion / 3:40 ¶ 24, sing mHealth consultation, providing care, or treating patient or clie… in Group discussion / 3:64 ¶ 51, Reading ability. Many SMS sent by Ethio-tele. every day, so this need… in Group discussion

3:16 ¶ 10 in Group discussion

Text quotation

Created by Girma Gilano on 3/9/2023

Whatever the mother say either for refusal I think we can convince them. Everything at initial point has its own struggling and final accepted so there will be no new things here, we teach them they accep

1 Codes:

- **Awareness creation: women_awarness**

19 Quotations:

1:1 ¶ 16, If one mother served all will need to take that will aslo motivate hou… in interview analysis / 1:67 ¶ 69, After mothers understand the service they will use and since they have… in interview analysis / 1:69 ¶ 70, onsultation, treatment follow up, other health education can be given… in interview analysis / 1:71 ¶ 73, I do not think there will a problem because we have to teach train and… in interview analysis / 1:73 ¶ 74, e can also be aware and work on behavioral change for not accepting mo… in interview analysis / 1:75 ¶ 76, I don't think it will have any problem because when we tell mother "it… in interview analysis / 1:101 ¶ 100, Throughout mothers may need support. We can help with understanding. I… in interview analysis / 1:105 ¶ 105, We have to teach mothers how to use mobile, what is mHealth, how to op… in interview analysis / 2:14 ¶ 26, For mother it may need social and BCC to get it welcomed completely in KII / 2:23 ¶ 36, Health development community members are the chance to convince mother… in KII / 2:34 ¶ 53, Encourage women education most importantly in the whole society. Use o… in KII / 3:16 ¶ 10, Whatever the mother say either for refusal I think we can convince the… in Group discussion / 3:17 ¶ 12, May be this mobile service can be accompanied with behavioral change e… in Group discussion / 3:21 ¶ 12, There may be a way perhaps to change the current ignorant behavior of… in Group discussion / 3:26 ¶ 14, They way of following success and promotion should be continuous. Moth… in Group discussion / 3:32 ¶ 19, This will create an option to counsel and follow up mothers are not co… in Group discussion / 3:39 ¶ 24, Consultation is what we usually do when circumstances allow us to do in Group discussion / 3:40 ¶ 24, sing mHealth consultation, providing care, or treating patient or clie… in Group discussion / 3:64 ¶ 51, Reading ability. Many SMS sent by Ethio-tele. every day, so this need… in Group discussion

3:17 ¶ 12 in Group discussion

Text quotation

Created by Girma Gilano on 3/9/2023

May be this mobile service can be accompanied with behavioral change educations

1 Codes:

- **Awareness creation: women_awarness**

19 Quotations:

1:1 ¶ 16, If one mother served all will need to take that will aslo motivate hou… in interview analysis / 1:67 ¶ 69, After mothers understand the service they will use and since they have… in interview analysis / 1:69 ¶ 70, onsultation, treatment follow up, other health education can be given… in interview analysis / 1:71 ¶ 73, I do not think there will a problem because we have to teach train and… in interview analysis / 1:73 ¶ 74, e can also be aware and work on behavioral change for not accepting mo… in interview analysis / 1:75 ¶ 76, I don't think it will have any problem because when we tell mother "it… in interview analysis / 1:101 ¶ 100, Throughout mothers may need support. We can help with understanding. I… in interview analysis / 1:105 ¶ 105, We have to teach mothers how to use mobile, what is mHealth, how to op… in interview analysis / 2:14 ¶ 26, For mother it may need social and BCC to get it welcomed completely in KII / 2:23 ¶ 36, Health development community members are the chance to convince mother… in KII / 2:34 ¶ 53, Encourage women education most importantly in the whole society. Use o… in KII / 3:16 ¶ 10, Whatever the mother say either for refusal I think we can convince the… in Group discussion / 3:17 ¶ 12, May be this mobile service can be accompanied with behavioral change e… in Group discussion / 3:21 ¶ 12, There may be a way perhaps to change the current ignorant behavior of… in Group discussion / 3:26 ¶ 14, They way of following success and promotion should be continuous. Moth… in Group discussion / 3:32 ¶ 19, This will create an option to counsel and follow up mothers are not co… in Group discussion / 3:39 ¶ 24, Consultation is what we usually do when circumstances allow us to do in Group discussion / 3:40 ¶ 24, sing mHealth consultation, providing care, or treating patient or clie… in Group discussion / 3:64 ¶ 51, Reading ability. Many SMS sent by Ethio-tele. every day, so this need… in Group discussion

3:21 ¶ 12 in Group discussion

Text quotation

Created by Girma Gilano on 3/9/2023

There may be a way perhaps to change the current ignorant behavior of mothers

2 Codes:

- **Awareness creation: community_awarness**

8 Quotations:

1:1 ¶ 16, If one mother served all will need to take that will aslo motivate hou… in interview analysis / 1:21 ¶ 32, is is because of the need for familiarization or awareness creation bo… in interview analysis / 2:23 ¶ 36, Health development community members are the chance to convince mother… in KII / 2:34 ¶ 53, Encourage women education most importantly in the whole society. Use o… in KII / 3:21 ¶ 12, There may be a way perhaps to change the current ignorant behavior of… in Group discussion / 3:40 ¶ 24, sing mHealth consultation, providing care, or treating patient or clie… in Group discussion / 3:45 ¶ 28, After mothers forum or community mobilization our service our work may… in Group discussion / 3:56 ¶ 41, wareness in the community and improve works in those area according to… in Group discussion

- **Awareness creation: women_awarness**

19 Quotations:

1:1 ¶ 16, If one mother served all will need to take that will aslo motivate hou… in interview analysis / 1:67 ¶ 69, After mothers understand the service they will use and since they have… in interview analysis / 1:69 ¶ 70, onsultation, treatment follow up, other health education can be given… in interview analysis / 1:71 ¶ 73, I do not think there will a problem because we have to teach train and… in interview analysis / 1:73 ¶ 74, e can also be aware and work on behavioral change for not accepting mo… in interview analysis / 1:75 ¶ 76, I don't think it will have any problem because when we tell mother "it… in interview analysis / 1:101 ¶ 100, Throughout mothers may need support. We can help with understanding. I… in

interview analysis / 1:105 ¶ 105, We have to teach mothers how to use mobile, what is mHealth, how to op… in interview analysis / 2:14 ¶ 26, For mother it may need social and BCC to get it welcomed completely in KII / 2:23 ¶ 36, Health development community members are the chance to convince mother… in KII / 2:34 ¶ 53, Encourage women education most importantly in the whole society. Use o… in KII / 3:16 ¶ 10, Whatever the mother say either for refusal I think we can convince the… in Group discussion / 3:17 ¶ 12, May be this mobile service can be accompanied with behavioral change e… in Group discussion / 3:21 ¶ 12, There may be a way perhaps to change the current ignorant behavior of… in Group discussion / 3:26 ¶ 14, They way of following success and promotion should be continuous. Moth… in Group discussion / 3:32 ¶ 19, This will create an option to counsel and follow up mothers are not co… in Group discussion / 3:39 ¶ 24, Consultation is what we usually do when circumstances allow us to do in Group discussion / 3:40 ¶ 24, sing mHealth consultation, providing care, or treating patient or clie… in Group discussion / 3:64 ¶ 51, Reading ability. Many SMS sent by Ethio-tele. every day, so this need… in Group discussion

3:26 ¶ 14 in Group discussion

Text quotation

Created by Girma Gilano on 3/9/2023

They way of following success and promotion should be continuous. Mother can be promote even to use and buy mobile phone to be pregnant and this can be common practice for women in the community. They may even ask a woman why she pregnant before having a phone

2 Codes:

- **Awareness creation: provider_awarness**

4 Quotations:

1:21 ¶ 32, is is because of the need for familiarization or awareness creation bo… in interview analysis / 1:73 ¶ 74, e can also be aware and work on behavioral change for not accepting mo… in interview analysis / 2:20 ¶ 33, In Health institution awareness training for health profession, increa… in KII / 3:26 ¶ 14, They way of following success and promotion should be continuous. Moth… in Group discussion

- **Awareness creation: women_awarness**

19 Quotations:

1:1 ¶ 16, If one mother served all will need to take that will aslo motivate hou… in interview analysis / 1:67 ¶ 69, After mothers understand the service they will use and since they have… in interview analysis / 1:69 ¶ 70, onsultation, treatment follow up, other health education can be given… in interview analysis / 1:71 ¶ 73, I do not think there will a problem because we have to teach train and… in interview analysis / 1:73 ¶ 74, e can also be aware and work on behavioral change for not accepting mo… in interview analysis / 1:75 ¶ 76, I don't think it will have any problem because when we tell mother "it… in interview analysis / 1:101 ¶ 100, Throughout mothers may need support. We can help with understanding. I… in interview analysis / 1:105 ¶ 105, We have to teach mothers how to use mobile, what is mHealth, how to op… in interview analysis / 2:14 ¶ 26, For mother it may need social and BCC to get it welcomed completely in KII / 2:23 ¶ 36, Health development community members are the chance to convince mother… in KII / 2:34 ¶ 53, Encourage women education most importantly in the whole society. Use o… in KII / 3:16 ¶ 10, Whatever the mother say either for refusal I think we can convince the… in Group discussion / 3:17 ¶ 12, May be this mobile service can be accompanied with behavioral change e… in Group discussion / 3:21 ¶ 12, There may be a way perhaps to change the current ignorant behavior of… in Group discussion / 3:26 ¶ 14, They way of following success and promotion should be continuous. Moth… in Group discussion / 3:32 ¶ 19, This will create an option to counsel and follow up mothers are

not co… in Group discussion / 3:39 ¶ 24, Consultation is what we usually do when circumstances allow us to do in Group discussion / 3:40 ¶ 24, sing mHealth consultation, providing care, or treating patient or clie… in Group discussion / 3:64 ¶ 51, Reading ability. Many SMS sent by Ethio-tele. every day, so this need… in Group discussion

3:32 ¶ 19 in Group discussion

Text quotation

Created by Girma Gilano on 3/9/2023

This will create an option to counsel and follow up mothers are not counseled, have special follow up issue, and some suspicious issues

1 Codes:

- **Awareness creation: women_awarness**

19 Quotations:

1:1 ¶ 16, If one mother served all will need to take that will aslo motivate hou… in interview analysis / 1:67 ¶ 69, After mothers understand the service they will use and since they have… in interview analysis / 1:69 ¶ 70, onsultation, treatment follow up, other health education can be given… in interview analysis / 1:71 ¶ 73, I do not think there will a problem because we have to teach train and… in interview analysis / 1:73 ¶ 74, e can also be aware and work on behavioral change for not accepting mo… in interview analysis / 1:75 ¶ 76, I don't think it will have any problem because when we tell mother "it… in interview analysis / 1:101 ¶ 100, Throughout mothers may need support. We can help with understanding. I… in interview analysis / 1:105 ¶ 105, We have to teach mothers how to use mobile, what is mHealth, how to op… in interview analysis / 2:14 ¶ 26, For mother it may need social and BCC to get it welcomed completely in KII / 2:23 ¶ 36, Health development community members are the chance to convince mother… in KII / 2:34 ¶ 53, Encourage women education most importantly in the whole society. Use o… in KII / 3:16 ¶ 10, Whatever the mother say either for refusal I think we can convince the… in Group discussion / 3:17 ¶ 12, May be this mobile service can be accompanied with behavioral change e… in Group discussion / 3:21 ¶ 12, There may be a way perhaps to change the current ignorant behavior of… in Group discussion / 3:26 ¶ 14, They way of following success and promotion should be continuous. Moth… in Group discussion / 3:32 ¶ 19, This will create an option to counsel and follow up mothers are not co… in Group discussion / 3:39 ¶ 24, Consultation is what we usually do when circumstances allow us to do in Group discussion / 3:40 ¶ 24, sing mHealth consultation, providing care, or treating patient or clie… in Group discussion / 3:64 ¶ 51, Reading ability. Many SMS sent by Ethio-tele. every day, so this need… in Group discussion

3:39 ¶ 24 in Group discussion

Text quotation

Created by Girma Gilano on 3/9/2023

Consultation is what we usually do when circumstances allow us to do

1 Codes:

- **Awareness creation: women_awarness**

19 Quotations:

1:1 ¶ 16, If one mother served all will need to take that will aslo motivate hou… in interview analysis / 1:67 ¶ 69, After mothers understand the service they will use and since they have… in interview analysis / 1:69 ¶ 70, onsultation, treatment follow up, other health education can be given… in interview analysis / 1:71 ¶ 73, I do not think there will a problem because we have to teach train and… in interview analysis / 1:73 ¶ 74, e can also be aware and work on behavioral change for not accepting mo… in interview analysis / 1:75 ¶ 76, I don't think it will have any problem because when we tell mother "it… in interview analysis / 1:101 ¶ 100, Throughout mothers may need support. We can help with understanding. I… in interview analysis / 1:105 ¶ 105, We have to teach mothers how to use mobile, what is mHealth, how to op… in interview analysis / 2:14 ¶ 26, For mother it may need social and BCC to get it welcomed completely in KII / 2:23 ¶ 36, Health development community members are the chance to convince mother… in KII / 2:34 ¶ 53, Encourage women education most importantly in the whole society. Use o… in KII / 3:16 ¶ 10, Whatever the mother say either for refusal I think we can convince the… in Group discussion / 3:17 ¶ 12, May be this mobile service can be accompanied with behavioral change e… in Group discussion / 3:21 ¶ 12, There may be a way perhaps to change the current ignorant behavior of… in Group discussion / 3:26 ¶ 14, They way of following success and promotion should be continuous. Moth… in Group discussion / 3:32 ¶ 19, This will create an option to counsel and follow up mothers are not co… in Group discussion / 3:39 ¶ 24, Consultation is what we usually do when circumstances allow us to do in Group discussion / 3:40 ¶ 24, sing mHealth consultation, providing care, or treating patient or clie… in Group discussion / 3:64 ¶ 51, Reading ability. Many SMS sent by Ethio-tele. every day, so this need… in Group discussion

3:40 ¶ 24 in Group discussion

Text quotation

Created by Girma Gilano on 3/9/2023

sing mHealth consultation, providing care, or treating patient or client look special because we can do whatever when we can or at any time.

3 Codes:

- **Awareness creation: community_awarness**

8 Quotations:

1:1 ¶ 16, If one mother served all will need to take that will aslo motivate hou… in interview analysis / 1:21 ¶ 32, is is because of the need for familiarization or awareness creation bo… in interview analysis / 2:23 ¶ 36, Health development community members are the chance to convince mother… in KII / 2:34 ¶ 53, Encourage women education most importantly in the whole society. Use o… in KII / 3:21 ¶ 12, There may be a way perhaps to change the current ignorant behavior of… in Group discussion / 3:40 ¶ 24, sing mHealth consultation, providing care, or treating patient or clie… in Group discussion / 3:45 ¶ 28, After mothers forum or community mobilization our service our work may… in Group discussion / 3:56 ¶ 41, wareness in the community and improve works in those area according to… in Group discussion

- **Awareness creation: family_awareness**

4 Quotations:

1:30 ¶ 37, I think if her husband is aware of the service, there will be no probl… in interview analysis / 3:40 ¶ 24, sing mHealth consultation, providing care, or treating patient or clie… in Group discussion / 3:45 ¶ 28, After mothers forum or community mobilization our service our work may… in Group discussion / 3:64 ¶ 51, Reading ability. Many SMS sent by Ethio-tele. every day, so this need… in Group discussion

- **Awareness creation: women_awarness**

19 Quotations:

1:1 ¶ 16, If one mother served all will need to take that will aslo motivate hou… in interview analysis / 1:67 ¶ 69, After mothers understand the service they will use and since they have… in interview analysis / 1:69 ¶ 70, onsultation, treatment follow up, other health education can be given… in interview analysis / 1:71 ¶ 73, I do not think there will a problem because we have to teach train and… in interview analysis / 1:73 ¶ 74, e can also be aware and work on behavioral change for not accepting mo… in interview analysis / 1:75 ¶ 76, I don't think it will have any problem because when we tell mother "it… in interview analysis / 1:101 ¶ 100, Throughout mothers may need support. We can help with understanding. I… in interview analysis / 1:105 ¶ 105, We have to teach mothers how to use mobile, what is mHealth, how to op… in interview analysis / 2:14 ¶ 26, For mother it may need social and BCC to get it welcomed completely in KII / 2:23 ¶ 36, Health development community members are the chance to convince mother… in KII / 2:34 ¶ 53, Encourage women education most importantly in the whole society. Use o… in KII / 3:16 ¶ 10, Whatever the mother say either for refusal I think we can convince the… in Group discussion / 3:17 ¶ 12, May be this mobile service can be accompanied with behavioral change e… in Group discussion / 3:21 ¶ 12, There may be a way perhaps to change the current ignorant behavior of… in Group discussion / 3:26 ¶ 14, They way of following success and promotion should be continuous. Moth… in Group discussion / 3:32 ¶ 19, This will create an option to counsel and follow up mothers are not co… in Group discussion / 3:39 ¶ 24, Consultation is what we usually do when circumstances allow us to do in Group discussion / 3:40 ¶ 24, sing mHealth consultation, providing care, or treating patient or clie… in Group discussion / 3:64 ¶ 51, Reading ability. Many SMS sent by Ethio-tele. every day, so this need… in Group discussion

3:64 ¶ 51 in Group discussion

Text quotation

Created by Girma Gilano on 3/9/2023

Reading ability. Many SMS sent by Ethio-tele. every day, so this need little awareness

2 Codes:

- **Awareness creation: family_awareness**

4 Quotations:

1:30 ¶ 37, I think if her husband is aware of the service, there will be no probl… in interview analysis / 3:40 ¶ 24, sing mHealth consultation, providing care, or treating patient or clie… in Group discussion / 3:45 ¶ 28, After mothers forum or community mobilization our service our work may… in Group discussion / 3:64 ¶ 51, Reading ability. Many SMS sent by Ethio-tele. every day, so this need… in Group discussion

- **Awareness creation: women_awarness**

19 Quotations:

1:1 ¶ 16, If one mother served all will need to take that will aslo motivate hou… in interview analysis / 1:67 ¶ 69, After mothers understand the service they will use and since they have… in interview analysis / 1:69 ¶ 70, onsultation, treatment follow up, other health education can be given… in interview analysis / 1:71 ¶ 73, I do not think there will a problem because we have to teach train and… in interview analysis / 1:73 ¶ 74, e can also be aware and work on behavioral change for not accepting mo… in interview analysis / 1:75 ¶ 76, I don't think it will have any problem because when we tell mother "it… in interview analysis / 1:101 ¶ 100, Throughout mothers may need support. We can help with understanding. I… in interview analysis / 1:105 ¶ 105, We have to teach mothers how to use mobile, what is mHealth, how to op… in interview analysis / 2:14 ¶ 26, For mother it may need social and BCC to get it welcomed completely in KII / 2:23 ¶ 36, Health development community members are the chance to convince

mother… in KII / 2:34 ¶ 53, Encourage women education most importantly in the whole society. Use o… in KII / 3:16 ¶ 10, Whatever the mother say either for refusal I think we can convince the… in Group discussion / 3:17 ¶ 12, May be this mobile service can be accompanied with behavioral change e… in Group discussion / 3:21 ¶ 12, There may be a way perhaps to change the current ignorant behavior of… in Group discussion / 3:26 ¶ 14, They way of following success and promotion should be continuous. Moth… in Group discussion / 3:32 ¶ 19, This will create an option to counsel and follow up mothers are not co… in Group discussion / 3:39 ¶ 24, Consultation is what we usually do when circumstances allow us to do in Group discussion / 3:40 ¶ 24, sing mHealth consultation, providing care, or treating patient or clie… in Group discussion / 3:64 ¶ 51, Reading ability. Many SMS sent by Ethio-tele. every day, so this need… in Group discussion

---

● Benefits of mHealth: ALarming

Created by Girma Gilano on 3/13/2023

Comment: by Girma Gilano

> *3/12/2023 11:46:46 AM, merged with Effectiviness 3/12/2023 1:12:09 PM, merged with Decision_making 3/12/2023 1:12:55 PM, merged with Role_improvement*

6 Quotations:

1:10 ¶ 24 in interview analysis

Text quotation

Created by Girma Gilano on 3/8/2023

it helps when people are busy with their work to remember appointments

1 Codes:

● Benefits of mHealth: ALarming

Comment: by Girma Gilano

> *3/12/2023 11:46:46 AM, merged with Effectiviness 3/12/2023 1:12:09 PM, merged with Decision_making 3/12/2023 1:12:55 PM, merged with Role_improvement*

6 Quotations:

1:10 ¶ 24, it helps when people are busy with their work to remember appointments in interview analysis / 1:25 ¶ 35, Mothers may not miss their appointment because they forget i in interview analysis / 1:53 ¶ 59, I think mHealth can improve access, appointment on time, availability… in interview analysis / 1:68 ¶ 70, Every system is getting digitalized so digitalizing health may have mu… in interview analysis / 3:27 ¶ 16, It can make the child health growth, for mothers to know danger signs,… in Group discussion / 3:47 ¶ 30, Appointment reminder, service on time, and reduce mortality, but befor… in Group discussion

1:25 ¶ 35 in interview analysis

Text quotation

Created by Girma Gilano on 3/8/2023

Mothers may not miss their appointment because they forget i

1 Codes:

- **Benefits of mHealth: ALarming**

Comment: by Girma Gilano

> *3/12/2023 11:46:46 AM, merged with Effectiviness 3/12/2023 1:12:09 PM, merged with Decision_making 3/12/2023 1:12:55 PM, merged with Role_improvement*

6 Quotations:

1:10 ¶ 24, it helps when people are busy with their work to remember appointments in interview analysis / 1:25 ¶ 35, Mothers may not miss their appointment because they forget i in interview analysis / 1:53 ¶ 59, I think mHealth can improve access, appointment on time, availability… in interview analysis / 1:68 ¶ 70, Every system is getting digitalized so digitalizing health may have mu… in interview analysis / 3:27 ¶ 16, It can make the child health growth, for mothers to know danger signs,… in Group discussion / 3:47 ¶ 30, Appointment reminder, service on time, and reduce mortality, but befor… in Group discussion

1:53 ¶ 59 in interview analysis

Text quotation

Created by Girma Gilano on 3/8/2023

I think mHealth can improve access, appointment on time, availability of information for decision making increased

2 Codes:

- **Benefits of mHealth: ALarming**

Comment: by Girma Gilano

> *3/12/2023 11:46:46 AM, merged with Effectiviness 3/12/2023 1:12:09 PM, merged with Decision_making 3/12/2023 1:12:55 PM, merged with Role_improvement*

6 Quotations:

1:10 ¶ 24, it helps when people are busy with their work to remember appointments in interview analysis / 1:25 ¶ 35, Mothers may not miss their appointment because they forget i in interview analysis / 1:53 ¶ 59, I think mHealth can improve access, appointment on time, availability… in interview analysis / 1:68 ¶ 70, Every system is getting digitalized so digitalizing health may have mu… in interview analysis / 3:27 ¶ 16, It can make the child health growth, for mothers to know danger signs,… in Group discussion / 3:47 ¶ 30, Appointment reminder, service on time, and reduce mortality, but befor… in Group discussion

- **Benefits of mHealth: Help_mothers**

Comment: by Girma Gilano

> *3/12/2023 11:46:46 AM, merged with Effectiviness 3/12/2023 1:12:09 PM, merged with Decision_making 3/12/2023 1:12:55 PM, merged with Role_improvement*

27 Quotations:

1:41 ¶ 47, ion comes after understanding of benefits and harms in interview analysis / 1:45 ¶ 52, Accessibility can be improved mothers get information at their homes in interview analysis / 1:51 ¶ 58,

She can access any information in interview analysis / 1:53 ¶ 59, I think mHealth can improve access, appointment on time, availability… in interview analysis / 1:65 ¶ 67, Her husband or children will also push her to go even if the condition… in interview analysis / 1:68 ¶ 70, Every system is getting digitalized so digitalizing health may have mu… in interview analysis / 1:77 ¶ 77, Rural mothers are respectful; they do what they told to do, so fear of… in interview analysis / 1:81 ¶ 80, pe mothers will turn to use mobiles, but only after getting its import… in interview analysis / 2:3 ¶ 11, It is helpful to also send information which are not appropriate in pe… in KII / 2:7 ¶ 17, Yeah, it will have effective change on community in KII / 2:9 ¶ 19, Yeah, previously mothers use the information they get during ANC, but… in KII / 2:11 ¶ 21, Information access, no transportation cost, and no professional resour… in KII / 2:36 ¶ 55, No need of motor or vehicles because it is mHealth. I will be happy to… in KII / 3:6 ¶ 5, They difference for mHealth is mother always get counseling or health… in Group discussion / 3:18 ¶ 12, It looks like mothers are tired of current service provision so the mH… in Group discussion / 3:23 ¶ 14, Previously, people think poor health system and there are many complia… in Group discussion / 3:24 ¶ 14, Over time this can change community perception and they may reebok mot… in Group discussion / 3:25 ¶ 14, There may be emerged women who are successful because of mHealth and t… in Group discussion / 3:27 ¶ 16, It can make the child health growth, for mothers to know danger signs,… in Group discussion / 3:38 ¶ 22, Of course having organized maternal data can also promote contacting a… in Group discussion / 3:41 ¶ 26, : It is a big opportunity to meet the patient again virtually especial… in Group discussion / 3:42 ¶ 26, Traditionally, when errors happen or if the patients go with the wrong… in Group discussion / 3:50 ¶ 33, Benefit for mothers for next pregnancies and healthy prospect for chil… in Group discussion / 3:59 ¶ 45, Satisfaction of their client service and familiarization with technolo… in Group discussion / 3:61 ¶ 47, Planning, decision making based on mHealth, and increased service take… in Group discussion / 3:62 ¶ 48, Support related to mHealth, fulfillment of deficiencies of human and o… in Group discussion / 3:67 ¶ 53, he respect for pregnancy mothers may think gave many births and not ca… in Group discussion

1:68 ¶ 70 in interview analysis

Text quotation

Created by Girma Gilano on 3/8/2023

Every system is getting digitalized so digitalizing health may have much advantage more than just appointme

3 Codes:

- **Benefits of mHealth: ALarming**

Comment: by Girma Gilano

> *3/12/2023 11:46:46 AM, merged with Effectiviness 3/12/2023 1:12:09 PM, merged with Decision_making 3/12/2023 1:12:55 PM, merged with Role_improvement*

6 Quotations:

1:10 ¶ 24, it helps when people are busy with their work to remember appointments in interview analysis / 1:25 ¶ 35, Mothers may not miss their appointment because they forget i in interview analysis / 1:53 ¶ 59, I think mHealth can improve access, appointment on time, availability… in interview analysis / 1:68 ¶ 70, Every system is getting digitalized so digitalizing health may have mu… in interview analysis / 3:27 ¶ 16, It can make the child health growth, for mothers to know danger signs,… in Group discussion / 3:47 ¶ 30, Appointment reminder, service on time, and reduce mortality, but befor… in Group discussion

- **Benefits of mHealth: Help_mothers**

Comment: by Girma Gilano

> *3/12/2023 11:46:46 AM, merged with Effectiviness 3/12/2023 1:12:09 PM, merged with Decision_making 3/12/2023 1:12:55 PM, merged with Role_improvement*

27 Quotations:

1:41 ¶ 47, ion comes after understanding of benefits and harms in interview analysis / 1:45 ¶ 52, Accessibility can be improved mothers get information at their homes in interview analysis / 1:51 ¶ 58, She can access any information in interview analysis / 1:53 ¶ 59, I think mHealth can improve access, appointment on time, availability… in interview analysis / 1:65 ¶ 67, Her husband or children will also push her to go even if the condition… in interview analysis / 1:68 ¶ 70, Every system is getting digitalized so digitalizing health may have mu… in interview analysis / 1:77 ¶ 77, Rural mothers are respectful; they do what they told to do, so fear of… in interview analysis / 1:81 ¶ 80, pe mothers will turn to use mobiles, but only after getting its import… in interview analysis / 2:3 ¶ 11, It is helpful to also send information which are not appropriate in pe… in KII / 2:7 ¶ 17, Yeah, it will have effective change on community in KII / 2:9 ¶ 19, Yeah, previously mothers use the information they get during ANC, but… in KII / 2:11 ¶ 21, Information access, no transportation cost, and no professional resour… in KII / 2:36 ¶ 55, No need of motor or vehicles because it is mHealth. I will be happy to… in KII / 3:6 ¶ 5, They difference for mHealth is mother always get counseling or health… in Group discussion / 3:18 ¶ 12, It looks like mothers are tired of current service provision so the mH… in Group discussion / 3:23 ¶ 14, Previously, people think poor health system and there are many complia… in Group discussion / 3:24 ¶ 14, Over time this can change community perception and they may reebok mot… in Group discussion / 3:25 ¶ 14, There may be emerged women who are successful because of mHealth and t… in Group discussion / 3:27 ¶ 16, It can make the child health growth, for mothers to know danger signs,… in Group discussion / 3:38 ¶ 22, Of course having organized maternal data can also promote contacting a… in Group discussion / 3:41 ¶ 26, : It is a big opportunity to meet the patient again virtually especial… in Group discussion / 3:42 ¶ 26, Traditionally, when errors happen or if the patients go with the wrong… in Group discussion / 3:50 ¶ 33, Benefit for mothers for next pregnancies and healthy prospect for chil… in Group discussion / 3:59 ¶ 45, Satisfaction of their client service and familiarization with technolo… in Group discussion / 3:61 ¶ 47, Planning, decision making based on mHealth, and increased service take… in Group discussion / 3:62 ¶ 48, Support related to mHealth, fulfillment of deficiencies of human and o… in Group discussion / 3:67 ¶ 53, he respect for pregnancy mothers may think gave many births and not ca… in Group discussion

● Benefits of mHealth: Help_professional

Comment: by Girma Gilano

> *3/12/2023 11:46:46 AM, merged with Effectiviness 3/12/2023 1:12:09 PM, merged with Decision_making 3/12/2023 1:12:55 PM, merged with Role_improvement*

30 Quotations:

1:24 ¶ 34, We always give best service to our best capacity, but mHealth may adva… in interview analysis / 1:34 ¶ 39, As HEWs we cannot reach for every mother on time but text can easily r… in interview analysis / 1:36 ¶ 40, This mHealth better than our previous our service in interview analysis / 1:50 ¶ 57, further improve the already improving service in interview analysis / 1:56 ¶ 62, Our current ANC and other formats now request mother to register their… in interview analysis / 1:68 ¶ 70, Every system is getting digitalized so digitalizing health may have mu… in interview analysis / 1:82 ¶ 81, ers usually take what we tell them, it will be successful. I don't thi… in interview analysis / 2:3 ¶ 11, It is helpful to also send information which are not appropriate in pe… in KII / 2:7 ¶ 17, Yeah, it will have effective change on community in KII / 2:9 ¶ 19, Yeah, previously mothers use the information they get during ANC, but… in KII / 2:35 ¶ 54, Since we have many gaps this should be seen as an opportunity in KII / 3:2 ¶ 3, Previous teaching of mother through home to home is going to be throug… in Group discussion / 3:12 ¶ 8, For educated mothers messages will be option less in Group discussion / 3:13 ¶ 8, Starting from me we feed additional food at 2months because the kind o… in Group discussion / 3:18 ¶ 12, It looks like mothers are tired of current service provision so the mH… in Group discussion / 3:25 ¶ 14, There may be emerged women who are successful because of mHealth and t… in Group discussion / 3:29 ¶ 17, As a health professionals applying technology can only easy our burden… in Group discussion / 3:34 ¶ 19, ecause of mHealth we may every address of women and now we can also ca… in Group discussion / 3:35 ¶ 20, We will provide the service for mothers we know now, but previously we… in Group discussion / 3:38 ¶ 22, Of course having organized maternal data can also promote contacting a… in Group discussion / 3:41 ¶ 26, : It is a big opportunity to meet the patient again virtually especial… in Group discussion / 3:42 ¶ 26, Traditionally, when errors happen or if the patients go with the wrong… in Group discussion / 3:43 ¶ 28, At the end this is very important piece of technology that can reduce… in Group discussion / 3:46 ¶ 28, Those infrastructure and awareness creation in staffs,

mothers, and co… in Group discussion / 3:52 ¶ 36, People will enjoy talking to others how health institution just concer… in Group discussion / 3:57 ¶ 42, Improve work environment as per the need of the technology and owning… in Group discussion / 3:58 ¶ 43, Aspiring more technologies and helping mothers, taking trainings, givi… in Group discussion / 3:60 ¶ 46, Experience with technology. Enable them to identify areas where mHealt… in Group discussion / 3:61 ¶ 47, Planning, decision making based on mHealth, and increased service take… in Group discussion / 3:62 ¶ 48, Support related to mHealth, fulfillment of deficiencies of human and o… in Group discussion

3:27 ¶ 16 in Group discussion

Text quotation

Created by Girma Gilano on 3/9/2023

It can make the child health growth, for mothers to know danger signs, and during PNC to keep herself from unnecessary early pregnancy. It has no problem as long as the message receiver is the mother or someone oriented on the issue

2 Codes:

Benefits of mHealth: ALarming

Comment: by Girma Gilano

> *3/12/2023 11:46:46 AM, merged with Effectiviness 3/12/2023 1:12:09 PM, merged with Decision_making 3/12/2023 1:12:55 PM, merged with Role_improvement*

6 Quotations:

1:10 ¶ 24, it helps when people are busy with their work to remember appointments in interview analysis / 1:25 ¶ 35, Mothers may not miss their appointment because they forget i in interview analysis / 1:53 ¶ 59, I think mHealth can improve access, appointment on time, availability… in interview analysis / 1:68 ¶ 70, Every system is getting digitalized so digitalizing health may have mu… in interview analysis / 3:27 ¶ 16, It can make the child health growth, for mothers to know danger signs,… in Group discussion / 3:47 ¶ 30, Appointment reminder, service on time, and reduce mortality, but befor… in Group discussion

Benefits of mHealth: Help_mothers

Comment: by Girma Gilano

> *3/12/2023 11:46:46 AM, merged with Effectiviness 3/12/2023 1:12:09 PM, merged with Decision_making 3/12/2023 1:12:55 PM, merged with Role_improvement*

27 Quotations:

1:41 ¶ 47, ion comes after understanding of benefits and harms in interview analysis / 1:45 ¶ 52, Accessibility can be improved mothers get information at their homes in interview analysis / 1:51 ¶ 58, She can access any information in interview analysis / 1:53 ¶ 59, I think mHealth can improve access, appointment on time, availability… in interview analysis / 1:65 ¶ 67, Her husband or children will also push her to go even if the condition… in interview analysis / 1:68 ¶ 70, Every system is getting digitalized so digitalizing health may have mu… in interview analysis / 1:77 ¶ 77, Rural mothers are respectful; they do what they told to do, so fear of… in interview analysis / 1:81 ¶ 80, pe mothers will turn to use mobiles, but only after getting its import… in interview analysis / 2:3 ¶ 11, It is helpful to also send information which are not appropriate in pe… in KII / 2:7 ¶ 17, Yeah, it will have effective change on community in KII / 2:9 ¶ 19, Yeah, previously mothers use the information they get during ANC, but… in KII / 2:11 ¶ 21, Information access, no transportation cost, and no professional resour… in KII / 2:36 ¶ 55, No need of motor or vehicles because it is mHealth. I will be happy to… in KII / 3:6 ¶ 5, They difference for mHealth is mother always get counseling or health… in Group discussion / 3:18 ¶ 12, It looks like mothers are tired of current service provision so the mH… in Group discussion / 3:23 ¶ 14, Previously, people think

poor health system and there are many complia… in Group discussion / 3:24 ¶ 14, Over time this can change community perception and they may reebok mot… in Group discussion / 3:25 ¶ 14, There may be emerged women who are successful because of mHealth and t… in Group discussion / 3:27 ¶ 16, It can make the child health growth, for mothers to know danger signs,… in Group discussion / 3:38 ¶ 22, Of course having organized maternal data can also promote contacting a… in Group discussion / 3:41 ¶ 26, : It is a big opportunity to meet the patient again virtually especial… in Group discussion / 3:42 ¶ 26, Traditionally, when errors happen or if the patients go with the wrong… in Group discussion / 3:50 ¶ 33, Benefit for mothers for next pregnancies and healthy prospect for chil… in Group discussion / 3:59 ¶ 45, Satisfaction of their client service and familiarization with technolo… in Group discussion / 3:61 ¶ 47, Planning, decision making based on mHealth, and increased service take… in Group discussion / 3:62 ¶ 48, Support related to mHealth, fulfillment of deficiencies of human and o… in Group discussion / 3:67 ¶ 53, he respect for pregnancy mothers may think gave many births and not ca… in Group discussion

3:47 ¶ 30 in Group discussion

Text quotation

Created by Girma Gilano on 3/9/2023

Appointment reminder, service on time, and reduce mortality, but before all these service awareness creation should take the first place.

1 Codes:

- **Benefits of mHealth: ALarming**

Comment: by Girma Gilano

> *3/12/2023 11:46:46 AM, merged with Effectiviness 3/12/2023 1:12:09 PM, merged with Decision_making 3/12/2023 1:12:55 PM, merged with Role_improvement*

6 Quotations:

1:10 ¶ 24, it helps when people are busy with their work to remember appointments in interview analysis / 1:25 ¶ 35, Mothers may not miss their appointment because they forget i in interview analysis / 1:53 ¶ 59, I think mHealth can improve access, appointment on time, availability… in interview analysis / 1:68 ¶ 70, Every system is getting digitalized so digitalizing health may have mu… in interview analysis / 3:27 ¶ 16, It can make the child health growth, for mothers to know danger signs,… in Group discussion / 3:47 ¶ 30, Appointment reminder, service on time, and reduce mortality, but befor… in Group discussion

---

Benefits of mHealth: Effective

Created by Girma Gilano on 3/13/2023

Comment: by Girma Gilano

> *3/12/2023 11:46:46 AM, merged with Effectiviness 3/12/2023 1:12:09 PM, merged with Decision_making 3/12/2023 1:12:55 PM, merged with Role_improvement*

6 Quotations:

1:48 ¶ 55 in interview analysis

Text quotation

Created by Girma Gilano on 3/8/2023

t can reduce cost, time, and other resources

1 Codes:

- **Benefits of mHealth: Effective**

  Comment: by Girma Gilano

  > *3/12/2023 11:46:46 AM, merged with Effectiviness 3/12/2023 1:12:09 PM, merged with Decision_making 3/12/2023 1:12:55 PM, merged with Role_improvement*

  6 Quotations:

  1:48 ¶ 55, t can reduce cost, time, and other resources in interview analysis / 1:60 ¶ 64, It will be very effective because it advances the services in interview analysis / 1:61 ¶ 65, I think it will be very effective because we can see the newly impleme… in interview analysis / 1:81 ¶ 80, pe mothers will turn to use mobiles, but only after getting its import… in interview analysis / 1:98 ¶ 98, If all input resources are fulfilled, there will be no problem. It is… in interview analysis / 2:7 ¶ 17, Yeah, it will have effective change on community in KII

1:60 ¶ 64 in interview analysis

Text quotation

Created by Girma Gilano on 3/8/2023

It will be very effective because it advances the services

1 Codes:

- **Benefits of mHealth: Effective**

  Comment: by Girma Gilano

  > *3/12/2023 11:46:46 AM, merged with Effectiviness 3/12/2023 1:12:09 PM, merged with Decision_making 3/12/2023 1:12:55 PM, merged with Role_improvement*

  6 Quotations:

  1:48 ¶ 55, t can reduce cost, time, and other resources in interview analysis / 1:60 ¶ 64, It will be very effective because it advances the services in interview analysis / 1:61 ¶ 65, I think it will be very effective because we can see the newly impleme… in interview analysis / 1:81 ¶ 80, pe mothers will turn to use mobiles, but only after getting its import… in interview analysis / 1:98 ¶ 98, If all input resources are fulfilled, there will be no problem. It is… in interview analysis / 2:7 ¶ 17, Yeah, it will have effective change on community in KII

1:61 ¶ 65 in interview analysis

Text quotation

Created by Girma Gilano on 3/8/2023

I think it will be very effective because we can see the newly implemented health insurance

1 Codes:

- **Benefits of mHealth: Effective**

  Comment: by Girma Gilano

  > *3/12/2023 11:46:46 AM, merged with Effectiviness 3/12/2023 1:12:09 PM, merged with Decision_making 3/12/2023 1:12:55 PM, merged with Role_improvement*

  #### 6 Quotations:

  1:48 ¶ 55, t can reduce cost, time, and other resources in interview analysis / 1:60 ¶ 64, It will be very effective because it advances the services in interview analysis / 1:61 ¶ 65, I think it will be very effective because we can see the newly impleme… in interview analysis / 1:81 ¶ 80, pe mothers will turn to use mobiles, but only after getting its import… in interview analysis / 1:98 ¶ 98, If all input resources are fulfilled, there will be no problem. It is… in interview analysis / 2:7 ¶ 17, Yeah, it will have effective change on community in KII

1:81 ¶ 80 in interview analysis

Text quotation

Created by Girma Gilano on 3/8/2023

pe mothers will turn to use mobiles, but only after getting its importance that why health education should be crucial part of this implementation

2 Codes:

- **Benefits of mHealth: Effective**

  Comment: by Girma Gilano

  > *3/12/2023 11:46:46 AM, merged with Effectiviness 3/12/2023 1:12:09 PM, merged with Decision_making 3/12/2023 1:12:55 PM, merged with Role_improvement*

  #### 6 Quotations:

  1:48 ¶ 55, t can reduce cost, time, and other resources in interview analysis / 1:60 ¶ 64, It will be very effective because it advances the services in interview analysis / 1:61 ¶ 65, I think it will be very effective because we can see the newly impleme… in interview analysis / 1:81 ¶ 80, pe mothers will turn to use mobiles, but only after getting its import… in interview analysis / 1:98 ¶ 98, If all input resources are fulfilled, there will be no problem. It is… in interview analysis / 2:7 ¶ 17, Yeah, it will have effective change on community in KII

- **Benefits of mHealth: Help_mothers**

  Comment: by Girma Gilano

  > *3/12/2023 11:46:46 AM, merged with Effectiviness 3/12/2023 1:12:09 PM, merged with Decision_making 3/12/2023 1:12:55 PM, merged with Role_improvement*

  #### 27 Quotations:

  1:41 ¶ 47, ion comes after understanding of benefits and harms in interview analysis / 1:45 ¶ 52, Accessibility can be improved mothers get information at their homes in interview analysis / 1:51 ¶ 58, She can access any information in interview analysis / 1:53 ¶ 59, I think mHealth can improve access, appointment on time, availability… in interview analysis / 1:65 ¶ 67, Her husband or children will also

push her to go even if the condition… in interview analysis / 1:68 ¶ 70, Every system is getting digitalized so digitalizing health may have mu… in interview analysis / 1:77 ¶ 77, Rural mothers are respectful; they do what they told to do, so fear of… in interview analysis / 1:81 ¶ 80, pe mothers will turn to use mobiles, but only after getting its import… in interview analysis / 2:3 ¶ 11, It is helpful to also send information which are not appropriate in pe… in KII / 2:7 ¶ 17, Yeah, it will have effective change on community in KII / 2:9 ¶ 19, Yeah, previously mothers use the information they get during ANC, but… in KII / 2:11 ¶ 21, Information access, no transportation cost, and no professional resour… in KII / 2:36 ¶ 55, No need of motor or vehicles because it is mHealth. I will be happy to… in KII / 3:6 ¶ 5, They difference for mHealth is mother always get counseling or health… in Group discussion / 3:18 ¶ 12, It looks like mothers are tired of current service provision so the mH… in Group discussion / 3:23 ¶ 14, Previously, people think poor health system and there are many complia… in Group discussion / 3:24 ¶ 14, Over time this can change community perception and they may reebok mot… in Group discussion / 3:25 ¶ 14, There may be emerged women who are successful because of mHealth and t… in Group discussion / 3:27 ¶ 16, It can make the child health growth, for mothers to know danger signs,… in Group discussion / 3:38 ¶ 22, Of course having organized maternal data can also promote contacting a… in Group discussion / 3:41 ¶ 26, : It is a big opportunity to meet the patient again virtually especial… in Group discussion / 3:42 ¶ 26, Traditionally, when errors happen or if the patients go with the wrong… in Group discussion / 3:50 ¶ 33, Benefit for mothers for next pregnancies and healthy prospect for chil… in Group discussion / 3:59 ¶ 45, Satisfaction of their client service and familiarization with technolo… in Group discussion / 3:61 ¶ 47, Planning, decision making based on mHealth, and increased service take… in Group discussion / 3:62 ¶ 48, Support related to mHealth, fulfillment of deficiencies of human and o… in Group discussion / 3:67 ¶ 53, he respect for pregnancy mothers may think gave many births and not ca… in Group discussion

1:98 ¶ 98 in interview analysis

Text quotation

Created by Girma Gilano on 3/9/2023

If all input resources are fulfilled, there will be no problem. It is not a chemical and is not harmful and it is helpful. I don’t see any obstacles because we give them by aware mothers, preparing how mother can read and all other necessary can fulfilling the service needs.

1 Codes:

- **Benefits of mHealth: Effective**

Comment: by Girma Gilano

> *3/12/2023 11:46:46 AM, merged with Effectiviness 3/12/2023 1:12:09 PM, merged with Decision_making 3/12/2023 1:12:55 PM, merged with Role_improvement*

6 Quotations:

1:48 ¶ 55, t can reduce cost, time, and other resources in interview analysis / 1:60 ¶ 64, It will be very effective because it advances the services in interview analysis / 1:61 ¶ 65, I think it will be very effective because we can see the newly impleme… in interview analysis / 1:81 ¶ 80, pe mothers will turn to use mobiles, but only after getting its import… in interview analysis / 1:98 ¶ 98, If all input resources are fulfilled, there will be no problem. It is… in interview analysis / 2:7 ¶ 17, Yeah, it will have effective change on community in KII

2:7 ¶ 17 in KII

Text quotation

Created by Girma Gilano on 3/9/2023

Yeah, it will have effective change on community

3 Codes:

Benefits of mHealth: Effective

Comment: by Girma Gilano

> *3/12/2023 11:46:46 AM, merged with Effectiviness 3/12/2023 1:12:09 PM, merged with Decision_making 3/12/2023 1:12:55 PM, merged with Role_improvement*

6 Quotations:

1:48 ¶ 55, t can reduce cost, time, and other resources in interview analysis / 1:60 ¶ 64, It will be very effective because it advances the services in interview analysis / 1:61 ¶ 65, I think it will be very effective because we can see the newly impleme… in interview analysis / 1:81 ¶ 80, pe mothers will turn to use mobiles, but only after getting its import… in interview analysis / 1:98 ¶ 98, If all input resources are fulfilled, there will be no problem. It is… in interview analysis / 2:7 ¶ 17, Yeah, it will have effective change on community in KII

Benefits of mHealth: Help_mothers

Comment: by Girma Gilano

> *3/12/2023 11:46:46 AM, merged with Effectiviness 3/12/2023 1:12:09 PM, merged with Decision_making 3/12/2023 1:12:55 PM, merged with Role_improvement*

27 Quotations:

1:41 ¶ 47, ion comes after understanding of benefits and harms in interview analysis / 1:45 ¶ 52, Accessibility can be improved mothers get information at their homes in interview analysis / 1:51 ¶ 58, She can access any information in interview analysis / 1:53 ¶ 59, I think mHealth can improve access, appointment on time, availability… in interview analysis / 1:65 ¶ 67, Her husband or children will also push her to go even if the condition… in interview analysis / 1:68 ¶ 70, Every system is getting digitalized so digitalizing health may have mu… in interview analysis / 1:77 ¶ 77, Rural mothers are respectful; they do what they told to do, so fear of… in interview analysis / 1:81 ¶ 80, pe mothers will turn to use mobiles, but only after getting its import… in interview analysis / 2:3 ¶ 11, It is helpful to also send information which are not appropriate in pe… in KII / 2:7 ¶ 17, Yeah, it will have effective change on community in KII / 2:9 ¶ 19, Yeah, previously mothers use the information they get during ANC, but… in KII / 2:11 ¶ 21, Information access, no transportation cost, and no professional resour… in KII / 2:36 ¶ 55, No need of motor or vehicles because it is mHealth. I will be happy to… in KII / 3:6 ¶ 5, They difference for mHealth is mother always get counseling or health… in Group discussion / 3:18 ¶ 12, It looks like mothers are tired of current service provision so the mH… in Group discussion / 3:23 ¶ 14, Previously, people think poor health system and there are many complia… in Group discussion / 3:24 ¶ 14, Over time this can change community perception and they may reebok mot… in Group discussion / 3:25 ¶ 14, There may be emerged women who are successful because of mHealth and t… in Group discussion / 3:27 ¶ 16, It can make the child health growth, for mothers to know danger signs,… in Group discussion / 3:38 ¶ 22, Of course having organized maternal data can also promote contacting a… in Group discussion / 3:41 ¶ 26, : It is a big opportunity to meet the patient again virtually especial… in Group discussion / 3:42 ¶ 26, Traditionally, when errors happen or if the patients go with the wrong… in Group discussion / 3:50 ¶ 33, Benefit for mothers for next pregnancies and healthy prospect for chil… in Group discussion / 3:59 ¶ 45, Satisfaction of their client service and familiarization with technolo… in Group discussion / 3:61 ¶ 47, Planning, decision making based on mHealth, and increased service take… in Group discussion / 3:62 ¶ 48, Support related to mHealth, fulfillment of deficiencies of human and o… in Group discussion / 3:67 ¶ 53, he respect for pregnancy mothers may think gave many births and not ca… in Group discussion

Benefits of mHealth: Help_professional

Comment: by Girma Gilano

3/12/2023 11:46:46 AM, merged with Effectiviness 3/12/2023 1:12:09 PM, merged with Decision_making 3/12/2023 1:12:55 PM, merged with Role_improvement

30 Quotations:

1:24 ¶ 34, We always give best service to our best capacity, but mHealth may adva… in interview analysis / 1:34 ¶ 39, As HEWs we cannot reach for every mother on time but text can easily r… in interview analysis / 1:36 ¶ 40, This mHealth better than our previous our service in interview analysis / 1:50 ¶ 57, further improve the already improving service in interview analysis / 1:56 ¶ 62, Our current ANC and other formats now request mother to register their… in interview analysis / 1:68 ¶ 70, Every system is getting digitalized so digitalizing health may have mu… in interview analysis / 1:82 ¶ 81, ers usually take what we tell them, it will be successful. I don't thi… in interview analysis / 2:3 ¶ 11, It is helpful to also send information which are not appropriate in pe… in KII / 2:7 ¶ 17, Yeah, it will have effective change on community in KII / 2:9 ¶ 19, Yeah, previously mothers use the information they get during ANC, but… in KII / 2:35 ¶ 54, Since we have many gaps this should be seen as an opportunity in KII / 3:2 ¶ 3, Previous teaching of mother through home to home is going to be throug… in Group discussion / 3:12 ¶ 8, For educated mothers messages will be option less in Group discussion / 3:13 ¶ 8, Starting from me we feed additional food at 2months because the kind o… in Group discussion / 3:18 ¶ 12, It looks like mothers are tired of current service provision so the mH… in Group discussion / 3:25 ¶ 14, There may be emerged women who are successful because of mHealth and t… in Group discussion / 3:29 ¶ 17, As a health professionals applying technology can only easy our burden… in Group discussion / 3:34 ¶ 19, ecause of mHealth we may every address of women and now we can also ca… in Group discussion / 3:35 ¶ 20, We will provide the service for mothers we know now, but previously we… in Group discussion / 3:38 ¶ 22, Of course having organized maternal data can also promote contacting a… in Group discussion / 3:41 ¶ 26, : It is a big opportunity to meet the patient again virtually especial… in Group discussion / 3:42 ¶ 26, Traditionally, when errors happen or if the patients go with the wrong… in Group discussion / 3:43 ¶ 28, At the end this is very important piece of technology that can reduce… in Group discussion / 3:46 ¶ 28, Those infrastructure and awareness creation in staffs, mothers, and co… in Group discussion / 3:52 ¶ 36, People will enjoy talking to others how health institution just concer… in Group discussion / 3:57 ¶ 42, Improve work environment as per the need of the technology and owning… in Group discussion / 3:58 ¶ 43, Aspiring more technologies and helping mothers, taking trainings, givi… in Group discussion / 3:60 ¶ 46, Experience with technology. Enable them to identify areas where mHealt… in Group discussion / 3:61 ¶ 47, Planning, decision making based on mHealth, and increased service take… in Group discussion / 3:62 ¶ 48, Support related to mHealth, fulfillment of deficiencies of human and o… in Group discussion

---

● Benefits of mHealth: Help_mothers

Created by Girma Gilano on 3/13/2023

Comment: by Girma Gilano

3/12/2023 11:46:46 AM, merged with Effectiviness 3/12/2023 1:12:09 PM, merged with Decision_making 3/12/2023 1:12:55 PM, merged with Role_improvement

27 Quotations:

1:41 ¶ 47 in interview analysis

Text quotation

Created by Girma Gilano on 3/8/2023

ion comes after understanding of benefits and harms

1 Codes:

- **Benefits of mHealth: Help_mothers**

  Comment: by Girma Gilano

  > *3/12/2023 11:46:46 AM, merged with Effectiviness 3/12/2023 1:12:09 PM, merged with Decision_making 3/12/2023 1:12:55 PM, merged with Role_improvement*

  #### 27 Quotations:

  1:41 ¶ 47, ion comes after understanding of benefits and harms in interview analysis / 1:45 ¶ 52, Accessibility can be improved mothers get information at their homes in interview analysis / 1:51 ¶ 58, She can access any information in interview analysis / 1:53 ¶ 59, I think mHealth can improve access, appointment on time, availability… in interview analysis / 1:65 ¶ 67, Her husband or children will also push her to go even if the condition… in interview analysis / 1:68 ¶ 70, Every system is getting digitalized so digitalizing health may have mu… in interview analysis / 1:77 ¶ 77, Rural mothers are respectful; they do what they told to do, so fear of… in interview analysis / 1:81 ¶ 80, pe mothers will turn to use mobiles, but only after getting its import… in interview analysis / 2:3 ¶ 11, It is helpful to also send information which are not appropriate in pe… in KII / 2:7 ¶ 17, Yeah, it will have effective change on community in KII / 2:9 ¶ 19, Yeah, previously mothers use the information they get during ANC, but… in KII / 2:11 ¶ 21, Information access, no transportation cost, and no professional resour… in KII / 2:36 ¶ 55, No need of motor or vehicles because it is mHealth. I will be happy to… in KII / 3:6 ¶ 5, They difference for mHealth is mother always get counseling or health… in Group discussion / 3:18 ¶ 12, It looks like mothers are tired of current service provision so the mH… in Group discussion / 3:23 ¶ 14, Previously, people think poor health system and there are many complia… in Group discussion / 3:24 ¶ 14, Over time this can change community perception and they may reebok mot… in Group discussion / 3:25 ¶ 14, There may be emerged women who are successful because of mHealth and t… in Group discussion / 3:27 ¶ 16, It can make the child health growth, for mothers to know danger signs,… in Group discussion / 3:38 ¶ 22, Of course having organized maternal data can also promote contacting a… in Group discussion / 3:41 ¶ 26, : It is a big opportunity to meet the patient again virtually especial… in Group discussion / 3:42 ¶ 26, Traditionally, when errors happen or if the patients go with the wrong… in Group discussion / 3:50 ¶ 33, Benefit for mothers for next pregnancies and healthy prospect for chil… in Group discussion / 3:59 ¶ 45, Satisfaction of their client service and familiarization with technolo… in Group discussion / 3:61 ¶ 47, Planning, decision making based on mHealth, and increased service take… in Group discussion / 3:62 ¶ 48, Support related to mHealth, fulfillment of deficiencies of human and o… in Group discussion / 3:67 ¶ 53, he respect for pregnancy mothers may think gave many births and not ca… in Group discussion

1:45 ¶ 52 in interview analysis

Text quotation

Created by Girma Gilano on 3/8/2023

Accessibility can be improved mothers get information at their homes

1 Codes:

- **Benefits of mHealth: Help_mothers**

  Comment: by Girma Gilano

  > *3/12/2023 11:46:46 AM, merged with Effectiviness 3/12/2023 1:12:09 PM, merged with Decision_making 3/12/2023 1:12:55 PM, merged with Role_improvement*

  #### 27 Quotations:

  1:41 ¶ 47, ion comes after understanding of benefits and harms in interview analysis / 1:45 ¶ 52, Accessibility can be improved mothers get information at their homes in interview analysis / 1:51 ¶ 58,

She can access any information in interview analysis / 1:53 ¶ 59, I think mHealth can improve access, appointment on time, availability… in interview analysis / 1:65 ¶ 67, Her husband or children will also push her to go even if the condition… in interview analysis / 1:68 ¶ 70, Every system is getting digitalized so digitalizing health may have mu… in interview analysis / 1:77 ¶ 77, Rural mothers are respectful; they do what they told to do, so fear of… in interview analysis / 1:81 ¶ 80, pe mothers will turn to use mobiles, but only after getting its import… in interview analysis / 2:3 ¶ 11, It is helpful to also send information which are not appropriate in pe… in KII / 2:7 ¶ 17, Yeah, it will have effective change on community in KII / 2:9 ¶ 19, Yeah, previously mothers use the information they get during ANC, but… in KII / 2:11 ¶ 21, Information access, no transportation cost, and no professional resour… in KII / 2:36 ¶ 55, No need of motor or vehicles because it is mHealth. I will be happy to… in KII / 3:6 ¶ 5, They difference for mHealth is mother always get counseling or health… in Group discussion / 3:18 ¶ 12, It looks like mothers are tired of current service provision so the mH… in Group discussion / 3:23 ¶ 14, Previously, people think poor health system and there are many complia… in Group discussion / 3:24 ¶ 14, Over time this can change community perception and they may reebok mot… in Group discussion / 3:25 ¶ 14, There may be emerged women who are successful because of mHealth and t… in Group discussion / 3:27 ¶ 16, It can make the child health growth, for mothers to know danger signs,… in Group discussion / 3:38 ¶ 22, Of course having organized maternal data can also promote contacting a… in Group discussion / 3:41 ¶ 26, : It is a big opportunity to meet the patient again virtually especial… in Group discussion / 3:42 ¶ 26, Traditionally, when errors happen or if the patients go with the wrong… in Group discussion / 3:50 ¶ 33, Benefit for mothers for next pregnancies and healthy prospect for chil… in Group discussion / 3:59 ¶ 45, Satisfaction of their client service and familiarization with technolo… in Group discussion / 3:61 ¶ 47, Planning, decision making based on mHealth, and increased service take… in Group discussion / 3:62 ¶ 48, Support related to mHealth, fulfillment of deficiencies of human and o… in Group discussion / 3:67 ¶ 53, he respect for pregnancy mothers may think gave many births and not ca… in Group discussion

1:51 ¶ 58 in interview analysis

Text quotation

Created by Girma Gilano on 3/8/2023

She can access any information

1 Codes:

- **Benefits of mHealth: Help_mothers**

Comment: by Girma Gilano

3/12/2023 11:46:46 AM, merged with Effectiviness 3/12/2023 1:12:09 PM, merged with Decision_making 3/12/2023 1:12:55 PM, merged with Role_improvement

27 Quotations:

1:41 ¶ 47, ion comes after understanding of benefits and harms in interview analysis / 1:45 ¶ 52, Accessibility can be improved mothers get information at their homes in interview analysis / 1:51 ¶ 58, She can access any information in interview analysis / 1:53 ¶ 59, I think mHealth can improve access, appointment on time, availability… in interview analysis / 1:65 ¶ 67, Her husband or children will also push her to go even if the condition… in interview analysis / 1:68 ¶ 70, Every system is getting digitalized so digitalizing health may have mu… in interview analysis / 1:77 ¶ 77, Rural mothers are respectful; they do what they told to do, so fear of… in interview analysis / 1:81 ¶ 80, pe mothers will turn to use mobiles, but only after getting its import… in interview analysis / 2:3 ¶ 11, It is helpful to also send information which are not appropriate in pe… in KII / 2:7 ¶ 17, Yeah, it will have effective change on community in KII / 2:9 ¶ 19, Yeah, previously mothers use the information they get during ANC, but… in KII / 2:11 ¶ 21, Information access, no transportation cost, and no professional resour… in KII / 2:36 ¶ 55, No need of motor or vehicles because it is mHealth. I will be happy to… in KII / 3:6 ¶ 5, They difference for mHealth is mother always get counseling or health… in Group discussion / 3:18 ¶ 12, It looks like mothers are tired of current service provision so the mH… in Group discussion / 3:23 ¶ 14, Previously, people think poor health system and there are many complia… in Group discussion / 3:24 ¶ 14, Over time this can

change community perception and they may reebok mot… in Group discussion / 3:25 ¶ 14, There may be emerged women who are successful because of mHealth and t… in Group discussion / 3:27 ¶ 16, It can make the child health growth, for mothers to know danger signs,… in Group discussion / 3:38 ¶ 22, Of course having organized maternal data can also promote contacting a… in Group discussion / 3:41 ¶ 26, : It is a big opportunity to meet the patient again virtually especial… in Group discussion / 3:42 ¶ 26, Traditionally, when errors happen or if the patients go with the wrong… in Group discussion / 3:50 ¶ 33, Benefit for mothers for next pregnancies and healthy prospect for chil… in Group discussion / 3:59 ¶ 45, Satisfaction of their client service and familiarization with technolo… in Group discussion / 3:61 ¶ 47, Planning, decision making based on mHealth, and increased service take… in Group discussion / 3:62 ¶ 48, Support related to mHealth, fulfillment of deficiencies of human and o… in Group discussion / 3:67 ¶ 53, he respect for pregnancy mothers may think gave many births and not ca… in Group discussion

1:53 ¶ 59 in interview analysis

Text quotation

Created by Girma Gilano on 3/8/2023

I think mHealth can improve access, appointment on time, availability of information for decision making increased

2 Codes:

- **Benefits of mHealth: ALarming**

Comment: by Girma Gilano

> *3/12/2023 11:46:46 AM, merged with Effectiviness 3/12/2023 1:12:09 PM, merged with Decision_making 3/12/2023 1:12:55 PM, merged with Role_improvement*

6 Quotations:

1:10 ¶ 24, it helps when people are busy with their work to remember appointments in interview analysis / 1:25 ¶ 35, Mothers may not miss their appointment because they forget i in interview analysis / 1:53 ¶ 59, I think mHealth can improve access, appointment on time, availability… in interview analysis / 1:68 ¶ 70, Every system is getting digitalized so digitalizing health may have mu… in interview analysis / 3:27 ¶ 16, It can make the child health growth, for mothers to know danger signs,… in Group discussion / 3:47 ¶ 30, Appointment reminder, service on time, and reduce mortality, but befor… in Group discussion

- **Benefits of mHealth: Help_mothers**

Comment: by Girma Gilano

> *3/12/2023 11:46:46 AM, merged with Effectiviness 3/12/2023 1:12:09 PM, merged with Decision_making 3/12/2023 1:12:55 PM, merged with Role_improvement*

27 Quotations:

1:41 ¶ 47, ion comes after understanding of benefits and harms in interview analysis / 1:45 ¶ 52, Accessibility can be improved mothers get information at their homes in interview analysis / 1:51 ¶ 58, She can access any information in interview analysis / 1:53 ¶ 59, I think mHealth can improve access, appointment on time, availability… in interview analysis / 1:65 ¶ 67, Her husband or children will also push her to go even if the condition… in interview analysis / 1:68 ¶ 70, Every system is getting digitalized so digitalizing health may have mu… in interview analysis / 1:77 ¶ 77, Rural mothers are respectful; they do what they told to do, so fear of… in interview analysis / 1:81 ¶ 80, pe mothers will turn to use mobiles, but only after getting its import… in interview analysis / 2:3 ¶ 11, It is helpful to also send information which are not appropriate in pe… in KII / 2:7 ¶ 17, Yeah, it will have effective change on community in KII / 2:9 ¶ 19, Yeah, previously mothers use the information they get during ANC, but… in KII / 2:11 ¶ 21, Information access, no transportation cost, and no professional resour… in KII / 2:36 ¶ 55, No need of

motor or vehicles because it is mHealth. I will be happy to… in KII / 3:6 ¶ 5, They difference for mHealth is mother always get counseling or health… in Group discussion / 3:18 ¶ 12, It looks like mothers are tired of current service provision so the mH… in Group discussion / 3:23 ¶ 14, Previously, people think poor health system and there are many complia… in Group discussion / 3:24 ¶ 14, Over time this can change community perception and they may reebok mot… in Group discussion / 3:25 ¶ 14, There may be emerged women who are successful because of mHealth and t… in Group discussion / 3:27 ¶ 16, It can make the child health growth, for mothers to know danger signs,… in Group discussion / 3:38 ¶ 22, Of course having organized maternal data can also promote contacting a… in Group discussion / 3:41 ¶ 26, : It is a big opportunity to meet the patient again virtually especial… in Group discussion / 3:42 ¶ 26, Traditionally, when errors happen or if the patients go with the wrong… in Group discussion / 3:50 ¶ 33, Benefit for mothers for next pregnancies and healthy prospect for chil… in Group discussion / 3:59 ¶ 45, Satisfaction of their client service and familiarization with technolo… in Group discussion / 3:61 ¶ 47, Planning, decision making based on mHealth, and increased service take… in Group discussion / 3:62 ¶ 48, Support related to mHealth, fulfillment of deficiencies of human and o… in Group discussion / 3:67 ¶ 53, he respect for pregnancy mothers may think gave many births and not ca… in Group discussion

1:65 ¶ 67 in interview analysis

Text quotation

Created by Girma Gilano on 3/8/2023

Her husband or children will also push her to go even if the condition may make the on a different day

1 Codes:

- **Benefits of mHealth: Help_mothers**

Comment: by Girma Gilano

> *3/12/2023 11:46:46 AM, merged with Effectiviness 3/12/2023 1:12:09 PM, merged with Decision_making 3/12/2023 1:12:55 PM, merged with Role_improvement*

27 Quotations:

1:41 ¶ 47, ion comes after understanding of benefits and harms in interview analysis / 1:45 ¶ 52, Accessibility can be improved mothers get information at their homes in interview analysis / 1:51 ¶ 58, She can access any information in interview analysis / 1:53 ¶ 59, I think mHealth can improve access, appointment on time, availability… in interview analysis / 1:65 ¶ 67, Her husband or children will also push her to go even if the condition… in interview analysis / 1:68 ¶ 70, Every system is getting digitalized so digitalizing health may have mu… in interview analysis / 1:77 ¶ 77, Rural mothers are respectful; they do what they told to do, so fear of… in interview analysis / 1:81 ¶ 80, pe mothers will turn to use mobiles, but only after getting its import… in interview analysis / 2:3 ¶ 11, It is helpful to also send information which are not appropriate in pe… in KII / 2:7 ¶ 17, Yeah, it will have effective change on community in KII / 2:9 ¶ 19, Yeah, previously mothers use the information they get during ANC, but… in KII / 2:11 ¶ 21, Information access, no transportation cost, and no professional resour… in KII / 2:36 ¶ 55, No need of motor or vehicles because it is mHealth. I will be happy to… in KII / 3:6 ¶ 5, They difference for mHealth is mother always get counseling or health… in Group discussion / 3:18 ¶ 12, It looks like mothers are tired of current service provision so the mH… in Group discussion / 3:23 ¶ 14, Previously, people think poor health system and there are many complia… in Group discussion / 3:24 ¶ 14, Over time this can change community perception and they may reebok mot… in Group discussion / 3:25 ¶ 14, There may be emerged women who are successful because of mHealth and t… in Group discussion / 3:27 ¶ 16, It can make the child health growth, for mothers to know danger signs,… in Group discussion / 3:38 ¶ 22, Of course having organized maternal data can also promote contacting a… in Group discussion / 3:41 ¶ 26, : It is a big opportunity to meet the patient again virtually especial… in Group discussion / 3:42 ¶ 26, Traditionally, when errors happen or if the patients go with the wrong… in Group discussion / 3:50 ¶ 33, Benefit for mothers for next pregnancies and healthy prospect for chil… in Group discussion / 3:59 ¶ 45, Satisfaction of their client service and familiarization with technolo… in Group discussion / 3:61 ¶ 47,

Planning, decision making based on mHealth, and increased service take… in Group discussion / 3:62 ¶ 48, Support related to mHealth, fulfillment of deficiencies of human and o… in Group discussion / 3:67 ¶ 53, he respect for pregnancy mothers may think gave many births and not ca… in Group discussion

1:68 ¶ 70 in interview analysis

Text quotation

Created by Girma Gilano on 3/8/2023

Every system is getting digitalized so digitalizing health may have much advantage more than just appointme

3 Codes:

Benefits of mHealth: ALarming

Comment: by Girma Gilano

> *3/12/2023 11:46:46 AM, merged with Effectiviness 3/12/2023 1:12:09 PM, merged with Decision_making 3/12/2023 1:12:55 PM, merged with Role_improvement*

6 Quotations:

1:10 ¶ 24, it helps when people are busy with their work to remember appointments in interview analysis / 1:25 ¶ 35, Mothers may not miss their appointment because they forget i in interview analysis / 1:53 ¶ 59, I think mHealth can improve access, appointment on time, availability… in interview analysis / 1:68 ¶ 70, Every system is getting digitalized so digitalizing health may have mu… in interview analysis / 3:27 ¶ 16, It can make the child health growth, for mothers to know danger signs,… in Group discussion / 3:47 ¶ 30, Appointment reminder, service on time, and reduce mortality, but befor… in Group discussion

Benefits of mHealth: Help_mothers

Comment: by Girma Gilano

> *3/12/2023 11:46:46 AM, merged with Effectiviness 3/12/2023 1:12:09 PM, merged with Decision_making 3/12/2023 1:12:55 PM, merged with Role_improvement*

27 Quotations:

1:41 ¶ 47, ion comes after understanding of benefits and harms in interview analysis / 1:45 ¶ 52, Accessibility can be improved mothers get information at their homes in interview analysis / 1:51 ¶ 58, She can access any information in interview analysis / 1:53 ¶ 59, I think mHealth can improve access, appointment on time, availability… in interview analysis / 1:65 ¶ 67, Her husband or children will also push her to go even if the condition… in interview analysis / 1:68 ¶ 70, Every system is getting digitalized so digitalizing health may have mu… in interview analysis / 1:77 ¶ 77, Rural mothers are respectful; they do what they told to do, so fear of… in interview analysis / 1:81 ¶ 80, pe mothers will turn to use mobiles, but only after getting its import… in interview analysis / 2:3 ¶ 11, It is helpful to also send information which are not appropriate in pe… in KII / 2:7 ¶ 17, Yeah, it will have effective change on community in KII / 2:9 ¶ 19, Yeah, previously mothers use the information they get during ANC, but… in KII / 2:11 ¶ 21, Information access, no transportation cost, and no professional resour… in KII / 2:36 ¶ 55, No need of motor or vehicles because it is mHealth. I will be happy to… in KII / 3:6 ¶ 5, They difference for mHealth is mother always get counseling or health… in Group discussion / 3:18 ¶ 12, It looks like mothers are tired of current service provision so the mH… in Group discussion / 3:23 ¶ 14, Previously, people think poor health system and there are many complia… in Group discussion / 3:24 ¶ 14, Over time this can change community perception and they may reebok mot… in Group discussion / 3:25 ¶ 14, There may be emerged women who are successful because of mHealth and t… in Group discussion / 3:27 ¶ 16, It can make the child health growth, for mothers to know danger signs,… in Group discussion / 3:38 ¶ 22, Of course having organized maternal data can also promote contacting a… in Group discussion / 3:41 ¶

26, : It is a big opportunity to meet the patient again virtually especial… in Group discussion / 3:42 ¶ 26, Traditionally, when errors happen or if the patients go with the wrong… in Group discussion / 3:50 ¶ 33, Benefit for mothers for next pregnancies and healthy prospect for chil… in Group discussion / 3:59 ¶ 45, Satisfaction of their client service and familiarization with technolo… in Group discussion / 3:61 ¶ 47, Planning, decision making based on mHealth, and increased service take… in Group discussion / 3:62 ¶ 48, Support related to mHealth, fulfillment of deficiencies of human and o… in Group discussion / 3:67 ¶ 53, he respect for pregnancy mothers may think gave many births and not ca… in Group discussion

Benefits of mHealth: Help_professional

Comment: by Girma Gilano

> *3/12/2023 11:46:46 AM, merged with Effectiviness 3/12/2023 1:12:09 PM, merged with Decision_making 3/12/2023 1:12:55 PM, merged with Role_improvement*

30 Quotations:

1:24 ¶ 34, We always give best service to our best capacity, but mHealth may adva… in interview analysis / 1:34 ¶ 39, As HEWs we cannot reach for every mother on time but text can easily r… in interview analysis / 1:36 ¶ 40, This mHealth better than our previous our service in interview analysis / 1:50 ¶ 57, further improve the already improving service in interview analysis / 1:56 ¶ 62, Our current ANC and other formats now request mother to register their… in interview analysis / 1:68 ¶ 70, Every system is getting digitalized so digitalizing health may have mu… in interview analysis / 1:82 ¶ 81, ers usually take what we tell them, it will be successful. I don’t thi… in interview analysis / 2:3 ¶ 11, It is helpful to also send information which are not appropriate in pe… in KII / 2:7 ¶ 17, Yeah, it will have effective change on community in KII / 2:9 ¶ 19, Yeah, previously mothers use the information they get during ANC, but… in KII / 2:35 ¶ 54, Since we have many gaps this should be seen as an opportunity in KII / 3:2 ¶ 3, Previous teaching of mother through home to home is going to be throug… in Group discussion / 3:12 ¶ 8, For educated mothers messages will be option less in Group discussion / 3:13 ¶ 8, Starting from me we feed additional food at 2months because the kind o… in Group discussion / 3:18 ¶ 12, It looks like mothers are tired of current service provision so the mH… in Group discussion / 3:25 ¶ 14, There may be emerged women who are successful because of mHealth and t… in Group discussion / 3:29 ¶ 17, As a health professionals applying technology can only easy our burden… in Group discussion / 3:34 ¶ 19, ecause of mHealth we may every address of women and now we can also ca… in Group discussion / 3:35 ¶ 20, We will provide the service for mothers we know now, but previously we… in Group discussion / 3:38 ¶ 22, Of course having organized maternal data can also promote contacting a… in Group discussion / 3:41 ¶ 26, : It is a big opportunity to meet the patient again virtually especial… in Group discussion / 3:42 ¶ 26, Traditionally, when errors happen or if the patients go with the wrong… in Group discussion / 3:43 ¶ 28, At the end this is very important piece of technology that can reduce… in Group discussion / 3:46 ¶ 28, Those infrastructure and awareness creation in staffs, mothers, and co… in Group discussion / 3:52 ¶ 36, People will enjoy talking to others how health institution just concer… in Group discussion / 3:57 ¶ 42, Improve work environment as per the need of the technology and owning… in Group discussion / 3:58 ¶ 43, Aspiring more technologies and helping mothers, taking trainings, givi… in Group discussion / 3:60 ¶ 46, Experience with technology. Enable them to identify areas where mHealt… in Group discussion / 3:61 ¶ 47, Planning, decision making based on mHealth, and increased service take… in Group discussion / 3:62 ¶ 48, Support related to mHealth, fulfillment of deficiencies of human and o… in Group discussion

1:77 ¶ 77 in interview analysis

Text quotation

Created by Girma Gilano on 3/8/2023, **modified** by Girma Gilano on 3/8/2023

Rural mothers are respectful; they do what they told to do, so fear of poor success is not a problem. Technology is not a new. Mobile is everywhere; messaging common so I don’t there will be a problem. Teaching mothers will be important

2 Codes:

Benefits of mHealth: Help_mothers

Comment: by Girma Gilano

> *3/12/2023 11:46:46 AM, merged with Effectiviness 3/12/2023 1:12:09 PM, merged with Decision_making 3/12/2023 1:12:55 PM, merged with Role_improvement*

27 Quotations:

1:41 ¶ 47, ion comes after understanding of benefits and harms in interview analysis / 1:45 ¶ 52, Accessibility can be improved mothers get information at their homes in interview analysis / 1:51 ¶ 58, She can access any information in interview analysis / 1:53 ¶ 59, I think mHealth can improve access, appointment on time, availability… in interview analysis / 1:65 ¶ 67, Her husband or children will also push her to go even if the condition… in interview analysis / 1:68 ¶ 70, Every system is getting digitalized so digitalizing health may have mu… in interview analysis / 1:77 ¶ 77, Rural mothers are respectful; they do what they told to do, so fear of… in interview analysis / 1:81 ¶ 80, pe mothers will turn to use mobiles, but only after getting its import… in interview analysis / 2:3 ¶ 11, It is helpful to also send information which are not appropriate in pe… in KII / 2:7 ¶ 17, Yeah, it will have effective change on community in KII / 2:9 ¶ 19, Yeah, previously mothers use the information they get during ANC, but… in KII / 2:11 ¶ 21, Information access, no transportation cost, and no professional resour… in KII / 2:36 ¶ 55, No need of motor or vehicles because it is mHealth. I will be happy to… in KII / 3:6 ¶ 5, They difference for mHealth is mother always get counseling or health… in Group discussion / 3:18 ¶ 12, It looks like mothers are tired of current service provision so the mH… in Group discussion / 3:23 ¶ 14, Previously, people think poor health system and there are many complia… in Group discussion / 3:24 ¶ 14, Over time this can change community perception and they may reebok mot… in Group discussion / 3:25 ¶ 14, There may be emerged women who are successful because of mHealth and t… in Group discussion / 3:27 ¶ 16, It can make the child health growth, for mothers to know danger signs,… in Group discussion / 3:38 ¶ 22, Of course having organized maternal data can also promote contacting a… in Group discussion / 3:41 ¶ 26, : It is a big opportunity to meet the patient again virtually especial… in Group discussion / 3:42 ¶ 26, Traditionally, when errors happen or if the patients go with the wrong… in Group discussion / 3:50 ¶ 33, Benefit for mothers for next pregnancies and healthy prospect for chil… in Group discussion / 3:59 ¶ 45, Satisfaction of their client service and familiarization with technolo… in Group discussion / 3:61 ¶ 47, Planning, decision making based on mHealth, and increased service take… in Group discussion / 3:62 ¶ 48, Support related to mHealth, fulfillment of deficiencies of human and o… in Group discussion / 3:67 ¶ 53, he respect for pregnancy mothers may think gave many births and not ca… in Group discussion

Benefits of mHealth: Improve_MCH

Comment: by Girma Gilano

> *3/12/2023 11:46:46 AM, merged with Effectiviness 3/12/2023 1:12:09 PM, merged with Decision_making 3/12/2023 1:12:55 PM, merged with Role_improvement*

25 Quotations:

1:6 ¶ 22, Non-use of the service because of negligence and forgetting can be im… in interview analysis / 1:26 ¶ 35, t can increase utilization better than that we have previously in interview analysis / 1:28 ¶ 36, Even since this digital it can further improve the service and increas… in interview analysis / 1:36 ¶ 40, This mHealth better than our previous our service in interview analysis / 1:43 ¶ 50, For maternal and child health the decision to take service is mothers… in interview analysis / 1:49 ¶ 56, Having information on time can improve the health of mothers and child… in interview analysis / 1:50 ¶ 57, further improve the already improving service in interview analysis / 1:57 ¶ 63, f it continue, it can be effective and be important than previous way… in interview analysis / 1:59 ¶ 64, I think whatever we put in to the community to improve health service… in interview analysis / 1:62 ¶ 66, This can improve and put our usual service one step forward in interview analysis / 1:74 ¶ 75, I don't think people can be affected negatively because they love to l… in interview analysis / 1:77 ¶ 77, Rural mothers are respectful; they do what they told to do, so fear of… in interview analysis / 1:78 ¶ 78, One thing I assure is it will increase up taking maternal and child he… in interview analysis / 1:83 ¶ 83, It can remind which is especially important for family planning. Witho… in interview analysis / 2:4 ¶ 12, If health system organized this way it will be helpful in KII / 2:6 ¶ 16, this is the main thing to improve maternal and child care especially u… in KII / 3:8 ¶ 6, After counseling and aware mother it can be improved in Group discussion / 3:9 ¶ 8, It

can improve those listed service (ANC, PNC, Breastfeeding and vacci… in Group discussion / 3:10 ¶ 8, Mother are already exposed to health education, but this one is direct… in Group discussion / 3:18 ¶ 12, It looks like mothers are tired of current service provision so the mH… in Group discussion / 3:19 ¶ 12, So in that case it can completely change the stream and interest. Or t… in Group discussion / 3:20 ¶ 12, They may even consider the increased risk of danger related to pregnan… in Group discussion / 3:43 ¶ 28, At the end this is very important piece of technology that can reduce… in Group discussion / 3:48 ¶ 31, Service at their home and Information and on time vaccination and Heal… in Group discussion / 3:49 ¶ 32, Healthy child feeding and behavioral change for mother that wll be lon… in Group discussion

1:81 ¶ 80 in interview analysis

Text quotation

Created by Girma Gilano on 3/8/2023

pe mothers will turn to use mobiles, but only after getting its importance that why health education should be crucial part of this implementation

2 Codes:

- **Benefits of mHealth: Effective**

Comment: by Girma Gilano

> *3/12/2023 11:46:46 AM, merged with Effectiviness 3/12/2023 1:12:09 PM, merged with Decision_making 3/12/2023 1:12:55 PM, merged with Role_improvement*

6 Quotations:

1:48 ¶ 55, t can reduce cost, time, and other resources in interview analysis / 1:60 ¶ 64, It will be very effective because it advances the services in interview analysis / 1:61 ¶ 65, I think it will be very effective because we can see the newly impleme… in interview analysis / 1:81 ¶ 80, pe mothers will turn to use mobiles, but only after getting its import… in interview analysis / 1:98 ¶ 98, If all input resources are fulfilled, there will be no problem. It is… in interview analysis / 2:7 ¶ 17, Yeah, it will have effective change on community in KII

- **Benefits of mHealth: Help_mothers**

Comment: by Girma Gilano

> *3/12/2023 11:46:46 AM, merged with Effectiviness 3/12/2023 1:12:09 PM, merged with Decision_making 3/12/2023 1:12:55 PM, merged with Role_improvement*

27 Quotations:

1:41 ¶ 47, ion comes after understanding of benefits and harms in interview analysis / 1:45 ¶ 52, Accessibility can be improved mothers get information at their homes in interview analysis / 1:51 ¶ 58, She can access any information in interview analysis / 1:53 ¶ 59, I think mHealth can improve access, appointment on time, availability… in interview analysis / 1:65 ¶ 67, Her husband or children will also push her to go even if the condition… in interview analysis / 1:68 ¶ 70, Every system is getting digitalized so digitalizing health may have mu… in interview analysis / 1:77 ¶ 77, Rural mothers are respectful; they do what they told to do, so fear of… in interview analysis / 1:81 ¶ 80, pe mothers will turn to use mobiles, but only after getting its import… in interview analysis / 2:3 ¶ 11, It is helpful to also send information which are not appropriate in pe… in KII / 2:7 ¶ 17, Yeah, it will have effective change on community in KII / 2:9 ¶ 19, Yeah, previously mothers use the information they get during ANC, but… in KII / 2:11 ¶ 21, Information access, no transportation cost, and no professional resour… in KII / 2:36 ¶ 55, No need of motor or vehicles because it is mHealth. I will be happy to… in KII / 3:6 ¶ 5, They difference for mHealth is mother always get counseling or health… in Group discussion / 3:18 ¶ 12, It looks like mothers are tired of current service provision so the mH… in Group discussion / 3:23 ¶ 14, Previously, people think

poor health system and there are many complia… in Group discussion / 3:24 ¶ 14, Over time this can change community perception and they may reebok mot… in Group discussion / 3:25 ¶ 14, There may be emerged women who are successful because of mHealth and t… in Group discussion / 3:27 ¶ 16, It can make the child health growth, for mothers to know danger signs,… in Group discussion / 3:38 ¶ 22, Of course having organized maternal data can also promote contacting a… in Group discussion / 3:41 ¶ 26, : It is a big opportunity to meet the patient again virtually especial… in Group discussion / 3:42 ¶ 26, Traditionally, when errors happen or if the patients go with the wrong… in Group discussion / 3:50 ¶ 33, Benefit for mothers for next pregnancies and healthy prospect for chil… in Group discussion / 3:59 ¶ 45, Satisfaction of their client service and familiarization with technolo… in Group discussion / 3:61 ¶ 47, Planning, decision making based on mHealth, and increased service take… in Group discussion / 3:62 ¶ 48, Support related to mHealth, fulfillment of deficiencies of human and o… in Group discussion / 3:67 ¶ 53, he respect for pregnancy mothers may think gave many births and not ca… in Group discussion

2:3 ¶ 11 in KII

Text quotation

Created by Girma Gilano on 3/9/2023

It is helpful to also send information which are not appropriate in person

2 Codes:

● Benefits of mHealth: Help_mothers

Comment: by Girma Gilano

> *3/12/2023 11:46:46 AM, merged with Effectiviness 3/12/2023 1:12:09 PM, merged with Decision_making 3/12/2023 1:12:55 PM, merged with Role_improvement*

27 Quotations:

1:41 ¶ 47, ion comes after understanding of benefits and harms in interview analysis / 1:45 ¶ 52, Accessibility can be improved mothers get information at their homes in interview analysis / 1:51 ¶ 58, She can access any information in interview analysis / 1:53 ¶ 59, I think mHealth can improve access, appointment on time, availability… in interview analysis / 1:65 ¶ 67, Her husband or children will also push her to go even if the condition… in interview analysis / 1:68 ¶ 70, Every system is getting digitalized so digitalizing health may have mu… in interview analysis / 1:77 ¶ 77, Rural mothers are respectful; they do what they told to do, so fear of… in interview analysis / 1:81 ¶ 80, pe mothers will turn to use mobiles, but only after getting its import… in interview analysis / 2:3 ¶ 11, It is helpful to also send information which are not appropriate in pe… in KII / 2:7 ¶ 17, Yeah, it will have effective change on community in KII / 2:9 ¶ 19, Yeah, previously mothers use the information they get during ANC, but… in KII / 2:11 ¶ 21, Information access, no transportation cost, and no professional resour… in KII / 2:36 ¶ 55, No need of motor or vehicles because it is mHealth. I will be happy to… in KII / 3:6 ¶ 5, They difference for mHealth is mother always get counseling or health… in Group discussion / 3:18 ¶ 12, It looks like mothers are tired of current service provision so the mH… in Group discussion / 3:23 ¶ 14, Previously, people think poor health system and there are many complia… in Group discussion / 3:24 ¶ 14, Over time this can change community perception and they may reebok mot… in Group discussion / 3:25 ¶ 14, There may be emerged women who are successful because of mHealth and t… in Group discussion / 3:27 ¶ 16, It can make the child health growth, for mothers to know danger signs,… in Group discussion / 3:38 ¶ 22, Of course having organized maternal data can also promote contacting a… in Group discussion / 3:41 ¶ 26, : It is a big opportunity to meet the patient again virtually especial… in Group discussion / 3:42 ¶ 26, Traditionally, when errors happen or if the patients go with the wrong… in Group discussion / 3:50 ¶ 33, Benefit for mothers for next pregnancies and healthy prospect for chil… in Group discussion / 3:59 ¶ 45, Satisfaction of their client service and familiarization with technolo… in Group discussion / 3:61 ¶ 47, Planning, decision making based on mHealth, and increased service take… in Group discussion / 3:62 ¶ 48, Support related to mHealth, fulfillment of deficiencies of human and o… in Group discussion / 3:67 ¶ 53, he respect for pregnancy mothers may think gave many births and not ca… in Group discussion

- **Benefits of mHealth: Help_professional**

  Comment: by Girma Gilano

  > *3/12/2023 11:46:46 AM, merged with Effectiviness 3/12/2023 1:12:09 PM, merged with Decision_making 3/12/2023 1:12:55 PM, merged with Role_improvement*

  30 Quotations:

  1:24 ¶ 34, We always give best service to our best capacity, but mHealth may adva… in interview analysis / 1:34 ¶ 39, As HEWs we cannot reach for every mother on time but text can easily r… in interview analysis / 1:36 ¶ 40, This mHealth better than our previous our service in interview analysis / 1:50 ¶ 57, further improve the already improving service in interview analysis / 1:56 ¶ 62, Our current ANC and other formats now request mother to register their… in interview analysis / 1:68 ¶ 70, Every system is getting digitalized so digitalizing health may have mu… in interview analysis / 1:82 ¶ 81, ers usually take what we tell them, it will be successful. I don't thi… in interview analysis / 2:3 ¶ 11, It is helpful to also send information which are not appropriate in pe… in KII / 2:7 ¶ 17, Yeah, it will have effective change on community in KII / 2:9 ¶ 19, Yeah, previously mothers use the information they get during ANC, but… in KII / 2:35 ¶ 54, Since we have many gaps this should be seen as an opportunity in KII / 3:2 ¶ 3, Previous teaching of mother through home to home is going to be throug… in Group discussion / 3:12 ¶ 8, For educated mothers messages will be option less in Group discussion / 3:13 ¶ 8, Starting from me we feed additional food at 2months because the kind o… in Group discussion / 3:18 ¶ 12, It looks like mothers are tired of current service provision so the mH… in Group discussion / 3:25 ¶ 14, There may be emerged women who are successful because of mHealth and t… in Group discussion / 3:29 ¶ 17, As a health professionals applying technology can only easy our burden… in Group discussion / 3:34 ¶ 19, ecause of mHealth we may every address of women and now we can also ca… in Group discussion / 3:35 ¶ 20, We will provide the service for mothers we know now, but previously we… in Group discussion / 3:38 ¶ 22, Of course having organized maternal data can also promote contacting a… in Group discussion / 3:41 ¶ 26, : It is a big opportunity to meet the patient again virtually especial… in Group discussion / 3:42 ¶ 26, Traditionally, when errors happen or if the patients go with the wrong… in Group discussion / 3:43 ¶ 28, At the end this is very important piece of technology that can reduce… in Group discussion / 3:46 ¶ 28, Those infrastructure and awareness creation in staffs, mothers, and co… in Group discussion / 3:52 ¶ 36, People will enjoy talking to others how health institution just concer… in Group discussion / 3:57 ¶ 42, Improve work environment as per the need of the technology and owning… in Group discussion / 3:58 ¶ 43, Aspiring more technologies and helping mothers, taking trainings, givi… in Group discussion / 3:60 ¶ 46, Experience with technology. Enable them to identify areas where mHealt… in Group discussion / 3:61 ¶ 47, Planning, decision making based on mHealth, and increased service take… in Group discussion / 3:62 ¶ 48, Support related to mHealth, fulfillment of deficiencies of human and o… in Group discussion

2:7 ¶ 17 in KII

Text quotation

Created by Girma Gilano on 3/9/2023

Yeah, it will have effective change on community

3 Codes:

- **Benefits of mHealth: Effective**

  Comment: by Girma Gilano

  > *3/12/2023 11:46:46 AM, merged with Effectiviness 3/12/2023 1:12:09 PM, merged with Decision_making 3/12/2023 1:12:55 PM, merged with Role_improvement*

  6 Quotations:

1:48 ¶ 55, t can reduce cost, time, and other resources in interview analysis / 1:60 ¶ 64, It will be very effective because it advances the services in interview analysis / 1:61 ¶ 65, I think it will be very effective because we can see the newly impleme… in interview analysis / 1:81 ¶ 80, pe mothers will turn to use mobiles, but only after getting its import… in interview analysis / 1:98 ¶ 98, If all input resources are fulfilled, there will be no problem. It is… in interview analysis / 2:7 ¶ 17, Yeah, it will have effective change on community in KII

Benefits of mHealth: Help_mothers

Comment: by Girma Gilano

> *3/12/2023 11:46:46 AM, merged with Effectiviness 3/12/2023 1:12:09 PM, merged with Decision_making 3/12/2023 1:12:55 PM, merged with Role_improvement*

27 Quotations:

1:41 ¶ 47, ion comes after understanding of benefits and harms in interview analysis / 1:45 ¶ 52, Accessibility can be improved mothers get information at their homes in interview analysis / 1:51 ¶ 58, She can access any information in interview analysis / 1:53 ¶ 59, I think mHealth can improve access, appointment on time, availability… in interview analysis / 1:65 ¶ 67, Her husband or children will also push her to go even if the condition… in interview analysis / 1:68 ¶ 70, Every system is getting digitalized so digitalizing health may have mu… in interview analysis / 1:77 ¶ 77, Rural mothers are respectful; they do what they told to do, so fear of… in interview analysis / 1:81 ¶ 80, pe mothers will turn to use mobiles, but only after getting its import… in interview analysis / 2:3 ¶ 11, It is helpful to also send information which are not appropriate in pe… in KII / 2:7 ¶ 17, Yeah, it will have effective change on community in KII / 2:9 ¶ 19, Yeah, previously mothers use the information they get during ANC, but… in KII / 2:11 ¶ 21, Information access, no transportation cost, and no professional resour… in KII / 2:36 ¶ 55, No need of motor or vehicles because it is mHealth. I will be happy to… in KII / 3:6 ¶ 5, They difference for mHealth is mother always get counseling or health… in Group discussion / 3:18 ¶ 12, It looks like mothers are tired of current service provision so the mH… in Group discussion / 3:23 ¶ 14, Previously, people think poor health system and there are many complia… in Group discussion / 3:24 ¶ 14, Over time this can change community perception and they may reebok mot… in Group discussion / 3:25 ¶ 14, There may be emerged women who are successful because of mHealth and t… in Group discussion / 3:27 ¶ 16, It can make the child health growth, for mothers to know danger signs,… in Group discussion / 3:38 ¶ 22, Of course having organized maternal data can also promote contacting a… in Group discussion / 3:41 ¶ 26, : It is a big opportunity to meet the patient again virtually especial… in Group discussion / 3:42 ¶ 26, Traditionally, when errors happen or if the patients go with the wrong… in Group discussion / 3:50 ¶ 33, Benefit for mothers for next pregnancies and healthy prospect for chil… in Group discussion / 3:59 ¶ 45, Satisfaction of their client service and familiarization with technolo… in Group discussion / 3:61 ¶ 47, Planning, decision making based on mHealth, and increased service take… in Group discussion / 3:62 ¶ 48, Support related to mHealth, fulfillment of deficiencies of human and o… in Group discussion / 3:67 ¶ 53, he respect for pregnancy mothers may think gave many births and not ca… in Group discussion

Benefits of mHealth: Help_professional

Comment: by Girma Gilano

> *3/12/2023 11:46:46 AM, merged with Effectiviness 3/12/2023 1:12:09 PM, merged with Decision_making 3/12/2023 1:12:55 PM, merged with Role_improvement*

30 Quotations:

1:24 ¶ 34, We always give best service to our best capacity, but mHealth may adva… in interview analysis / 1:34 ¶ 39, As HEWs we cannot reach for every mother on time but text can easily r… in interview analysis / 1:36 ¶ 40, This mHealth better than our previous our service in interview analysis / 1:50 ¶ 57, further improve the already improving service in interview analysis / 1:56 ¶ 62, Our current ANC and other formats now request mother to register their… in interview analysis / 1:68 ¶ 70, Every system is getting digitalized so digitalizing health may have mu… in interview analysis / 1:82 ¶ 81, ers usually take what we tell them, it will be successful. I don't thi… in interview analysis / 2:3 ¶ 11, It is helpful to also send information which are not appropriate in pe… in KII / 2:7 ¶ 17, Yeah, it will have effective change on community in KII / 2:9 ¶ 19, Yeah, previously mothers use the information they get

during ANC, but… in KII / 2:35 ¶ 54, Since we have many gaps this should be seen as an opportunity in KII / 3:2 ¶ 3, Previous teaching of mother through home to home is going to be throug… in Group discussion / 3:12 ¶ 8, For educated mothers messages will be option less in Group discussion / 3:13 ¶ 8, Starting from me we feed additional food at 2months because the kind o… in Group discussion / 3:18 ¶ 12, It looks like mothers are tired of current service provision so the mH… in Group discussion / 3:25 ¶ 14, There may be emerged women who are successful because of mHealth and t… in Group discussion / 3:29 ¶ 17, As a health professionals applying technology can only easy our burden… in Group discussion / 3:34 ¶ 19, ecause of mHealth we may every address of women and now we can also ca… in Group discussion / 3:35 ¶ 20, We will provide the service for mothers we know now, but previously we… in Group discussion / 3:38 ¶ 22, Of course having organized maternal data can also promote contacting a… in Group discussion / 3:41 ¶ 26, : It is a big opportunity to meet the patient again virtually especial… in Group discussion / 3:42 ¶ 26, Traditionally, when errors happen or if the patients go with the wrong… in Group discussion / 3:43 ¶ 28, At the end this is very important piece of technology that can reduce… in Group discussion / 3:46 ¶ 28, Those infrastructure and awareness creation in staffs, mothers, and co… in Group discussion / 3:52 ¶ 36, People will enjoy talking to others how health institution just concer… in Group discussion / 3:57 ¶ 42, Improve work environment as per the need of the technology and owning… in Group discussion / 3:58 ¶ 43, Aspiring more technologies and helping mothers, taking trainings, givi… in Group discussion / 3:60 ¶ 46, Experience with technology. Enable them to identify areas where mHealt… in Group discussion / 3:61 ¶ 47, Planning, decision making based on mHealth, and increased service take… in Group discussion / 3:62 ¶ 48, Support related to mHealth, fulfillment of deficiencies of human and o… in Group discussion

2:9 ¶ 19 in KII

Text quotation

Created by Girma Gilano on 3/9/2023

Yeah, previously mothers use the information they get during ANC, but mothers may forget as time progress so mHealth helpful backup

2 Codes:

- **Benefits of mHealth: Help_mothers**

Comment: by Girma Gilano

> *3/12/2023 11:46:46 AM, merged with Effectiviness 3/12/2023 1:12:09 PM, merged with Decision_making 3/12/2023 1:12:55 PM, merged with Role_improvement*

27 Quotations:

1:41 ¶ 47, ion comes after understanding of benefits and harms in interview analysis / 1:45 ¶ 52, Accessibility can be improved mothers get information at their homes in interview analysis / 1:51 ¶ 58, She can access any information in interview analysis / 1:53 ¶ 59, I think mHealth can improve access, appointment on time, availability… in interview analysis / 1:65 ¶ 67, Her husband or children will also push her to go even if the condition… in interview analysis / 1:68 ¶ 70, Every system is getting digitalized so digitalizing health may have mu… in interview analysis / 1:77 ¶ 77, Rural mothers are respectful; they do what they told to do, so fear of… in interview analysis / 1:81 ¶ 80, pe mothers will turn to use mobiles, but only after getting its import… in interview analysis / 2:3 ¶ 11, It is helpful to also send information which are not appropriate in pe… in KII / 2:7 ¶ 17, Yeah, it will have effective change on community in KII / 2:9 ¶ 19, Yeah, previously mothers use the information they get during ANC, but… in KII / 2:11 ¶ 21, Information access, no transportation cost, and no professional resour… in KII / 2:36 ¶ 55, No need of motor or vehicles because it is mHealth. I will be happy to… in KII / 3:6 ¶ 5, They difference for mHealth is mother always get counseling or health… in Group discussion / 3:18 ¶ 12, It looks like mothers are tired of current service provision so the mH… in Group discussion / 3:23 ¶ 14, Previously, people think poor health system and there are many complia… in Group discussion / 3:24 ¶ 14, Over time this can change community perception and they may reebok mot… in Group discussion / 3:25 ¶ 14, There may be emerged women who are successful because of mHealth and t… in Group discussion / 3:27 ¶ 16, It

can make the child health growth, for mothers to know danger signs,… in Group discussion / 3:38 ¶ 22, Of course having organized maternal data can also promote contacting a… in Group discussion / 3:41 ¶ 26, : It is a big opportunity to meet the patient again virtually especial… in Group discussion / 3:42 ¶ 26, Traditionally, when errors happen or if the patients go with the wrong… in Group discussion / 3:50 ¶ 33, Benefit for mothers for next pregnancies and healthy prospect for chil… in Group discussion / 3:59 ¶ 45, Satisfaction of their client service and familiarization with technolo… in Group discussion / 3:61 ¶ 47, Planning, decision making based on mHealth, and increased service take… in Group discussion / 3:62 ¶ 48, Support related to mHealth, fulfillment of deficiencies of human and o… in Group discussion / 3:67 ¶ 53, he respect for pregnancy mothers may think gave many births and not ca… in Group discussion

● Benefits of mHealth: Help_professional

Comment: by Girma Gilano

> *3/12/2023 11:46:46 AM, merged with Effectiviness 3/12/2023 1:12:09 PM, merged with Decision_making 3/12/2023 1:12:55 PM, merged with Role_improvement*

30 Quotations:

1:24 ¶ 34, We always give best service to our best capacity, but mHealth may adva… in interview analysis / 1:34 ¶ 39, As HEWs we cannot reach for every mother on time but text can easily r… in interview analysis / 1:36 ¶ 40, This mHealth better than our previous our service in interview analysis / 1:50 ¶ 57, further improve the already improving service in interview analysis / 1:56 ¶ 62, Our current ANC and other formats now request mother to register their… in interview analysis / 1:68 ¶ 70, Every system is getting digitalized so digitalizing health may have mu… in interview analysis / 1:82 ¶ 81, ers usually take what we tell them, it will be successful. I don’t thi… in interview analysis / 2:3 ¶ 11, It is helpful to also send information which are not appropriate in pe… in KII / 2:7 ¶ 17, Yeah, it will have effective change on community in KII / 2:9 ¶ 19, Yeah, previously mothers use the information they get during ANC, but… in KII / 2:35 ¶ 54, Since we have many gaps this should be seen as an opportunity in KII / 3:2 ¶ 3, Previous teaching of mother through home to home is going to be throug… in Group discussion / 3:12 ¶ 8, For educated mothers messages will be option less in Group discussion / 3:13 ¶ 8, Starting from me we feed additional food at 2months because the kind o… in Group discussion / 3:18 ¶ 12, It looks like mothers are tired of current service provision so the mH… in Group discussion / 3:25 ¶ 14, There may be emerged women who are successful because of mHealth and t… in Group discussion / 3:29 ¶ 17, As a health professionals applying technology can only easy our burden… in Group discussion / 3:34 ¶ 19, ecause of mHealth we may every address of women and now we can also ca… in Group discussion / 3:35 ¶ 20, We will provide the service for mothers we know now, but previously we… in Group discussion / 3:38 ¶ 22, Of course having organized maternal data can also promote contacting a… in Group discussion / 3:41 ¶ 26, : It is a big opportunity to meet the patient again virtually especial… in Group discussion / 3:42 ¶ 26, Traditionally, when errors happen or if the patients go with the wrong… in Group discussion / 3:43 ¶ 28, At the end this is very important piece of technology that can reduce… in Group discussion / 3:46 ¶ 28, Those infrastructure and awareness creation in staffs, mothers, and co… in Group discussion / 3:52 ¶ 36, People will enjoy talking to others how health institution just concer… in Group discussion / 3:57 ¶ 42, Improve work environment as per the need of the technology and owning… in Group discussion / 3:58 ¶ 43, Aspiring more technologies and helping mothers, taking trainings, givi… in Group discussion / 3:60 ¶ 46, Experience with technology. Enable them to identify areas where mHealt… in Group discussion / 3:61 ¶ 47, Planning, decision making based on mHealth, and increased service take… in Group discussion / 3:62 ¶ 48, Support related to mHealth, fulfillment of deficiencies of human and o… in Group discussion

2:11 ¶ 21 in KII

Text quotation

Created by Girma Gilano on 3/9/2023

Information access, no transportation cost, and no professional resource waste are very good parts of the mHealth

1 Codes:

- **Benefits of mHealth: Help_mothers**

Comment: by Girma Gilano

> *3/12/2023 11:46:46 AM, merged with Effectiviness 3/12/2023 1:12:09 PM, merged with Decision_making 3/12/2023 1:12:55 PM, merged with Role_improvement*

27 Quotations:

1:41 ¶ 47, ion comes after understanding of benefits and harms in interview analysis / 1:45 ¶ 52, Accessibility can be improved mothers get information at their homes in interview analysis / 1:51 ¶ 58, She can access any information in interview analysis / 1:53 ¶ 59, I think mHealth can improve access, appointment on time, availability… in interview analysis / 1:65 ¶ 67, Her husband or children will also push her to go even if the condition… in interview analysis / 1:68 ¶ 70, Every system is getting digitalized so digitalizing health may have mu… in interview analysis / 1:77 ¶ 77, Rural mothers are respectful; they do what they told to do, so fear of… in interview analysis / 1:81 ¶ 80, pe mothers will turn to use mobiles, but only after getting its import… in interview analysis / 2:3 ¶ 11, It is helpful to also send information which are not appropriate in pe… in KII / 2:7 ¶ 17, Yeah, it will have effective change on community in KII / 2:9 ¶ 19, Yeah, previously mothers use the information they get during ANC, but… in KII / 2:11 ¶ 21, Information access, no transportation cost, and no professional resour… in KII / 2:36 ¶ 55, No need of motor or vehicles because it is mHealth. I will be happy to… in KII / 3:6 ¶ 5, They difference for mHealth is mother always get counseling or health… in Group discussion / 3:18 ¶ 12, It looks like mothers are tired of current service provision so the mH… in Group discussion / 3:23 ¶ 14, Previously, people think poor health system and there are many complia… in Group discussion / 3:24 ¶ 14, Over time this can change community perception and they may reebok mot… in Group discussion / 3:25 ¶ 14, There may be emerged women who are successful because of mHealth and t… in Group discussion / 3:27 ¶ 16, It can make the child health growth, for mothers to know danger signs,… in Group discussion / 3:38 ¶ 22, Of course having organized maternal data can also promote contacting a… in Group discussion / 3:41 ¶ 26, : It is a big opportunity to meet the patient again virtually especial… in Group discussion / 3:42 ¶ 26, Traditionally, when errors happen or if the patients go with the wrong… in Group discussion / 3:50 ¶ 33, Benefit for mothers for next pregnancies and healthy prospect for chil… in Group discussion / 3:59 ¶ 45, Satisfaction of their client service and familiarization with technolo… in Group discussion / 3:61 ¶ 47, Planning, decision making based on mHealth, and increased service take… in Group discussion / 3:62 ¶ 48, Support related to mHealth, fulfillment of deficiencies of human and o… in Group discussion / 3:67 ¶ 53, he respect for pregnancy mothers may think gave many births and not ca… in Group discussion

2:36 ¶ 55 in KII

Text quotation

Created by Girma Gilano on 3/9/2023

No need of motor or vehicles because it is mHealth. I will be happy to tell people this service

1 Codes:

- **Benefits of mHealth: Help_mothers**

Comment: by Girma Gilano

> *3/12/2023 11:46:46 AM, merged with Effectiviness 3/12/2023 1:12:09 PM, merged with Decision_making 3/12/2023 1:12:55 PM, merged with Role_improvement*

27 Quotations:

1:41 ¶ 47, ion comes after understanding of benefits and harms in interview analysis / 1:45 ¶ 52, Accessibility can be improved mothers get information at their homes in interview analysis / 1:51 ¶ 58, She can access any information in interview analysis / 1:53 ¶ 59, I think mHealth can improve access, appointment on time, availability… in interview analysis / 1:65 ¶ 67, Her husband or children will also push her to go even if the condition… in interview analysis / 1:68 ¶ 70, Every system is getting digitalized so digitalizing health may have mu… in interview analysis / 1:77 ¶ 77, Rural mothers are respectful; they do what they told to do, so fear of… in interview analysis / 1:81 ¶ 80, pe mothers will turn to use mobiles, but only after getting its import… in interview analysis / 2:3 ¶ 11, It is helpful to also send information which are not appropriate in pe… in KII / 2:7 ¶ 17, Yeah, it will have effective change on community in KII / 2:9 ¶ 19, Yeah, previously mothers use the information they get during ANC, but… in KII / 2:11 ¶ 21, Information access, no transportation cost, and no professional resour… in KII / 2:36 ¶ 55, No need of motor or vehicles because it is mHealth. I will be happy to… in KII / 3:6 ¶ 5, They difference for mHealth is mother always get counseling or health… in Group discussion / 3:18 ¶ 12, It looks like mothers are tired of current service provision so the mH… in Group discussion / 3:23 ¶ 14, Previously, people think poor health system and there are many complia… in Group discussion / 3:24 ¶ 14, Over time this can change community perception and they may reebok mot… in Group discussion / 3:25 ¶ 14, There may be emerged women who are successful because of mHealth and t… in Group discussion / 3:27 ¶ 16, It can make the child health growth, for mothers to know danger signs,… in Group discussion / 3:38 ¶ 22, Of course having organized maternal data can also promote contacting a… in Group discussion / 3:41 ¶ 26, : It is a big opportunity to meet the patient again virtually especial… in Group discussion / 3:42 ¶ 26, Traditionally, when errors happen or if the patients go with the wrong… in Group discussion / 3:50 ¶ 33, Benefit for mothers for next pregnancies and healthy prospect for chil… in Group discussion / 3:59 ¶ 45, Satisfaction of their client service and familiarization with technolo… in Group discussion / 3:61 ¶ 47, Planning, decision making based on mHealth, and increased service take… in Group discussion / 3:62 ¶ 48, Support related to mHealth, fulfillment of deficiencies of human and o… in Group discussion / 3:67 ¶ 53, he respect for pregnancy mothers may think gave many births and not ca… in Group discussion

3:6 ¶ 5 in Group discussion

Text quotation

Created by Girma Gilano on 3/9/2023

They difference for mHealth is mother always get counseling or health education unlike that of visits when large queue limits they counseling and professionals do finishing work. I think mHealth can be benefited if the current behavior can be improved

2 Codes:

- **Benefits of mHealth: Help_mothers**

Comment: by Girma Gilano

> *3/12/2023 11:46:46 AM, merged with Effectiviness 3/12/2023 1:12:09 PM, merged with Decision_making 3/12/2023 1:12:55 PM, merged with Role_improvement*

27 Quotations:

1:41 ¶ 47, ion comes after understanding of benefits and harms in interview analysis / 1:45 ¶ 52, Accessibility can be improved mothers get information at their homes in interview analysis / 1:51 ¶ 58, She can access any information in interview analysis / 1:53 ¶ 59, I think mHealth can improve access, appointment on time, availability… in interview analysis / 1:65 ¶ 67, Her husband or children will also push her to go even if the condition… in interview analysis / 1:68 ¶ 70, Every system is getting digitalized so digitalizing health may have mu… in interview analysis / 1:77 ¶ 77, Rural mothers are respectful; they do what they told to do, so fear of… in interview analysis / 1:81 ¶ 80, pe mothers will turn to use mobiles, but only after getting its import… in interview analysis / 2:3 ¶ 11, It is helpful to also send information which are not appropriate in pe… in KII / 2:7 ¶ 17, Yeah, it will have effective change on community in KII / 2:9 ¶ 19, Yeah, previously mothers use the information they get during ANC, but… in KII / 2:11 ¶ 21, Information access, no transportation cost, and no professional resour… in KII / 2:36 ¶ 55, No need of

motor or vehicles because it is mHealth. I will be happy to… in KII / 3:6 ¶ 5, They difference for mHealth is mother always get counseling or health… in Group discussion / 3:18 ¶ 12, It looks like mothers are tired of current service provision so the mH… in Group discussion / 3:23 ¶ 14, Previously, people think poor health system and there are many complia… in Group discussion / 3:24 ¶ 14, Over time this can change community perception and they may reebok mot… in Group discussion / 3:25 ¶ 14, There may be emerged women who are successful because of mHealth and t… in Group discussion / 3:27 ¶ 16, It can make the child health growth, for mothers to know danger signs,… in Group discussion / 3:38 ¶ 22, Of course having organized maternal data can also promote contacting a… in Group discussion / 3:41 ¶ 26, : It is a big opportunity to meet the patient again virtually especial… in Group discussion / 3:42 ¶ 26, Traditionally, when errors happen or if the patients go with the wrong… in Group discussion / 3:50 ¶ 33, Benefit for mothers for next pregnancies and healthy prospect for chil… in Group discussion / 3:59 ¶ 45, Satisfaction of their client service and familiarization with technolo… in Group discussion / 3:61 ¶ 47, Planning, decision making based on mHealth, and increased service take… in Group discussion / 3:62 ¶ 48, Support related to mHealth, fulfillment of deficiencies of human and o… in Group discussion / 3:67 ¶ 53, he respect for pregnancy mothers may think gave many births and not ca… in Group discussion

Benefits of mHealth: Improve_decision

Comment: by Girma Gilano

> *3/12/2023 11:46:46 AM, merged with Effectiviness 3/12/2023 1:12:09 PM, merged with Decision_making 3/12/2023 1:12:55 PM, merged with Role_improvement*

17 Quotations:

1:5 ¶ 20, t will improve mothers’ time wastage for information that they can get… in interview analysis / 1:23 ¶ 33, will be helpful because it will remain in the mother’s hand for a long… in interview analysis / 1:40 ¶ 45, s it can improve their decision making. This service could increase wo… in interview analysis / 1:42 ¶ 49, provide information to make decisions, motivate her to convince her hu… in interview analysis / 1:49 ¶ 56, Having information on time can improve the health of mothers and child… in interview analysis / 1:55 ¶ 60, mHealth can provide evidence to make decision and can improve everythi… in interview analysis / 1:64 ¶ 67, However, having this information in her hand will continuously strike… in interview analysis / 1:70 ¶ 71, Service can be improved through mHealth because atleast having informa… in interview analysis / 2:15 ¶ 27, Mothers should understand initially the important, but on our side I d… in KII / 3:6 ¶ 5, They difference for mHealth is mother always get counseling or health… in Group discussion / 3:10 ¶ 8, Mother are already exposed to health education, but this one is direct… in Group discussion / 3:20 ¶ 12, They may even consider the increased risk of danger related to pregnan… in Group discussion / 3:24 ¶ 14, Over time this can change community perception and they may reebok mot… in Group discussion / 3:37 ¶ 20, It can also introduce labor division because we may know who must serv… in Group discussion / 3:48 ¶ 31, Service at their home and Information and on time vaccination and Heal… in Group discussion / 3:52 ¶ 36, People will enjoy talking to others how health institution just concer… in Group discussion / 3:67 ¶ 53, he respect for pregnancy mothers may think gave many births and not ca… in Group discussion

3:18 ¶ 12 in Group discussion

Text quotation

Created by Girma Gilano on 3/9/2023

It looks like mothers are tired of current service provision so the mHealth may be liked by them

3 Codes:

Benefits of mHealth: Help_mothers

Comment: by Girma Gilano

3/12/2023 11:46:46 AM, merged with Effectiviness 3/12/2023 1:12:09 PM, merged with Decision_making 3/12/2023 1:12:55 PM, merged with Role_improvement

27 Quotations:

1:41 ¶ 47, ion comes after understanding of benefits and harms in interview analysis / 1:45 ¶ 52, Accessibility can be improved mothers get information at their homes in interview analysis / 1:51 ¶ 58, She can access any information in interview analysis / 1:53 ¶ 59, I think mHealth can improve access, appointment on time, availability… in interview analysis / 1:65 ¶ 67, Her husband or children will also push her to go even if the condition… in interview analysis / 1:68 ¶ 70, Every system is getting digitalized so digitalizing health may have mu… in interview analysis / 1:77 ¶ 77, Rural mothers are respectful; they do what they told to do, so fear of… in interview analysis / 1:81 ¶ 80, pe mothers will turn to use mobiles, but only after getting its import… in interview analysis / 2:3 ¶ 11, It is helpful to also send information which are not appropriate in pe… in KII / 2:7 ¶ 17, Yeah, it will have effective change on community in KII / 2:9 ¶ 19, Yeah, previously mothers use the information they get during ANC, but… in KII / 2:11 ¶ 21, Information access, no transportation cost, and no professional resour… in KII / 2:36 ¶ 55, No need of motor or vehicles because it is mHealth. I will be happy to… in KII / 3:6 ¶ 5, They difference for mHealth is mother always get counseling or health… in Group discussion / 3:18 ¶ 12, It looks like mothers are tired of current service provision so the mH… in Group discussion / 3:23 ¶ 14, Previously, people think poor health system and there are many complia… in Group discussion / 3:24 ¶ 14, Over time this can change community perception and they may reebok mot… in Group discussion / 3:25 ¶ 14, There may be emerged women who are successful because of mHealth and t… in Group discussion / 3:27 ¶ 16, It can make the child health growth, for mothers to know danger signs,… in Group discussion / 3:38 ¶ 22, Of course having organized maternal data can also promote contacting a… in Group discussion / 3:41 ¶ 26, : It is a big opportunity to meet the patient again virtually especial… in Group discussion / 3:42 ¶ 26, Traditionally, when errors happen or if the patients go with the wrong… in Group discussion / 3:50 ¶ 33, Benefit for mothers for next pregnancies and healthy prospect for chil… in Group discussion / 3:59 ¶ 45, Satisfaction of their client service and familiarization with technolo… in Group discussion / 3:61 ¶ 47, Planning, decision making based on mHealth, and increased service take… in Group discussion / 3:62 ¶ 48, Support related to mHealth, fulfillment of deficiencies of human and o… in Group discussion / 3:67 ¶ 53, he respect for pregnancy mothers may think gave many births and not ca… in Group discussion

● Benefits of mHealth: Help_professional

Comment: by Girma Gilano

3/12/2023 11:46:46 AM, merged with Effectiviness 3/12/2023 1:12:09 PM, merged with Decision_making 3/12/2023 1:12:55 PM, merged with Role_improvement

30 Quotations:

1:24 ¶ 34, We always give best service to our best capacity, but mHealth may adva… in interview analysis / 1:34 ¶ 39, As HEWs we cannot reach for every mother on time but text can easily r… in interview analysis / 1:36 ¶ 40, This mHealth better than our previous our service in interview analysis / 1:50 ¶ 57, further improve the already improving service in interview analysis / 1:56 ¶ 62, Our current ANC and other formats now request mother to register their… in interview analysis / 1:68 ¶ 70, Every system is getting digitalized so digitalizing health may have mu… in interview analysis / 1:82 ¶ 81, ers usually take what we tell them, it will be successful. I don’t thi… in interview analysis / 2:3 ¶ 11, It is helpful to also send information which are not appropriate in pe… in KII / 2:7 ¶ 17, Yeah, it will have effective change on community in KII / 2:9 ¶ 19, Yeah, previously mothers use the information they get during ANC, but… in KII / 2:35 ¶ 54, Since we have many gaps this should be seen as an opportunity in KII / 3:2 ¶ 3, Previous teaching of mother through home to home is going to be throug… in Group discussion / 3:12 ¶ 8, For educated mothers messages will be option less in Group discussion / 3:13 ¶ 8, Starting from me we feed additional food at 2months because the kind o… in Group discussion / 3:18 ¶ 12, It looks like mothers are tired of current service provision so the mH… in Group discussion / 3:25 ¶ 14, There may be emerged women who are successful because of mHealth and t… in Group discussion / 3:29 ¶ 17, As a health professionals applying technology can only easy our burden… in Group discussion / 3:34 ¶ 19, ecause of mHealth we may every address of women and now we can also ca… in Group discussion / 3:35 ¶ 20, We will provide the service for mothers we know now, but previously we… in Group discussion / 3:38 ¶ 22, Of course having organized maternal data can also promote contacting a… in Group discussion / 3:41 ¶ 26, : It is a big opportunity to meet the patient again virtually

especial… in Group discussion / 3:42 ¶ 26, Traditionally, when errors happen or if the patients go with the wrong… in Group discussion / 3:43 ¶ 28, At the end this is very important piece of technology that can reduce… in Group discussion / 3:46 ¶ 28, Those infrastructure and awareness creation in staffs, mothers, and co… in Group discussion / 3:52 ¶ 36, People will enjoy talking to others how health institution just concer… in Group discussion / 3:57 ¶ 42, Improve work environment as per the need of the technology and owning… in Group discussion / 3:58 ¶ 43, Aspiring more technologies and helping mothers, taking trainings, givi… in Group discussion / 3:60 ¶ 46, Experience with technology. Enable them to identify areas where mHealt… in Group discussion / 3:61 ¶ 47, Planning, decision making based on mHealth, and increased service take… in Group discussion / 3:62 ¶ 48, Support related to mHealth, fulfillment of deficiencies of human and o… in Group discussion

- **Benefits of mHealth: Improve_MCH**

Comment: by Girma Gilano

> *3/12/2023 11:46:46 AM, merged with Effectiviness 3/12/2023 1:12:09 PM, merged with Decision_making 3/12/2023 1:12:55 PM, merged with Role_improvement*

25 Quotations:

1:6 ¶ 22, Non-use of the service because of negligence and forgetting can be im… in interview analysis / 1:26 ¶ 35, t can increase utilization better than that we have previously in interview analysis / 1:28 ¶ 36, Even since this digital it can further improve the service and increas… in interview analysis / 1:36 ¶ 40, This mHealth better than our previous our service in interview analysis / 1:43 ¶ 50, For maternal and child health the decision to take service is mothers… in interview analysis / 1:49 ¶ 56, Having information on time can improve the health of mothers and child… in interview analysis / 1:50 ¶ 57, further improve the already improving service in interview analysis / 1:57 ¶ 63, f it continue, it can be effective and be important than previous way… in interview analysis / 1:59 ¶ 64, I think whatever we put in to the community to improve health service… in interview analysis / 1:62 ¶ 66, This can improve and put our usual service one step forward in interview analysis / 1:74 ¶ 75, I don’t think people can be affected negatively because they love to l… in interview analysis / 1:77 ¶ 77, Rural mothers are respectful; they do what they told to do, so fear of… in interview analysis / 1:78 ¶ 78, One thing I assure is it will increase up taking maternal and child he… in interview analysis / 1:83 ¶ 83, It can remind which is especially important for family planning. Witho… in interview analysis / 2:4 ¶ 12, If health system organized this way it will be helpful in KII / 2:6 ¶ 16, this is the main thing to improve maternal and child care especially u… in KII / 3:8 ¶ 6, After counseling and aware mother it can be improved in Group discussion / 3:9 ¶ 8, It can improve those listed service (ANC, PNC, Breastfeeding and vacci… in Group discussion / 3:10 ¶ 8, Mother are already exposed to health education, but this one is direct… in Group discussion / 3:18 ¶ 12, It looks like mothers are tired of current service provision so the mH… in Group discussion / 3:19 ¶ 12, So in that case it can completely change the stream and interest. Or t… in Group discussion / 3:20 ¶ 12, They may even consider the increased risk of danger related to pregnan… in Group discussion / 3:43 ¶ 28, At the end this is very important piece of technology that can reduce… in Group discussion / 3:48 ¶ 31, Service at their home and Information and on time vaccination and Heal… in Group discussion / 3:49 ¶ 32, Healthy child feeding and behavioral change for mother that wll be lon… in Group discussion

3:23 ¶ 14 in Group discussion

Text quotation

Created by Girma Gilano on 3/9/2023

Previously, people think poor health system and there are many compliant for which reason they are not coming to service area; mHealth may tell the people that the provider and the government are now giving good attention

1 Codes:

- **Benefits of mHealth: Help_mothers**

Comment: by Girma Gilano

> *3/12/2023 11:46:46 AM, merged with Effectiviness 3/12/2023 1:12:09 PM, merged with Decision_making 3/12/2023 1:12:55 PM, merged with Role_improvement*

27 Quotations:

1:41 ¶ 47, ion comes after understanding of benefits and harms in interview analysis / 1:45 ¶ 52, Accessibility can be improved mothers get information at their homes in interview analysis / 1:51 ¶ 58, She can access any information in interview analysis / 1:53 ¶ 59, I think mHealth can improve access, appointment on time, availability… in interview analysis / 1:65 ¶ 67, Her husband or children will also push her to go even if the condition… in interview analysis / 1:68 ¶ 70, Every system is getting digitalized so digitalizing health may have mu… in interview analysis / 1:77 ¶ 77, Rural mothers are respectful; they do what they told to do, so fear of… in interview analysis / 1:81 ¶ 80, pe mothers will turn to use mobiles, but only after getting its import… in interview analysis / 2:3 ¶ 11, It is helpful to also send information which are not appropriate in pe… in KII / 2:7 ¶ 17, Yeah, it will have effective change on community in KII / 2:9 ¶ 19, Yeah, previously mothers use the information they get during ANC, but… in KII / 2:11 ¶ 21, Information access, no transportation cost, and no professional resour… in KII / 2:36 ¶ 55, No need of motor or vehicles because it is mHealth. I will be happy to… in KII / 3:6 ¶ 5, They difference for mHealth is mother always get counseling or health… in Group discussion / 3:18 ¶ 12, It looks like mothers are tired of current service provision so the mH… in Group discussion / 3:23 ¶ 14, Previously, people think poor health system and there are many complia… in Group discussion / 3:24 ¶ 14, Over time this can change community perception and they may reebok mot… in Group discussion / 3:25 ¶ 14, There may be emerged women who are successful because of mHealth and t… in Group discussion / 3:27 ¶ 16, It can make the child health growth, for mothers to know danger signs,… in Group discussion / 3:38 ¶ 22, Of course having organized maternal data can also promote contacting a… in Group discussion / 3:41 ¶ 26, : It is a big opportunity to meet the patient again virtually especial… in Group discussion / 3:42 ¶ 26, Traditionally, when errors happen or if the patients go with the wrong… in Group discussion / 3:50 ¶ 33, Benefit for mothers for next pregnancies and healthy prospect for chil… in Group discussion / 3:59 ¶ 45, Satisfaction of their client service and familiarization with technolo… in Group discussion / 3:61 ¶ 47, Planning, decision making based on mHealth, and increased service take… in Group discussion / 3:62 ¶ 48, Support related to mHealth, fulfillment of deficiencies of human and o… in Group discussion / 3:67 ¶ 53, he respect for pregnancy mothers may think gave many births and not ca… in Group discussion

3:24 ¶ 14 in Group discussion

Text quotation

Created by Girma Gilano on 3/9/2023

Over time this can change community perception and they may reebok mothers who faced the health problem because of poor follow up and poor usage of messages because they already knew that they are been under intensive attention follow up

2 Codes:

- **Benefits of mHealth: Help_mothers**

Comment: by Girma Gilano

> *3/12/2023 11:46:46 AM, merged with Effectiviness 3/12/2023 1:12:09 PM, merged with Decision_making 3/12/2023 1:12:55 PM, merged with Role_improvement*

27 Quotations:

1:41 ¶ 47, ion comes after understanding of benefits and harms in interview analysis / 1:45 ¶ 52, Accessibility can be improved mothers get information at their homes in interview analysis / 1:51 ¶ 58, She can access any information in interview analysis / 1:53 ¶ 59, I think mHealth can improve access, appointment on time, availability… in interview analysis / 1:65 ¶ 67, Her husband or children will also

push her to go even if the condition… in interview analysis / 1:68 ¶ 70, Every system is getting digitalized so digitalizing health may have mu… in interview analysis / 1:77 ¶ 77, Rural mothers are respectful; they do what they told to do, so fear of… in interview analysis / 1:81 ¶ 80, pe mothers will turn to use mobiles, but only after getting its import… in interview analysis / 2:3 ¶ 11, It is helpful to also send information which are not appropriate in pe… in KII / 2:7 ¶ 17, Yeah, it will have effective change on community in KII / 2:9 ¶ 19, Yeah, previously mothers use the information they get during ANC, but… in KII / 2:11 ¶ 21, Information access, no transportation cost, and no professional resour… in KII / 2:36 ¶ 55, No need of motor or vehicles because it is mHealth. I will be happy to… in KII / 3:6 ¶ 5, They difference for mHealth is mother always get counseling or health… in Group discussion / 3:18 ¶ 12, It looks like mothers are tired of current service provision so the mH… in Group discussion / 3:23 ¶ 14, Previously, people think poor health system and there are many complia… in Group discussion / 3:24 ¶ 14, Over time this can change community perception and they may reebok mot… in Group discussion / 3:25 ¶ 14, There may be emerged women who are successful because of mHealth and t… in Group discussion / 3:27 ¶ 16, It can make the child health growth, for mothers to know danger signs,… in Group discussion / 3:38 ¶ 22, Of course having organized maternal data can also promote contacting a… in Group discussion / 3:41 ¶ 26, : It is a big opportunity to meet the patient again virtually especial… in Group discussion / 3:42 ¶ 26, Traditionally, when errors happen or if the patients go with the wrong… in Group discussion / 3:50 ¶ 33, Benefit for mothers for next pregnancies and healthy prospect for chil… in Group discussion / 3:59 ¶ 45, Satisfaction of their client service and familiarization with technolo… in Group discussion / 3:61 ¶ 47, Planning, decision making based on mHealth, and increased service take… in Group discussion / 3:62 ¶ 48, Support related to mHealth, fulfillment of deficiencies of human and o… in Group discussion / 3:67 ¶ 53, he respect for pregnancy mothers may think gave many births and not ca… in Group discussion

● Benefits of mHealth: Improve_decision

Comment: by Girma Gilano

> *3/12/2023 11:46:46 AM, merged with Effectiviness 3/12/2023 1:12:09 PM, merged with Decision_making 3/12/2023 1:12:55 PM, merged with Role_improvement*

17 Quotations:

1:5 ¶ 20, t will improve mothers' time wastage for information that they can get… in interview analysis / 1:23 ¶ 33, will be helpful because it will remain in the mother's hand for a long… in interview analysis / 1:40 ¶ 45, s it can improve their decision making. This service could increase wo… in interview analysis / 1:42 ¶ 49, provide information to make decisions, motivate her to convince her hu… in interview analysis / 1:49 ¶ 56, Having information on time can improve the health of mothers and child… in interview analysis / 1:55 ¶ 60, mHealth can provide evidence to make decision and can improve everythi… in interview analysis / 1:64 ¶ 67, However, having this information in her hand will continuously strike… in interview analysis / 1:70 ¶ 71, Service can be improved through mHealth because atleast having informa… in interview analysis / 2:15 ¶ 27, Mothers should understand initially the important, but on our side I d… in KII / 3:6 ¶ 5, They difference for mHealth is mother always get counseling or health… in Group discussion / 3:10 ¶ 8, Mother are already exposed to health education, but this one is direct… in Group discussion / 3:20 ¶ 12, They may even consider the increased risk of danger related to pregnan… in Group discussion / 3:24 ¶ 14, Over time this can change community perception and they may reebok mot… in Group discussion / 3:37 ¶ 20, It can also introduce labor division because we may know who must serv… in Group discussion / 3:48 ¶ 31, Service at their home and Information and on time vaccination and Heal… in Group discussion / 3:52 ¶ 36, People will enjoy talking to others how health institution just concer… in Group discussion / 3:67 ¶ 53, he respect for pregnancy mothers may think gave many births and not ca… in Group discussion

3:25 ¶ 14 in Group discussion

Text quotation

Created by Girma Gilano on 3/9/2023

There may be emerged women who are successful because of mHealth and they may tell the story of mHealth which further educate the community

2 Codes:

Benefits of mHealth: Help_mothers

Comment: by Girma Gilano

> *3/12/2023 11:46:46 AM, merged with Effectiviness 3/12/2023 1:12:09 PM, merged with Decision_making 3/12/2023 1:12:55 PM, merged with Role_improvement*

27 Quotations:

1:41 ¶ 47, ion comes after understanding of benefits and harms in interview analysis / 1:45 ¶ 52, Accessibility can be improved mothers get information at their homes in interview analysis / 1:51 ¶ 58, She can access any information in interview analysis / 1:53 ¶ 59, I think mHealth can improve access, appointment on time, availability… in interview analysis / 1:65 ¶ 67, Her husband or children will also push her to go even if the condition… in interview analysis / 1:68 ¶ 70, Every system is getting digitalized so digitalizing health may have mu… in interview analysis / 1:77 ¶ 77, Rural mothers are respectful; they do what they told to do, so fear of… in interview analysis / 1:81 ¶ 80, pe mothers will turn to use mobiles, but only after getting its import… in interview analysis / 2:3 ¶ 11, It is helpful to also send information which are not appropriate in pe… in KII / 2:7 ¶ 17, Yeah, it will have effective change on community in KII / 2:9 ¶ 19, Yeah, previously mothers use the information they get during ANC, but… in KII / 2:11 ¶ 21, Information access, no transportation cost, and no professional resour… in KII / 2:36 ¶ 55, No need of motor or vehicles because it is mHealth. I will be happy to… in KII / 3:6 ¶ 5, They difference for mHealth is mother always get counseling or health… in Group discussion / 3:18 ¶ 12, It looks like mothers are tired of current service provision so the mH… in Group discussion / 3:23 ¶ 14, Previously, people think poor health system and there are many complia… in Group discussion / 3:24 ¶ 14, Over time this can change community perception and they may reebok mot… in Group discussion / 3:25 ¶ 14, There may be emerged women who are successful because of mHealth and t… in Group discussion / 3:27 ¶ 16, It can make the child health growth, for mothers to know danger signs,… in Group discussion / 3:38 ¶ 22, Of course having organized maternal data can also promote contacting a… in Group discussion / 3:41 ¶ 26, : It is a big opportunity to meet the patient again virtually especial… in Group discussion / 3:42 ¶ 26, Traditionally, when errors happen or if the patients go with the wrong… in Group discussion / 3:50 ¶ 33, Benefit for mothers for next pregnancies and healthy prospect for chil… in Group discussion / 3:59 ¶ 45, Satisfaction of their client service and familiarization with technolo… in Group discussion / 3:61 ¶ 47, Planning, decision making based on mHealth, and increased service take… in Group discussion / 3:62 ¶ 48, Support related to mHealth, fulfillment of deficiencies of human and o… in Group discussion / 3:67 ¶ 53, he respect for pregnancy mothers may think gave many births and not ca… in Group discussion

Benefits of mHealth: Help_professional

Comment: by Girma Gilano

> *3/12/2023 11:46:46 AM, merged with Effectiviness 3/12/2023 1:12:09 PM, merged with Decision_making 3/12/2023 1:12:55 PM, merged with Role_improvement*

30 Quotations:

1:24 ¶ 34, We always give best service to our best capacity, but mHealth may adva… in interview analysis / 1:34 ¶ 39, As HEWs we cannot reach for every mother on time but text can easily r… in interview analysis / 1:36 ¶ 40, This mHealth better than our previous our service in interview analysis / 1:50 ¶ 57, further improve the already improving service in interview analysis / 1:56 ¶ 62, Our current ANC and other formats now request mother to register their… in interview analysis / 1:68 ¶ 70, Every system is getting digitalized so digitalizing health may have mu… in interview analysis / 1:82 ¶ 81, ers usually take what we tell them, it will be successful. I don't thi… in interview analysis / 2:3 ¶ 11, It is helpful to also send information which are not appropriate in pe… in KII / 2:7 ¶ 17, Yeah, it will have effective change on community in KII / 2:9 ¶ 19, Yeah, previously mothers use the information they get during ANC, but… in KII / 2:35 ¶ 54, Since we have many gaps this should be seen as an opportunity in KII / 3:2 ¶ 3, Previous teaching of mother through home to home is going to be throug… in Group discussion / 3:12 ¶ 8, For educated mothers messages will be option less in Group discussion / 3:13 ¶ 8, Starting from me we feed additional food at 2months because the kind o… in Group discussion / 3:18 ¶ 12, It looks like mothers are tired of current service provision so the mH… in Group discussion / 3:25 ¶

14, There may be emerged women who are successful because of mHealth and t… in Group discussion / 3:29 ¶ 17, As a health professionals applying technology can only easy our burden… in Group discussion / 3:34 ¶ 19, ecause of mHealth we may every address of women and now we can also ca… in Group discussion / 3:35 ¶ 20, We will provide the service for mothers we know now, but previously we… in Group discussion / 3:38 ¶ 22, Of course having organized maternal data can also promote contacting a… in Group discussion / 3:41 ¶ 26, : It is a big opportunity to meet the patient again virtually especial… in Group discussion / 3:42 ¶ 26, Traditionally, when errors happen or if the patients go with the wrong… in Group discussion / 3:43 ¶ 28, At the end this is very important piece of technology that can reduce… in Group discussion / 3:46 ¶ 28, Those infrastructure and awareness creation in staffs, mothers, and co… in Group discussion / 3:52 ¶ 36, People will enjoy talking to others how health institution just concer… in Group discussion / 3:57 ¶ 42, Improve work environment as per the need of the technology and owning… in Group discussion / 3:58 ¶ 43, Aspiring more technologies and helping mothers, taking trainings, givi… in Group discussion / 3:60 ¶ 46, Experience with technology. Enable them to identify areas where mHealt… in Group discussion / 3:61 ¶ 47, Planning, decision making based on mHealth, and increased service take… in Group discussion / 3:62 ¶ 48, Support related to mHealth, fulfillment of deficiencies of human and o… in Group discussion

3:27 ¶ 16 in Group discussion

Text quotation

Created by Girma Gilano on 3/9/2023

It can make the child health growth, for mothers to know danger signs, and during PNC to keep herself from unnecessary early pregnancy. It has no problem as long as the message receiver is the mother or someone oriented on the issue

2 Codes:

- **Benefits of mHealth: ALarming**

Comment: by Girma Gilano

> *3/12/2023 11:46:46 AM, merged with Effectiviness 3/12/2023 1:12:09 PM, merged with Decision_making 3/12/2023 1:12:55 PM, merged with Role_improvement*

6 Quotations:

1:10 ¶ 24, it helps when people are busy with their work to remember appointments in interview analysis / 1:25 ¶ 35, Mothers may not miss their appointment because they forget i in interview analysis / 1:53 ¶ 59, I think mHealth can improve access, appointment on time, availability… in interview analysis / 1:68 ¶ 70, Every system is getting digitalized so digitalizing health may have mu… in interview analysis / 3:27 ¶ 16, It can make the child health growth, for mothers to know danger signs,… in Group discussion / 3:47 ¶ 30, Appointment reminder, service on time, and reduce mortality, but befor… in Group discussion

- **Benefits of mHealth: Help_mothers**

Comment: by Girma Gilano

> *3/12/2023 11:46:46 AM, merged with Effectiviness 3/12/2023 1:12:09 PM, merged with Decision_making 3/12/2023 1:12:55 PM, merged with Role_improvement*

27 Quotations:

1:41 ¶ 47, ion comes after understanding of benefits and harms in interview analysis / 1:45 ¶ 52, Accessibility can be improved mothers get information at their homes in interview analysis / 1:51 ¶ 58, She can access any information in interview analysis / 1:53 ¶ 59, I think mHealth can improve access, appointment on time, availability… in interview analysis / 1:65 ¶ 67, Her husband or children will also push her to go even if the condition… in interview analysis / 1:68 ¶ 70, Every system is getting digitalized

so digitalizing health may have mu… in interview analysis / 1:77 ¶ 77, Rural mothers are respectful; they do what they told to do, so fear of… in interview analysis / 1:81 ¶ 80, pe mothers will turn to use mobiles, but only after getting its import… in interview analysis / 2:3 ¶ 11, It is helpful to also send information which are not appropriate in pe… in KII / 2:7 ¶ 17, Yeah, it will have effective change on community in KII / 2:9 ¶ 19, Yeah, previously mothers use the information they get during ANC, but… in KII / 2:11 ¶ 21, Information access, no transportation cost, and no professional resour… in KII / 2:36 ¶ 55, No need of motor or vehicles because it is mHealth. I will be happy to… in KII / 3:6 ¶ 5, They difference for mHealth is mother always get counseling or health… in Group discussion / 3:18 ¶ 12, It looks like mothers are tired of current service provision so the mH… in Group discussion / 3:23 ¶ 14, Previously, people think poor health system and there are many complia… in Group discussion / 3:24 ¶ 14, Over time this can change community perception and they may reebok mot… in Group discussion / 3:25 ¶ 14, There may be emerged women who are successful because of mHealth and t… in Group discussion / 3:27 ¶ 16, It can make the child health growth, for mothers to know danger signs,… in Group discussion / 3:38 ¶ 22, Of course having organized maternal data can also promote contacting a… in Group discussion / 3:41 ¶ 26, : It is a big opportunity to meet the patient again virtually especial… in Group discussion / 3:42 ¶ 26, Traditionally, when errors happen or if the patients go with the wrong… in Group discussion / 3:50 ¶ 33, Benefit for mothers for next pregnancies and healthy prospect for chil… in Group discussion / 3:59 ¶ 45, Satisfaction of their client service and familiarization with technolo… in Group discussion / 3:61 ¶ 47, Planning, decision making based on mHealth, and increased service take… in Group discussion / 3:62 ¶ 48, Support related to mHealth, fulfillment of deficiencies of human and o… in Group discussion / 3:67 ¶ 53, he respect for pregnancy mothers may think gave many births and not ca… in Group discussion

3:38 ¶ 22 in Group discussion

Text quotation

Created by Girma Gilano on 3/9/2023

Of course having organized maternal data can also promote contacting atleast risky mothers even without funds.

2 Codes:

- **Benefits of mHealth: Help_mothers**

Comment: by Girma Gilano

> *3/12/2023 11:46:46 AM, merged with Effectiviness 3/12/2023 1:12:09 PM, merged with Decision_making 3/12/2023 1:12:55 PM, merged with Role_improvement*

27 Quotations:

1:41 ¶ 47, ion comes after understanding of benefits and harms in interview analysis / 1:45 ¶ 52, Accessibility can be improved mothers get information at their homes in interview analysis / 1:51 ¶ 58, She can access any information in interview analysis / 1:53 ¶ 59, I think mHealth can improve access, appointment on time, availability… in interview analysis / 1:65 ¶ 67, Her husband or children will also push her to go even if the condition… in interview analysis / 1:68 ¶ 70, Every system is getting digitalized so digitalizing health may have mu… in interview analysis / 1:77 ¶ 77, Rural mothers are respectful; they do what they told to do, so fear of… in interview analysis / 1:81 ¶ 80, pe mothers will turn to use mobiles, but only after getting its import… in interview analysis / 2:3 ¶ 11, It is helpful to also send information which are not appropriate in pe… in KII / 2:7 ¶ 17, Yeah, it will have effective change on community in KII / 2:9 ¶ 19, Yeah, previously mothers use the information they get during ANC, but… in KII / 2:11 ¶ 21, Information access, no transportation cost, and no professional resour… in KII / 2:36 ¶ 55, No need of motor or vehicles because it is mHealth. I will be happy to… in KII / 3:6 ¶ 5, They difference for mHealth is mother always get counseling or health… in Group discussion / 3:18 ¶ 12, It looks like mothers are tired of current service provision so the mH… in Group discussion / 3:23 ¶ 14, Previously, people think poor health system and there are many complia… in Group discussion / 3:24 ¶ 14, Over time this can change community perception and they may reebok mot… in Group discussion / 3:25 ¶ 14, There may be emerged women who are successful because of mHealth and t… in Group discussion / 3:27 ¶ 16, It

can make the child health growth, for mothers to know danger signs,… in Group discussion / 3:38 ¶ 22, Of course having organized maternal data can also promote contacting a… in Group discussion / 3:41 ¶ 26, : It is a big opportunity to meet the patient again virtually especial… in Group discussion / 3:42 ¶ 26, Traditionally, when errors happen or if the patients go with the wrong… in Group discussion / 3:50 ¶ 33, Benefit for mothers for next pregnancies and healthy prospect for chil… in Group discussion / 3:59 ¶ 45, Satisfaction of their client service and familiarization with technolo… in Group discussion / 3:61 ¶ 47, Planning, decision making based on mHealth, and increased service take… in Group discussion / 3:62 ¶ 48, Support related to mHealth, fulfillment of deficiencies of human and o… in Group discussion / 3:67 ¶ 53, he respect for pregnancy mothers may think gave many births and not ca… in Group discussion

● Benefits of mHealth: Help_professional

Comment: by Girma Gilano

> *3/12/2023 11:46:46 AM, merged with Effectiviness 3/12/2023 1:12:09 PM, merged with Decision_making 3/12/2023 1:12:55 PM, merged with Role_improvement*

30 Quotations:

1:24 ¶ 34, We always give best service to our best capacity, but mHealth may adva… in interview analysis / 1:34 ¶ 39, As HEWs we cannot reach for every mother on time but text can easily r… in interview analysis / 1:36 ¶ 40, This mHealth better than our previous our service in interview analysis / 1:50 ¶ 57, further improve the already improving service in interview analysis / 1:56 ¶ 62, Our current ANC and other formats now request mother to register their… in interview analysis / 1:68 ¶ 70, Every system is getting digitalized so digitalizing health may have mu… in interview analysis / 1:82 ¶ 81, ers usually take what we tell them, it will be successful. I don’t thi… in interview analysis / 2:3 ¶ 11, It is helpful to also send information which are not appropriate in pe… in KII / 2:7 ¶ 17, Yeah, it will have effective change on community in KII / 2:9 ¶ 19, Yeah, previously mothers use the information they get during ANC, but… in KII / 2:35 ¶ 54, Since we have many gaps this should be seen as an opportunity in KII / 3:2 ¶ 3, Previous teaching of mother through home to home is going to be throug… in Group discussion / 3:12 ¶ 8, For educated mothers messages will be option less in Group discussion / 3:13 ¶ 8, Starting from me we feed additional food at 2months because the kind o… in Group discussion / 3:18 ¶ 12, It looks like mothers are tired of current service provision so the mH… in Group discussion / 3:25 ¶ 14, There may be emerged women who are successful because of mHealth and t… in Group discussion / 3:29 ¶ 17, As a health professionals applying technology can only easy our burden… in Group discussion / 3:34 ¶ 19, ecause of mHealth we may every address of women and now we can also ca… in Group discussion / 3:35 ¶ 20, We will provide the service for mothers we know now, but previously we… in Group discussion / 3:38 ¶ 22, Of course having organized maternal data can also promote contacting a… in Group discussion / 3:41 ¶ 26, : It is a big opportunity to meet the patient again virtually especial… in Group discussion / 3:42 ¶ 26, Traditionally, when errors happen or if the patients go with the wrong… in Group discussion / 3:43 ¶ 28, At the end this is very important piece of technology that can reduce… in Group discussion / 3:46 ¶ 28, Those infrastructure and awareness creation in staffs, mothers, and co… in Group discussion / 3:52 ¶ 36, People will enjoy talking to others how health institution just concer… in Group discussion / 3:57 ¶ 42, Improve work environment as per the need of the technology and owning… in Group discussion / 3:58 ¶ 43, Aspiring more technologies and helping mothers, taking trainings, givi… in Group discussion / 3:60 ¶ 46, Experience with technology. Enable them to identify areas where mHealt… in Group discussion / 3:61 ¶ 47, Planning, decision making based on mHealth, and increased service take… in Group discussion / 3:62 ¶ 48, Support related to mHealth, fulfillment of deficiencies of human and o… in Group discussion

3:41 ¶ 26 in Group discussion

Text quotation

Created by Girma Gilano on 3/9/2023, **modified** by Girma Gilano on 3/9/2023

: It is a big opportunity to meet the patient again virtually especially when you missed something in person. Consultation normally should not be a onetime activity rather an effective consultation should be continuous.

2 Codes:

● Benefits of mHealth: Help_mothers

Comment: by Girma Gilano

> *3/12/2023 11:46:46 AM, merged with Effectiviness 3/12/2023 1:12:09 PM, merged with Decision_making 3/12/2023 1:12:55 PM, merged with Role_improvement*

27 Quotations:

1:41 ¶ 47, ion comes after understanding of benefits and harms in interview analysis / 1:45 ¶ 52, Accessibility can be improved mothers get information at their homes in interview analysis / 1:51 ¶ 58, She can access any information in interview analysis / 1:53 ¶ 59, I think mHealth can improve access, appointment on time, availability… in interview analysis / 1:65 ¶ 67, Her husband or children will also push her to go even if the condition… in interview analysis / 1:68 ¶ 70, Every system is getting digitalized so digitalizing health may have mu… in interview analysis / 1:77 ¶ 77, Rural mothers are respectful; they do what they told to do, so fear of… in interview analysis / 1:81 ¶ 80, pe mothers will turn to use mobiles, but only after getting its import… in interview analysis / 2:3 ¶ 11, It is helpful to also send information which are not appropriate in pe… in KII / 2:7 ¶ 17, Yeah, it will have effective change on community in KII / 2:9 ¶ 19, Yeah, previously mothers use the information they get during ANC, but… in KII / 2:11 ¶ 21, Information access, no transportation cost, and no professional resour… in KII / 2:36 ¶ 55, No need of motor or vehicles because it is mHealth. I will be happy to… in KII / 3:6 ¶ 5, They difference for mHealth is mother always get counseling or health… in Group discussion / 3:18 ¶ 12, It looks like mothers are tired of current service provision so the mH… in Group discussion / 3:23 ¶ 14, Previously, people think poor health system and there are many complia… in Group discussion / 3:24 ¶ 14, Over time this can change community perception and they may reebok mot… in Group discussion / 3:25 ¶ 14, There may be emerged women who are successful because of mHealth and t… in Group discussion / 3:27 ¶ 16, It can make the child health growth, for mothers to know danger signs,… in Group discussion / 3:38 ¶ 22, Of course having organized maternal data can also promote contacting a… in Group discussion / 3:41 ¶ 26, : It is a big opportunity to meet the patient again virtually especial… in Group discussion / 3:42 ¶ 26, Traditionally, when errors happen or if the patients go with the wrong… in Group discussion / 3:50 ¶ 33, Benefit for mothers for next pregnancies and healthy prospect for chil… in Group discussion / 3:59 ¶ 45, Satisfaction of their client service and familiarization with technolo… in Group discussion / 3:61 ¶ 47, Planning, decision making based on mHealth, and increased service take… in Group discussion / 3:62 ¶ 48, Support related to mHealth, fulfillment of deficiencies of human and o… in Group discussion / 3:67 ¶ 53, he respect for pregnancy mothers may think gave many births and not ca… in Group discussion

● Benefits of mHealth: Help_professional

Comment: by Girma Gilano

> *3/12/2023 11:46:46 AM, merged with Effectiviness 3/12/2023 1:12:09 PM, merged with Decision_making 3/12/2023 1:12:55 PM, merged with Role_improvement*

30 Quotations:

1:24 ¶ 34, We always give best service to our best capacity, but mHealth may adva… in interview analysis / 1:34 ¶ 39, As HEWs we cannot reach for every mother on time but text can easily r… in interview analysis / 1:36 ¶ 40, This mHealth better than our previous our service in interview analysis / 1:50 ¶ 57, further improve the already improving service in interview analysis / 1:56 ¶ 62, Our current ANC and other formats now request mother to register their… in interview analysis / 1:68 ¶ 70, Every system is getting digitalized so digitalizing health may have mu… in interview analysis / 1:82 ¶ 81, ers usually take what we tell them, it will be successful. I don't thi… in interview analysis / 2:3 ¶ 11, It is helpful to also send information which are not appropriate in pe… in KII / 2:7 ¶ 17, Yeah, it will have effective change on community in KII / 2:9 ¶ 19, Yeah, previously mothers use the information they get during ANC, but… in KII / 2:35 ¶ 54, Since we have many gaps this should be seen as an opportunity in KII / 3:2 ¶ 3, Previous teaching of mother through home to home is going to be throug… in Group discussion / 3:12 ¶ 8, For educated mothers messages will be option less in Group discussion / 3:13 ¶ 8, Starting from me we feed additional food at 2months because the kind o… in Group discussion / 3:18 ¶ 12, It looks like mothers are tired of current service provision so the mH… in Group discussion / 3:25 ¶

14, There may be emerged women who are successful because of mHealth and t… in Group discussion / 3:29 ¶ 17, As a health professionals applying technology can only easy our burden… in Group discussion / 3:34 ¶ 19, ecause of mHealth we may every address of women and now we can also ca… in Group discussion / 3:35 ¶ 20, We will provide the service for mothers we know now, but previously we… in Group discussion / 3:38 ¶ 22, Of course having organized maternal data can also promote contacting a… in Group discussion / 3:41 ¶ 26, : It is a big opportunity to meet the patient again virtually especial… in Group discussion / 3:42 ¶ 26, Traditionally, when errors happen or if the patients go with the wrong… in Group discussion / 3:43 ¶ 28, At the end this is very important piece of technology that can reduce… in Group discussion / 3:46 ¶ 28, Those infrastructure and awareness creation in staffs, mothers, and co… in Group discussion / 3:52 ¶ 36, People will enjoy talking to others how health institution just concer… in Group discussion / 3:57 ¶ 42, Improve work environment as per the need of the technology and owning… in Group discussion / 3:58 ¶ 43, Aspiring more technologies and helping mothers, taking trainings, givi… in Group discussion / 3:60 ¶ 46, Experience with technology. Enable them to identify areas where mHealt… in Group discussion / 3:61 ¶ 47, Planning, decision making based on mHealth, and increased service take… in Group discussion / 3:62 ¶ 48, Support related to mHealth, fulfillment of deficiencies of human and o… in Group discussion

3:42 ¶ 26 in Group discussion

Text quotation

Created by Girma Gilano on 3/9/2023

Traditionally, when errors happen or if the patients go with the wrong drug there is no way to correct it back. It means the patient have to suffer the error and then come back if alive, but this time we have organized evidence and further messages

2 Codes:

- **Benefits of mHealth: Help_mothers**

Comment: by Girma Gilano

> *3/12/2023 11:46:46 AM, merged with Effectiviness 3/12/2023 1:12:09 PM, merged with Decision_making 3/12/2023 1:12:55 PM, merged with Role_improvement*

27 Quotations:

1:41 ¶ 47, ion comes after understanding of benefits and harms in interview analysis / 1:45 ¶ 52, Accessibility can be improved mothers get information at their homes in interview analysis / 1:51 ¶ 58, She can access any information in interview analysis / 1:53 ¶ 59, I think mHealth can improve access, appointment on time, availability… in interview analysis / 1:65 ¶ 67, Her husband or children will also push her to go even if the condition… in interview analysis / 1:68 ¶ 70, Every system is getting digitalized so digitalizing health may have mu… in interview analysis / 1:77 ¶ 77, Rural mothers are respectful; they do what they told to do, so fear of… in interview analysis / 1:81 ¶ 80, pe mothers will turn to use mobiles, but only after getting its import… in interview analysis / 2:3 ¶ 11, It is helpful to also send information which are not appropriate in pe… in KII / 2:7 ¶ 17, Yeah, it will have effective change on community in KII / 2:9 ¶ 19, Yeah, previously mothers use the information they get during ANC, but… in KII / 2:11 ¶ 21, Information access, no transportation cost, and no professional resour… in KII / 2:36 ¶ 55, No need of motor or vehicles because it is mHealth. I will be happy to… in KII / 3:6 ¶ 5, They difference for mHealth is mother always get counseling or health… in Group discussion / 3:18 ¶ 12, It looks like mothers are tired of current service provision so the mH… in Group discussion / 3:23 ¶ 14, Previously, people think poor health system and there are many complia… in Group discussion / 3:24 ¶ 14, Over time this can change community perception and they may reebok mot… in Group discussion / 3:25 ¶ 14, There may be emerged women who are successful because of mHealth and t… in Group discussion / 3:27 ¶ 16, It can make the child health growth, for mothers to know danger signs,… in Group discussion / 3:38 ¶ 22, Of course having organized maternal data can also promote contacting a… in Group discussion / 3:41 ¶ 26, : It is a big opportunity to meet the patient again virtually especial… in Group discussion / 3:42 ¶ 26, Traditionally, when errors happen or if the patients go with the wrong… in Group discussion / 3:50 ¶ 33,

Benefit for mothers for next pregnancies and healthy prospect for chil… in Group discussion / 3:59 ¶ 45, Satisfaction of their client service and familiarization with technolo… in Group discussion / 3:61 ¶ 47, Planning, decision making based on mHealth, and increased service take… in Group discussion / 3:62 ¶ 48, Support related to mHealth, fulfillment of deficiencies of human and o… in Group discussion / 3:67 ¶ 53, he respect for pregnancy mothers may think gave many births and not ca… in Group discussion

- **Benefits of mHealth: Help_professional**

Comment: by Girma Gilano

> *3/12/2023 11:46:46 AM, merged with Effectiviness 3/12/2023 1:12:09 PM, merged with Decision_making 3/12/2023 1:12:55 PM, merged with Role_improvement*

30 Quotations:

1:24 ¶ 34, We always give best service to our best capacity, but mHealth may adva… in interview analysis / 1:34 ¶ 39, As HEWs we cannot reach for every mother on time but text can easily r… in interview analysis / 1:36 ¶ 40, This mHealth better than our previous our service in interview analysis / 1:50 ¶ 57, further improve the already improving service in interview analysis / 1:56 ¶ 62, Our current ANC and other formats now request mother to register their… in interview analysis / 1:68 ¶ 70, Every system is getting digitalized so digitalizing health may have mu… in interview analysis / 1:82 ¶ 81, ers usually take what we tell them, it will be successful. I don't thi… in interview analysis / 2:3 ¶ 11, It is helpful to also send information which are not appropriate in pe… in KII / 2:7 ¶ 17, Yeah, it will have effective change on community in KII / 2:9 ¶ 19, Yeah, previously mothers use the information they get during ANC, but… in KII / 2:35 ¶ 54, Since we have many gaps this should be seen as an opportunity in KII / 3:2 ¶ 3, Previous teaching of mother through home to home is going to be throug… in Group discussion / 3:12 ¶ 8, For educated mothers messages will be option less in Group discussion / 3:13 ¶ 8, Starting from me we feed additional food at 2months because the kind o… in Group discussion / 3:18 ¶ 12, It looks like mothers are tired of current service provision so the mH… in Group discussion / 3:25 ¶ 14, There may be emerged women who are successful because of mHealth and t… in Group discussion / 3:29 ¶ 17, As a health professionals applying technology can only easy our burden… in Group discussion / 3:34 ¶ 19, ecause of mHealth we may every address of women and now we can also ca… in Group discussion / 3:35 ¶ 20, We will provide the service for mothers we know now, but previously we… in Group discussion / 3:38 ¶ 22, Of course having organized maternal data can also promote contacting a… in Group discussion / 3:41 ¶ 26, : It is a big opportunity to meet the patient again virtually especial… in Group discussion / 3:42 ¶ 26, Traditionally, when errors happen or if the patients go with the wrong… in Group discussion / 3:43 ¶ 28, At the end this is very important piece of technology that can reduce… in Group discussion / 3:46 ¶ 28, Those infrastructure and awareness creation in staffs, mothers, and co… in Group discussion / 3:52 ¶ 36, People will enjoy talking to others how health institution just concer… in Group discussion / 3:57 ¶ 42, Improve work environment as per the need of the technology and owning… in Group discussion / 3:58 ¶ 43, Aspiring more technologies and helping mothers, taking trainings, givi… in Group discussion / 3:60 ¶ 46, Experience with technology. Enable them to identify areas where mHealt… in Group discussion / 3:61 ¶ 47, Planning, decision making based on mHealth, and increased service take… in Group discussion / 3:62 ¶ 48, Support related to mHealth, fulfillment of deficiencies of human and o… in Group discussion

3:50 ¶ 33 in Group discussion

Text quotation

Created by Girma Gilano on 3/9/2023

Benefit for mothers for next pregnancies and healthy prospect for children

1 Codes:

- **Benefits of mHealth: Help_mothers**

Comment: by Girma Gilano

> *3/12/2023 11:46:46 AM, merged with Effectiviness 3/12/2023 1:12:09 PM, merged with Decision_making 3/12/2023 1:12:55 PM, merged with Role_improvement*

27 Quotations:

1:41 ¶ 47, ion comes after understanding of benefits and harms in interview analysis / 1:45 ¶ 52, Accessibility can be improved mothers get information at their homes in interview analysis / 1:51 ¶ 58, She can access any information in interview analysis / 1:53 ¶ 59, I think mHealth can improve access, appointment on time, availability… in interview analysis / 1:65 ¶ 67, Her husband or children will also push her to go even if the condition… in interview analysis / 1:68 ¶ 70, Every system is getting digitalized so digitalizing health may have mu… in interview analysis / 1:77 ¶ 77, Rural mothers are respectful; they do what they told to do, so fear of… in interview analysis / 1:81 ¶ 80, pe mothers will turn to use mobiles, but only after getting its import… in interview analysis / 2:3 ¶ 11, It is helpful to also send information which are not appropriate in pe… in KII / 2:7 ¶ 17, Yeah, it will have effective change on community in KII / 2:9 ¶ 19, Yeah, previously mothers use the information they get during ANC, but… in KII / 2:11 ¶ 21, Information access, no transportation cost, and no professional resour… in KII / 2:36 ¶ 55, No need of motor or vehicles because it is mHealth. I will be happy to… in KII / 3:6 ¶ 5, They difference for mHealth is mother always get counseling or health… in Group discussion / 3:18 ¶ 12, It looks like mothers are tired of current service provision so the mH… in Group discussion / 3:23 ¶ 14, Previously, people think poor health system and there are many complia… in Group discussion / 3:24 ¶ 14, Over time this can change community perception and they may reebok mot… in Group discussion / 3:25 ¶ 14, There may be emerged women who are successful because of mHealth and t… in Group discussion / 3:27 ¶ 16, It can make the child health growth, for mothers to know danger signs,… in Group discussion / 3:38 ¶ 22, Of course having organized maternal data can also promote contacting a… in Group discussion / 3:41 ¶ 26, : It is a big opportunity to meet the patient again virtually especial… in Group discussion / 3:42 ¶ 26, Traditionally, when errors happen or if the patients go with the wrong… in Group discussion / 3:50 ¶ 33, Benefit for mothers for next pregnancies and healthy prospect for chil… in Group discussion / 3:59 ¶ 45, Satisfaction of their client service and familiarization with technolo… in Group discussion / 3:61 ¶ 47, Planning, decision making based on mHealth, and increased service take… in Group discussion / 3:62 ¶ 48, Support related to mHealth, fulfillment of deficiencies of human and o… in Group discussion / 3:67 ¶ 53, he respect for pregnancy mothers may think gave many births and not ca… in Group discussion

3:59 ¶ 45 in Group discussion

Text quotation

Created by Girma Gilano on 3/9/2023

Satisfaction of their client service and familiarization with technolog

1 Codes:

- **Benefits of mHealth: Help_mothers**

Comment: by Girma Gilano

> *3/12/2023 11:46:46 AM, merged with Effectiviness 3/12/2023 1:12:09 PM, merged with Decision_making 3/12/2023 1:12:55 PM, merged with Role_improvement*

27 Quotations:

1:41 ¶ 47, ion comes after understanding of benefits and harms in interview analysis / 1:45 ¶ 52, Accessibility can be improved mothers get information at their homes in interview analysis / 1:51 ¶ 58, She can access any information in interview analysis / 1:53 ¶ 59, I think mHealth can improve access, appointment on time, availability… in interview analysis / 1:65 ¶ 67, Her husband or children will also push her to go even if the condition… in interview analysis / 1:68 ¶ 70, Every system is getting digitalized so digitalizing health may have mu… in interview analysis / 1:77 ¶ 77, Rural mothers are respectful; they do what they told to do, so fear of… in interview analysis / 1:81 ¶ 80, pe mothers will turn to use mobiles, but only after getting its import… in interview analysis / 2:3 ¶ 11, It is helpful to also send information

which are not appropriate in pe… in KII / 2:7 ¶ 17, Yeah, it will have effective change on community in KII / 2:9 ¶ 19, Yeah, previously mothers use the information they get during ANC, but… in KII / 2:11 ¶ 21, Information access, no transportation cost, and no professional resour… in KII / 2:36 ¶ 55, No need of motor or vehicles because it is mHealth. I will be happy to… in KII / 3:6 ¶ 5, They difference for mHealth is mother always get counseling or health… in Group discussion / 3:18 ¶ 12, It looks like mothers are tired of current service provision so the mH… in Group discussion / 3:23 ¶ 14, Previously, people think poor health system and there are many complia… in Group discussion / 3:24 ¶ 14, Over time this can change community perception and they may reebok mot… in Group discussion / 3:25 ¶ 14, There may be emerged women who are successful because of mHealth and t… in Group discussion / 3:27 ¶ 16, It can make the child health growth, for mothers to know danger signs,… in Group discussion / 3:38 ¶ 22, Of course having organized maternal data can also promote contacting a… in Group discussion / 3:41 ¶ 26, : It is a big opportunity to meet the patient again virtually especial… in Group discussion / 3:42 ¶ 26, Traditionally, when errors happen or if the patients go with the wrong… in Group discussion / 3:50 ¶ 33, Benefit for mothers for next pregnancies and healthy prospect for chil… in Group discussion / 3:59 ¶ 45, Satisfaction of their client service and familiarization with technolo… in Group discussion / 3:61 ¶ 47, Planning, decision making based on mHealth, and increased service take… in Group discussion / 3:62 ¶ 48, Support related to mHealth, fulfillment of deficiencies of human and o… in Group discussion / 3:67 ¶ 53, he respect for pregnancy mothers may think gave many births and not ca… in Group discussion

3:61 ¶ 47 in Group discussion

Text quotation

Created by Girma Gilano on 3/9/2023

Planning, decision making based on mHealth, and increased service takers

2 Codes:

- **Benefits of mHealth: Help_mothers**

Comment: by Girma Gilano

> *3/12/2023 11:46:46 AM, merged with Effectiviness 3/12/2023 1:12:09 PM, merged with Decision_making 3/12/2023 1:12:55 PM, merged with Role_improvement*

27 Quotations:

1:41 ¶ 47, ion comes after understanding of benefits and harms in interview analysis / 1:45 ¶ 52, Accessibility can be improved mothers get information at their homes in interview analysis / 1:51 ¶ 58, She can access any information in interview analysis / 1:53 ¶ 59, I think mHealth can improve access, appointment on time, availability… in interview analysis / 1:65 ¶ 67, Her husband or children will also push her to go even if the condition… in interview analysis / 1:68 ¶ 70, Every system is getting digitalized so digitalizing health may have mu… in interview analysis / 1:77 ¶ 77, Rural mothers are respectful; they do what they told to do, so fear of… in interview analysis / 1:81 ¶ 80, pe mothers will turn to use mobiles, but only after getting its import… in interview analysis / 2:3 ¶ 11, It is helpful to also send information which are not appropriate in pe… in KII / 2:7 ¶ 17, Yeah, it will have effective change on community in KII / 2:9 ¶ 19, Yeah, previously mothers use the information they get during ANC, but… in KII / 2:11 ¶ 21, Information access, no transportation cost, and no professional resour… in KII / 2:36 ¶ 55, No need of motor or vehicles because it is mHealth. I will be happy to… in KII / 3:6 ¶ 5, They difference for mHealth is mother always get counseling or health… in Group discussion / 3:18 ¶ 12, It looks like mothers are tired of current service provision so the mH… in Group discussion / 3:23 ¶ 14, Previously, people think poor health system and there are many complia… in Group discussion / 3:24 ¶ 14, Over time this can change community perception and they may reebok mot… in Group discussion / 3:25 ¶ 14, There may be emerged women who are successful because of mHealth and t… in Group discussion / 3:27 ¶ 16, It can make the child health growth, for mothers to know danger signs,… in Group discussion / 3:38 ¶ 22, Of course having organized maternal data can also promote contacting a… in Group discussion / 3:41 ¶ 26, : It is a big opportunity to meet the patient again virtually especial… in Group discussion / 3:42 ¶ 26, Traditionally, when errors happen or if the patients go with the wrong… in Group discussion / 3:50 ¶ 33,

Benefit for mothers for next pregnancies and healthy prospect for chil… in Group discussion / 3:59 ¶ 45, Satisfaction of their client service and familiarization with technolo… in Group discussion / 3:61 ¶ 47, Planning, decision making based on mHealth, and increased service take… in Group discussion / 3:62 ¶ 48, Support related to mHealth, fulfillment of deficiencies of human and o… in Group discussion / 3:67 ¶ 53, he respect for pregnancy mothers may think gave many births and not ca… in Group discussion

Benefits of mHealth: Help_professional

Comment: by Girma Gilano

> *3/12/2023 11:46:46 AM, merged with Effectiviness 3/12/2023 1:12:09 PM, merged with Decision_making 3/12/2023 1:12:55 PM, merged with Role_improvement*

30 Quotations:

1:24 ¶ 34, We always give best service to our best capacity, but mHealth may adva… in interview analysis / 1:34 ¶ 39, As HEWs we cannot reach for every mother on time but text can easily r… in interview analysis / 1:36 ¶ 40, This mHealth better than our previous our service in interview analysis / 1:50 ¶ 57, further improve the already improving service in interview analysis / 1:56 ¶ 62, Our current ANC and other formats now request mother to register their… in interview analysis / 1:68 ¶ 70, Every system is getting digitalized so digitalizing health may have mu… in interview analysis / 1:82 ¶ 81, ers usually take what we tell them, it will be successful. I don't thi… in interview analysis / 2:3 ¶ 11, It is helpful to also send information which are not appropriate in pe… in KII / 2:7 ¶ 17, Yeah, it will have effective change on community in KII / 2:9 ¶ 19, Yeah, previously mothers use the information they get during ANC, but… in KII / 2:35 ¶ 54, Since we have many gaps this should be seen as an opportunity in KII / 3:2 ¶ 3, Previous teaching of mother through home to home is going to be throug… in Group discussion / 3:12 ¶ 8, For educated mothers messages will be option less in Group discussion / 3:13 ¶ 8, Starting from me we feed additional food at 2months because the kind o… in Group discussion / 3:18 ¶ 12, It looks like mothers are tired of current service provision so the mH… in Group discussion / 3:25 ¶ 14, There may be emerged women who are successful because of mHealth and t… in Group discussion / 3:29 ¶ 17, As a health professionals applying technology can only easy our burden… in Group discussion / 3:34 ¶ 19, ecause of mHealth we may every address of women and now we can also ca… in Group discussion / 3:35 ¶ 20, We will provide the service for mothers we know now, but previously we… in Group discussion / 3:38 ¶ 22, Of course having organized maternal data can also promote contacting a… in Group discussion / 3:41 ¶ 26, : It is a big opportunity to meet the patient again virtually especial… in Group discussion / 3:42 ¶ 26, Traditionally, when errors happen or if the patients go with the wrong… in Group discussion / 3:43 ¶ 28, At the end this is very important piece of technology that can reduce… in Group discussion / 3:46 ¶ 28, Those infrastructure and awareness creation in staffs, mothers, and co… in Group discussion / 3:52 ¶ 36, People will enjoy talking to others how health institution just concer… in Group discussion / 3:57 ¶ 42, Improve work environment as per the need of the technology and owning… in Group discussion / 3:58 ¶ 43, Aspiring more technologies and helping mothers, taking trainings, givi… in Group discussion / 3:60 ¶ 46, Experience with technology. Enable them to identify areas where mHealt… in Group discussion / 3:61 ¶ 47, Planning, decision making based on mHealth, and increased service take… in Group discussion / 3:62 ¶ 48, Support related to mHealth, fulfillment of deficiencies of human and o… in Group discussion

3:62 ¶ 48 in Group discussion

Text quotation

Created by Girma Gilano on 3/9/2023

Support related to mHealth, fulfillment of deficiencies of human and other resource limitations, fulfilling the maternal and child health vision, and respect from the community. Acceptance from the community

2 Codes:

Benefits of mHealth: Help_mothers

Comment: by Girma Gilano

> *3/12/2023 11:46:46 AM, merged with Effectiviness 3/12/2023 1:12:09 PM, merged with Decision_making 3/12/2023 1:12:55 PM, merged with Role_improvement*

27 Quotations:

1:41 ¶ 47, ion comes after understanding of benefits and harms in interview analysis / 1:45 ¶ 52, Accessibility can be improved mothers get information at their homes in interview analysis / 1:51 ¶ 58, She can access any information in interview analysis / 1:53 ¶ 59, I think mHealth can improve access, appointment on time, availability… in interview analysis / 1:65 ¶ 67, Her husband or children will also push her to go even if the condition… in interview analysis / 1:68 ¶ 70, Every system is getting digitalized so digitalizing health may have mu… in interview analysis / 1:77 ¶ 77, Rural mothers are respectful; they do what they told to do, so fear of… in interview analysis / 1:81 ¶ 80, pe mothers will turn to use mobiles, but only after getting its import… in interview analysis / 2:3 ¶ 11, It is helpful to also send information which are not appropriate in pe… in KII / 2:7 ¶ 17, Yeah, it will have effective change on community in KII / 2:9 ¶ 19, Yeah, previously mothers use the information they get during ANC, but… in KII / 2:11 ¶ 21, Information access, no transportation cost, and no professional resour… in KII / 2:36 ¶ 55, No need of motor or vehicles because it is mHealth. I will be happy to… in KII / 3:6 ¶ 5, They difference for mHealth is mother always get counseling or health… in Group discussion / 3:18 ¶ 12, It looks like mothers are tired of current service provision so the mH… in Group discussion / 3:23 ¶ 14, Previously, people think poor health system and there are many complia… in Group discussion / 3:24 ¶ 14, Over time this can change community perception and they may reebok mot… in Group discussion / 3:25 ¶ 14, There may be emerged women who are successful because of mHealth and t… in Group discussion / 3:27 ¶ 16, It can make the child health growth, for mothers to know danger signs,… in Group discussion / 3:38 ¶ 22, Of course having organized maternal data can also promote contacting a… in Group discussion / 3:41 ¶ 26, : It is a big opportunity to meet the patient again virtually especial… in Group discussion / 3:42 ¶ 26, Traditionally, when errors happen or if the patients go with the wrong… in Group discussion / 3:50 ¶ 33, Benefit for mothers for next pregnancies and healthy prospect for chil… in Group discussion / 3:59 ¶ 45, Satisfaction of their client service and familiarization with technolo… in Group discussion / 3:61 ¶ 47, Planning, decision making based on mHealth, and increased service take… in Group discussion / 3:62 ¶ 48, Support related to mHealth, fulfillment of deficiencies of human and o… in Group discussion / 3:67 ¶ 53, he respect for pregnancy mothers may think gave many births and not ca… in Group discussion

Benefits of mHealth: Help_professional

Comment: by Girma Gilano

> *3/12/2023 11:46:46 AM, merged with Effectiviness 3/12/2023 1:12:09 PM, merged with Decision_making 3/12/2023 1:12:55 PM, merged with Role_improvement*

30 Quotations:

1:24 ¶ 34, We always give best service to our best capacity, but mHealth may adva… in interview analysis / 1:34 ¶ 39, As HEWs we cannot reach for every mother on time but text can easily r… in interview analysis / 1:36 ¶ 40, This mHealth better than our previous our service in interview analysis / 1:50 ¶ 57, further improve the already improving service in interview analysis / 1:56 ¶ 62, Our current ANC and other formats now request mother to register their… in interview analysis / 1:68 ¶ 70, Every system is getting digitalized so digitalizing health may have mu… in interview analysis / 1:82 ¶ 81, ers usually take what we tell them, it will be successful. I don’t thi… in interview analysis / 2:3 ¶ 11, It is helpful to also send information which are not appropriate in pe… in KII / 2:7 ¶ 17, Yeah, it will have effective change on community in KII / 2:9 ¶ 19, Yeah, previously mothers use the information they get during ANC, but… in KII / 2:35 ¶ 54, Since we have many gaps this should be seen as an opportunity in KII / 3:2 ¶ 3, Previous teaching of mother through home to home is going to be throug… in Group discussion / 3:12 ¶ 8, For educated mothers messages will be option less in Group discussion / 3:13 ¶ 8, Starting from me we feed additional food at 2months because the kind o… in Group discussion / 3:18 ¶ 12, It looks like mothers are tired of current service provision so the mH… in Group discussion / 3:25 ¶ 14, There may be emerged women who are successful because of mHealth and t… in Group discussion / 3:29 ¶ 17, As a health professionals applying technology can only easy our burden… in Group

discussion / 3:34 ¶ 19, ecause of mHealth we may every address of women and now we can also ca… in Group discussion / 3:35 ¶ 20, We will provide the service for mothers we know now, but previously we… in Group discussion / 3:38 ¶ 22, Of course having organized maternal data can also promote contacting a… in Group discussion / 3:41 ¶ 26, : It is a big opportunity to meet the patient again virtually especial… in Group discussion / 3:42 ¶ 26, Traditionally, when errors happen or if the patients go with the wrong… in Group discussion / 3:43 ¶ 28, At the end this is very important piece of technology that can reduce… in Group discussion / 3:46 ¶ 28, Those infrastructure and awareness creation in staffs, mothers, and co… in Group discussion / 3:52 ¶ 36, People will enjoy talking to others how health institution just concer… in Group discussion / 3:57 ¶ 42, Improve work environment as per the need of the technology and owning… in Group discussion / 3:58 ¶ 43, Aspiring more technologies and helping mothers, taking trainings, givi… in Group discussion / 3:60 ¶ 46, Experience with technology. Enable them to identify areas where mHealt… in Group discussion / 3:61 ¶ 47, Planning, decision making based on mHealth, and increased service take… in Group discussion / 3:62 ¶ 48, Support related to mHealth, fulfillment of deficiencies of human and o… in Group discussion

3:67 ¶ 53 in Group discussion

Text quotation

Created by Girma Gilano on 3/9/2023

he respect for pregnancy mothers may think gave many births and not caring seriously. Need of behavioral change for full considerations

2 Codes:

- **Benefits of mHealth: Help_mothers**

Comment: by Girma Gilano

> *3/12/2023 11:46:46 AM, merged with Effectiviness 3/12/2023 1:12:09 PM, merged with Decision_making 3/12/2023 1:12:55 PM, merged with Role_improvement*

27 Quotations:

1:41 ¶ 47, ion comes after understanding of benefits and harms in interview analysis / 1:45 ¶ 52, Accessibility can be improved mothers get information at their homes in interview analysis / 1:51 ¶ 58, She can access any information in interview analysis / 1:53 ¶ 59, I think mHealth can improve access, appointment on time, availability… in interview analysis / 1:65 ¶ 67, Her husband or children will also push her to go even if the condition… in interview analysis / 1:68 ¶ 70, Every system is getting digitalized so digitalizing health may have mu… in interview analysis / 1:77 ¶ 77, Rural mothers are respectful; they do what they told to do, so fear of… in interview analysis / 1:81 ¶ 80, pe mothers will turn to use mobiles, but only after getting its import… in interview analysis / 2:3 ¶ 11, It is helpful to also send information which are not appropriate in pe… in KII / 2:7 ¶ 17, Yeah, it will have effective change on community in KII / 2:9 ¶ 19, Yeah, previously mothers use the information they get during ANC, but… in KII / 2:11 ¶ 21, Information access, no transportation cost, and no professional resour… in KII / 2:36 ¶ 55, No need of motor or vehicles because it is mHealth. I will be happy to… in KII / 3:6 ¶ 5, They difference for mHealth is mother always get counseling or health… in Group discussion / 3:18 ¶ 12, It looks like mothers are tired of current service provision so the mH… in Group discussion / 3:23 ¶ 14, Previously, people think poor health system and there are many complia… in Group discussion / 3:24 ¶ 14, Over time this can change community perception and they may reebok mot… in Group discussion / 3:25 ¶ 14, There may be emerged women who are successful because of mHealth and t… in Group discussion / 3:27 ¶ 16, It can make the child health growth, for mothers to know danger signs,… in Group discussion / 3:38 ¶ 22, Of course having organized maternal data can also promote contacting a… in Group discussion / 3:41 ¶ 26, : It is a big opportunity to meet the patient again virtually especial… in Group discussion / 3:42 ¶ 26, Traditionally, when errors happen or if the patients go with the wrong… in Group discussion / 3:50 ¶ 33, Benefit for mothers for next pregnancies and healthy prospect for chil… in Group discussion / 3:59 ¶ 45, Satisfaction of their client service and familiarization with technolo… in Group discussion / 3:61 ¶ 47, Planning, decision making based on mHealth, and increased service take… in Group discussion / 3:62 ¶

48, Support related to mHealth, fulfillment of deficiencies of human and o… in Group discussion / 3:67 ¶ 53, he respect for pregnancy mothers may think gave many births and not ca… in Group discussion

Benefits of mHealth: Improve_decision

Comment: by Girma Gilano

> *3/12/2023 11:46:46 AM, merged with Effectiviness 3/12/2023 1:12:09 PM, merged with Decision_making 3/12/2023 1:12:55 PM, merged with Role_improvement*

17 Quotations:

1:5 ¶ 20, t will improve mothers' time wastage for information that they can get… in interview analysis / 1:23 ¶ 33, will be helpful because it will remain in the mother's hand for a long… in interview analysis / 1:40 ¶ 45, s it can improve their decision making. This service could increase wo… in interview analysis / 1:42 ¶ 49, provide information to make decisions, motivate her to convince her hu… in interview analysis / 1:49 ¶ 56, Having information on time can improve the health of mothers and child… in interview analysis / 1:55 ¶ 60, mHealth can provide evidence to make decision and can improve everythi… in interview analysis / 1:64 ¶ 67, However, having this information in her hand will continuously strike… in interview analysis / 1:70 ¶ 71, Service can be improved through mHealth because atleast having informa… in interview analysis / 2:15 ¶ 27, Mothers should understand initially the important, but on our side I d… in KII / 3:6 ¶ 5, They difference for mHealth is mother always get counseling or health… in Group discussion / 3:10 ¶ 8, Mother are already exposed to health education, but this one is direct… in Group discussion / 3:20 ¶ 12, They may even consider the increased risk of danger related to pregnan… in Group discussion / 3:24 ¶ 14, Over time this can change community perception and they may reebok mot… in Group discussion / 3:37 ¶ 20, It can also introduce labor division because we may know who must serv… in Group discussion / 3:48 ¶ 31, Service at their home and Information and on time vaccination and Heal… in Group discussion / 3:52 ¶ 36, People will enjoy talking to others how health institution just concer… in Group discussion / 3:67 ¶ 53, he respect for pregnancy mothers may think gave many births and not ca… in Group discussion

---

Benefits of mHealth: Help_professional

Created by Girma Gilano on 3/13/2023

Comment: by Girma Gilano

> *3/12/2023 11:46:46 AM, merged with Effectiviness 3/12/2023 1:12:09 PM, merged with Decision_making 3/12/2023 1:12:55 PM, merged with Role_improvement*

30 Quotations:

1:24 ¶ 34 in interview analysis

Text quotation

Created by Girma Gilano on 3/8/2023

We always give best service to our best capacity, but mHealth may advance this service

1 Codes:

Benefits of mHealth: Help_professional

Comment: by Girma Gilano

> *3/12/2023 11:46:46 AM, merged with Effectiviness 3/12/2023 1:12:09 PM, merged with Decision_making 3/12/2023 1:12:55 PM, merged with Role_improvement*

30 Quotations:

1:24 ¶ 34, We always give best service to our best capacity, but mHealth may adva… in interview analysis / 1:34 ¶ 39, As HEWs we cannot reach for every mother on time but text can easily r… in interview analysis / 1:36 ¶ 40, This mHealth better than our previous our service in interview analysis / 1:50 ¶ 57, further improve the already improving service in interview analysis / 1:56 ¶ 62, Our current ANC and other formats now request mother to register their… in interview analysis / 1:68 ¶ 70, Every system is getting digitalized so digitalizing health may have mu… in interview analysis / 1:82 ¶ 81, ers usually take what we tell them, it will be successful. I don't thi… in interview analysis / 2:3 ¶ 11, It is helpful to also send information which are not appropriate in pe… in KII / 2:7 ¶ 17, Yeah, it will have effective change on community in KII / 2:9 ¶ 19, Yeah, previously mothers use the information they get during ANC, but… in KII / 2:35 ¶ 54, Since we have many gaps this should be seen as an opportunity in KII / 3:2 ¶ 3, Previous teaching of mother through home to home is going to be throug… in Group discussion / 3:12 ¶ 8, For educated mothers messages will be option less in Group discussion / 3:13 ¶ 8, Starting from me we feed additional food at 2months because the kind o… in Group discussion / 3:18 ¶ 12, It looks like mothers are tired of current service provision so the mH… in Group discussion / 3:25 ¶ 14, There may be emerged women who are successful because of mHealth and t… in Group discussion / 3:29 ¶ 17, As a health professionals applying technology can only easy our burden… in Group discussion / 3:34 ¶ 19, ecause of mHealth we may every address of women and now we can also ca… in Group discussion / 3:35 ¶ 20, We will provide the service for mothers we know now, but previously we… in Group discussion / 3:38 ¶ 22, Of course having organized maternal data can also promote contacting a… in Group discussion / 3:41 ¶ 26, : It is a big opportunity to meet the patient again virtually especial… in Group discussion / 3:42 ¶ 26, Traditionally, when errors happen or if the patients go with the wrong… in Group discussion / 3:43 ¶ 28, At the end this is very important piece of technology that can reduce… in Group discussion / 3:46 ¶ 28, Those infrastructure and awareness creation in staffs, mothers, and co… in Group discussion / 3:52 ¶ 36, People will enjoy talking to others how health institution just concer… in Group discussion / 3:57 ¶ 42, Improve work environment as per the need of the technology and owning… in Group discussion / 3:58 ¶ 43, Aspiring more technologies and helping mothers, taking trainings, givi… in Group discussion / 3:60 ¶ 46, Experience with technology. Enable them to identify areas where mHealt… in Group discussion / 3:61 ¶ 47, Planning, decision making based on mHealth, and increased service take… in Group discussion / 3:62 ¶ 48, Support related to mHealth, fulfillment of deficiencies of human and o… in Group discussion

1:34 ¶ 39 in interview analysis

Text quotation

Created by Girma Gilano on 3/8/2023

As HEWs we cannot reach for every mother on time but text can easily reach to every mother

1 Codes:

- ### Benefits of mHealth: Help_professional

Comment: by Girma Gilano

> *3/12/2023 11:46:46 AM, merged with Effectiviness 3/12/2023 1:12:09 PM, merged with Decision_making 3/12/2023 1:12:55 PM, merged with Role_improvement*

30 Quotations:

1:24 ¶ 34, We always give best service to our best capacity, but mHealth may adva… in interview analysis / 1:34 ¶ 39, As HEWs we cannot reach for every mother on time but text can easily r… in

interview analysis / 1:36 ¶ 40, This mHealth better than our previous our service in interview analysis / 1:50 ¶ 57, further improve the already improving service in interview analysis / 1:56 ¶ 62, Our current ANC and other formats now request mother to register their… in interview analysis / 1:68 ¶ 70, Every system is getting digitalized so digitalizing health may have mu… in interview analysis / 1:82 ¶ 81, ers usually take what we tell them, it will be successful. I don't thi… in interview analysis / 2:3 ¶ 11, It is helpful to also send information which are not appropriate in pe… in KII / 2:7 ¶ 17, Yeah, it will have effective change on community in KII / 2:9 ¶ 19, Yeah, previously mothers use the information they get during ANC, but… in KII / 2:35 ¶ 54, Since we have many gaps this should be seen as an opportunity in KII / 3:2 ¶ 3, Previous teaching of mother through home to home is going to be throug… in Group discussion / 3:12 ¶ 8, For educated mothers messages will be option less in Group discussion / 3:13 ¶ 8, Starting from me we feed additional food at 2months because the kind o… in Group discussion / 3:18 ¶ 12, It looks like mothers are tired of current service provision so the mH… in Group discussion / 3:25 ¶ 14, There may be emerged women who are successful because of mHealth and t… in Group discussion / 3:29 ¶ 17, As a health professionals applying technology can only easy our burden… in Group discussion / 3:34 ¶ 19, ecause of mHealth we may every address of women and now we can also ca… in Group discussion / 3:35 ¶ 20, We will provide the service for mothers we know now, but previously we… in Group discussion / 3:38 ¶ 22, Of course having organized maternal data can also promote contacting a… in Group discussion / 3:41 ¶ 26, : It is a big opportunity to meet the patient again virtually especial… in Group discussion / 3:42 ¶ 26, Traditionally, when errors happen or if the patients go with the wrong… in Group discussion / 3:43 ¶ 28, At the end this is very important piece of technology that can reduce… in Group discussion / 3:46 ¶ 28, Those infrastructure and awareness creation in staffs, mothers, and co… in Group discussion / 3:52 ¶ 36, People will enjoy talking to others how health institution just concer… in Group discussion / 3:57 ¶ 42, Improve work environment as per the need of the technology and owning… in Group discussion / 3:58 ¶ 43, Aspiring more technologies and helping mothers, taking trainings, givi… in Group discussion / 3:60 ¶ 46, Experience with technology. Enable them to identify areas where mHealt… in Group discussion / 3:61 ¶ 47, Planning, decision making based on mHealth, and increased service take… in Group discussion / 3:62 ¶ 48, Support related to mHealth, fulfillment of deficiencies of human and o… in Group discussion

1:36 ¶ 40 in interview analysis

Text quotation

Created by Girma Gilano on 3/8/2023

This mHealth better than our previous our service

2 Codes:

- **Benefits of mHealth: Help_professional**

Comment: by Girma Gilano

> *3/12/2023 11:46:46 AM, merged with Effectiviness 3/12/2023 1:12:09 PM, merged with Decision_making 3/12/2023 1:12:55 PM, merged with Role_improvement*

30 Quotations:

1:24 ¶ 34, We always give best service to our best capacity, but mHealth may adva… in interview analysis / 1:34 ¶ 39, As HEWs we cannot reach for every mother on time but text can easily r… in interview analysis / 1:36 ¶ 40, This mHealth better than our previous our service in interview analysis / 1:50 ¶ 57, further improve the already improving service in interview analysis / 1:56 ¶ 62, Our current ANC and other formats now request mother to register their… in interview analysis / 1:68 ¶ 70, Every system is getting digitalized so digitalizing health may have mu… in interview analysis / 1:82 ¶ 81, ers usually take what we tell them, it will be successful. I don't thi… in interview analysis / 2:3 ¶ 11, It is helpful to also send information which are not appropriate in pe… in KII / 2:7 ¶ 17, Yeah, it will have effective change on community in KII / 2:9 ¶ 19, Yeah, previously mothers use the information they get during ANC, but… in KII / 2:35 ¶ 54, Since we have many gaps this should be seen as an opportunity in KII / 3:2 ¶ 3, Previous teaching of mother through home to home is going to be throug… in Group

discussion / 3:12 ¶ 8, For educated mothers messages will be option less in Group discussion / 3:13 ¶ 8, Starting from me we feed additional food at 2months because the kind o… in Group discussion / 3:18 ¶ 12, It looks like mothers are tired of current service provision so the mH… in Group discussion / 3:25 ¶ 14, There may be emerged women who are successful because of mHealth and t… in Group discussion / 3:29 ¶ 17, As a health professionals applying technology can only easy our burden… in Group discussion / 3:34 ¶ 19, ecause of mHealth we may every address of women and now we can also ca… in Group discussion / 3:35 ¶ 20, We will provide the service for mothers we know now, but previously we… in Group discussion / 3:38 ¶ 22, Of course having organized maternal data can also promote contacting a… in Group discussion / 3:41 ¶ 26, : It is a big opportunity to meet the patient again virtually especial… in Group discussion / 3:42 ¶ 26, Traditionally, when errors happen or if the patients go with the wrong… in Group discussion / 3:43 ¶ 28, At the end this is very important piece of technology that can reduce… in Group discussion / 3:46 ¶ 28, Those infrastructure and awareness creation in staffs, mothers, and co… in Group discussion / 3:52 ¶ 36, People will enjoy talking to others how health institution just concer… in Group discussion / 3:57 ¶ 42, Improve work environment as per the need of the technology and owning… in Group discussion / 3:58 ¶ 43, Aspiring more technologies and helping mothers, taking trainings, givi… in Group discussion / 3:60 ¶ 46, Experience with technology. Enable them to identify areas where mHealt… in Group discussion / 3:61 ¶ 47, Planning, decision making based on mHealth, and increased service take… in Group discussion / 3:62 ¶ 48, Support related to mHealth, fulfillment of deficiencies of human and o… in Group discussion

- **Benefits of mHealth: Improve_MCH**

Comment: by Girma Gilano

> *3/12/2023 11:46:46 AM, merged with Effectiviness 3/12/2023 1:12:09 PM, merged with Decision_making 3/12/2023 1:12:55 PM, merged with Role_improvement*

25 Quotations:

1:6 ¶ 22, Non-use of the service because of negligence and forgetting can be im… in interview analysis / 1:26 ¶ 35, t can increase utilization better than that we have previously in interview analysis / 1:28 ¶ 36, Even since this digital it can further improve the service and increas… in interview analysis / 1:36 ¶ 40, This mHealth better than our previous our service in interview analysis / 1:43 ¶ 50, For maternal and child health the decision to take service is mothers… in interview analysis / 1:49 ¶ 56, Having information on time can improve the health of mothers and child… in interview analysis / 1:50 ¶ 57, further improve the already improving service in interview analysis / 1:57 ¶ 63, f it continue, it can be effective and be important than previous way… in interview analysis / 1:59 ¶ 64, I think whatever we put in to the community to improve health service… in interview analysis / 1:62 ¶ 66, This can improve and put our usual service one step forward in interview analysis / 1:74 ¶ 75, I don't think people can be affected negatively because they love to l… in interview analysis / 1:77 ¶ 77, Rural mothers are respectful; they do what they told to do, so fear of… in interview analysis / 1:78 ¶ 78, One thing I assure is it will increase up taking maternal and child he… in interview analysis / 1:83 ¶ 83, It can remind which is especially important for family planning. Witho… in interview analysis / 2:4 ¶ 12, If health system organized this way it will be helpful in KII / 2:6 ¶ 16, this is the main thing to improve maternal and child care especially u… in KII / 3:8 ¶ 6, After counseling and aware mother it can be improved in Group discussion / 3:9 ¶ 8, It can improve those listed service (ANC, PNC, Breastfeeding and vacci… in Group discussion / 3:10 ¶ 8, Mother are already exposed to health education, but this one is direct… in Group discussion / 3:18 ¶ 12, It looks like mothers are tired of current service provision so the mH… in Group discussion / 3:19 ¶ 12, So in that case it can completely change the stream and interest. Or t… in Group discussion / 3:20 ¶ 12, They may even consider the increased risk of danger related to pregnan… in Group discussion / 3:43 ¶ 28, At the end this is very important piece of technology that can reduce… in Group discussion / 3:48 ¶ 31, Service at their home and Information and on time vaccination and Heal… in Group discussion / 3:49 ¶ 32, Healthy child feeding and behavioral change for mother that wll be lon… in Group discussion

1:50 ¶ 57 in interview analysis

Text quotation

Created by Girma Gilano on 3/8/2023

further improve the already improving service

2 Codes:

● Benefits of mHealth: Help_professional

Comment: by Girma Gilano

> *3/12/2023 11:46:46 AM, merged with Effectiviness 3/12/2023 1:12:09 PM, merged with Decision_making 3/12/2023 1:12:55 PM, merged with Role_improvement*

30 Quotations:

1:24 ¶ 34, We always give best service to our best capacity, but mHealth may adva… in interview analysis / 1:34 ¶ 39, As HEWs we cannot reach for every mother on time but text can easily r… in interview analysis / 1:36 ¶ 40, This mHealth better than our previous our service in interview analysis / 1:50 ¶ 57, further improve the already improving service in interview analysis / 1:56 ¶ 62, Our current ANC and other formats now request mother to register their… in interview analysis / 1:68 ¶ 70, Every system is getting digitalized so digitalizing health may have mu… in interview analysis / 1:82 ¶ 81, ers usually take what we tell them, it will be successful. I don't thi… in interview analysis / 2:3 ¶ 11, It is helpful to also send information which are not appropriate in pe… in KII / 2:7 ¶ 17, Yeah, it will have effective change on community in KII / 2:9 ¶ 19, Yeah, previously mothers use the information they get during ANC, but… in KII / 2:35 ¶ 54, Since we have many gaps this should be seen as an opportunity in KII / 3:2 ¶ 3, Previous teaching of mother through home to home is going to be throug… in Group discussion / 3:12 ¶ 8, For educated mothers messages will be option less in Group discussion / 3:13 ¶ 8, Starting from me we feed additional food at 2months because the kind o… in Group discussion / 3:18 ¶ 12, It looks like mothers are tired of current service provision so the mH… in Group discussion / 3:25 ¶ 14, There may be emerged women who are successful because of mHealth and t… in Group discussion / 3:29 ¶ 17, As a health professionals applying technology can only easy our burden… in Group discussion / 3:34 ¶ 19, ecause of mHealth we may every address of women and now we can also ca… in Group discussion / 3:35 ¶ 20, We will provide the service for mothers we know now, but previously we… in Group discussion / 3:38 ¶ 22, Of course having organized maternal data can also promote contacting a… in Group discussion / 3:41 ¶ 26, : It is a big opportunity to meet the patient again virtually especial… in Group discussion / 3:42 ¶ 26, Traditionally, when errors happen or if the patients go with the wrong… in Group discussion / 3:43 ¶ 28, At the end this is very important piece of technology that can reduce… in Group discussion / 3:46 ¶ 28, Those infrastructure and awareness creation in staffs, mothers, and co… in Group discussion / 3:52 ¶ 36, People will enjoy talking to others how health institution just concer… in Group discussion / 3:57 ¶ 42, Improve work environment as per the need of the technology and owning… in Group discussion / 3:58 ¶ 43, Aspiring more technologies and helping mothers, taking trainings, givi… in Group discussion / 3:60 ¶ 46, Experience with technology. Enable them to identify areas where mHealt… in Group discussion / 3:61 ¶ 47, Planning, decision making based on mHealth, and increased service take… in Group discussion / 3:62 ¶ 48, Support related to mHealth, fulfillment of deficiencies of human and o… in Group discussion

● Benefits of mHealth: Improve_MCH

Comment: by Girma Gilano

> *3/12/2023 11:46:46 AM, merged with Effectiviness 3/12/2023 1:12:09 PM, merged with Decision_making 3/12/2023 1:12:55 PM, merged with Role_improvement*

25 Quotations:

1:6 ¶ 22, Non-use of the service because of negligence and forgetting can be im… in interview analysis / 1:26 ¶ 35, t can increase utilization better than that we have previously in interview analysis / 1:28 ¶ 36, Even since this digital it can further improve the service and increas… in interview analysis / 1:36 ¶ 40, This mHealth better than our previous our service in interview analysis / 1:43 ¶ 50, For maternal and child health the decision to take service is mothers… in interview analysis / 1:49 ¶ 56, Having information on time can improve the health of mothers and child… in interview analysis / 1:50 ¶ 57, further improve the already improving service in interview analysis / 1:57 ¶ 63, f it continue, it can be effective and be

important than previous way… in interview analysis / 1:59 ¶ 64, I think whatever we put in to the community to improve health service… in interview analysis / 1:62 ¶ 66, This can improve and put our usual service one step forward in interview analysis / 1:74 ¶ 75, I don't think people can be affected negatively because they love to l… in interview analysis / 1:77 ¶ 77, Rural mothers are respectful; they do what they told to do, so fear of… in interview analysis / 1:78 ¶ 78, One thing I assure is it will increase up taking maternal and child he… in interview analysis / 1:83 ¶ 83, It can remind which is especially important for family planning. Witho… in interview analysis / 2:4 ¶ 12, If health system organized this way it will be helpful in KII / 2:6 ¶ 16, this is the main thing to improve maternal and child care especially u… in KII / 3:8 ¶ 6, After counseling and aware mother it can be improved in Group discussion / 3:9 ¶ 8, It can improve those listed service (ANC, PNC, Breastfeeding and vacci… in Group discussion / 3:10 ¶ 8, Mother are already exposed to health education, but this one is direct… in Group discussion / 3:18 ¶ 12, It looks like mothers are tired of current service provision so the mH… in Group discussion / 3:19 ¶ 12, So in that case it can completely change the stream and interest. Or t… in Group discussion / 3:20 ¶ 12, They may even consider the increased risk of danger related to pregnan… in Group discussion / 3:43 ¶ 28, At the end this is very important piece of technology that can reduce… in Group discussion / 3:48 ¶ 31, Service at their home and Information and on time vaccination and Heal… in Group discussion / 3:49 ¶ 32, Healthy child feeding and behavioral change for mother that wll be lon… in Group discussion

1:56 ¶ 62 in interview analysis

Text quotation

Created by Girma Gilano on 3/8/2023

Our current ANC and other formats now request mother to register their favorite phone number.

1 Codes:

- **Benefits of mHealth: Help_professional**

Comment: by Girma Gilano

> *3/12/2023 11:46:46 AM, merged with Effectiviness 3/12/2023 1:12:09 PM, merged with Decision_making 3/12/2023 1:12:55 PM, merged with Role_improvement*

30 Quotations:

1:24 ¶ 34, We always give best service to our best capacity, but mHealth may adva… in interview analysis / 1:34 ¶ 39, As HEWs we cannot reach for every mother on time but text can easily r… in interview analysis / 1:36 ¶ 40, This mHealth better than our previous our service in interview analysis / 1:50 ¶ 57, further improve the already improving service in interview analysis / 1:56 ¶ 62, Our current ANC and other formats now request mother to register their… in interview analysis / 1:68 ¶ 70, Every system is getting digitalized so digitalizing health may have mu… in interview analysis / 1:82 ¶ 81, ers usually take what we tell them, it will be successful. I don't thi… in interview analysis / 2:3 ¶ 11, It is helpful to also send information which are not appropriate in pe… in KII / 2:7 ¶ 17, Yeah, it will have effective change on community in KII / 2:9 ¶ 19, Yeah, previously mothers use the information they get during ANC, but… in KII / 2:35 ¶ 54, Since we have many gaps this should be seen as an opportunity in KII / 3:2 ¶ 3, Previous teaching of mother through home to home is going to be throug… in Group discussion / 3:12 ¶ 8, For educated mothers messages will be option less in Group discussion / 3:13 ¶ 8, Starting from me we feed additional food at 2months because the kind o… in Group discussion / 3:18 ¶ 12, It looks like mothers are tired of current service provision so the mH… in Group discussion / 3:25 ¶ 14, There may be emerged women who are successful because of mHealth and t… in Group discussion / 3:29 ¶ 17, As a health professionals applying technology can only easy our burden… in Group discussion / 3:34 ¶ 19, ecause of mHealth we may every address of women and now we can also ca… in Group discussion / 3:35 ¶ 20, We will provide the service for mothers we know now, but previously we… in Group discussion / 3:38 ¶ 22, Of course having organized maternal data can also promote contacting a… in Group discussion / 3:41 ¶ 26, : It is a big opportunity to meet the patient again virtually especial… in Group discussion / 3:42 ¶ 26, Traditionally, when errors happen or if the patients go with

the wrong… in Group discussion / 3:43 ¶ 28, At the end this is very important piece of technology that can reduce… in Group discussion / 3:46 ¶ 28, Those infrastructure and awareness creation in staffs, mothers, and co… in Group discussion / 3:52 ¶ 36, People will enjoy talking to others how health institution just concer… in Group discussion / 3:57 ¶ 42, Improve work environment as per the need of the technology and owning… in Group discussion / 3:58 ¶ 43, Aspiring more technologies and helping mothers, taking trainings, givi… in Group discussion / 3:60 ¶ 46, Experience with technology. Enable them to identify areas where mHealt… in Group discussion / 3:61 ¶ 47, Planning, decision making based on mHealth, and increased service take… in Group discussion / 3:62 ¶ 48, Support related to mHealth, fulfillment of deficiencies of human and o… in Group discussion

1:68 ¶ 70 in interview analysis

Text quotation

Created by Girma Gilano on 3/8/2023

Every system is getting digitalized so digitalizing health may have much advantage more than just appointme

3 Codes:

- **Benefits of mHealth: ALarming**

Comment: by Girma Gilano

> *3/12/2023 11:46:46 AM, merged with Effectiviness 3/12/2023 1:12:09 PM, merged with Decision_making 3/12/2023 1:12:55 PM, merged with Role_improvement*

6 Quotations:

1:10 ¶ 24, it helps when people are busy with their work to remember appointments in interview analysis / 1:25 ¶ 35, Mothers may not miss their appointment because they forget i in interview analysis / 1:53 ¶ 59, I think mHealth can improve access, appointment on time, availability… in interview analysis / 1:68 ¶ 70, Every system is getting digitalized so digitalizing health may have mu… in interview analysis / 3:27 ¶ 16, It can make the child health growth, for mothers to know danger signs,… in Group discussion / 3:47 ¶ 30, Appointment reminder, service on time, and reduce mortality, but befor… in Group discussion

- **Benefits of mHealth: Help_mothers**

Comment: by Girma Gilano

> *3/12/2023 11:46:46 AM, merged with Effectiviness 3/12/2023 1:12:09 PM, merged with Decision_making 3/12/2023 1:12:55 PM, merged with Role_improvement*

27 Quotations:

1:41 ¶ 47, ion comes after understanding of benefits and harms in interview analysis / 1:45 ¶ 52, Accessibility can be improved mothers get information at their homes in interview analysis / 1:51 ¶ 58, She can access any information in interview analysis / 1:53 ¶ 59, I think mHealth can improve access, appointment on time, availability… in interview analysis / 1:65 ¶ 67, Her husband or children will also push her to go even if the condition… in interview analysis / 1:68 ¶ 70, Every system is getting digitalized so digitalizing health may have mu… in interview analysis / 1:77 ¶ 77, Rural mothers are respectful; they do what they told to do, so fear of… in interview analysis / 1:81 ¶ 80, pe mothers will turn to use mobiles, but only after getting its import… in interview analysis / 2:3 ¶ 11, It is helpful to also send information which are not appropriate in pe… in KII / 2:7 ¶ 17, Yeah, it will have effective change on community in KII / 2:9 ¶ 19, Yeah, previously mothers use the information they get during ANC, but… in KII / 2:11 ¶ 21, Information access, no transportation cost, and no professional resour… in KII / 2:36 ¶ 55, No need of motor or vehicles because it is mHealth. I will be happy to… in KII / 3:6 ¶ 5, They difference for mHealth is mother always get counseling or health… in Group discussion / 3:18 ¶ 12, It looks like mothers are

tired of current service provision so the mH… in Group discussion / 3:23 ¶ 14, Previously, people think poor health system and there are many complia… in Group discussion / 3:24 ¶ 14, Over time this can change community perception and they may reebok mot… in Group discussion / 3:25 ¶ 14, There may be emerged women who are successful because of mHealth and t… in Group discussion / 3:27 ¶ 16, It can make the child health growth, for mothers to know danger signs,… in Group discussion / 3:38 ¶ 22, Of course having organized maternal data can also promote contacting a… in Group discussion / 3:41 ¶ 26, : It is a big opportunity to meet the patient again virtually especial… in Group discussion / 3:42 ¶ 26, Traditionally, when errors happen or if the patients go with the wrong… in Group discussion / 3:50 ¶ 33, Benefit for mothers for next pregnancies and healthy prospect for chil… in Group discussion / 3:59 ¶ 45, Satisfaction of their client service and familiarization with technolo… in Group discussion / 3:61 ¶ 47, Planning, decision making based on mHealth, and increased service take… in Group discussion / 3:62 ¶ 48, Support related to mHealth, fulfillment of deficiencies of human and o… in Group discussion / 3:67 ¶ 53, he respect for pregnancy mothers may think gave many births and not ca… in Group discussion

Benefits of mHealth: Help_professional

Comment: by Girma Gilano

> *3/12/2023 11:46:46 AM, merged with Effectiviness 3/12/2023 1:12:09 PM, merged with Decision_making 3/12/2023 1:12:55 PM, merged with Role_improvement*

30 Quotations:

1:24 ¶ 34, We always give best service to our best capacity, but mHealth may adva… in interview analysis / 1:34 ¶ 39, As HEWs we cannot reach for every mother on time but text can easily r… in interview analysis / 1:36 ¶ 40, This mHealth better than our previous our service in interview analysis / 1:50 ¶ 57, further improve the already improving service in interview analysis / 1:56 ¶ 62, Our current ANC and other formats now request mother to register their… in interview analysis / 1:68 ¶ 70, Every system is getting digitalized so digitalizing health may have mu… in interview analysis / 1:82 ¶ 81, ers usually take what we tell them, it will be successful. I don’t thi… in interview analysis / 2:3 ¶ 11, It is helpful to also send information which are not appropriate in pe… in KII / 2:7 ¶ 17, Yeah, it will have effective change on community in KII / 2:9 ¶ 19, Yeah, previously mothers use the information they get during ANC, but… in KII / 2:35 ¶ 54, Since we have many gaps this should be seen as an opportunity in KII / 3:2 ¶ 3, Previous teaching of mother through home to home is going to be throug… in Group discussion / 3:12 ¶ 8, For educated mothers messages will be option less in Group discussion / 3:13 ¶ 8, Starting from me we feed additional food at 2months because the kind o… in Group discussion / 3:18 ¶ 12, It looks like mothers are tired of current service provision so the mH… in Group discussion / 3:25 ¶ 14, There may be emerged women who are successful because of mHealth and t… in Group discussion / 3:29 ¶ 17, As a health professionals applying technology can only easy our burden… in Group discussion / 3:34 ¶ 19, ecause of mHealth we may every address of women and now we can also ca… in Group discussion / 3:35 ¶ 20, We will provide the service for mothers we know now, but previously we… in Group discussion / 3:38 ¶ 22, Of course having organized maternal data can also promote contacting a… in Group discussion / 3:41 ¶ 26, : It is a big opportunity to meet the patient again virtually especial… in Group discussion / 3:42 ¶ 26, Traditionally, when errors happen or if the patients go with the wrong… in Group discussion / 3:43 ¶ 28, At the end this is very important piece of technology that can reduce… in Group discussion / 3:46 ¶ 28, Those infrastructure and awareness creation in staffs, mothers, and co… in Group discussion / 3:52 ¶ 36, People will enjoy talking to others how health institution just concer… in Group discussion / 3:57 ¶ 42, Improve work environment as per the need of the technology and owning… in Group discussion / 3:58 ¶ 43, Aspiring more technologies and helping mothers, taking trainings, givi… in Group discussion / 3:60 ¶ 46, Experience with technology. Enable them to identify areas where mHealt… in Group discussion / 3:61 ¶ 47, Planning, decision making based on mHealth, and increased service take… in Group discussion / 3:62 ¶ 48, Support related to mHealth, fulfillment of deficiencies of human and o… in Group discussion

1:82 ¶ 81 in interview analysis

Text quotation

Created by Girma Gilano on 3/8/2023

ers usually take what we tell them, it will be successful. I don't think reading is a problem because families have all learned people inside, the problem is which phone is accessible or near to mother

1 Codes:

- **Benefits of mHealth: Help_professional**

  Comment: by Girma Gilano

  > *3/12/2023 11:46:46 AM, merged with Effectiviness 3/12/2023 1:12:09 PM, merged with Decision_making 3/12/2023 1:12:55 PM, merged with Role_improvement*

  30 Quotations:

  1:24 ¶ 34, We always give best service to our best capacity, but mHealth may adva… in interview analysis / 1:34 ¶ 39, As HEWs we cannot reach for every mother on time but text can easily r… in interview analysis / 1:36 ¶ 40, This mHealth better than our previous our service in interview analysis / 1:50 ¶ 57, further improve the already improving service in interview analysis / 1:56 ¶ 62, Our current ANC and other formats now request mother to register their… in interview analysis / 1:68 ¶ 70, Every system is getting digitalized so digitalizing health may have mu… in interview analysis / 1:82 ¶ 81, ers usually take what we tell them, it will be successful. I don't thi… in interview analysis / 2:3 ¶ 11, It is helpful to also send information which are not appropriate in pe… in KII / 2:7 ¶ 17, Yeah, it will have effective change on community in KII / 2:9 ¶ 19, Yeah, previously mothers use the information they get during ANC, but… in KII / 2:35 ¶ 54, Since we have many gaps this should be seen as an opportunity in KII / 3:2 ¶ 3, Previous teaching of mother through home to home is going to be throug… in Group discussion / 3:12 ¶ 8, For educated mothers messages will be option less in Group discussion / 3:13 ¶ 8, Starting from me we feed additional food at 2months because the kind o… in Group discussion / 3:18 ¶ 12, It looks like mothers are tired of current service provision so the mH… in Group discussion / 3:25 ¶ 14, There may be emerged women who are successful because of mHealth and t… in Group discussion / 3:29 ¶ 17, As a health professionals applying technology can only easy our burden… in Group discussion / 3:34 ¶ 19, ecause of mHealth we may every address of women and now we can also ca… in Group discussion / 3:35 ¶ 20, We will provide the service for mothers we know now, but previously we… in Group discussion / 3:38 ¶ 22, Of course having organized maternal data can also promote contacting a… in Group discussion / 3:41 ¶ 26, : It is a big opportunity to meet the patient again virtually especial… in Group discussion / 3:42 ¶ 26, Traditionally, when errors happen or if the patients go with the wrong… in Group discussion / 3:43 ¶ 28, At the end this is very important piece of technology that can reduce… in Group discussion / 3:46 ¶ 28, Those infrastructure and awareness creation in staffs, mothers, and co… in Group discussion / 3:52 ¶ 36, People will enjoy talking to others how health institution just concer… in Group discussion / 3:57 ¶ 42, Improve work environment as per the need of the technology and owning… in Group discussion / 3:58 ¶ 43, Aspiring more technologies and helping mothers, taking trainings, givi… in Group discussion / 3:60 ¶ 46, Experience with technology. Enable them to identify areas where mHealt… in Group discussion / 3:61 ¶ 47, Planning, decision making based on mHealth, and increased service take… in Group discussion / 3:62 ¶ 48, Support related to mHealth, fulfillment of deficiencies of human and o… in Group discussion

2:3 ¶ 11 in KII

Text quotation

Created by Girma Gilano on 3/9/2023

It is helpful to also send information which are not appropriate in person

2 Codes:

- **Benefits of mHealth: Help_mothers**

Comment: by Girma Gilano

> *3/12/2023 11:46:46 AM, merged with Effectiviness 3/12/2023 1:12:09 PM, merged with Decision_making 3/12/2023 1:12:55 PM, merged with Role_improvement*

27 Quotations:

1:41 ¶ 47, ion comes after understanding of benefits and harms in interview analysis / 1:45 ¶ 52, Accessibility can be improved mothers get information at their homes in interview analysis / 1:51 ¶ 58, She can access any information in interview analysis / 1:53 ¶ 59, I think mHealth can improve access, appointment on time, availability… in interview analysis / 1:65 ¶ 67, Her husband or children will also push her to go even if the condition… in interview analysis / 1:68 ¶ 70, Every system is getting digitalized so digitalizing health may have mu… in interview analysis / 1:77 ¶ 77, Rural mothers are respectful; they do what they told to do, so fear of… in interview analysis / 1:81 ¶ 80, pe mothers will turn to use mobiles, but only after getting its import… in interview analysis / 2:3 ¶ 11, It is helpful to also send information which are not appropriate in pe… in KII / 2:7 ¶ 17, Yeah, it will have effective change on community in KII / 2:9 ¶ 19, Yeah, previously mothers use the information they get during ANC, but… in KII / 2:11 ¶ 21, Information access, no transportation cost, and no professional resour… in KII / 2:36 ¶ 55, No need of motor or vehicles because it is mHealth. I will be happy to… in KII / 3:6 ¶ 5, They difference for mHealth is mother always get counseling or health… in Group discussion / 3:18 ¶ 12, It looks like mothers are tired of current service provision so the mH… in Group discussion / 3:23 ¶ 14, Previously, people think poor health system and there are many complia… in Group discussion / 3:24 ¶ 14, Over time this can change community perception and they may reebok mot… in Group discussion / 3:25 ¶ 14, There may be emerged women who are successful because of mHealth and t… in Group discussion / 3:27 ¶ 16, It can make the child health growth, for mothers to know danger signs,… in Group discussion / 3:38 ¶ 22, Of course having organized maternal data can also promote contacting a… in Group discussion / 3:41 ¶ 26, : It is a big opportunity to meet the patient again virtually especial… in Group discussion / 3:42 ¶ 26, Traditionally, when errors happen or if the patients go with the wrong… in Group discussion / 3:50 ¶ 33, Benefit for mothers for next pregnancies and healthy prospect for chil… in Group discussion / 3:59 ¶ 45, Satisfaction of their client service and familiarization with technolo… in Group discussion / 3:61 ¶ 47, Planning, decision making based on mHealth, and increased service take… in Group discussion / 3:62 ¶ 48, Support related to mHealth, fulfillment of deficiencies of human and o… in Group discussion / 3:67 ¶ 53, he respect for pregnancy mothers may think gave many births and not ca… in Group discussion

● Benefits of mHealth: Help_professional

Comment: by Girma Gilano

> *3/12/2023 11:46:46 AM, merged with Effectiviness 3/12/2023 1:12:09 PM, merged with Decision_making 3/12/2023 1:12:55 PM, merged with Role_improvement*

30 Quotations:

1:24 ¶ 34, We always give best service to our best capacity, but mHealth may adva… in interview analysis / 1:34 ¶ 39, As HEWs we cannot reach for every mother on time but text can easily r… in interview analysis / 1:36 ¶ 40, This mHealth better than our previous our service in interview analysis / 1:50 ¶ 57, further improve the already improving service in interview analysis / 1:56 ¶ 62, Our current ANC and other formats now request mother to register their… in interview analysis / 1:68 ¶ 70, Every system is getting digitalized so digitalizing health may have mu… in interview analysis / 1:82 ¶ 81, ers usually take what we tell them, it will be successful. I don’t thi… in interview analysis / 2:3 ¶ 11, It is helpful to also send information which are not appropriate in pe… in KII / 2:7 ¶ 17, Yeah, it will have effective change on community in KII / 2:9 ¶ 19, Yeah, previously mothers use the information they get during ANC, but… in KII / 2:35 ¶ 54, Since we have many gaps this should be seen as an opportunity in KII / 3:2 ¶ 3, Previous teaching of mother through home to home is going to be throug… in Group discussion / 3:12 ¶ 8, For educated mothers messages will be option less in Group discussion / 3:13 ¶ 8, Starting from me we feed additional food at 2months because the kind o… in Group discussion / 3:18 ¶ 12, It looks like mothers are tired of current service provision so the mH… in Group discussion / 3:25 ¶ 14, There may be emerged women who are successful because of mHealth and t… in Group discussion / 3:29 ¶ 17, As a health professionals applying technology can only easy our burden… in Group discussion / 3:34 ¶ 19, ecause of mHealth we may every address of women and now we can also ca… in Group discussion / 3:35 ¶ 20, We will provide the service for mothers we know now, but previously we… in Group discussion / 3:38 ¶ 22, Of course having organized maternal data can also promote

contacting a… in Group discussion / 3:41 ¶ 26, : It is a big opportunity to meet the patient again virtually especial… in Group discussion / 3:42 ¶ 26, Traditionally, when errors happen or if the patients go with the wrong… in Group discussion / 3:43 ¶ 28, At the end this is very important piece of technology that can reduce… in Group discussion / 3:46 ¶ 28, Those infrastructure and awareness creation in staffs, mothers, and co… in Group discussion / 3:52 ¶ 36, People will enjoy talking to others how health institution just concer… in Group discussion / 3:57 ¶ 42, Improve work environment as per the need of the technology and owning… in Group discussion / 3:58 ¶ 43, Aspiring more technologies and helping mothers, taking trainings, givi… in Group discussion / 3:60 ¶ 46, Experience with technology. Enable them to identify areas where mHealt… in Group discussion / 3:61 ¶ 47, Planning, decision making based on mHealth, and increased service take… in Group discussion / 3:62 ¶ 48, Support related to mHealth, fulfillment of deficiencies of human and o… in Group discussion

2:7 ¶ 17 in KII

Text quotation

Created by Girma Gilano on 3/9/2023

Yeah, it will have effective change on community

3 Codes:

Benefits of mHealth: Effective

Comment: by Girma Gilano

> *3/12/2023 11:46:46 AM, merged with Effectiviness 3/12/2023 1:12:09 PM, merged with Decision_making 3/12/2023 1:12:55 PM, merged with Role_improvement*

6 Quotations:

1:48 ¶ 55, t can reduce cost, time, and other resources in interview analysis / 1:60 ¶ 64, It will be very effective because it advances the services in interview analysis / 1:61 ¶ 65, I think it will be very effective because we can see the newly impleme… in interview analysis / 1:81 ¶ 80, pe mothers will turn to use mobiles, but only after getting its import… in interview analysis / 1:98 ¶ 98, If all input resources are fulfilled, there will be no problem. It is… in interview analysis / 2:7 ¶ 17, Yeah, it will have effective change on community in KII

Benefits of mHealth: Help_mothers

Comment: by Girma Gilano

> *3/12/2023 11:46:46 AM, merged with Effectiviness 3/12/2023 1:12:09 PM, merged with Decision_making 3/12/2023 1:12:55 PM, merged with Role_improvement*

27 Quotations:

1:41 ¶ 47, ion comes after understanding of benefits and harms in interview analysis / 1:45 ¶ 52, Accessibility can be improved mothers get information at their homes in interview analysis / 1:51 ¶ 58, She can access any information in interview analysis / 1:53 ¶ 59, I think mHealth can improve access, appointment on time, availability… in interview analysis / 1:65 ¶ 67, Her husband or children will also push her to go even if the condition… in interview analysis / 1:68 ¶ 70, Every system is getting digitalized so digitalizing health may have mu… in interview analysis / 1:77 ¶ 77, Rural mothers are respectful; they do what they told to do, so fear of… in interview analysis / 1:81 ¶ 80, pe mothers will turn to use mobiles, but only after getting its import… in interview analysis / 2:3 ¶ 11, It is helpful to also send information which are not appropriate in pe… in KII / 2:7 ¶ 17, Yeah, it will have effective change on community in KII / 2:9 ¶ 19, Yeah, previously mothers use the information they get during ANC, but… in KII / 2:11 ¶ 21, Information access, no transportation cost, and no professional resour… in KII / 2:36 ¶ 55, No need of motor or vehicles because it is mHealth. I will be happy to… in KII / 3:6 ¶ 5, They difference for mHealth

is mother always get counseling or health… in Group discussion / 3:18 ¶ 12, It looks like mothers are tired of current service provision so the mH… in Group discussion / 3:23 ¶ 14, Previously, people think poor health system and there are many complia… in Group discussion / 3:24 ¶ 14, Over time this can change community perception and they may reebok mot… in Group discussion / 3:25 ¶ 14, There may be emerged women who are successful because of mHealth and t… in Group discussion / 3:27 ¶ 16, It can make the child health growth, for mothers to know danger signs,… in Group discussion / 3:38 ¶ 22, Of course having organized maternal data can also promote contacting a… in Group discussion / 3:41 ¶ 26, : It is a big opportunity to meet the patient again virtually especial… in Group discussion / 3:42 ¶ 26, Traditionally, when errors happen or if the patients go with the wrong… in Group discussion / 3:50 ¶ 33, Benefit for mothers for next pregnancies and healthy prospect for chil… in Group discussion / 3:59 ¶ 45, Satisfaction of their client service and familiarization with technolo… in Group discussion / 3:61 ¶ 47, Planning, decision making based on mHealth, and increased service take… in Group discussion / 3:62 ¶ 48, Support related to mHealth, fulfillment of deficiencies of human and o… in Group discussion / 3:67 ¶ 53, he respect for pregnancy mothers may think gave many births and not ca… in Group discussion

Benefits of mHealth: Help_professional

Comment: by Girma Gilano

> *3/12/2023 11:46:46 AM, merged with Effectiviness 3/12/2023 1:12:09 PM, merged with Decision_making 3/12/2023 1:12:55 PM, merged with Role_improvement*

30 Quotations:

1:24 ¶ 34, We always give best service to our best capacity, but mHealth may adva… in interview analysis / 1:34 ¶ 39, As HEWs we cannot reach for every mother on time but text can easily r… in interview analysis / 1:36 ¶ 40, This mHealth better than our previous our service in interview analysis / 1:50 ¶ 57, further improve the already improving service in interview analysis / 1:56 ¶ 62, Our current ANC and other formats now request mother to register their… in interview analysis / 1:68 ¶ 70, Every system is getting digitalized so digitalizing health may have mu… in interview analysis / 1:82 ¶ 81, ers usually take what we tell them, it will be successful. I don't thi… in interview analysis / 2:3 ¶ 11, It is helpful to also send information which are not appropriate in pe… in KII / 2:7 ¶ 17, Yeah, it will have effective change on community in KII / 2:9 ¶ 19, Yeah, previously mothers use the information they get during ANC, but… in KII / 2:35 ¶ 54, Since we have many gaps this should be seen as an opportunity in KII / 3:2 ¶ 3, Previous teaching of mother through home to home is going to be throug… in Group discussion / 3:12 ¶ 8, For educated mothers messages will be option less in Group discussion / 3:13 ¶ 8, Starting from me we feed additional food at 2months because the kind o… in Group discussion / 3:18 ¶ 12, It looks like mothers are tired of current service provision so the mH… in Group discussion / 3:25 ¶ 14, There may be emerged women who are successful because of mHealth and t… in Group discussion / 3:29 ¶ 17, As a health professionals applying technology can only easy our burden… in Group discussion / 3:34 ¶ 19, ecause of mHealth we may every address of women and now we can also ca… in Group discussion / 3:35 ¶ 20, We will provide the service for mothers we know now, but previously we… in Group discussion / 3:38 ¶ 22, Of course having organized maternal data can also promote contacting a… in Group discussion / 3:41 ¶ 26, : It is a big opportunity to meet the patient again virtually especial… in Group discussion / 3:42 ¶ 26, Traditionally, when errors happen or if the patients go with the wrong… in Group discussion / 3:43 ¶ 28, At the end this is very important piece of technology that can reduce… in Group discussion / 3:46 ¶ 28, Those infrastructure and awareness creation in staffs, mothers, and co… in Group discussion / 3:52 ¶ 36, People will enjoy talking to others how health institution just concer… in Group discussion / 3:57 ¶ 42, Improve work environment as per the need of the technology and owning… in Group discussion / 3:58 ¶ 43, Aspiring more technologies and helping mothers, taking trainings, givi… in Group discussion / 3:60 ¶ 46, Experience with technology. Enable them to identify areas where mHealt… in Group discussion / 3:61 ¶ 47, Planning, decision making based on mHealth, and increased service take… in Group discussion / 3:62 ¶ 48, Support related to mHealth, fulfillment of deficiencies of human and o… in Group discussion

2:9 ¶ 19 in KII

Text quotation

Created by Girma Gilano on 3/9/2023

Yeah, previously mothers use the information they get during ANC, but mothers may forget as time progress so mHealth helpful backup

2 Codes:

● Benefits of mHealth: Help_mothers

Comment: by Girma Gilano

> *3/12/2023 11:46:46 AM, merged with Effectiviness 3/12/2023 1:12:09 PM, merged with Decision_making 3/12/2023 1:12:55 PM, merged with Role_improvement*

27 Quotations:

1:41 ¶ 47, ion comes after understanding of benefits and harms in interview analysis / 1:45 ¶ 52, Accessibility can be improved mothers get information at their homes in interview analysis / 1:51 ¶ 58, She can access any information in interview analysis / 1:53 ¶ 59, I think mHealth can improve access, appointment on time, availability… in interview analysis / 1:65 ¶ 67, Her husband or children will also push her to go even if the condition… in interview analysis / 1:68 ¶ 70, Every system is getting digitalized so digitalizing health may have mu… in interview analysis / 1:77 ¶ 77, Rural mothers are respectful; they do what they told to do, so fear of… in interview analysis / 1:81 ¶ 80, pe mothers will turn to use mobiles, but only after getting its import… in interview analysis / 2:3 ¶ 11, It is helpful to also send information which are not appropriate in pe… in KII / 2:7 ¶ 17, Yeah, it will have effective change on community in KII / 2:9 ¶ 19, Yeah, previously mothers use the information they get during ANC, but… in KII / 2:11 ¶ 21, Information access, no transportation cost, and no professional resour… in KII / 2:36 ¶ 55, No need of motor or vehicles because it is mHealth. I will be happy to… in KII / 3:6 ¶ 5, They difference for mHealth is mother always get counseling or health… in Group discussion / 3:18 ¶ 12, It looks like mothers are tired of current service provision so the mH… in Group discussion / 3:23 ¶ 14, Previously, people think poor health system and there are many complia… in Group discussion / 3:24 ¶ 14, Over time this can change community perception and they may reebok mot… in Group discussion / 3:25 ¶ 14, There may be emerged women who are successful because of mHealth and t… in Group discussion / 3:27 ¶ 16, It can make the child health growth, for mothers to know danger signs,… in Group discussion / 3:38 ¶ 22, Of course having organized maternal data can also promote contacting a… in Group discussion / 3:41 ¶ 26, : It is a big opportunity to meet the patient again virtually especial… in Group discussion / 3:42 ¶ 26, Traditionally, when errors happen or if the patients go with the wrong… in Group discussion / 3:50 ¶ 33, Benefit for mothers for next pregnancies and healthy prospect for chil… in Group discussion / 3:59 ¶ 45, Satisfaction of their client service and familiarization with technolo… in Group discussion / 3:61 ¶ 47, Planning, decision making based on mHealth, and increased service take… in Group discussion / 3:62 ¶ 48, Support related to mHealth, fulfillment of deficiencies of human and o… in Group discussion / 3:67 ¶ 53, he respect for pregnancy mothers may think gave many births and not ca… in Group discussion

● Benefits of mHealth: Help_professional

Comment: by Girma Gilano

> *3/12/2023 11:46:46 AM, merged with Effectiviness 3/12/2023 1:12:09 PM, merged with Decision_making 3/12/2023 1:12:55 PM, merged with Role_improvement*

30 Quotations:

1:24 ¶ 34, We always give best service to our best capacity, but mHealth may adva… in interview analysis / 1:34 ¶ 39, As HEWs we cannot reach for every mother on time but text can easily r… in interview analysis / 1:36 ¶ 40, This mHealth better than our previous our service in interview analysis / 1:50 ¶ 57, further improve the already improving service in interview analysis / 1:56 ¶ 62, Our current ANC and other formats now request mother to register their… in interview analysis / 1:68 ¶ 70, Every system is getting digitalized so digitalizing health may have mu… in interview analysis / 1:82 ¶ 81, ers usually take what we tell them, it will be successful. I don't thi… in interview analysis / 2:3 ¶ 11, It is helpful to also send information which are not appropriate in pe… in KII / 2:7 ¶ 17, Yeah, it will have effective change on community in KII / 2:9 ¶ 19, Yeah, previously mothers use the information they get during ANC, but… in KII / 2:35 ¶ 54, Since we have many gaps this should be seen as an opportunity in

KII / 3:2 ¶ 3, Previous teaching of mother through home to home is going to be throug… in Group discussion / 3:12 ¶ 8, For educated mothers messages will be option less in Group discussion / 3:13 ¶ 8, Starting from me we feed additional food at 2months because the kind o… in Group discussion / 3:18 ¶ 12, It looks like mothers are tired of current service provision so the mH… in Group discussion / 3:25 ¶ 14, There may be emerged women who are successful because of mHealth and t… in Group discussion / 3:29 ¶ 17, As a health professionals applying technology can only easy our burden… in Group discussion / 3:34 ¶ 19, ecause of mHealth we may every address of women and now we can also ca… in Group discussion / 3:35 ¶ 20, We will provide the service for mothers we know now, but previously we… in Group discussion / 3:38 ¶ 22, Of course having organized maternal data can also promote contacting a… in Group discussion / 3:41 ¶ 26, : It is a big opportunity to meet the patient again virtually especial… in Group discussion / 3:42 ¶ 26, Traditionally, when errors happen or if the patients go with the wrong… in Group discussion / 3:43 ¶ 28, At the end this is very important piece of technology that can reduce… in Group discussion / 3:46 ¶ 28, Those infrastructure and awareness creation in staffs, mothers, and co… in Group discussion / 3:52 ¶ 36, People will enjoy talking to others how health institution just concer… in Group discussion / 3:57 ¶ 42, Improve work environment as per the need of the technology and owning… in Group discussion / 3:58 ¶ 43, Aspiring more technologies and helping mothers, taking trainings, givi… in Group discussion / 3:60 ¶ 46, Experience with technology. Enable them to identify areas where mHealt… in Group discussion / 3:61 ¶ 47, Planning, decision making based on mHealth, and increased service take… in Group discussion / 3:62 ¶ 48, Support related to mHealth, fulfillment of deficiencies of human and o… in Group discussion

2:35 ¶ 54 in KII

Text quotation

Created by Girma Gilano on 3/9/2023

Since we have many gaps this should be seen as an opportunity

1 Codes:

- **Benefits of mHealth: Help_professional**

Comment: by Girma Gilano

> *3/12/2023 11:46:46 AM, merged with Effectiviness 3/12/2023 1:12:09 PM, merged with Decision_making 3/12/2023 1:12:55 PM, merged with Role_improvement*

30 Quotations:

1:24 ¶ 34, We always give best service to our best capacity, but mHealth may adva… in interview analysis / 1:34 ¶ 39, As HEWs we cannot reach for every mother on time but text can easily r… in interview analysis / 1:36 ¶ 40, This mHealth better than our previous our service in interview analysis / 1:50 ¶ 57, further improve the already improving service in interview analysis / 1:56 ¶ 62, Our current ANC and other formats now request mother to register their… in interview analysis / 1:68 ¶ 70, Every system is getting digitalized so digitalizing health may have mu… in interview analysis / 1:82 ¶ 81, ers usually take what we tell them, it will be successful. I don't thi… in interview analysis / 2:3 ¶ 11, It is helpful to also send information which are not appropriate in pe… in KII / 2:7 ¶ 17, Yeah, it will have effective change on community in KII / 2:9 ¶ 19, Yeah, previously mothers use the information they get during ANC, but… in KII / 2:35 ¶ 54, Since we have many gaps this should be seen as an opportunity in KII / 3:2 ¶ 3, Previous teaching of mother through home to home is going to be throug… in Group discussion / 3:12 ¶ 8, For educated mothers messages will be option less in Group discussion / 3:13 ¶ 8, Starting from me we feed additional food at 2months because the kind o… in Group discussion / 3:18 ¶ 12, It looks like mothers are tired of current service provision so the mH… in Group discussion / 3:25 ¶ 14, There may be emerged women who are successful because of mHealth and t… in Group discussion / 3:29 ¶ 17, As a health professionals applying technology can only easy our burden… in Group discussion / 3:34 ¶ 19, ecause of mHealth we may every address of women and now we can also ca… in Group discussion / 3:35 ¶ 20, We will provide the service for mothers we know now, but previously we… in Group discussion / 3:38 ¶ 22, Of course having organized maternal data can also promote

contacting a… in Group discussion / 3:41 ¶ 26, : It is a big opportunity to meet the patient again virtually especial… in Group discussion / 3:42 ¶ 26, Traditionally, when errors happen or if the patients go with the wrong… in Group discussion / 3:43 ¶ 28, At the end this is very important piece of technology that can reduce… in Group discussion / 3:46 ¶ 28, Those infrastructure and awareness creation in staffs, mothers, and co… in Group discussion / 3:52 ¶ 36, People will enjoy talking to others how health institution just concer… in Group discussion / 3:57 ¶ 42, Improve work environment as per the need of the technology and owning… in Group discussion / 3:58 ¶ 43, Aspiring more technologies and helping mothers, taking trainings, givi… in Group discussion / 3:60 ¶ 46, Experience with technology. Enable them to identify areas where mHealt… in Group discussion / 3:61 ¶ 47, Planning, decision making based on mHealth, and increased service take… in Group discussion / 3:62 ¶ 48, Support related to mHealth, fulfillment of deficiencies of human and o… in Group discussion

3:2 ¶ 3 in Group discussion

Text quotation

Created by Girma Gilano on 3/9/2023

Previous teaching of mother through home to home is going to be through mHealth that very affordable and easily accessible.

1 Codes:

● Benefits of mHealth: Help_professional

Comment: by Girma Gilano

> *3/12/2023 11:46:46 AM, merged with Effectiviness 3/12/2023 1:12:09 PM, merged with Decision_making 3/12/2023 1:12:55 PM, merged with Role_improvement*

30 Quotations:

1:24 ¶ 34, We always give best service to our best capacity, but mHealth may adva… in interview analysis / 1:34 ¶ 39, As HEWs we cannot reach for every mother on time but text can easily r… in interview analysis / 1:36 ¶ 40, This mHealth better than our previous our service in interview analysis / 1:50 ¶ 57, further improve the already improving service in interview analysis / 1:56 ¶ 62, Our current ANC and other formats now request mother to register their… in interview analysis / 1:68 ¶ 70, Every system is getting digitalized so digitalizing health may have mu… in interview analysis / 1:82 ¶ 81, ers usually take what we tell them, it will be successful. I don't thi… in interview analysis / 2:3 ¶ 11, It is helpful to also send information which are not appropriate in pe… in KII / 2:7 ¶ 17, Yeah, it will have effective change on community in KII / 2:9 ¶ 19, Yeah, previously mothers use the information they get during ANC, but… in KII / 2:35 ¶ 54, Since we have many gaps this should be seen as an opportunity in KII / 3:2 ¶ 3, Previous teaching of mother through home to home is going to be throug… in Group discussion / 3:12 ¶ 8, For educated mothers messages will be option less in Group discussion / 3:13 ¶ 8, Starting from me we feed additional food at 2months because the kind o… in Group discussion / 3:18 ¶ 12, It looks like mothers are tired of current service provision so the mH… in Group discussion / 3:25 ¶ 14, There may be emerged women who are successful because of mHealth and t… in Group discussion / 3:29 ¶ 17, As a health professionals applying technology can only easy our burden… in Group discussion / 3:34 ¶ 19, ecause of mHealth we may every address of women and now we can also ca… in Group discussion / 3:35 ¶ 20, We will provide the service for mothers we know now, but previously we… in Group discussion / 3:38 ¶ 22, Of course having organized maternal data can also promote contacting a… in Group discussion / 3:41 ¶ 26, : It is a big opportunity to meet the patient again virtually especial… in Group discussion / 3:42 ¶ 26, Traditionally, when errors happen or if the patients go with the wrong… in Group discussion / 3:43 ¶ 28, At the end this is very important piece of technology that can reduce… in Group discussion / 3:46 ¶ 28, Those infrastructure and awareness creation in staffs, mothers, and co… in Group discussion / 3:52 ¶ 36, People will enjoy talking to others how health institution just concer… in Group discussion / 3:57 ¶ 42, Improve work environment as per the need of the technology and owning… in Group discussion / 3:58 ¶ 43, Aspiring more technologies and helping mothers, taking trainings, givi… in Group discussion / 3:60 ¶ 46, Experience with technology. Enable

them to identify areas where mHealt… in Group discussion / 3:61 ¶ 47, Planning, decision making based on mHealth, and increased service take… in Group discussion / 3:62 ¶ 48, Support related to mHealth, fulfillment of deficiencies of human and o… in Group discussion

3:12 ¶ 8 in Group discussion

Text quotation

Created by Girma Gilano on 3/9/2023

For educated mothers messages will be option less

1 Codes:

- **Benefits of mHealth: Help_professional**

Comment: by Girma Gilano

> *3/12/2023 11:46:46 AM, merged with Effectiviness 3/12/2023 1:12:09 PM, merged with Decision_making 3/12/2023 1:12:55 PM, merged with Role_improvement*

30 Quotations:

1:24 ¶ 34, We always give best service to our best capacity, but mHealth may adva… in interview analysis / 1:34 ¶ 39, As HEWs we cannot reach for every mother on time but text can easily r… in interview analysis / 1:36 ¶ 40, This mHealth better than our previous our service in interview analysis / 1:50 ¶ 57, further improve the already improving service in interview analysis / 1:56 ¶ 62, Our current ANC and other formats now request mother to register their… in interview analysis / 1:68 ¶ 70, Every system is getting digitalized so digitalizing health may have mu… in interview analysis / 1:82 ¶ 81, ers usually take what we tell them, it will be successful. I don't thi… in interview analysis / 2:3 ¶ 11, It is helpful to also send information which are not appropriate in pe… in KII / 2:7 ¶ 17, Yeah, it will have effective change on community in KII / 2:9 ¶ 19, Yeah, previously mothers use the information they get during ANC, but… in KII / 2:35 ¶ 54, Since we have many gaps this should be seen as an opportunity in KII / 3:2 ¶ 3, Previous teaching of mother through home to home is going to be throug… in Group discussion / 3:12 ¶ 8, For educated mothers messages will be option less in Group discussion / 3:13 ¶ 8, Starting from me we feed additional food at 2months because the kind o… in Group discussion / 3:18 ¶ 12, It looks like mothers are tired of current service provision so the mH… in Group discussion / 3:25 ¶ 14, There may be emerged women who are successful because of mHealth and t… in Group discussion / 3:29 ¶ 17, As a health professionals applying technology can only easy our burden… in Group discussion / 3:34 ¶ 19, ecause of mHealth we may every address of women and now we can also ca… in Group discussion / 3:35 ¶ 20, We will provide the service for mothers we know now, but previously we… in Group discussion / 3:38 ¶ 22, Of course having organized maternal data can also promote contacting a… in Group discussion / 3:41 ¶ 26, : It is a big opportunity to meet the patient again virtually especial… in Group discussion / 3:42 ¶ 26, Traditionally, when errors happen or if the patients go with the wrong… in Group discussion / 3:43 ¶ 28, At the end this is very important piece of technology that can reduce… in Group discussion / 3:46 ¶ 28, Those infrastructure and awareness creation in staffs, mothers, and co… in Group discussion / 3:52 ¶ 36, People will enjoy talking to others how health institution just concer… in Group discussion / 3:57 ¶ 42, Improve work environment as per the need of the technology and owning… in Group discussion / 3:58 ¶ 43, Aspiring more technologies and helping mothers, taking trainings, givi… in Group discussion / 3:60 ¶ 46, Experience with technology. Enable them to identify areas where mHealt… in Group discussion / 3:61 ¶ 47, Planning, decision making based on mHealth, and increased service take… in Group discussion / 3:62 ¶ 48, Support related to mHealth, fulfillment of deficiencies of human and o… in Group discussion

3:13 ¶ 8 in Group discussion

Text quotation

Created by Girma Gilano on 3/9/2023

Starting from me we feed additional food at 2months because the kind of job we engaged, but now I think the government provided right for infant and this could be helpfu

1 Codes:

● Benefits of mHealth: Help_professional

Comment: by Girma Gilano

> *3/12/2023 11:46:46 AM, merged with Effectiviness 3/12/2023 1:12:09 PM, merged with Decision_making 3/12/2023 1:12:55 PM, merged with Role_improvement*

30 Quotations:

1:24 ¶ 34, We always give best service to our best capacity, but mHealth may adva… in interview analysis / 1:34 ¶ 39, As HEWs we cannot reach for every mother on time but text can easily r… in interview analysis / 1:36 ¶ 40, This mHealth better than our previous our service in interview analysis / 1:50 ¶ 57, further improve the already improving service in interview analysis / 1:56 ¶ 62, Our current ANC and other formats now request mother to register their… in interview analysis / 1:68 ¶ 70, Every system is getting digitalized so digitalizing health may have mu… in interview analysis / 1:82 ¶ 81, ers usually take what we tell them, it will be successful. I don't thi… in interview analysis / 2:3 ¶ 11, It is helpful to also send information which are not appropriate in pe… in KII / 2:7 ¶ 17, Yeah, it will have effective change on community in KII / 2:9 ¶ 19, Yeah, previously mothers use the information they get during ANC, but… in KII / 2:35 ¶ 54, Since we have many gaps this should be seen as an opportunity in KII / 3:2 ¶ 3, Previous teaching of mother through home to home is going to be throug… in Group discussion / 3:12 ¶ 8, For educated mothers messages will be option less in Group discussion / 3:13 ¶ 8, Starting from me we feed additional food at 2months because the kind o… in Group discussion / 3:18 ¶ 12, It looks like mothers are tired of current service provision so the mH… in Group discussion / 3:25 ¶ 14, There may be emerged women who are successful because of mHealth and t… in Group discussion / 3:29 ¶ 17, As a health professionals applying technology can only easy our burden… in Group discussion / 3:34 ¶ 19, ecause of mHealth we may every address of women and now we can also ca… in Group discussion / 3:35 ¶ 20, We will provide the service for mothers we know now, but previously we… in Group discussion / 3:38 ¶ 22, Of course having organized maternal data can also promote contacting a… in Group discussion / 3:41 ¶ 26, : It is a big opportunity to meet the patient again virtually especial… in Group discussion / 3:42 ¶ 26, Traditionally, when errors happen or if the patients go with the wrong… in Group discussion / 3:43 ¶ 28, At the end this is very important piece of technology that can reduce… in Group discussion / 3:46 ¶ 28, Those infrastructure and awareness creation in staffs, mothers, and co… in Group discussion / 3:52 ¶ 36, People will enjoy talking to others how health institution just concer… in Group discussion / 3:57 ¶ 42, Improve work environment as per the need of the technology and owning… in Group discussion / 3:58 ¶ 43, Aspiring more technologies and helping mothers, taking trainings, givi… in Group discussion / 3:60 ¶ 46, Experience with technology. Enable them to identify areas where mHealt… in Group discussion / 3:61 ¶ 47, Planning, decision making based on mHealth, and increased service take… in Group discussion / 3:62 ¶ 48, Support related to mHealth, fulfillment of deficiencies of human and o… in Group discussion

3:18 ¶ 12 in Group discussion

Text quotation

Created by Girma Gilano on 3/9/2023

It looks like mothers are tired of current service provision so the mHealth may be liked by them

3 Codes:

● Benefits of mHealth: Help_mothers

Comment: by Girma Gilano

> *3/12/2023 11:46:46 AM, merged with Effectiviness 3/12/2023 1:12:09 PM, merged with Decision_making 3/12/2023 1:12:55 PM, merged with Role_improvement*

27 Quotations:

1:41 ¶ 47, ion comes after understanding of benefits and harms in interview analysis / 1:45 ¶ 52, Accessibility can be improved mothers get information at their homes in interview analysis / 1:51 ¶ 58, She can access any information in interview analysis / 1:53 ¶ 59, I think mHealth can improve access, appointment on time, availability… in interview analysis / 1:65 ¶ 67, Her husband or children will also push her to go even if the condition… in interview analysis / 1:68 ¶ 70, Every system is getting digitalized so digitalizing health may have mu… in interview analysis / 1:77 ¶ 77, Rural mothers are respectful; they do what they told to do, so fear of… in interview analysis / 1:81 ¶ 80, pe mothers will turn to use mobiles, but only after getting its import… in interview analysis / 2:3 ¶ 11, It is helpful to also send information which are not appropriate in pe… in KII / 2:7 ¶ 17, Yeah, it will have effective change on community in KII / 2:9 ¶ 19, Yeah, previously mothers use the information they get during ANC, but… in KII / 2:11 ¶ 21, Information access, no transportation cost, and no professional resour… in KII / 2:36 ¶ 55, No need of motor or vehicles because it is mHealth. I will be happy to… in KII / 3:6 ¶ 5, They difference for mHealth is mother always get counseling or health… in Group discussion / 3:18 ¶ 12, It looks like mothers are tired of current service provision so the mH… in Group discussion / 3:23 ¶ 14, Previously, people think poor health system and there are many complia… in Group discussion / 3:24 ¶ 14, Over time this can change community perception and they may reebok mot… in Group discussion / 3:25 ¶ 14, There may be emerged women who are successful because of mHealth and t… in Group discussion / 3:27 ¶ 16, It can make the child health growth, for mothers to know danger signs,… in Group discussion / 3:38 ¶ 22, Of course having organized maternal data can also promote contacting a… in Group discussion / 3:41 ¶ 26, : It is a big opportunity to meet the patient again virtually especial… in Group discussion / 3:42 ¶ 26, Traditionally, when errors happen or if the patients go with the wrong… in Group discussion / 3:50 ¶ 33, Benefit for mothers for next pregnancies and healthy prospect for chil… in Group discussion / 3:59 ¶ 45, Satisfaction of their client service and familiarization with technolo… in Group discussion / 3:61 ¶ 47, Planning, decision making based on mHealth, and increased service take… in Group discussion / 3:62 ¶ 48, Support related to mHealth, fulfillment of deficiencies of human and o… in Group discussion / 3:67 ¶ 53, he respect for pregnancy mothers may think gave many births and not ca… in Group discussion

● Benefits of mHealth: Help_professional

Comment: by Girma Gilano

> *3/12/2023 11:46:46 AM, merged with Effectiviness 3/12/2023 1:12:09 PM, merged with Decision_making 3/12/2023 1:12:55 PM, merged with Role_improvement*

30 Quotations:

1:24 ¶ 34, We always give best service to our best capacity, but mHealth may adva… in interview analysis / 1:34 ¶ 39, As HEWs we cannot reach for every mother on time but text can easily r… in interview analysis / 1:36 ¶ 40, This mHealth better than our previous our service in interview analysis / 1:50 ¶ 57, further improve the already improving service in interview analysis / 1:56 ¶ 62, Our current ANC and other formats now request mother to register their… in interview analysis / 1:68 ¶ 70, Every system is getting digitalized so digitalizing health may have mu… in interview analysis / 1:82 ¶ 81, ers usually take what we tell them, it will be successful. I don’t thi… in interview analysis / 2:3 ¶ 11, It is helpful to also send information which are not appropriate in pe… in KII / 2:7 ¶ 17, Yeah, it will have effective change on community in KII / 2:9 ¶ 19, Yeah, previously mothers use the information they get during ANC, but… in KII / 2:35 ¶ 54, Since we have many gaps this should be seen as an opportunity in KII / 3:2 ¶ 3, Previous teaching of mother through home to home is going to be throug… in Group discussion / 3:12 ¶ 8, For educated mothers messages will be option less in Group discussion / 3:13 ¶ 8, Starting from me we feed additional food at 2months because the kind o… in Group discussion / 3:18 ¶ 12, It looks like mothers are tired of current service provision so the mH… in Group discussion / 3:25 ¶ 14, There may be emerged women who are successful because of mHealth and t… in Group discussion / 3:29 ¶ 17, As a health professionals applying technology can only easy our burden… in Group discussion / 3:34 ¶ 19, ecause of mHealth we may every address of women and now we can also ca… in Group discussion / 3:35 ¶ 20, We will provide the service for mothers we know now, but previously we… in Group discussion / 3:38 ¶ 22, Of course having organized maternal data can also promote

contacting a… in Group discussion / 3:41 ¶ 26, : It is a big opportunity to meet the patient again virtually especial… in Group discussion / 3:42 ¶ 26, Traditionally, when errors happen or if the patients go with the wrong… in Group discussion / 3:43 ¶ 28, At the end this is very important piece of technology that can reduce… in Group discussion / 3:46 ¶ 28, Those infrastructure and awareness creation in staffs, mothers, and co… in Group discussion / 3:52 ¶ 36, People will enjoy talking to others how health institution just concer… in Group discussion / 3:57 ¶ 42, Improve work environment as per the need of the technology and owning… in Group discussion / 3:58 ¶ 43, Aspiring more technologies and helping mothers, taking trainings, givi… in Group discussion / 3:60 ¶ 46, Experience with technology. Enable them to identify areas where mHealt… in Group discussion / 3:61 ¶ 47, Planning, decision making based on mHealth, and increased service take… in Group discussion / 3:62 ¶ 48, Support related to mHealth, fulfillment of deficiencies of human and o… in Group discussion

- **Benefits of mHealth: Improve_MCH**

Comment: by Girma Gilano

> *3/12/2023 11:46:46 AM, merged with Effectiviness 3/12/2023 1:12:09 PM, merged with Decision_making 3/12/2023 1:12:55 PM, merged with Role_improvement*

25 Quotations:

1:6 ¶ 22, Non-use of the service because of negligence and forgetting can be im… in interview analysis / 1:26 ¶ 35, t can increase utilization better than that we have previously in interview analysis / 1:28 ¶ 36, Even since this digital it can further improve the service and increas… in interview analysis / 1:36 ¶ 40, This mHealth better than our previous our service in interview analysis / 1:43 ¶ 50, For maternal and child health the decision to take service is mothers… in interview analysis / 1:49 ¶ 56, Having information on time can improve the health of mothers and child… in interview analysis / 1:50 ¶ 57, further improve the already improving service in interview analysis / 1:57 ¶ 63, f it continue, it can be effective and be important than previous way… in interview analysis / 1:59 ¶ 64, I think whatever we put in to the community to improve health service… in interview analysis / 1:62 ¶ 66, This can improve and put our usual service one step forward in interview analysis / 1:74 ¶ 75, I don't think people can be affected negatively because they love to l… in interview analysis / 1:77 ¶ 77, Rural mothers are respectful; they do what they told to do, so fear of… in interview analysis / 1:78 ¶ 78, One thing I assure is it will increase up taking maternal and child he… in interview analysis / 1:83 ¶ 83, It can remind which is especially important for family planning. Witho… in interview analysis / 2:4 ¶ 12, If health system organized this way it will be helpful in KII / 2:6 ¶ 16, this is the main thing to improve maternal and child care especially u… in KII / 3:8 ¶ 6, After counseling and aware mother it can be improved in Group discussion / 3:9 ¶ 8, It can improve those listed service (ANC, PNC, Breastfeeding and vacci… in Group discussion / 3:10 ¶ 8, Mother are already exposed to health education, but this one is direct… in Group discussion / 3:18 ¶ 12, It looks like mothers are tired of current service provision so the mH… in Group discussion / 3:19 ¶ 12, So in that case it can completely change the stream and interest. Or t… in Group discussion / 3:20 ¶ 12, They may even consider the increased risk of danger related to pregnan… in Group discussion / 3:43 ¶ 28, At the end this is very important piece of technology that can reduce… in Group discussion / 3:48 ¶ 31, Service at their home and Information and on time vaccination and Heal… in Group discussion / 3:49 ¶ 32, Healthy child feeding and behavioral change for mother that wll be lon… in Group discussion

3:25 ¶ 14 in Group discussion

Text quotation

Created by Girma Gilano on 3/9/2023

There may be emerged women who are successful because of mHealth and they may tell the story of mHealth which further educate the community

2 Codes:

- **Benefits of mHealth: Help_mothers**

Comment: by Girma Gilano

> *3/12/2023 11:46:46 AM, merged with Effectiviness 3/12/2023 1:12:09 PM, merged with Decision_making 3/12/2023 1:12:55 PM, merged with Role_improvement*

27 Quotations:

1:41 ¶ 47, ion comes after understanding of benefits and harms in interview analysis / 1:45 ¶ 52, Accessibility can be improved mothers get information at their homes in interview analysis / 1:51 ¶ 58, She can access any information in interview analysis / 1:53 ¶ 59, I think mHealth can improve access, appointment on time, availability… in interview analysis / 1:65 ¶ 67, Her husband or children will also push her to go even if the condition… in interview analysis / 1:68 ¶ 70, Every system is getting digitalized so digitalizing health may have mu… in interview analysis / 1:77 ¶ 77, Rural mothers are respectful; they do what they told to do, so fear of… in interview analysis / 1:81 ¶ 80, pe mothers will turn to use mobiles, but only after getting its import… in interview analysis / 2:3 ¶ 11, It is helpful to also send information which are not appropriate in pe… in KII / 2:7 ¶ 17, Yeah, it will have effective change on community in KII / 2:9 ¶ 19, Yeah, previously mothers use the information they get during ANC, but… in KII / 2:11 ¶ 21, Information access, no transportation cost, and no professional resour… in KII / 2:36 ¶ 55, No need of motor or vehicles because it is mHealth. I will be happy to… in KII / 3:6 ¶ 5, They difference for mHealth is mother always get counseling or health… in Group discussion / 3:18 ¶ 12, It looks like mothers are tired of current service provision so the mH… in Group discussion / 3:23 ¶ 14, Previously, people think poor health system and there are many complia… in Group discussion / 3:24 ¶ 14, Over time this can change community perception and they may reebok mot… in Group discussion / 3:25 ¶ 14, There may be emerged women who are successful because of mHealth and t… in Group discussion / 3:27 ¶ 16, It can make the child health growth, for mothers to know danger signs,… in Group discussion / 3:38 ¶ 22, Of course having organized maternal data can also promote contacting a… in Group discussion / 3:41 ¶ 26, : It is a big opportunity to meet the patient again virtually especial… in Group discussion / 3:42 ¶ 26, Traditionally, when errors happen or if the patients go with the wrong… in Group discussion / 3:50 ¶ 33, Benefit for mothers for next pregnancies and healthy prospect for chil… in Group discussion / 3:59 ¶ 45, Satisfaction of their client service and familiarization with technolo… in Group discussion / 3:61 ¶ 47, Planning, decision making based on mHealth, and increased service take… in Group discussion / 3:62 ¶ 48, Support related to mHealth, fulfillment of deficiencies of human and o… in Group discussion / 3:67 ¶ 53, he respect for pregnancy mothers may think gave many births and not ca… in Group discussion

● Benefits of mHealth: Help_professional

Comment: by Girma Gilano

> *3/12/2023 11:46:46 AM, merged with Effectiviness 3/12/2023 1:12:09 PM, merged with Decision_making 3/12/2023 1:12:55 PM, merged with Role_improvement*

30 Quotations:

1:24 ¶ 34, We always give best service to our best capacity, but mHealth may adva… in interview analysis / 1:34 ¶ 39, As HEWs we cannot reach for every mother on time but text can easily r… in interview analysis / 1:36 ¶ 40, This mHealth better than our previous our service in interview analysis / 1:50 ¶ 57, further improve the already improving service in interview analysis / 1:56 ¶ 62, Our current ANC and other formats now request mother to register their… in interview analysis / 1:68 ¶ 70, Every system is getting digitalized so digitalizing health may have mu… in interview analysis / 1:82 ¶ 81, ers usually take what we tell them, it will be successful. I don't thi… in interview analysis / 2:3 ¶ 11, It is helpful to also send information which are not appropriate in pe… in KII / 2:7 ¶ 17, Yeah, it will have effective change on community in KII / 2:9 ¶ 19, Yeah, previously mothers use the information they get during ANC, but… in KII / 2:35 ¶ 54, Since we have many gaps this should be seen as an opportunity in KII / 3:2 ¶ 3, Previous teaching of mother through home to home is going to be throug… in Group discussion / 3:12 ¶ 8, For educated mothers messages will be option less in Group discussion / 3:13 ¶ 8, Starting from me we feed additional food at 2months because the kind o… in Group discussion / 3:18 ¶ 12, It looks like mothers are tired of current service provision so the mH… in Group discussion / 3:25 ¶ 14, There may be emerged women who are successful because of mHealth and t… in Group discussion / 3:29 ¶ 17, As a health professionals applying technology can only easy our burden… in Group discussion / 3:34 ¶ 19, ecause of mHealth we may every address of women and now we can also ca… in Group discussion / 3:35 ¶ 20, We will provide the service for mothers we know now, but previously we… in Group discussion / 3:38 ¶ 22, Of course having organized maternal data can also promote

contacting a… in Group discussion / 3:41 ¶ 26, : It is a big opportunity to meet the patient again virtually especial… in Group discussion / 3:42 ¶ 26, Traditionally, when errors happen or if the patients go with the wrong… in Group discussion / 3:43 ¶ 28, At the end this is very important piece of technology that can reduce… in Group discussion / 3:46 ¶ 28, Those infrastructure and awareness creation in staffs, mothers, and co… in Group discussion / 3:52 ¶ 36, People will enjoy talking to others how health institution just concer… in Group discussion / 3:57 ¶ 42, Improve work environment as per the need of the technology and owning… in Group discussion / 3:58 ¶ 43, Aspiring more technologies and helping mothers, taking trainings, givi… in Group discussion / 3:60 ¶ 46, Experience with technology. Enable them to identify areas where mHealt… in Group discussion / 3:61 ¶ 47, Planning, decision making based on mHealth, and increased service take… in Group discussion / 3:62 ¶ 48, Support related to mHealth, fulfillment of deficiencies of human and o… in Group discussion

3:29 ¶ 17 in Group discussion

Text quotation

Created by Girma Gilano on 3/9/2023

As a health professionals applying technology can only easy our burden of job. The main thing in this service is mothers having mobile

1 Codes:

- **Benefits of mHealth: Help_professional**

Comment: by Girma Gilano

> *3/12/2023 11:46:46 AM, merged with Effectiviness 3/12/2023 1:12:09 PM, merged with Decision_making 3/12/2023 1:12:55 PM, merged with Role_improvement*

30 Quotations:

1:24 ¶ 34, We always give best service to our best capacity, but mHealth may adva… in interview analysis / 1:34 ¶ 39, As HEWs we cannot reach for every mother on time but text can easily r… in interview analysis / 1:36 ¶ 40, This mHealth better than our previous our service in interview analysis / 1:50 ¶ 57, further improve the already improving service in interview analysis / 1:56 ¶ 62, Our current ANC and other formats now request mother to register their… in interview analysis / 1:68 ¶ 70, Every system is getting digitalized so digitalizing health may have mu… in interview analysis / 1:82 ¶ 81, ers usually take what we tell them, it will be successful. I don't thi… in interview analysis / 2:3 ¶ 11, It is helpful to also send information which are not appropriate in pe… in KII / 2:7 ¶ 17, Yeah, it will have effective change on community in KII / 2:9 ¶ 19, Yeah, previously mothers use the information they get during ANC, but… in KII / 2:35 ¶ 54, Since we have many gaps this should be seen as an opportunity in KII / 3:2 ¶ 3, Previous teaching of mother through home to home is going to be throug… in Group discussion / 3:12 ¶ 8, For educated mothers messages will be option less in Group discussion / 3:13 ¶ 8, Starting from me we feed additional food at 2months because the kind o… in Group discussion / 3:18 ¶ 12, It looks like mothers are tired of current service provision so the mH… in Group discussion / 3:25 ¶ 14, There may be emerged women who are successful because of mHealth and t… in Group discussion / 3:29 ¶ 17, As a health professionals applying technology can only easy our burden… in Group discussion / 3:34 ¶ 19, ecause of mHealth we may every address of women and now we can also ca… in Group discussion / 3:35 ¶ 20, We will provide the service for mothers we know now, but previously we… in Group discussion / 3:38 ¶ 22, Of course having organized maternal data can also promote contacting a… in Group discussion / 3:41 ¶ 26, : It is a big opportunity to meet the patient again virtually especial… in Group discussion / 3:42 ¶ 26, Traditionally, when errors happen or if the patients go with the wrong… in Group discussion / 3:43 ¶ 28, At the end this is very important piece of technology that can reduce… in Group discussion / 3:46 ¶ 28, Those infrastructure and awareness creation in staffs, mothers, and co… in Group discussion / 3:52 ¶ 36, People will enjoy talking to others how health institution just concer… in Group discussion / 3:57 ¶ 42, Improve work environment as per the need of the technology and owning… in Group discussion / 3:58 ¶ 43, Aspiring more technologies and helping mothers, taking trainings, givi… in Group discussion / 3:60 ¶ 46, Experience with technology. Enable

them to identify areas where mHealt… in Group discussion / 3:61 ¶ 47, Planning, decision making based on mHealth, and increased service take… in Group discussion / 3:62 ¶ 48, Support related to mHealth, fulfillment of deficiencies of human and o… in Group discussion

3:34 ¶ 19 in Group discussion

Text quotation

Created by Girma Gilano on 3/9/2023

ecause of mHealth we may every address of women and now we can also call them when necessary. It will be very helpful specially organized service provision. Everyone respect health professionals so hopefully mother s also well respond to our requests

1 Codes:

- **Benefits of mHealth: Help_professional**

Comment: by Girma Gilano

> *3/12/2023 11:46:46 AM, merged with Effectiviness 3/12/2023 1:12:09 PM, merged with Decision_making 3/12/2023 1:12:55 PM, merged with Role_improvement*

30 Quotations:

1:24 ¶ 34, We always give best service to our best capacity, but mHealth may adva… in interview analysis / 1:34 ¶ 39, As HEWs we cannot reach for every mother on time but text can easily r… in interview analysis / 1:36 ¶ 40, This mHealth better than our previous our service in interview analysis / 1:50 ¶ 57, further improve the already improving service in interview analysis / 1:56 ¶ 62, Our current ANC and other formats now request mother to register their… in interview analysis / 1:68 ¶ 70, Every system is getting digitalized so digitalizing health may have mu… in interview analysis / 1:82 ¶ 81, ers usually take what we tell them, it will be successful. I don't thi… in interview analysis / 2:3 ¶ 11, It is helpful to also send information which are not appropriate in pe… in KII / 2:7 ¶ 17, Yeah, it will have effective change on community in KII / 2:9 ¶ 19, Yeah, previously mothers use the information they get during ANC, but… in KII / 2:35 ¶ 54, Since we have many gaps this should be seen as an opportunity in KII / 3:2 ¶ 3, Previous teaching of mother through home to home is going to be throug… in Group discussion / 3:12 ¶ 8, For educated mothers messages will be option less in Group discussion / 3:13 ¶ 8, Starting from me we feed additional food at 2months because the kind o… in Group discussion / 3:18 ¶ 12, It looks like mothers are tired of current service provision so the mH… in Group discussion / 3:25 ¶ 14, There may be emerged women who are successful because of mHealth and t… in Group discussion / 3:29 ¶ 17, As a health professionals applying technology can only easy our burden… in Group discussion / 3:34 ¶ 19, ecause of mHealth we may every address of women and now we can also ca… in Group discussion / 3:35 ¶ 20, We will provide the service for mothers we know now, but previously we… in Group discussion / 3:38 ¶ 22, Of course having organized maternal data can also promote contacting a… in Group discussion / 3:41 ¶ 26, : It is a big opportunity to meet the patient again virtually especial… in Group discussion / 3:42 ¶ 26, Traditionally, when errors happen or if the patients go with the wrong… in Group discussion / 3:43 ¶ 28, At the end this is very important piece of technology that can reduce… in Group discussion / 3:46 ¶ 28, Those infrastructure and awareness creation in staffs, mothers, and co… in Group discussion / 3:52 ¶ 36, People will enjoy talking to others how health institution just concer… in Group discussion / 3:57 ¶ 42, Improve work environment as per the need of the technology and owning… in Group discussion / 3:58 ¶ 43, Aspiring more technologies and helping mothers, taking trainings, givi… in Group discussion / 3:60 ¶ 46, Experience with technology. Enable them to identify areas where mHealt… in Group discussion / 3:61 ¶ 47, Planning, decision making based on mHealth, and increased service take… in Group discussion / 3:62 ¶ 48, Support related to mHealth, fulfillment of deficiencies of human and o… in Group discussion

3:35 ¶ 20 in Group discussion

Text quotation

Created by Girma Gilano on 3/9/2023

We will provide the service for mothers we know now, but previously we just provide service for whoever coming and do not those who are not coming

1 Codes:

- **Benefits of mHealth: Help_professional**

  Comment: by Girma Gilano

  > *3/12/2023 11:46:46 AM, merged with Effectiviness 3/12/2023 1:12:09 PM, merged with Decision_making 3/12/2023 1:12:55 PM, merged with Role_improvement*

  30 Quotations:

  1:24 ¶ 34, We always give best service to our best capacity, but mHealth may adva… in interview analysis / 1:34 ¶ 39, As HEWs we cannot reach for every mother on time but text can easily r… in interview analysis / 1:36 ¶ 40, This mHealth better than our previous our service in interview analysis / 1:50 ¶ 57, further improve the already improving service in interview analysis / 1:56 ¶ 62, Our current ANC and other formats now request mother to register their… in interview analysis / 1:68 ¶ 70, Every system is getting digitalized so digitalizing health may have mu… in interview analysis / 1:82 ¶ 81, ers usually take what we tell them, it will be successful. I don't thi… in interview analysis / 2:3 ¶ 11, It is helpful to also send information which are not appropriate in pe… in KII / 2:7 ¶ 17, Yeah, it will have effective change on community in KII / 2:9 ¶ 19, Yeah, previously mothers use the information they get during ANC, but… in KII / 2:35 ¶ 54, Since we have many gaps this should be seen as an opportunity in KII / 3:2 ¶ 3, Previous teaching of mother through home to home is going to be throug… in Group discussion / 3:12 ¶ 8, For educated mothers messages will be option less in Group discussion / 3:13 ¶ 8, Starting from me we feed additional food at 2months because the kind o… in Group discussion / 3:18 ¶ 12, It looks like mothers are tired of current service provision so the mH… in Group discussion / 3:25 ¶ 14, There may be emerged women who are successful because of mHealth and t… in Group discussion / 3:29 ¶ 17, As a health professionals applying technology can only easy our burden… in Group discussion / 3:34 ¶ 19, ecause of mHealth we may every address of women and now we can also ca… in Group discussion / 3:35 ¶ 20, We will provide the service for mothers we know now, but previously we… in Group discussion / 3:38 ¶ 22, Of course having organized maternal data can also promote contacting a… in Group discussion / 3:41 ¶ 26, : It is a big opportunity to meet the patient again virtually especial… in Group discussion / 3:42 ¶ 26, Traditionally, when errors happen or if the patients go with the wrong… in Group discussion / 3:43 ¶ 28, At the end this is very important piece of technology that can reduce… in Group discussion / 3:46 ¶ 28, Those infrastructure and awareness creation in staffs, mothers, and co… in Group discussion / 3:52 ¶ 36, People will enjoy talking to others how health institution just concer… in Group discussion / 3:57 ¶ 42, Improve work environment as per the need of the technology and owning… in Group discussion / 3:58 ¶ 43, Aspiring more technologies and helping mothers, taking trainings, givi… in Group discussion / 3:60 ¶ 46, Experience with technology. Enable them to identify areas where mHealt… in Group discussion / 3:61 ¶ 47, Planning, decision making based on mHealth, and increased service take… in Group discussion / 3:62 ¶ 48, Support related to mHealth, fulfillment of deficiencies of human and o… in Group discussion

3:38 ¶ 22 in Group discussion

Text quotation

Created by Girma Gilano on 3/9/2023

Of course having organized maternal data can also promote contacting atleast risky mothers even without funds.

2 Codes:

● Benefits of mHealth: Help_mothers

Comment: by Girma Gilano

> *3/12/2023 11:46:46 AM, merged with Effectiviness 3/12/2023 1:12:09 PM, merged with Decision_making 3/12/2023 1:12:55 PM, merged with Role_improvement*

27 Quotations:

1:41 ¶ 47, ion comes after understanding of benefits and harms in interview analysis / 1:45 ¶ 52, Accessibility can be improved mothers get information at their homes in interview analysis / 1:51 ¶ 58, She can access any information in interview analysis / 1:53 ¶ 59, I think mHealth can improve access, appointment on time, availability… in interview analysis / 1:65 ¶ 67, Her husband or children will also push her to go even if the condition… in interview analysis / 1:68 ¶ 70, Every system is getting digitalized so digitalizing health may have mu… in interview analysis / 1:77 ¶ 77, Rural mothers are respectful; they do what they told to do, so fear of… in interview analysis / 1:81 ¶ 80, pe mothers will turn to use mobiles, but only after getting its import… in interview analysis / 2:3 ¶ 11, It is helpful to also send information which are not appropriate in pe… in KII / 2:7 ¶ 17, Yeah, it will have effective change on community in KII / 2:9 ¶ 19, Yeah, previously mothers use the information they get during ANC, but… in KII / 2:11 ¶ 21, Information access, no transportation cost, and no professional resour… in KII / 2:36 ¶ 55, No need of motor or vehicles because it is mHealth. I will be happy to… in KII / 3:6 ¶ 5, They difference for mHealth is mother always get counseling or health… in Group discussion / 3:18 ¶ 12, It looks like mothers are tired of current service provision so the mH… in Group discussion / 3:23 ¶ 14, Previously, people think poor health system and there are many complia… in Group discussion / 3:24 ¶ 14, Over time this can change community perception and they may reebok mot… in Group discussion / 3:25 ¶ 14, There may be emerged women who are successful because of mHealth and t… in Group discussion / 3:27 ¶ 16, It can make the child health growth, for mothers to know danger signs,… in Group discussion / 3:38 ¶ 22, Of course having organized maternal data can also promote contacting a… in Group discussion / 3:41 ¶ 26, : It is a big opportunity to meet the patient again virtually especial… in Group discussion / 3:42 ¶ 26, Traditionally, when errors happen or if the patients go with the wrong… in Group discussion / 3:50 ¶ 33, Benefit for mothers for next pregnancies and healthy prospect for chil… in Group discussion / 3:59 ¶ 45, Satisfaction of their client service and familiarization with technolo… in Group discussion / 3:61 ¶ 47, Planning, decision making based on mHealth, and increased service take… in Group discussion / 3:62 ¶ 48, Support related to mHealth, fulfillment of deficiencies of human and o… in Group discussion / 3:67 ¶ 53, he respect for pregnancy mothers may think gave many births and not ca… in Group discussion

● Benefits of mHealth: Help_professional

Comment: by Girma Gilano

> *3/12/2023 11:46:46 AM, merged with Effectiviness 3/12/2023 1:12:09 PM, merged with Decision_making 3/12/2023 1:12:55 PM, merged with Role_improvement*

30 Quotations:

1:24 ¶ 34, We always give best service to our best capacity, but mHealth may adva… in interview analysis / 1:34 ¶ 39, As HEWs we cannot reach for every mother on time but text can easily r… in interview analysis / 1:36 ¶ 40, This mHealth better than our previous our service in interview analysis / 1:50 ¶ 57, further improve the already improving service in interview analysis / 1:56 ¶ 62, Our current ANC and other formats now request mother to register their… in interview analysis / 1:68 ¶ 70, Every system is getting digitalized so digitalizing health may have mu… in interview analysis / 1:82 ¶ 81, ers usually take what we tell them, it will be successful. I don't thi… in interview analysis / 2:3 ¶ 11, It is helpful to also send information which are not appropriate in pe… in KII / 2:7 ¶ 17, Yeah, it will have effective change on community in KII / 2:9 ¶ 19, Yeah, previously mothers use the information they get during ANC, but… in KII / 2:35 ¶ 54, Since we have many gaps this should be seen as an opportunity in KII / 3:2 ¶ 3, Previous teaching of mother through home to home is going to be throug… in Group discussion / 3:12 ¶ 8, For educated mothers messages will be option less in Group discussion / 3:13 ¶ 8, Starting from me we feed additional food at 2months because the kind o… in Group discussion / 3:18 ¶ 12, It looks like mothers are tired of current service provision so the mH… in Group discussion / 3:25 ¶

14, There may be emerged women who are successful because of mHealth and t… in Group discussion / 3:29 ¶ 17, As a health professionals applying technology can only easy our burden… in Group discussion / 3:34 ¶ 19, ecause of mHealth we may every address of women and now we can also ca… in Group discussion / 3:35 ¶ 20, We will provide the service for mothers we know now, but previously we… in Group discussion / 3:38 ¶ 22, Of course having organized maternal data can also promote contacting a… in Group discussion / 3:41 ¶ 26, : It is a big opportunity to meet the patient again virtually especial… in Group discussion / 3:42 ¶ 26, Traditionally, when errors happen or if the patients go with the wrong… in Group discussion / 3:43 ¶ 28, At the end this is very important piece of technology that can reduce… in Group discussion / 3:46 ¶ 28, Those infrastructure and awareness creation in staffs, mothers, and co… in Group discussion / 3:52 ¶ 36, People will enjoy talking to others how health institution just concer… in Group discussion / 3:57 ¶ 42, Improve work environment as per the need of the technology and owning… in Group discussion / 3:58 ¶ 43, Aspiring more technologies and helping mothers, taking trainings, givi… in Group discussion / 3:60 ¶ 46, Experience with technology. Enable them to identify areas where mHealt… in Group discussion / 3:61 ¶ 47, Planning, decision making based on mHealth, and increased service take… in Group discussion / 3:62 ¶ 48, Support related to mHealth, fulfillment of deficiencies of human and o… in Group discussion

3:41 ¶ 26 in Group discussion

Text quotation

Created by Girma Gilano on 3/9/2023, **modified** by Girma Gilano on 3/9/2023

: It is a big opportunity to meet the patient again virtually especially when you missed something in person. Consultation normally should not be a onetime activity rather an effective consultation should be continuous.

2 Codes:

- **Benefits of mHealth: Help_mothers**

Comment: by Girma Gilano

> *3/12/2023 11:46:46 AM, merged with Effectiviness 3/12/2023 1:12:09 PM, merged with Decision_making 3/12/2023 1:12:55 PM, merged with Role_improvement*

27 Quotations:

1:41 ¶ 47, ion comes after understanding of benefits and harms in interview analysis / 1:45 ¶ 52, Accessibility can be improved mothers get information at their homes in interview analysis / 1:51 ¶ 58, She can access any information in interview analysis / 1:53 ¶ 59, I think mHealth can improve access, appointment on time, availability… in interview analysis / 1:65 ¶ 67, Her husband or children will also push her to go even if the condition… in interview analysis / 1:68 ¶ 70, Every system is getting digitalized so digitalizing health may have mu… in interview analysis / 1:77 ¶ 77, Rural mothers are respectful; they do what they told to do, so fear of… in interview analysis / 1:81 ¶ 80, pe mothers will turn to use mobiles, but only after getting its import… in interview analysis / 2:3 ¶ 11, It is helpful to also send information which are not appropriate in pe… in KII / 2:7 ¶ 17, Yeah, it will have effective change on community in KII / 2:9 ¶ 19, Yeah, previously mothers use the information they get during ANC, but… in KII / 2:11 ¶ 21, Information access, no transportation cost, and no professional resour… in KII / 2:36 ¶ 55, No need of motor or vehicles because it is mHealth. I will be happy to… in KII / 3:6 ¶ 5, They difference for mHealth is mother always get counseling or health… in Group discussion / 3:18 ¶ 12, It looks like mothers are tired of current service provision so the mH… in Group discussion / 3:23 ¶ 14, Previously, people think poor health system and there are many complia… in Group discussion / 3:24 ¶ 14, Over time this can change community perception and they may reebok mot… in Group discussion / 3:25 ¶ 14, There may be emerged women who are successful because of mHealth and t… in Group discussion / 3:27 ¶ 16, It can make the child health growth, for mothers to know danger signs,… in Group discussion / 3:38 ¶ 22, Of course having organized maternal data can also promote contacting a… in Group discussion / 3:41 ¶ 26, : It is a big opportunity to meet the patient again virtually especial… in Group discussion / 3:42 ¶ 26, Traditionally, when errors happen or if the patients go with the wrong… in Group discussion / 3:50 ¶ 33,

Benefit for mothers for next pregnancies and healthy prospect for chil… in Group discussion / 3:59 ¶ 45, Satisfaction of their client service and familiarization with technolo… in Group discussion / 3:61 ¶ 47, Planning, decision making based on mHealth, and increased service take… in Group discussion / 3:62 ¶ 48, Support related to mHealth, fulfillment of deficiencies of human and o… in Group discussion / 3:67 ¶ 53, he respect for pregnancy mothers may think gave many births and not ca… in Group discussion

● Benefits of mHealth: Help_professional

Comment: by Girma Gilano

> *3/12/2023 11:46:46 AM, merged with Effectiviness 3/12/2023 1:12:09 PM, merged with Decision_making 3/12/2023 1:12:55 PM, merged with Role_improvement*

30 Quotations:

1:24 ¶ 34, We always give best service to our best capacity, but mHealth may adva… in interview analysis / 1:34 ¶ 39, As HEWs we cannot reach for every mother on time but text can easily r… in interview analysis / 1:36 ¶ 40, This mHealth better than our previous our service in interview analysis / 1:50 ¶ 57, further improve the already improving service in interview analysis / 1:56 ¶ 62, Our current ANC and other formats now request mother to register their… in interview analysis / 1:68 ¶ 70, Every system is getting digitalized so digitalizing health may have mu… in interview analysis / 1:82 ¶ 81, ers usually take what we tell them, it will be successful. I don't thi… in interview analysis / 2:3 ¶ 11, It is helpful to also send information which are not appropriate in pe… in KII / 2:7 ¶ 17, Yeah, it will have effective change on community in KII / 2:9 ¶ 19, Yeah, previously mothers use the information they get during ANC, but… in KII / 2:35 ¶ 54, Since we have many gaps this should be seen as an opportunity in KII / 3:2 ¶ 3, Previous teaching of mother through home to home is going to be throug… in Group discussion / 3:12 ¶ 8, For educated mothers messages will be option less in Group discussion / 3:13 ¶ 8, Starting from me we feed additional food at 2months because the kind o… in Group discussion / 3:18 ¶ 12, It looks like mothers are tired of current service provision so the mH… in Group discussion / 3:25 ¶ 14, There may be emerged women who are successful because of mHealth and t… in Group discussion / 3:29 ¶ 17, As a health professionals applying technology can only easy our burden… in Group discussion / 3:34 ¶ 19, ecause of mHealth we may every address of women and now we can also ca… in Group discussion / 3:35 ¶ 20, We will provide the service for mothers we know now, but previously we… in Group discussion / 3:38 ¶ 22, Of course having organized maternal data can also promote contacting a… in Group discussion / 3:41 ¶ 26, : It is a big opportunity to meet the patient again virtually especial… in Group discussion / 3:42 ¶ 26, Traditionally, when errors happen or if the patients go with the wrong… in Group discussion / 3:43 ¶ 28, At the end this is very important piece of technology that can reduce… in Group discussion / 3:46 ¶ 28, Those infrastructure and awareness creation in staffs, mothers, and co… in Group discussion / 3:52 ¶ 36, People will enjoy talking to others how health institution just concer… in Group discussion / 3:57 ¶ 42, Improve work environment as per the need of the technology and owning… in Group discussion / 3:58 ¶ 43, Aspiring more technologies and helping mothers, taking trainings, givi… in Group discussion / 3:60 ¶ 46, Experience with technology. Enable them to identify areas where mHealt… in Group discussion / 3:61 ¶ 47, Planning, decision making based on mHealth, and increased service take… in Group discussion / 3:62 ¶ 48, Support related to mHealth, fulfillment of deficiencies of human and o… in Group discussion

3:42 ¶ 26 in Group discussion

Text quotation

Created by Girma Gilano on 3/9/2023

Traditionally, when errors happen or if the patients go with the wrong drug there is no way to correct it back. It means the patient have to suffer the error and then come back if alive, but this time we have organized evidence and further messages

2 Codes:

Benefits of mHealth: Help_mothers

Comment: by Girma Gilano

> *3/12/2023 11:46:46 AM, merged with Effectiviness 3/12/2023 1:12:09 PM, merged with Decision_making 3/12/2023 1:12:55 PM, merged with Role_improvement*

27 Quotations:

1:41 ¶ 47, ion comes after understanding of benefits and harms in interview analysis / 1:45 ¶ 52, Accessibility can be improved mothers get information at their homes in interview analysis / 1:51 ¶ 58, She can access any information in interview analysis / 1:53 ¶ 59, I think mHealth can improve access, appointment on time, availability… in interview analysis / 1:65 ¶ 67, Her husband or children will also push her to go even if the condition… in interview analysis / 1:68 ¶ 70, Every system is getting digitalized so digitalizing health may have mu… in interview analysis / 1:77 ¶ 77, Rural mothers are respectful; they do what they told to do, so fear of… in interview analysis / 1:81 ¶ 80, pe mothers will turn to use mobiles, but only after getting its import… in interview analysis / 2:3 ¶ 11, It is helpful to also send information which are not appropriate in pe… in KII / 2:7 ¶ 17, Yeah, it will have effective change on community in KII / 2:9 ¶ 19, Yeah, previously mothers use the information they get during ANC, but… in KII / 2:11 ¶ 21, Information access, no transportation cost, and no professional resour… in KII / 2:36 ¶ 55, No need of motor or vehicles because it is mHealth. I will be happy to… in KII / 3:6 ¶ 5, They difference for mHealth is mother always get counseling or health… in Group discussion / 3:18 ¶ 12, It looks like mothers are tired of current service provision so the mH… in Group discussion / 3:23 ¶ 14, Previously, people think poor health system and there are many complia… in Group discussion / 3:24 ¶ 14, Over time this can change community perception and they may reebok mot… in Group discussion / 3:25 ¶ 14, There may be emerged women who are successful because of mHealth and t… in Group discussion / 3:27 ¶ 16, It can make the child health growth, for mothers to know danger signs,… in Group discussion / 3:38 ¶ 22, Of course having organized maternal data can also promote contacting a… in Group discussion / 3:41 ¶ 26, : It is a big opportunity to meet the patient again virtually especial… in Group discussion / 3:42 ¶ 26, Traditionally, when errors happen or if the patients go with the wrong… in Group discussion / 3:50 ¶ 33, Benefit for mothers for next pregnancies and healthy prospect for chil… in Group discussion / 3:59 ¶ 45, Satisfaction of their client service and familiarization with technolo… in Group discussion / 3:61 ¶ 47, Planning, decision making based on mHealth, and increased service take… in Group discussion / 3:62 ¶ 48, Support related to mHealth, fulfillment of deficiencies of human and o… in Group discussion / 3:67 ¶ 53, he respect for pregnancy mothers may think gave many births and not ca… in Group discussion

Benefits of mHealth: Help_professional

Comment: by Girma Gilano

> *3/12/2023 11:46:46 AM, merged with Effectiviness 3/12/2023 1:12:09 PM, merged with Decision_making 3/12/2023 1:12:55 PM, merged with Role_improvement*

30 Quotations:

1:24 ¶ 34, We always give best service to our best capacity, but mHealth may adva… in interview analysis / 1:34 ¶ 39, As HEWs we cannot reach for every mother on time but text can easily r… in interview analysis / 1:36 ¶ 40, This mHealth better than our previous our service in interview analysis / 1:50 ¶ 57, further improve the already improving service in interview analysis / 1:56 ¶ 62, Our current ANC and other formats now request mother to register their… in interview analysis / 1:68 ¶ 70, Every system is getting digitalized so digitalizing health may have mu… in interview analysis / 1:82 ¶ 81, ers usually take what we tell them, it will be successful. I don't thi… in interview analysis / 2:3 ¶ 11, It is helpful to also send information which are not appropriate in pe… in KII / 2:7 ¶ 17, Yeah, it will have effective change on community in KII / 2:9 ¶ 19, Yeah, previously mothers use the information they get during ANC, but… in KII / 2:35 ¶ 54, Since we have many gaps this should be seen as an opportunity in KII / 3:2 ¶ 3, Previous teaching of mother through home to home is going to be throug… in Group discussion / 3:12 ¶ 8, For educated mothers messages will be option less in Group discussion / 3:13 ¶ 8, Starting from me we feed additional food at 2months because the kind o… in Group discussion / 3:18 ¶ 12, It looks like mothers are tired of current service provision so the mH… in Group discussion / 3:25 ¶ 14, There may be emerged women who are successful because of mHealth and t… in Group discussion / 3:29 ¶ 17, As a health professionals applying technology can only easy our burden… in Group

discussion / 3:34 ¶ 19, ecause of mHealth we may every address of women and now we can also ca… in Group discussion / 3:35 ¶ 20, We will provide the service for mothers we know now, but previously we… in Group discussion / 3:38 ¶ 22, Of course having organized maternal data can also promote contacting a… in Group discussion / 3:41 ¶ 26, : It is a big opportunity to meet the patient again virtually especial… in Group discussion / 3:42 ¶ 26, Traditionally, when errors happen or if the patients go with the wrong… in Group discussion / 3:43 ¶ 28, At the end this is very important piece of technology that can reduce… in Group discussion / 3:46 ¶ 28, Those infrastructure and awareness creation in staffs, mothers, and co… in Group discussion / 3:52 ¶ 36, People will enjoy talking to others how health institution just concer… in Group discussion / 3:57 ¶ 42, Improve work environment as per the need of the technology and owning… in Group discussion / 3:58 ¶ 43, Aspiring more technologies and helping mothers, taking trainings, givi… in Group discussion / 3:60 ¶ 46, Experience with technology. Enable them to identify areas where mHealt… in Group discussion / 3:61 ¶ 47, Planning, decision making based on mHealth, and increased service take… in Group discussion / 3:62 ¶ 48, Support related to mHealth, fulfillment of deficiencies of human and o… in Group discussion

3:43 ¶ 28 in Group discussion

Text quotation

Created by Girma Gilano on 3/9/2023

At the end this is very important piece of technology that can reduce mortality of mothers and infant

2 Codes:

- **Benefits of mHealth: Help_professional**

Comment: by Girma Gilano

> *3/12/2023 11:46:46 AM, merged with Effectiviness 3/12/2023 1:12:09 PM, merged with Decision_making 3/12/2023 1:12:55 PM, merged with Role_improvement*

30 Quotations:

1:24 ¶ 34, We always give best service to our best capacity, but mHealth may adva… in interview analysis / 1:34 ¶ 39, As HEWs we cannot reach for every mother on time but text can easily r… in interview analysis / 1:36 ¶ 40, This mHealth better than our previous our service in interview analysis / 1:50 ¶ 57, further improve the already improving service in interview analysis / 1:56 ¶ 62, Our current ANC and other formats now request mother to register their… in interview analysis / 1:68 ¶ 70, Every system is getting digitalized so digitalizing health may have mu… in interview analysis / 1:82 ¶ 81, ers usually take what we tell them, it will be successful. I don't thi… in interview analysis / 2:3 ¶ 11, It is helpful to also send information which are not appropriate in pe… in KII / 2:7 ¶ 17, Yeah, it will have effective change on community in KII / 2:9 ¶ 19, Yeah, previously mothers use the information they get during ANC, but… in KII / 2:35 ¶ 54, Since we have many gaps this should be seen as an opportunity in KII / 3:2 ¶ 3, Previous teaching of mother through home to home is going to be throug… in Group discussion / 3:12 ¶ 8, For educated mothers messages will be option less in Group discussion / 3:13 ¶ 8, Starting from me we feed additional food at 2months because the kind o… in Group discussion / 3:18 ¶ 12, It looks like mothers are tired of current service provision so the mH… in Group discussion / 3:25 ¶ 14, There may be emerged women who are successful because of mHealth and t… in Group discussion / 3:29 ¶ 17, As a health professionals applying technology can only easy our burden… in Group discussion / 3:34 ¶ 19, ecause of mHealth we may every address of women and now we can also ca… in Group discussion / 3:35 ¶ 20, We will provide the service for mothers we know now, but previously we… in Group discussion / 3:38 ¶ 22, Of course having organized maternal data can also promote contacting a… in Group discussion / 3:41 ¶ 26, : It is a big opportunity to meet the patient again virtually especial… in Group discussion / 3:42 ¶ 26, Traditionally, when errors happen or if the patients go with the wrong… in Group discussion / 3:43 ¶ 28, At the end this is very important piece of technology that can reduce… in Group discussion / 3:46 ¶ 28, Those infrastructure and awareness creation in staffs, mothers, and co… in Group discussion / 3:52 ¶ 36, People will enjoy talking to others how health

institution just concer… in Group discussion / 3:57 ¶ 42, Improve work environment as per the need of the technology and owning… in Group discussion / 3:58 ¶ 43, Aspiring more technologies and helping mothers, taking trainings, givi… in Group discussion / 3:60 ¶ 46, Experience with technology. Enable them to identify areas where mHealt… in Group discussion / 3:61 ¶ 47, Planning, decision making based on mHealth, and increased service take… in Group discussion / 3:62 ¶ 48, Support related to mHealth, fulfillment of deficiencies of human and o… in Group discussion

- **Benefits of mHealth: Improve_MCH**

Comment: by Girma Gilano

> *3/12/2023 11:46:46 AM, merged with Effectiviness 3/12/2023 1:12:09 PM, merged with Decision_making 3/12/2023 1:12:55 PM, merged with Role_improvement*

25 Quotations:

1:6 ¶ 22, Non-use of the service because of negligence and forgetting can be im… in interview analysis / 1:26 ¶ 35, t can increase utilization better than that we have previously in interview analysis / 1:28 ¶ 36, Even since this digital it can further improve the service and increas… in interview analysis / 1:36 ¶ 40, This mHealth better than our previous our service in interview analysis / 1:43 ¶ 50, For maternal and child health the decision to take service is mothers… in interview analysis / 1:49 ¶ 56, Having information on time can improve the health of mothers and child… in interview analysis / 1:50 ¶ 57, further improve the already improving service in interview analysis / 1:57 ¶ 63, f it continue, it can be effective and be important than previous way… in interview analysis / 1:59 ¶ 64, I think whatever we put in to the community to improve health service… in interview analysis / 1:62 ¶ 66, This can improve and put our usual service one step forward in interview analysis / 1:74 ¶ 75, I don't think people can be affected negatively because they love to l… in interview analysis / 1:77 ¶ 77, Rural mothers are respectful; they do what they told to do, so fear of… in interview analysis / 1:78 ¶ 78, One thing I assure is it will increase up taking maternal and child he… in interview analysis / 1:83 ¶ 83, It can remind which is especially important for family planning. Witho… in interview analysis / 2:4 ¶ 12, If health system organized this way it will be helpful in KII / 2:6 ¶ 16, this is the main thing to improve maternal and child care especially u… in KII / 3:8 ¶ 6, After counseling and aware mother it can be improved in Group discussion / 3:9 ¶ 8, It can improve those listed service (ANC, PNC, Breastfeeding and vacci… in Group discussion / 3:10 ¶ 8, Mother are already exposed to health education, but this one is direct… in Group discussion / 3:18 ¶ 12, It looks like mothers are tired of current service provision so the mH… in Group discussion / 3:19 ¶ 12, So in that case it can completely change the stream and interest. Or t… in Group discussion / 3:20 ¶ 12, They may even consider the increased risk of danger related to pregnan… in Group discussion / 3:43 ¶ 28, At the end this is very important piece of technology that can reduce… in Group discussion / 3:48 ¶ 31, Service at their home and Information and on time vaccination and Heal… in Group discussion / 3:49 ¶ 32, Healthy child feeding and behavioral change for mother that wll be lon… in Group discussion

3:46 ¶ 28 in Group discussion

Text quotation

Created by Girma Gilano on 3/9/2023

Those infrastructure and awareness creation in staffs, mothers, and community will safe everything

1 Codes:

- **Benefits of mHealth: Help_professional**

Comment: by Girma Gilano

> *3/12/2023 11:46:46 AM, merged with Effectiviness 3/12/2023 1:12:09 PM, merged with Decision_making 3/12/2023 1:12:55 PM, merged with Role_improvement*

30 Quotations:

1:24 ¶ 34, We always give best service to our best capacity, but mHealth may adva… in interview analysis / 1:34 ¶ 39, As HEWs we cannot reach for every mother on time but text can easily r… in interview analysis / 1:36 ¶ 40, This mHealth better than our previous our service in interview analysis / 1:50 ¶ 57, further improve the already improving service in interview analysis / 1:56 ¶ 62, Our current ANC and other formats now request mother to register their… in interview analysis / 1:68 ¶ 70, Every system is getting digitalized so digitalizing health may have mu… in interview analysis / 1:82 ¶ 81, ers usually take what we tell them, it will be successful. I don't thi… in interview analysis / 2:3 ¶ 11, It is helpful to also send information which are not appropriate in pe… in KII / 2:7 ¶ 17, Yeah, it will have effective change on community in KII / 2:9 ¶ 19, Yeah, previously mothers use the information they get during ANC, but… in KII / 2:35 ¶ 54, Since we have many gaps this should be seen as an opportunity in KII / 3:2 ¶ 3, Previous teaching of mother through home to home is going to be throug… in Group discussion / 3:12 ¶ 8, For educated mothers messages will be option less in Group discussion / 3:13 ¶ 8, Starting from me we feed additional food at 2months because the kind o… in Group discussion / 3:18 ¶ 12, It looks like mothers are tired of current service provision so the mH… in Group discussion / 3:25 ¶ 14, There may be emerged women who are successful because of mHealth and t… in Group discussion / 3:29 ¶ 17, As a health professionals applying technology can only easy our burden… in Group discussion / 3:34 ¶ 19, ecause of mHealth we may every address of women and now we can also ca… in Group discussion / 3:35 ¶ 20, We will provide the service for mothers we know now, but previously we… in Group discussion / 3:38 ¶ 22, Of course having organized maternal data can also promote contacting a… in Group discussion / 3:41 ¶ 26, : It is a big opportunity to meet the patient again virtually especial… in Group discussion / 3:42 ¶ 26, Traditionally, when errors happen or if the patients go with the wrong… in Group discussion / 3:43 ¶ 28, At the end this is very important piece of technology that can reduce… in Group discussion / 3:46 ¶ 28, Those infrastructure and awareness creation in staffs, mothers, and co… in Group discussion / 3:52 ¶ 36, People will enjoy talking to others how health institution just concer… in Group discussion / 3:57 ¶ 42, Improve work environment as per the need of the technology and owning… in Group discussion / 3:58 ¶ 43, Aspiring more technologies and helping mothers, taking trainings, givi… in Group discussion / 3:60 ¶ 46, Experience with technology. Enable them to identify areas where mHealt… in Group discussion / 3:61 ¶ 47, Planning, decision making based on mHealth, and increased service take… in Group discussion / 3:62 ¶ 48, Support related to mHealth, fulfillment of deficiencies of human and o… in Group discussion

3:52 ¶ 36 in Group discussion

Text quotation

Created by Girma Gilano on 3/9/2023

People will enjoy talking to others how health institution just concerned for their health, but some which is around previously may be suspected until they get full awareness.

2 Codes:

- **Benefits of mHealth: Help_professional**

Comment: by Girma Gilano

> *3/12/2023 11:46:46 AM, merged with Effectiviness 3/12/2023 1:12:09 PM, merged with Decision_making 3/12/2023 1:12:55 PM, merged with Role_improvement*

30 Quotations:

1:24 ¶ 34, We always give best service to our best capacity, but mHealth may adva… in interview analysis / 1:34 ¶ 39, As HEWs we cannot reach for every mother on time but text can easily r… in interview analysis / 1:36 ¶ 40, This mHealth better than our previous our service in interview analysis / 1:50 ¶ 57, further improve the already improving service in interview analysis / 1:56 ¶ 62, Our current ANC and other formats now request mother to register their… in interview analysis / 1:68 ¶ 70, Every system is getting digitalized so digitalizing health may have mu… in interview analysis / 1:82 ¶ 81, ers

usually take what we tell them, it will be successful. I don’t thi… in interview analysis / 2:3 ¶ 11, It is helpful to also send information which are not appropriate in pe… in KII / 2:7 ¶ 17, Yeah, it will have effective change on community in KII / 2:9 ¶ 19, Yeah, previously mothers use the information they get during ANC, but… in KII / 2:35 ¶ 54, Since we have many gaps this should be seen as an opportunity in KII / 3:2 ¶ 3, Previous teaching of mother through home to home is going to be throug… in Group discussion / 3:12 ¶ 8, For educated mothers messages will be option less in Group discussion / 3:13 ¶ 8, Starting from me we feed additional food at 2months because the kind o… in Group discussion / 3:18 ¶ 12, It looks like mothers are tired of current service provision so the mH… in Group discussion / 3:25 ¶ 14, There may be emerged women who are successful because of mHealth and t… in Group discussion / 3:29 ¶ 17, As a health professionals applying technology can only easy our burden… in Group discussion / 3:34 ¶ 19, ecause of mHealth we may every address of women and now we can also ca… in Group discussion / 3:35 ¶ 20, We will provide the service for mothers we know now, but previously we… in Group discussion / 3:38 ¶ 22, Of course having organized maternal data can also promote contacting a… in Group discussion / 3:41 ¶ 26, : It is a big opportunity to meet the patient again virtually especial… in Group discussion / 3:42 ¶ 26, Traditionally, when errors happen or if the patients go with the wrong… in Group discussion / 3:43 ¶ 28, At the end this is very important piece of technology that can reduce… in Group discussion / 3:46 ¶ 28, Those infrastructure and awareness creation in staffs, mothers, and co… in Group discussion / 3:52 ¶ 36, People will enjoy talking to others how health institution just concer… in Group discussion / 3:57 ¶ 42, Improve work environment as per the need of the technology and owning… in Group discussion / 3:58 ¶ 43, Aspiring more technologies and helping mothers, taking trainings, givi… in Group discussion / 3:60 ¶ 46, Experience with technology. Enable them to identify areas where mHealt… in Group discussion / 3:61 ¶ 47, Planning, decision making based on mHealth, and increased service take… in Group discussion / 3:62 ¶ 48, Support related to mHealth, fulfillment of deficiencies of human and o… in Group discussion

● Benefits of mHealth: Improve_decision

Comment: by Girma Gilano

> *3/12/2023 11:46:46 AM, merged with Effectiviness 3/12/2023 1:12:09 PM, merged with Decision_making 3/12/2023 1:12:55 PM, merged with Role_improvement*

17 Quotations:

1:5 ¶ 20, t will improve mothers’ time wastage for information that they can get… in interview analysis / 1:23 ¶ 33, will be helpful because it will remain in the mother’s hand for a long… in interview analysis / 1:40 ¶ 45, s it can improve their decision making. This service could increase wo… in interview analysis / 1:42 ¶ 49, provide information to make decisions, motivate her to convince her hu… in interview analysis / 1:49 ¶ 56, Having information on time can improve the health of mothers and child… in interview analysis / 1:55 ¶ 60, mHealth can provide evidence to make decision and can improve everythi… in interview analysis / 1:64 ¶ 67, However, having this information in her hand will continuously strike… in interview analysis / 1:70 ¶ 71, Service can be improved through mHealth because atleast having informa… in interview analysis / 2:15 ¶ 27, Mothers should understand initially the important, but on our side I d… in KII / 3:6 ¶ 5, They difference for mHealth is mother always get counseling or health… in Group discussion / 3:10 ¶ 8, Mother are already exposed to health education, but this one is direct… in Group discussion / 3:20 ¶ 12, They may even consider the increased risk of danger related to pregnan… in Group discussion / 3:24 ¶ 14, Over time this can change community perception and they may reebok mot… in Group discussion / 3:37 ¶ 20, It can also introduce labor division because we may know who must serv… in Group discussion / 3:48 ¶ 31, Service at their home and Information and on time vaccination and Heal… in Group discussion / 3:52 ¶ 36, People will enjoy talking to others how health institution just concer… in Group discussion / 3:67 ¶ 53, he respect for pregnancy mothers may think gave many births and not ca… in Group discussion

3:57 ¶ 42 in Group discussion

Text quotation

Created by Girma Gilano on 3/9/2023

Improve work environment as per the need of the technology and owning the technology

1 Codes:

● **Benefits of mHealth: Help_professional**

Comment: by Girma Gilano

> *3/12/2023 11:46:46 AM, merged with Effectiviness 3/12/2023 1:12:09 PM, merged with Decision_making 3/12/2023 1:12:55 PM, merged with Role_improvement*

30 Quotations:

1:24 ¶ 34, We always give best service to our best capacity, but mHealth may adva… in interview analysis / 1:34 ¶ 39, As HEWs we cannot reach for every mother on time but text can easily r… in interview analysis / 1:36 ¶ 40, This mHealth better than our previous our service in interview analysis / 1:50 ¶ 57, further improve the already improving service in interview analysis / 1:56 ¶ 62, Our current ANC and other formats now request mother to register their… in interview analysis / 1:68 ¶ 70, Every system is getting digitalized so digitalizing health may have mu… in interview analysis / 1:82 ¶ 81, ers usually take what we tell them, it will be successful. I don't thi… in interview analysis / 2:3 ¶ 11, It is helpful to also send information which are not appropriate in pe… in KII / 2:7 ¶ 17, Yeah, it will have effective change on community in KII / 2:9 ¶ 19, Yeah, previously mothers use the information they get during ANC, but… in KII / 2:35 ¶ 54, Since we have many gaps this should be seen as an opportunity in KII / 3:2 ¶ 3, Previous teaching of mother through home to home is going to be throug… in Group discussion / 3:12 ¶ 8, For educated mothers messages will be option less in Group discussion / 3:13 ¶ 8, Starting from me we feed additional food at 2months because the kind o… in Group discussion / 3:18 ¶ 12, It looks like mothers are tired of current service provision so the mH… in Group discussion / 3:25 ¶ 14, There may be emerged women who are successful because of mHealth and t… in Group discussion / 3:29 ¶ 17, As a health professionals applying technology can only easy our burden… in Group discussion / 3:34 ¶ 19, ecause of mHealth we may every address of women and now we can also ca… in Group discussion / 3:35 ¶ 20, We will provide the service for mothers we know now, but previously we… in Group discussion / 3:38 ¶ 22, Of course having organized maternal data can also promote contacting a… in Group discussion / 3:41 ¶ 26, : It is a big opportunity to meet the patient again virtually especial… in Group discussion / 3:42 ¶ 26, Traditionally, when errors happen or if the patients go with the wrong… in Group discussion / 3:43 ¶ 28, At the end this is very important piece of technology that can reduce… in Group discussion / 3:46 ¶ 28, Those infrastructure and awareness creation in staffs, mothers, and co… in Group discussion / 3:52 ¶ 36, People will enjoy talking to others how health institution just concer… in Group discussion / 3:57 ¶ 42, Improve work environment as per the need of the technology and owning… in Group discussion / 3:58 ¶ 43, Aspiring more technologies and helping mothers, taking trainings, givi… in Group discussion / 3:60 ¶ 46, Experience with technology. Enable them to identify areas where mHealt… in Group discussion / 3:61 ¶ 47, Planning, decision making based on mHealth, and increased service take… in Group discussion / 3:62 ¶ 48, Support related to mHealth, fulfillment of deficiencies of human and o… in Group discussion

3:58 ¶ 43 in Group discussion

Text quotation

Created by Girma Gilano on 3/9/2023

Aspiring more technologies and helping mothers, taking trainings, giving trainings after getting themselves initially, helping new employee, continuing with the technology application

1 Codes:

● **Benefits of mHealth: Help_professional**

Comment: by Girma Gilano

3/12/2023 11:46:46 AM, merged with Effectiviness 3/12/2023 1:12:09 PM, merged with Decision_making 3/12/2023 1:12:55 PM, merged with Role_improvement

30 Quotations:

1:24 ¶ 34, We always give best service to our best capacity, but mHealth may adva… in interview analysis / 1:34 ¶ 39, As HEWs we cannot reach for every mother on time but text can easily r… in interview analysis / 1:36 ¶ 40, This mHealth better than our previous our service in interview analysis / 1:50 ¶ 57, further improve the already improving service in interview analysis / 1:56 ¶ 62, Our current ANC and other formats now request mother to register their… in interview analysis / 1:68 ¶ 70, Every system is getting digitalized so digitalizing health may have mu… in interview analysis / 1:82 ¶ 81, ers usually take what we tell them, it will be successful. I don't thi… in interview analysis / 2:3 ¶ 11, It is helpful to also send information which are not appropriate in pe… in KII / 2:7 ¶ 17, Yeah, it will have effective change on community in KII / 2:9 ¶ 19, Yeah, previously mothers use the information they get during ANC, but… in KII / 2:35 ¶ 54, Since we have many gaps this should be seen as an opportunity in KII / 3:2 ¶ 3, Previous teaching of mother through home to home is going to be throug… in Group discussion / 3:12 ¶ 8, For educated mothers messages will be option less in Group discussion / 3:13 ¶ 8, Starting from me we feed additional food at 2months because the kind o… in Group discussion / 3:18 ¶ 12, It looks like mothers are tired of current service provision so the mH… in Group discussion / 3:25 ¶ 14, There may be emerged women who are successful because of mHealth and t… in Group discussion / 3:29 ¶ 17, As a health professionals applying technology can only easy our burden… in Group discussion / 3:34 ¶ 19, ecause of mHealth we may every address of women and now we can also ca… in Group discussion / 3:35 ¶ 20, We will provide the service for mothers we know now, but previously we… in Group discussion / 3:38 ¶ 22, Of course having organized maternal data can also promote contacting a… in Group discussion / 3:41 ¶ 26, : It is a big opportunity to meet the patient again virtually especial… in Group discussion / 3:42 ¶ 26, Traditionally, when errors happen or if the patients go with the wrong… in Group discussion / 3:43 ¶ 28, At the end this is very important piece of technology that can reduce… in Group discussion / 3:46 ¶ 28, Those infrastructure and awareness creation in staffs, mothers, and co… in Group discussion / 3:52 ¶ 36, People will enjoy talking to others how health institution just concer… in Group discussion / 3:57 ¶ 42, Improve work environment as per the need of the technology and owning… in Group discussion / 3:58 ¶ 43, Aspiring more technologies and helping mothers, taking trainings, givi… in Group discussion / 3:60 ¶ 46, Experience with technology. Enable them to identify areas where mHealt… in Group discussion / 3:61 ¶ 47, Planning, decision making based on mHealth, and increased service take… in Group discussion / 3:62 ¶ 48, Support related to mHealth, fulfillment of deficiencies of human and o… in Group discussion

3:60 ¶ 46 in Group discussion

Text quotation

Created by Girma Gilano on 3/9/2023

Experience with technology. Enable them to identify areas where mHealth can be applied. Achieving their objectives

1 Codes:

● Benefits of mHealth: Help_professional

Comment: by Girma Gilano

3/12/2023 11:46:46 AM, merged with Effectiviness 3/12/2023 1:12:09 PM, merged with Decision_making 3/12/2023 1:12:55 PM, merged with Role_improvement

30 Quotations:

1:24 ¶ 34, We always give best service to our best capacity, but mHealth may adva… in interview analysis / 1:34 ¶ 39, As HEWs we cannot reach for every mother on time but text can easily r… in interview analysis / 1:36 ¶ 40, This mHealth better than our previous our service in interview analysis /

1:50 ¶ 57, further improve the already improving service in interview analysis / 1:56 ¶ 62, Our current ANC and other formats now request mother to register their… in interview analysis / 1:68 ¶ 70, Every system is getting digitalized so digitalizing health may have mu… in interview analysis / 1:82 ¶ 81, ers usually take what we tell them, it will be successful. I don't thi… in interview analysis / 2:3 ¶ 11, It is helpful to also send information which are not appropriate in pe… in KII / 2:7 ¶ 17, Yeah, it will have effective change on community in KII / 2:9 ¶ 19, Yeah, previously mothers use the information they get during ANC, but… in KII / 2:35 ¶ 54, Since we have many gaps this should be seen as an opportunity in KII / 3:2 ¶ 3, Previous teaching of mother through home to home is going to be throug… in Group discussion / 3:12 ¶ 8, For educated mothers messages will be option less in Group discussion / 3:13 ¶ 8, Starting from me we feed additional food at 2months because the kind o… in Group discussion / 3:18 ¶ 12, It looks like mothers are tired of current service provision so the mH… in Group discussion / 3:25 ¶ 14, There may be emerged women who are successful because of mHealth and t… in Group discussion / 3:29 ¶ 17, As a health professionals applying technology can only easy our burden… in Group discussion / 3:34 ¶ 19, ecause of mHealth we may every address of women and now we can also ca… in Group discussion / 3:35 ¶ 20, We will provide the service for mothers we know now, but previously we… in Group discussion / 3:38 ¶ 22, Of course having organized maternal data can also promote contacting a… in Group discussion / 3:41 ¶ 26, : It is a big opportunity to meet the patient again virtually especial… in Group discussion / 3:42 ¶ 26, Traditionally, when errors happen or if the patients go with the wrong… in Group discussion / 3:43 ¶ 28, At the end this is very important piece of technology that can reduce… in Group discussion / 3:46 ¶ 28, Those infrastructure and awareness creation in staffs, mothers, and co… in Group discussion / 3:52 ¶ 36, People will enjoy talking to others how health institution just concer… in Group discussion / 3:57 ¶ 42, Improve work environment as per the need of the technology and owning… in Group discussion / 3:58 ¶ 43, Aspiring more technologies and helping mothers, taking trainings, givi… in Group discussion / 3:60 ¶ 46, Experience with technology. Enable them to identify areas where mHealt… in Group discussion / 3:61 ¶ 47, Planning, decision making based on mHealth, and increased service take… in Group discussion / 3:62 ¶ 48, Support related to mHealth, fulfillment of deficiencies of human and o… in Group discussion

3:61 ¶ 47 in Group discussion

Text quotation

Created by Girma Gilano on 3/9/2023

Planning, decision making based on mHealth, and increased service takers

2 Codes:

- **Benefits of mHealth: Help_mothers**

Comment: by Girma Gilano

> *3/12/2023 11:46:46 AM, merged with Effectiviness 3/12/2023 1:12:09 PM, merged with Decision_making 3/12/2023 1:12:55 PM, merged with Role_improvement*

27 Quotations:

1:41 ¶ 47, ion comes after understanding of benefits and harms in interview analysis / 1:45 ¶ 52, Accessibility can be improved mothers get information at their homes in interview analysis / 1:51 ¶ 58, She can access any information in interview analysis / 1:53 ¶ 59, I think mHealth can improve access, appointment on time, availability… in interview analysis / 1:65 ¶ 67, Her husband or children will also push her to go even if the condition… in interview analysis / 1:68 ¶ 70, Every system is getting digitalized so digitalizing health may have mu… in interview analysis / 1:77 ¶ 77, Rural mothers are respectful; they do what they told to do, so fear of… in interview analysis / 1:81 ¶ 80, pe mothers will turn to use mobiles, but only after getting its import… in interview analysis / 2:3 ¶ 11, It is helpful to also send information which are not appropriate in pe… in KII / 2:7 ¶ 17, Yeah, it will have effective change on community in KII / 2:9 ¶ 19, Yeah, previously mothers use the information they get during ANC, but… in KII / 2:11 ¶ 21, Information access, no transportation cost, and no professional resour… in KII / 2:36 ¶ 55, No need of motor or vehicles because it is mHealth. I will be happy to… in KII / 3:6 ¶ 5, They difference for mHealth

is mother always get counseling or health… in Group discussion / 3:18 ¶ 12, It looks like mothers are tired of current service provision so the mH… in Group discussion / 3:23 ¶ 14, Previously, people think poor health system and there are many complia… in Group discussion / 3:24 ¶ 14, Over time this can change community perception and they may reebok mot… in Group discussion / 3:25 ¶ 14, There may be emerged women who are successful because of mHealth and t… in Group discussion / 3:27 ¶ 16, It can make the child health growth, for mothers to know danger signs,… in Group discussion / 3:38 ¶ 22, Of course having organized maternal data can also promote contacting a… in Group discussion / 3:41 ¶ 26, : It is a big opportunity to meet the patient again virtually especial… in Group discussion / 3:42 ¶ 26, Traditionally, when errors happen or if the patients go with the wrong… in Group discussion / 3:50 ¶ 33, Benefit for mothers for next pregnancies and healthy prospect for chil… in Group discussion / 3:59 ¶ 45, Satisfaction of their client service and familiarization with technolo… in Group discussion / 3:61 ¶ 47, Planning, decision making based on mHealth, and increased service take… in Group discussion / 3:62 ¶ 48, Support related to mHealth, fulfillment of deficiencies of human and o… in Group discussion / 3:67 ¶ 53, he respect for pregnancy mothers may think gave many births and not ca… in Group discussion

● Benefits of mHealth: Help_professional

Comment: by Girma Gilano

> *3/12/2023 11:46:46 AM, merged with Effectiviness 3/12/2023 1:12:09 PM, merged with Decision_making 3/12/2023 1:12:55 PM, merged with Role_improvement*

30 Quotations:

1:24 ¶ 34, We always give best service to our best capacity, but mHealth may adva… in interview analysis / 1:34 ¶ 39, As HEWs we cannot reach for every mother on time but text can easily r… in interview analysis / 1:36 ¶ 40, This mHealth better than our previous our service in interview analysis / 1:50 ¶ 57, further improve the already improving service in interview analysis / 1:56 ¶ 62, Our current ANC and other formats now request mother to register their… in interview analysis / 1:68 ¶ 70, Every system is getting digitalized so digitalizing health may have mu… in interview analysis / 1:82 ¶ 81, ers usually take what we tell them, it will be successful. I don't thi… in interview analysis / 2:3 ¶ 11, It is helpful to also send information which are not appropriate in pe… in KII / 2:7 ¶ 17, Yeah, it will have effective change on community in KII / 2:9 ¶ 19, Yeah, previously mothers use the information they get during ANC, but… in KII / 2:35 ¶ 54, Since we have many gaps this should be seen as an opportunity in KII / 3:2 ¶ 3, Previous teaching of mother through home to home is going to be throug… in Group discussion / 3:12 ¶ 8, For educated mothers messages will be option less in Group discussion / 3:13 ¶ 8, Starting from me we feed additional food at 2months because the kind o… in Group discussion / 3:18 ¶ 12, It looks like mothers are tired of current service provision so the mH… in Group discussion / 3:25 ¶ 14, There may be emerged women who are successful because of mHealth and t… in Group discussion / 3:29 ¶ 17, As a health professionals applying technology can only easy our burden… in Group discussion / 3:34 ¶ 19, ecause of mHealth we may every address of women and now we can also ca… in Group discussion / 3:35 ¶ 20, We will provide the service for mothers we know now, but previously we… in Group discussion / 3:38 ¶ 22, Of course having organized maternal data can also promote contacting a… in Group discussion / 3:41 ¶ 26, : It is a big opportunity to meet the patient again virtually especial… in Group discussion / 3:42 ¶ 26, Traditionally, when errors happen or if the patients go with the wrong… in Group discussion / 3:43 ¶ 28, At the end this is very important piece of technology that can reduce… in Group discussion / 3:46 ¶ 28, Those infrastructure and awareness creation in staffs, mothers, and co… in Group discussion / 3:52 ¶ 36, People will enjoy talking to others how health institution just concer… in Group discussion / 3:57 ¶ 42, Improve work environment as per the need of the technology and owning… in Group discussion / 3:58 ¶ 43, Aspiring more technologies and helping mothers, taking trainings, givi… in Group discussion / 3:60 ¶ 46, Experience with technology. Enable them to identify areas where mHealt… in Group discussion / 3:61 ¶ 47, Planning, decision making based on mHealth, and increased service take… in Group discussion / 3:62 ¶ 48, Support related to mHealth, fulfillment of deficiencies of human and o… in Group discussion

3:62 ¶ 48 in Group discussion

Text quotation

Created by Girma Gilano on 3/9/2023

Support related to mHealth, fulfillment of deficiencies of human and other resource limitations, fulfilling the maternal and child health vision, and respect from the community. Acceptance from the community

2 Codes:

Benefits of mHealth: Help_mothers

Comment: by Girma Gilano

> *3/12/2023 11:46:46 AM, merged with Effectiviness 3/12/2023 1:12:09 PM, merged with Decision_making 3/12/2023 1:12:55 PM, merged with Role_improvement*

27 Quotations:

1:41 ¶ 47, ion comes after understanding of benefits and harms in interview analysis / 1:45 ¶ 52, Accessibility can be improved mothers get information at their homes in interview analysis / 1:51 ¶ 58, She can access any information in interview analysis / 1:53 ¶ 59, I think mHealth can improve access, appointment on time, availability… in interview analysis / 1:65 ¶ 67, Her husband or children will also push her to go even if the condition… in interview analysis / 1:68 ¶ 70, Every system is getting digitalized so digitalizing health may have mu… in interview analysis / 1:77 ¶ 77, Rural mothers are respectful; they do what they told to do, so fear of… in interview analysis / 1:81 ¶ 80, pe mothers will turn to use mobiles, but only after getting its import… in interview analysis / 2:3 ¶ 11, It is helpful to also send information which are not appropriate in pe… in KII / 2:7 ¶ 17, Yeah, it will have effective change on community in KII / 2:9 ¶ 19, Yeah, previously mothers use the information they get during ANC, but… in KII / 2:11 ¶ 21, Information access, no transportation cost, and no professional resour… in KII / 2:36 ¶ 55, No need of motor or vehicles because it is mHealth. I will be happy to… in KII / 3:6 ¶ 5, They difference for mHealth is mother always get counseling or health… in Group discussion / 3:18 ¶ 12, It looks like mothers are tired of current service provision so the mH… in Group discussion / 3:23 ¶ 14, Previously, people think poor health system and there are many complia… in Group discussion / 3:24 ¶ 14, Over time this can change community perception and they may reebok mot… in Group discussion / 3:25 ¶ 14, There may be emerged women who are successful because of mHealth and t… in Group discussion / 3:27 ¶ 16, It can make the child health growth, for mothers to know danger signs,… in Group discussion / 3:38 ¶ 22, Of course having organized maternal data can also promote contacting a… in Group discussion / 3:41 ¶ 26, : It is a big opportunity to meet the patient again virtually especial… in Group discussion / 3:42 ¶ 26, Traditionally, when errors happen or if the patients go with the wrong… in Group discussion / 3:50 ¶ 33, Benefit for mothers for next pregnancies and healthy prospect for chil… in Group discussion / 3:59 ¶ 45, Satisfaction of their client service and familiarization with technolo… in Group discussion / 3:61 ¶ 47, Planning, decision making based on mHealth, and increased service take… in Group discussion / 3:62 ¶ 48, Support related to mHealth, fulfillment of deficiencies of human and o… in Group discussion / 3:67 ¶ 53, he respect for pregnancy mothers may think gave many births and not ca… in Group discussion

Benefits of mHealth: Help_professional

Comment: by Girma Gilano

> *3/12/2023 11:46:46 AM, merged with Effectiviness 3/12/2023 1:12:09 PM, merged with Decision_making 3/12/2023 1:12:55 PM, merged with Role_improvement*

30 Quotations:

1:24 ¶ 34, We always give best service to our best capacity, but mHealth may adva… in interview analysis / 1:34 ¶ 39, As HEWs we cannot reach for every mother on time but text can easily r… in interview analysis / 1:36 ¶ 40, This mHealth better than our previous our service in interview analysis / 1:50 ¶ 57, further improve the already improving service in interview analysis / 1:56 ¶ 62, Our current ANC and other formats now request mother to register their… in interview analysis / 1:68 ¶ 70, Every system is getting digitalized so digitalizing health may have mu… in interview analysis / 1:82 ¶ 81, ers usually take what we tell them, it will be successful. I don't thi… in interview analysis / 2:3 ¶ 11, It is helpful to also send information which are not appropriate in pe… in KII / 2:7 ¶ 17, Yeah, it will have effective change on community in KII / 2:9 ¶ 19, Yeah, previously mothers use the information they get

during ANC, but… in KII / 2:35 ¶ 54, Since we have many gaps this should be seen as an opportunity in KII / 3:2 ¶ 3, Previous teaching of mother through home to home is going to be throug… in Group discussion / 3:12 ¶ 8, For educated mothers messages will be option less in Group discussion / 3:13 ¶ 8, Starting from me we feed additional food at 2months because the kind o… in Group discussion / 3:18 ¶ 12, It looks like mothers are tired of current service provision so the mH… in Group discussion / 3:25 ¶ 14, There may be emerged women who are successful because of mHealth and t… in Group discussion / 3:29 ¶ 17, As a health professionals applying technology can only easy our burden… in Group discussion / 3:34 ¶ 19, ecause of mHealth we may every address of women and now we can also ca… in Group discussion / 3:35 ¶ 20, We will provide the service for mothers we know now, but previously we… in Group discussion / 3:38 ¶ 22, Of course having organized maternal data can also promote contacting a… in Group discussion / 3:41 ¶ 26, : It is a big opportunity to meet the patient again virtually especial… in Group discussion / 3:42 ¶ 26, Traditionally, when errors happen or if the patients go with the wrong… in Group discussion / 3:43 ¶ 28, At the end this is very important piece of technology that can reduce… in Group discussion / 3:46 ¶ 28, Those infrastructure and awareness creation in staffs, mothers, and co… in Group discussion / 3:52 ¶ 36, People will enjoy talking to others how health institution just concer… in Group discussion / 3:57 ¶ 42, Improve work environment as per the need of the technology and owning… in Group discussion / 3:58 ¶ 43, Aspiring more technologies and helping mothers, taking trainings, givi… in Group discussion / 3:60 ¶ 46, Experience with technology. Enable them to identify areas where mHealt… in Group discussion / 3:61 ¶ 47, Planning, decision making based on mHealth, and increased service take… in Group discussion / 3:62 ¶ 48, Support related to mHealth, fulfillment of deficiencies of human and o… in Group discussion

---

● Benefits of mHealth: Improve_decision

Created by Girma Gilano on 3/13/2023

Comment: by Girma Gilano

> *3/12/2023 11:46:46 AM, merged with Effectiviness 3/12/2023 1:12:09 PM, merged with Decision_making 3/12/2023 1:12:55 PM, merged with Role_improvement*

17 Quotations:

1:5 ¶ 20 in interview analysis

Text quotation

Created by Girma Gilano on 3/8/2023

t will improve mothers’ time wastage for information that they can get through mHealth

1 Codes:

● Benefits of mHealth: Improve_decision

Comment: by Girma Gilano

> *3/12/2023 11:46:46 AM, merged with Effectiviness 3/12/2023 1:12:09 PM, merged with Decision_making 3/12/2023 1:12:55 PM, merged with Role_improvement*

17 Quotations:

1:5 ¶ 20, t will improve mothers’ time wastage for information that they can get… in interview analysis / 1:23 ¶ 33, will be helpful because it will remain in the mother’s hand for a long… in interview analysis /

1:40 ¶ 45, s it can improve their decision making. This service could increase wo… in interview analysis / 1:42 ¶ 49, provide information to make decisions, motivate her to convince her hu… in interview analysis / 1:49 ¶ 56, Having information on time can improve the health of mothers and child… in interview analysis / 1:55 ¶ 60, mHealth can provide evidence to make decision and can improve everythi… in interview analysis / 1:64 ¶ 67, However, having this information in her hand will continuously strike… in interview analysis / 1:70 ¶ 71, Service can be improved through mHealth because atleast having informa… in interview analysis / 2:15 ¶ 27, Mothers should understand initially the important, but on our side I d… in KII / 3:6 ¶ 5, They difference for mHealth is mother always get counseling or health… in Group discussion / 3:10 ¶ 8, Mother are already exposed to health education, but this one is direct… in Group discussion / 3:20 ¶ 12, They may even consider the increased risk of danger related to pregnan… in Group discussion / 3:24 ¶ 14, Over time this can change community perception and they may reebok mot… in Group discussion / 3:37 ¶ 20, It can also introduce labor division because we may know who must serv… in Group discussion / 3:48 ¶ 31, Service at their home and Information and on time vaccination and Heal… in Group discussion / 3:52 ¶ 36, People will enjoy talking to others how health institution just concer… in Group discussion / 3:67 ¶ 53, he respect for pregnancy mothers may think gave many births and not ca… in Group discussion

1:23 ¶ 33 in interview analysis

Text quotation

Created by Girma Gilano on 3/8/2023

will be helpful because it will remain in the mother’s hand for a long time and she can read at a time

1 Codes:

- **Benefits of mHealth: Improve_decision**

Comment: by Girma Gilano

> *3/12/2023 11:46:46 AM, merged with Effectiviness 3/12/2023 1:12:09 PM, merged with Decision_making 3/12/2023 1:12:55 PM, merged with Role_improvement*

17 Quotations:

1:5 ¶ 20, t will improve mothers’ time wastage for information that they can get… in interview analysis / 1:23 ¶ 33, will be helpful because it will remain in the mother’s hand for a long… in interview analysis / 1:40 ¶ 45, s it can improve their decision making. This service could increase wo… in interview analysis / 1:42 ¶ 49, provide information to make decisions, motivate her to convince her hu… in interview analysis / 1:49 ¶ 56, Having information on time can improve the health of mothers and child… in interview analysis / 1:55 ¶ 60, mHealth can provide evidence to make decision and can improve everythi… in interview analysis / 1:64 ¶ 67, However, having this information in her hand will continuously strike… in interview analysis / 1:70 ¶ 71, Service can be improved through mHealth because atleast having informa… in interview analysis / 2:15 ¶ 27, Mothers should understand initially the important, but on our side I d… in KII / 3:6 ¶ 5, They difference for mHealth is mother always get counseling or health… in Group discussion / 3:10 ¶ 8, Mother are already exposed to health education, but this one is direct… in Group discussion / 3:20 ¶ 12, They may even consider the increased risk of danger related to pregnan… in Group discussion / 3:24 ¶ 14, Over time this can change community perception and they may reebok mot… in Group discussion / 3:37 ¶ 20, It can also introduce labor division because we may know who must serv… in Group discussion / 3:48 ¶ 31, Service at their home and Information and on time vaccination and Heal… in Group discussion / 3:52 ¶ 36, People will enjoy talking to others how health institution just concer… in Group discussion / 3:67 ¶ 53, he respect for pregnancy mothers may think gave many births and not ca… in Group discussion

1:40 ¶ 45 in interview analysis

Text quotation

Created by Girma Gilano on 3/8/2023

s it can improve their decision making. This service could increase women self-decision especially, on ANC, PNC, feeding practice, evidence can helps her

1 Codes:

- **Benefits of mHealth: Improve_decision**

Comment: by Girma Gilano

> *3/12/2023 11:46:46 AM, merged with Effectiviness 3/12/2023 1:12:09 PM, merged with Decision_making 3/12/2023 1:12:55 PM, merged with Role_improvement*

17 Quotations:

1:5 ¶ 20, t will improve mothers' time wastage for information that they can get… in interview analysis / 1:23 ¶ 33, will be helpful because it will remain in the mother's hand for a long… in interview analysis / 1:40 ¶ 45, s it can improve their decision making. This service could increase wo… in interview analysis / 1:42 ¶ 49, provide information to make decisions, motivate her to convince her hu… in interview analysis / 1:49 ¶ 56, Having information on time can improve the health of mothers and child… in interview analysis / 1:55 ¶ 60, mHealth can provide evidence to make decision and can improve everythi… in interview analysis / 1:64 ¶ 67, However, having this information in her hand will continuously strike… in interview analysis / 1:70 ¶ 71, Service can be improved through mHealth because atleast having informa… in interview analysis / 2:15 ¶ 27, Mothers should understand initially the important, but on our side I d… in KII / 3:6 ¶ 5, They difference for mHealth is mother always get counseling or health… in Group discussion / 3:10 ¶ 8, Mother are already exposed to health education, but this one is direct… in Group discussion / 3:20 ¶ 12, They may even consider the increased risk of danger related to pregnan… in Group discussion / 3:24 ¶ 14, Over time this can change community perception and they may reebok mot… in Group discussion / 3:37 ¶ 20, It can also introduce labor division because we may know who must serv… in Group discussion / 3:48 ¶ 31, Service at their home and Information and on time vaccination and Heal… in Group discussion / 3:52 ¶ 36, People will enjoy talking to others how health institution just concer… in Group discussion / 3:67 ¶ 53, he respect for pregnancy mothers may think gave many births and not ca… in Group discussion

1:42 ¶ 49 in interview analysis

Text quotation

Created by Girma Gilano on 3/8/2023

provide information to make decisions, motivate her to convince her husband.

1 Codes:

- **Benefits of mHealth: Improve_decision**

Comment: by Girma Gilano

> *3/12/2023 11:46:46 AM, merged with Effectiviness 3/12/2023 1:12:09 PM, merged with Decision_making 3/12/2023 1:12:55 PM, merged with Role_improvement*

17 Quotations:

1:5 ¶ 20, t will improve mothers' time wastage for information that they can get… in interview analysis / 1:23 ¶ 33, will be helpful because it will remain in the mother's hand for a long… in interview analysis / 1:40 ¶ 45, s it can improve their decision making. This service could increase wo… in interview analysis / 1:42 ¶ 49, provide information to make decisions, motivate her to convince her hu… in interview analysis

/ 1:49 ¶ 56, Having information on time can improve the health of mothers and child… in interview analysis / 1:55 ¶ 60, mHealth can provide evidence to make decision and can improve everythi… in interview analysis / 1:64 ¶ 67, However, having this information in her hand will continuously strike… in interview analysis / 1:70 ¶ 71, Service can be improved through mHealth because atleast having informa… in interview analysis / 2:15 ¶ 27, Mothers should understand initially the important, but on our side I d… in KII / 3:6 ¶ 5, They difference for mHealth is mother always get counseling or health… in Group discussion / 3:10 ¶ 8, Mother are already exposed to health education, but this one is direct… in Group discussion / 3:20 ¶ 12, They may even consider the increased risk of danger related to pregnan… in Group discussion / 3:24 ¶ 14, Over time this can change community perception and they may reebok mot… in Group discussion / 3:37 ¶ 20, It can also introduce labor division because we may know who must serv… in Group discussion / 3:48 ¶ 31, Service at their home and Information and on time vaccination and Heal… in Group discussion / 3:52 ¶ 36, People will enjoy talking to others how health institution just concer… in Group discussion / 3:67 ¶ 53, he respect for pregnancy mothers may think gave many births and not ca… in Group discussion

1:49 ¶ 56 in interview analysis

Text quotation

Created by Girma Gilano on 3/8/2023

Having information on time can improve the health of mothers and children

2 Codes:

- **Benefits of mHealth: Improve_decision**

Comment: by Girma Gilano

> *3/12/2023 11:46:46 AM, merged with Effectiviness 3/12/2023 1:12:09 PM, merged with Decision_making 3/12/2023 1:12:55 PM, merged with Role_improvement*

17 Quotations:

1:5 ¶ 20, t will improve mothers' time wastage for information that they can get… in interview analysis / 1:23 ¶ 33, will be helpful because it will remain in the mother's hand for a long… in interview analysis / 1:40 ¶ 45, s it can improve their decision making. This service could increase wo… in interview analysis / 1:42 ¶ 49, provide information to make decisions, motivate her to convince her hu… in interview analysis / 1:49 ¶ 56, Having information on time can improve the health of mothers and child… in interview analysis / 1:55 ¶ 60, mHealth can provide evidence to make decision and can improve everythi… in interview analysis / 1:64 ¶ 67, However, having this information in her hand will continuously strike… in interview analysis / 1:70 ¶ 71, Service can be improved through mHealth because atleast having informa… in interview analysis / 2:15 ¶ 27, Mothers should understand initially the important, but on our side I d… in KII / 3:6 ¶ 5, They difference for mHealth is mother always get counseling or health… in Group discussion / 3:10 ¶ 8, Mother are already exposed to health education, but this one is direct… in Group discussion / 3:20 ¶ 12, They may even consider the increased risk of danger related to pregnan… in Group discussion / 3:24 ¶ 14, Over time this can change community perception and they may reebok mot… in Group discussion / 3:37 ¶ 20, It can also introduce labor division because we may know who must serv… in Group discussion / 3:48 ¶ 31, Service at their home and Information and on time vaccination and Heal… in Group discussion / 3:52 ¶ 36, People will enjoy talking to others how health institution just concer… in Group discussion / 3:67 ¶ 53, he respect for pregnancy mothers may think gave many births and not ca… in Group discussion

- **Benefits of mHealth: Improve_MCH**

Comment: by Girma Gilano

> *3/12/2023 11:46:46 AM, merged with Effectiviness 3/12/2023 1:12:09 PM, merged with Decision_making 3/12/2023 1:12:55 PM, merged with Role_improvement*

25 Quotations:

1:6 ¶ 22, Non-use of the service because of negligence and forgetting can be im… in interview analysis / 1:26 ¶ 35, t can increase utilization better than that we have previously in interview analysis / 1:28 ¶ 36, Even since this digital it can further improve the service and increas… in interview analysis / 1:36 ¶ 40, This mHealth better than our previous our service in interview analysis / 1:43 ¶ 50, For maternal and child health the decision to take service is mothers… in interview analysis / 1:49 ¶ 56, Having information on time can improve the health of mothers and child… in interview analysis / 1:50 ¶ 57, further improve the already improving service in interview analysis / 1:57 ¶ 63, f it continue, it can be effective and be important than previous way… in interview analysis / 1:59 ¶ 64, I think whatever we put in to the community to improve health service… in interview analysis / 1:62 ¶ 66, This can improve and put our usual service one step forward in interview analysis / 1:74 ¶ 75, I don't think people can be affected negatively because they love to l… in interview analysis / 1:77 ¶ 77, Rural mothers are respectful; they do what they told to do, so fear of… in interview analysis / 1:78 ¶ 78, One thing I assure is it will increase up taking maternal and child he… in interview analysis / 1:83 ¶ 83, It can remind which is especially important for family planning. Witho… in interview analysis / 2:4 ¶ 12, If health system organized this way it will be helpful in KII / 2:6 ¶ 16, this is the main thing to improve maternal and child care especially u… in KII / 3:8 ¶ 6, After counseling and aware mother it can be improved in Group discussion / 3:9 ¶ 8, It can improve those listed service (ANC, PNC, Breastfeeding and vacci… in Group discussion / 3:10 ¶ 8, Mother are already exposed to health education, but this one is direct… in Group discussion / 3:18 ¶ 12, It looks like mothers are tired of current service provision so the mH… in Group discussion / 3:19 ¶ 12, So in that case it can completely change the stream and interest. Or t… in Group discussion / 3:20 ¶ 12, They may even consider the increased risk of danger related to pregnan… in Group discussion / 3:43 ¶ 28, At the end this is very important piece of technology that can reduce… in Group discussion / 3:48 ¶ 31, Service at their home and Information and on time vaccination and Heal… in Group discussion / 3:49 ¶ 32, Healthy child feeding and behavioral change for mother that wll be lon… in Group discussion

1:55 ¶ 60 in interview analysis

Text quotation

Created by Girma Gilano on 3/8/2023

mHealth can provide evidence to make decision and can improve everything mothers need to take the service

1 Codes:

- **Benefits of mHealth: Improve_decision**

Comment: by Girma Gilano

> *3/12/2023 11:46:46 AM, merged with Effectiviness 3/12/2023 1:12:09 PM, merged with Decision_making 3/12/2023 1:12:55 PM, merged with Role_improvement*

17 Quotations:

1:5 ¶ 20, t will improve mothers' time wastage for information that they can get… in interview analysis / 1:23 ¶ 33, will be helpful because it will remain in the mother's hand for a long… in interview analysis / 1:40 ¶ 45, s it can improve their decision making. This service could increase wo… in interview analysis / 1:42 ¶ 49, provide information to make decisions, motivate her to convince her hu… in interview analysis / 1:49 ¶ 56, Having information on time can improve the health of mothers and child… in interview analysis / 1:55 ¶ 60, mHealth can provide evidence to make decision and can improve everythi… in interview analysis / 1:64 ¶ 67, However, having this information in her hand will continuously strike… in interview analysis / 1:70 ¶ 71, Service can be improved through mHealth because atleast having informa… in interview analysis / 2:15 ¶ 27, Mothers should understand initially the important, but on our side I d… in KII / 3:6 ¶ 5, They difference for mHealth is mother always get counseling or health… in Group discussion / 3:10 ¶ 8, Mother are already exposed to health education, but this one is direct… in Group discussion / 3:20 ¶ 12, They may even consider the increased risk of danger related to pregnan…

in Group discussion / 3:24 ¶ 14, Over time this can change community perception and they may reebok mot… in Group discussion / 3:37 ¶ 20, It can also introduce labor division because we may know who must serv… in Group discussion / 3:48 ¶ 31, Service at their home and Information and on time vaccination and Heal… in Group discussion / 3:52 ¶ 36, People will enjoy talking to others how health institution just concer… in Group discussion / 3:67 ¶ 53, he respect for pregnancy mothers may think gave many births and not ca… in Group discussion

1:64 ¶ 67 in interview analysis

Text quotation

Created by Girma Gilano on 3/8/2023

However, having this information in her hand will continuously strike her to go to the appointmen

1 Codes:

- **Benefits of mHealth: Improve_decision**

Comment: by Girma Gilano

> *3/12/2023 11:46:46 AM, merged with Effectiviness 3/12/2023 1:12:09 PM, merged with Decision_making 3/12/2023 1:12:55 PM, merged with Role_improvement*

17 Quotations:

1:5 ¶ 20, t will improve mothers' time wastage for information that they can get… in interview analysis / 1:23 ¶ 33, will be helpful because it will remain in the mother's hand for a long… in interview analysis / 1:40 ¶ 45, s it can improve their decision making. This service could increase wo… in interview analysis / 1:42 ¶ 49, provide information to make decisions, motivate her to convince her hu… in interview analysis / 1:49 ¶ 56, Having information on time can improve the health of mothers and child… in interview analysis / 1:55 ¶ 60, mHealth can provide evidence to make decision and can improve everythi… in interview analysis / 1:64 ¶ 67, However, having this information in her hand will continuously strike… in interview analysis / 1:70 ¶ 71, Service can be improved through mHealth because atleast having informa… in interview analysis / 2:15 ¶ 27, Mothers should understand initially the important, but on our side I d… in KII / 3:6 ¶ 5, They difference for mHealth is mother always get counseling or health… in Group discussion / 3:10 ¶ 8, Mother are already exposed to health education, but this one is direct… in Group discussion / 3:20 ¶ 12, They may even consider the increased risk of danger related to pregnan… in Group discussion / 3:24 ¶ 14, Over time this can change community perception and they may reebok mot… in Group discussion / 3:37 ¶ 20, It can also introduce labor division because we may know who must serv… in Group discussion / 3:48 ¶ 31, Service at their home and Information and on time vaccination and Heal… in Group discussion / 3:52 ¶ 36, People will enjoy talking to others how health institution just concer… in Group discussion / 3:67 ¶ 53, he respect for pregnancy mothers may think gave many births and not ca… in Group discussion

1:70 ¶ 71 in interview analysis

Text quotation

Created by Girma Gilano on 3/8/2023

Service can be improved through mHealth because atleast having information is better than nothing

1 Codes:

- **Benefits of mHealth: Improve_decision**

Comment: by Girma Gilano

> *3/12/2023 11:46:46 AM, merged with Effectiviness 3/12/2023 1:12:09 PM, merged with Decision_making 3/12/2023 1:12:55 PM, merged with Role_improvement*

17 Quotations:

1:5 ¶ 20, t will improve mothers' time wastage for information that they can get… in interview analysis / 1:23 ¶ 33, will be helpful because it will remain in the mother's hand for a long… in interview analysis / 1:40 ¶ 45, s it can improve their decision making. This service could increase wo… in interview analysis / 1:42 ¶ 49, provide information to make decisions, motivate her to convince her hu… in interview analysis / 1:49 ¶ 56, Having information on time can improve the health of mothers and child… in interview analysis / 1:55 ¶ 60, mHealth can provide evidence to make decision and can improve everythi… in interview analysis / 1:64 ¶ 67, However, having this information in her hand will continuously strike… in interview analysis / 1:70 ¶ 71, Service can be improved through mHealth because atleast having informa… in interview analysis / 2:15 ¶ 27, Mothers should understand initially the important, but on our side I d… in KII / 3:6 ¶ 5, They difference for mHealth is mother always get counseling or health… in Group discussion / 3:10 ¶ 8, Mother are already exposed to health education, but this one is direct… in Group discussion / 3:20 ¶ 12, They may even consider the increased risk of danger related to pregnan… in Group discussion / 3:24 ¶ 14, Over time this can change community perception and they may reebok mot… in Group discussion / 3:37 ¶ 20, It can also introduce labor division because we may know who must serv… in Group discussion / 3:48 ¶ 31, Service at their home and Information and on time vaccination and Heal… in Group discussion / 3:52 ¶ 36, People will enjoy talking to others how health institution just concer… in Group discussion / 3:67 ¶ 53, he respect for pregnancy mothers may think gave many births and not ca… in Group discussion

2:15 ¶ 27 in KII

Text quotation

Created by Girma Gilano on 3/9/2023

Mothers should understand initially the important, but on our side I don't think there will be any problem

1 Codes:

- **Benefits of mHealth: Improve_decision**

Comment: by Girma Gilano

> *3/12/2023 11:46:46 AM, merged with Effectiviness 3/12/2023 1:12:09 PM, merged with Decision_making 3/12/2023 1:12:55 PM, merged with Role_improvement*

17 Quotations:

1:5 ¶ 20, t will improve mothers' time wastage for information that they can get… in interview analysis / 1:23 ¶ 33, will be helpful because it will remain in the mother's hand for a long… in interview analysis / 1:40 ¶ 45, s it can improve their decision making. This service could increase wo… in interview analysis / 1:42 ¶ 49, provide information to make decisions, motivate her to convince her hu… in interview analysis / 1:49 ¶ 56, Having information on time can improve the health of mothers and child… in interview analysis / 1:55 ¶ 60, mHealth can provide evidence to make decision and can improve everythi… in interview analysis / 1:64 ¶ 67, However, having this information in her hand will continuously strike… in interview analysis / 1:70 ¶ 71, Service can be improved through mHealth because atleast having informa… in interview analysis / 2:15 ¶ 27, Mothers should understand initially the important, but on our side I d… in KII / 3:6 ¶ 5, They difference for mHealth is mother always get counseling or health… in Group discussion / 3:10 ¶ 8, Mother are already exposed to health education, but this one is direct… in

Group discussion / 3:20 ¶ 12, They may even consider the increased risk of danger related to pregnan… in Group discussion / 3:24 ¶ 14, Over time this can change community perception and they may reebok mot… in Group discussion / 3:37 ¶ 20, It can also introduce labor division because we may know who must serv… in Group discussion / 3:48 ¶ 31, Service at their home and Information and on time vaccination and Heal… in Group discussion / 3:52 ¶ 36, People will enjoy talking to others how health institution just concer… in Group discussion / 3:67 ¶ 53, he respect for pregnancy mothers may think gave many births and not ca… in Group discussion

3:6 ¶ 5 in Group discussion

Text quotation

Created by Girma Gilano on 3/9/2023

They difference for mHealth is mother always get counseling or health education unlike that of visits when large queue limits they counseling and professionals do finishing work. I think mHealth can be benefited if the current behavior can be improved

2 Codes:

- **Benefits of mHealth: Help_mothers**

Comment: by Girma Gilano

> *3/12/2023 11:46:46 AM, merged with Effectiviness 3/12/2023 1:12:09 PM, merged with Decision_making 3/12/2023 1:12:55 PM, merged with Role_improvement*

27 Quotations:

1:41 ¶ 47, ion comes after understanding of benefits and harms in interview analysis / 1:45 ¶ 52, Accessibility can be improved mothers get information at their homes in interview analysis / 1:51 ¶ 58, She can access any information in interview analysis / 1:53 ¶ 59, I think mHealth can improve access, appointment on time, availability… in interview analysis / 1:65 ¶ 67, Her husband or children will also push her to go even if the condition… in interview analysis / 1:68 ¶ 70, Every system is getting digitalized so digitalizing health may have mu… in interview analysis / 1:77 ¶ 77, Rural mothers are respectful; they do what they told to do, so fear of… in interview analysis / 1:81 ¶ 80, pe mothers will turn to use mobiles, but only after getting its import… in interview analysis / 2:3 ¶ 11, It is helpful to also send information which are not appropriate in pe… in KII / 2:7 ¶ 17, Yeah, it will have effective change on community in KII / 2:9 ¶ 19, Yeah, previously mothers use the information they get during ANC, but… in KII / 2:11 ¶ 21, Information access, no transportation cost, and no professional resour… in KII / 2:36 ¶ 55, No need of motor or vehicles because it is mHealth. I will be happy to… in KII / 3:6 ¶ 5, They difference for mHealth is mother always get counseling or health… in Group discussion / 3:18 ¶ 12, It looks like mothers are tired of current service provision so the mH… in Group discussion / 3:23 ¶ 14, Previously, people think poor health system and there are many complia… in Group discussion / 3:24 ¶ 14, Over time this can change community perception and they may reebok mot… in Group discussion / 3:25 ¶ 14, There may be emerged women who are successful because of mHealth and t… in Group discussion / 3:27 ¶ 16, It can make the child health growth, for mothers to know danger signs,… in Group discussion / 3:38 ¶ 22, Of course having organized maternal data can also promote contacting a… in Group discussion / 3:41 ¶ 26, : It is a big opportunity to meet the patient again virtually especial… in Group discussion / 3:42 ¶ 26, Traditionally, when errors happen or if the patients go with the wrong… in Group discussion / 3:50 ¶ 33, Benefit for mothers for next pregnancies and healthy prospect for chil… in Group discussion / 3:59 ¶ 45, Satisfaction of their client service and familiarization with technolo… in Group discussion / 3:61 ¶ 47, Planning, decision making based on mHealth, and increased service take… in Group discussion / 3:62 ¶ 48, Support related to mHealth, fulfillment of deficiencies of human and o… in Group discussion / 3:67 ¶ 53, he respect for pregnancy mothers may think gave many births and not ca… in Group discussion

- **Benefits of mHealth: Improve_decision**

Comment: by Girma Gilano

3/12/2023 11:46:46 AM, merged with Effectiviness 3/12/2023 1:12:09 PM, merged with Decision_making 3/12/2023 1:12:55 PM, merged with Role_improvement

17 Quotations:

1:5 ¶ 20, t will improve mothers' time wastage for information that they can get… in interview analysis / 1:23 ¶ 33, will be helpful because it will remain in the mother's hand for a long… in interview analysis / 1:40 ¶ 45, s it can improve their decision making. This service could increase wo… in interview analysis / 1:42 ¶ 49, provide information to make decisions, motivate her to convince her hu… in interview analysis / 1:49 ¶ 56, Having information on time can improve the health of mothers and child… in interview analysis / 1:55 ¶ 60, mHealth can provide evidence to make decision and can improve everythi… in interview analysis / 1:64 ¶ 67, However, having this information in her hand will continuously strike… in interview analysis / 1:70 ¶ 71, Service can be improved through mHealth because atleast having informa… in interview analysis / 2:15 ¶ 27, Mothers should understand initially the important, but on our side I d… in KII / 3:6 ¶ 5, They difference for mHealth is mother always get counseling or health… in Group discussion / 3:10 ¶ 8, Mother are already exposed to health education, but this one is direct… in Group discussion / 3:20 ¶ 12, They may even consider the increased risk of danger related to pregnan… in Group discussion / 3:24 ¶ 14, Over time this can change community perception and they may reebok mot… in Group discussion / 3:37 ¶ 20, It can also introduce labor division because we may know who must serv… in Group discussion / 3:48 ¶ 31, Service at their home and Information and on time vaccination and Heal… in Group discussion / 3:52 ¶ 36, People will enjoy talking to others how health institution just concer… in Group discussion / 3:67 ¶ 53, he respect for pregnancy mothers may think gave many births and not ca… in Group discussion

3:10 ¶ 8 in Group discussion

Text quotation

Created by Girma Gilano on 3/9/2023

Mother are already exposed to health education, but this one is directly motivates mothers at their home. I think it will improve maternal and child health if we use it appropriately

2 Codes:

- **Benefits of mHealth: Improve_decision**

Comment: by Girma Gilano

3/12/2023 11:46:46 AM, merged with Effectiviness 3/12/2023 1:12:09 PM, merged with Decision_making 3/12/2023 1:12:55 PM, merged with Role_improvement

17 Quotations:

1:5 ¶ 20, t will improve mothers' time wastage for information that they can get… in interview analysis / 1:23 ¶ 33, will be helpful because it will remain in the mother's hand for a long… in interview analysis / 1:40 ¶ 45, s it can improve their decision making. This service could increase wo… in interview analysis / 1:42 ¶ 49, provide information to make decisions, motivate her to convince her hu… in interview analysis / 1:49 ¶ 56, Having information on time can improve the health of mothers and child… in interview analysis / 1:55 ¶ 60, mHealth can provide evidence to make decision and can improve everythi… in interview analysis / 1:64 ¶ 67, However, having this information in her hand will continuously strike… in interview analysis / 1:70 ¶ 71, Service can be improved through mHealth because atleast having informa… in interview analysis / 2:15 ¶ 27, Mothers should understand initially the important, but on our side I d… in KII / 3:6 ¶ 5, They difference for mHealth is mother always get counseling or health… in Group discussion / 3:10 ¶ 8, Mother are already exposed to health education, but this one is direct… in Group discussion / 3:20 ¶ 12, They may even consider the increased risk of danger related to pregnan… in Group discussion / 3:24 ¶ 14, Over time this can change community perception and they may reebok mot… in Group discussion / 3:37 ¶ 20, It can also introduce labor division because we may know who

must serv… in Group discussion / 3:48 ¶ 31, Service at their home and Information and on time vaccination and Heal… in Group discussion / 3:52 ¶ 36, People will enjoy talking to others how health institution just concer… in Group discussion / 3:67 ¶ 53, he respect for pregnancy mothers may think gave many births and not ca… in Group discussion

- **Benefits of mHealth: Improve_MCH**

Comment: by Girma Gilano

> *3/12/2023 11:46:46 AM, merged with Effectiviness 3/12/2023 1:12:09 PM, merged with Decision_making 3/12/2023 1:12:55 PM, merged with Role_improvement*

25 Quotations:

1:6 ¶ 22, Non-use of the service because of negligence and forgetting can be im… in interview analysis / 1:26 ¶ 35, t can increase utilization better than that we have previously in interview analysis / 1:28 ¶ 36, Even since this digital it can further improve the service and increas… in interview analysis / 1:36 ¶ 40, This mHealth better than our previous our service in interview analysis / 1:43 ¶ 50, For maternal and child health the decision to take service is mothers… in interview analysis / 1:49 ¶ 56, Having information on time can improve the health of mothers and child… in interview analysis / 1:50 ¶ 57, further improve the already improving service in interview analysis / 1:57 ¶ 63, f it continue, it can be effective and be important than previous way… in interview analysis / 1:59 ¶ 64, I think whatever we put in to the community to improve health service… in interview analysis / 1:62 ¶ 66, This can improve and put our usual service one step forward in interview analysis / 1:74 ¶ 75, I don't think people can be affected negatively because they love to l… in interview analysis / 1:77 ¶ 77, Rural mothers are respectful; they do what they told to do, so fear of… in interview analysis / 1:78 ¶ 78, One thing I assure is it will increase up taking maternal and child he… in interview analysis / 1:83 ¶ 83, It can remind which is especially important for family planning. Witho… in interview analysis / 2:4 ¶ 12, If health system organized this way it will be helpful in KII / 2:6 ¶ 16, this is the main thing to improve maternal and child care especially u… in KII / 3:8 ¶ 6, After counseling and aware mother it can be improved in Group discussion / 3:9 ¶ 8, It can improve those listed service (ANC, PNC, Breastfeeding and vacci… in Group discussion / 3:10 ¶ 8, Mother are already exposed to health education, but this one is direct… in Group discussion / 3:18 ¶ 12, It looks like mothers are tired of current service provision so the mH… in Group discussion / 3:19 ¶ 12, So in that case it can completely change the stream and interest. Or t… in Group discussion / 3:20 ¶ 12, They may even consider the increased risk of danger related to pregnan… in Group discussion / 3:43 ¶ 28, At the end this is very important piece of technology that can reduce… in Group discussion / 3:48 ¶ 31, Service at their home and Information and on time vaccination and Heal… in Group discussion / 3:49 ¶ 32, Healthy child feeding and behavioral change for mother that wll be lon… in Group discussion

3:20 ¶ 12 in Group discussion

Text quotation

Created by Girma Gilano on 3/9/2023

They may even consider the increased risk of danger related to pregnancy, child feeding, and vaccination. May be the emergence of new diseases can enforce them to vaccinate their children

2 Codes:

- **Benefits of mHealth: Improve_decision**

Comment: by Girma Gilano

> *3/12/2023 11:46:46 AM, merged with Effectiviness 3/12/2023 1:12:09 PM, merged with Decision_making 3/12/2023 1:12:55 PM, merged with Role_improvement*

17 Quotations:

1:5 ¶ 20, t will improve mothers' time wastage for information that they can get… in interview analysis / 1:23 ¶ 33, will be helpful because it will remain in the mother's hand for a long… in interview analysis / 1:40 ¶ 45, s it can improve their decision making. This service could increase wo… in interview analysis / 1:42 ¶ 49, provide information to make decisions, motivate her to convince her hu… in interview analysis / 1:49 ¶ 56, Having information on time can improve the health of mothers and child… in interview analysis / 1:55 ¶ 60, mHealth can provide evidence to make decision and can improve everythi… in interview analysis / 1:64 ¶ 67, However, having this information in her hand will continuously strike… in interview analysis / 1:70 ¶ 71, Service can be improved through mHealth because atleast having informa… in interview analysis / 2:15 ¶ 27, Mothers should understand initially the important, but on our side I d… in KII / 3:6 ¶ 5, They difference for mHealth is mother always get counseling or health… in Group discussion / 3:10 ¶ 8, Mother are already exposed to health education, but this one is direct… in Group discussion / 3:20 ¶ 12, They may even consider the increased risk of danger related to pregnan… in Group discussion / 3:24 ¶ 14, Over time this can change community perception and they may reebok mot… in Group discussion / 3:37 ¶ 20, It can also introduce labor division because we may know who must serv… in Group discussion / 3:48 ¶ 31, Service at their home and Information and on time vaccination and Heal… in Group discussion / 3:52 ¶ 36, People will enjoy talking to others how health institution just concer… in Group discussion / 3:67 ¶ 53, he respect for pregnancy mothers may think gave many births and not ca… in Group discussion

● Benefits of mHealth: Improve_MCH

Comment: by Girma Gilano

> *3/12/2023 11:46:46 AM, merged with Effectiviness 3/12/2023 1:12:09 PM, merged with Decision_making 3/12/2023 1:12:55 PM, merged with Role_improvement*

25 Quotations:

1:6 ¶ 22, Non-use of the service because of negligence and forgetting can be im… in interview analysis / 1:26 ¶ 35, t can increase utilization better than that we have previously in interview analysis / 1:28 ¶ 36, Even since this digital it can further improve the service and increas… in interview analysis / 1:36 ¶ 40, This mHealth better than our previous our service in interview analysis / 1:43 ¶ 50, For maternal and child health the decision to take service is mothers… in interview analysis / 1:49 ¶ 56, Having information on time can improve the health of mothers and child… in interview analysis / 1:50 ¶ 57, further improve the already improving service in interview analysis / 1:57 ¶ 63, f it continue, it can be effective and be important than previous way… in interview analysis / 1:59 ¶ 64, I think whatever we put in to the community to improve health service… in interview analysis / 1:62 ¶ 66, This can improve and put our usual service one step forward in interview analysis / 1:74 ¶ 75, I don't think people can be affected negatively because they love to l… in interview analysis / 1:77 ¶ 77, Rural mothers are respectful; they do what they told to do, so fear of… in interview analysis / 1:78 ¶ 78, One thing I assure is it will increase up taking maternal and child he… in interview analysis / 1:83 ¶ 83, It can remind which is especially important for family planning. Witho… in interview analysis / 2:4 ¶ 12, If health system organized this way it will be helpful in KII / 2:6 ¶ 16, this is the main thing to improve maternal and child care especially u… in KII / 3:8 ¶ 6, After counseling and aware mother it can be improved in Group discussion / 3:9 ¶ 8, It can improve those listed service (ANC, PNC, Breastfeeding and vacci… in Group discussion / 3:10 ¶ 8, Mother are already exposed to health education, but this one is direct… in Group discussion / 3:18 ¶ 12, It looks like mothers are tired of current service provision so the mH… in Group discussion / 3:19 ¶ 12, So in that case it can completely change the stream and interest. Or t… in Group discussion / 3:20 ¶ 12, They may even consider the increased risk of danger related to pregnan… in Group discussion / 3:43 ¶ 28, At the end this is very important piece of technology that can reduce… in Group discussion / 3:48 ¶ 31, Service at their home and Information and on time vaccination and Heal… in Group discussion / 3:49 ¶ 32, Healthy child feeding and behavioral change for mother that wll be lon… in Group discussion

3:24 ¶ 14 in Group discussion

Text quotation

Created by Girma Gilano on 3/9/2023

Over time this can change community perception and they may reebok mothers who faced the health problem because of poor follow up and poor usage of messages because they already knew that they are been under intensive attention follow up

2 Codes:

● Benefits of mHealth: Help_mothers

Comment: by Girma Gilano

> *3/12/2023 11:46:46 AM, merged with Effectiviness 3/12/2023 1:12:09 PM, merged with Decision_making 3/12/2023 1:12:55 PM, merged with Role_improvement*

27 Quotations:

1:41 ¶ 47, ion comes after understanding of benefits and harms in interview analysis / 1:45 ¶ 52, Accessibility can be improved mothers get information at their homes in interview analysis / 1:51 ¶ 58, She can access any information in interview analysis / 1:53 ¶ 59, I think mHealth can improve access, appointment on time, availability… in interview analysis / 1:65 ¶ 67, Her husband or children will also push her to go even if the condition… in interview analysis / 1:68 ¶ 70, Every system is getting digitalized so digitalizing health may have mu… in interview analysis / 1:77 ¶ 77, Rural mothers are respectful; they do what they told to do, so fear of… in interview analysis / 1:81 ¶ 80, pe mothers will turn to use mobiles, but only after getting its import… in interview analysis / 2:3 ¶ 11, It is helpful to also send information which are not appropriate in pe… in KII / 2:7 ¶ 17, Yeah, it will have effective change on community in KII / 2:9 ¶ 19, Yeah, previously mothers use the information they get during ANC, but… in KII / 2:11 ¶ 21, Information access, no transportation cost, and no professional resour… in KII / 2:36 ¶ 55, No need of motor or vehicles because it is mHealth. I will be happy to… in KII / 3:6 ¶ 5, They difference for mHealth is mother always get counseling or health… in Group discussion / 3:18 ¶ 12, It looks like mothers are tired of current service provision so the mH… in Group discussion / 3:23 ¶ 14, Previously, people think poor health system and there are many complia… in Group discussion / 3:24 ¶ 14, Over time this can change community perception and they may reebok mot… in Group discussion / 3:25 ¶ 14, There may be emerged women who are successful because of mHealth and t… in Group discussion / 3:27 ¶ 16, It can make the child health growth, for mothers to know danger signs,… in Group discussion / 3:38 ¶ 22, Of course having organized maternal data can also promote contacting a… in Group discussion / 3:41 ¶ 26, : It is a big opportunity to meet the patient again virtually especial… in Group discussion / 3:42 ¶ 26, Traditionally, when errors happen or if the patients go with the wrong… in Group discussion / 3:50 ¶ 33, Benefit for mothers for next pregnancies and healthy prospect for chil… in Group discussion / 3:59 ¶ 45, Satisfaction of their client service and familiarization with technolo… in Group discussion / 3:61 ¶ 47, Planning, decision making based on mHealth, and increased service take… in Group discussion / 3:62 ¶ 48, Support related to mHealth, fulfillment of deficiencies of human and o… in Group discussion / 3:67 ¶ 53, he respect for pregnancy mothers may think gave many births and not ca… in Group discussion

● Benefits of mHealth: Improve_decision

Comment: by Girma Gilano

> *3/12/2023 11:46:46 AM, merged with Effectiviness 3/12/2023 1:12:09 PM, merged with Decision_making 3/12/2023 1:12:55 PM, merged with Role_improvement*

17 Quotations:

1:5 ¶ 20, t will improve mothers' time wastage for information that they can get… in interview analysis / 1:23 ¶ 33, will be helpful because it will remain in the mother's hand for a long… in interview analysis / 1:40 ¶ 45, s it can improve their decision making. This service could increase wo… in interview analysis / 1:42 ¶ 49, provide information to make decisions, motivate her to convince her hu… in interview analysis / 1:49 ¶ 56, Having information on time can improve the health of mothers and child… in interview analysis / 1:55 ¶ 60, mHealth can provide evidence to make decision and can improve everythi… in interview analysis / 1:64 ¶ 67, However, having this information in her hand will continuously strike… in interview analysis / 1:70 ¶ 71, Service can be improved through mHealth because atleast having informa… in interview analysis / 2:15 ¶ 27, Mothers should understand initially the important, but on our

side I d… in KII / 3:6 ¶ 5, They difference for mHealth is mother always get counseling or health… in Group discussion / 3:10 ¶ 8, Mother are already exposed to health education, but this one is direct… in Group discussion / 3:20 ¶ 12, They may even consider the increased risk of danger related to pregnan… in Group discussion / 3:24 ¶ 14, Over time this can change community perception and they may reebok mot… in Group discussion / 3:37 ¶ 20, It can also introduce labor division because we may know who must serv… in Group discussion / 3:48 ¶ 31, Service at their home and Information and on time vaccination and Heal… in Group discussion / 3:52 ¶ 36, People will enjoy talking to others how health institution just concer… in Group discussion / 3:67 ¶ 53, he respect for pregnancy mothers may think gave many births and not ca… in Group discussion

3:37 ¶ 20 in Group discussion

Text quotation

Created by Girma Gilano on 3/9/2023

It can also introduce labor division because we may know who must serve whom or how many. Previously we just communicate numbers and percent, and tell people reduced or increased but we do not know why and when the number becomes that.

1 Codes:

- **Benefits of mHealth: Improve_decision**

Comment: by Girma Gilano

> *3/12/2023 11:46:46 AM, merged with Effectiviness 3/12/2023 1:12:09 PM, merged with Decision_making 3/12/2023 1:12:55 PM, merged with Role_improvement*

17 Quotations:

1:5 ¶ 20, t will improve mothers' time wastage for information that they can get… in interview analysis / 1:23 ¶ 33, will be helpful because it will remain in the mother's hand for a long… in interview analysis / 1:40 ¶ 45, s it can improve their decision making. This service could increase wo… in interview analysis / 1:42 ¶ 49, provide information to make decisions, motivate her to convince her hu… in interview analysis / 1:49 ¶ 56, Having information on time can improve the health of mothers and child… in interview analysis / 1:55 ¶ 60, mHealth can provide evidence to make decision and can improve everythi… in interview analysis / 1:64 ¶ 67, However, having this information in her hand will continuously strike… in interview analysis / 1:70 ¶ 71, Service can be improved through mHealth because atleast having informa… in interview analysis / 2:15 ¶ 27, Mothers should understand initially the important, but on our side I d… in KII / 3:6 ¶ 5, They difference for mHealth is mother always get counseling or health… in Group discussion / 3:10 ¶ 8, Mother are already exposed to health education, but this one is direct… in Group discussion / 3:20 ¶ 12, They may even consider the increased risk of danger related to pregnan… in Group discussion / 3:24 ¶ 14, Over time this can change community perception and they may reebok mot… in Group discussion / 3:37 ¶ 20, It can also introduce labor division because we may know who must serv… in Group discussion / 3:48 ¶ 31, Service at their home and Information and on time vaccination and Heal… in Group discussion / 3:52 ¶ 36, People will enjoy talking to others how health institution just concer… in Group discussion / 3:67 ¶ 53, he respect for pregnancy mothers may think gave many births and not ca… in Group discussion

3:48 ¶ 31 in Group discussion

Text quotation

Created by Girma Gilano on 3/9/2023

Service at their home and Information and on time vaccination and Healthy child live

2 Codes:

● Benefits of mHealth: Improve_decision

Comment: by Girma Gilano

> *3/12/2023 11:46:46 AM, merged with Effectiviness 3/12/2023 1:12:09 PM, merged with Decision_making 3/12/2023 1:12:55 PM, merged with Role_improvement*

17 Quotations:

1:5 ¶ 20, t will improve mothers' time wastage for information that they can get… in interview analysis / 1:23 ¶ 33, will be helpful because it will remain in the mother's hand for a long… in interview analysis / 1:40 ¶ 45, s it can improve their decision making. This service could increase wo… in interview analysis / 1:42 ¶ 49, provide information to make decisions, motivate her to convince her hu… in interview analysis / 1:49 ¶ 56, Having information on time can improve the health of mothers and child… in interview analysis / 1:55 ¶ 60, mHealth can provide evidence to make decision and can improve everythi… in interview analysis / 1:64 ¶ 67, However, having this information in her hand will continuously strike… in interview analysis / 1:70 ¶ 71, Service can be improved through mHealth because atleast having informa… in interview analysis / 2:15 ¶ 27, Mothers should understand initially the important, but on our side I d… in KII / 3:6 ¶ 5, They difference for mHealth is mother always get counseling or health… in Group discussion / 3:10 ¶ 8, Mother are already exposed to health education, but this one is direct… in Group discussion / 3:20 ¶ 12, They may even consider the increased risk of danger related to pregnan… in Group discussion / 3:24 ¶ 14, Over time this can change community perception and they may reebok mot… in Group discussion / 3:37 ¶ 20, It can also introduce labor division because we may know who must serv… in Group discussion / 3:48 ¶ 31, Service at their home and Information and on time vaccination and Heal… in Group discussion / 3:52 ¶ 36, People will enjoy talking to others how health institution just concer… in Group discussion / 3:67 ¶ 53, he respect for pregnancy mothers may think gave many births and not ca… in Group discussion

● Benefits of mHealth: Improve_MCH

Comment: by Girma Gilano

> *3/12/2023 11:46:46 AM, merged with Effectiviness 3/12/2023 1:12:09 PM, merged with Decision_making 3/12/2023 1:12:55 PM, merged with Role_improvement*

25 Quotations:

1:6 ¶ 22, Non-use of the service because of negligence and forgetting can be im… in interview analysis / 1:26 ¶ 35, t can increase utilization better than that we have previously in interview analysis / 1:28 ¶ 36, Even since this digital it can further improve the service and increas… in interview analysis / 1:36 ¶ 40, This mHealth better than our previous our service in interview analysis / 1:43 ¶ 50, For maternal and child health the decision to take service is mothers… in interview analysis / 1:49 ¶ 56, Having information on time can improve the health of mothers and child… in interview analysis / 1:50 ¶ 57, further improve the already improving service in interview analysis / 1:57 ¶ 63, f it continue, it can be effective and be important than previous way… in interview analysis / 1:59 ¶ 64, I think whatever we put in to the community to improve health service… in interview analysis / 1:62 ¶ 66, This can improve and put our usual service one step forward in interview analysis / 1:74 ¶ 75, I don't think people can be affected negatively because they love to l… in interview analysis / 1:77 ¶ 77, Rural mothers are respectful; they do what they told to do, so fear of… in interview analysis / 1:78 ¶ 78, One thing I assure is it will increase up taking maternal and child he… in interview analysis / 1:83 ¶ 83, It can remind which is especially important for family planning. Witho… in interview analysis / 2:4 ¶ 12, If health system organized this way it will be helpful in KII / 2:6 ¶ 16, this is the main thing to improve maternal and child care especially u… in KII / 3:8 ¶ 6, After counseling and aware mother it can be improved in Group discussion / 3:9 ¶ 8, It can improve those listed service (ANC, PNC, Breastfeeding and vacci… in Group discussion / 3:10 ¶ 8, Mother are already exposed to health education, but this one is direct… in Group discussion / 3:18 ¶ 12, It looks like mothers are tired of current service provision so the mH… in Group discussion / 3:19 ¶ 12, So in that case it can completely change the stream and interest. Or t… in Group discussion / 3:20 ¶ 12, They may even consider the increased risk of danger related to pregnan… in Group discussion / 3:43 ¶ 28, At the end this is very important piece of technology that can reduce… in Group discussion / 3:48 ¶

31, Service at their home and Information and on time vaccination and Heal… in Group discussion / 3:49 ¶ 32, Healthy child feeding and behavioral change for mother that wll be lon… in Group discussion

3:52 ¶ 36 in Group discussion

Text quotation

Created by Girma Gilano on 3/9/2023

People will enjoy talking to others how health institution just concerned for their health, but some which is around previously may be suspected until they get full awareness.

2 Codes:

- **Benefits of mHealth: Help_professional**

Comment: by Girma Gilano

> *3/12/2023 11:46:46 AM, merged with Effectiviness 3/12/2023 1:12:09 PM, merged with Decision_making 3/12/2023 1:12:55 PM, merged with Role_improvement*

30 Quotations:

1:24 ¶ 34, We always give best service to our best capacity, but mHealth may adva… in interview analysis / 1:34 ¶ 39, As HEWs we cannot reach for every mother on time but text can easily r… in interview analysis / 1:36 ¶ 40, This mHealth better than our previous our service in interview analysis / 1:50 ¶ 57, further improve the already improving service in interview analysis / 1:56 ¶ 62, Our current ANC and other formats now request mother to register their… in interview analysis / 1:68 ¶ 70, Every system is getting digitalized so digitalizing health may have mu… in interview analysis / 1:82 ¶ 81, ers usually take what we tell them, it will be successful. I don't thi… in interview analysis / 2:3 ¶ 11, It is helpful to also send information which are not appropriate in pe… in KII / 2:7 ¶ 17, Yeah, it will have effective change on community in KII / 2:9 ¶ 19, Yeah, previously mothers use the information they get during ANC, but… in KII / 2:35 ¶ 54, Since we have many gaps this should be seen as an opportunity in KII / 3:2 ¶ 3, Previous teaching of mother through home to home is going to be throug… in Group discussion / 3:12 ¶ 8, For educated mothers messages will be option less in Group discussion / 3:13 ¶ 8, Starting from me we feed additional food at 2months because the kind o… in Group discussion / 3:18 ¶ 12, It looks like mothers are tired of current service provision so the mH… in Group discussion / 3:25 ¶ 14, There may be emerged women who are successful because of mHealth and t… in Group discussion / 3:29 ¶ 17, As a health professionals applying technology can only easy our burden… in Group discussion / 3:34 ¶ 19, ecause of mHealth we may every address of women and now we can also ca… in Group discussion / 3:35 ¶ 20, We will provide the service for mothers we know now, but previously we… in Group discussion / 3:38 ¶ 22, Of course having organized maternal data can also promote contacting a… in Group discussion / 3:41 ¶ 26, : It is a big opportunity to meet the patient again virtually especial… in Group discussion / 3:42 ¶ 26, Traditionally, when errors happen or if the patients go with the wrong… in Group discussion / 3:43 ¶ 28, At the end this is very important piece of technology that can reduce… in Group discussion / 3:46 ¶ 28, Those infrastructure and awareness creation in staffs, mothers, and co… in Group discussion / 3:52 ¶ 36, People will enjoy talking to others how health institution just concer… in Group discussion / 3:57 ¶ 42, Improve work environment as per the need of the technology and owning… in Group discussion / 3:58 ¶ 43, Aspiring more technologies and helping mothers, taking trainings, givi… in Group discussion / 3:60 ¶ 46, Experience with technology. Enable them to identify areas where mHealt… in Group discussion / 3:61 ¶ 47, Planning, decision making based on mHealth, and increased service take… in Group discussion / 3:62 ¶ 48, Support related to mHealth, fulfillment of deficiencies of human and o… in Group discussion

- **Benefits of mHealth: Improve_decision**

Comment: by Girma Gilano

3/12/2023 11:46:46 AM, merged with Effectiviness 3/12/2023 1:12:09 PM, merged with Decision_making 3/12/2023 1:12:55 PM, merged with Role_improvement

17 Quotations:

1:5 ¶ 20, t will improve mothers' time wastage for information that they can get… in interview analysis / 1:23 ¶ 33, will be helpful because it will remain in the mother's hand for a long… in interview analysis / 1:40 ¶ 45, s it can improve their decision making. This service could increase wo… in interview analysis / 1:42 ¶ 49, provide information to make decisions, motivate her to convince her hu… in interview analysis / 1:49 ¶ 56, Having information on time can improve the health of mothers and child… in interview analysis / 1:55 ¶ 60, mHealth can provide evidence to make decision and can improve everythi… in interview analysis / 1:64 ¶ 67, However, having this information in her hand will continuously strike… in interview analysis / 1:70 ¶ 71, Service can be improved through mHealth because atleast having informa… in interview analysis / 2:15 ¶ 27, Mothers should understand initially the important, but on our side I d… in KII / 3:6 ¶ 5, They difference for mHealth is mother always get counseling or health… in Group discussion / 3:10 ¶ 8, Mother are already exposed to health education, but this one is direct… in Group discussion / 3:20 ¶ 12, They may even consider the increased risk of danger related to pregnan… in Group discussion / 3:24 ¶ 14, Over time this can change community perception and they may reebok mot… in Group discussion / 3:37 ¶ 20, It can also introduce labor division because we may know who must serv… in Group discussion / 3:48 ¶ 31, Service at their home and Information and on time vaccination and Heal… in Group discussion / 3:52 ¶ 36, People will enjoy talking to others how health institution just concer… in Group discussion / 3:67 ¶ 53, he respect for pregnancy mothers may think gave many births and not ca… in Group discussion

3:67 ¶ 53 in Group discussion

Text quotation

Created by Girma Gilano on 3/9/2023

he respect for pregnancy mothers may think gave many births and not caring seriously. Need of behavioral change for full considerations

2 Codes:

- **Benefits of mHealth: Help_mothers**

Comment: by Girma Gilano

3/12/2023 11:46:46 AM, merged with Effectiviness 3/12/2023 1:12:09 PM, merged with Decision_making 3/12/2023 1:12:55 PM, merged with Role_improvement

27 Quotations:

1:41 ¶ 47, ion comes after understanding of benefits and harms in interview analysis / 1:45 ¶ 52, Accessibility can be improved mothers get information at their homes in interview analysis / 1:51 ¶ 58, She can access any information in interview analysis / 1:53 ¶ 59, I think mHealth can improve access, appointment on time, availability… in interview analysis / 1:65 ¶ 67, Her husband or children will also push her to go even if the condition… in interview analysis / 1:68 ¶ 70, Every system is getting digitalized so digitalizing health may have mu… in interview analysis / 1:77 ¶ 77, Rural mothers are respectful; they do what they told to do, so fear of… in interview analysis / 1:81 ¶ 80, pe mothers will turn to use mobiles, but only after getting its import… in interview analysis / 2:3 ¶ 11, It is helpful to also send information which are not appropriate in pe… in KII / 2:7 ¶ 17, Yeah, it will have effective change on community in KII / 2:9 ¶ 19, Yeah, previously mothers use the information they get during ANC, but… in KII / 2:11 ¶ 21, Information access, no transportation cost, and no professional resour… in KII / 2:36 ¶ 55, No need of motor or vehicles because it is mHealth. I will be happy to… in KII / 3:6 ¶ 5, They difference for mHealth is mother always get counseling or health… in Group discussion / 3:18 ¶ 12, It looks like mothers are tired of current service provision so the mH… in Group discussion / 3:23 ¶ 14, Previously, people think poor health system and there are many complia… in Group discussion / 3:24 ¶ 14, Over time this can

change community perception and they may reebok mot… in Group discussion / 3:25 ¶ 14, There may be emerged women who are successful because of mHealth and t… in Group discussion / 3:27 ¶ 16, It can make the child health growth, for mothers to know danger signs,… in Group discussion / 3:38 ¶ 22, Of course having organized maternal data can also promote contacting a… in Group discussion / 3:41 ¶ 26, : It is a big opportunity to meet the patient again virtually especial… in Group discussion / 3:42 ¶ 26, Traditionally, when errors happen or if the patients go with the wrong… in Group discussion / 3:50 ¶ 33, Benefit for mothers for next pregnancies and healthy prospect for chil… in Group discussion / 3:59 ¶ 45, Satisfaction of their client service and familiarization with technolo… in Group discussion / 3:61 ¶ 47, Planning, decision making based on mHealth, and increased service take… in Group discussion / 3:62 ¶ 48, Support related to mHealth, fulfillment of deficiencies of human and o… in Group discussion / 3:67 ¶ 53, he respect for pregnancy mothers may think gave many births and not ca… in Group discussion

Benefits of mHealth: Improve_decision

Comment: by Girma Gilano

> *3/12/2023 11:46:46 AM, merged with Effectiviness 3/12/2023 1:12:09 PM, merged with Decision_making 3/12/2023 1:12:55 PM, merged with Role_improvement*

17 Quotations:

1:5 ¶ 20, t will improve mothers' time wastage for information that they can get… in interview analysis / 1:23 ¶ 33, will be helpful because it will remain in the mother's hand for a long… in interview analysis / 1:40 ¶ 45, s it can improve their decision making. This service could increase wo… in interview analysis / 1:42 ¶ 49, provide information to make decisions, motivate her to convince her hu… in interview analysis / 1:49 ¶ 56, Having information on time can improve the health of mothers and child… in interview analysis / 1:55 ¶ 60, mHealth can provide evidence to make decision and can improve everythi… in interview analysis / 1:64 ¶ 67, However, having this information in her hand will continuously strike… in interview analysis / 1:70 ¶ 71, Service can be improved through mHealth because atleast having informa… in interview analysis / 2:15 ¶ 27, Mothers should understand initially the important, but on our side I d… in KII / 3:6 ¶ 5, They difference for mHealth is mother always get counseling or health… in Group discussion / 3:10 ¶ 8, Mother are already exposed to health education, but this one is direct… in Group discussion / 3:20 ¶ 12, They may even consider the increased risk of danger related to pregnan… in Group discussion / 3:24 ¶ 14, Over time this can change community perception and they may reebok mot… in Group discussion / 3:37 ¶ 20, It can also introduce labor division because we may know who must serv… in Group discussion / 3:48 ¶ 31, Service at their home and Information and on time vaccination and Heal… in Group discussion / 3:52 ¶ 36, People will enjoy talking to others how health institution just concer… in Group discussion / 3:67 ¶ 53, he respect for pregnancy mothers may think gave many births and not ca… in Group discussion

---

Benefits of mHealth: Improve_MCH

Created by Girma Gilano on 3/13/2023

Comment: by Girma Gilano

> *3/12/2023 11:46:46 AM, merged with Effectiviness 3/12/2023 1:12:09 PM, merged with Decision_making 3/12/2023 1:12:55 PM, merged with Role_improvement*

25 Quotations:

1:6 ¶ 22 in interview analysis

Text quotation

Created by Girma Gilano on 3/8/2023

Non-use of the service because of negligence and forgetting can be improved by frequent messages

1 Codes:

● **Benefits of mHealth: Improve_MCH**

Comment: by Girma Gilano

> *3/12/2023 11:46:46 AM, merged with Effectiviness 3/12/2023 1:12:09 PM, merged with Decision_making 3/12/2023 1:12:55 PM, merged with Role_improvement*

25 Quotations:

1:6 ¶ 22, Non-use of the service because of negligence and forgetting can be im… in interview analysis / 1:26 ¶ 35, t can increase utilization better than that we have previously in interview analysis / 1:28 ¶ 36, Even since this digital it can further improve the service and increas… in interview analysis / 1:36 ¶ 40, This mHealth better than our previous our service in interview analysis / 1:43 ¶ 50, For maternal and child health the decision to take service is mothers… in interview analysis / 1:49 ¶ 56, Having information on time can improve the health of mothers and child… in interview analysis / 1:50 ¶ 57, further improve the already improving service in interview analysis / 1:57 ¶ 63, f it continue, it can be effective and be important than previous way… in interview analysis / 1:59 ¶ 64, I think whatever we put in to the community to improve health service… in interview analysis / 1:62 ¶ 66, This can improve and put our usual service one step forward in interview analysis / 1:74 ¶ 75, I don't think people can be affected negatively because they love to l… in interview analysis / 1:77 ¶ 77, Rural mothers are respectful; they do what they told to do, so fear of… in interview analysis / 1:78 ¶ 78, One thing I assure is it will increase up taking maternal and child he… in interview analysis / 1:83 ¶ 83, It can remind which is especially important for family planning. Witho… in interview analysis / 2:4 ¶ 12, If health system organized this way it will be helpful in KII / 2:6 ¶ 16, this is the main thing to improve maternal and child care especially u… in KII / 3:8 ¶ 6, After counseling and aware mother it can be improved in Group discussion / 3:9 ¶ 8, It can improve those listed service (ANC, PNC, Breastfeeding and vacci… in Group discussion / 3:10 ¶ 8, Mother are already exposed to health education, but this one is direct… in Group discussion / 3:18 ¶ 12, It looks like mothers are tired of current service provision so the mH… in Group discussion / 3:19 ¶ 12, So in that case it can completely change the stream and interest. Or t… in Group discussion / 3:20 ¶ 12, They may even consider the increased risk of danger related to pregnan… in Group discussion / 3:43 ¶ 28, At the end this is very important piece of technology that can reduce… in Group discussion / 3:48 ¶ 31, Service at their home and Information and on time vaccination and Heal… in Group discussion / 3:49 ¶ 32, Healthy child feeding and behavioral change for mother that wll be lon… in Group discussion

1:26 ¶ 35 in interview analysis

Text quotation

Created by Girma Gilano on 3/8/2023

t can increase utilization better than that we have previously

1 Codes:

● **Benefits of mHealth: Improve_MCH**

Comment: by Girma Gilano

> *3/12/2023 11:46:46 AM, merged with Effectiviness 3/12/2023 1:12:09 PM, merged with Decision_making 3/12/2023 1:12:55 PM, merged with Role_improvement*

25 Quotations:

1:6 ¶ 22, Non-use of the service because of negligence and forgetting can be im… in interview analysis / 1:26 ¶ 35, t can increase utilization better than that we have previously in interview analysis / 1:28 ¶ 36, Even since this digital it can further improve the service and increas… in interview analysis / 1:36 ¶ 40, This mHealth better than our previous our service in interview analysis / 1:43 ¶ 50, For maternal and child health the decision to take service is mothers… in interview analysis / 1:49 ¶ 56, Having information on time can improve the health of mothers and child… in interview analysis / 1:50 ¶ 57, further improve the already improving service in interview analysis / 1:57 ¶ 63, f it continue, it can be effective and be important than previous way… in interview analysis / 1:59 ¶ 64, I think whatever we put in to the community to improve health service… in interview analysis / 1:62 ¶ 66, This can improve and put our usual service one step forward in interview analysis / 1:74 ¶ 75, I don't think people can be affected negatively because they love to l… in interview analysis / 1:77 ¶ 77, Rural mothers are respectful; they do what they told to do, so fear of… in interview analysis / 1:78 ¶ 78, One thing I assure is it will increase up taking maternal and child he… in interview analysis / 1:83 ¶ 83, It can remind which is especially important for family planning. Witho… in interview analysis / 2:4 ¶ 12, If health system organized this way it will be helpful in KII / 2:6 ¶ 16, this is the main thing to improve maternal and child care especially u… in KII / 3:8 ¶ 6, After counseling and aware mother it can be improved in Group discussion / 3:9 ¶ 8, It can improve those listed service (ANC, PNC, Breastfeeding and vacci… in Group discussion / 3:10 ¶ 8, Mother are already exposed to health education, but this one is direct… in Group discussion / 3:18 ¶ 12, It looks like mothers are tired of current service provision so the mH… in Group discussion / 3:19 ¶ 12, So in that case it can completely change the stream and interest. Or t… in Group discussion / 3:20 ¶ 12, They may even consider the increased risk of danger related to pregnan… in Group discussion / 3:43 ¶ 28, At the end this is very important piece of technology that can reduce… in Group discussion / 3:48 ¶ 31, Service at their home and Information and on time vaccination and Heal… in Group discussion / 3:49 ¶ 32, Healthy child feeding and behavioral change for mother that wll be lon… in Group discussion

1:28 ¶ 36 in interview analysis

Text quotation

Created by Girma Gilano on 3/8/2023

Even since this digital it can further improve the service and increase interest

1 Codes:

- **Benefits of mHealth: Improve_MCH**

Comment: by Girma Gilano

> *3/12/2023 11:46:46 AM, merged with Effectiviness 3/12/2023 1:12:09 PM, merged with Decision_making 3/12/2023 1:12:55 PM, merged with Role_improvement*

25 Quotations:

1:6 ¶ 22, Non-use of the service because of negligence and forgetting can be im… in interview analysis / 1:26 ¶ 35, t can increase utilization better than that we have previously in interview analysis / 1:28 ¶ 36, Even since this digital it can further improve the service and increas… in interview analysis / 1:36 ¶ 40, This mHealth better than our previous our service in interview analysis / 1:43 ¶ 50, For maternal and child health the decision to take service is mothers… in interview analysis / 1:49 ¶ 56, Having information on time can improve the health of mothers and child… in interview analysis / 1:50 ¶ 57, further improve the already improving service in interview analysis / 1:57 ¶ 63, f it continue, it can be effective and be important than previous way… in interview analysis / 1:59 ¶ 64, I think whatever we put in to the community to improve health service… in interview analysis / 1:62 ¶ 66, This can improve and put our usual service one step forward in interview analysis / 1:74 ¶ 75, I don't think people can be affected negatively because they love to l… in interview analysis / 1:77 ¶ 77, Rural mothers are respectful; they do what they told to do, so fear of… in interview analysis / 1:78 ¶ 78, One thing I assure is it will increase up taking maternal and child he… in interview analysis / 1:83 ¶ 83, It can remind which is especially important for family planning. Witho… in interview analysis / 2:4 ¶ 12, If health system organized this way

it will be helpful in KII / 2:6 ¶ 16, this is the main thing to improve maternal and child care especially u… in KII / 3:8 ¶ 6, After counseling and aware mother it can be improved in Group discussion / 3:9 ¶ 8, It can improve those listed service (ANC, PNC, Breastfeeding and vacci… in Group discussion / 3:10 ¶ 8, Mother are already exposed to health education, but this one is direct… in Group discussion / 3:18 ¶ 12, It looks like mothers are tired of current service provision so the mH… in Group discussion / 3:19 ¶ 12, So in that case it can completely change the stream and interest. Or t… in Group discussion / 3:20 ¶ 12, They may even consider the increased risk of danger related to pregnan… in Group discussion / 3:43 ¶ 28, At the end this is very important piece of technology that can reduce… in Group discussion / 3:48 ¶ 31, Service at their home and Information and on time vaccination and Heal… in Group discussion / 3:49 ¶ 32, Healthy child feeding and behavioral change for mother that wll be lon… in Group discussion

1:36 ¶ 40 in interview analysis

Text quotation

Created by Girma Gilano on 3/8/2023

This mHealth better than our previous our service

2 Codes:

- **Benefits of mHealth: Help_professional**

Comment: by Girma Gilano

> *3/12/2023 11:46:46 AM, merged with Effectiviness 3/12/2023 1:12:09 PM, merged with Decision_making 3/12/2023 1:12:55 PM, merged with Role_improvement*

30 Quotations:

1:24 ¶ 34, We always give best service to our best capacity, but mHealth may adva… in interview analysis / 1:34 ¶ 39, As HEWs we cannot reach for every mother on time but text can easily r… in interview analysis / 1:36 ¶ 40, This mHealth better than our previous our service in interview analysis / 1:50 ¶ 57, further improve the already improving service in interview analysis / 1:56 ¶ 62, Our current ANC and other formats now request mother to register their… in interview analysis / 1:68 ¶ 70, Every system is getting digitalized so digitalizing health may have mu… in interview analysis / 1:82 ¶ 81, ers usually take what we tell them, it will be successful. I don't thi… in interview analysis / 2:3 ¶ 11, It is helpful to also send information which are not appropriate in pe… in KII / 2:7 ¶ 17, Yeah, it will have effective change on community in KII / 2:9 ¶ 19, Yeah, previously mothers use the information they get during ANC, but… in KII / 2:35 ¶ 54, Since we have many gaps this should be seen as an opportunity in KII / 3:2 ¶ 3, Previous teaching of mother through home to home is going to be throug… in Group discussion / 3:12 ¶ 8, For educated mothers messages will be option less in Group discussion / 3:13 ¶ 8, Starting from me we feed additional food at 2months because the kind o… in Group discussion / 3:18 ¶ 12, It looks like mothers are tired of current service provision so the mH… in Group discussion / 3:25 ¶ 14, There may be emerged women who are successful because of mHealth and t… in Group discussion / 3:29 ¶ 17, As a health professionals applying technology can only easy our burden… in Group discussion / 3:34 ¶ 19, ecause of mHealth we may every address of women and now we can also ca… in Group discussion / 3:35 ¶ 20, We will provide the service for mothers we know now, but previously we… in Group discussion / 3:38 ¶ 22, Of course having organized maternal data can also promote contacting a… in Group discussion / 3:41 ¶ 26, : It is a big opportunity to meet the patient again virtually especial… in Group discussion / 3:42 ¶ 26, Traditionally, when errors happen or if the patients go with the wrong… in Group discussion / 3:43 ¶ 28, At the end this is very important piece of technology that can reduce… in Group discussion / 3:46 ¶ 28, Those infrastructure and awareness creation in staffs, mothers, and co… in Group discussion / 3:52 ¶ 36, People will enjoy talking to others how health institution just concer… in Group discussion / 3:57 ¶ 42, Improve work environment as per the need of the technology and owning… in Group discussion / 3:58 ¶ 43, Aspiring more technologies and helping mothers, taking trainings, givi… in Group discussion / 3:60 ¶ 46, Experience with technology. Enable them to identify areas where mHealt… in Group discussion / 3:61 ¶ 47, Planning, decision making based

on mHealth, and increased service take… in Group discussion / 3:62 ¶ 48, Support related to mHealth, fulfillment of deficiencies of human and o… in Group discussion

- **Benefits of mHealth: Improve_MCH**

Comment: by Girma Gilano

> *3/12/2023 11:46:46 AM, merged with Effectiviness 3/12/2023 1:12:09 PM, merged with Decision_making 3/12/2023 1:12:55 PM, merged with Role_improvement*

25 Quotations:

1:6 ¶ 22, Non-use of the service because of negligence and forgetting can be im… in interview analysis / 1:26 ¶ 35, t can increase utilization better than that we have previously in interview analysis / 1:28 ¶ 36, Even since this digital it can further improve the service and increas… in interview analysis / 1:36 ¶ 40, This mHealth better than our previous our service in interview analysis / 1:43 ¶ 50, For maternal and child health the decision to take service is mothers… in interview analysis / 1:49 ¶ 56, Having information on time can improve the health of mothers and child… in interview analysis / 1:50 ¶ 57, further improve the already improving service in interview analysis / 1:57 ¶ 63, f it continue, it can be effective and be important than previous way… in interview analysis / 1:59 ¶ 64, I think whatever we put in to the community to improve health service… in interview analysis / 1:62 ¶ 66, This can improve and put our usual service one step forward in interview analysis / 1:74 ¶ 75, I don't think people can be affected negatively because they love to l… in interview analysis / 1:77 ¶ 77, Rural mothers are respectful; they do what they told to do, so fear of… in interview analysis / 1:78 ¶ 78, One thing I assure is it will increase up taking maternal and child he… in interview analysis / 1:83 ¶ 83, It can remind which is especially important for family planning. Witho… in interview analysis / 2:4 ¶ 12, If health system organized this way it will be helpful in KII / 2:6 ¶ 16, this is the main thing to improve maternal and child care especially u… in KII / 3:8 ¶ 6, After counseling and aware mother it can be improved in Group discussion / 3:9 ¶ 8, It can improve those listed service (ANC, PNC, Breastfeeding and vacci… in Group discussion / 3:10 ¶ 8, Mother are already exposed to health education, but this one is direct… in Group discussion / 3:18 ¶ 12, It looks like mothers are tired of current service provision so the mH… in Group discussion / 3:19 ¶ 12, So in that case it can completely change the stream and interest. Or t… in Group discussion / 3:20 ¶ 12, They may even consider the increased risk of danger related to pregnan… in Group discussion / 3:43 ¶ 28, At the end this is very important piece of technology that can reduce… in Group discussion / 3:48 ¶ 31, Service at their home and Information and on time vaccination and Heal… in Group discussion / 3:49 ¶ 32, Healthy child feeding and behavioral change for mother that wll be lon… in Group discussion

1:43 ¶ 50 in interview analysis

Text quotation

Created by Girma Gilano on 3/8/2023

For maternal and child health the decision to take service is mothers duty

1 Codes:

- **Benefits of mHealth: Improve_MCH**

Comment: by Girma Gilano

> *3/12/2023 11:46:46 AM, merged with Effectiviness 3/12/2023 1:12:09 PM, merged with Decision_making 3/12/2023 1:12:55 PM, merged with Role_improvement*

25 Quotations:

1:6 ¶ 22, Non-use of the service because of negligence and forgetting can be im… in interview analysis / 1:26 ¶ 35, t can increase utilization better than that we have previously in interview analysis / 1:28 ¶ 36, Even since this digital it can further improve the service and increas… in interview analysis / 1:36 ¶ 40,

This mHealth better than our previous our service in interview analysis / 1:43 ¶ 50, For maternal and child health the decision to take service is mothers… in interview analysis / 1:49 ¶ 56, Having information on time can improve the health of mothers and child… in interview analysis / 1:50 ¶ 57, further improve the already improving service in interview analysis / 1:57 ¶ 63, f it continue, it can be effective and be important than previous way… in interview analysis / 1:59 ¶ 64, I think whatever we put in to the community to improve health service… in interview analysis / 1:62 ¶ 66, This can improve and put our usual service one step forward in interview analysis / 1:74 ¶ 75, I don't think people can be affected negatively because they love to l… in interview analysis / 1:77 ¶ 77, Rural mothers are respectful; they do what they told to do, so fear of… in interview analysis / 1:78 ¶ 78, One thing I assure is it will increase up taking maternal and child he… in interview analysis / 1:83 ¶ 83, It can remind which is especially important for family planning. Witho… in interview analysis / 2:4 ¶ 12, If health system organized this way it will be helpful in KII / 2:6 ¶ 16, this is the main thing to improve maternal and child care especially u… in KII / 3:8 ¶ 6, After counseling and aware mother it can be improved in Group discussion / 3:9 ¶ 8, It can improve those listed service (ANC, PNC, Breastfeeding and vacci… in Group discussion / 3:10 ¶ 8, Mother are already exposed to health education, but this one is direct… in Group discussion / 3:18 ¶ 12, It looks like mothers are tired of current service provision so the mH… in Group discussion / 3:19 ¶ 12, So in that case it can completely change the stream and interest. Or t… in Group discussion / 3:20 ¶ 12, They may even consider the increased risk of danger related to pregnan… in Group discussion / 3:43 ¶ 28, At the end this is very important piece of technology that can reduce… in Group discussion / 3:48 ¶ 31, Service at their home and Information and on time vaccination and Heal… in Group discussion / 3:49 ¶ 32, Healthy child feeding and behavioral change for mother that wll be lon… in Group discussion

1:49 ¶ 56 in interview analysis

Text quotation

Created by Girma Gilano on 3/8/2023

Having information on time can improve the health of mothers and children

2 Codes:

- **Benefits of mHealth: Improve_decision**

Comment: by Girma Gilano

> *3/12/2023 11:46:46 AM, merged with Effectiviness 3/12/2023 1:12:09 PM, merged with Decision_making 3/12/2023 1:12:55 PM, merged with Role_improvement*

17 Quotations:

1:5 ¶ 20, t will improve mothers' time wastage for information that they can get… in interview analysis / 1:23 ¶ 33, will be helpful because it will remain in the mother's hand for a long… in interview analysis / 1:40 ¶ 45, s it can improve their decision making. This service could increase wo… in interview analysis / 1:42 ¶ 49, provide information to make decisions, motivate her to convince her hu… in interview analysis / 1:49 ¶ 56, Having information on time can improve the health of mothers and child… in interview analysis / 1:55 ¶ 60, mHealth can provide evidence to make decision and can improve everythi… in interview analysis / 1:64 ¶ 67, However, having this information in her hand will continuously strike… in interview analysis / 1:70 ¶ 71, Service can be improved through mHealth because atleast having informa… in interview analysis / 2:15 ¶ 27, Mothers should understand initially the important, but on our side I d… in KII / 3:6 ¶ 5, They difference for mHealth is mother always get counseling or health… in Group discussion / 3:10 ¶ 8, Mother are already exposed to health education, but this one is direct… in Group discussion / 3:20 ¶ 12, They may even consider the increased risk of danger related to pregnan… in Group discussion / 3:24 ¶ 14, Over time this can change community perception and they may reebok mot… in Group discussion / 3:37 ¶ 20, It can also introduce labor division because we may know who must serv… in Group discussion / 3:48 ¶ 31, Service at their home and Information and on time vaccination and Heal… in Group discussion / 3:52 ¶ 36, People will enjoy talking to others how health institution just concer… in Group discussion / 3:67 ¶ 53, he respect for pregnancy mothers may think gave many births and not ca… in Group discussion

- **Benefits of mHealth: Improve_MCH**

  Comment: by Girma Gilano

  > *3/12/2023 11:46:46 AM, merged with Effectiviness 3/12/2023 1:12:09 PM, merged with Decision_making 3/12/2023 1:12:55 PM, merged with Role_improvement*

  25 Quotations:

  1:6 ¶ 22, Non-use of the service because of negligence and forgetting can be im… in interview analysis / 1:26 ¶ 35, t can increase utilization better than that we have previously in interview analysis / 1:28 ¶ 36, Even since this digital it can further improve the service and increas… in interview analysis / 1:36 ¶ 40, This mHealth better than our previous our service in interview analysis / 1:43 ¶ 50, For maternal and child health the decision to take service is mothers… in interview analysis / 1:49 ¶ 56, Having information on time can improve the health of mothers and child… in interview analysis / 1:50 ¶ 57, further improve the already improving service in interview analysis / 1:57 ¶ 63, f it continue, it can be effective and be important than previous way… in interview analysis / 1:59 ¶ 64, I think whatever we put in to the community to improve health service… in interview analysis / 1:62 ¶ 66, This can improve and put our usual service one step forward in interview analysis / 1:74 ¶ 75, I don't think people can be affected negatively because they love to l… in interview analysis / 1:77 ¶ 77, Rural mothers are respectful; they do what they told to do, so fear of… in interview analysis / 1:78 ¶ 78, One thing I assure is it will increase up taking maternal and child he… in interview analysis / 1:83 ¶ 83, It can remind which is especially important for family planning. Witho… in interview analysis / 2:4 ¶ 12, If health system organized this way it will be helpful in KII / 2:6 ¶ 16, this is the main thing to improve maternal and child care especially u… in KII / 3:8 ¶ 6, After counseling and aware mother it can be improved in Group discussion / 3:9 ¶ 8, It can improve those listed service (ANC, PNC, Breastfeeding and vacci… in Group discussion / 3:10 ¶ 8, Mother are already exposed to health education, but this one is direct… in Group discussion / 3:18 ¶ 12, It looks like mothers are tired of current service provision so the mH… in Group discussion / 3:19 ¶ 12, So in that case it can completely change the stream and interest. Or t… in Group discussion / 3:20 ¶ 12, They may even consider the increased risk of danger related to pregnan… in Group discussion / 3:43 ¶ 28, At the end this is very important piece of technology that can reduce… in Group discussion / 3:48 ¶ 31, Service at their home and Information and on time vaccination and Heal… in Group discussion / 3:49 ¶ 32, Healthy child feeding and behavioral change for mother that wll be lon… in Group discussion

1:50 ¶ 57 in interview analysis

Text quotation

Created by Girma Gilano on 3/8/2023

further improve the already improving service

2 Codes:

- **Benefits of mHealth: Help_professional**

  Comment: by Girma Gilano

  > *3/12/2023 11:46:46 AM, merged with Effectiviness 3/12/2023 1:12:09 PM, merged with Decision_making 3/12/2023 1:12:55 PM, merged with Role_improvement*

  30 Quotations:

  1:24 ¶ 34, We always give best service to our best capacity, but mHealth may adva… in interview analysis / 1:34 ¶ 39, As HEWs we cannot reach for every mother on time but text can easily r… in interview analysis / 1:36 ¶ 40, This mHealth better than our previous our service in interview analysis / 1:50 ¶ 57, further improve the already improving service in interview analysis / 1:56 ¶ 62, Our current ANC and other formats now request mother to register their… in interview analysis / 1:68 ¶ 70, Every system is getting digitalized so digitalizing health may have mu… in interview analysis / 1:82 ¶ 81, ers

usually take what we tell them, it will be successful. I don't thi… in interview analysis / 2:3 ¶ 11, It is helpful to also send information which are not appropriate in pe… in KII / 2:7 ¶ 17, Yeah, it will have effective change on community in KII / 2:9 ¶ 19, Yeah, previously mothers use the information they get during ANC, but… in KII / 2:35 ¶ 54, Since we have many gaps this should be seen as an opportunity in KII / 3:2 ¶ 3, Previous teaching of mother through home to home is going to be throug… in Group discussion / 3:12 ¶ 8, For educated mothers messages will be option less in Group discussion / 3:13 ¶ 8, Starting from me we feed additional food at 2months because the kind o… in Group discussion / 3:18 ¶ 12, It looks like mothers are tired of current service provision so the mH… in Group discussion / 3:25 ¶ 14, There may be emerged women who are successful because of mHealth and t… in Group discussion / 3:29 ¶ 17, As a health professionals applying technology can only easy our burden… in Group discussion / 3:34 ¶ 19, ecause of mHealth we may every address of women and now we can also ca… in Group discussion / 3:35 ¶ 20, We will provide the service for mothers we know now, but previously we… in Group discussion / 3:38 ¶ 22, Of course having organized maternal data can also promote contacting a… in Group discussion / 3:41 ¶ 26, : It is a big opportunity to meet the patient again virtually especial… in Group discussion / 3:42 ¶ 26, Traditionally, when errors happen or if the patients go with the wrong… in Group discussion / 3:43 ¶ 28, At the end this is very important piece of technology that can reduce… in Group discussion / 3:46 ¶ 28, Those infrastructure and awareness creation in staffs, mothers, and co… in Group discussion / 3:52 ¶ 36, People will enjoy talking to others how health institution just concer… in Group discussion / 3:57 ¶ 42, Improve work environment as per the need of the technology and owning… in Group discussion / 3:58 ¶ 43, Aspiring more technologies and helping mothers, taking trainings, givi… in Group discussion / 3:60 ¶ 46, Experience with technology. Enable them to identify areas where mHealt… in Group discussion / 3:61 ¶ 47, Planning, decision making based on mHealth, and increased service take… in Group discussion / 3:62 ¶ 48, Support related to mHealth, fulfillment of deficiencies of human and o… in Group discussion

Benefits of mHealth: Improve_MCH

Comment: by Girma Gilano

> *3/12/2023 11:46:46 AM, merged with Effectiviness 3/12/2023 1:12:09 PM, merged with Decision_making 3/12/2023 1:12:55 PM, merged with Role_improvement*

25 Quotations:

1:6 ¶ 22, Non-use of the service because of negligence and forgetting can be im… in interview analysis / 1:26 ¶ 35, t can increase utilization better than that we have previously in interview analysis / 1:28 ¶ 36, Even since this digital it can further improve the service and increas… in interview analysis / 1:36 ¶ 40, This mHealth better than our previous our service in interview analysis / 1:43 ¶ 50, For maternal and child health the decision to take service is mothers… in interview analysis / 1:49 ¶ 56, Having information on time can improve the health of mothers and child… in interview analysis / 1:50 ¶ 57, further improve the already improving service in interview analysis / 1:57 ¶ 63, f it continue, it can be effective and be important than previous way… in interview analysis / 1:59 ¶ 64, I think whatever we put in to the community to improve health service… in interview analysis / 1:62 ¶ 66, This can improve and put our usual service one step forward in interview analysis / 1:74 ¶ 75, I don't think people can be affected negatively because they love to l… in interview analysis / 1:77 ¶ 77, Rural mothers are respectful; they do what they told to do, so fear of… in interview analysis / 1:78 ¶ 78, One thing I assure is it will increase up taking maternal and child he… in interview analysis / 1:83 ¶ 83, It can remind which is especially important for family planning. Witho… in interview analysis / 2:4 ¶ 12, If health system organized this way it will be helpful in KII / 2:6 ¶ 16, this is the main thing to improve maternal and child care especially u… in KII / 3:8 ¶ 6, After counseling and aware mother it can be improved in Group discussion / 3:9 ¶ 8, It can improve those listed service (ANC, PNC, Breastfeeding and vacci… in Group discussion / 3:10 ¶ 8, Mother are already exposed to health education, but this one is direct… in Group discussion / 3:18 ¶ 12, It looks like mothers are tired of current service provision so the mH… in Group discussion / 3:19 ¶ 12, So in that case it can completely change the stream and interest. Or t… in Group discussion / 3:20 ¶ 12, They may even consider the increased risk of danger related to pregnan… in Group discussion / 3:43 ¶ 28, At the end this is very important piece of technology that can reduce… in Group discussion / 3:48 ¶ 31, Service at their home and Information and on time vaccination and Heal… in Group discussion / 3:49 ¶ 32, Healthy child feeding and behavioral change for mother that wll be lon… in Group discussion

1:57 ¶ 63 in interview analysis

Text quotation

Created by Girma Gilano on 3/8/2023

f it continue, it can be effective and be important than previous way of service provision

1 Codes:

- **Benefits of mHealth: Improve_MCH**

Comment: by Girma Gilano

> *3/12/2023 11:46:46 AM, merged with Effectiviness 3/12/2023 1:12:09 PM, merged with Decision_making 3/12/2023 1:12:55 PM, merged with Role_improvement*

25 Quotations:

1:6 ¶ 22, Non-use of the service because of negligence and forgetting can be im… in interview analysis / 1:26 ¶ 35, t can increase utilization better than that we have previously in interview analysis / 1:28 ¶ 36, Even since this digital it can further improve the service and increas… in interview analysis / 1:36 ¶ 40, This mHealth better than our previous our service in interview analysis / 1:43 ¶ 50, For maternal and child health the decision to take service is mothers… in interview analysis / 1:49 ¶ 56, Having information on time can improve the health of mothers and child… in interview analysis / 1:50 ¶ 57, further improve the already improving service in interview analysis / 1:57 ¶ 63, f it continue, it can be effective and be important than previous way… in interview analysis / 1:59 ¶ 64, I think whatever we put in to the community to improve health service… in interview analysis / 1:62 ¶ 66, This can improve and put our usual service one step forward in interview analysis / 1:74 ¶ 75, I don't think people can be affected negatively because they love to l… in interview analysis / 1:77 ¶ 77, Rural mothers are respectful; they do what they told to do, so fear of… in interview analysis / 1:78 ¶ 78, One thing I assure is it will increase up taking maternal and child he… in interview analysis / 1:83 ¶ 83, It can remind which is especially important for family planning. Witho… in interview analysis / 2:4 ¶ 12, If health system organized this way it will be helpful in KII / 2:6 ¶ 16, this is the main thing to improve maternal and child care especially u… in KII / 3:8 ¶ 6, After counseling and aware mother it can be improved in Group discussion / 3:9 ¶ 8, It can improve those listed service (ANC, PNC, Breastfeeding and vacci… in Group discussion / 3:10 ¶ 8, Mother are already exposed to health education, but this one is direct… in Group discussion / 3:18 ¶ 12, It looks like mothers are tired of current service provision so the mH… in Group discussion / 3:19 ¶ 12, So in that case it can completely change the stream and interest. Or t… in Group discussion / 3:20 ¶ 12, They may even consider the increased risk of danger related to pregnan… in Group discussion / 3:43 ¶ 28, At the end this is very important piece of technology that can reduce… in Group discussion / 3:48 ¶ 31, Service at their home and Information and on time vaccination and Heal… in Group discussion / 3:49 ¶ 32, Healthy child feeding and behavioral change for mother that wll be lon… in Group discussion

1:59 ¶ 64 in interview analysis

Text quotation

Created by Girma Gilano on 3/8/2023

I think whatever we put in to the community to improve health service are always bring improvement beside the some resistances that can be happen

1 Codes:

- **Benefits of mHealth: Improve_MCH**

Comment: by Girma Gilano

3/12/2023 11:46:46 AM, merged with Effectiviness 3/12/2023 1:12:09 PM, merged with Decision_making 3/12/2023 1:12:55 PM, merged with Role_improvement

25 Quotations:

1:6 ¶ 22, Non-use of the service because of negligence and forgetting can be im… in interview analysis / 1:26 ¶ 35, t can increase utilization better than that we have previously in interview analysis / 1:28 ¶ 36, Even since this digital it can further improve the service and increas… in interview analysis / 1:36 ¶ 40, This mHealth better than our previous our service in interview analysis / 1:43 ¶ 50, For maternal and child health the decision to take service is mothers… in interview analysis / 1:49 ¶ 56, Having information on time can improve the health of mothers and child… in interview analysis / 1:50 ¶ 57, further improve the already improving service in interview analysis / 1:57 ¶ 63, f it continue, it can be effective and be important than previous way… in interview analysis / 1:59 ¶ 64, I think whatever we put in to the community to improve health service… in interview analysis / 1:62 ¶ 66, This can improve and put our usual service one step forward in interview analysis / 1:74 ¶ 75, I don't think people can be affected negatively because they love to l… in interview analysis / 1:77 ¶ 77, Rural mothers are respectful; they do what they told to do, so fear of… in interview analysis / 1:78 ¶ 78, One thing I assure is it will increase up taking maternal and child he… in interview analysis / 1:83 ¶ 83, It can remind which is especially important for family planning. Witho… in interview analysis / 2:4 ¶ 12, If health system organized this way it will be helpful in KII / 2:6 ¶ 16, this is the main thing to improve maternal and child care especially u… in KII / 3:8 ¶ 6, After counseling and aware mother it can be improved in Group discussion / 3:9 ¶ 8, It can improve those listed service (ANC, PNC, Breastfeeding and vacci… in Group discussion / 3:10 ¶ 8, Mother are already exposed to health education, but this one is direct… in Group discussion / 3:18 ¶ 12, It looks like mothers are tired of current service provision so the mH… in Group discussion / 3:19 ¶ 12, So in that case it can completely change the stream and interest. Or t… in Group discussion / 3:20 ¶ 12, They may even consider the increased risk of danger related to pregnan… in Group discussion / 3:43 ¶ 28, At the end this is very important piece of technology that can reduce… in Group discussion / 3:48 ¶ 31, Service at their home and Information and on time vaccination and Heal… in Group discussion / 3:49 ¶ 32, Healthy child feeding and behavioral change for mother that wll be lon… in Group discussion

1:62 ¶ 66 in interview analysis

Text quotation

Created by Girma Gilano on 3/8/2023

This can improve and put our usual service one step forward

1 Codes:

- **Benefits of mHealth: Improve_MCH**

Comment: by Girma Gilano

3/12/2023 11:46:46 AM, merged with Effectiviness 3/12/2023 1:12:09 PM, merged with Decision_making 3/12/2023 1:12:55 PM, merged with Role_improvement

25 Quotations:

1:6 ¶ 22, Non-use of the service because of negligence and forgetting can be im… in interview analysis / 1:26 ¶ 35, t can increase utilization better than that we have previously in interview analysis / 1:28 ¶ 36, Even since this digital it can further improve the service and increas… in interview analysis / 1:36 ¶ 40, This mHealth better than our previous our service in interview analysis / 1:43 ¶ 50, For maternal and child health the decision to take service is mothers… in interview analysis / 1:49 ¶ 56, Having information on time can improve the health of mothers and child… in interview analysis / 1:50 ¶ 57, further improve the already improving service in interview analysis / 1:57 ¶ 63, f it continue, it can be effective and be important than previous way… in interview analysis / 1:59 ¶ 64, I think whatever we put in to the community to improve health service… in interview analysis / 1:62 ¶ 66, This can improve and put our usual service one step forward in interview analysis / 1:74 ¶ 75, I don't think people can be affected

negatively because they love to l… in interview analysis / 1:77 ¶ 77, Rural mothers are respectful; they do what they told to do, so fear of… in interview analysis / 1:78 ¶ 78, One thing I assure is it will increase up taking maternal and child he… in interview analysis / 1:83 ¶ 83, It can remind which is especially important for family planning. Witho… in interview analysis / 2:4 ¶ 12, If health system organized this way it will be helpful in KII / 2:6 ¶ 16, this is the main thing to improve maternal and child care especially u… in KII / 3:8 ¶ 6, After counseling and aware mother it can be improved in Group discussion / 3:9 ¶ 8, It can improve those listed service (ANC, PNC, Breastfeeding and vacci… in Group discussion / 3:10 ¶ 8, Mother are already exposed to health education, but this one is direct… in Group discussion / 3:18 ¶ 12, It looks like mothers are tired of current service provision so the mH… in Group discussion / 3:19 ¶ 12, So in that case it can completely change the stream and interest. Or t… in Group discussion / 3:20 ¶ 12, They may even consider the increased risk of danger related to pregnan… in Group discussion / 3:43 ¶ 28, At the end this is very important piece of technology that can reduce… in Group discussion / 3:48 ¶ 31, Service at their home and Information and on time vaccination and Heal… in Group discussion / 3:49 ¶ 32, Healthy child feeding and behavioral change for mother that wll be lon… in Group discussion

1:74 ¶ 75 in interview analysis

Text quotation

Created by Girma Gilano on 3/8/2023

I don’t think people can be affected negatively because they love to learn new things, especially, that improve service, they appreciate how government improve health

1 Codes:

- **Benefits of mHealth: Improve_MCH**

Comment: by Girma Gilano

> *3/12/2023 11:46:46 AM, merged with Effectiviness 3/12/2023 1:12:09 PM, merged with Decision_making 3/12/2023 1:12:55 PM, merged with Role_improvement*

25 Quotations:

1:6 ¶ 22, Non-use of the service because of negligence and forgetting can be im… in interview analysis / 1:26 ¶ 35, t can increase utilization better than that we have previously in interview analysis / 1:28 ¶ 36, Even since this digital it can further improve the service and increas… in interview analysis / 1:36 ¶ 40, This mHealth better than our previous our service in interview analysis / 1:43 ¶ 50, For maternal and child health the decision to take service is mothers… in interview analysis / 1:49 ¶ 56, Having information on time can improve the health of mothers and child… in interview analysis / 1:50 ¶ 57, further improve the already improving service in interview analysis / 1:57 ¶ 63, f it continue, it can be effective and be important than previous way… in interview analysis / 1:59 ¶ 64, I think whatever we put in to the community to improve health service… in interview analysis / 1:62 ¶ 66, This can improve and put our usual service one step forward in interview analysis / 1:74 ¶ 75, I don’t think people can be affected negatively because they love to l… in interview analysis / 1:77 ¶ 77, Rural mothers are respectful; they do what they told to do, so fear of… in interview analysis / 1:78 ¶ 78, One thing I assure is it will increase up taking maternal and child he… in interview analysis / 1:83 ¶ 83, It can remind which is especially important for family planning. Witho… in interview analysis / 2:4 ¶ 12, If health system organized this way it will be helpful in KII / 2:6 ¶ 16, this is the main thing to improve maternal and child care especially u… in KII / 3:8 ¶ 6, After counseling and aware mother it can be improved in Group discussion / 3:9 ¶ 8, It can improve those listed service (ANC, PNC, Breastfeeding and vacci… in Group discussion / 3:10 ¶ 8, Mother are already exposed to health education, but this one is direct… in Group discussion / 3:18 ¶ 12, It looks like mothers are tired of current service provision so the mH… in Group discussion / 3:19 ¶ 12, So in that case it can completely change the stream and interest. Or t… in Group discussion / 3:20 ¶ 12, They may even consider the increased risk of danger related to pregnan… in Group discussion / 3:43 ¶ 28, At the end this is very important piece of technology that can reduce… in Group discussion / 3:48 ¶ 31, Service at their home and Information and on time vaccination and Heal… in Group discussion / 3:49 ¶ 32, Healthy child feeding and behavioral change for mother that wll be lon… in Group discussion

1:77 ¶ 77 in interview analysis

Text quotation

Created by Girma Gilano on 3/8/2023, **modified** by Girma Gilano on 3/8/2023

Rural mothers are respectful; they do what they told to do, so fear of poor success is not a problem. Technology is not a new. Mobile is everywhere; messaging common so I don't there will be a problem. Teaching mothers will be important

2 Codes:

- **Benefits of mHealth: Help_mothers**

Comment: by Girma Gilano

> *3/12/2023 11:46:46 AM, merged with Effectiviness 3/12/2023 1:12:09 PM, merged with Decision_making 3/12/2023 1:12:55 PM, merged with Role_improvement*

27 Quotations:

1:41 ¶ 47, ion comes after understanding of benefits and harms in interview analysis / 1:45 ¶ 52, Accessibility can be improved mothers get information at their homes in interview analysis / 1:51 ¶ 58, She can access any information in interview analysis / 1:53 ¶ 59, I think mHealth can improve access, appointment on time, availability… in interview analysis / 1:65 ¶ 67, Her husband or children will also push her to go even if the condition… in interview analysis / 1:68 ¶ 70, Every system is getting digitalized so digitalizing health may have mu… in interview analysis / 1:77 ¶ 77, Rural mothers are respectful; they do what they told to do, so fear of… in interview analysis / 1:81 ¶ 80, pe mothers will turn to use mobiles, but only after getting its import… in interview analysis / 2:3 ¶ 11, It is helpful to also send information which are not appropriate in pe… in KII / 2:7 ¶ 17, Yeah, it will have effective change on community in KII / 2:9 ¶ 19, Yeah, previously mothers use the information they get during ANC, but… in KII / 2:11 ¶ 21, Information access, no transportation cost, and no professional resour… in KII / 2:36 ¶ 55, No need of motor or vehicles because it is mHealth. I will be happy to… in KII / 3:6 ¶ 5, They difference for mHealth is mother always get counseling or health… in Group discussion / 3:18 ¶ 12, It looks like mothers are tired of current service provision so the mH… in Group discussion / 3:23 ¶ 14, Previously, people think poor health system and there are many complia… in Group discussion / 3:24 ¶ 14, Over time this can change community perception and they may reebok mot… in Group discussion / 3:25 ¶ 14, There may be emerged women who are successful because of mHealth and t… in Group discussion / 3:27 ¶ 16, It can make the child health growth, for mothers to know danger signs,… in Group discussion / 3:38 ¶ 22, Of course having organized maternal data can also promote contacting a… in Group discussion / 3:41 ¶ 26, : It is a big opportunity to meet the patient again virtually especial… in Group discussion / 3:42 ¶ 26, Traditionally, when errors happen or if the patients go with the wrong… in Group discussion / 3:50 ¶ 33, Benefit for mothers for next pregnancies and healthy prospect for chil… in Group discussion / 3:59 ¶ 45, Satisfaction of their client service and familiarization with technolo… in Group discussion / 3:61 ¶ 47, Planning, decision making based on mHealth, and increased service take… in Group discussion / 3:62 ¶ 48, Support related to mHealth, fulfillment of deficiencies of human and o… in Group discussion / 3:67 ¶ 53, he respect for pregnancy mothers may think gave many births and not ca… in Group discussion

- **Benefits of mHealth: Improve_MCH**

Comment: by Girma Gilano

> *3/12/2023 11:46:46 AM, merged with Effectiviness 3/12/2023 1:12:09 PM, merged with Decision_making 3/12/2023 1:12:55 PM, merged with Role_improvement*

25 Quotations:

1:6 ¶ 22, Non-use of the service because of negligence and forgetting can be im… in interview analysis / 1:26 ¶ 35, t can increase utilization better than that we have previously in interview analysis / 1:28 ¶ 36,

Even since this digital it can further improve the service and increas… in interview analysis / 1:36 ¶ 40, This mHealth better than our previous our service in interview analysis / 1:43 ¶ 50, For maternal and child health the decision to take service is mothers… in interview analysis / 1:49 ¶ 56, Having information on time can improve the health of mothers and child… in interview analysis / 1:50 ¶ 57, further improve the already improving service in interview analysis / 1:57 ¶ 63, f it continue, it can be effective and be important than previous way… in interview analysis / 1:59 ¶ 64, I think whatever we put in to the community to improve health service… in interview analysis / 1:62 ¶ 66, This can improve and put our usual service one step forward in interview analysis / 1:74 ¶ 75, I don't think people can be affected negatively because they love to l… in interview analysis / 1:77 ¶ 77, Rural mothers are respectful; they do what they told to do, so fear of… in interview analysis / 1:78 ¶ 78, One thing I assure is it will increase up taking maternal and child he… in interview analysis / 1:83 ¶ 83, It can remind which is especially important for family planning. Witho… in interview analysis / 2:4 ¶ 12, If health system organized this way it will be helpful in KII / 2:6 ¶ 16, this is the main thing to improve maternal and child care especially u… in KII / 3:8 ¶ 6, After counseling and aware mother it can be improved in Group discussion / 3:9 ¶ 8, It can improve those listed service (ANC, PNC, Breastfeeding and vacci… in Group discussion / 3:10 ¶ 8, Mother are already exposed to health education, but this one is direct… in Group discussion / 3:18 ¶ 12, It looks like mothers are tired of current service provision so the mH… in Group discussion / 3:19 ¶ 12, So in that case it can completely change the stream and interest. Or t… in Group discussion / 3:20 ¶ 12, They may even consider the increased risk of danger related to pregnan… in Group discussion / 3:43 ¶ 28, At the end this is very important piece of technology that can reduce… in Group discussion / 3:48 ¶ 31, Service at their home and Information and on time vaccination and Heal… in Group discussion / 3:49 ¶ 32, Healthy child feeding and behavioral change for mother that wll be lon… in Group discussion

1:78 ¶ 78 in interview analysis

Text quotation

Created by Girma Gilano on 3/8/2023

One thing I assure is it will increase up taking maternal and child health service

1 Codes:

- **Benefits of mHealth: Improve_MCH**

Comment: by Girma Gilano

> *3/12/2023 11:46:46 AM, merged with Effectiviness 3/12/2023 1:12:09 PM, merged with Decision_making 3/12/2023 1:12:55 PM, merged with Role_improvement*

25 Quotations:

1:6 ¶ 22, Non-use of the service because of negligence and forgetting can be im… in interview analysis / 1:26 ¶ 35, t can increase utilization better than that we have previously in interview analysis / 1:28 ¶ 36, Even since this digital it can further improve the service and increas… in interview analysis / 1:36 ¶ 40, This mHealth better than our previous our service in interview analysis / 1:43 ¶ 50, For maternal and child health the decision to take service is mothers… in interview analysis / 1:49 ¶ 56, Having information on time can improve the health of mothers and child… in interview analysis / 1:50 ¶ 57, further improve the already improving service in interview analysis / 1:57 ¶ 63, f it continue, it can be effective and be important than previous way… in interview analysis / 1:59 ¶ 64, I think whatever we put in to the community to improve health service… in interview analysis / 1:62 ¶ 66, This can improve and put our usual service one step forward in interview analysis / 1:74 ¶ 75, I don't think people can be affected negatively because they love to l… in interview analysis / 1:77 ¶ 77, Rural mothers are respectful; they do what they told to do, so fear of… in interview analysis / 1:78 ¶ 78, One thing I assure is it will increase up taking maternal and child he… in interview analysis / 1:83 ¶ 83, It can remind which is especially important for family planning. Witho… in interview analysis / 2:4 ¶ 12, If health system organized this way it will be helpful in KII / 2:6 ¶ 16, this is the main thing to improve maternal and child care especially u… in KII / 3:8 ¶ 6, After counseling and aware mother it can be improved in Group discussion / 3:9 ¶ 8, It can improve those listed service (ANC, PNC, Breastfeeding and vacci… in Group discussion / 3:10 ¶ 8,

Mother are already exposed to health education, but this one is direct… in Group discussion / 3:18 ¶ 12, It looks like mothers are tired of current service provision so the mH… in Group discussion / 3:19 ¶ 12, So in that case it can completely change the stream and interest. Or t… in Group discussion / 3:20 ¶ 12, They may even consider the increased risk of danger related to pregnan… in Group discussion / 3:43 ¶ 28, At the end this is very important piece of technology that can reduce… in Group discussion / 3:48 ¶ 31, Service at their home and Information and on time vaccination and Heal… in Group discussion / 3:49 ¶ 32, Healthy child feeding and behavioral change for mother that wll be lon… in Group discussion

1:83 ¶ 83 in interview analysis

Text quotation

Created by Girma Gilano on 3/9/2023, **modified** by Girma Gilano on 3/9/2023

It can remind which is especially important for family planning. Without human travel to home, to tell teach mothers and no transportation cost. For mother who needs C/S and SC children, it will be helpful

1 Codes:

- **Benefits of mHealth: Improve_MCH**

Comment: by Girma Gilano

> *3/12/2023 11:46:46 AM, merged with Effectiviness 3/12/2023 1:12:09 PM, merged with Decision_making 3/12/2023 1:12:55 PM, merged with Role_improvement*

25 Quotations:

1:6 ¶ 22, Non-use of the service because of negligence and forgetting can be im… in interview analysis / 1:26 ¶ 35, t can increase utilization better than that we have previously in interview analysis / 1:28 ¶ 36, Even since this digital it can further improve the service and increas… in interview analysis / 1:36 ¶ 40, This mHealth better than our previous our service in interview analysis / 1:43 ¶ 50, For maternal and child health the decision to take service is mothers… in interview analysis / 1:49 ¶ 56, Having information on time can improve the health of mothers and child… in interview analysis / 1:50 ¶ 57, further improve the already improving service in interview analysis / 1:57 ¶ 63, f it continue, it can be effective and be important than previous way… in interview analysis / 1:59 ¶ 64, I think whatever we put in to the community to improve health service… in interview analysis / 1:62 ¶ 66, This can improve and put our usual service one step forward in interview analysis / 1:74 ¶ 75, I don't think people can be affected negatively because they love to l… in interview analysis / 1:77 ¶ 77, Rural mothers are respectful; they do what they told to do, so fear of… in interview analysis / 1:78 ¶ 78, One thing I assure is it will increase up taking maternal and child he… in interview analysis / 1:83 ¶ 83, It can remind which is especially important for family planning. Witho… in interview analysis / 2:4 ¶ 12, If health system organized this way it will be helpful in KII / 2:6 ¶ 16, this is the main thing to improve maternal and child care especially u… in KII / 3:8 ¶ 6, After counseling and aware mother it can be improved in Group discussion / 3:9 ¶ 8, It can improve those listed service (ANC, PNC, Breastfeeding and vacci… in Group discussion / 3:10 ¶ 8, Mother are already exposed to health education, but this one is direct… in Group discussion / 3:18 ¶ 12, It looks like mothers are tired of current service provision so the mH… in Group discussion / 3:19 ¶ 12, So in that case it can completely change the stream and interest. Or t… in Group discussion / 3:20 ¶ 12, They may even consider the increased risk of danger related to pregnan… in Group discussion / 3:43 ¶ 28, At the end this is very important piece of technology that can reduce… in Group discussion / 3:48 ¶ 31, Service at their home and Information and on time vaccination and Heal… in Group discussion / 3:49 ¶ 32, Healthy child feeding and behavioral change for mother that wll be lon… in Group discussion

2:4 ¶ 12 in KII

Text quotation

Created by Girma Gilano on 3/9/2023

If health system organized this way it will be helpful

1 Codes:

- **Benefits of mHealth: Improve_MCH**

  Comment: by Girma Gilano

  > *3/12/2023 11:46:46 AM, merged with Effectiviness 3/12/2023 1:12:09 PM, merged with Decision_making 3/12/2023 1:12:55 PM, merged with Role_improvement*

  25 Quotations:

  1:6 ¶ 22, Non-use of the service because of negligence and forgetting can be im… in interview analysis / 1:26 ¶ 35, t can increase utilization better than that we have previously in interview analysis / 1:28 ¶ 36, Even since this digital it can further improve the service and increas… in interview analysis / 1:36 ¶ 40, This mHealth better than our previous our service in interview analysis / 1:43 ¶ 50, For maternal and child health the decision to take service is mothers… in interview analysis / 1:49 ¶ 56, Having information on time can improve the health of mothers and child… in interview analysis / 1:50 ¶ 57, further improve the already improving service in interview analysis / 1:57 ¶ 63, f it continue, it can be effective and be important than previous way… in interview analysis / 1:59 ¶ 64, I think whatever we put in to the community to improve health service… in interview analysis / 1:62 ¶ 66, This can improve and put our usual service one step forward in interview analysis / 1:74 ¶ 75, I don't think people can be affected negatively because they love to l… in interview analysis / 1:77 ¶ 77, Rural mothers are respectful; they do what they told to do, so fear of… in interview analysis / 1:78 ¶ 78, One thing I assure is it will increase up taking maternal and child he… in interview analysis / 1:83 ¶ 83, It can remind which is especially important for family planning. Witho… in interview analysis / 2:4 ¶ 12, If health system organized this way it will be helpful in KII / 2:6 ¶ 16, this is the main thing to improve maternal and child care especially u… in KII / 3:8 ¶ 6, After counseling and aware mother it can be improved in Group discussion / 3:9 ¶ 8, It can improve those listed service (ANC, PNC, Breastfeeding and vacci… in Group discussion / 3:10 ¶ 8, Mother are already exposed to health education, but this one is direct… in Group discussion / 3:18 ¶ 12, It looks like mothers are tired of current service provision so the mH… in Group discussion / 3:19 ¶ 12, So in that case it can completely change the stream and interest. Or t… in Group discussion / 3:20 ¶ 12, They may even consider the increased risk of danger related to pregnan… in Group discussion / 3:43 ¶ 28, At the end this is very important piece of technology that can reduce… in Group discussion / 3:48 ¶ 31, Service at their home and Information and on time vaccination and Heal… in Group discussion / 3:49 ¶ 32, Healthy child feeding and behavioral change for mother that wll be lon… in Group discussion

2:6 ¶ 16 in KII

Text quotation

Created by Girma Gilano on 3/9/2023

this is the main thing to improve maternal and child care especially using message education

1 Codes:

- **Benefits of mHealth: Improve_MCH**

  Comment: by Girma Gilano

  > *3/12/2023 11:46:46 AM, merged with Effectiviness 3/12/2023 1:12:09 PM, merged with Decision_making 3/12/2023 1:12:55 PM, merged with Role_improvement*

25 Quotations:

1:6 ¶ 22, Non-use of the service because of negligence and forgetting can be im… in interview analysis / 1:26 ¶ 35, t can increase utilization better than that we have previously in interview analysis / 1:28 ¶ 36, Even since this digital it can further improve the service and increas… in interview analysis / 1:36 ¶ 40, This mHealth better than our previous our service in interview analysis / 1:43 ¶ 50, For maternal and child health the decision to take service is mothers… in interview analysis / 1:49 ¶ 56, Having information on time can improve the health of mothers and child… in interview analysis / 1:50 ¶ 57, further improve the already improving service in interview analysis / 1:57 ¶ 63, f it continue, it can be effective and be important than previous way… in interview analysis / 1:59 ¶ 64, I think whatever we put in to the community to improve health service… in interview analysis / 1:62 ¶ 66, This can improve and put our usual service one step forward in interview analysis / 1:74 ¶ 75, I don't think people can be affected negatively because they love to l… in interview analysis / 1:77 ¶ 77, Rural mothers are respectful; they do what they told to do, so fear of… in interview analysis / 1:78 ¶ 78, One thing I assure is it will increase up taking maternal and child he… in interview analysis / 1:83 ¶ 83, It can remind which is especially important for family planning. Witho… in interview analysis / 2:4 ¶ 12, If health system organized this way it will be helpful in KII / 2:6 ¶ 16, this is the main thing to improve maternal and child care especially u… in KII / 3:8 ¶ 6, After counseling and aware mother it can be improved in Group discussion / 3:9 ¶ 8, It can improve those listed service (ANC, PNC, Breastfeeding and vacci… in Group discussion / 3:10 ¶ 8, Mother are already exposed to health education, but this one is direct… in Group discussion / 3:18 ¶ 12, It looks like mothers are tired of current service provision so the mH… in Group discussion / 3:19 ¶ 12, So in that case it can completely change the stream and interest. Or t… in Group discussion / 3:20 ¶ 12, They may even consider the increased risk of danger related to pregnan… in Group discussion / 3:43 ¶ 28, At the end this is very important piece of technology that can reduce… in Group discussion / 3:48 ¶ 31, Service at their home and Information and on time vaccination and Heal… in Group discussion / 3:49 ¶ 32, Healthy child feeding and behavioral change for mother that wll be lon… in Group discussion

3:8 ¶ 6 in Group discussion

Text quotation

Created by Girma Gilano on 3/9/2023

After counseling and aware mother it can be improved

1 Codes:

- **Benefits of mHealth: Improve_MCH**

Comment: by Girma Gilano

> *3/12/2023 11:46:46 AM, merged with Effectiviness 3/12/2023 1:12:09 PM, merged with Decision_making 3/12/2023 1:12:55 PM, merged with Role_improvement*

25 Quotations:

1:6 ¶ 22, Non-use of the service because of negligence and forgetting can be im… in interview analysis / 1:26 ¶ 35, t can increase utilization better than that we have previously in interview analysis / 1:28 ¶ 36, Even since this digital it can further improve the service and increas… in interview analysis / 1:36 ¶ 40, This mHealth better than our previous our service in interview analysis / 1:43 ¶ 50, For maternal and child health the decision to take service is mothers… in interview analysis / 1:49 ¶ 56, Having information on time can improve the health of mothers and child… in interview analysis / 1:50 ¶ 57, further improve the already improving service in interview analysis / 1:57 ¶ 63, f it continue, it can be effective and be important than previous way… in interview analysis / 1:59 ¶ 64, I think whatever we put in to the community to improve health service… in interview analysis / 1:62 ¶ 66, This can improve and put our usual service one step forward in interview analysis / 1:74 ¶ 75, I don't think people can be affected negatively because they love to l… in interview analysis / 1:77 ¶ 77, Rural mothers are respectful; they do what they told to do, so fear of… in interview analysis / 1:78 ¶ 78, One thing I assure is it will increase up taking maternal and child he… in interview analysis / 1:83 ¶ 83, It can remind which is especially important for family planning. Witho… in interview analysis / 2:4 ¶ 12, If health system organized this way

it will be helpful in KII / 2:6 ¶ 16, this is the main thing to improve maternal and child care especially u… in KII / 3:8 ¶ 6, After counseling and aware mother it can be improved in Group discussion / 3:9 ¶ 8, It can improve those listed service (ANC, PNC, Breastfeeding and vacci… in Group discussion / 3:10 ¶ 8, Mother are already exposed to health education, but this one is direct… in Group discussion / 3:18 ¶ 12, It looks like mothers are tired of current service provision so the mH… in Group discussion / 3:19 ¶ 12, So in that case it can completely change the stream and interest. Or t… in Group discussion / 3:20 ¶ 12, They may even consider the increased risk of danger related to pregnan… in Group discussion / 3:43 ¶ 28, At the end this is very important piece of technology that can reduce… in Group discussion / 3:48 ¶ 31, Service at their home and Information and on time vaccination and Heal… in Group discussion / 3:49 ¶ 32, Healthy child feeding and behavioral change for mother that wll be lon… in Group discussion

3:9 ¶ 8 in Group discussion

Text quotation

Created by Girma Gilano on 3/9/2023, **modified** by Girma Gilano on 3/9/2023

It can improve those listed service (ANC, PNC, Breastfeeding and vaccine).

1 Codes:

- **Benefits of mHealth: Improve_MCH**

Comment: by Girma Gilano

> *3/12/2023 11:46:46 AM, merged with Effectiviness 3/12/2023 1:12:09 PM, merged with Decision_making 3/12/2023 1:12:55 PM, merged with Role_improvement*

25 Quotations:

1:6 ¶ 22, Non-use of the service because of negligence and forgetting can be im… in interview analysis / 1:26 ¶ 35, t can increase utilization better than that we have previously in interview analysis / 1:28 ¶ 36, Even since this digital it can further improve the service and increas… in interview analysis / 1:36 ¶ 40, This mHealth better than our previous our service in interview analysis / 1:43 ¶ 50, For maternal and child health the decision to take service is mothers… in interview analysis / 1:49 ¶ 56, Having information on time can improve the health of mothers and child… in interview analysis / 1:50 ¶ 57, further improve the already improving service in interview analysis / 1:57 ¶ 63, f it continue, it can be effective and be important than previous way… in interview analysis / 1:59 ¶ 64, I think whatever we put in to the community to improve health service… in interview analysis / 1:62 ¶ 66, This can improve and put our usual service one step forward in interview analysis / 1:74 ¶ 75, I don't think people can be affected negatively because they love to l… in interview analysis / 1:77 ¶ 77, Rural mothers are respectful; they do what they told to do, so fear of… in interview analysis / 1:78 ¶ 78, One thing I assure is it will increase up taking maternal and child he… in interview analysis / 1:83 ¶ 83, It can remind which is especially important for family planning. Witho… in interview analysis / 2:4 ¶ 12, If health system organized this way it will be helpful in KII / 2:6 ¶ 16, this is the main thing to improve maternal and child care especially u… in KII / 3:8 ¶ 6, After counseling and aware mother it can be improved in Group discussion / 3:9 ¶ 8, It can improve those listed service (ANC, PNC, Breastfeeding and vacci… in Group discussion / 3:10 ¶ 8, Mother are already exposed to health education, but this one is direct… in Group discussion / 3:18 ¶ 12, It looks like mothers are tired of current service provision so the mH… in Group discussion / 3:19 ¶ 12, So in that case it can completely change the stream and interest. Or t… in Group discussion / 3:20 ¶ 12, They may even consider the increased risk of danger related to pregnan… in Group discussion / 3:43 ¶ 28, At the end this is very important piece of technology that can reduce… in Group discussion / 3:48 ¶ 31, Service at their home and Information and on time vaccination and Heal… in Group discussion / 3:49 ¶ 32, Healthy child feeding and behavioral change for mother that wll be lon… in Group discussion

3:10 ¶ 8 in Group discussion

Text quotation

Created by Girma Gilano on 3/9/2023

Mother are already exposed to health education, but this one is directly motivates mothers at their home. I think it will improve maternal and child health if we use it appropriately

2 Codes:

● Benefits of mHealth: Improve_decision

Comment: by Girma Gilano

> *3/12/2023 11:46:46 AM, merged with Effectiviness 3/12/2023 1:12:09 PM, merged with Decision_making 3/12/2023 1:12:55 PM, merged with Role_improvement*

17 Quotations:

1:5 ¶ 20, t will improve mothers' time wastage for information that they can get… in interview analysis / 1:23 ¶ 33, will be helpful because it will remain in the mother's hand for a long… in interview analysis / 1:40 ¶ 45, s it can improve their decision making. This service could increase wo… in interview analysis / 1:42 ¶ 49, provide information to make decisions, motivate her to convince her hu… in interview analysis / 1:49 ¶ 56, Having information on time can improve the health of mothers and child… in interview analysis / 1:55 ¶ 60, mHealth can provide evidence to make decision and can improve everythi… in interview analysis / 1:64 ¶ 67, However, having this information in her hand will continuously strike… in interview analysis / 1:70 ¶ 71, Service can be improved through mHealth because atleast having informa… in interview analysis / 2:15 ¶ 27, Mothers should understand initially the important, but on our side I d… in KII / 3:6 ¶ 5, They difference for mHealth is mother always get counseling or health… in Group discussion / 3:10 ¶ 8, Mother are already exposed to health education, but this one is direct… in Group discussion / 3:20 ¶ 12, They may even consider the increased risk of danger related to pregnan… in Group discussion / 3:24 ¶ 14, Over time this can change community perception and they may reebok mot… in Group discussion / 3:37 ¶ 20, It can also introduce labor division because we may know who must serv… in Group discussion / 3:48 ¶ 31, Service at their home and Information and on time vaccination and Heal… in Group discussion / 3:52 ¶ 36, People will enjoy talking to others how health institution just concer… in Group discussion / 3:67 ¶ 53, he respect for pregnancy mothers may think gave many births and not ca… in Group discussion

● Benefits of mHealth: Improve_MCH

Comment: by Girma Gilano

> *3/12/2023 11:46:46 AM, merged with Effectiviness 3/12/2023 1:12:09 PM, merged with Decision_making 3/12/2023 1:12:55 PM, merged with Role_improvement*

25 Quotations:

1:6 ¶ 22, Non-use of the service because of negligence and forgetting can be im… in interview analysis / 1:26 ¶ 35, t can increase utilization better than that we have previously in interview analysis / 1:28 ¶ 36, Even since this digital it can further improve the service and increas… in interview analysis / 1:36 ¶ 40, This mHealth better than our previous our service in interview analysis / 1:43 ¶ 50, For maternal and child health the decision to take service is mothers… in interview analysis / 1:49 ¶ 56, Having information on time can improve the health of mothers and child… in interview analysis / 1:50 ¶ 57, further improve the already improving service in interview analysis / 1:57 ¶ 63, f it continue, it can be effective and be important than previous way… in interview analysis / 1:59 ¶ 64, I think whatever we put in to the community to improve health service… in interview analysis / 1:62 ¶ 66, This can improve and put our usual service one step forward in interview analysis / 1:74 ¶ 75, I don't think people can be affected negatively because they love to l… in interview analysis / 1:77 ¶ 77, Rural mothers are respectful; they do what they told to do, so fear of… in interview analysis / 1:78 ¶ 78, One thing I assure is it will increase up taking maternal and child he… in interview analysis / 1:83 ¶ 83, It can remind which is especially important for family planning. Witho… in interview analysis / 2:4 ¶ 12, If health system organized this way it will be helpful in KII / 2:6 ¶ 16, this is the main thing to improve maternal and child care especially u…

in KII / 3:8 ¶ 6, After counseling and aware mother it can be improved in Group discussion / 3:9 ¶ 8, It can improve those listed service (ANC, PNC, Breastfeeding and vacci… in Group discussion / 3:10 ¶ 8, Mother are already exposed to health education, but this one is direct… in Group discussion / 3:18 ¶ 12, It looks like mothers are tired of current service provision so the mH… in Group discussion / 3:19 ¶ 12, So in that case it can completely change the stream and interest. Or t… in Group discussion / 3:20 ¶ 12, They may even consider the increased risk of danger related to pregnan… in Group discussion / 3:43 ¶ 28, At the end this is very important piece of technology that can reduce… in Group discussion / 3:48 ¶ 31, Service at their home and Information and on time vaccination and Heal… in Group discussion / 3:49 ¶ 32, Healthy child feeding and behavioral change for mother that wll be lon… in Group discussion

3:18 ¶ 12 in Group discussion

Text quotation

Created by Girma Gilano on 3/9/2023

It looks like mothers are tired of current service provision so the mHealth may be liked by them

3 Codes:

- **Benefits of mHealth: Help_mothers**

Comment: by Girma Gilano

> *3/12/2023 11:46:46 AM, merged with Effectiviness 3/12/2023 1:12:09 PM, merged with Decision_making 3/12/2023 1:12:55 PM, merged with Role_improvement*

27 Quotations:

1:41 ¶ 47, ion comes after understanding of benefits and harms in interview analysis / 1:45 ¶ 52, Accessibility can be improved mothers get information at their homes in interview analysis / 1:51 ¶ 58, She can access any information in interview analysis / 1:53 ¶ 59, I think mHealth can improve access, appointment on time, availability… in interview analysis / 1:65 ¶ 67, Her husband or children will also push her to go even if the condition… in interview analysis / 1:68 ¶ 70, Every system is getting digitalized so digitalizing health may have mu… in interview analysis / 1:77 ¶ 77, Rural mothers are respectful; they do what they told to do, so fear of… in interview analysis / 1:81 ¶ 80, pe mothers will turn to use mobiles, but only after getting its import… in interview analysis / 2:3 ¶ 11, It is helpful to also send information which are not appropriate in pe… in KII / 2:7 ¶ 17, Yeah, it will have effective change on community in KII / 2:9 ¶ 19, Yeah, previously mothers use the information they get during ANC, but… in KII / 2:11 ¶ 21, Information access, no transportation cost, and no professional resour… in KII / 2:36 ¶ 55, No need of motor or vehicles because it is mHealth. I will be happy to… in KII / 3:6 ¶ 5, They difference for mHealth is mother always get counseling or health… in Group discussion / 3:18 ¶ 12, It looks like mothers are tired of current service provision so the mH… in Group discussion / 3:23 ¶ 14, Previously, people think poor health system and there are many complia… in Group discussion / 3:24 ¶ 14, Over time this can change community perception and they may reebok mot… in Group discussion / 3:25 ¶ 14, There may be emerged women who are successful because of mHealth and t… in Group discussion / 3:27 ¶ 16, It can make the child health growth, for mothers to know danger signs,… in Group discussion / 3:38 ¶ 22, Of course having organized maternal data can also promote contacting a… in Group discussion / 3:41 ¶ 26, : It is a big opportunity to meet the patient again virtually especial… in Group discussion / 3:42 ¶ 26, Traditionally, when errors happen or if the patients go with the wrong… in Group discussion / 3:50 ¶ 33, Benefit for mothers for next pregnancies and healthy prospect for chil… in Group discussion / 3:59 ¶ 45, Satisfaction of their client service and familiarization with technolo… in Group discussion / 3:61 ¶ 47, Planning, decision making based on mHealth, and increased service take… in Group discussion / 3:62 ¶ 48, Support related to mHealth, fulfillment of deficiencies of human and o… in Group discussion / 3:67 ¶ 53, he respect for pregnancy mothers may think gave many births and not ca… in Group discussion

- **Benefits of mHealth: Help_professional**

Comment: by Girma Gilano

3/12/2023 11:46:46 AM, merged with Effectiviness 3/12/2023 1:12:09 PM, merged with Decision_making 3/12/2023 1:12:55 PM, merged with Role_improvement

30 Quotations:

1:24 ¶ 34, We always give best service to our best capacity, but mHealth may adva… in interview analysis / 1:34 ¶ 39, As HEWs we cannot reach for every mother on time but text can easily r… in interview analysis / 1:36 ¶ 40, This mHealth better than our previous our service in interview analysis / 1:50 ¶ 57, further improve the already improving service in interview analysis / 1:56 ¶ 62, Our current ANC and other formats now request mother to register their… in interview analysis / 1:68 ¶ 70, Every system is getting digitalized so digitalizing health may have mu… in interview analysis / 1:82 ¶ 81, ers usually take what we tell them, it will be successful. I don't thi… in interview analysis / 2:3 ¶ 11, It is helpful to also send information which are not appropriate in pe… in KII / 2:7 ¶ 17, Yeah, it will have effective change on community in KII / 2:9 ¶ 19, Yeah, previously mothers use the information they get during ANC, but… in KII / 2:35 ¶ 54, Since we have many gaps this should be seen as an opportunity in KII / 3:2 ¶ 3, Previous teaching of mother through home to home is going to be throug… in Group discussion / 3:12 ¶ 8, For educated mothers messages will be option less in Group discussion / 3:13 ¶ 8, Starting from me we feed additional food at 2months because the kind o… in Group discussion / 3:18 ¶ 12, It looks like mothers are tired of current service provision so the mH… in Group discussion / 3:25 ¶ 14, There may be emerged women who are successful because of mHealth and t… in Group discussion / 3:29 ¶ 17, As a health professionals applying technology can only easy our burden… in Group discussion / 3:34 ¶ 19, ecause of mHealth we may every address of women and now we can also ca… in Group discussion / 3:35 ¶ 20, We will provide the service for mothers we know now, but previously we… in Group discussion / 3:38 ¶ 22, Of course having organized maternal data can also promote contacting a… in Group discussion / 3:41 ¶ 26, : It is a big opportunity to meet the patient again virtually especial… in Group discussion / 3:42 ¶ 26, Traditionally, when errors happen or if the patients go with the wrong… in Group discussion / 3:43 ¶ 28, At the end this is very important piece of technology that can reduce… in Group discussion / 3:46 ¶ 28, Those infrastructure and awareness creation in staffs, mothers, and co… in Group discussion / 3:52 ¶ 36, People will enjoy talking to others how health institution just concer… in Group discussion / 3:57 ¶ 42, Improve work environment as per the need of the technology and owning… in Group discussion / 3:58 ¶ 43, Aspiring more technologies and helping mothers, taking trainings, givi… in Group discussion / 3:60 ¶ 46, Experience with technology. Enable them to identify areas where mHealt… in Group discussion / 3:61 ¶ 47, Planning, decision making based on mHealth, and increased service take… in Group discussion / 3:62 ¶ 48, Support related to mHealth, fulfillment of deficiencies of human and o… in Group discussion

● Benefits of mHealth: Improve_MCH

Comment: by Girma Gilano

3/12/2023 11:46:46 AM, merged with Effectiviness 3/12/2023 1:12:09 PM, merged with Decision_making 3/12/2023 1:12:55 PM, merged with Role_improvement

25 Quotations:

1:6 ¶ 22, Non-use of the service because of negligence and forgetting can be im… in interview analysis / 1:26 ¶ 35, t can increase utilization better than that we have previously in interview analysis / 1:28 ¶ 36, Even since this digital it can further improve the service and increas… in interview analysis / 1:36 ¶ 40, This mHealth better than our previous our service in interview analysis / 1:43 ¶ 50, For maternal and child health the decision to take service is mothers… in interview analysis / 1:49 ¶ 56, Having information on time can improve the health of mothers and child… in interview analysis / 1:50 ¶ 57, further improve the already improving service in interview analysis / 1:57 ¶ 63, f it continue, it can be effective and be important than previous way… in interview analysis / 1:59 ¶ 64, I think whatever we put in to the community to improve health service… in interview analysis / 1:62 ¶ 66, This can improve and put our usual service one step forward in interview analysis / 1:74 ¶ 75, I don't think people can be affected negatively because they love to l… in interview analysis / 1:77 ¶ 77, Rural mothers are respectful; they do what they told to do, so fear of… in interview analysis / 1:78 ¶ 78, One thing I assure is it will increase up taking maternal and child he… in interview analysis / 1:83 ¶ 83, It can remind which is especially important for family planning. Witho… in interview analysis / 2:4 ¶ 12, If health system organized this way it will be helpful in KII / 2:6 ¶ 16, this is the main thing to improve maternal and child care especially u…

in KII / 3:8 ¶ 6, After counseling and aware mother it can be improved in Group discussion / 3:9 ¶ 8, It can improve those listed service (ANC, PNC, Breastfeeding and vacci… in Group discussion / 3:10 ¶ 8, Mother are already exposed to health education, but this one is direct… in Group discussion / 3:18 ¶ 12, It looks like mothers are tired of current service provision so the mH… in Group discussion / 3:19 ¶ 12, So in that case it can completely change the stream and interest. Or t… in Group discussion / 3:20 ¶ 12, They may even consider the increased risk of danger related to pregnan… in Group discussion / 3:43 ¶ 28, At the end this is very important piece of technology that can reduce… in Group discussion / 3:48 ¶ 31, Service at their home and Information and on time vaccination and Heal… in Group discussion / 3:49 ¶ 32, Healthy child feeding and behavioral change for mother that wll be lon… in Group discussion

3:19 ¶ 12 in Group discussion

Text quotation

Created by Girma Gilano on 3/9/2023

So in that case it can completely change the stream and interest. Or they may consider the attention they are getting.

1 Codes:

● Benefits of mHealth: Improve_MCH

Comment: by Girma Gilano

> *3/12/2023 11:46:46 AM, merged with Effectiviness 3/12/2023 1:12:09 PM, merged with Decision_making 3/12/2023 1:12:55 PM, merged with Role_improvement*

25 Quotations:

1:6 ¶ 22, Non-use of the service because of negligence and forgetting can be im… in interview analysis / 1:26 ¶ 35, t can increase utilization better than that we have previously in interview analysis / 1:28 ¶ 36, Even since this digital it can further improve the service and increas… in interview analysis / 1:36 ¶ 40, This mHealth better than our previous our service in interview analysis / 1:43 ¶ 50, For maternal and child health the decision to take service is mothers… in interview analysis / 1:49 ¶ 56, Having information on time can improve the health of mothers and child… in interview analysis / 1:50 ¶ 57, further improve the already improving service in interview analysis / 1:57 ¶ 63, f it continue, it can be effective and be important than previous way… in interview analysis / 1:59 ¶ 64, I think whatever we put in to the community to improve health service… in interview analysis / 1:62 ¶ 66, This can improve and put our usual service one step forward in interview analysis / 1:74 ¶ 75, I don't think people can be affected negatively because they love to l… in interview analysis / 1:77 ¶ 77, Rural mothers are respectful; they do what they told to do, so fear of… in interview analysis / 1:78 ¶ 78, One thing I assure is it will increase up taking maternal and child he… in interview analysis / 1:83 ¶ 83, It can remind which is especially important for family planning. Witho… in interview analysis / 2:4 ¶ 12, If health system organized this way it will be helpful in KII / 2:6 ¶ 16, this is the main thing to improve maternal and child care especially u… in KII / 3:8 ¶ 6, After counseling and aware mother it can be improved in Group discussion / 3:9 ¶ 8, It can improve those listed service (ANC, PNC, Breastfeeding and vacci… in Group discussion / 3:10 ¶ 8, Mother are already exposed to health education, but this one is direct… in Group discussion / 3:18 ¶ 12, It looks like mothers are tired of current service provision so the mH… in Group discussion / 3:19 ¶ 12, So in that case it can completely change the stream and interest. Or t… in Group discussion / 3:20 ¶ 12, They may even consider the increased risk of danger related to pregnan… in Group discussion / 3:43 ¶ 28, At the end this is very important piece of technology that can reduce… in Group discussion / 3:48 ¶ 31, Service at their home and Information and on time vaccination and Heal… in Group discussion / 3:49 ¶ 32, Healthy child feeding and behavioral change for mother that wll be lon… in Group discussion

3:20 ¶ 12 in Group discussion

Text quotation

Created by Girma Gilano on 3/9/2023

They may even consider the increased risk of danger related to pregnancy, child feeding, and vaccination. May be the emergence of new diseases can enforce them to vaccinate their children

2 Codes:

● Benefits of mHealth: Improve_decision

Comment: by Girma Gilano

> *3/12/2023 11:46:46 AM, merged with Effectiviness 3/12/2023 1:12:09 PM, merged with Decision_making 3/12/2023 1:12:55 PM, merged with Role_improvement*

17 Quotations:

1:5 ¶ 20, t will improve mothers' time wastage for information that they can get… in interview analysis / 1:23 ¶ 33, will be helpful because it will remain in the mother's hand for a long… in interview analysis / 1:40 ¶ 45, s it can improve their decision making. This service could increase wo… in interview analysis / 1:42 ¶ 49, provide information to make decisions, motivate her to convince her hu… in interview analysis / 1:49 ¶ 56, Having information on time can improve the health of mothers and child… in interview analysis / 1:55 ¶ 60, mHealth can provide evidence to make decision and can improve everythi… in interview analysis / 1:64 ¶ 67, However, having this information in her hand will continuously strike… in interview analysis / 1:70 ¶ 71, Service can be improved through mHealth because atleast having informa… in interview analysis / 2:15 ¶ 27, Mothers should understand initially the important, but on our side I d… in KII / 3:6 ¶ 5, They difference for mHealth is mother always get counseling or health… in Group discussion / 3:10 ¶ 8, Mother are already exposed to health education, but this one is direct… in Group discussion / 3:20 ¶ 12, They may even consider the increased risk of danger related to pregnan… in Group discussion / 3:24 ¶ 14, Over time this can change community perception and they may reebok mot… in Group discussion / 3:37 ¶ 20, It can also introduce labor division because we may know who must serv… in Group discussion / 3:48 ¶ 31, Service at their home and Information and on time vaccination and Heal… in Group discussion / 3:52 ¶ 36, People will enjoy talking to others how health institution just concer… in Group discussion / 3:67 ¶ 53, he respect for pregnancy mothers may think gave many births and not ca… in Group discussion

● Benefits of mHealth: Improve_MCH

Comment: by Girma Gilano

> *3/12/2023 11:46:46 AM, merged with Effectiviness 3/12/2023 1:12:09 PM, merged with Decision_making 3/12/2023 1:12:55 PM, merged with Role_improvement*

25 Quotations:

1:6 ¶ 22, Non-use of the service because of negligence and forgetting can be im… in interview analysis / 1:26 ¶ 35, t can increase utilization better than that we have previously in interview analysis / 1:28 ¶ 36, Even since this digital it can further improve the service and increas… in interview analysis / 1:36 ¶ 40, This mHealth better than our previous our service in interview analysis / 1:43 ¶ 50, For maternal and child health the decision to take service is mothers… in interview analysis / 1:49 ¶ 56, Having information on time can improve the health of mothers and child… in interview analysis / 1:50 ¶ 57, further improve the already improving service in interview analysis / 1:57 ¶ 63, f it continue, it can be effective and be important than previous way… in interview analysis / 1:59 ¶ 64, I think whatever we put in to the community to improve health service… in interview analysis / 1:62 ¶ 66, This can improve and put our usual service one step forward in interview analysis / 1:74 ¶ 75, I don't think people can be affected negatively because they love to l… in interview analysis / 1:77 ¶ 77, Rural mothers are respectful; they do what they told to do, so fear of… in interview analysis / 1:78 ¶ 78, One thing I assure is it will increase up taking maternal and child he… in interview analysis / 1:83 ¶ 83, It can remind which is especially important for family planning. Witho… in interview analysis / 2:4 ¶ 12, If health system organized this way

it will be helpful in KII / 2:6 ¶ 16, this is the main thing to improve maternal and child care especially u… in KII / 3:8 ¶ 6, After counseling and aware mother it can be improved in Group discussion / 3:9 ¶ 8, It can improve those listed service (ANC, PNC, Breastfeeding and vacci… in Group discussion / 3:10 ¶ 8, Mother are already exposed to health education, but this one is direct… in Group discussion / 3:18 ¶ 12, It looks like mothers are tired of current service provision so the mH… in Group discussion / 3:19 ¶ 12, So in that case it can completely change the stream and interest. Or t… in Group discussion / 3:20 ¶ 12, They may even consider the increased risk of danger related to pregnan… in Group discussion / 3:43 ¶ 28, At the end this is very important piece of technology that can reduce… in Group discussion / 3:48 ¶ 31, Service at their home and Information and on time vaccination and Heal… in Group discussion / 3:49 ¶ 32, Healthy child feeding and behavioral change for mother that wll be lon… in Group discussion

3:43 ¶ 28 in Group discussion

Text quotation

Created by Girma Gilano on 3/9/2023

At the end this is very important piece of technology that can reduce mortality of mothers and infant

2 Codes:

- **Benefits of mHealth: Help_professional**

Comment: by Girma Gilano

> *3/12/2023 11:46:46 AM, merged with Effectiviness 3/12/2023 1:12:09 PM, merged with Decision_making 3/12/2023 1:12:55 PM, merged with Role_improvement*

30 Quotations:

1:24 ¶ 34, We always give best service to our best capacity, but mHealth may adva… in interview analysis / 1:34 ¶ 39, As HEWs we cannot reach for every mother on time but text can easily r… in interview analysis / 1:36 ¶ 40, This mHealth better than our previous our service in interview analysis / 1:50 ¶ 57, further improve the already improving service in interview analysis / 1:56 ¶ 62, Our current ANC and other formats now request mother to register their… in interview analysis / 1:68 ¶ 70, Every system is getting digitalized so digitalizing health may have mu… in interview analysis / 1:82 ¶ 81, ers usually take what we tell them, it will be successful. I don't thi… in interview analysis / 2:3 ¶ 11, It is helpful to also send information which are not appropriate in pe… in KII / 2:7 ¶ 17, Yeah, it will have effective change on community in KII / 2:9 ¶ 19, Yeah, previously mothers use the information they get during ANC, but… in KII / 2:35 ¶ 54, Since we have many gaps this should be seen as an opportunity in KII / 3:2 ¶ 3, Previous teaching of mother through home to home is going to be throug… in Group discussion / 3:12 ¶ 8, For educated mothers messages will be option less in Group discussion / 3:13 ¶ 8, Starting from me we feed additional food at 2months because the kind o… in Group discussion / 3:18 ¶ 12, It looks like mothers are tired of current service provision so the mH… in Group discussion / 3:25 ¶ 14, There may be emerged women who are successful because of mHealth and t… in Group discussion / 3:29 ¶ 17, As a health professionals applying technology can only easy our burden… in Group discussion / 3:34 ¶ 19, ecause of mHealth we may every address of women and now we can also ca… in Group discussion / 3:35 ¶ 20, We will provide the service for mothers we know now, but previously we… in Group discussion / 3:38 ¶ 22, Of course having organized maternal data can also promote contacting a… in Group discussion / 3:41 ¶ 26, : It is a big opportunity to meet the patient again virtually especial… in Group discussion / 3:42 ¶ 26, Traditionally, when errors happen or if the patients go with the wrong… in Group discussion / 3:43 ¶ 28, At the end this is very important piece of technology that can reduce… in Group discussion / 3:46 ¶ 28, Those infrastructure and awareness creation in staffs, mothers, and co… in Group discussion / 3:52 ¶ 36, People will enjoy talking to others how health institution just concer… in Group discussion / 3:57 ¶ 42, Improve work environment as per the need of the technology and owning… in Group discussion / 3:58 ¶ 43, Aspiring more technologies and helping mothers, taking trainings, givi… in Group discussion / 3:60 ¶ 46, Experience with technology. Enable them to identify areas where mHealt… in Group discussion / 3:61 ¶ 47, Planning, decision making based

on mHealth, and increased service take… in Group discussion / 3:62 ¶ 48, Support related to mHealth, fulfillment of deficiencies of human and o… in Group discussion

● **Benefits of mHealth: Improve_MCH**

Comment: by Girma Gilano

> *3/12/2023 11:46:46 AM, merged with Effectiviness 3/12/2023 1:12:09 PM, merged with Decision_making 3/12/2023 1:12:55 PM, merged with Role_improvement*

25 Quotations:

1:6 ¶ 22, Non-use of the service because of negligence and forgetting can be im… in interview analysis / 1:26 ¶ 35, t can increase utilization better than that we have previously in interview analysis / 1:28 ¶ 36, Even since this digital it can further improve the service and increas… in interview analysis / 1:36 ¶ 40, This mHealth better than our previous our service in interview analysis / 1:43 ¶ 50, For maternal and child health the decision to take service is mothers… in interview analysis / 1:49 ¶ 56, Having information on time can improve the health of mothers and child… in interview analysis / 1:50 ¶ 57, further improve the already improving service in interview analysis / 1:57 ¶ 63, f it continue, it can be effective and be important than previous way… in interview analysis / 1:59 ¶ 64, I think whatever we put in to the community to improve health service… in interview analysis / 1:62 ¶ 66, This can improve and put our usual service one step forward in interview analysis / 1:74 ¶ 75, I don't think people can be affected negatively because they love to l… in interview analysis / 1:77 ¶ 77, Rural mothers are respectful; they do what they told to do, so fear of… in interview analysis / 1:78 ¶ 78, One thing I assure is it will increase up taking maternal and child he… in interview analysis / 1:83 ¶ 83, It can remind which is especially important for family planning. Witho… in interview analysis / 2:4 ¶ 12, If health system organized this way it will be helpful in KII / 2:6 ¶ 16, this is the main thing to improve maternal and child care especially u… in KII / 3:8 ¶ 6, After counseling and aware mother it can be improved in Group discussion / 3:9 ¶ 8, It can improve those listed service (ANC, PNC, Breastfeeding and vacci… in Group discussion / 3:10 ¶ 8, Mother are already exposed to health education, but this one is direct… in Group discussion / 3:18 ¶ 12, It looks like mothers are tired of current service provision so the mH… in Group discussion / 3:19 ¶ 12, So in that case it can completely change the stream and interest. Or t… in Group discussion / 3:20 ¶ 12, They may even consider the increased risk of danger related to pregnan… in Group discussion / 3:43 ¶ 28, At the end this is very important piece of technology that can reduce… in Group discussion / 3:48 ¶ 31, Service at their home and Information and on time vaccination and Heal… in Group discussion / 3:49 ¶ 32, Healthy child feeding and behavioral change for mother that wll be lon… in Group discussion

3:48 ¶ 31 in Group discussion

Text quotation

Created by Girma Gilano on 3/9/2023

Service at their home and Information and on time vaccination and Healthy child live

2 Codes:

● **Benefits of mHealth: Improve_decision**

Comment: by Girma Gilano

> *3/12/2023 11:46:46 AM, merged with Effectiviness 3/12/2023 1:12:09 PM, merged with Decision_making 3/12/2023 1:12:55 PM, merged with Role_improvement*

17 Quotations:

1:5 ¶ 20, t will improve mothers' time wastage for information that they can get… in interview analysis / 1:23 ¶ 33, will be helpful because it will remain in the mother's hand for a long… in interview analysis / 1:40 ¶ 45, s it can improve their decision making. This service could increase wo… in interview analysis /

1:42 ¶ 49, provide information to make decisions, motivate her to convince her hu… in interview analysis / 1:49 ¶ 56, Having information on time can improve the health of mothers and child… in interview analysis / 1:55 ¶ 60, mHealth can provide evidence to make decision and can improve everythi… in interview analysis / 1:64 ¶ 67, However, having this information in her hand will continuously strike… in interview analysis / 1:70 ¶ 71, Service can be improved through mHealth because atleast having informa… in interview analysis / 2:15 ¶ 27, Mothers should understand initially the important, but on our side I d… in KII / 3:6 ¶ 5, They difference for mHealth is mother always get counseling or health… in Group discussion / 3:10 ¶ 8, Mother are already exposed to health education, but this one is direct… in Group discussion / 3:20 ¶ 12, They may even consider the increased risk of danger related to pregnan… in Group discussion / 3:24 ¶ 14, Over time this can change community perception and they may reebok mot… in Group discussion / 3:37 ¶ 20, It can also introduce labor division because we may know who must serv… in Group discussion / 3:48 ¶ 31, Service at their home and Information and on time vaccination and Heal… in Group discussion / 3:52 ¶ 36, People will enjoy talking to others how health institution just concer… in Group discussion / 3:67 ¶ 53, he respect for pregnancy mothers may think gave many births and not ca… in Group discussion

Benefits of mHealth: Improve_MCH

Comment: by Girma Gilano

> *3/12/2023 11:46:46 AM, merged with Effectiviness 3/12/2023 1:12:09 PM, merged with Decision_making 3/12/2023 1:12:55 PM, merged with Role_improvement*

25 Quotations:

1:6 ¶ 22, Non-use of the service because of negligence and forgetting can be im… in interview analysis / 1:26 ¶ 35, t can increase utilization better than that we have previously in interview analysis / 1:28 ¶ 36, Even since this digital it can further improve the service and increas… in interview analysis / 1:36 ¶ 40, This mHealth better than our previous our service in interview analysis / 1:43 ¶ 50, For maternal and child health the decision to take service is mothers… in interview analysis / 1:49 ¶ 56, Having information on time can improve the health of mothers and child… in interview analysis / 1:50 ¶ 57, further improve the already improving service in interview analysis / 1:57 ¶ 63, f it continue, it can be effective and be important than previous way… in interview analysis / 1:59 ¶ 64, I think whatever we put in to the community to improve health service… in interview analysis / 1:62 ¶ 66, This can improve and put our usual service one step forward in interview analysis / 1:74 ¶ 75, I don’t think people can be affected negatively because they love to l… in interview analysis / 1:77 ¶ 77, Rural mothers are respectful; they do what they told to do, so fear of… in interview analysis / 1:78 ¶ 78, One thing I assure is it will increase up taking maternal and child he… in interview analysis / 1:83 ¶ 83, It can remind which is especially important for family planning. Witho… in interview analysis / 2:4 ¶ 12, If health system organized this way it will be helpful in KII / 2:6 ¶ 16, this is the main thing to improve maternal and child care especially u… in KII / 3:8 ¶ 6, After counseling and aware mother it can be improved in Group discussion / 3:9 ¶ 8, It can improve those listed service (ANC, PNC, Breastfeeding and vacci… in Group discussion / 3:10 ¶ 8, Mother are already exposed to health education, but this one is direct… in Group discussion / 3:18 ¶ 12, It looks like mothers are tired of current service provision so the mH… in Group discussion / 3:19 ¶ 12, So in that case it can completely change the stream and interest. Or t… in Group discussion / 3:20 ¶ 12, They may even consider the increased risk of danger related to pregnan… in Group discussion / 3:43 ¶ 28, At the end this is very important piece of technology that can reduce… in Group discussion / 3:48 ¶ 31, Service at their home and Information and on time vaccination and Heal… in Group discussion / 3:49 ¶ 32, Healthy child feeding and behavioral change for mother that wll be lon… in Group discussion

3:49 ¶ 32 in Group discussion

Text quotation

Created by Girma Gilano on 3/9/2023, **modified** by Girma Gilano on 3/9/2023

Healthy child feeding and behavioral change for mother that wll be long lasting

1 Codes:

Benefits of mHealth: Improve_MCH

Comment: by Girma Gilano

3/12/2023 11:46:46 AM, merged with Effectiviness 3/12/2023 1:12:09 PM, merged with Decision_making 3/12/2023 1:12:55 PM, merged with Role_improvement

25 Quotations:

1:6 ¶ 22, Non-use of the service because of negligence and forgetting can be im… in interview analysis / 1:26 ¶ 35, t can increase utilization better than that we have previously in interview analysis / 1:28 ¶ 36, Even since this digital it can further improve the service and increas… in interview analysis / 1:36 ¶ 40, This mHealth better than our previous our service in interview analysis / 1:43 ¶ 50, For maternal and child health the decision to take service is mothers… in interview analysis / 1:49 ¶ 56, Having information on time can improve the health of mothers and child… in interview analysis / 1:50 ¶ 57, further improve the already improving service in interview analysis / 1:57 ¶ 63, f it continue, it can be effective and be important than previous way… in interview analysis / 1:59 ¶ 64, I think whatever we put in to the community to improve health service… in interview analysis / 1:62 ¶ 66, This can improve and put our usual service one step forward in interview analysis / 1:74 ¶ 75, I don't think people can be affected negatively because they love to l… in interview analysis / 1:77 ¶ 77, Rural mothers are respectful; they do what they told to do, so fear of… in interview analysis / 1:78 ¶ 78, One thing I assure is it will increase up taking maternal and child he… in interview analysis / 1:83 ¶ 83, It can remind which is especially important for family planning. Witho… in interview analysis / 2:4 ¶ 12, If health system organized this way it will be helpful in KII / 2:6 ¶ 16, this is the main thing to improve maternal and child care especially u… in KII / 3:8 ¶ 6, After counseling and aware mother it can be improved in Group discussion / 3:9 ¶ 8, It can improve those listed service (ANC, PNC, Breastfeeding and vacci… in Group discussion / 3:10 ¶ 8, Mother are already exposed to health education, but this one is direct… in Group discussion / 3:18 ¶ 12, It looks like mothers are tired of current service provision so the mH… in Group discussion / 3:19 ¶ 12, So in that case it can completely change the stream and interest. Or t… in Group discussion / 3:20 ¶ 12, They may even consider the increased risk of danger related to pregnan… in Group discussion / 3:43 ¶ 28, At the end this is very important piece of technology that can reduce… in Group discussion / 3:48 ¶ 31, Service at their home and Information and on time vaccination and Heal… in Group discussion / 3:49 ¶ 32, Healthy child feeding and behavioral change for mother that wll be lon… in Group discussion

---

Challenges of mHealth impleentation: Acceptance

Created by Girma Gilano on 3/12/2023

Comment: by Girma Gilano

3/12/2023 12:56:31 PM, merged with Security 3/12/2023 12:56:54 PM, merged with Readiness

17 Quotations:

1:18 ¶ 30 in interview analysis

Text quotation

Created by Girma Gilano on 3/8/2023

I think it will improve if someone get information from her phone, she should be someone who actively using mobile

1 Codes:

- **Challenges of mHealth impleentation: Acceptance**

  Comment: by Girma Gilano

  > *3/12/2023 12:56:31 PM, merged with Security 3/12/2023 12:56:54 PM, merged with Readiness*

  #### 17 Quotations:

  1:18 ¶ 30, I think it will improve if someone get information from her phone, she… in interview analysis / 1:35 ¶ 39, Sometimes people may think mobile make women rude in interview analysis / 1:37 ¶ 40, t, when women take messages husband may think she is meeting someone.… in interview analysis / 1:84 ¶ 84, ometime our community may not say pregnancy until the conception reac… in interview analysis / 1:102 ¶ 101, Reading is one of the difficulties. May be absence of electricity, in… in interview analysis / 1:106 ¶ 106, All of them are solvable but may need time to solve in interview analysis / 2:8 ¶ 18, The practice will make better because our community need more such ser… in KII / 2:25 ¶ 40, Some projects phase out without making any impact in KII / 2:26 ¶ 41, Rejection by government/politics. Everyone is not modernized, but for… in KII / 2:27 ¶ 42, Competition between groups in KII / 2:28 ¶ 43, Message understanding of the mothers and questions related to why mHea… in KII / 2:33 ¶ 51, In my view, husbands my think different if some for example calling ev… in KII / 2:43 ¶ 61, Preparing place for installation, computers and mobiles are necessary,… in KII / 3:11 ¶ 8, Sometimes people may leave support and the program may fail but if str… in Group discussion / 3:44 ¶ 28, It is a reborn for health system, we hope more technology will come to… in Group discussion / 3:51 ¶ 35, No unique culture, it is all similar. In our culture I think people li… in Group discussion / 3:54 ¶ 38, : No cultural influence because our respect whatever health profession… in Group discussion

1:35 ¶ 39 in interview analysis

Text quotation

Created by Girma Gilano on 3/8/2023

Sometimes people may think mobile make women rude

1 Codes:

- **Challenges of mHealth impleentation: Acceptance**

  Comment: by Girma Gilano

  > *3/12/2023 12:56:31 PM, merged with Security 3/12/2023 12:56:54 PM, merged with Readiness*

  #### 17 Quotations:

  1:18 ¶ 30, I think it will improve if someone get information from her phone, she… in interview analysis / 1:35 ¶ 39, Sometimes people may think mobile make women rude in interview analysis / 1:37 ¶ 40, t, when women take messages husband may think she is meeting someone.… in interview analysis / 1:84 ¶ 84, ometime our community may not say pregnancy until the conception reac… in interview analysis / 1:102 ¶ 101, Reading is one of the difficulties. May be absence of electricity, in… in interview analysis / 1:106 ¶ 106, All of them are solvable but may need time to solve in interview analysis / 2:8 ¶ 18, The practice will make better because our community need more such ser… in KII / 2:25 ¶ 40, Some projects phase out without making any impact in KII / 2:26 ¶ 41, Rejection by government/politics. Everyone is not modernized, but for… in KII / 2:27 ¶ 42, Competition between groups in KII / 2:28 ¶ 43, Message understanding of the mothers and questions related to why mHea… in KII / 2:33 ¶ 51, In my view, husbands my think different if some for example calling ev… in KII / 2:43 ¶ 61, Preparing place for installation, computers and mobiles are necessary,… in KII / 3:11 ¶ 8, Sometimes people may leave

support and the program may fail but if str… in Group discussion / 3:44 ¶ 28, It is a reborn for health system, we hope more technology will come to… in Group discussion / 3:51 ¶ 35, No unique culture, it is all similar. In our culture I think people li… in Group discussion / 3:54 ¶ 38, : No cultural influence because our respect whatever health profession… in Group discussion

1:37 ¶ 40 in interview analysis

Text quotation

Created by Girma Gilano on 3/8/2023

t, when women take messages husband may think she is meeting someone. This service may increase workload on professionals

1 Codes:

- **Challenges of mHealth impleentation: Acceptance**

Comment: by Girma Gilano

> *3/12/2023 12:56:31 PM, merged with Security 3/12/2023 12:56:54 PM, merged with Readiness*

17 Quotations:

1:18 ¶ 30, I think it will improve if someone get information from her phone, she… in interview analysis / 1:35 ¶ 39, Sometimes people may think mobile make women rude in interview analysis / 1:37 ¶ 40, t, when women take messages husband may think she is meeting someone.… in interview analysis / 1:84 ¶ 84, ometime our community may not say pregnancy until the conception reac… in interview analysis / 1:102 ¶ 101, Reading is one of the difficulties. May be absence of electricity, in… in interview analysis / 1:106 ¶ 106, All of them are solvable but may need time to solve in interview analysis / 2:8 ¶ 18, The practice will make better because our community need more such ser… in KII / 2:25 ¶ 40, Some projects phase out without making any impact in KII / 2:26 ¶ 41, Rejection by government/politics. Everyone is not modernized, but for… in KII / 2:27 ¶ 42, Competition between groups in KII / 2:28 ¶ 43, Message understanding of the mothers and questions related to why mHea… in KII / 2:33 ¶ 51, In my view, husbands my think different if some for example calling ev… in KII / 2:43 ¶ 61, Preparing place for installation, computers and mobiles are necessary,… in KII / 3:11 ¶ 8, Sometimes people may leave support and the program may fail but if str… in Group discussion / 3:44 ¶ 28, It is a reborn for health system, we hope more technology will come to… in Group discussion / 3:51 ¶ 35, No unique culture, it is all similar. In our culture I think people li… in Group discussion / 3:54 ¶ 38, : No cultural influence because our respect whatever health profession… in Group discussion

1:84 ¶ 84 in interview analysis

Text quotation

Created by Girma Gilano on 3/9/2023, **modified** by Girma Gilano on 3/9/2023

ometime our community may not say pregnancy until the conception reach third months, so application of mHealth may declare it early so which may make some inconvenience

1 Codes:

- **Challenges of mHealth impleentation: Acceptance**

Comment: by Girma Gilano

3/12/2023 12:56:31 PM, merged with Security 3/12/2023 12:56:54 PM, merged with Readiness

17 Quotations:

1:18 ¶ 30, I think it will improve if someone get information from her phone, she… in interview analysis / 1:35 ¶ 39, Sometimes people may think mobile make women rude in interview analysis / 1:37 ¶ 40, t, when women take messages husband may think she is meeting someone.… in interview analysis / 1:84 ¶ 84, ometime our community may not say pregnancy until the conception reac… in interview analysis / 1:102 ¶ 101, Reading is one of the difficulties. May be absence of electricity, in… in interview analysis / 1:106 ¶ 106, All of them are solvable but may need time to solve in interview analysis / 2:8 ¶ 18, The practice will make better because our community need more such ser… in KII / 2:25 ¶ 40, Some projects phase out without making any impact in KII / 2:26 ¶ 41, Rejection by government/politics. Everyone is not modernized, but for… in KII / 2:27 ¶ 42, Competition between groups in KII / 2:28 ¶ 43, Message understanding of the mothers and questions related to why mHea… in KII / 2:33 ¶ 51, In my view, husbands my think different if some for example calling ev… in KII / 2:43 ¶ 61, Preparing place for installation, computers and mobiles are necessary,… in KII / 3:11 ¶ 8, Sometimes people may leave support and the program may fail but if str… in Group discussion / 3:44 ¶ 28, It is a reborn for health system, we hope more technology will come to… in Group discussion / 3:51 ¶ 35, No unique culture, it is all similar. In our culture I think people li… in Group discussion / 3:54 ¶ 38, : No cultural influence because our respect whatever health profession… in Group discussion

1:102 ¶ 101 in interview analysis

Text quotation

Created by Girma Gilano on 3/9/2023

Reading is one of the difficulties. May be absence of electricity, inability to read, lack of mobile phone, lack of interest, lack of support, uninviting political environmen

1 Codes:

- **Challenges of mHealth impleentation: Acceptance**

Comment: by Girma Gilano

3/12/2023 12:56:31 PM, merged with Security 3/12/2023 12:56:54 PM, merged with Readiness

17 Quotations:

1:18 ¶ 30, I think it will improve if someone get information from her phone, she… in interview analysis / 1:35 ¶ 39, Sometimes people may think mobile make women rude in interview analysis / 1:37 ¶ 40, t, when women take messages husband may think she is meeting someone.… in interview analysis / 1:84 ¶ 84, ometime our community may not say pregnancy until the conception reac… in interview analysis / 1:102 ¶ 101, Reading is one of the difficulties. May be absence of electricity, in… in interview analysis / 1:106 ¶ 106, All of them are solvable but may need time to solve in interview analysis / 2:8 ¶ 18, The practice will make better because our community need more such ser… in KII / 2:25 ¶ 40, Some projects phase out without making any impact in KII / 2:26 ¶ 41, Rejection by government/politics. Everyone is not modernized, but for… in KII / 2:27 ¶ 42, Competition between groups in KII / 2:28 ¶ 43, Message understanding of the mothers and questions related to why mHea… in KII / 2:33 ¶ 51, In my view, husbands my think different if some for example calling ev… in KII / 2:43 ¶ 61, Preparing place for installation, computers and mobiles are necessary,… in KII / 3:11 ¶ 8, Sometimes people may leave support and the program may fail but if str… in Group discussion / 3:44 ¶ 28, It is a reborn for health system, we hope more technology will come to… in Group discussion / 3:51 ¶ 35, No unique culture, it is all similar. In our culture I think people li… in Group discussion / 3:54 ¶ 38, : No cultural influence because our respect whatever health profession… in Group discussion

1:106 ¶ 106 in interview analysis

Text quotation

Created by Girma Gilano on 3/9/2023

All of them are solvable but may need time to solve

3 Codes:

- **Challenges of mHealth impleentation: Acceptance**

Comment: by Girma Gilano

> *3/12/2023 12:56:31 PM, merged with Security 3/12/2023 12:56:54 PM, merged with Readiness*

17 Quotations:

1:18 ¶ 30, I think it will improve if someone get information from her phone, she… in interview analysis / 1:35 ¶ 39, Sometimes people may think mobile make women rude in interview analysis / 1:37 ¶ 40, t, when women take messages husband may think she is meeting someone.… in interview analysis / 1:84 ¶ 84, ometime our community may not say pregnancy until the conception reac… in interview analysis / 1:102 ¶ 101, Reading is one of the difficulties. May be absence of electricity, in… in interview analysis / 1:106 ¶ 106, All of them are solvable but may need time to solve in interview analysis / 2:8 ¶ 18, The practice will make better because our community need more such ser… in KII / 2:25 ¶ 40, Some projects phase out without making any impact in KII / 2:26 ¶ 41, Rejection by government/politics. Everyone is not modernized, but for… in KII / 2:27 ¶ 42, Competition between groups in KII / 2:28 ¶ 43, Message understanding of the mothers and questions related to why mHea… in KII / 2:33 ¶ 51, In my view, husbands my think different if some for example calling ev… in KII / 2:43 ¶ 61, Preparing place for installation, computers and mobiles are necessary,… in KII / 3:11 ¶ 8, Sometimes people may leave support and the program may fail but if str… in Group discussion / 3:44 ¶ 28, It is a reborn for health system, we hope more technology will come to… in Group discussion / 3:51 ¶ 35, No unique culture, it is all similar. In our culture I think people li… in Group discussion / 3:54 ¶ 38, : No cultural influence because our respect whatever health profession… in Group discussion

- **Challenges of mHealth impleentation: Devices_handling**

Comment: by Girma Gilano

> *3/12/2023 12:56:31 PM, merged with Security 3/12/2023 12:56:54 PM, merged with Readiness*

12 Quotations:

1:54 ¶ 59, But no all mothers have mobiles. Some time they may loss their mobile… in interview analysis / 1:58 ¶ 63, This can improve the service but not all women have phone, many mother… in interview analysis / 1:63 ¶ 66, I can say it can be effective, but not all mother have mobiles, mobile… in interview analysis / 1:93 ¶ 93, Economy, maternal capacity of reading and understanding. Our mothers a… in interview analysis / 1:94 ¶ 94, Uneducated, inability to read, electricity, Mothers may not want to us… in interview analysis / 1:96 ¶ 96, May not have phone because of economy i.e. every mother may not phone… in interview analysis / 1:97 ¶ 97, Mother lost existing phone within the service period, broken Phone whi… in interview analysis / 1:106 ¶ 106, All of them are solvable but may need time to solve in interview analysis / 2:29 ¶ 44, Infants are not owners of the service but with family only and if fami… in KII / 3:28 ¶ 17, This service is possible and when mother do have mobile unless another… in Group discussion / 3:53 ¶ 37, Mobile is now part of the basic needs of our community. Mobile service… in Group discussion / 3:68 ¶ 55, Sustainability of the program is the concern, who will always look aft… in Group discussion

- ## Challenges of mHealth impleentation: Readiness

Comment: by Girma Gilano

> *3/12/2023 12:56:31 PM, merged with Security 3/12/2023 12:56:54 PM, merged with Readiness 3/19/2023 2:04:55 AM, merged with Challenges of mHealth impleentation: Resources 3/12/2023 12:56:31 PM, merged with Security 3/12/2023 12:56:54 PM, merged with Readiness*

15 Quotations:

1:79 ¶ 78, There could be a little challenge for this because the service is new in interview analysis / 1:85 ¶ 85, But we have to stress continuity and ownership, otherwise when there i… in interview analysis / 1:93 ¶ 93, Economy, maternal capacity of reading and understanding. Our mothers a… in interview analysis / 1:94 ¶ 94, Uneducated, inability to read, electricity, Mothers may not want to us… in interview analysis / 1:106 ¶ 106, All of them are solvable but may need time to solve in interview analysis / 2:1 ¶ 9, If anything necessary fulfilled, it is possible to implement. in KII / 2:18 ¶ 31, Yes, it will be difficult without infrastructure, it will be completel… in KII / 2:21 ¶ 34, This health facility is little bit less organized in materials compute… in KII / 2:22 ¶ 35, I think rural health institutions have a better maternal and child hea… in KII / 2:40 ¶ 60, I have to full resources to start this service. Awareness to health pr… in KII / 2:42 ¶ 63, Fulfilling infrastructures and giving trainings or creating awareness… in KII / 3:1 ¶ 3, May be it is more fit to towns and less likely for rural because of di… in Group discussion / 3:53 ¶ 37, Mobile is now part of the basic needs of our community. Mobile service… in Group discussion / 3:68 ¶ 55, Sustainability of the program is the concern, who will always look aft… in Group discussion / 3:70 ¶ 58, Resource to maintain the service. Interruption of supports, government… in Group discussion

2:8 ¶ 18 in KII

Text quotation

Created by Girma Gilano on 3/9/2023

The practice will make better because our community need more such service

1 Codes:

- ## Challenges of mHealth impleentation: Acceptance

Comment: by Girma Gilano

> *3/12/2023 12:56:31 PM, merged with Security 3/12/2023 12:56:54 PM, merged with Readiness*

17 Quotations:

1:18 ¶ 30, I think it will improve if someone get information from her phone, she… in interview analysis / 1:35 ¶ 39, Sometimes people may think mobile make women rude in interview analysis / 1:37 ¶ 40, t, when women take messages husband may think she is meeting someone.… in interview analysis / 1:84 ¶ 84, ometime our community may not say pregnancy until the conception reac… in interview analysis / 1:102 ¶ 101, Reading is one of the difficulties. May be absence of electricity, in… in interview analysis / 1:106 ¶ 106, All of them are solvable but may need time to solve in interview analysis / 2:8 ¶ 18, The practice will make better because our community need more such ser… in KII / 2:25 ¶ 40, Some projects phase out without making any impact in KII / 2:26 ¶ 41, Rejection by government/politics. Everyone is not modernized, but for… in KII / 2:27 ¶ 42, Competition between groups in KII / 2:28 ¶ 43, Message understanding of the mothers and questions related to why mHea… in KII / 2:33 ¶ 51, In my view, husbands my think different if some for example calling ev… in KII / 2:43 ¶ 61, Preparing place for installation, computers and mobiles are necessary,… in KII / 3:11 ¶ 8, Sometimes people may leave support and the program may fail but if str… in Group discussion / 3:44 ¶ 28, It is a reborn for health system, we hope more technology will come to… in Group discussion / 3:51 ¶ 35, No unique culture, it is

all similar. In our culture I think people li… in Group discussion / 3:54 ¶ 38, : No cultural influence because our respect whatever health profession… in Group discussion

2:25 ¶ 40 in KII

Text quotation

Created by Girma Gilano on 3/9/2023

Some projects phase out without making any impact

1 Codes:

- **Challenges of mHealth impleentation: Acceptance**

Comment: by Girma Gilano

> *3/12/2023 12:56:31 PM, merged with Security 3/12/2023 12:56:54 PM, merged with Readiness*

17 Quotations:

1:18 ¶ 30, I think it will improve if someone get information from her phone, she… in interview analysis / 1:35 ¶ 39, Sometimes people may think mobile make women rude in interview analysis / 1:37 ¶ 40, t, when women take messages husband may think she is meeting someone.… in interview analysis / 1:84 ¶ 84, ometime our community may not say pregnancy until the conception reac… in interview analysis / 1:102 ¶ 101, Reading is one of the difficulties. May be absence of electricity, in… in interview analysis / 1:106 ¶ 106, All of them are solvable but may need time to solve in interview analysis / 2:8 ¶ 18, The practice will make better because our community need more such ser… in KII / 2:25 ¶ 40, Some projects phase out without making any impact in KII / 2:26 ¶ 41, Rejection by government/politics. Everyone is not modernized, but for… in KII / 2:27 ¶ 42, Competition between groups in KII / 2:28 ¶ 43, Message understanding of the mothers and questions related to why mHea… in KII / 2:33 ¶ 51, In my view, husbands my think different if some for example calling ev… in KII / 2:43 ¶ 61, Preparing place for installation, computers and mobiles are necessary,… in KII / 3:11 ¶ 8, Sometimes people may leave support and the program may fail but if str… in Group discussion / 3:44 ¶ 28, It is a reborn for health system, we hope more technology will come to… in Group discussion / 3:51 ¶ 35, No unique culture, it is all similar. In our culture I think people li… in Group discussion / 3:54 ¶ 38, : No cultural influence because our respect whatever health profession… in Group discussion

2:26 ¶ 41 in KII

Text quotation

Created by Girma Gilano on 3/9/2023

Rejection by government/politics. Everyone is not modernized, but for using mothers it will be best

1 Codes:

- **Challenges of mHealth impleentation: Acceptance**

Comment: by Girma Gilano

> *3/12/2023 12:56:31 PM, merged with Security 3/12/2023 12:56:54 PM, merged with Readiness*

17 Quotations:

1:18 ¶ 30, I think it will improve if someone get information from her phone, she… in interview analysis / 1:35 ¶ 39, Sometimes people may think mobile make women rude in interview analysis / 1:37 ¶ 40, t, when women take messages husband may think she is meeting someone.… in interview analysis / 1:84 ¶ 84, ometime our community may not say pregnancy until the conception reac… in interview analysis / 1:102 ¶ 101, Reading is one of the difficulties. May be absence of electricity, in… in interview analysis / 1:106 ¶ 106, All of them are solvable but may need time to solve in interview analysis / 2:8 ¶ 18, The practice will make better because our community need more such ser… in KII / 2:25 ¶ 40, Some projects phase out without making any impact in KII / 2:26 ¶ 41, Rejection by government/politics. Everyone is not modernized, but for… in KII / 2:27 ¶ 42, Competition between groups in KII / 2:28 ¶ 43, Message understanding of the mothers and questions related to why mHea… in KII / 2:33 ¶ 51, In my view, husbands my think different if some for example calling ev… in KII / 2:43 ¶ 61, Preparing place for installation, computers and mobiles are necessary,… in KII / 3:11 ¶ 8, Sometimes people may leave support and the program may fail but if str… in Group discussion / 3:44 ¶ 28, It is a reborn for health system, we hope more technology will come to… in Group discussion / 3:51 ¶ 35, No unique culture, it is all similar. In our culture I think people li… in Group discussion / 3:54 ¶ 38, : No cultural influence because our respect whatever health profession… in Group discussion

2:27 ¶ 42 in KII

Text quotation

Created by Girma Gilano on 3/9/2023

Competition between groups

1 Codes:

- **Challenges of mHealth impleentation: Acceptance**

Comment: by Girma Gilano

> *3/12/2023 12:56:31 PM, merged with Security 3/12/2023 12:56:54 PM, merged with Readiness*

17 Quotations:

1:18 ¶ 30, I think it will improve if someone get information from her phone, she… in interview analysis / 1:35 ¶ 39, Sometimes people may think mobile make women rude in interview analysis / 1:37 ¶ 40, t, when women take messages husband may think she is meeting someone.… in interview analysis / 1:84 ¶ 84, ometime our community may not say pregnancy until the conception reac… in interview analysis / 1:102 ¶ 101, Reading is one of the difficulties. May be absence of electricity, in… in interview analysis / 1:106 ¶ 106, All of them are solvable but may need time to solve in interview analysis / 2:8 ¶ 18, The practice will make better because our community need more such ser… in KII / 2:25 ¶ 40, Some projects phase out without making any impact in KII / 2:26 ¶ 41, Rejection by government/politics. Everyone is not modernized, but for… in KII / 2:27 ¶ 42, Competition between groups in KII / 2:28 ¶ 43, Message understanding of the mothers and questions related to why mHea… in KII / 2:33 ¶ 51, In my view, husbands my think different if some for example calling ev… in KII / 2:43 ¶ 61, Preparing place for installation, computers and mobiles are necessary,… in KII / 3:11 ¶ 8, Sometimes people may leave support and the program may fail but if str… in Group discussion / 3:44 ¶ 28, It is a reborn for health system, we hope more technology will come to… in Group discussion / 3:51 ¶ 35, No unique culture, it is all similar. In our culture I think people li… in Group discussion / 3:54 ¶ 38, : No cultural influence because our respect whatever health profession… in Group discussion

2:28 ¶ 43 in KII

Text quotation

Created by Girma Gilano on 3/9/2023

Message understanding of the mothers and questions related to why mHealth

1 Codes:

- **Challenges of mHealth impleentation: Acceptance**

Comment: by Girma Gilano

> *3/12/2023 12:56:31 PM, merged with Security 3/12/2023 12:56:54 PM, merged with Readiness*

17 Quotations:

1:18 ¶ 30, I think it will improve if someone get information from her phone, she… in interview analysis / 1:35 ¶ 39, Sometimes people may think mobile make women rude in interview analysis / 1:37 ¶ 40, t, when women take messages husband may think she is meeting someone.… in interview analysis / 1:84 ¶ 84, ometime our community may not say pregnancy until the conception reac… in interview analysis / 1:102 ¶ 101, Reading is one of the difficulties. May be absence of electricity, in… in interview analysis / 1:106 ¶ 106, All of them are solvable but may need time to solve in interview analysis / 2:8 ¶ 18, The practice will make better because our community need more such ser… in KII / 2:25 ¶ 40, Some projects phase out without making any impact in KII / 2:26 ¶ 41, Rejection by government/politics. Everyone is not modernized, but for… in KII / 2:27 ¶ 42, Competition between groups in KII / 2:28 ¶ 43, Message understanding of the mothers and questions related to why mHea… in KII / 2:33 ¶ 51, In my view, husbands my think different if some for example calling ev… in KII / 2:43 ¶ 61, Preparing place for installation, computers and mobiles are necessary,… in KII / 3:11 ¶ 8, Sometimes people may leave support and the program may fail but if str… in Group discussion / 3:44 ¶ 28, It is a reborn for health system, we hope more technology will come to… in Group discussion / 3:51 ¶ 35, No unique culture, it is all similar. In our culture I think people li… in Group discussion / 3:54 ¶ 38, : No cultural influence because our respect whatever health profession… in Group discussion

2:33 ¶ 51 in KII

Text quotation

Created by Girma Gilano on 3/9/2023

In my view, husbands my think different if some for example calling every time

1 Codes:

- **Challenges of mHealth impleentation: Acceptance**

Comment: by Girma Gilano

> *3/12/2023 12:56:31 PM, merged with Security 3/12/2023 12:56:54 PM, merged with Readiness*

17 Quotations:

1:18 ¶ 30, I think it will improve if someone get information from her phone, she… in interview analysis / 1:35 ¶ 39, Sometimes people may think mobile make women rude in interview analysis / 1:37 ¶ 40, t, when women take messages husband may think she is meeting someone.… in interview analysis / 1:84 ¶ 84, ometime our community may not say pregnancy until the conception reac… in interview analysis / 1:102 ¶ 101, Reading is one of the difficulties. May be absence of electricity, in… in interview analysis / 1:106 ¶ 106, All of them are solvable but may need time to solve in interview analysis / 2:8 ¶ 18, The practice will make better because our community need more such ser… in KII / 2:25 ¶ 40, Some projects

phase out without making any impact in KII / 2:26 ¶ 41, Rejection by government/politics. Everyone is not modernized, but for… in KII / 2:27 ¶ 42, Competition between groups in KII / 2:28 ¶ 43, Message understanding of the mothers and questions related to why mHea… in KII / 2:33 ¶ 51, In my view, husbands my think different if some for example calling ev… in KII / 2:43 ¶ 61, Preparing place for installation, computers and mobiles are necessary,… in KII / 3:11 ¶ 8, Sometimes people may leave support and the program may fail but if str… in Group discussion / 3:44 ¶ 28, It is a reborn for health system, we hope more technology will come to… in Group discussion / 3:51 ¶ 35, No unique culture, it is all similar. In our culture I think people li… in Group discussion / 3:54 ¶ 38, : No cultural influence because our respect whatever health profession… in Group discussion

2:43 ¶ 61 in KII

Text quotation

Created by Girma Gilano on 3/9/2023

Preparing place for installation, computers and mobiles are necessary, and staff awareness

1 Codes:

- **Challenges of mHealth impleentation: Acceptance**

Comment: by Girma Gilano

> *3/12/2023 12:56:31 PM, merged with Security 3/12/2023 12:56:54 PM, merged with Readiness*

17 Quotations:

1:18 ¶ 30, I think it will improve if someone get information from her phone, she… in interview analysis / 1:35 ¶ 39, Sometimes people may think mobile make women rude in interview analysis / 1:37 ¶ 40, t, when women take messages husband may think she is meeting someone.… in interview analysis / 1:84 ¶ 84, ometime our community may not say pregnancy until the conception reac… in interview analysis / 1:102 ¶ 101, Reading is one of the difficulties. May be absence of electricity, in… in interview analysis / 1:106 ¶ 106, All of them are solvable but may need time to solve in interview analysis / 2:8 ¶ 18, The practice will make better because our community need more such ser… in KII / 2:25 ¶ 40, Some projects phase out without making any impact in KII / 2:26 ¶ 41, Rejection by government/politics. Everyone is not modernized, but for… in KII / 2:27 ¶ 42, Competition between groups in KII / 2:28 ¶ 43, Message understanding of the mothers and questions related to why mHea… in KII / 2:33 ¶ 51, In my view, husbands my think different if some for example calling ev… in KII / 2:43 ¶ 61, Preparing place for installation, computers and mobiles are necessary,… in KII / 3:11 ¶ 8, Sometimes people may leave support and the program may fail but if str… in Group discussion / 3:44 ¶ 28, It is a reborn for health system, we hope more technology will come to… in Group discussion / 3:51 ¶ 35, No unique culture, it is all similar. In our culture I think people li… in Group discussion / 3:54 ¶ 38, : No cultural influence because our respect whatever health profession… in Group discussion

3:11 ¶ 8 in Group discussion

Text quotation

Created by Girma Gilano on 3/9/2023

Sometimes people may leave support and the program may fail but if strictly followed it will be important

1 Codes:

- **Challenges of mHealth impleentation: Acceptance**

  Comment: by Girma Gilano

  > *3/12/2023 12:56:31 PM, merged with Security 3/12/2023 12:56:54 PM, merged with Readiness*

  17 Quotations:

  1:18 ¶ 30, I think it will improve if someone get information from her phone, she… in interview analysis / 1:35 ¶ 39, Sometimes people may think mobile make women rude in interview analysis / 1:37 ¶ 40, t, when women take messages husband may think she is meeting someone.… in interview analysis / 1:84 ¶ 84, ometime our community may not say pregnancy until the conception reac… in interview analysis / 1:102 ¶ 101, Reading is one of the difficulties. May be absence of electricity, in… in interview analysis / 1:106 ¶ 106, All of them are solvable but may need time to solve in interview analysis / 2:8 ¶ 18, The practice will make better because our community need more such ser… in KII / 2:25 ¶ 40, Some projects phase out without making any impact in KII / 2:26 ¶ 41, Rejection by government/politics. Everyone is not modernized, but for… in KII / 2:27 ¶ 42, Competition between groups in KII / 2:28 ¶ 43, Message understanding of the mothers and questions related to why mHea… in KII / 2:33 ¶ 51, In my view, husbands my think different if some for example calling ev… in KII / 2:43 ¶ 61, Preparing place for installation, computers and mobiles are necessary,… in KII / 3:11 ¶ 8, Sometimes people may leave support and the program may fail but if str… in Group discussion / 3:44 ¶ 28, It is a reborn for health system, we hope more technology will come to… in Group discussion / 3:51 ¶ 35, No unique culture, it is all similar. In our culture I think people li… in Group discussion / 3:54 ¶ 38, : No cultural influence because our respect whatever health profession… in Group discussion

3:44 ¶ 28 in Group discussion

Text quotation

Created by Girma Gilano on 3/9/2023

It is a reborn for health system, we hope more technology will come to resurrect or health care.

1 Codes:

- **Challenges of mHealth impleentation: Acceptance**

  Comment: by Girma Gilano

  > *3/12/2023 12:56:31 PM, merged with Security 3/12/2023 12:56:54 PM, merged with Readiness*

  17 Quotations:

  1:18 ¶ 30, I think it will improve if someone get information from her phone, she… in interview analysis / 1:35 ¶ 39, Sometimes people may think mobile make women rude in interview analysis / 1:37 ¶ 40, t, when women take messages husband may think she is meeting someone.… in interview analysis / 1:84 ¶ 84, ometime our community may not say pregnancy until the conception reac… in interview analysis / 1:102 ¶ 101, Reading is one of the difficulties. May be absence of electricity, in… in interview analysis / 1:106 ¶ 106, All of them are solvable but may need time to solve in interview analysis / 2:8 ¶ 18, The practice will make better because our community need more such ser… in KII / 2:25 ¶ 40, Some projects phase out without making any impact in KII / 2:26 ¶ 41, Rejection by government/politics. Everyone is not modernized, but for… in KII / 2:27 ¶ 42, Competition between groups in KII / 2:28 ¶ 43, Message understanding of the mothers and questions related to why mHea… in KII / 2:33 ¶ 51, In my view, husbands my think different if some for example calling ev… in KII / 2:43 ¶ 61, Preparing place for installation, computers and mobiles are necessary,… in KII / 3:11 ¶ 8, Sometimes people may leave support and the program may fail but if str… in Group discussion / 3:44 ¶ 28, It is a reborn for health

system, we hope more technology will come to… in Group discussion / 3:51 ¶ 35, No unique culture, it is all similar. In our culture I think people li… in Group discussion / 3:54 ¶ 38, : No cultural influence because our respect whatever health profession… in Group discussion

3:51 ¶ 35 in Group discussion

Text quotation

Created by Girma Gilano on 3/9/2023

No unique culture, it is all similar. In our culture I think people like learning new things. Even it is not helpful since provided by health professional, mothers always respect. I can't see any culture contradict the technology

1 Codes:

- **Challenges of mHealth impleentation: Acceptance**

Comment: by Girma Gilano

> *3/12/2023 12:56:31 PM, merged with Security 3/12/2023 12:56:54 PM, merged with Readiness*

17 Quotations:

1:18 ¶ 30, I think it will improve if someone get information from her phone, she… in interview analysis / 1:35 ¶ 39, Sometimes people may think mobile make women rude in interview analysis / 1:37 ¶ 40, t, when women take messages husband may think she is meeting someone.… in interview analysis / 1:84 ¶ 84, ometime our community may not say pregnancy until the conception reac… in interview analysis / 1:102 ¶ 101, Reading is one of the difficulties. May be absence of electricity, in… in interview analysis / 1:106 ¶ 106, All of them are solvable but may need time to solve in interview analysis / 2:8 ¶ 18, The practice will make better because our community need more such ser… in KII / 2:25 ¶ 40, Some projects phase out without making any impact in KII / 2:26 ¶ 41, Rejection by government/politics. Everyone is not modernized, but for… in KII / 2:27 ¶ 42, Competition between groups in KII / 2:28 ¶ 43, Message understanding of the mothers and questions related to why mHea… in KII / 2:33 ¶ 51, In my view, husbands my think different if some for example calling ev… in KII / 2:43 ¶ 61, Preparing place for installation, computers and mobiles are necessary,… in KII / 3:11 ¶ 8, Sometimes people may leave support and the program may fail but if str… in Group discussion / 3:44 ¶ 28, It is a reborn for health system, we hope more technology will come to… in Group discussion / 3:51 ¶ 35, No unique culture, it is all similar. In our culture I think people li… in Group discussion / 3:54 ¶ 38, : No cultural influence because our respect whatever health profession… in Group discussion

3:54 ¶ 38 in Group discussion

Text quotation

Created by Girma Gilano on 3/9/2023

: No cultural influence because our respect whatever health professionals order

1 Codes:

- **Challenges of mHealth impleentation: Acceptance**

Comment: by Girma Gilano

3/12/2023 12:56:31 PM, merged with Security 3/12/2023 12:56:54 PM, merged with Readiness

17 Quotations:

1:18 ¶ 30, I think it will improve if someone get information from her phone, she… in interview analysis / 1:35 ¶ 39, Sometimes people may think mobile make women rude in interview analysis / 1:37 ¶ 40, t, when women take messages husband may think she is meeting someone…. in interview analysis / 1:84 ¶ 84, ometime our community may not say pregnancy until the conception reac… in interview analysis / 1:102 ¶ 101, Reading is one of the difficulties. May be absence of electricity, in… in interview analysis / 1:106 ¶ 106, All of them are solvable but may need time to solve in interview analysis / 2:8 ¶ 18, The practice will make better because our community need more such ser… in KII / 2:25 ¶ 40, Some projects phase out without making any impact in KII / 2:26 ¶ 41, Rejection by government/politics. Everyone is not modernized, but for… in KII / 2:27 ¶ 42, Competition between groups in KII / 2:28 ¶ 43, Message understanding of the mothers and questions related to why mHea… in KII / 2:33 ¶ 51, In my view, husbands my think different if some for example calling ev… in KII / 2:43 ¶ 61, Preparing place for installation, computers and mobiles are necessary,… in KII / 3:11 ¶ 8, Sometimes people may leave support and the program may fail but if str… in Group discussion / 3:44 ¶ 28, It is a reborn for health system, we hope more technology will come to… in Group discussion / 3:51 ¶ 35, No unique culture, it is all similar. In our culture I think people li… in Group discussion / 3:54 ¶ 38, : No cultural influence because our respect whatever health profession… in Group discussion

---

● Challenges of mHealth impleentation: Awarneess

Created by Girma Gilano on 3/12/2023

Comment: by Girma Gilano

3/12/2023 12:56:31 PM, merged with Security 3/12/2023 12:56:54 PM, merged with Readiness

11 Quotations:

1:8 ¶ 23 in interview analysis

Text quotation

Created by Girma Gilano on 3/8/2023

here when a child is born it feels like good responsibility to give diet immediately

1 Codes:

● Challenges of mHealth impleentation: Awarneess

Comment: by Girma Gilano

3/12/2023 12:56:31 PM, merged with Security 3/12/2023 12:56:54 PM, merged with Readiness

11 Quotations:

1:8 ¶ 23, here when a child is born it feels like good responsibility to give di… in interview analysis / 1:9 ¶ 23, People are proud of this early feeding/food/ in interview analysis / 1:15 ¶ 28, We know usually even

hospitals allow zero level milk and that may be c… in interview analysis / 1:66 ¶ 68, messages should consider educational level and understanding of mother… in interview analysis / 1:91 ¶ 90, If the mother is not someone who read the message directly, the childr… in interview analysis / 1:92 ¶ 91, When the mother takes these service without the knowledge of her husba… in interview analysis / 1:99 ¶ 99, There is always an obstacle but can be reduced by awareness creation.… in interview analysis / 2:10 ¶ 20, Since most people mothers use mobile I think this is the better way of… in KII / 2:24 ¶ 39, If no mobile in mothers' hand. Uneducated mothers in KII / 3:5 ¶ 4, Whatever it is, we counsel them all the time and they knew that they w… in Group discussion / 3:63 ¶ 50, Maternal education, phone, maternal work behavior, and lack of awaren… in Group discussion

1:9 ¶ 23 in interview analysis

Text quotation

Created by Girma Gilano on 3/8/2023

People are proud of this early feeding/food/

1 Codes:

- **Challenges of mHealth impleentation: Awarneess**

Comment: by Girma Gilano

> *3/12/2023 12:56:31 PM, merged with Security 3/12/2023 12:56:54 PM, merged with Readiness*

11 Quotations:

1:8 ¶ 23, here when a child is born it feels like good responsibility to give di… in interview analysis / 1:9 ¶ 23, People are proud of this early feeding/food/ in interview analysis / 1:15 ¶ 28, We know usually even hospitals allow zero level milk and that may be c… in interview analysis / 1:66 ¶ 68, messages should consider educational level and understanding of mother… in interview analysis / 1:91 ¶ 90, If the mother is not someone who read the message directly, the childr… in interview analysis / 1:92 ¶ 91, When the mother takes these service without the knowledge of her husba… in interview analysis / 1:99 ¶ 99, There is always an obstacle but can be reduced by awareness creation.… in interview analysis / 2:10 ¶ 20, Since most people mothers use mobile I think this is the better way of… in KII / 2:24 ¶ 39, If no mobile in mothers' hand. Uneducated mothers in KII / 3:5 ¶ 4, Whatever it is, we counsel them all the time and they knew that they w… in Group discussion / 3:63 ¶ 50, Maternal education, phone, maternal work behavior, and lack of awaren… in Group discussion

1:15 ¶ 28 in interview analysis

Text quotation

Created by Girma Gilano on 3/8/2023

We know usually even hospitals allow zero level milk and that may be confusing mother to give milk at eerily time

1 Codes:

- **Challenges of mHealth impleentation: Awarneess**

Comment: by Girma Gilano

> *3/12/2023 12:56:31 PM, merged with Security 3/12/2023 12:56:54 PM, merged with Readiness*

11 Quotations:

1:8 ¶ 23, here when a child is born it feels like good responsibility to give di… in interview analysis / 1:9 ¶ 23, People are proud of this early feeding/food/ in interview analysis / 1:15 ¶ 28, We know usually even hospitals allow zero level milk and that may be c… in interview analysis / 1:66 ¶ 68, messages should consider educational level and understanding of mother… in interview analysis / 1:91 ¶ 90, If the mother is not someone who read the message directly, the childr… in interview analysis / 1:92 ¶ 91, When the mother takes these service without the knowledge of her husba… in interview analysis / 1:99 ¶ 99, There is always an obstacle but can be reduced by awareness creation.… in interview analysis / 2:10 ¶ 20, Since most people mothers use mobile I think this is the better way of… in KII / 2:24 ¶ 39, If no mobile in mothers' hand. Uneducated mothers in KII / 3:5 ¶ 4, Whatever it is, we counsel them all the time and they knew that they w… in Group discussion / 3:63 ¶ 50, Maternal education, phone, maternal work behavior, and lack of awaren… in Group discussion

1:66 ¶ 68 in interview analysis

Text quotation

Created by Girma Gilano on 3/8/2023

messages should consider educational level and understanding of mothers

1 Codes:

- **Challenges of mHealth impleentation: Awarneess**

Comment: by Girma Gilano

> *3/12/2023 12:56:31 PM, merged with Security 3/12/2023 12:56:54 PM, merged with Readiness*

11 Quotations:

1:8 ¶ 23, here when a child is born it feels like good responsibility to give di… in interview analysis / 1:9 ¶ 23, People are proud of this early feeding/food/ in interview analysis / 1:15 ¶ 28, We know usually even hospitals allow zero level milk and that may be c… in interview analysis / 1:66 ¶ 68, messages should consider educational level and understanding of mother… in interview analysis / 1:91 ¶ 90, If the mother is not someone who read the message directly, the childr… in interview analysis / 1:92 ¶ 91, When the mother takes these service without the knowledge of her husba… in interview analysis / 1:99 ¶ 99, There is always an obstacle but can be reduced by awareness creation.… in interview analysis / 2:10 ¶ 20, Since most people mothers use mobile I think this is the better way of… in KII / 2:24 ¶ 39, If no mobile in mothers' hand. Uneducated mothers in KII / 3:5 ¶ 4, Whatever it is, we counsel them all the time and they knew that they w… in Group discussion / 3:63 ¶ 50, Maternal education, phone, maternal work behavior, and lack of awaren… in Group discussion

1:91 ¶ 90 in interview analysis

Text quotation

Created by Girma Gilano on 3/9/2023

If the mother is not someone who read the message directly, the children or husband might need awareness too

1 Codes:

- **Challenges of mHealth impleentation: Awarneess**

  Comment: by Girma Gilano

  > *3/12/2023 12:56:31 PM, merged with Security 3/12/2023 12:56:54 PM, merged with Readiness*

  #### 11 Quotations:

  1:8 ¶ 23, here when a child is born it feels like good responsibility to give di… in interview analysis / 1:9 ¶ 23, People are proud of this early feeding/food/ in interview analysis / 1:15 ¶ 28, We know usually even hospitals allow zero level milk and that may be c… in interview analysis / 1:66 ¶ 68, messages should consider educational level and understanding of mother… in interview analysis / 1:91 ¶ 90, If the mother is not someone who read the message directly, the childr… in interview analysis / 1:92 ¶ 91, When the mother takes these service without the knowledge of her husba… in interview analysis / 1:99 ¶ 99, There is always an obstacle but can be reduced by awareness creation.… in interview analysis / 2:10 ¶ 20, Since most people mothers use mobile I think this is the better way of… in KII / 2:24 ¶ 39, If no mobile in mothers' hand. Uneducated mothers in KII / 3:5 ¶ 4, Whatever it is, we counsel them all the time and they knew that they w… in Group discussion / 3:63 ¶ 50, Maternal education, phone, maternal work behavior, and lack of awaren… in Group discussion

1:92 ¶ 91 in interview analysis

Text quotation

Created by Girma Gilano on 3/9/2023

When the mother takes these service without the knowledge of her husband that can be a problem when he find out. So the husband should be aware too. May be only when mother search for someone to read her message outside the HH

1 Codes:

- **Challenges of mHealth impleentation: Awarneess**

  Comment: by Girma Gilano

  > *3/12/2023 12:56:31 PM, merged with Security 3/12/2023 12:56:54 PM, merged with Readiness*

  #### 11 Quotations:

  1:8 ¶ 23, here when a child is born it feels like good responsibility to give di… in interview analysis / 1:9 ¶ 23, People are proud of this early feeding/food/ in interview analysis / 1:15 ¶ 28, We know usually even hospitals allow zero level milk and that may be c… in interview analysis / 1:66 ¶ 68, messages should consider educational level and understanding of mother… in interview analysis / 1:91 ¶ 90, If the mother is not someone who read the message directly, the childr… in interview analysis / 1:92 ¶ 91, When the mother takes these service without the knowledge of her husba… in interview analysis / 1:99 ¶ 99, There is always an obstacle but can be reduced by awareness creation.… in interview analysis / 2:10 ¶ 20, Since most people mothers use mobile I think this is the better way of… in KII / 2:24 ¶ 39, If no mobile in mothers' hand. Uneducated mothers in KII / 3:5 ¶ 4, Whatever it is, we counsel them all the time and they knew that they w… in Group discussion / 3:63 ¶ 50, Maternal education, phone, maternal work behavior, and lack of awaren… in Group discussion

1:99 ¶ 99 in interview analysis

Text quotation

Created by Girma Gilano on 3/9/2023, **modified** by Girma Gilano on 3/9/2023

There is always an obstacle but can be reduced by awareness creation. Readability, education, mobile access, network and refusal may be problems

1 Codes:

- **Challenges of mHealth impleentation: Awarneess**

  Comment: by Girma Gilano

  > *3/12/2023 12:56:31 PM, merged with Security 3/12/2023 12:56:54 PM, merged with Readiness*

  11 Quotations:

  1:8 ¶ 23, here when a child is born it feels like good responsibility to give di… in interview analysis / 1:9 ¶ 23, People are proud of this early feeding/food/ in interview analysis / 1:15 ¶ 28, We know usually even hospitals allow zero level milk and that may be c… in interview analysis / 1:66 ¶ 68, messages should consider educational level and understanding of mother… in interview analysis / 1:91 ¶ 90, If the mother is not someone who read the message directly, the childr… in interview analysis / 1:92 ¶ 91, When the mother takes these service without the knowledge of her husba… in interview analysis / 1:99 ¶ 99, There is always an obstacle but can be reduced by awareness creation.… in interview analysis / 2:10 ¶ 20, Since most people mothers use mobile I think this is the better way of… in KII / 2:24 ¶ 39, If no mobile in mothers' hand. Uneducated mothers in KII / 3:5 ¶ 4, Whatever it is, we counsel them all the time and they knew that they w… in Group discussion / 3:63 ¶ 50, Maternal education, phone, maternal work behavior, and lack of awaren… in Group discussion

2:10 ¶ 20 in KII

Text quotation

Created by Girma Gilano on 3/9/2023

Since most people mothers use mobile I think this is the better way of getting best out of it

1 Codes:

- **Challenges of mHealth impleentation: Awarneess**

  Comment: by Girma Gilano

  > *3/12/2023 12:56:31 PM, merged with Security 3/12/2023 12:56:54 PM, merged with Readiness*

  11 Quotations:

  1:8 ¶ 23, here when a child is born it feels like good responsibility to give di… in interview analysis / 1:9 ¶ 23, People are proud of this early feeding/food/ in interview analysis / 1:15 ¶ 28, We know usually even hospitals allow zero level milk and that may be c… in interview analysis / 1:66 ¶ 68, messages should consider educational level and understanding of mother… in interview analysis / 1:91 ¶ 90, If the mother is not someone who read the message directly, the childr… in interview analysis / 1:92 ¶ 91, When the mother takes these service without the knowledge of her husba… in interview analysis / 1:99 ¶ 99, There is always an obstacle but can be reduced by awareness creation.… in interview analysis / 2:10 ¶ 20, Since most people mothers use mobile I think this is the better way of… in KII / 2:24 ¶ 39, If no mobile in

mothers' hand. Uneducated mothers in KII / 3:5 ¶ 4, Whatever it is, we counsel them all the time and they knew that they w… in Group discussion / 3:63 ¶ 50, Maternal education, phone, maternal work behavior, and lack of awaren… in Group discussion

2:24 ¶ 39 in KII

Text quotation

Created by Girma Gilano on 3/9/2023

If no mobile in mothers' hand. Uneducated mothers

1 Codes:

- **Challenges of mHealth impleentation: Awarneess**

Comment: by Girma Gilano

> *3/12/2023 12:56:31 PM, merged with Security 3/12/2023 12:56:54 PM, merged with Readiness*

11 Quotations:

1:8 ¶ 23, here when a child is born it feels like good responsibility to give di… in interview analysis / 1:9 ¶ 23, People are proud of this early feeding/food/ in interview analysis / 1:15 ¶ 28, We know usually even hospitals allow zero level milk and that may be c… in interview analysis / 1:66 ¶ 68, messages should consider educational level and understanding of mother… in interview analysis / 1:91 ¶ 90, If the mother is not someone who read the message directly, the childr… in interview analysis / 1:92 ¶ 91, When the mother takes these service without the knowledge of her husba… in interview analysis / 1:99 ¶ 99, There is always an obstacle but can be reduced by awareness creation.… in interview analysis / 2:10 ¶ 20, Since most people mothers use mobile I think this is the better way of… in KII / 2:24 ¶ 39, If no mobile in mothers' hand. Uneducated mothers in KII / 3:5 ¶ 4, Whatever it is, we counsel them all the time and they knew that they w… in Group discussion / 3:63 ¶ 50, Maternal education, phone, maternal work behavior, and lack of awaren… in Group discussion

3:5 ¶ 4 in Group discussion

Text quotation

Created by Girma Gilano on 3/9/2023

Whatever it is, we counsel them all the time and they knew that they will face medically dangerous problem if ANC, PNC, and feeding of children was not followed accordingly. However, they do not follow and take more service even they consider as no difference

1 Codes:

- **Challenges of mHealth impleentation: Awarneess**

Comment: by Girma Gilano

> *3/12/2023 12:56:31 PM, merged with Security 3/12/2023 12:56:54 PM, merged with Readiness*

11 Quotations:

1:8 ¶ 23, here when a child is born it feels like good responsibility to give di… in interview analysis / 1:9 ¶ 23, People are proud of this early feeding/food/ in interview analysis / 1:15 ¶ 28, We know usually even hospitals allow zero level milk and that may be c… in interview analysis / 1:66 ¶ 68, messages should consider educational level and understanding of mother… in interview analysis / 1:91 ¶ 90, If the mother is not someone who read the message directly, the childr… in interview analysis / 1:92 ¶ 91, When the mother takes these service without the knowledge of her husba… in interview analysis / 1:99 ¶ 99, There is always an obstacle but can be reduced by awareness creation.… in interview analysis / 2:10 ¶ 20, Since most people mothers use mobile I think this is the better way of… in KII / 2:24 ¶ 39, If no mobile in mothers' hand. Uneducated mothers in KII / 3:5 ¶ 4, Whatever it is, we counsel them all the time and they knew that they w… in Group discussion / 3:63 ¶ 50, Maternal education, phone, maternal work behavior, and lack of awaren… in Group discussion

3:63 ¶ 50 in Group discussion

Text quotation

Created by Girma Gilano on 3/9/2023

Maternal education, phone, maternal work behavior, and lack of awareness of mHealth benefits

1 Codes:

- **Challenges of mHealth impleentation: Awarneess**

Comment: by Girma Gilano

> *3/12/2023 12:56:31 PM, merged with Security 3/12/2023 12:56:54 PM, merged with Readiness*

11 Quotations:

1:8 ¶ 23, here when a child is born it feels like good responsibility to give di… in interview analysis / 1:9 ¶ 23, People are proud of this early feeding/food/ in interview analysis / 1:15 ¶ 28, We know usually even hospitals allow zero level milk and that may be c… in interview analysis / 1:66 ¶ 68, messages should consider educational level and understanding of mother… in interview analysis / 1:91 ¶ 90, If the mother is not someone who read the message directly, the childr… in interview analysis / 1:92 ¶ 91, When the mother takes these service without the knowledge of her husba… in interview analysis / 1:99 ¶ 99, There is always an obstacle but can be reduced by awareness creation.… in interview analysis / 2:10 ¶ 20, Since most people mothers use mobile I think this is the better way of… in KII / 2:24 ¶ 39, If no mobile in mothers' hand. Uneducated mothers in KII / 3:5 ¶ 4, Whatever it is, we counsel them all the time and they knew that they w… in Group discussion / 3:63 ¶ 50, Maternal education, phone, maternal work behavior, and lack of awaren… in Group discussion

---

● Challenges of mHealth impleentation: Devices_handling

Created by Girma Gilano on 3/12/2023, **modified** by Girma Gilano on 3/12/2023

Comment: by Girma Gilano

> *3/12/2023 12:56:31 PM, merged with Security 3/12/2023 12:56:54 PM, merged with Readiness*

12 Quotations:

1:54 ¶ 59 in interview analysis

Text quotation

Created by Girma Gilano on 3/8/2023

But no all mothers have mobiles. Some time they may loss their mobile while on appointment and there is still the chance of losing mother even with mHealth

1 Codes:

- **Challenges of mHealth impleentation: Devices_handling**

  Comment: by Girma Gilano

  > *3/12/2023 12:56:31 PM, merged with Security 3/12/2023 12:56:54 PM, merged with Readiness*

  12 Quotations:

  1:54 ¶ 59, But no all mothers have mobiles. Some time they may loss their mobile… in interview analysis / 1:58 ¶ 63, This can improve the service but not all women have phone, many mother… in interview analysis / 1:63 ¶ 66, I can say it can be effective, but not all mother have mobiles, mobile… in interview analysis / 1:93 ¶ 93, Economy, maternal capacity of reading and understanding. Our mothers a… in interview analysis / 1:94 ¶ 94, Uneducated, inability to read, electricity, Mothers may not want to us… in interview analysis / 1:96 ¶ 96, May not have phone because of economy i.e. every mother may not phone… in interview analysis / 1:97 ¶ 97, Mother lost existing phone within the service period, broken Phone whi… in interview analysis / 1:106 ¶ 106, All of them are solvable but may need time to solve in interview analysis / 2:29 ¶ 44, Infants are not owners of the service but with family only and if fami… in KII / 3:28 ¶ 17, This service is possible and when mother do have mobile unless another… in Group discussion / 3:53 ¶ 37, Mobile is now part of the basic needs of our community. Mobile service… in Group discussion / 3:68 ¶ 55, Sustainability of the program is the concern, who will always look aft… in Group discussion

1:58 ¶ 63 in interview analysis

Text quotation

Created by Girma Gilano on 3/8/2023, **modified** by Girma Gilano on 3/8/2023

This can improve the service but not all women have phone, many mothers

1 Codes:

- **Challenges of mHealth impleentation: Devices_handling**

  Comment: by Girma Gilano

  > *3/12/2023 12:56:31 PM, merged with Security 3/12/2023 12:56:54 PM, merged with Readiness*

  12 Quotations:

  1:54 ¶ 59, But no all mothers have mobiles. Some time they may loss their mobile… in interview analysis / 1:58 ¶ 63, This can improve the service but not all women have phone, many mother… in interview analysis / 1:63 ¶ 66, I can say it can be effective, but not all mother have mobiles, mobile… in interview analysis / 1:93 ¶ 93, Economy, maternal capacity of reading and understanding. Our mothers a… in

interview analysis / 1:94 ¶ 94, Uneducated, inability to read, electricity, Mothers may not want to us… in interview analysis / 1:96 ¶ 96, May not have phone because of economy i.e. every mother may not phone… in interview analysis / 1:97 ¶ 97, Mother lost existing phone within the service period, broken Phone whi… in interview analysis / 1:106 ¶ 106, All of them are solvable but may need time to solve in interview analysis / 2:29 ¶ 44, Infants are not owners of the service but with family only and if fami… in KII / 3:28 ¶ 17, This service is possible and when mother do have mobile unless another… in Group discussion / 3:53 ¶ 37, Mobile is now part of the basic needs of our community. Mobile service… in Group discussion / 3:68 ¶ 55, Sustainability of the program is the concern, who will always look aft… in Group discussion

1:63 ¶ 66 in interview analysis

Text quotation

Created by Girma Gilano on 3/8/2023

I can say it can be effective, but not all mother have mobiles, mobile is now expensive

1 Codes:

- **Challenges of mHealth impleentation: Devices_handling**

Comment: by Girma Gilano

> *3/12/2023 12:56:31 PM, merged with Security 3/12/2023 12:56:54 PM, merged with Readiness*

12 Quotations:

1:54 ¶ 59, But no all mothers have mobiles. Some time they may loss their mobile… in interview analysis / 1:58 ¶ 63, This can improve the service but not all women have phone, many mother… in interview analysis / 1:63 ¶ 66, I can say it can be effective, but not all mother have mobiles, mobile… in interview analysis / 1:93 ¶ 93, Economy, maternal capacity of reading and understanding. Our mothers a… in interview analysis / 1:94 ¶ 94, Uneducated, inability to read, electricity, Mothers may not want to us… in interview analysis / 1:96 ¶ 96, May not have phone because of economy i.e. every mother may not phone… in interview analysis / 1:97 ¶ 97, Mother lost existing phone within the service period, broken Phone whi… in interview analysis / 1:106 ¶ 106, All of them are solvable but may need time to solve in interview analysis / 2:29 ¶ 44, Infants are not owners of the service but with family only and if fami… in KII / 3:28 ¶ 17, This service is possible and when mother do have mobile unless another… in Group discussion / 3:53 ¶ 37, Mobile is now part of the basic needs of our community. Mobile service… in Group discussion / 3:68 ¶ 55, Sustainability of the program is the concern, who will always look aft… in Group discussion

1:93 ¶ 93 in interview analysis

Text quotation

Created by Girma Gilano on 3/9/2023

Economy, maternal capacity of reading and understanding. Our mothers are living in rural and further farm areas where they may not have charged mobile.

2 Codes:

- **Challenges of mHealth impleentation: Devices_handling**

Comment: by Girma Gilano

3/12/2023 12:56:31 PM, merged with Security 3/12/2023 12:56:54 PM, merged with Readiness

12 Quotations:

1:54 ¶ 59, But no all mothers have mobiles. Some time they may loss their mobile… in interview analysis / 1:58 ¶ 63, This can improve the service but not all women have phone, many mother… in interview analysis / 1:63 ¶ 66, I can say it can be effective, but not all mother have mobiles, mobile… in interview analysis / 1:93 ¶ 93, Economy, maternal capacity of reading and understanding. Our mothers a… in interview analysis / 1:94 ¶ 94, Uneducated, inability to read, electricity, Mothers may not want to us… in interview analysis / 1:96 ¶ 96, May not have phone because of economy i.e. every mother may not phone… in interview analysis / 1:97 ¶ 97, Mother lost existing phone within the service period, broken Phone whi… in interview analysis / 1:106 ¶ 106, All of them are solvable but may need time to solve in interview analysis / 2:29 ¶ 44, Infants are not owners of the service but with family only and if fami… in KII / 3:28 ¶ 17, This service is possible and when mother do have mobile unless another… in Group discussion / 3:53 ¶ 37, Mobile is now part of the basic needs of our community. Mobile service… in Group discussion / 3:68 ¶ 55, Sustainability of the program is the concern, who will always look aft… in Group discussion

- **Challenges of mHealth impleentation: Readiness**

Comment: by Girma Gilano

3/12/2023 12:56:31 PM, merged with Security 3/12/2023 12:56:54 PM, merged with Readiness 3/19/2023 2:04:55 AM, merged with Challenges of mHealth impleentation: Resources 3/12/2023 12:56:31 PM, merged with Security 3/12/2023 12:56:54 PM, merged with Readiness

15 Quotations:

1:79 ¶ 78, There could be a little challenge for this because the service is new in interview analysis / 1:85 ¶ 85, But we have to stress continuity and ownership, otherwise when there i… in interview analysis / 1:93 ¶ 93, Economy, maternal capacity of reading and understanding. Our mothers a… in interview analysis / 1:94 ¶ 94, Uneducated, inability to read, electricity, Mothers may not want to us… in interview analysis / 1:106 ¶ 106, All of them are solvable but may need time to solve in interview analysis / 2:1 ¶ 9, If anything necessary fulfilled, it is possible to implement. in KII / 2:18 ¶ 31, Yes, it will be difficult without infrastructure, it will be completel… in KII / 2:21 ¶ 34, This health facility is little bit less organized in materials compute… in KII / 2:22 ¶ 35, I think rural health institutions have a better maternal and child hea… in KII / 2:40 ¶ 60, I have to full resources to start this service. Awareness to health pr… in KII / 2:42 ¶ 63, Fulfilling infrastructures and giving trainings or creating awareness… in KII / 3:1 ¶ 3, May be it is more fit to towns and less likely for rural because of di… in Group discussion / 3:53 ¶ 37, Mobile is now part of the basic needs of our community. Mobile service… in Group discussion / 3:68 ¶ 55, Sustainability of the program is the concern, who will always look aft… in Group discussion / 3:70 ¶ 58, Resource to maintain the service. Interruption of supports, government… in Group discussion

1:94 ¶ 94 in interview analysis

Text quotation

Created by Girma Gilano on 3/9/2023

Uneducated, inability to read, electricity, Mothers may not want to use mobile phone

2 Codes:

- **Challenges of mHealth impleentation: Devices_handling**

Comment: by Girma Gilano

3/12/2023 12:56:31 PM, merged with Security 3/12/2023 12:56:54 PM, merged with Readiness

12 Quotations:

1:54 ¶ 59, But no all mothers have mobiles. Some time they may loss their mobile… in interview analysis / 1:58 ¶ 63, This can improve the service but not all women have phone, many mother… in interview analysis / 1:63 ¶ 66, I can say it can be effective, but not all mother have mobiles, mobile… in interview analysis / 1:93 ¶ 93, Economy, maternal capacity of reading and understanding. Our mothers a… in interview analysis / 1:94 ¶ 94, Uneducated, inability to read, electricity, Mothers may not want to us… in interview analysis / 1:96 ¶ 96, May not have phone because of economy i.e. every mother may not phone… in interview analysis / 1:97 ¶ 97, Mother lost existing phone within the service period, broken Phone whi… in interview analysis / 1:106 ¶ 106, All of them are solvable but may need time to solve in interview analysis / 2:29 ¶ 44, Infants are not owners of the service but with family only and if fami… in KII / 3:28 ¶ 17, This service is possible and when mother do have mobile unless another… in Group discussion / 3:53 ¶ 37, Mobile is now part of the basic needs of our community. Mobile service… in Group discussion / 3:68 ¶ 55, Sustainability of the program is the concern, who will always look aft… in Group discussion

- **Challenges of mHealth impleentation: Readiness**

Comment: by Girma Gilano

3/12/2023 12:56:31 PM, merged with Security 3/12/2023 12:56:54 PM, merged with Readiness 3/19/2023 2:04:55 AM, merged with Challenges of mHealth impleentation: Resources 3/12/2023 12:56:31 PM, merged with Security 3/12/2023 12:56:54 PM, merged with Readiness

15 Quotations:

1:79 ¶ 78, There could be a little challenge for this because the service is new in interview analysis / 1:85 ¶ 85, But we have to stress continuity and ownership, otherwise when there i… in interview analysis / 1:93 ¶ 93, Economy, maternal capacity of reading and understanding. Our mothers a… in interview analysis / 1:94 ¶ 94, Uneducated, inability to read, electricity, Mothers may not want to us… in interview analysis / 1:106 ¶ 106, All of them are solvable but may need time to solve in interview analysis / 2:1 ¶ 9, If anything necessary fulfilled, it is possible to implement. in KII / 2:18 ¶ 31, Yes, it will be difficult without infrastructure, it will be completel… in KII / 2:21 ¶ 34, This health facility is little bit less organized in materials compute… in KII / 2:22 ¶ 35, I think rural health institutions have a better maternal and child hea… in KII / 2:40 ¶ 60, I have to full resources to start this service. Awareness to health pr… in KII / 2:42 ¶ 63, Fulfilling infrastructures and giving trainings or creating awareness… in KII / 3:1 ¶ 3, May be it is more fit to towns and less likely for rural because of di… in Group discussion / 3:53 ¶ 37, Mobile is now part of the basic needs of our community. Mobile service… in Group discussion / 3:68 ¶ 55, Sustainability of the program is the concern, who will always look aft… in Group discussion / 3:70 ¶ 58, Resource to maintain the service. Interruption of supports, government… in Group discussion

1:96 ¶ 96 in interview analysis

Text quotation

Created by Girma Gilano on 3/9/2023

May not have phone because of economy i.e. every mother may not phone and less familiarity with the devices

1 Codes:

- **Challenges of mHealth impleentation: Devices_handling**

Comment: by Girma Gilano

> *3/12/2023 12:56:31 PM, merged with Security 3/12/2023 12:56:54 PM, merged with Readiness*

12 Quotations:

1:54 ¶ 59, But no all mothers have mobiles. Some time they may loss their mobile… in interview analysis / 1:58 ¶ 63, This can improve the service but not all women have phone, many mother… in interview analysis / 1:63 ¶ 66, I can say it can be effective, but not all mother have mobiles, mobile… in interview analysis / 1:93 ¶ 93, Economy, maternal capacity of reading and understanding. Our mothers a… in interview analysis / 1:94 ¶ 94, Uneducated, inability to read, electricity, Mothers may not want to us… in interview analysis / 1:96 ¶ 96, May not have phone because of economy i.e. every mother may not phone… in interview analysis / 1:97 ¶ 97, Mother lost existing phone within the service period, broken Phone whi… in interview analysis / 1:106 ¶ 106, All of them are solvable but may need time to solve in interview analysis / 2:29 ¶ 44, Infants are not owners of the service but with family only and if fami… in KII / 3:28 ¶ 17, This service is possible and when mother do have mobile unless another… in Group discussion / 3:53 ¶ 37, Mobile is now part of the basic needs of our community. Mobile service… in Group discussion / 3:68 ¶ 55, Sustainability of the program is the concern, who will always look aft… in Group discussion

1:97 ¶ 97 in interview analysis

Text quotation

Created by Girma Gilano on 3/9/2023

Mother lost existing phone within the service period, broken Phone which cannot allow mother read or access, and current market to get new mobile phone

1 Codes:

- **Challenges of mHealth impleentation: Devices_handling**

Comment: by Girma Gilano

> *3/12/2023 12:56:31 PM, merged with Security 3/12/2023 12:56:54 PM, merged with Readiness*

12 Quotations:

1:54 ¶ 59, But no all mothers have mobiles. Some time they may loss their mobile… in interview analysis / 1:58 ¶ 63, This can improve the service but not all women have phone, many mother… in interview analysis / 1:63 ¶ 66, I can say it can be effective, but not all mother have mobiles, mobile… in interview analysis / 1:93 ¶ 93, Economy, maternal capacity of reading and understanding. Our mothers a… in interview analysis / 1:94 ¶ 94, Uneducated, inability to read, electricity, Mothers may not want to us… in interview analysis / 1:96 ¶ 96, May not have phone because of economy i.e. every mother may not phone… in interview analysis / 1:97 ¶ 97, Mother lost existing phone within the service period, broken Phone whi… in interview analysis / 1:106 ¶ 106, All of them are solvable but may need time to solve in interview analysis / 2:29 ¶ 44, Infants are not owners of the service but with family only and if fami… in KII / 3:28 ¶ 17, This service is possible and when mother do have mobile unless another… in Group discussion / 3:53 ¶ 37, Mobile is now part of the basic needs of our community. Mobile service… in Group discussion / 3:68 ¶ 55, Sustainability of the program is the concern, who will always look aft… in Group discussion

1:106 ¶ 106 in interview analysis

Text quotation

Created by Girma Gilano on 3/9/2023

All of them are solvable but may need time to solve

3 Codes:

● Challenges of mHealth impleentation: Acceptance

Comment: by Girma Gilano

> *3/12/2023 12:56:31 PM, merged with Security 3/12/2023 12:56:54 PM, merged with Readiness*

17 Quotations:

1:18 ¶ 30, I think it will improve if someone get information from her phone, she… in interview analysis / 1:35 ¶ 39, Sometimes people may think mobile make women rude in interview analysis / 1:37 ¶ 40, t, when women take messages husband may think she is meeting someone.… in interview analysis / 1:84 ¶ 84, ometime our community may not say pregnancy until the conception reac… in interview analysis / 1:102 ¶ 101, Reading is one of the difficulties. May be absence of electricity, in… in interview analysis / 1:106 ¶ 106, All of them are solvable but may need time to solve in interview analysis / 2:8 ¶ 18, The practice will make better because our community need more such ser… in KII / 2:25 ¶ 40, Some projects phase out without making any impact in KII / 2:26 ¶ 41, Rejection by government/politics. Everyone is not modernized, but for… in KII / 2:27 ¶ 42, Competition between groups in KII / 2:28 ¶ 43, Message understanding of the mothers and questions related to why mHea… in KII / 2:33 ¶ 51, In my view, husbands my think different if some for example calling ev… in KII / 2:43 ¶ 61, Preparing place for installation, computers and mobiles are necessary,… in KII / 3:11 ¶ 8, Sometimes people may leave support and the program may fail but if str… in Group discussion / 3:44 ¶ 28, It is a reborn for health system, we hope more technology will come to… in Group discussion / 3:51 ¶ 35, No unique culture, it is all similar. In our culture I think people li… in Group discussion / 3:54 ¶ 38, : No cultural influence because our respect whatever health profession… in Group discussion

● Challenges of mHealth impleentation: Devices_handling

Comment: by Girma Gilano

> *3/12/2023 12:56:31 PM, merged with Security 3/12/2023 12:56:54 PM, merged with Readiness*

12 Quotations:

1:54 ¶ 59, But no all mothers have mobiles. Some time they may loss their mobile… in interview analysis / 1:58 ¶ 63, This can improve the service but not all women have phone, many mother… in interview analysis / 1:63 ¶ 66, I can say it can be effective, but not all mother have mobiles, mobile… in interview analysis / 1:93 ¶ 93, Economy, maternal capacity of reading and understanding. Our mothers a… in interview analysis / 1:94 ¶ 94, Uneducated, inability to read, electricity, Mothers may not want to us… in interview analysis / 1:96 ¶ 96, May not have phone because of economy i.e. every mother may not phone… in interview analysis / 1:97 ¶ 97, Mother lost existing phone within the service period, broken Phone whi… in interview analysis / 1:106 ¶ 106, All of them are solvable but may need time to solve in interview analysis / 2:29 ¶ 44, Infants are not owners of the service but with family only and if fami… in KII / 3:28 ¶ 17, This service is possible and when mother do have mobile unless another… in Group discussion / 3:53 ¶ 37, Mobile is now part of the basic needs of our community. Mobile service… in Group discussion / 3:68 ¶ 55, Sustainability of the program is the concern, who will always look aft… in Group discussion

● Challenges of mHealth impleentation: Readiness

Comment: by Girma Gilano

> *3/12/2023 12:56:31 PM, merged with Security 3/12/2023 12:56:54 PM, merged with Readiness 3/19/2023 2:04:55 AM, merged with Challenges of mHealth impleentation: Resources 3/12/2023 12:56:31 PM, merged with Security 3/12/2023 12:56:54 PM, merged with Readiness*

15 Quotations:

1:79 ¶ 78, There could be a little challenge for this because the service is new in interview analysis / 1:85 ¶ 85, But we have to stress continuity and ownership, otherwise when there i… in interview analysis / 1:93 ¶ 93, Economy, maternal capacity of reading and understanding. Our mothers a… in interview analysis / 1:94 ¶ 94, Uneducated, inability to read, electricity, Mothers may not want to us… in interview analysis / 1:106 ¶ 106, All of them are solvable but may need time to solve in interview analysis / 2:1 ¶ 9, If anything necessary fulfilled, it is possible to implement. in KII / 2:18 ¶ 31, Yes, it will be difficult without infrastructure, it will be completel… in KII / 2:21 ¶ 34, This health facility is little bit less organized in materials compute… in KII / 2:22 ¶ 35, I think rural health institutions have a better maternal and child hea… in KII / 2:40 ¶ 60, I have to full resources to start this service. Awareness to health pr… in KII / 2:42 ¶ 63, Fulfilling infrastructures and giving trainings or creating awareness… in KII / 3:1 ¶ 3, May be it is more fit to towns and less likely for rural because of di… in Group discussion / 3:53 ¶ 37, Mobile is now part of the basic needs of our community. Mobile service… in Group discussion / 3:68 ¶ 55, Sustainability of the program is the concern, who will always look aft… in Group discussion / 3:70 ¶ 58, Resource to maintain the service. Interruption of supports, government… in Group discussion

2:29 ¶ 44 in KII

Text quotation

Created by Girma Gilano on 3/9/2023

Infants are not owners of the service but with family only and if family have not capacity to use due to different reason, for instance, everyone may not use mobile

1 Codes:

- **Challenges of mHealth impleentation: Devices_handling**

Comment: by Girma Gilano

> *3/12/2023 12:56:31 PM, merged with Security 3/12/2023 12:56:54 PM, merged with Readiness*

12 Quotations:

1:54 ¶ 59, But no all mothers have mobiles. Some time they may loss their mobile… in interview analysis / 1:58 ¶ 63, This can improve the service but not all women have phone, many mother… in interview analysis / 1:63 ¶ 66, I can say it can be effective, but not all mother have mobiles, mobile… in interview analysis / 1:93 ¶ 93, Economy, maternal capacity of reading and understanding. Our mothers a… in interview analysis / 1:94 ¶ 94, Uneducated, inability to read, electricity, Mothers may not want to us… in interview analysis / 1:96 ¶ 96, May not have phone because of economy i.e. every mother may not phone… in interview analysis / 1:97 ¶ 97, Mother lost existing phone within the service period, broken Phone whi… in interview analysis / 1:106 ¶ 106, All of them are solvable but may need time to solve in interview analysis / 2:29 ¶ 44, Infants are not owners of the service but with family only and if fami… in KII / 3:28 ¶ 17, This service is possible and when mother do have mobile unless another… in Group discussion / 3:53 ¶ 37, Mobile is now part of the basic needs of our community. Mobile service… in Group discussion / 3:68 ¶ 55, Sustainability of the program is the concern, who will always look aft… in Group discussion

3:28 ¶ 17 in Group discussion

Text quotation

Created by Girma Gilano on 3/9/2023

This service is possible and when mother do have mobile unless another option is availabl

1 Codes:

- **Challenges of mHealth impleentation: Devices_handling**

  Comment: by Girma Gilano

  > *3/12/2023 12:56:31 PM, merged with Security 3/12/2023 12:56:54 PM, merged with Readiness*

  ### 12 Quotations:

  1:54 ¶ 59, But no all mothers have mobiles. Some time they may loss their mobile… in interview analysis / 1:58 ¶ 63, This can improve the service but not all women have phone, many mother… in interview analysis / 1:63 ¶ 66, I can say it can be effective, but not all mother have mobiles, mobile… in interview analysis / 1:93 ¶ 93, Economy, maternal capacity of reading and understanding. Our mothers a… in interview analysis / 1:94 ¶ 94, Uneducated, inability to read, electricity, Mothers may not want to us… in interview analysis / 1:96 ¶ 96, May not have phone because of economy i.e. every mother may not phone… in interview analysis / 1:97 ¶ 97, Mother lost existing phone within the service period, broken Phone whi… in interview analysis / 1:106 ¶ 106, All of them are solvable but may need time to solve in interview analysis / 2:29 ¶ 44, Infants are not owners of the service but with family only and if fami… in KII / 3:28 ¶ 17, This service is possible and when mother do have mobile unless another… in Group discussion / 3:53 ¶ 37, Mobile is now part of the basic needs of our community. Mobile service… in Group discussion / 3:68 ¶ 55, Sustainability of the program is the concern, who will always look aft… in Group discussion

3:53 ¶ 37 in Group discussion

Text quotation

Created by Girma Gilano on 3/9/2023

Mobile is now part of the basic needs of our community. Mobile services are the most used thing in the community, so what possible culture could avoid using mHealth? Exactly there is no culture

2 Codes:

- **Challenges of mHealth impleentation: Devices_handling**

  Comment: by Girma Gilano

  > *3/12/2023 12:56:31 PM, merged with Security 3/12/2023 12:56:54 PM, merged with Readiness*

  ### 12 Quotations:

  1:54 ¶ 59, But no all mothers have mobiles. Some time they may loss their mobile… in interview analysis / 1:58 ¶ 63, This can improve the service but not all women have phone, many mother… in interview analysis / 1:63 ¶ 66, I can say it can be effective, but not all mother have mobiles, mobile… in interview analysis / 1:93 ¶ 93, Economy, maternal capacity of reading and understanding. Our mothers a… in interview analysis / 1:94 ¶ 94, Uneducated, inability to read, electricity, Mothers may not want to us… in

interview analysis / 1:96 ¶ 96, May not have phone because of economy i.e. every mother may not phone… in interview analysis / 1:97 ¶ 97, Mother lost existing phone within the service period, broken Phone whi… in interview analysis / 1:106 ¶ 106, All of them are solvable but may need time to solve in interview analysis / 2:29 ¶ 44, Infants are not owners of the service but with family only and if fami… in KII / 3:28 ¶ 17, This service is possible and when mother do have mobile unless another… in Group discussion / 3:53 ¶ 37, Mobile is now part of the basic needs of our community. Mobile service… in Group discussion / 3:68 ¶ 55, Sustainability of the program is the concern, who will always look aft… in Group discussion

- **Challenges of mHealth impleentation: Readiness**

Comment: by Girma Gilano

> *3/12/2023 12:56:31 PM, merged with Security 3/12/2023 12:56:54 PM, merged with Readiness 3/19/2023 2:04:55 AM, merged with Challenges of mHealth impleentation: Resources 3/12/2023 12:56:31 PM, merged with Security 3/12/2023 12:56:54 PM, merged with Readiness*

15 Quotations:

1:79 ¶ 78, There could be a little challenge for this because the service is new in interview analysis / 1:85 ¶ 85, But we have to stress continuity and ownership, otherwise when there i… in interview analysis / 1:93 ¶ 93, Economy, maternal capacity of reading and understanding. Our mothers a… in interview analysis / 1:94 ¶ 94, Uneducated, inability to read, electricity, Mothers may not want to us… in interview analysis / 1:106 ¶ 106, All of them are solvable but may need time to solve in interview analysis / 2:1 ¶ 9, If anything necessary fulfilled, it is possible to implement. in KII / 2:18 ¶ 31, Yes, it will be difficult without infrastructure, it will be completel… in KII / 2:21 ¶ 34, This health facility is little bit less organized in materials compute… in KII / 2:22 ¶ 35, I think rural health institutions have a better maternal and child hea… in KII / 2:40 ¶ 60, I have to full resources to start this service. Awareness to health pr… in KII / 2:42 ¶ 63, Fulfilling infrastructures and giving trainings or creating awareness… in KII / 3:1 ¶ 3, May be it is more fit to towns and less likely for rural because of di… in Group discussion / 3:53 ¶ 37, Mobile is now part of the basic needs of our community. Mobile service… in Group discussion / 3:68 ¶ 55, Sustainability of the program is the concern, who will always look aft… in Group discussion / 3:70 ¶ 58, Resource to maintain the service. Interruption of supports, government… in Group discussion

3:68 ¶ 55 in Group discussion

Text quotation

Created by Girma Gilano on 3/9/2023

Sustainability of the program is the concern, who will always look after computer

2 Codes:

- **Challenges of mHealth impleentation: Devices_handling**

Comment: by Girma Gilano

> *3/12/2023 12:56:31 PM, merged with Security 3/12/2023 12:56:54 PM, merged with Readiness*

12 Quotations:

1:54 ¶ 59, But no all mothers have mobiles. Some time they may loss their mobile… in interview analysis / 1:58 ¶ 63, This can improve the service but not all women have phone, many mother… in interview analysis / 1:63 ¶ 66, I can say it can be effective, but not all mother have mobiles, mobile… in interview analysis / 1:93 ¶ 93, Economy, maternal capacity of reading and understanding. Our mothers a… in interview analysis / 1:94 ¶ 94, Uneducated, inability to read, electricity, Mothers may not want to us… in

interview analysis / 1:96 ¶ 96, May not have phone because of economy i.e. every mother may not phone… in interview analysis / 1:97 ¶ 97, Mother lost existing phone within the service period, broken Phone whi… in interview analysis / 1:106 ¶ 106, All of them are solvable but may need time to solve in interview analysis / 2:29 ¶ 44, Infants are not owners of the service but with family only and if fami… in KII / 3:28 ¶ 17, This service is possible and when mother do have mobile unless another… in Group discussion / 3:53 ¶ 37, Mobile is now part of the basic needs of our community. Mobile service… in Group discussion / 3:68 ¶ 55, Sustainability of the program is the concern, who will always look aft… in Group discussion

● Challenges of mHealth impleentation: Readiness

Comment: by Girma Gilano

> *3/12/2023 12:56:31 PM, merged with Security 3/12/2023 12:56:54 PM, merged with Readiness 3/19/2023 2:04:55 AM, merged with Challenges of mHealth impleentation: Resources 3/12/2023 12:56:31 PM, merged with Security 3/12/2023 12:56:54 PM, merged with Readiness*

15 Quotations:

1:79 ¶ 78, There could be a little challenge for this because the service is new in interview analysis / 1:85 ¶ 85, But we have to stress continuity and ownership, otherwise when there i… in interview analysis / 1:93 ¶ 93, Economy, maternal capacity of reading and understanding. Our mothers a… in interview analysis / 1:94 ¶ 94, Uneducated, inability to read, electricity, Mothers may not want to us… in interview analysis / 1:106 ¶ 106, All of them are solvable but may need time to solve in interview analysis / 2:1 ¶ 9, If anything necessary fulfilled, it is possible to implement. in KII / 2:18 ¶ 31, Yes, it will be difficult without infrastructure, it will be completel… in KII / 2:21 ¶ 34, This health facility is little bit less organized in materials compute… in KII / 2:22 ¶ 35, I think rural health institutions have a better maternal and child hea… in KII / 2:40 ¶ 60, I have to full resources to start this service. Awareness to health pr… in KII / 2:42 ¶ 63, Fulfilling infrastructures and giving trainings or creating awareness… in KII / 3:1 ¶ 3, May be it is more fit to towns and less likely for rural because of di… in Group discussion / 3:53 ¶ 37, Mobile is now part of the basic needs of our community. Mobile service… in Group discussion / 3:68 ¶ 55, Sustainability of the program is the concern, who will always look aft… in Group discussion / 3:70 ¶ 58, Resource to maintain the service. Interruption of supports, government… in Group discussion

---

● Challenges of mHealth impleentation: negligence

Created by Girma Gilano on 3/12/2023

Comment: by Girma Gilano

> *3/12/2023 12:56:31 PM, merged with Security 3/12/2023 12:56:54 PM, merged with Readiness*

7 Quotations:

1:3 ¶ 19 in interview analysis

Text quotation

Created by Girma Gilano on 3/8/2023

but some are negligent, and mothers knew the consequence of not complying

1 Codes:

- **Challenges of mHealth impleentation: negligence**

  Comment: by Girma Gilano

  > *3/12/2023 12:56:31 PM, merged with Security 3/12/2023 12:56:54 PM, merged with Readiness*

  7 Quotations:

  1:3 ¶ 19, but some are negligent, and mothers knew the consequence of not comply… in interview analysis / 1:7 ¶ 22, Normally, the mother come to health institution at 45 days but some st… in interview analysis / 1:72 ¶ 74, I don’t think there will be a problem unless the carelessness of profe… in interview analysis / 1:95 ¶ 95, Because of this service is new some acceptance can be happen until eve… in interview analysis / 3:15 ¶ 10, I think it will be more helpful for rural mothers, for towns every mot… in Group discussion / 3:65 ¶ 52, Reluctance, being bored of many messages over time, .pre occupation wi… in Group discussion / 3:66 ¶ 53, Previous ignorance nature for services in Group discussion

1:7 ¶ 22 in interview analysis

Text quotation

Created by Girma Gilano on 3/8/2023

Normally, the mother come to health institution at 45 days but some still negligent or forget the date or not aware of the date and do not come that is where mHealth can change

1 Codes:

- **Challenges of mHealth impleentation: negligence**

  Comment: by Girma Gilano

  > *3/12/2023 12:56:31 PM, merged with Security 3/12/2023 12:56:54 PM, merged with Readiness*

  7 Quotations:

  1:3 ¶ 19, but some are negligent, and mothers knew the consequence of not comply… in interview analysis / 1:7 ¶ 22, Normally, the mother come to health institution at 45 days but some st… in interview analysis / 1:72 ¶ 74, I don’t think there will be a problem unless the carelessness of profe… in interview analysis / 1:95 ¶ 95, Because of this service is new some acceptance can be happen until eve… in interview analysis / 3:15 ¶ 10, I think it will be more helpful for rural mothers, for towns every mot… in Group discussion / 3:65 ¶ 52, Reluctance, being bored of many messages over time, .pre occupation wi… in Group discussion / 3:66 ¶ 53, Previous ignorance nature for services in Group discussion

1:72 ¶ 74 in interview analysis

Text quotation

Created by Girma Gilano on 3/8/2023

I don’t think there will be a problem unless the carelessness of professionals affected those mothers acceptance; they might say is there anything like that?

1 Codes:

- **Challenges of mHealth impleentation: negligence**

  Comment: by Girma Gilano

  > *3/12/2023 12:56:31 PM, merged with Security 3/12/2023 12:56:54 PM, merged with Readiness*

  ### 7 Quotations:

  1:3 ¶ 19, but some are negligent, and mothers knew the consequence of not comply… in interview analysis / 1:7 ¶ 22, Normally, the mother come to health institution at 45 days but some st… in interview analysis / 1:72 ¶ 74, I don't think there will be a problem unless the carelessness of profe… in interview analysis / 1:95 ¶ 95, Because of this service is new some acceptance can be happen until eve… in interview analysis / 3:15 ¶ 10, I think it will be more helpful for rural mothers, for towns every mot… in Group discussion / 3:65 ¶ 52, Reluctance, being bored of many messages over time, .pre occupation wi… in Group discussion / 3:66 ¶ 53, Previous ignorance nature for services in Group discussion

1:95 ¶ 95 in interview analysis

Text quotation

Created by Girma Gilano on 3/9/2023

Because of this service is new some acceptance can be happen until everyone understand that awareness is necessary because every new service need some effort to aware the users and the potential users, meaning the whole community should get awareness on this issue

1 Codes:

- **Challenges of mHealth impleentation: negligence**

  Comment: by Girma Gilano

  > *3/12/2023 12:56:31 PM, merged with Security 3/12/2023 12:56:54 PM, merged with Readiness*

  ### 7 Quotations:

  1:3 ¶ 19, but some are negligent, and mothers knew the consequence of not comply… in interview analysis / 1:7 ¶ 22, Normally, the mother come to health institution at 45 days but some st… in interview analysis / 1:72 ¶ 74, I don't think there will be a problem unless the carelessness of profe… in interview analysis / 1:95 ¶ 95, Because of this service is new some acceptance can be happen until eve… in interview analysis / 3:15 ¶ 10, I think it will be more helpful for rural mothers, for towns every mot… in Group discussion / 3:65 ¶ 52, Reluctance, being bored of many messages over time, .pre occupation wi… in Group discussion / 3:66 ¶ 53, Previous ignorance nature for services in Group discussion

3:15 ¶ 10 in Group discussion

Text quotation

Created by Girma Gilano on 3/9/2023

I think it will be more helpful for rural mothers, for towns every mother feed her child early additional food and that look like unchangeable

1 Codes:

- ### Challenges of mHealth impleentation: negligence

Comment: by Girma Gilano

> *3/12/2023 12:56:31 PM, merged with Security 3/12/2023 12:56:54 PM, merged with Readiness*

7 Quotations:

1:3 ¶ 19, but some are negligent, and mothers knew the consequence of not comply… in interview analysis / 1:7 ¶ 22, Normally, the mother come to health institution at 45 days but some st… in interview analysis / 1:72 ¶ 74, I don't think there will be a problem unless the carelessness of profe… in interview analysis / 1:95 ¶ 95, Because of this service is new some acceptance can be happen until eve… in interview analysis / 3:15 ¶ 10, I think it will be more helpful for rural mothers, for towns every mot… in Group discussion / 3:65 ¶ 52, Reluctance, being bored of many messages over time, .pre occupation wi… in Group discussion / 3:66 ¶ 53, Previous ignorance nature for services in Group discussion

3:65 ¶ 52 in Group discussion

Text quotation

Created by Girma Gilano on 3/9/2023

Reluctance, being bored of many messages over time, .pre occupation with important thing, hand-to-mouth life style, and daily laborer work

2 Codes:

- ### Challenges of mHealth impleentation: negligence

Comment: by Girma Gilano

> *3/12/2023 12:56:31 PM, merged with Security 3/12/2023 12:56:54 PM, merged with Readiness*

7 Quotations:

1:3 ¶ 19, but some are negligent, and mothers knew the consequence of not comply… in interview analysis / 1:7 ¶ 22, Normally, the mother come to health institution at 45 days but some st… in interview analysis / 1:72 ¶ 74, I don't think there will be a problem unless the carelessness of profe… in interview analysis / 1:95 ¶ 95, Because of this service is new some acceptance can be happen until eve… in interview analysis / 3:15 ¶ 10, I think it will be more helpful for rural mothers, for towns every mot… in Group discussion / 3:65 ¶ 52, Reluctance, being bored of many messages over time, .pre occupation wi… in Group discussion / 3:66 ¶ 53, Previous ignorance nature for services in Group discussion

- ### Challenges of mHealth impleentation: workload

Comment: by Girma Gilano

> *3/12/2023 12:56:31 PM, merged with Security 3/12/2023 12:56:54 PM, merged with Readiness*

7 Quotations:

1:2 ¶ 18, By the way mother knew their appointment but it is just the load of wo… in interview analysis / 1:16 ¶ 29, Usually mothers know their date but since they are daily laborer, they… in interview analysis /

1:17 ¶ 30, Some mothers are the heavy workers and cannot avoid missing the appoin… in interview analysis / 1:31 ¶ 38, this will increase pressure on professionals in interview analysis / 3:3 ¶ 4, I appreciate to have technology assistance, but mothers do not forget… in Group discussion / 3:4 ¶ 4, Daily laborers usually do not have time for themselves because of the… in Group discussion / 3:65 ¶ 52, Reluctance, being bored of many messages over time, .pre occupation wi… in Group discussion

3:66 ¶ 53 in Group discussion

Text quotation

Created by Girma Gilano on 3/9/2023

Previous ignorance nature for services

1 Codes:

- **Challenges of mHealth impleentation: negligence**

Comment: by Girma Gilano

> *3/12/2023 12:56:31 PM, merged with Security 3/12/2023 12:56:54 PM, merged with Readiness*

7 Quotations:

1:3 ¶ 19, but some are negligent, and mothers knew the consequence of not comply… in interview analysis / 1:7 ¶ 22, Normally, the mother come to health institution at 45 days but some st… in interview analysis / 1:72 ¶ 74, I don't think there will be a problem unless the carelessness of profe… in interview analysis / 1:95 ¶ 95, Because of this service is new some acceptance can be happen until eve… in interview analysis / 3:15 ¶ 10, I think it will be more helpful for rural mothers, for towns every mot… in Group discussion / 3:65 ¶ 52, Reluctance, being bored of many messages over time, .pre occupation wi… in Group discussion / 3:66 ¶ 53, Previous ignorance nature for services in Group discussion

---

● Challenges of mHealth impleentation: Readiness

Created by Girma Gilano on 3/12/2023, **modified** by Girma Gilano on 3/19/2023

Comment: by Girma Gilano

> *3/12/2023 12:56:31 PM, merged with Security 3/12/2023 12:56:54 PM, merged with Readiness 3/19/2023 2:04:55 AM, merged with Challenges of mHealth impleentation: Resources 3/12/2023 12:56:31 PM, merged with Security 3/12/2023 12:56:54 PM, merged with Readiness*

15 Quotations:

1:79 ¶ 78 in interview analysis

Text quotation

Created by Girma Gilano on 3/8/2023

There could be a little challenge for this because the service is new

1 Codes:

● Challenges of mHealth impleentation: Readiness

Comment: by Girma Gilano

> *3/12/2023 12:56:31 PM, merged with Security 3/12/2023 12:56:54 PM, merged with Readiness 3/19/2023 2:04:55 AM, merged with Challenges of mHealth impleentation: Resources 3/12/2023 12:56:31 PM, merged with Security 3/12/2023 12:56:54 PM, merged with Readiness*

15 Quotations:

1:79 ¶ 78, There could be a little challenge for this because the service is new in interview analysis / 1:85 ¶ 85, But we have to stress continuity and ownership, otherwise when there i… in interview analysis / 1:93 ¶ 93, Economy, maternal capacity of reading and understanding. Our mothers a… in interview analysis / 1:94 ¶ 94, Uneducated, inability to read, electricity, Mothers may not want to us… in interview analysis / 1:106 ¶ 106, All of them are solvable but may need time to solve in interview analysis / 2:1 ¶ 9, If anything necessary fulfilled, it is possible to implement. in KII / 2:18 ¶ 31, Yes, it will be difficult without infrastructure, it will be completel… in KII / 2:21 ¶ 34, This health facility is little bit less organized in materials compute… in KII / 2:22 ¶ 35, I think rural health institutions have a better maternal and child hea… in KII / 2:40 ¶ 60, I have to full resources to start this service. Awareness to health pr… in KII / 2:42 ¶ 63, Fulfilling infrastructures and giving trainings or creating awareness… in KII / 3:1 ¶ 3, May be it is more fit to towns and less likely for rural because of di… in Group discussion / 3:53 ¶ 37, Mobile is now part of the basic needs of our community. Mobile service… in Group discussion / 3:68 ¶ 55, Sustainability of the program is the concern, who will always look aft… in Group discussion / 3:70 ¶ 58, Resource to maintain the service. Interruption of supports, government… in Group discussion

1:85 ¶ 85 in interview analysis

Text quotation

Created by Girma Gilano on 3/9/2023

But we have to stress continuity and ownership, otherwise when there is an interruption mother may lose hope and distribute the information

1 Codes:

● Challenges of mHealth impleentation: Readiness

Comment: by Girma Gilano

> *3/12/2023 12:56:31 PM, merged with Security 3/12/2023 12:56:54 PM, merged with Readiness 3/19/2023 2:04:55 AM, merged with Challenges of mHealth impleentation: Resources 3/12/2023 12:56:31 PM, merged with Security 3/12/2023 12:56:54 PM, merged with Readiness*

15 Quotations:

1:79 ¶ 78, There could be a little challenge for this because the service is new in interview analysis / 1:85 ¶ 85, But we have to stress continuity and ownership, otherwise when there i… in interview analysis / 1:93 ¶ 93, Economy, maternal capacity of reading and understanding. Our mothers a… in interview analysis / 1:94 ¶ 94, Uneducated, inability to read, electricity, Mothers may not want to us… in interview analysis / 1:106 ¶ 106, All of them are solvable but may need time to solve in interview analysis / 2:1 ¶ 9, If anything necessary fulfilled, it is possible to implement. in KII / 2:18 ¶ 31, Yes, it will be difficult without infrastructure, it will be completel… in KII / 2:21 ¶ 34, This health facility is little bit less organized in

materials compute… in KII / 2:22 ¶ 35, I think rural health institutions have a better maternal and child hea… in KII / 2:40 ¶ 60, I have to full resources to start this service. Awareness to health pr… in KII / 2:42 ¶ 63, Fulfilling infrastructures and giving trainings or creating awareness… in KII / 3:1 ¶ 3, May be it is more fit to towns and less likely for rural because of di… in Group discussion / 3:53 ¶ 37, Mobile is now part of the basic needs of our community. Mobile service… in Group discussion / 3:68 ¶ 55, Sustainability of the program is the concern, who will always look aft… in Group discussion / 3:70 ¶ 58, Resource to maintain the service. Interruption of supports, government… in Group discussion

1:93 ¶ 93 in interview analysis

Text quotation

Created by Girma Gilano on 3/9/2023

Economy, maternal capacity of reading and understanding. Our mothers are living in rural and further farm areas where they may not have charged mobile.

2 Codes:

- **Challenges of mHealth impleentation: Devices_handling**

Comment: by Girma Gilano

> *3/12/2023 12:56:31 PM, merged with Security 3/12/2023 12:56:54 PM, merged with Readiness*

12 Quotations:

1:54 ¶ 59, But no all mothers have mobiles. Some time they may loss their mobile… in interview analysis / 1:58 ¶ 63, This can improve the service but not all women have phone, many mother… in interview analysis / 1:63 ¶ 66, I can say it can be effective, but not all mother have mobiles, mobile… in interview analysis / 1:93 ¶ 93, Economy, maternal capacity of reading and understanding. Our mothers a… in interview analysis / 1:94 ¶ 94, Uneducated, inability to read, electricity, Mothers may not want to us… in interview analysis / 1:96 ¶ 96, May not have phone because of economy i.e. every mother may not phone… in interview analysis / 1:97 ¶ 97, Mother lost existing phone within the service period, broken Phone whi… in interview analysis / 1:106 ¶ 106, All of them are solvable but may need time to solve in interview analysis / 2:29 ¶ 44, Infants are not owners of the service but with family only and if fami… in KII / 3:28 ¶ 17, This service is possible and when mother do have mobile unless another… in Group discussion / 3:53 ¶ 37, Mobile is now part of the basic needs of our community. Mobile service… in Group discussion / 3:68 ¶ 55, Sustainability of the program is the concern, who will always look aft… in Group discussion

- **Challenges of mHealth impleentation: Readiness**

Comment: by Girma Gilano

> *3/12/2023 12:56:31 PM, merged with Security 3/12/2023 12:56:54 PM, merged with Readiness 3/19/2023 2:04:55 AM, merged with Challenges of mHealth impleentation: Resources 3/12/2023 12:56:31 PM, merged with Security 3/12/2023 12:56:54 PM, merged with Readiness*

15 Quotations:

1:79 ¶ 78, There could be a little challenge for this because the service is new in interview analysis / 1:85 ¶ 85, But we have to stress continuity and ownership, otherwise when there i… in interview analysis / 1:93 ¶ 93, Economy, maternal capacity of reading and understanding. Our mothers a… in interview analysis / 1:94 ¶ 94, Uneducated, inability to read, electricity, Mothers may not want to us… in interview analysis / 1:106 ¶ 106, All of them are solvable but may need time to solve in interview analysis / 2:1 ¶ 9,

If anything necessary fulfilled, it is possible to implement. in KII / 2:18 ¶ 31, Yes, it will be difficult without infrastructure, it will be completel… in KII / 2:21 ¶ 34, This health facility is little bit less organized in materials compute… in KII / 2:22 ¶ 35, I think rural health institutions have a better maternal and child hea… in KII / 2:40 ¶ 60, I have to full resources to start this service. Awareness to health pr… in KII / 2:42 ¶ 63, Fulfilling infrastructures and giving trainings or creating awareness… in KII / 3:1 ¶ 3, May be it is more fit to towns and less likely for rural because of di… in Group discussion / 3:53 ¶ 37, Mobile is now part of the basic needs of our community. Mobile service… in Group discussion / 3:68 ¶ 55, Sustainability of the program is the concern, who will always look aft… in Group discussion / 3:70 ¶ 58, Resource to maintain the service. Interruption of supports, government… in Group discussion

1:94 ¶ 94 in interview analysis

Text quotation

Created by Girma Gilano on 3/9/2023

Uneducated, inability to read, electricity, Mothers may not want to use mobile phone

2 Codes:

- **Challenges of mHealth impleentation: Devices_handling**

Comment: by Girma Gilano

> *3/12/2023 12:56:31 PM, merged with Security 3/12/2023 12:56:54 PM, merged with Readiness*

12 Quotations:

1:54 ¶ 59, But no all mothers have mobiles. Some time they may loss their mobile… in interview analysis / 1:58 ¶ 63, This can improve the service but not all women have phone, many mother… in interview analysis / 1:63 ¶ 66, I can say it can be effective, but not all mother have mobiles, mobile… in interview analysis / 1:93 ¶ 93, Economy, maternal capacity of reading and understanding. Our mothers a… in interview analysis / 1:94 ¶ 94, Uneducated, inability to read, electricity, Mothers may not want to us… in interview analysis / 1:96 ¶ 96, May not have phone because of economy i.e. every mother may not phone… in interview analysis / 1:97 ¶ 97, Mother lost existing phone within the service period, broken Phone whi… in interview analysis / 1:106 ¶ 106, All of them are solvable but may need time to solve in interview analysis / 2:29 ¶ 44, Infants are not owners of the service but with family only and if fami… in KII / 3:28 ¶ 17, This service is possible and when mother do have mobile unless another… in Group discussion / 3:53 ¶ 37, Mobile is now part of the basic needs of our community. Mobile service… in Group discussion / 3:68 ¶ 55, Sustainability of the program is the concern, who will always look aft… in Group discussion

- **Challenges of mHealth impleentation: Readiness**

Comment: by Girma Gilano

> *3/12/2023 12:56:31 PM, merged with Security 3/12/2023 12:56:54 PM, merged with Readiness 3/19/2023 2:04:55 AM, merged with Challenges of mHealth impleentation: Resources 3/12/2023 12:56:31 PM, merged with Security 3/12/2023 12:56:54 PM, merged with Readiness*

15 Quotations:

1:79 ¶ 78, There could be a little challenge for this because the service is new in interview analysis / 1:85 ¶ 85, But we have to stress continuity and ownership, otherwise when there i… in interview analysis / 1:93 ¶ 93, Economy, maternal capacity of reading and understanding. Our mothers a… in interview analysis / 1:94 ¶ 94, Uneducated, inability to read, electricity, Mothers may not want to us… in interview analysis / 1:106 ¶ 106, All of them are solvable but may need time to solve in interview analysis / 2:1 ¶ 9,

If anything necessary fulfilled, it is possible to implement. in KII / 2:18 ¶ 31, Yes, it will be difficult without infrastructure, it will be completel… in KII / 2:21 ¶ 34, This health facility is little bit less organized in materials compute… in KII / 2:22 ¶ 35, I think rural health institutions have a better maternal and child hea… in KII / 2:40 ¶ 60, I have to full resources to start this service. Awareness to health pr… in KII / 2:42 ¶ 63, Fulfilling infrastructures and giving trainings or creating awareness… in KII / 3:1 ¶ 3, May be it is more fit to towns and less likely for rural because of di… in Group discussion / 3:53 ¶ 37, Mobile is now part of the basic needs of our community. Mobile service… in Group discussion / 3:68 ¶ 55, Sustainability of the program is the concern, who will always look aft… in Group discussion / 3:70 ¶ 58, Resource to maintain the service. Interruption of supports, government… in Group discussion

1:106 ¶ 106 in interview analysis

Text quotation

Created by Girma Gilano on 3/9/2023

All of them are solvable but may need time to solve

3 Codes:

● Challenges of mHealth impleentation: Acceptance

Comment: by Girma Gilano

> *3/12/2023 12:56:31 PM, merged with Security 3/12/2023 12:56:54 PM, merged with Readiness*

17 Quotations:

1:18 ¶ 30, I think it will improve if someone get information from her phone, she… in interview analysis / 1:35 ¶ 39, Sometimes people may think mobile make women rude in interview analysis / 1:37 ¶ 40, t, when women take messages husband may think she is meeting someone.… in interview analysis / 1:84 ¶ 84, ometime our community may not say pregnancy until the conception reac… in interview analysis / 1:102 ¶ 101, Reading is one of the difficulties. May be absence of electricity, in… in interview analysis / 1:106 ¶ 106, All of them are solvable but may need time to solve in interview analysis / 2:8 ¶ 18, The practice will make better because our community need more such ser… in KII / 2:25 ¶ 40, Some projects phase out without making any impact in KII / 2:26 ¶ 41, Rejection by government/politics. Everyone is not modernized, but for… in KII / 2:27 ¶ 42, Competition between groups in KII / 2:28 ¶ 43, Message understanding of the mothers and questions related to why mHea… in KII / 2:33 ¶ 51, In my view, husbands my think different if some for example calling ev… in KII / 2:43 ¶ 61, Preparing place for installation, computers and mobiles are necessary,… in KII / 3:11 ¶ 8, Sometimes people may leave support and the program may fail but if str… in Group discussion / 3:44 ¶ 28, It is a reborn for health system, we hope more technology will come to… in Group discussion / 3:51 ¶ 35, No unique culture, it is all similar. In our culture I think people li… in Group discussion / 3:54 ¶ 38, : No cultural influence because our respect whatever health profession… in Group discussion

● Challenges of mHealth impleentation: Devices_handling

Comment: by Girma Gilano

> *3/12/2023 12:56:31 PM, merged with Security 3/12/2023 12:56:54 PM, merged with Readiness*

12 Quotations:

1:54 ¶ 59, But no all mothers have mobiles. Some time they may loss their mobile… in interview analysis / 1:58 ¶ 63, This can improve the service but not all women have phone, many mother… in interview analysis / 1:63 ¶ 66, I can say it can be effective, but not all mother have mobiles, mobile… in interview analysis / 1:93 ¶ 93, Economy, maternal capacity of reading and understanding. Our mothers a… in

interview analysis / 1:94 ¶ 94, Uneducated, inability to read, electricity, Mothers may not want to us… in interview analysis / 1:96 ¶ 96, May not have phone because of economy i.e. every mother may not phone… in interview analysis / 1:97 ¶ 97, Mother lost existing phone within the service period, broken Phone whi… in interview analysis / 1:106 ¶ 106, All of them are solvable but may need time to solve in interview analysis / 2:29 ¶ 44, Infants are not owners of the service but with family only and if fami… in KII / 3:28 ¶ 17, This service is possible and when mother do have mobile unless another… in Group discussion / 3:53 ¶ 37, Mobile is now part of the basic needs of our community. Mobile service… in Group discussion / 3:68 ¶ 55, Sustainability of the program is the concern, who will always look aft… in Group discussion

- **Challenges of mHealth impleentation: Readiness**

Comment: by Girma Gilano

> *3/12/2023 12:56:31 PM, merged with Security 3/12/2023 12:56:54 PM, merged with Readiness 3/19/2023 2:04:55 AM, merged with Challenges of mHealth impleentation: Resources 3/12/2023 12:56:31 PM, merged with Security 3/12/2023 12:56:54 PM, merged with Readiness*

15 Quotations:

1:79 ¶ 78, There could be a little challenge for this because the service is new in interview analysis / 1:85 ¶ 85, But we have to stress continuity and ownership, otherwise when there i… in interview analysis / 1:93 ¶ 93, Economy, maternal capacity of reading and understanding. Our mothers a… in interview analysis / 1:94 ¶ 94, Uneducated, inability to read, electricity, Mothers may not want to us… in interview analysis / 1:106 ¶ 106, All of them are solvable but may need time to solve in interview analysis / 2:1 ¶ 9, If anything necessary fulfilled, it is possible to implement. in KII / 2:18 ¶ 31, Yes, it will be difficult without infrastructure, it will be completel… in KII / 2:21 ¶ 34, This health facility is little bit less organized in materials compute… in KII / 2:22 ¶ 35, I think rural health institutions have a better maternal and child hea… in KII / 2:40 ¶ 60, I have to full resources to start this service. Awareness to health pr… in KII / 2:42 ¶ 63, Fulfilling infrastructures and giving trainings or creating awareness… in KII / 3:1 ¶ 3, May be it is more fit to towns and less likely for rural because of di… in Group discussion / 3:53 ¶ 37, Mobile is now part of the basic needs of our community. Mobile service… in Group discussion / 3:68 ¶ 55, Sustainability of the program is the concern, who will always look aft… in Group discussion / 3:70 ¶ 58, Resource to maintain the service. Interruption of supports, government… in Group discussion

2:1 ¶ 9 in KII

Text quotation

Created by Girma Gilano on 3/9/2023

If anything necessary fulfilled, it is possible to implement.

1 Codes:

- **Challenges of mHealth impleentation: Readiness**

Comment: by Girma Gilano

> *3/12/2023 12:56:31 PM, merged with Security 3/12/2023 12:56:54 PM, merged with Readiness 3/19/2023 2:04:55 AM, merged with Challenges of mHealth impleentation: Resources 3/12/2023 12:56:31 PM, merged with Security 3/12/2023 12:56:54 PM, merged with Readiness*

15 Quotations:

1:79 ¶ 78, There could be a little challenge for this because the service is new in interview analysis / 1:85 ¶ 85, But we have to stress continuity and ownership, otherwise when there i… in interview analysis / 1:93 ¶ 93, Economy, maternal capacity of reading and understanding. Our mothers a… in interview analysis / 1:94 ¶ 94, Uneducated, inability to read, electricity, Mothers may not want to us… in interview analysis / 1:106 ¶ 106, All of them are solvable but may need time to solve in interview analysis / 2:1 ¶ 9, If anything necessary fulfilled, it is possible to implement. in KII / 2:18 ¶ 31, Yes, it will be difficult without infrastructure, it will be completel… in KII / 2:21 ¶ 34, This health facility is little bit less organized in materials compute… in KII / 2:22 ¶ 35, I think rural health institutions have a better maternal and child hea… in KII / 2:40 ¶ 60, I have to full resources to start this service. Awareness to health pr… in KII / 2:42 ¶ 63, Fulfilling infrastructures and giving trainings or creating awareness… in KII / 3:1 ¶ 3, May be it is more fit to towns and less likely for rural because of di… in Group discussion / 3:53 ¶ 37, Mobile is now part of the basic needs of our community. Mobile service… in Group discussion / 3:68 ¶ 55, Sustainability of the program is the concern, who will always look aft… in Group discussion / 3:70 ¶ 58, Resource to maintain the service. Interruption of supports, government… in Group discussion

2:18 ¶ 31 in KII

Text quotation

Created by Girma Gilano on 3/9/2023

Yes, it will be difficult without infrastructure, it will be completely impossible for example if no network, internet, and professional/skill of these activities

1 Codes:

- **Challenges of mHealth impleentation: Readiness**

Comment: by Girma Gilano

> *3/12/2023 12:56:31 PM, merged with Security 3/12/2023 12:56:54 PM, merged with Readiness 3/19/2023 2:04:55 AM, merged with Challenges of mHealth impleentation: Resources 3/12/2023 12:56:31 PM, merged with Security 3/12/2023 12:56:54 PM, merged with Readiness*

15 Quotations:

1:79 ¶ 78, There could be a little challenge for this because the service is new in interview analysis / 1:85 ¶ 85, But we have to stress continuity and ownership, otherwise when there i… in interview analysis / 1:93 ¶ 93, Economy, maternal capacity of reading and understanding. Our mothers a… in interview analysis / 1:94 ¶ 94, Uneducated, inability to read, electricity, Mothers may not want to us… in interview analysis / 1:106 ¶ 106, All of them are solvable but may need time to solve in interview analysis / 2:1 ¶ 9, If anything necessary fulfilled, it is possible to implement. in KII / 2:18 ¶ 31, Yes, it will be difficult without infrastructure, it will be completel… in KII / 2:21 ¶ 34, This health facility is little bit less organized in materials compute… in KII / 2:22 ¶ 35, I think rural health institutions have a better maternal and child hea… in KII / 2:40 ¶ 60, I have to full resources to start this service. Awareness to health pr… in KII / 2:42 ¶ 63, Fulfilling infrastructures and giving trainings or creating awareness… in KII / 3:1 ¶ 3, May be it is more fit to towns and less likely for rural because of di… in Group discussion / 3:53 ¶ 37, Mobile is now part of the basic needs of our community. Mobile service… in Group discussion / 3:68 ¶ 55, Sustainability of the program is the concern, who will always look aft… in Group discussion / 3:70 ¶ 58, Resource to maintain the service. Interruption of supports, government… in Group discussion

2:21 ¶ 34 in KII

Text quotation

Created by Girma Gilano on 3/9/2023

This health facility is little bit less organized in materials computers

1 Codes:

● Challenges of mHealth impleentation: Readiness

Comment: by Girma Gilano

> *3/12/2023 12:56:31 PM, merged with Security 3/12/2023 12:56:54 PM, merged with Readiness 3/19/2023 2:04:55 AM, merged with Challenges of mHealth impleentation: Resources 3/12/2023 12:56:31 PM, merged with Security 3/12/2023 12:56:54 PM, merged with Readiness*

15 Quotations:

1:79 ¶ 78, There could be a little challenge for this because the service is new in interview analysis / 1:85 ¶ 85, But we have to stress continuity and ownership, otherwise when there i… in interview analysis / 1:93 ¶ 93, Economy, maternal capacity of reading and understanding. Our mothers a… in interview analysis / 1:94 ¶ 94, Uneducated, inability to read, electricity, Mothers may not want to us… in interview analysis / 1:106 ¶ 106, All of them are solvable but may need time to solve in interview analysis / 2:1 ¶ 9, If anything necessary fulfilled, it is possible to implement. in KII / 2:18 ¶ 31, Yes, it will be difficult without infrastructure, it will be completel… in KII / 2:21 ¶ 34, This health facility is little bit less organized in materials compute… in KII / 2:22 ¶ 35, I think rural health institutions have a better maternal and child hea… in KII / 2:40 ¶ 60, I have to full resources to start this service. Awareness to health pr… in KII / 2:42 ¶ 63, Fulfilling infrastructures and giving trainings or creating awareness… in KII / 3:1 ¶ 3, May be it is more fit to towns and less likely for rural because of di… in Group discussion / 3:53 ¶ 37, Mobile is now part of the basic needs of our community. Mobile service… in Group discussion / 3:68 ¶ 55, Sustainability of the program is the concern, who will always look aft… in Group discussion / 3:70 ¶ 58, Resource to maintain the service. Interruption of supports, government… in Group discussion

2:22 ¶ 35 in KII

Text quotation

Created by Girma Gilano on 3/9/2023

I think rural health institutions have a better maternal and child health related service

1 Codes:

● Challenges of mHealth impleentation: Readiness

Comment: by Girma Gilano

> *3/12/2023 12:56:31 PM, merged with Security 3/12/2023 12:56:54 PM, merged with Readiness 3/19/2023 2:04:55 AM, merged with Challenges of mHealth impleentation: Resources 3/12/2023 12:56:31 PM, merged with Security 3/12/2023 12:56:54 PM, merged with Readiness*

15 Quotations:

1:79 ¶ 78, There could be a little challenge for this because the service is new in interview analysis / 1:85 ¶ 85, But we have to stress continuity and ownership, otherwise when there i… in interview analysis / 1:93 ¶ 93, Economy, maternal capacity of reading and understanding. Our mothers a… in interview analysis / 1:94 ¶ 94, Uneducated, inability to read, electricity, Mothers may not want to us… in interview analysis / 1:106 ¶ 106, All of them are solvable but may need time to solve in interview analysis / 2:1 ¶ 9, If anything necessary fulfilled, it is possible to implement. in KII / 2:18 ¶ 31, Yes, it will be difficult without

infrastructure, it will be completel… in KII / 2:21 ¶ 34, This health facility is little bit less organized in materials compute… in KII / 2:22 ¶ 35, I think rural health institutions have a better maternal and child hea… in KII / 2:40 ¶ 60, I have to full resources to start this service. Awareness to health pr… in KII / 2:42 ¶ 63, Fulfilling infrastructures and giving trainings or creating awareness… in KII / 3:1 ¶ 3, May be it is more fit to towns and less likely for rural because of di… in Group discussion / 3:53 ¶ 37, Mobile is now part of the basic needs of our community. Mobile service… in Group discussion / 3:68 ¶ 55, Sustainability of the program is the concern, who will always look aft… in Group discussion / 3:70 ¶ 58, Resource to maintain the service. Interruption of supports, government… in Group discussion

2:40 ¶ 60 in KII

Text quotation

Created by Girma Gilano on 3/9/2023

I have to full resources to start this service. Awareness to health professionals

1 Codes:

- **Challenges of mHealth impleentation: Readiness**

Comment: by Girma Gilano

> *3/12/2023 12:56:31 PM, merged with Security 3/12/2023 12:56:54 PM, merged with Readiness 3/19/2023 2:04:55 AM, merged with Challenges of mHealth impleentation: Resources 3/12/2023 12:56:31 PM, merged with Security 3/12/2023 12:56:54 PM, merged with Readiness*

15 Quotations:

1:79 ¶ 78, There could be a little challenge for this because the service is new in interview analysis / 1:85 ¶ 85, But we have to stress continuity and ownership, otherwise when there i… in interview analysis / 1:93 ¶ 93, Economy, maternal capacity of reading and understanding. Our mothers a… in interview analysis / 1:94 ¶ 94, Uneducated, inability to read, electricity, Mothers may not want to us… in interview analysis / 1:106 ¶ 106, All of them are solvable but may need time to solve in interview analysis / 2:1 ¶ 9, If anything necessary fulfilled, it is possible to implement. in KII / 2:18 ¶ 31, Yes, it will be difficult without infrastructure, it will be completel… in KII / 2:21 ¶ 34, This health facility is little bit less organized in materials compute… in KII / 2:22 ¶ 35, I think rural health institutions have a better maternal and child hea… in KII / 2:40 ¶ 60, I have to full resources to start this service. Awareness to health pr… in KII / 2:42 ¶ 63, Fulfilling infrastructures and giving trainings or creating awareness… in KII / 3:1 ¶ 3, May be it is more fit to towns and less likely for rural because of di… in Group discussion / 3:53 ¶ 37, Mobile is now part of the basic needs of our community. Mobile service… in Group discussion / 3:68 ¶ 55, Sustainability of the program is the concern, who will always look aft… in Group discussion / 3:70 ¶ 58, Resource to maintain the service. Interruption of supports, government… in Group discussion

2:42 ¶ 63 in KII

Text quotation

Created by Girma Gilano on 3/9/2023

Fulfilling infrastructures and giving trainings or creating awareness both in staffs and communities

1 Codes:

● Challenges of mHealth impleentation: Readiness

Comment: by Girma Gilano

> *3/12/2023 12:56:31 PM, merged with Security 3/12/2023 12:56:54 PM, merged with Readiness 3/19/2023 2:04:55 AM, merged with Challenges of mHealth impleentation: Resources 3/12/2023 12:56:31 PM, merged with Security 3/12/2023 12:56:54 PM, merged with Readiness*

15 Quotations:

1:79 ¶ 78, There could be a little challenge for this because the service is new in interview analysis / 1:85 ¶ 85, But we have to stress continuity and ownership, otherwise when there i… in interview analysis / 1:93 ¶ 93, Economy, maternal capacity of reading and understanding. Our mothers a… in interview analysis / 1:94 ¶ 94, Uneducated, inability to read, electricity, Mothers may not want to us… in interview analysis / 1:106 ¶ 106, All of them are solvable but may need time to solve in interview analysis / 2:1 ¶ 9, If anything necessary fulfilled, it is possible to implement. in KII / 2:18 ¶ 31, Yes, it will be difficult without infrastructure, it will be completel… in KII / 2:21 ¶ 34, This health facility is little bit less organized in materials compute… in KII / 2:22 ¶ 35, I think rural health institutions have a better maternal and child hea… in KII / 2:40 ¶ 60, I have to full resources to start this service. Awareness to health pr… in KII / 2:42 ¶ 63, Fulfilling infrastructures and giving trainings or creating awareness… in KII / 3:1 ¶ 3, May be it is more fit to towns and less likely for rural because of di… in Group discussion / 3:53 ¶ 37, Mobile is now part of the basic needs of our community. Mobile service… in Group discussion / 3:68 ¶ 55, Sustainability of the program is the concern, who will always look aft… in Group discussion / 3:70 ¶ 58, Resource to maintain the service. Interruption of supports, government… in Group discussion

3:1 ¶ 3 in Group discussion

Text quotation

Created by Girma Gilano on 3/9/2023

May be it is more fit to towns and less likely for rural because of different reasons

1 Codes:

● Challenges of mHealth impleentation: Readiness

Comment: by Girma Gilano

> *3/12/2023 12:56:31 PM, merged with Security 3/12/2023 12:56:54 PM, merged with Readiness 3/19/2023 2:04:55 AM, merged with Challenges of mHealth impleentation: Resources 3/12/2023 12:56:31 PM, merged with Security 3/12/2023 12:56:54 PM, merged with Readiness*

15 Quotations:

1:79 ¶ 78, There could be a little challenge for this because the service is new in interview analysis / 1:85 ¶ 85, But we have to stress continuity and ownership, otherwise when there i… in interview analysis / 1:93 ¶ 93, Economy, maternal capacity of reading and understanding. Our mothers a… in interview analysis / 1:94 ¶ 94, Uneducated, inability to read, electricity, Mothers may not want to us… in interview analysis / 1:106 ¶ 106, All of them are solvable but may need time to solve in interview analysis / 2:1 ¶ 9, If anything necessary fulfilled, it is possible to implement. in KII / 2:18 ¶ 31, Yes, it will be difficult without infrastructure, it will be completel… in KII / 2:21 ¶ 34, This health facility is little bit less organized in materials compute… in KII / 2:22 ¶ 35, I think rural health institutions have a better maternal and child hea… in KII / 2:40 ¶ 60, I have to full resources to start this service. Awareness to health pr… in KII / 2:42 ¶ 63, Fulfilling infrastructures and giving trainings or creating awareness… in KII / 3:1 ¶ 3, May be it is more fit to towns and less likely for rural because of di… in Group discussion / 3:53 ¶ 37, Mobile is

now part of the basic needs of our community. Mobile service… in Group discussion / 3:68 ¶ 55, Sustainability of the program is the concern, who will always look aft… in Group discussion / 3:70 ¶ 58, Resource to maintain the service. Interruption of supports, government… in Group discussion

3:53 ¶ 37 in Group discussion

Text quotation

Created by Girma Gilano on 3/9/2023

Mobile is now part of the basic needs of our community. Mobile services are the most used thing in the community, so what possible culture could avoid using mHealth? Exactly there is no culture

2 Codes:

- **Challenges of mHealth impleentation: Devices_handling**

Comment: by Girma Gilano

> *3/12/2023 12:56:31 PM, merged with Security 3/12/2023 12:56:54 PM, merged with Readiness*

12 Quotations:

1:54 ¶ 59, But no all mothers have mobiles. Some time they may loss their mobile… in interview analysis / 1:58 ¶ 63, This can improve the service but not all women have phone, many mother… in interview analysis / 1:63 ¶ 66, I can say it can be effective, but not all mother have mobiles, mobile… in interview analysis / 1:93 ¶ 93, Economy, maternal capacity of reading and understanding. Our mothers a… in interview analysis / 1:94 ¶ 94, Uneducated, inability to read, electricity, Mothers may not want to us… in interview analysis / 1:96 ¶ 96, May not have phone because of economy i.e. every mother may not phone… in interview analysis / 1:97 ¶ 97, Mother lost existing phone within the service period, broken Phone whi… in interview analysis / 1:106 ¶ 106, All of them are solvable but may need time to solve in interview analysis / 2:29 ¶ 44, Infants are not owners of the service but with family only and if fami… in KII / 3:28 ¶ 17, This service is possible and when mother do have mobile unless another… in Group discussion / 3:53 ¶ 37, Mobile is now part of the basic needs of our community. Mobile service… in Group discussion / 3:68 ¶ 55, Sustainability of the program is the concern, who will always look aft… in Group discussion

- **Challenges of mHealth impleentation: Readiness**

Comment: by Girma Gilano

> *3/12/2023 12:56:31 PM, merged with Security 3/12/2023 12:56:54 PM, merged with Readiness 3/19/2023 2:04:55 AM, merged with Challenges of mHealth impleentation: Resources 3/12/2023 12:56:31 PM, merged with Security 3/12/2023 12:56:54 PM, merged with Readiness*

15 Quotations:

1:79 ¶ 78, There could be a little challenge for this because the service is new in interview analysis / 1:85 ¶ 85, But we have to stress continuity and ownership, otherwise when there i… in interview analysis / 1:93 ¶ 93, Economy, maternal capacity of reading and understanding. Our mothers a… in interview analysis / 1:94 ¶ 94, Uneducated, inability to read, electricity, Mothers may not want to us… in interview analysis / 1:106 ¶ 106, All of them are solvable but may need time to solve in interview analysis / 2:1 ¶ 9, If anything necessary fulfilled, it is possible to implement. in KII / 2:18 ¶ 31, Yes, it will be difficult without infrastructure, it will be completel… in KII / 2:21 ¶ 34, This health facility is little bit less organized in materials compute… in KII / 2:22 ¶ 35, I think rural health institutions have a better maternal and child

hea… in KII / 2:40 ¶ 60, I have to full resources to start this service. Awareness to health pr… in KII / 2:42 ¶ 63, Fulfilling infrastructures and giving trainings or creating awareness… in KII / 3:1 ¶ 3, May be it is more fit to towns and less likely for rural because of di… in Group discussion / 3:53 ¶ 37, Mobile is now part of the basic needs of our community. Mobile service… in Group discussion / 3:68 ¶ 55, Sustainability of the program is the concern, who will always look aft… in Group discussion / 3:70 ¶ 58, Resource to maintain the service. Interruption of supports, government… in Group discussion

3:68 ¶ 55 in Group discussion

Text quotation

Created by Girma Gilano on 3/9/2023

Sustainability of the program is the concern, who will always look after computer

2 Codes:

- **Challenges of mHealth impleentation: Devices_handling**

Comment: by Girma Gilano

> *3/12/2023 12:56:31 PM, merged with Security 3/12/2023 12:56:54 PM, merged with Readiness*

12 Quotations:

1:54 ¶ 59, But no all mothers have mobiles. Some time they may loss their mobile… in interview analysis / 1:58 ¶ 63, This can improve the service but not all women have phone, many mother… in interview analysis / 1:63 ¶ 66, I can say it can be effective, but not all mother have mobiles, mobile… in interview analysis / 1:93 ¶ 93, Economy, maternal capacity of reading and understanding. Our mothers a… in interview analysis / 1:94 ¶ 94, Uneducated, inability to read, electricity, Mothers may not want to us… in interview analysis / 1:96 ¶ 96, May not have phone because of economy i.e. every mother may not phone… in interview analysis / 1:97 ¶ 97, Mother lost existing phone within the service period, broken Phone whi… in interview analysis / 1:106 ¶ 106, All of them are solvable but may need time to solve in interview analysis / 2:29 ¶ 44, Infants are not owners of the service but with family only and if fami… in KII / 3:28 ¶ 17, This service is possible and when mother do have mobile unless another… in Group discussion / 3:53 ¶ 37, Mobile is now part of the basic needs of our community. Mobile service… in Group discussion / 3:68 ¶ 55, Sustainability of the program is the concern, who will always look aft… in Group discussion

- **Challenges of mHealth impleentation: Readiness**

Comment: by Girma Gilano

> *3/12/2023 12:56:31 PM, merged with Security 3/12/2023 12:56:54 PM, merged with Readiness 3/19/2023 2:04:55 AM, merged with Challenges of mHealth impleentation: Resources 3/12/2023 12:56:31 PM, merged with Security 3/12/2023 12:56:54 PM, merged with Readiness*

15 Quotations:

1:79 ¶ 78, There could be a little challenge for this because the service is new in interview analysis / 1:85 ¶ 85, But we have to stress continuity and ownership, otherwise when there i… in interview analysis / 1:93 ¶ 93, Economy, maternal capacity of reading and understanding. Our mothers a… in interview analysis / 1:94 ¶ 94, Uneducated, inability to read, electricity, Mothers may not want to us… in interview analysis / 1:106 ¶ 106, All of them are solvable but may need time to solve in interview analysis / 2:1 ¶ 9, If anything necessary fulfilled, it is possible to implement. in KII / 2:18 ¶ 31, Yes, it will be difficult without infrastructure, it will be completel… in KII / 2:21 ¶ 34, This health facility is little bit less organized in materials compute… in KII / 2:22 ¶ 35, I think rural health institutions have a better maternal and child

hea… in KII / 2:40 ¶ 60, I have to full resources to start this service. Awareness to health pr… in KII / 2:42 ¶ 63, Fulfilling infrastructures and giving trainings or creating awareness… in KII / 3:1 ¶ 3, May be it is more fit to towns and less likely for rural because of di… in Group discussion / 3:53 ¶ 37, Mobile is now part of the basic needs of our community. Mobile service… in Group discussion / 3:68 ¶ 55, Sustainability of the program is the concern, who will always look aft… in Group discussion / 3:70 ¶ 58, Resource to maintain the service. Interruption of supports, government… in Group discussion

3:70 ¶ 58 in Group discussion

Text quotation

Created by Girma Gilano on 3/9/2023, **modified** by Girma Gilano on 3/9/2023

Resource to maintain the service. Interruption of supports, government concerns. Internet availability, and electricity interruptions and internet interruption might tempt the service

1 Codes:

● Challenges of mHealth impleentation: Readiness

Comment: by Girma Gilano

> *3/12/2023 12:56:31 PM, merged with Security 3/12/2023 12:56:54 PM, merged with Readiness 3/19/2023 2:04:55 AM, merged with Challenges of mHealth impleentation: Resources 3/12/2023 12:56:31 PM, merged with Security 3/12/2023 12:56:54 PM, merged with Readiness*

15 Quotations:

1:79 ¶ 78, There could be a little challenge for this because the service is new in interview analysis / 1:85 ¶ 85, But we have to stress continuity and ownership, otherwise when there i… in interview analysis / 1:93 ¶ 93, Economy, maternal capacity of reading and understanding. Our mothers a… in interview analysis / 1:94 ¶ 94, Uneducated, inability to read, electricity, Mothers may not want to us… in interview analysis / 1:106 ¶ 106, All of them are solvable but may need time to solve in interview analysis / 2:1 ¶ 9, If anything necessary fulfilled, it is possible to implement. in KII / 2:18 ¶ 31, Yes, it will be difficult without infrastructure, it will be completel… in KII / 2:21 ¶ 34, This health facility is little bit less organized in materials compute… in KII / 2:22 ¶ 35, I think rural health institutions have a better maternal and child hea… in KII / 2:40 ¶ 60, I have to full resources to start this service. Awareness to health pr… in KII / 2:42 ¶ 63, Fulfilling infrastructures and giving trainings or creating awareness… in KII / 3:1 ¶ 3, May be it is more fit to towns and less likely for rural because of di… in Group discussion / 3:53 ¶ 37, Mobile is now part of the basic needs of our community. Mobile service… in Group discussion / 3:68 ¶ 55, Sustainability of the program is the concern, who will always look aft… in Group discussion / 3:70 ¶ 58, Resource to maintain the service. Interruption of supports, government… in Group discussion

---

● Challenges of mHealth impleentation: Security

Created by Girma Gilano on 3/12/2023

Comment: by Girma Gilano

> *3/12/2023 12:56:31 PM, merged with Security 3/12/2023 12:56:54 PM, merged with Readiness*

6 Quotations:

1:80 ¶ 79 in interview analysis

Text quotation

Created by Girma Gilano on 3/8/2023

Reading ability and of a person who can read in some cases can cause some problem, if children are not even learned

1 Codes:

- **Challenges of mHealth impleentation: Security**

Comment: by Girma Gilano

> *3/12/2023 12:56:31 PM, merged with Security 3/12/2023 12:56:54 PM, merged with Readiness*

6 Quotations:

1:80 ¶ 79, Reading ability and of a person who can read in some cases can cause… in interview analysis / 1:87 ¶ 85, Privacy and confidentiality are not a problem as the exchange is betwe… in interview analysis / 1:88 ¶ 86, If everyone can access the information every women accept, there could… in interview analysis / 1:89 ¶ 87, Mothers do not hide information. Health professionals have ethics, so… in interview analysis / 1:90 ¶ 88, As health professionals we have to keep client privacy. Generally we d… in interview analysis / 1:100 ¶ 99, Confidentiality and privacy should be kept by a person who registering… in interview analysis

1:87 ¶ 85 in interview analysis

Text quotation

Created by Girma Gilano on 3/9/2023

Privacy and confidentiality are not a problem as the exchange is between mother and professional

1 Codes:

- **Challenges of mHealth impleentation: Security**

Comment: by Girma Gilano

> *3/12/2023 12:56:31 PM, merged with Security 3/12/2023 12:56:54 PM, merged with Readiness*

6 Quotations:

1:80 ¶ 79, Reading ability and of a person who can read in some cases can cause… in interview analysis / 1:87 ¶ 85, Privacy and confidentiality are not a problem as the exchange is betwe… in interview analysis / 1:88 ¶ 86, If everyone can access the information every women accept, there could… in interview analysis / 1:89 ¶ 87, Mothers do not hide information. Health professionals have ethics, so… in interview analysis / 1:90 ¶ 88, As health professionals we have to keep client privacy. Generally we d… in interview analysis / 1:100 ¶ 99, Confidentiality and privacy should be kept by a person who registering… in interview analysis

1:88 ¶ 86 in interview analysis

Text quotation

Created by Girma Gilano on 3/9/2023

If everyone can access the information every women accept, there could be little security issue. The information going to mothers hand not anywhere else so why we fear

1 Codes:

- **Challenges of mHealth impleentation: Security**

  Comment: by Girma Gilano

  > *3/12/2023 12:56:31 PM, merged with Security 3/12/2023 12:56:54 PM, merged with Readiness*

  6 Quotations:

  1:80 ¶ 79, Reading ability and of a person who can read in some cases can cause… in interview analysis / 1:87 ¶ 85, Privacy and confidentiality are not a problem as the exchange is betwe… in interview analysis / 1:88 ¶ 86, If everyone can access the information every women accept, there could… in interview analysis / 1:89 ¶ 87, Mothers do not hide information. Health professionals have ethics, so… in interview analysis / 1:90 ¶ 88, As health professionals we have to keep client privacy. Generally we d… in interview analysis / 1:100 ¶ 99, Confidentiality and privacy should be kept by a person who registering… in interview analysis

1:89 ¶ 87 in interview analysis

Text quotation

Created by Girma Gilano on 3/9/2023

Mothers do not hide information. Health professionals have ethics, so I don't the any breaching on provider side

1 Codes:

- **Challenges of mHealth impleentation: Security**

  Comment: by Girma Gilano

  > *3/12/2023 12:56:31 PM, merged with Security 3/12/2023 12:56:54 PM, merged with Readiness*

  6 Quotations:

  1:80 ¶ 79, Reading ability and of a person who can read in some cases can cause… in interview analysis / 1:87 ¶ 85, Privacy and confidentiality are not a problem as the exchange is betwe… in interview analysis / 1:88 ¶ 86, If everyone can access the information every women accept, there could… in interview analysis / 1:89 ¶ 87, Mothers do not hide information. Health professionals have ethics, so… in interview analysis / 1:90 ¶ 88, As health professionals we have to keep client privacy. Generally we d… in interview analysis / 1:100 ¶ 99, Confidentiality and privacy should be kept by a person who registering… in interview analysis

1:90 ¶ 88 in interview analysis

Text quotation

Created by Girma Gilano on 3/9/2023, **modified** by Girma Gilano on 3/9/2023

As health professionals we have to keep client privacy. Generally we don't think this will breach client privacy. No security issue because the exchange of information is between professionals and mothers.

1 Codes:

- **Challenges of mHealth impleentation: Security**

  Comment: by Girma Gilano

  > *3/12/2023 12:56:31 PM, merged with Security 3/12/2023 12:56:54 PM, merged with Readiness*

  6 Quotations:

  1:80 ¶ 79, Reading ability and of a person who can read in some cases can cause… in interview analysis / 1:87 ¶ 85, Privacy and confidentiality are not a problem as the exchange is betwe… in interview analysis / 1:88 ¶ 86, If everyone can access the information every women accept, there could… in interview analysis / 1:89 ¶ 87, Mothers do not hide information. Health professionals have ethics, so… in interview analysis / 1:90 ¶ 88, As health professionals we have to keep client privacy. Generally we d… in interview analysis / 1:100 ¶ 99, Confidentiality and privacy should be kept by a person who registering… in interview analysis

1:100 ¶ 99 in interview analysis

Text quotation

Created by Girma Gilano on 3/9/2023

Confidentiality and privacy should be kept by a person who registering information i.e. understanding of the message should be considered otherwise mother may go for who can read the message

1 Codes:

- **Challenges of mHealth impleentation: Security**

  Comment: by Girma Gilano

  > *3/12/2023 12:56:31 PM, merged with Security 3/12/2023 12:56:54 PM, merged with Readiness*

  6 Quotations:

  1:80 ¶ 79, Reading ability and of a person who can read in some cases can cause… in interview analysis / 1:87 ¶ 85, Privacy and confidentiality are not a problem as the exchange is betwe… in interview analysis / 1:88 ¶ 86, If everyone can access the information every women accept, there could… in interview analysis / 1:89 ¶ 87, Mothers do not hide information. Health professionals have ethics, so… in interview analysis / 1:90 ¶ 88, As health professionals we have to keep client privacy.

Generally we d… in interview analysis / 1:100 ¶ 99, Confidentiality and privacy should be kept by a person who registering… in interview analysis

---

● Challenges of mHealth impleentation: workload

Created by Girma Gilano on 3/12/2023

Comment: by Girma Gilano

> *3/12/2023 12:56:31 PM, merged with Security 3/12/2023 12:56:54 PM, merged with Readiness*

7 Quotations:

1:2 ¶ 18 in interview analysis

Text quotation

Created by Girma Gilano on 3/8/2023

By the way mother knew their appointment but it is just the load of work they have everyday. Unless she says I am sick, she cannot allowed to go to the health institutions. It looks like there is no way to change but there should be some strategies for mother of daily laborer

1 Codes:

● Challenges of mHealth impleentation: workload

Comment: by Girma Gilano

> *3/12/2023 12:56:31 PM, merged with Security 3/12/2023 12:56:54 PM, merged with Readiness*

7 Quotations:

1:2 ¶ 18, By the way mother knew their appointment but it is just the load of wo… in interview analysis / 1:16 ¶ 29, Usually mothers know their date but since they are daily laborer, they… in interview analysis / 1:17 ¶ 30, Some mothers are the heavy workers and cannot avoid missing the appoin… in interview analysis / 1:31 ¶ 38, this will increase pressure on professionals in interview analysis / 3:3 ¶ 4, I appreciate to have technology assistance, but mothers do not forget… in Group discussion / 3:4 ¶ 4, Daily laborers usually do not have time for themselves because of the… in Group discussion / 3:65 ¶ 52, Reluctance, being bored of many messages over time, .pre occupation wi… in Group discussion

1:16 ¶ 29 in interview analysis

Text quotation

Created by Girma Gilano on 3/8/2023

Usually mothers know their date but since they are daily laborer, they do not have time, when they are not working and also want to come the have complain false reason to come for ANC

1 Codes:

- **Challenges of mHealth impleentation: workload**

  Comment: by Girma Gilano

  > *3/12/2023 12:56:31 PM, merged with Security 3/12/2023 12:56:54 PM, merged with Readiness*

  7 Quotations:

  1:2 ¶ 18, By the way mother knew their appointment but it is just the load of wo… in interview analysis / 1:16 ¶ 29, Usually mothers know their date but since they are daily laborer, they… in interview analysis / 1:17 ¶ 30, Some mothers are the heavy workers and cannot avoid missing the appoin… in interview analysis / 1:31 ¶ 38, this will increase pressure on professionals in interview analysis / 3:3 ¶ 4, I appreciate to have technology assistance, but mothers do not forget… in Group discussion / 3:4 ¶ 4, Daily laborers usually do not have time for themselves because of the… in Group discussion / 3:65 ¶ 52, Reluctance, being bored of many messages over time, .pre occupation wi… in Group discussion

1:17 ¶ 30 in interview analysis

Text quotation

Created by Girma Gilano on 3/8/2023

Some mothers are the heavy workers and cannot avoid missing the appointment. This will fill our gap and reduce mortality

1 Codes:

- **Challenges of mHealth impleentation: workload**

  Comment: by Girma Gilano

  > *3/12/2023 12:56:31 PM, merged with Security 3/12/2023 12:56:54 PM, merged with Readiness*

  7 Quotations:

  1:2 ¶ 18, By the way mother knew their appointment but it is just the load of wo… in interview analysis / 1:16 ¶ 29, Usually mothers know their date but since they are daily laborer, they… in interview analysis / 1:17 ¶ 30, Some mothers are the heavy workers and cannot avoid missing the appoin… in interview analysis / 1:31 ¶ 38, this will increase pressure on professionals in interview analysis / 3:3 ¶ 4, I appreciate to have technology assistance, but mothers do not forget… in Group discussion / 3:4 ¶ 4, Daily laborers usually do not have time for themselves because of the… in Group discussion / 3:65 ¶ 52, Reluctance, being bored of many messages over time, .pre occupation wi… in Group discussion

1:31 ¶ 38 in interview analysis

Text quotation

Created by Girma Gilano on 3/8/2023

this will increase pressure on professionals

1 Codes:

- **Challenges of mHealth impleentation: workload**

  Comment: by Girma Gilano

  > *3/12/2023 12:56:31 PM, merged with Security 3/12/2023 12:56:54 PM, merged with Readiness*

  7 Quotations:

  1:2 ¶ 18, By the way mother knew their appointment but it is just the load of wo… in interview analysis / 1:16 ¶ 29, Usually mothers know their date but since they are daily laborer, they… in interview analysis / 1:17 ¶ 30, Some mothers are the heavy workers and cannot avoid missing the appoin… in interview analysis / 1:31 ¶ 38, this will increase pressure on professionals in interview analysis / 3:3 ¶ 4, I appreciate to have technology assistance, but mothers do not forget… in Group discussion / 3:4 ¶ 4, Daily laborers usually do not have time for themselves because of the… in Group discussion / 3:65 ¶ 52, Reluctance, being bored of many messages over time, .pre occupation wi… in Group discussion

3:3 ¶ 4 in Group discussion

Text quotation

Created by Girma Gilano on 3/9/2023

I appreciate to have technology assistance, but mothers do not forget appointment but they held back by their work intensity. May be they are giving more attention for their work than health

1 Codes:

- **Challenges of mHealth impleentation: workload**

  Comment: by Girma Gilano

  > *3/12/2023 12:56:31 PM, merged with Security 3/12/2023 12:56:54 PM, merged with Readiness*

  7 Quotations:

  1:2 ¶ 18, By the way mother knew their appointment but it is just the load of wo… in interview analysis / 1:16 ¶ 29, Usually mothers know their date but since they are daily laborer, they… in interview analysis / 1:17 ¶ 30, Some mothers are the heavy workers and cannot avoid missing the appoin… in interview analysis / 1:31 ¶ 38, this will increase pressure on professionals in interview analysis / 3:3 ¶ 4, I appreciate to have technology assistance, but mothers do not forget… in Group discussion / 3:4 ¶ 4, Daily laborers usually do not have time for themselves because of the… in Group discussion / 3:65 ¶ 52, Reluctance, being bored of many messages over time, .pre occupation wi… in Group discussion

3:4 ¶ 4 in Group discussion

Text quotation

Created by Girma Gilano on 3/9/2023

Daily laborers usually do not have time for themselves because of the strictness of the work.

1 Codes:

- **Challenges of mHealth impleentation: workload**

Comment: by Girma Gilano

> *3/12/2023 12:56:31 PM, merged with Security 3/12/2023 12:56:54 PM, merged with Readiness*

7 Quotations:

1:2 ¶ 18, By the way mother knew their appointment but it is just the load of wo… in interview analysis / 1:16 ¶ 29, Usually mothers know their date but since they are daily laborer, they… in interview analysis / 1:17 ¶ 30, Some mothers are the heavy workers and cannot avoid missing the appoin… in interview analysis / 1:31 ¶ 38, this will increase pressure on professionals in interview analysis / 3:3 ¶ 4, I appreciate to have technology assistance, but mothers do not forget… in Group discussion / 3:4 ¶ 4, Daily laborers usually do not have time for themselves because of the… in Group discussion / 3:65 ¶ 52, Reluctance, being bored of many messages over time, .pre occupation wi… in Group discussion

3:65 ¶ 52 in Group discussion

Text quotation

Created by Girma Gilano on 3/9/2023

Reluctance, being bored of many messages over time, .pre occupation with important thing, hand-to-mouth life style, and daily laborer work

2 Codes:

- **Challenges of mHealth impleentation: negligence**

Comment: by Girma Gilano

> *3/12/2023 12:56:31 PM, merged with Security 3/12/2023 12:56:54 PM, merged with Readiness*

7 Quotations:

1:3 ¶ 19, but some are negligent, and mothers knew the consequence of not comply… in interview analysis / 1:7 ¶ 22, Normally, the mother come to health institution at 45 days but some st… in interview analysis / 1:72 ¶ 74, I don't think there will be a problem unless the carelessness of profe… in interview analysis / 1:95 ¶ 95, Because of this service is new some acceptance can be happen until eve… in interview analysis / 3:15 ¶ 10, I think it will be more helpful for rural mothers, for towns every mot… in Group discussion / 3:65 ¶ 52, Reluctance, being bored of many messages over time, .pre occupation wi… in Group discussion / 3:66 ¶ 53, Previous ignorance nature for services in Group discussion

- **Challenges of mHealth impleentation: workload**

Comment: by Girma Gilano

> *3/12/2023 12:56:31 PM, merged with Security 3/12/2023 12:56:54 PM, merged with Readiness*

7 Quotations:

1:2 ¶ 18, By the way mother knew their appointment but it is just the load of wo… in interview analysis / 1:16 ¶ 29, Usually mothers know their date but since they are daily laborer, they… in interview analysis / 1:17 ¶ 30, Some mothers are the heavy workers and cannot avoid missing the appoin… in interview analysis / 1:31 ¶ 38, this will increase pressure on professionals in interview analysis / 3:3 ¶ 4, I appreciate to have technology assistance, but mothers do not forget… in Group discussion / 3:4 ¶ 4, Daily laborers usually do not have time for themselves because of the… in Group discussion / 3:65 ¶ 52, Reluctance, being bored of many messages over time, .pre occupation wi… in Group discussion

---

Solutions: Family_help

Created by Girma Gilano on 3/12/2023, **modified** by Girma Gilano on 3/12/2023

Comment: by Girma Gilano

> *3/12/2023 1:51:52 PM, merged with Solutions: Local_help (2)*

8 Quotations:

1:12 ¶ 26 in interview analysis

Text quotation

Created by Girma Gilano on 3/8/2023

She can use her children's education to use the service

1 Codes:

Solutions: Family_help

Comment: by Girma Gilano

> *3/12/2023 1:51:52 PM, merged with Solutions: Local_help (2)*

8 Quotations:

1:12 ¶ 26, She can use her children's education to use the service in interview analysis / 1:103 ¶ 103, The thing is there are learned children in every household, so possibl… in interview analysis / 1:107 ¶ 106, Most women read the local language so if it can be in the local langua… in interview analysis / 1:108 ¶ 107, Teaching households to use phone especially to have successful pregnan… in interview analysis / 1:110 ¶ 109, Women's education and society overall should be promoted to learn at l… in interview analysis / 1:111 ¶ 110, To get mothers using this program husband should take large responsibi… in interview analysis / 3:30 ¶ 17, The service indeed can be provided when any phone in house like spouse… in Group discussion / 3:71 ¶ 60, Maternal education can be improved through adult education (Golmasa ti… in Group discussion

1:103 ¶ 103 in interview analysis

Text quotation

Created by Girma Gilano on 3/9/2023

The thing is there are learned children in every household, so possibly every mother can use this service if implemented.

1 Codes:

- **Solutions: Family_help**

Comment: by Girma Gilano

> *3/12/2023 1:51:52 PM, merged with Solutions: Local_help (2)*

8 Quotations:

1:12 ¶ 26, She can use her children's education to use the service in interview analysis / 1:103 ¶ 103, The thing is there are learned children in every household, so possibl… in interview analysis / 1:107 ¶ 106, Most women read the local language so if it can be in the local langua… in interview analysis / 1:108 ¶ 107, Teaching households to use phone especially to have successful pregnan… in interview analysis / 1:110 ¶ 109, Women's education and society overall should be promoted to learn at l… in interview analysis / 1:111 ¶ 110, To get mothers using this program husband should take large responsibi… in interview analysis / 3:30 ¶ 17, The service indeed can be provided when any phone in house like spouse… in Group discussion / 3:71 ¶ 60, Maternal education can be improved through adult education (Golmasa ti… in Group discussion

1:107 ¶ 106 in interview analysis

Text quotation

Created by Girma Gilano on 3/9/2023

Most women read the local language so if it can be in the local language it will be helpful. This means more than half of mothers can use this service. Almost every husband has a mobile and there taught children in every house to solve the maternal inability to read. A mobilizing community for maternal education and behavioral change is importan

2 Codes:

- **Solutions: Family_help**

Comment: by Girma Gilano

> *3/12/2023 1:51:52 PM, merged with Solutions: Local_help (2)*

8 Quotations:

1:12 ¶ 26, She can use her children's education to use the service in interview analysis / 1:103 ¶ 103, The thing is there are learned children in every household, so possibl… in interview analysis / 1:107 ¶ 106, Most women read the local language so if it can be in the local langua… in interview analysis / 1:108 ¶ 107, Teaching households to use phone especially to have successful pregnan… in interview analysis / 1:110 ¶ 109, Women's education and society overall should be promoted to learn at l… in interview analysis / 1:111 ¶ 110, To get mothers using this program husband should take large responsibi… in interview analysis / 3:30 ¶ 17, The service indeed can be provided when any phone in house like spouse… in Group discussion / 3:71 ¶ 60, Maternal education can be improved through adult education (Golmasa ti… in Group discussion

- **Solutions: Forum_help**

Comment: by Girma Gilano

3/12/2023 1:51:41 PM, merged with Solutions: Local_help 3/19/2023 2:59:22 AM, merged with Solutions: HDA

7 Quotations:

1:11 ¶ 26, for uneducated we have to teach through mothers forum, community forum… in interview analysis / 1:107 ¶ 106, Most women read the local language so if it can be in the local langua… in interview analysis / 1:109 ¶ 108, sing mothers forum, HDA, voluntary health team(በጎ ጤና ቡድን), community w… in interview analysis / 1:112 ¶ 111, Creating conducive political environment, creating awareness, using mo… in interview analysis / 2:19 ¶ 32, Human development and health development army can improve all the limi… in KII / 3:71 ¶ 60, Maternal education can be improved through adult education (Golmasa ti… in Group discussion / 3:74 ¶ 63, Using voluntary health team in each community to promote mHealth impli… in Group discussion

1:108 ¶ 107 in interview analysis

Text quotation

Created by Girma Gilano on 3/9/2023

Teaching households to use phone especially to have successful pregnancy and child development. Because of the importance of the service mother should request to have a phone nearby her. For uneducated mothers, Golmasa education, use of their children

1 Codes:

- **Solutions: Family_help**

Comment: by Girma Gilano

3/12/2023 1:51:52 PM, merged with Solutions: Local_help (2)

8 Quotations:

1:12 ¶ 26, She can use her children's education to use the service in interview analysis / 1:103 ¶ 103, The thing is there are learned children in every household, so possibl… in interview analysis / 1:107 ¶ 106, Most women read the local language so if it can be in the local langua… in interview analysis / 1:108 ¶ 107, Teaching households to use phone especially to have successful pregnan… in interview analysis / 1:110 ¶ 109, Women's education and society overall should be promoted to learn at l… in interview analysis / 1:111 ¶ 110, To get mothers using this program husband should take large responsibi… in interview analysis / 3:30 ¶ 17, The service indeed can be provided when any phone in house like spouse… in Group discussion / 3:71 ¶ 60, Maternal education can be improved through adult education (Golmasa ti… in Group discussion

1:110 ¶ 109 in interview analysis

Text quotation

Created by Girma Gilano on 3/9/2023

Women's education and society overall should be promoted to learn at least enable them to read is important. Before application of mHealth overall discussion might be necessary with the whole community

1 Codes:

- **Solutions: Family_help**

  Comment: by Girma Gilano

  > *3/12/2023 1:51:52 PM, merged with Solutions: Local_help (2)*

  8 Quotations:

  1:12 ¶ 26, She can use her children's education to use the service in interview analysis / 1:103 ¶ 103, The thing is there are learned children in every household, so possibl… in interview analysis / 1:107 ¶ 106, Most women read the local language so if it can be in the local langua… in interview analysis / 1:108 ¶ 107, Teaching households to use phone especially to have successful pregnan… in interview analysis / 1:110 ¶ 109, Women's education and society overall should be promoted to learn at l… in interview analysis / 1:111 ¶ 110, To get mothers using this program husband should take large responsibi… in interview analysis / 3:30 ¶ 17, The service indeed can be provided when any phone in house like spouse… in Group discussion / 3:71 ¶ 60, Maternal education can be improved through adult education (Golmasa ti… in Group discussion

1:111 ¶ 110 in interview analysis

Text quotation

Created by Girma Gilano on 3/9/2023

To get mothers using this program husband should take large responsibility, if we cannot do that they may even stand against the program. If the mother do not have mobile I think we can use husband, child, any phone in the house to access the mother. We have to use very possible strategies to get mother into this service

1 Codes:

- **Solutions: Family_help**

  Comment: by Girma Gilano

  > *3/12/2023 1:51:52 PM, merged with Solutions: Local_help (2)*

  8 Quotations:

  1:12 ¶ 26, She can use her children's education to use the service in interview analysis / 1:103 ¶ 103, The thing is there are learned children in every household, so possibl… in interview analysis / 1:107 ¶ 106, Most women read the local language so if it can be in the local langua… in interview analysis / 1:108 ¶ 107, Teaching households to use phone especially to have successful pregnan… in interview analysis / 1:110 ¶ 109, Women's education and society overall should be promoted to learn at l… in interview analysis / 1:111 ¶ 110, To get mothers using this program husband should take large responsibi… in interview analysis / 3:30 ¶ 17, The service indeed can be provided when any phone in house like spouse… in Group discussion / 3:71 ¶ 60, Maternal education can be improved through adult education (Golmasa ti… in Group discussion

3:30 ¶ 17 in Group discussion

Text quotation

Created by Girma Gilano on 3/9/2023

The service indeed can be provided when any phone in house like spouse, children and even neighborhoods phone

1 Codes:

● Solutions: Family_help

Comment: by Girma Gilano

> *3/12/2023 1:51:52 PM, merged with Solutions: Local_help (2)*

8 Quotations:

1:12 ¶ 26, She can use her children’s education to use the service in interview analysis / 1:103 ¶ 103, The thing is there are learned children in every household, so possibl… in interview analysis / 1:107 ¶ 106, Most women read the local language so if it can be in the local langua… in interview analysis / 1:108 ¶ 107, Teaching households to use phone especially to have successful pregnan… in interview analysis / 1:110 ¶ 109, Women’s education and society overall should be promoted to learn at l… in interview analysis / 1:111 ¶ 110, To get mothers using this program husband should take large responsibi… in interview analysis / 3:30 ¶ 17, The service indeed can be provided when any phone in house like spouse… in Group discussion / 3:71 ¶ 60, Maternal education can be improved through adult education (Golmasa ti… in Group discussion

3:71 ¶ 60 in Group discussion

Text quotation

Created by Girma Gilano on 3/9/2023

Maternal education can be improved through adult education (Golmasa timhert). Educating female as a community and using women forum to reach and aware mothers

3 Codes:

● Solutions: Family_help

Comment: by Girma Gilano

> *3/12/2023 1:51:52 PM, merged with Solutions: Local_help (2)*

8 Quotations:

1:12 ¶ 26, She can use her children’s education to use the service in interview analysis / 1:103 ¶ 103, The thing is there are learned children in every household, so possibl… in interview analysis / 1:107 ¶ 106, Most women read the local language so if it can be in the local langua… in interview analysis / 1:108 ¶ 107, Teaching households to use phone especially to have successful pregnan… in interview analysis / 1:110 ¶ 109, Women’s education and society overall should be promoted to learn at l… in interview analysis / 1:111 ¶ 110, To get mothers using this program husband should take large responsibi… in interview analysis / 3:30 ¶ 17, The service indeed can be provided when any phone in house like spouse… in Group discussion / 3:71 ¶ 60, Maternal education can be improved through adult education (Golmasa ti… in Group discussion

● Solutions: Forum_help

Comment: by Girma Gilano

> *3/12/2023 1:51:41 PM, merged with Solutions: Local_help 3/19/2023 2:59:22 AM, merged with Solutions: HDA*

7 Quotations:

1:11 ¶ 26, for uneducated we have to teach through mothers forum, community forum… in interview analysis / 1:107 ¶ 106, Most women read the local language so if it can be in the local langua… in interview analysis / 1:109 ¶ 108, sing mothers forum, HDA, voluntary health team(በጎ ጤና ቡድን), community w… in interview analysis / 1:112 ¶ 111, Creating conducive political environment, creating awareness, using mo… in interview analysis / 2:19 ¶ 32, Human development and health development army can improve all the limi… in KII / 3:71 ¶ 60, Maternal education can be improved through adult education (Golmasa ti… in Group discussion / 3:74 ¶ 63, Using voluntary health team in each community to promote mHealth impli… in Group discussion

- **Solutions: Professional_support**

9 Quotations:

1:13 ¶ 27, Rural mothers do not have road, transport, money, and motivation or ne… in interview analysis / 1:14 ¶ 28, It can change the community behavior and they may then become dependen… in interview analysis / 1:104 ¶ 104, We usually expect some challenges or struggle to make a new service br… in interview analysis / 3:22 ¶ 12, Except primi gravida, all para gravida mothers knew the consequence of… in Group discussion / 3:33 ¶ 19, Large queues are not disrupting counseling service provision because w… in Group discussion / 3:36 ¶ 20, We really do not know what we are doing while this mHealth may change… in Group discussion / 3:71 ¶ 60, Maternal education can be improved through adult education (Golmasa ti… in Group discussion / 3:72 ¶ 61, Using HEWs to reach to lost or bored mothers. Considering children for… in Group discussion / 3:73 ¶ 62, Increasing the maternal and child care to household level and intensiv… in Group discussion

---

Solutions: Forum_help

Created by Girma Gilano on 3/12/2023, **modified** by Girma Gilano on 3/19/2023

Comment: by Girma Gilano

> *3/12/2023 1:51:41 PM, merged with Solutions: Local_help 3/19/2023 2:59:22 AM, merged with Solutions: HDA*

7 Quotations:

1:11 ¶ 26 in interview analysis

Text quotation

Created by Girma Gilano on 3/8/2023

for uneducated we have to teach through mothers forum, community forum, and adult education

1 Codes:

- **Solutions: Forum_help**

Comment: by Girma Gilano

> *3/12/2023 1:51:41 PM, merged with Solutions: Local_help 3/19/2023 2:59:22 AM, merged with Solutions: HDA*

7 Quotations:

1:11 ¶ 26, for uneducated we have to teach through mothers forum, community forum… in interview analysis / 1:107 ¶ 106, Most women read the local language so if it can be in the local langua… in interview analysis / 1:109 ¶ 108, sing mothers forum, HDA, voluntary health team(በጎ ጤና ቡድን), community w… in interview analysis / 1:112 ¶ 111, Creating conducive political environment, creating awareness, using mo… in interview analysis / 2:19 ¶ 32, Human development and health development army can improve all the limi… in KII / 3:71 ¶ 60, Maternal education can be improved through adult education (Golmasa ti… in Group discussion / 3:74 ¶ 63, Using voluntary health team in each community to promote mHealth impli… in Group discussion

1:107 ¶ 106 in interview analysis

Text quotation

Created by Girma Gilano on 3/9/2023

Most women read the local language so if it can be in the local language it will be helpful. This means more than half of mothers can use this service. Almost every husband has a mobile and there taught children in every house to solve the maternal inability to read. A mobilizing community for maternal education and behavioral change is importan

2 Codes:

● Solutions: Family_help

Comment: by Girma Gilano

> *3/12/2023 1:51:52 PM, merged with Solutions: Local_help (2)*

8 Quotations:

1:12 ¶ 26, She can use her children's education to use the service in interview analysis / 1:103 ¶ 103, The thing is there are learned children in every household, so possibl… in interview analysis / 1:107 ¶ 106, Most women read the local language so if it can be in the local langua… in interview analysis / 1:108 ¶ 107, Teaching households to use phone especially to have successful pregnan… in interview analysis / 1:110 ¶ 109, Women's education and society overall should be promoted to learn at l… in interview analysis / 1:111 ¶ 110, To get mothers using this program husband should take large responsibi… in interview analysis / 3:30 ¶ 17, The service indeed can be provided when any phone in house like spouse… in Group discussion / 3:71 ¶ 60, Maternal education can be improved through adult education (Golmasa ti… in Group discussion

● Solutions: Forum_help

Comment: by Girma Gilano

> *3/12/2023 1:51:41 PM, merged with Solutions: Local_help 3/19/2023 2:59:22 AM, merged with Solutions: HDA*

7 Quotations:

1:11 ¶ 26, for uneducated we have to teach through mothers forum, community forum… in interview analysis / 1:107 ¶ 106, Most women read the local language so if it can be in the local langua… in interview analysis / 1:109 ¶ 108, sing mothers forum, HDA, voluntary health team(በጎ ጤና ቡድን), community w… in interview analysis / 1:112 ¶ 111, Creating conducive political environment, creating awareness, using mo… in interview analysis / 2:19 ¶ 32, Human development and health development army can improve all the limi… in KII / 3:71 ¶ 60, Maternal education can be improved through adult education (Golmasa ti… in Group discussion / 3:74 ¶ 63, Using voluntary health team in each community to promote mHealth impli… in Group discussion

1:109 ¶ 108 in interview analysis

Text quotation

Created by Girma Gilano on 3/9/2023

sing mothers forum, HDA, voluntary health team(በጎ ጤና ቡድን), community wing movement(የህዝብ ክንፍ ንቅናቄ), one-to-five (1 ለ 5), and using HEWs usual program. Mothers’ forum will be a nice thing to increase awareness of mothers. Using HEWs can also help the mother get aware of the mHealth care. In villages without electricity, there are solar centers for charging mobiles so no problem just we can support those centers

1 Codes:

- **Solutions: Forum_help**

Comment: by Girma Gilano

> *3/12/2023 1:51:41 PM, merged with Solutions: Local_help 3/19/2023 2:59:22 AM, merged with Solutions: HDA*

7 Quotations:

1:11 ¶ 26, for uneducated we have to teach through mothers forum, community forum… in interview analysis / 1:107 ¶ 106, Most women read the local language so if it can be in the local langua… in interview analysis / 1:109 ¶ 108, sing mothers forum, HDA, voluntary health team(በጎ ጤና ቡድን), community w… in interview analysis / 1:112 ¶ 111, Creating conducive political environment, creating awareness, using mo… in interview analysis / 2:19 ¶ 32, Human development and health development army can improve all the limi… in KII / 3:71 ¶ 60, Maternal education can be improved through adult education (Golmasa ti… in Group discussion / 3:74 ¶ 63, Using voluntary health team in each community to promote mHealth impli… in Group discussion

1:112 ¶ 111 in interview analysis

Text quotation

Created by Girma Gilano on 3/9/2023

Creating conducive political environment, creating awareness, using mothers forum appropriately, using voluntary health team of the kebeles, trying through health development army, using one-to-five political arrangement for information dissemination. When mothers take an intended service, previously there is a time when small hats provided for their children so may such an issues can be helpful if possible. And this promotes utilizations

1 Codes:

- **Solutions: Forum_help**

Comment: by Girma Gilano

> *3/12/2023 1:51:41 PM, merged with Solutions: Local_help 3/19/2023 2:59:22 AM, merged with Solutions: HDA*

7 Quotations:

1:11 ¶ 26, for uneducated we have to teach through mothers forum, community forum… in interview analysis / 1:107 ¶ 106, Most women read the local language so if it can be in the local langua… in interview analysis / 1:109 ¶ 108, sing mothers forum, HDA, voluntary health team(በጎ ጤና ቡድን), community w… in interview analysis / 1:112 ¶ 111, Creating conducive political environment, creating awareness, using mo… in interview analysis / 2:19 ¶ 32, Human development and health development army can improve all the limi… in KII / 3:71 ¶ 60, Maternal education can be improved through adult education (Golmasa ti… in Group discussion / 3:74 ¶ 63, Using voluntary health team in each community to promote mHealth impli… in Group discussion

2:19 ¶ 32 in KII

Text quotation

Created by Girma Gilano on 3/9/2023

Human development and health development army can improve all the limitation between HEWs, HC, and community

1 Codes:

- **Solutions: Forum_help**

Comment: by Girma Gilano

> *3/12/2023 1:51:41 PM, merged with Solutions: Local_help 3/19/2023 2:59:22 AM, merged with Solutions: HDA*

7 Quotations:

1:11 ¶ 26, for uneducated we have to teach through mothers forum, community forum… in interview analysis / 1:107 ¶ 106, Most women read the local language so if it can be in the local langua… in interview analysis / 1:109 ¶ 108, sing mothers forum, HDA, voluntary health team(በጎ ጤና ቡድን), community w… in interview analysis / 1:112 ¶ 111, Creating conducive political environment, creating awareness, using mo… in interview analysis / 2:19 ¶ 32, Human development and health development army can improve all the limi… in KII / 3:71 ¶ 60, Maternal education can be improved through adult education (Golmasa ti… in Group discussion / 3:74 ¶ 63, Using voluntary health team in each community to promote mHealth impli… in Group discussion

3:71 ¶ 60 in Group discussion

Text quotation

Created by Girma Gilano on 3/9/2023

Maternal education can be improved through adult education (Golmasa timhert). Educating female as a community and using women forum to reach and aware mothers

3 Codes:

- **Solutions: Family_help**

Comment: by Girma Gilano

> *3/12/2023 1:51:52 PM, merged with Solutions: Local_help (2)*

8 Quotations:

1:12 ¶ 26, She can use her children’s education to use the service in interview analysis / 1:103 ¶ 103, The thing is there are learned children in every household, so possibl… in interview analysis / 1:107 ¶ 106, Most women read the local language so if it can be in the local langua… in interview analysis / 1:108 ¶ 107, Teaching households to use phone especially to have successful pregnan… in interview analysis / 1:110 ¶ 109, Women’s education and society overall should be promoted to learn at l… in interview analysis / 1:111 ¶ 110, To get mothers using this program husband should take large responsibi… in interview analysis / 3:30 ¶ 17, The service indeed can be provided when any phone in house like spouse… in Group discussion / 3:71 ¶ 60, Maternal education can be improved through adult education (Golmasa ti… in Group discussion

● Solutions: Forum_help

Comment: by Girma Gilano

> *3/12/2023 1:51:41 PM, merged with Solutions: Local_help 3/19/2023 2:59:22 AM, merged with Solutions: HDA*

7 Quotations:

1:11 ¶ 26, for uneducated we have to teach through mothers forum, community forum… in interview analysis / 1:107 ¶ 106, Most women read the local language so if it can be in the local langua… in interview analysis / 1:109 ¶ 108, sing mothers forum, HDA, voluntary health team(በጎ ጤና ቡድን), community w… in interview analysis / 1:112 ¶ 111, Creating conducive political environment, creating awareness, using mo… in interview analysis / 2:19 ¶ 32, Human development and health development army can improve all the limi… in KII / 3:71 ¶ 60, Maternal education can be improved through adult education (Golmasa ti… in Group discussion / 3:74 ¶ 63, Using voluntary health team in each community to promote mHealth impli… in Group discussion

● Solutions: Professional_support

9 Quotations:

1:13 ¶ 27, Rural mothers do not have road, transport, money, and motivation or ne… in interview analysis / 1:14 ¶ 28, It can change the community behavior and they may then become dependen… in interview analysis / 1:104 ¶ 104, We usually expect some challenges or struggle to make a new service br… in interview analysis / 3:22 ¶ 12, Except primi gravida, all para gravida mothers knew the consequence of… in Group discussion / 3:33 ¶ 19, Large queues are not disrupting counseling service provision because w… in Group discussion / 3:36 ¶ 20, We really do not know what we are doing while this mHealth may change… in Group discussion / 3:71 ¶ 60, Maternal education can be improved through adult education (Golmasa ti… in Group discussion / 3:72 ¶ 61, Using HEWs to reach to lost or bored mothers. Considering children for… in Group discussion / 3:73 ¶ 62, Increasing the maternal and child care to household level and intensiv… in Group discussion

3:74 ¶ 63 in Group discussion

Text quotation

Created by Girma Gilano on 3/9/2023

Using voluntary health team in each community to promote mHealth implications, using every contact to promote the mHealth service, working in collaboration with other stakeholders for resources and support, and using politics/government in area of difficult to support community mobilization.

1 Codes:

Solutions: Forum_help

Comment: by Girma Gilano

> *3/12/2023 1:51:41 PM, merged with Solutions: Local_help 3/19/2023 2:59:22 AM, merged with Solutions: HDA*

7 Quotations:

1:11 ¶ 26, for uneducated we have to teach through mothers forum, community forum… in interview analysis / 1:107 ¶ 106, Most women read the local language so if it can be in the local langua… in interview analysis / 1:109 ¶ 108, sing mothers forum, HDA, voluntary health team(በጎ ጤና ቡድን), community w… in interview analysis / 1:112 ¶ 111, Creating conducive political environment, creating awareness, using mo… in interview analysis / 2:19 ¶ 32, Human development and health development army can improve all the limi… in KII / 3:71 ¶ 60, Maternal education can be improved through adult education (Golmasa ti… in Group discussion / 3:74 ¶ 63, Using voluntary health team in each community to promote mHealth impli… in Group discussion

---

Solutions: Professional_support

Created by Girma Gilano on 3/12/2023

9 Quotations:

1:13 ¶ 27 in interview analysis

Text quotation

Created by Girma Gilano on 3/8/2023

Rural mothers do not have road, transport, money, and motivation or need support. mHealth

1 Codes:

Solutions: Professional_support

9 Quotations:

1:13 ¶ 27, Rural mothers do not have road, transport, money, and motivation or ne… in interview analysis / 1:14 ¶ 28, It can change the community behavior and they may then become dependen… in interview analysis / 1:104 ¶ 104, We usually expect some challenges or struggle to make a new service br… in interview analysis / 3:22 ¶ 12, Except primi gravida, all para gravida mothers knew the consequence of… in Group discussion / 3:33 ¶ 19, Large queues are not disrupting counseling service provision because w… in Group discussion / 3:36 ¶ 20, We really do not know what we are doing while this mHealth may change… in Group discussion / 3:71 ¶ 60, Maternal education can be improved through adult education (Golmasa ti… in Group discussion / 3:72 ¶ 61, Using HEWs to reach to lost or bored mothers. Considering children for… in Group discussion / 3:73 ¶ 62, Increasing the maternal and child care to household level and intensiv… in Group discussion

1:14 ¶ 28 in interview analysis

Text quotation

Created by Girma Gilano on 3/8/2023

It can change the community behavior and they may then become dependent on mHealth

1 Codes:

- **Solutions: Professional_support**

9 Quotations:

1:13 ¶ 27, Rural mothers do not have road, transport, money, and motivation or ne… in interview analysis / 1:14 ¶ 28, It can change the community behavior and they may then become dependen… in interview analysis / 1:104 ¶ 104, We usually expect some challenges or struggle to make a new service br… in interview analysis / 3:22 ¶ 12, Except primi gravida, all para gravida mothers knew the consequence of… in Group discussion / 3:33 ¶ 19, Large queues are not disrupting counseling service provision because w… in Group discussion / 3:36 ¶ 20, We really do not know what we are doing while this mHealth may change… in Group discussion / 3:71 ¶ 60, Maternal education can be improved through adult education (Golmasa ti… in Group discussion / 3:72 ¶ 61, Using HEWs to reach to lost or bored mothers. Considering children for… in Group discussion / 3:73 ¶ 62, Increasing the maternal and child care to household level and intensiv… in Group discussion

1:104 ¶ 104 in interview analysis

Text quotation

Created by Girma Gilano on 3/9/2023

We usually expect some challenges or struggle to make a new service breakthrough and accepted safely by every profession, mothers, and community at large. Creating awareness and finding a person to look after them

1 Codes:

- **Solutions: Professional_support**

9 Quotations:

1:13 ¶ 27, Rural mothers do not have road, transport, money, and motivation or ne… in interview analysis / 1:14 ¶ 28, It can change the community behavior and they may then become dependen… in interview analysis / 1:104 ¶ 104, We usually expect some challenges or struggle to make a new service br… in interview analysis / 3:22 ¶ 12, Except primi gravida, all para gravida mothers knew the consequence of… in Group discussion / 3:33 ¶ 19, Large queues are not disrupting counseling service provision because w… in Group discussion / 3:36 ¶ 20, We really do not know what we are doing while this mHealth may change… in Group discussion / 3:71 ¶ 60, Maternal education can be improved through adult education (Golmasa ti… in Group discussion / 3:72 ¶ 61, Using HEWs to reach to lost or bored mothers. Considering children for… in Group discussion / 3:73 ¶ 62, Increasing the maternal and child care to household level and intensiv… in Group discussion

3:22 ¶ 12 in Group discussion

Text quotation

Created by Girma Gilano on 3/9/2023

Except primi gravida, all para gravida mothers knew the consequence of not using the counseling information in enough amounts. It is important to maintain that information flowing but behavioral change could be very important to solve this problem

1 Codes:

- **Solutions: Professional_support**

9 Quotations:

1:13 ¶ 27, Rural mothers do not have road, transport, money, and motivation or ne… in interview analysis / 1:14 ¶ 28, It can change the community behavior and they may then become dependen… in interview analysis / 1:104 ¶ 104, We usually expect some challenges or struggle to make a new service br… in interview analysis / 3:22 ¶ 12, Except primi gravida, all para gravida mothers knew the consequence of… in Group discussion / 3:33 ¶ 19, Large queues are not disrupting counseling service provision because w… in Group discussion / 3:36 ¶ 20, We really do not know what we are doing while this mHealth may change… in Group discussion / 3:71 ¶ 60, Maternal education can be improved through adult education (Golmasa ti… in Group discussion / 3:72 ¶ 61, Using HEWs to reach to lost or bored mothers. Considering children for… in Group discussion / 3:73 ¶ 62, Increasing the maternal and child care to household level and intensiv… in Group discussion

3:33 ¶ 19 in Group discussion

Text quotation

Created by Girma Gilano on 3/9/2023

Large queues are not disrupting counseling service provision because we have another chance to get the mother

1 Codes:

- **Solutions: Professional_support**

9 Quotations:

1:13 ¶ 27, Rural mothers do not have road, transport, money, and motivation or ne… in interview analysis / 1:14 ¶ 28, It can change the community behavior and they may then become dependen… in interview analysis / 1:104 ¶ 104, We usually expect some challenges or struggle to make a new service br… in interview analysis / 3:22 ¶ 12, Except primi gravida, all para gravida mothers knew the consequence of… in Group discussion / 3:33 ¶ 19, Large queues are not disrupting counseling service provision because w… in Group discussion / 3:36 ¶ 20, We really do not know what we are doing while this mHealth may change… in Group discussion / 3:71 ¶ 60, Maternal education can be improved through adult education (Golmasa ti… in Group discussion / 3:72 ¶ 61, Using HEWs to reach to lost or bored mothers. Considering children for… in Group discussion / 3:73 ¶ 62, Increasing the maternal and child care to household level and intensiv… in Group discussion

3:36 ¶ 20 in Group discussion

Text quotation

Created by Girma Gilano on 3/9/2023

We really do not know what we are doing while this mHealth may change this so that we can know every one we serve.

1 Codes:

- **Solutions: Professional_support**

9 Quotations:

1:13 ¶ 27, Rural mothers do not have road, transport, money, and motivation or ne… in interview analysis / 1:14 ¶ 28, It can change the community behavior and they may then become dependen… in interview analysis / 1:104 ¶ 104, We usually expect some challenges or struggle to make a new service br… in interview analysis / 3:22 ¶ 12, Except primi gravida, all para gravida mothers knew the consequence of… in Group discussion / 3:33 ¶ 19, Large queues are not disrupting counseling service provision because w… in Group discussion / 3:36 ¶ 20, We really do not know what we are doing while this mHealth may change… in Group discussion / 3:71 ¶ 60, Maternal education can be improved through adult education (Golmasa ti… in Group discussion / 3:72 ¶ 61, Using HEWs to reach to lost or bored mothers. Considering children for… in Group discussion / 3:73 ¶ 62, Increasing the maternal and child care to household level and intensiv… in Group discussion

3:71 ¶ 60 in Group discussion

Text quotation

Created by Girma Gilano on 3/9/2023

Maternal education can be improved through adult education (Golmasa timhert). Educating female as a community and using women forum to reach and aware mothers

3 Codes:

- **Solutions: Family_help**

Comment: by Girma Gilano

> *3/12/2023 1:51:52 PM, merged with Solutions: Local_help (2)*

8 Quotations:

1:12 ¶ 26, She can use her children's education to use the service in interview analysis / 1:103 ¶ 103, The thing is there are learned children in every household, so possibl… in interview analysis / 1:107 ¶ 106, Most women read the local language so if it can be in the local langua… in interview analysis / 1:108 ¶ 107, Teaching households to use phone especially to have successful pregnan… in interview analysis / 1:110 ¶ 109, Women's education and society overall should be promoted to learn at l… in interview analysis / 1:111 ¶ 110, To get mothers using this program husband should take large responsibi… in interview analysis / 3:30 ¶ 17, The service indeed can be provided when any phone in house like spouse… in Group discussion / 3:71 ¶ 60, Maternal education can be improved through adult education (Golmasa ti… in Group discussion

- **Solutions: Forum_help**

Comment: by Girma Gilano

> *3/12/2023 1:51:41 PM, merged with Solutions: Local_help 3/19/2023 2:59:22 AM, merged with Solutions: HDA*

7 Quotations:

1:11 ¶ 26, for uneducated we have to teach through mothers forum, community forum… in interview analysis / 1:107 ¶ 106, Most women read the local language so if it can be in the local langua… in interview analysis / 1:109 ¶ 108, sing mothers forum, HDA, voluntary health team(በጎ ጤና ቡድን), community w… in interview analysis / 1:112 ¶ 111, Creating conducive political environment, creating awareness, using mo… in interview analysis / 2:19 ¶ 32, Human development and health development army can improve all the limi… in KII / 3:71 ¶ 60, Maternal education can be improved through adult education (Golmasa ti… in Group discussion / 3:74 ¶ 63, Using voluntary health team in each community to promote mHealth impli… in Group discussion

- **Solutions: Professional_support**

9 Quotations:

1:13 ¶ 27, Rural mothers do not have road, transport, money, and motivation or ne… in interview analysis / 1:14 ¶ 28, It can change the community behavior and they may then become dependen… in interview analysis / 1:104 ¶ 104, We usually expect some challenges or struggle to make a new service br… in interview analysis / 3:22 ¶ 12, Except primi gravida, all para gravida mothers knew the consequence of… in Group discussion / 3:33 ¶ 19, Large queues are not disrupting counseling service provision because w… in Group discussion / 3:36 ¶ 20, We really do not know what we are doing while this mHealth may change… in Group discussion / 3:71 ¶ 60, Maternal education can be improved through adult education (Golmasa ti… in Group discussion / 3:72 ¶ 61, Using HEWs to reach to lost or bored mothers. Considering children for… in Group discussion / 3:73 ¶ 62, Increasing the maternal and child care to household level and intensiv… in Group discussion

3:72 ¶ 61 in Group discussion

Text quotation

Created by Girma Gilano on 3/9/2023

Using HEWs to reach to lost or bored mothers. Considering children for those mother are not learned. Additionally, use of spouse phone is a big alternative, Involving partner in maternal and child care, and use of children phone

1 Codes:

- **Solutions: Professional_support**

9 Quotations:

1:13 ¶ 27, Rural mothers do not have road, transport, money, and motivation or ne… in interview analysis / 1:14 ¶ 28, It can change the community behavior and they may then become dependen… in interview analysis / 1:104 ¶ 104, We usually expect some challenges or struggle to make a new service br… in interview analysis / 3:22 ¶ 12, Except primi gravida, all para gravida mothers knew the consequence of… in Group discussion / 3:33 ¶ 19, Large queues are not disrupting counseling service provision because w… in Group discussion / 3:36 ¶ 20, We really do not know what we are doing while this mHealth may change… in Group discussion / 3:71 ¶ 60, Maternal education can be improved through adult education (Golmasa ti… in Group discussion / 3:72 ¶ 61, Using HEWs to reach to lost or bored mothers. Considering children for… in Group discussion / 3:73 ¶ 62, Increasing the maternal and child care to household level and intensiv… in Group discussion

3:73 ¶ 62 in Group discussion

Text quotation

Created by Girma Gilano on 3/9/2023

Increasing the maternal and child care to household level and intensive health education to ensure women aware of her problems. Community mobilization to motivate women to use mHealth might be also important. Using community wing political office to educate, motivate women and community can be used

1 Codes:

- **Solutions: Professional_support**

9 Quotations:

1:13 ¶ 27, Rural mothers do not have road, transport, money, and motivation or ne… in interview analysis / 1:14 ¶ 28, It can change the community behavior and they may then become dependen… in interview analysis / 1:104 ¶ 104, We usually expect some challenges or struggle to make a new service br… in interview analysis / 3:22 ¶ 12, Except primi gravida, all para gravida mothers knew the consequence of… in Group discussion / 3:33 ¶ 19, Large queues are not disrupting counseling service provision because w… in Group discussion / 3:36 ¶ 20, We really do not know what we are doing while this mHealth may change… in Group discussion / 3:71 ¶ 60, Maternal education can be improved through adult education (Golmasa ti… in Group discussion / 3:72 ¶ 61, Using HEWs to reach to lost or bored mothers. Considering children for… in Group discussion / 3:73 ¶ 62, Increasing the maternal and child care to household level and intensiv… in Group discussion
