# Supplementary material for: The role of mHealth intervention to improve maternal and child health: A provider-based qualitative study in Southern Ethiopia
Source: PLoS One. 2024 Feb 8;19(2):e0295539. doi: 10.1371/journal.pone.0295539 (PMC10852240; doi:10.1371/journal.pone.0295539)
Supplement: S1 File — (ZIP) [file pone.0295539.s003.zip › supplementars evidences/table 2 subcodes.pdf]

Table 3: Subthemes and their relationship with the source of data

Code Co-occurrence Analysis | Code Manager | Code-Document Analysis

Code	Count	1: interview... (107)	2: KII (45)	3: Group dis... (74)	Totals
Acceptability	28	8	15	5	28
Acceptability: Fiting_current	9	4	3	2	9
Acceptability: Improve_care	2	1	1		2
Acceptability: support_professionals	10	2	6	2	10
Acceptability: Technology_care	13	1	7	5	13
Awareness creation	24	10	4	10	24
Awareness creation: community_awarness	8	2	2	4	8
Awareness creation: family_awareness	4	1		3	4
Awareness creation: provider_awarness	4	2	1	1	4
Awareness creation: women_awarness	19	8	3	8	19
Benefits of mHealth	82	38	9	35	82
Benefits of mHealth: ALarming	6	4		2	6
Benefits of mHealth: Effective	6	5	1		6
Benefits of mHealth: Help_mothers	27	8	5	14	27
Benefits of mHealth: Help_professional	30	7	4	19	30
Benefits of mHealth: Improve_decision	17	8	1	8	17
Benefits of mHealth: Improve_MCH	25	14	2	9	25
Challenges of mHealth impleentation	68	36	16	16	68
Challenges of mHealth impleentation: Accep...	17	6	7	4	17
Challenges of mHealth impleentation: Awar...	11	7	2	2	11
Challenges of mHealth impleentation: Devic...	12	8	1	3	12
Challenges of mHealth impleentation: neglig...	7	4		3	7
Challenges of mHealth impleentation: Readi...	15	5	6	4	15
Challenges of mHealth impleentation: Security	6	6			6
Challenges of mHealth impleentation: workl...	7	4		3	7
Solutions	21	12	1	8	21
Solutions: Family_help	8	6		2	8
Solutions: Forum_help	7	4	1	2	7
Solutions: Professional_support	9	3		6	9
Totals		224	98	180	502

NB: KII=Key informants' in-depth interview; MCH=Mother and Child Health HDA= Health Development Army
