# Supplementary figures and images for: The role of mHealth intervention to improve maternal and child health: A provider-based qualitative study in Southern Ethiopia
Source: PLoS One. 2024 Feb 8;19(2):e0295539. doi: 10.1371/journal.pone.0295539 (PMC10852240; doi:10.1371/journal.pone.0295539)

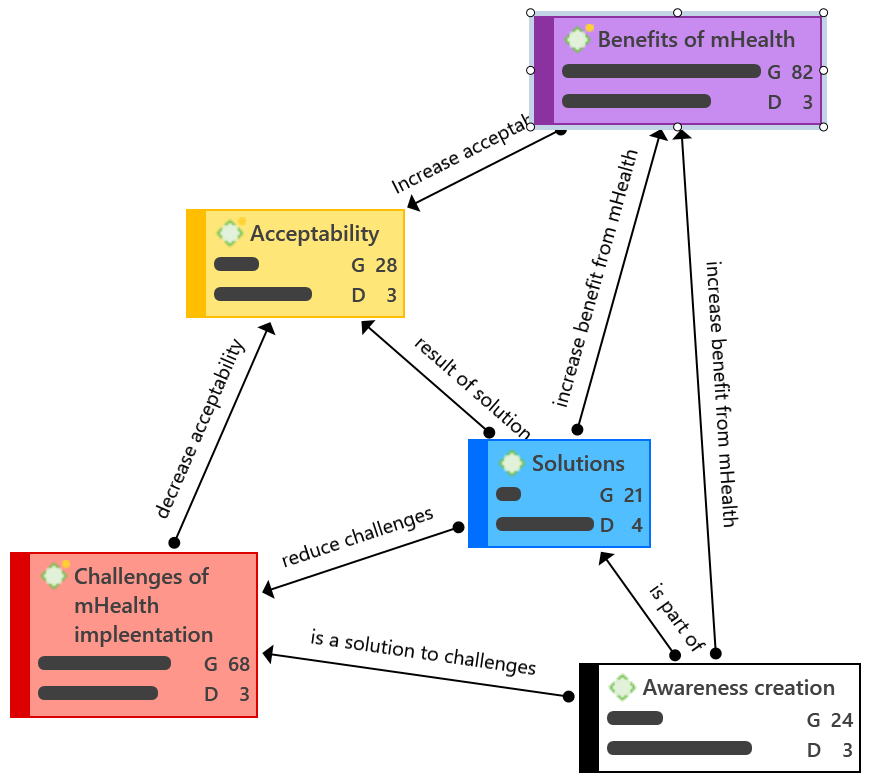

Supplement: S1 File — (ZIP) [file pone.0295539.s003.zip › supplementars evidences/New Network1.png]
