# Supplementary material for: The role of mHealth intervention to improve maternal and child health: A provider-based qualitative study in Southern Ethiopia
Source: PLoS One. 2024 Feb 8;19(2):e0295539. doi: 10.1371/journal.pone.0295539 (PMC10852240; doi:10.1371/journal.pone.0295539)
Supplement: Attachment — Submitted filename: Response to Reviewers.docx [file pone.0295539.s004.docx]

**Editorial comments**

**Comments**: We note that the grant information you provided in the ‘Funding Information’ and ‘Financial Disclosure’ sections do not match.

**Response**: Thank you for the comment. we insert the grant number in Financial Disclosure section.

**Comments**: Please include your full ethics statement in the ‘Methods’ section of your manuscript file. In your statement, please include the full name of the IRB or ethics committee who approved or waived your study, as well as whether or not you obtained informed written or verbal consent. If consent was waived for your study, please include this information in your statement as well.

**Response**: Thank you for the comment. We improved ethical approval (page 7, line195-196)

**Comments**: Please ensure that you refer to Figure 1 in your text as, if accepted, production will need this reference to link the reader to the figure.

**Responses**: Thank you for the comment. There is no figure in the document anymore.

 We recommend that you contact the original copyright holder with the Content Permission Form (http://journals.plos.org/plosone/s/file?id=7c09/content-permission-form.pdf) and the following text:

**Reviewers’ comments**

**Reviewer#1**

**Comment**: Define what mHealth is. Readers might have no idea what you are talking about.

**Response**: Thank you for the comments. We defined mHealth (page 3, line 81-84)

**Comment**: Introduce the complete word for mHealth (Mobile health) for the first time that you used this abbreviation.
**Responses**: Thank you for the comments. We defined mHealth (page 3, line 81)

**Comment**: The paper misses a good and comprehensive related work section. Currently, related work is limited and mixed with introduction. I think it would be better if you separate the related work from the introduction, create a section specific to the related work, and completely explain the applications and benefits that mHealth solutions have had in this subject.

**Response**: Thank you for the comments. We completely modified the introduction section short and precise (page 3-4, line 69-107)

**Comment**: Did you conduct the interviews and FGD in-person or online? Please explain this in the paper.
**Response**: Thank you for the comments. Yes, it is presented under Source Population and sample size section (page 5, line 141-151)
**Comment**: I did not understand what this number is in front of the quotes e.g. (II 4.1) or (KII 7.2). I think it would be better if you write (KII, his/her profession, sex, age)
**Response**: Thank you for the comments. Of course the numbers are the reference to the source code in the original source. They are traceable. We appreciate your comments and changed this format (page 10-17 line 237-495)
**Comment**: What is a mother forum? Is it some kind of in-person meetings that are held for the mothers? Please explain that in the paper.

**Responses**: Thank you for the comments. We added mothers forum definition(page 12, line 296-298)

**comment**s: The discussion needs to be revised. Now, it is more like the results section, like you are reporting the results again. You need to interpret your results with a broad perspective with the help of others’ work in the discussion section.

Please read this instruction for rewriting the discussion.
"The discussion reviews the findings and puts them into the context of the overall research. It brings together all the sections that came before it and allows a reader to see the connections
between each part of the research paper. In a discussion section, the author engages in three necessary steps: interpretation, analysis, and explanation. An effective discussion section will tell
a reader why the research results are important and where they fit in the current literature, while also being self-critical and candid about the shortcomings of the study."

**Responses**: Thank you for the comments. The discussion is completely changed and more sound now.
