# Supplementary material for: The role of mHealth intervention to improve maternal and child health: A provider-based qualitative study in Southern Ethiopia
Source: PLoS One. 2024 Feb 8;19(2):e0295539. doi: 10.1371/journal.pone.0295539 (PMC10852240; doi:10.1371/journal.pone.0295539)
Supplement: Attachment — Submitted filename: Reviewer Comments for Authors.docx [file pone.0295539.s005.docx]

Reviewer #1: In this paper, the authors conducted a qualitative study on health professionals' perspectives towards the role of mHealth in improving maternal and child health in Southern Ethiopia. The paper is well-written and easy to follow, addressing an interesting subject with great potential for facilitating the adoption of mHealth in developing countries like Ethiopia. However, there are some areas that need improvement and clarification:

1- Define what mHealth is. Readers might have no idea what you are talking about.

2- Introduce the complete word for mHealth (Mobile health) for the first time that you used this abbreviation.

3- The paper misses a good and comprehensive related work section. Currently, related work is limited and mixed with introduction. I think it would be better if you separate the related work from the introduction, create a section specific to the related work, and completely explain the applications and benefits that mHealth solutions have had in this subject.

4- Did you conduct the interviews and FGD in-person or online? Please explain this in the paper.

5- I did not understand what this number is in front of the quotes e.g. (II 4.1) or (KII 7.2). I think it would be better if you write (KII, his/her profession, sex, age)

5- What is a mother forum? Is it some kind of in-person meetings that are held for the mothers? Please explain that in the paper.

6- The discussion needs to be revised. Now, it is more like the results section, like you are reporting the results again. You need to interpret your results with a broad perspective with the help of others’ work in the discussion section.

Please read this instruction for rewriting the discussion.
"The discussion reviews the findings and puts them into the context of the overall research. It brings together all the sections that came before it and allows a reader to see the connections
between each part of the research paper. In a discussion section, the author engages in three necessary steps: interpretation, analysis, and explanation. An effective discussion section will tell
a reader why the research results are important and where they fit in the current literature, while also being self-critical and candid about the shortcomings of the study."
